# Stereoselective hydrogen atom transfer to acyclic radicals: a switch enabling diastereodivergent borylative radical cascades

**DOI:** 10.1038/s41467-022-28071-8

**Published:** 2022-01-20

**Authors:** Tian Ye, Feng-Lian Zhang, Hui-Min Xia, Xi Zhou, Zhi-Xiang Yu, Yi-Feng Wang

**Affiliations:** 1 Department of Chemistry, University of Science and Technology of China, 96 Jinzhai Road, 230026 Hefei, Anhui China; 2 Beijing National Laboratory for Molecular Sciences (BNLMS), Key Laboratory of Bioorganic Chemistry and Molecular Engineering of Ministry of Education, College of Chemistry, Peking University, 100871 Beijing, China; 3 State Key Laboratory of Elemento-Organic Chemistry, Nankai University, 300071 Tianjin, China

**Keywords:** Stereochemistry, Synthetic chemistry methodology

## Abstract

Radical cascade reactions are powerful tools to construct structurally complex molecules. However, the stereochemical control of acyclic radical intermediates remains a persistent challenge, due to the low differentiation between the two faces of these species. This hurdle further makes stereodivergent synthesis rather more difficult to be accomplished, in particular for intermediates resulted from complex cascades. Here we report an efficient strategy for stereoselective hydrogen atom transfer (HAT) to acyclic carbon radicals, which are generated via *N*-heterocyclic carbene (NHC)-boryl radicals triggered addition-translocation-cyclization cascades. A synergistic control by the NHC subunit and a thiol catalyst has proved effective for one facial HAT, while a ZnI_2_-chelation protocol allows for the preferential reaction to the opposite face. Such a stereoselectivity switch enables diastereodivergent construction of heterocycles tethering a boron-substituted stereocenter. Mechanistic studies suggest two complementary ways to tune HAT diastereoselectivity. The stereospecific conversions of the resulting boron-handled products to diverse functionalized molecules are demonstrated.

## Abstract (web summary)

Stereochemical control of acyclic carbon radical intermediates remains a challenge, due to the difficulty in differentiation between the two faces of the planar species. Here the authors show diastereodivergent hydrogen atom transfer to acyclic carbon radicals via control of N-heterocyclic carbene–borane reagents with either a thiol catalyst or a Lewis acid.

## Introduction

Construction of complex organic molecules in step- and atom-economic manners with high levels of stereoselectivity control is a preeminent goal in synthetic chemistry^1,2^. Radical cascade reactions represent one of the most efficient strategies to assemble densely functionalized architectures from simple starting materials in one step^3–5^. However, the stereochemical control in these reactions remains an inherently difficult issue when acyclic carbon stereocenters are created through complex cascade processes, due to the persistent challenge associated with the low differentiation between the two faces of acyclic alkyl radicals^6,7^. This hurdle also makes the stereodivergent synthesis, which is highly demanded in medicinal studies^8^, rather difficult to be achieved.

**Fig. 1:**
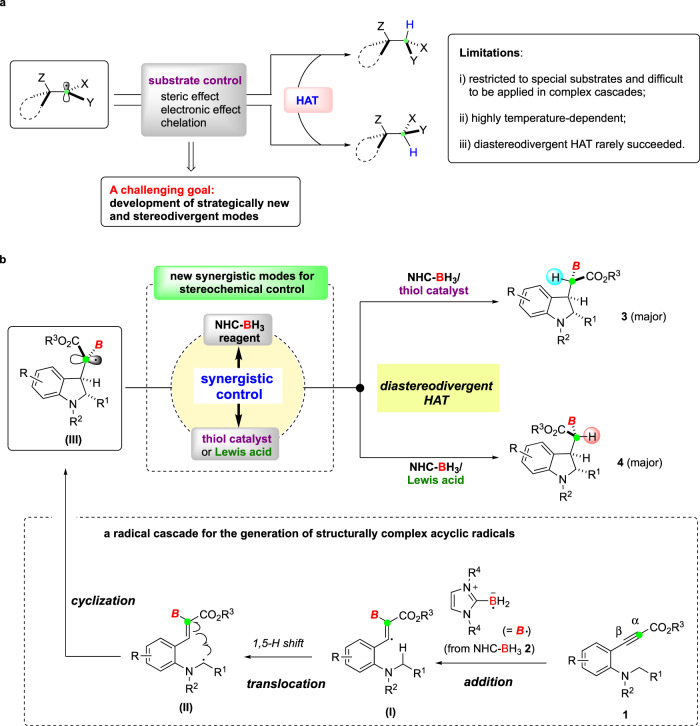
Diastereoselective HAT to acyclic radicals and the applications in synthesis. **a** Reported diastereoselective HAT of acyclic radicals methods mainly rely on substrate control. **b** This work: diastereodivergent HAT of structurally complex radical intermediates synergistically controlled by NHC-BH_3_ reagent with either a thiol catalyst or a Lewis acid. NHC *N*-heterocyclic carbene, HAT hydrogen atom transfer.

Hydrogen atom transfer (HAT) reactions often serve as a key step to terminate a radical cascade sequence through the reduction of the resulting radical intermediate^9–11^. However, HAT to acyclic radical intermediates with high levels of diastereoselectivity is a subject of persistent challenge^12–15^. In this context, some stereoselective HAT approaches have been reported, which include, for example, introducing different steric or electronic interactions in acyclic substrates (Fig. 1a). In addition, chelation by the use of Lewis acids has been applied so that the corresponding acyclic systems become rigid and high diastereoselectivity is consequently possible to be realized^16,17^. Although these approaches have proven effective in certain cases, some limitations and drawbacks still remain: (i) most methods are restricted to specific substrates with the pre-installation of different functional groups onto specific positions for steric, electronic, or complexation purposes, but such tactics are hardly applied in the reduction of radicals derived from complex radical cascades; (ii) stereocontrol is highly temperature-dependent and relatively lower temperature (usually below room temperature) is often necessary to ensure high diastereoselectivity, but this becomes disfavored for radical cascades that need high temperature^18,19^; (iii) diastereodivergent HAT reactions have only been reported in limited cases^20^, and a general strategy for diastereodivergency is still lacking. Therefore, developing new strategies that can render the reduction of acyclic radical intermediates to be stereoselectively distinguishable and switchable is highly desirable for advancing radical chemistry in synthesis.

*N*-heterocyclic carbene (NHC)-borane complexes have recently been developed as an important class of hydrogen atom donor, as pioneered by Fensterbank, Lacôte, Malacria, and Curran^21^. These complexes feature easy accessibility, low toxicity, and broad applicability. Moreover, the group of Curran and Taniguchi^22–25^, our group^26–29^, and others^30–33^ disclosed that these complexes are viable for the synthesis of organoboron molecules via radical cascade reactions, wherein both the boryl moiety and hydrogen atom are transferred to products. A polarity reversal catalyst^34,35^, such as thiol compound, is sometimes required to maintain efficient chain propagation. It is known that NHC molecules with various substitutions and stereochemistry are either commercially available or can be synthesized readily. Therefore, the NHC subunit would be considered as an important and tunable element for stereocontrol, where various potential factors, including steric or electronic effect, weak interactions between catalysts or substrates, and complexation with Lewis acid, would be used to modulate HAT stereoselectivity. Meanwhile, the NHC-boryl moiety is installed during the reaction process, thereby avoiding the prior installation of specific stereoselectivity-determining functionalities into substrates, which is desired in complex radical cascade reactions.

With this in mind, we hypothesized an NHC-boryl radical triggered radical addition-translocation-cyclization (RATC) cascade^36–39^ that could generate a boron-substituted structurally complex acyclic radical intermediate. As shown in Fig. 1b, the reaction starts with the α-addition of an NHC-boryl radical to arylpropiolates **1**^28^, followed by 1,5-hydrogen atom shift and intramolecular 5-*exo* cyclization, yielding an indoline ring with *trans*-stereochemistry of cyclic substituents. The following HAT to the resulting acyclic radical **III** with high stereocontrol is perceived to be a major challenge for achieving the goal of stereoselective and stereodivergent synthesis of boron-tethered indolines.

Here we report our realization of diastereoselective HAT to this acyclic radical, which is accomplished by a synergistic control of NHC-BH_3_ reagents together with either a thiol catalyst or a Lewis acid. Notably, these two modes operate in complementary ways that offer a stereochemistry switch to enable diastereodivergent synthesis of borylated products **3** and **4**. Mechanistic investigations of the detailed mechanisms are carried out to understand how the present HAT reactions take place and the origins of stereochemistry. Significantly, the formed boron-tethered indoline derivatives would be of great interest in synthetic and medicinal chemistry, given the fact that indoline is a core motif in numerous biologically relevant molecules^40,41^. Moreover, the boron moiety can be used in stereospecific diversifications^42–44^, providing direct and economical accesses to various functionalities through late-stage functionalizations, thus circumventing the traditional lengthy de novo synthesis.

## Results

### NHC-BH_3_/thiol catalyst-controlled stereoselective HAT to acyclic radicals generated via borylative RATC

We commenced our study by investigating the reaction of **1a** and NHC-BH_3_ **2a** in the presence of di-*tert*-butyl hyponitrite (TBHN) as the radical initiator. The reaction took place as expected, affording diastereomers **3a-Me** and **4a-Me** in 52% combined yield and a 67:33 diastereomeric ratio (dr) (Table 1, entry 1). Guindon has found that the exocyclic effect plays a vital role in the stereoselective reduction of carbon-centered radicals locating *exo* to a heterocycle^14^. However, the low dr value obtained in our reaction suggested that such an effect was not significant for the reduction of radical **III**.

**Table 1:** Optimization of the reaction conditions^a^.


Entry	2	Initiator	RSH	3a + 4a	3a:4a
				Yield (%)^b^	dr^c^
1	**2a**	TBHN	–	52	67:33
2	**2b**	TBHN	–	12	83:17
3	**2b**	TBHN	**A**	79	90:10
4	**2b**	TBHN	**B**	52	90:10
5	**2b**	TBHN	**C**	79	92:8
6	**2b**	TBHN	**D**	85	94:6
7	**2b**	TBHN	**E**	85	93:7
8	**2b**	TBHN	**F**	80	93:7
9	**2b**	TBHN	**G**	82	88:12
10	**2b**	TBHN	**H**	93	95:5
11	**2a**	TBHN	**H**	74	88:12
12	**2c**	TBHN	**H**	trace	ND
13^d^	**2b**	ABVN	**H**	93^e^	95:5
14^f^	**2b**	AIBN	**H**	85	91:9
15^g^	**2b**	ACCN	**H**	85	87:13
16	**2b**	–	**H**	No reaction (**1a**: 95%)	

^a^Reaction conditions: **1a** (0.2–0.3 mmol), **2** (1.2 equiv), radical initiator (0.2 equiv), RSH (0.2 equiv), toluene (2–3 mL), 50 °C for 3 h. *ND* not detected, *TBHN* di-*tert*-butyl hyponitrite, *ABVN* 2,2-azobisisoheptonitrile, *AIBN* azobisisobutyronitrile, *ACCN* azobiscyclohexanecarbonitrile.

^b^NMR yield using tetrachloroethane as an internal standard.

^c^dr was determined by ^1^H NMR analysis of the crude reaction mixture.

^d^The reaction was run at 60 °C.

^e^Isolated yield.

^f^The reaction was run at 80 °C.

^g^The reaction was run at 95 °C.

Next, we sought to examine various NHC-BH_3_ complexes and thiol catalysts to improve the diastereoselectivity. The use of **2b** instead of **2a** led to a lower conversion, but with a slightly increased dr (entry 2). Interestingly, when a thiol compound (thiol **A**, **B**, or **C**) was employed as a polarity reversal catalyst^34,35^, both the product yield (52–79%) and stereoselectivity (dr, 90:10-92:8) were enhanced (entries 3–5). Furthermore, using *ortho*-substituted thiophenols increased the dr (93:7 to 94:6) while maintaining high product yields (entries 6-8). However, 2,4,6-triisopropyl substituted thiol **G** resulted in a much lower dr (88:12) although good product yield could be retained (entry 9). Eventually, thiol **H** was found to be optimal, furnishing **3a-*i*-Pr** in excellent yield and dr (entry 10). Using thiol **H** as the catalyst, the reaction with **2a** resulted in a decrease of dr to 88:12 (entry 11), implying that the isopropyl group of NHC-BH_3_ **2b** is also crucial to maintain high diastereoselectivity. The reaction of **2c** bearing a 2,6-diisopropylphenyl motif led to nearly no reaction (entry 12), presumably due to the increased difficulty in the addition step. These findings support a fact that the stereoselectivity of HAT to acyclic radical **III** is cooperatively controlled by the substituents of both NHC-BH_3_ and the thiol catalyst. When the radical initiator was changed to 2,2-azobisisoheptonitrile (ABVN), **3a-*i*-Pr** was formed in 93% yield and 95:5 dr (entry 13). However, the employment of azobisisobutyronitrile (AIBN) and azobiscyclohexanecarbonitrile (ACCN), which require elevated temperature (80–95 °C) for initiation, caused decreased diastereoselectivity (entries 14–15). The control experiment showed that no reaction occurred in the absence of a radical initiator (entry 16), verifying a radical reaction mechanism of this transformation.

**Fig. 2:**
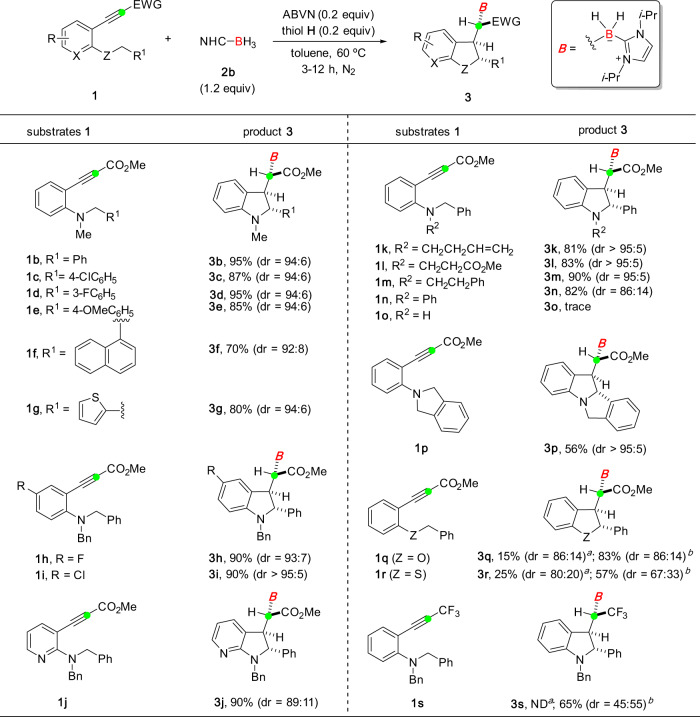
NHC-BH_3_/thiol catalyst-controlled stereoselective synthesis of borylated molecules derived from RATC. Reactions were performed with substrate **1** (0.2–0.5 mmol), **2b** (1.2 equiv), ABVN (0.2 equiv), and thiol **H** (0.2 equiv) in toluene (2–3 mL) at 60 °C for 3–12 h. The yield is recorded for the combination of diastereomers **3** and **4**. dr refers to the ratio of **3**:**4**. ND, not detected. ^a^Results obtained with the reaction conducted under standard reaction conditions and a competing hydroboration reaction of the alkyne moiety was observed. ^b^Results obtained with the reaction conducted using **2a** (1.2 equiv) and ACCN (0.5 equiv) at 95 °C.

The generality of this NHC-BH_3_/thiol catalyst-controlled diastereoselective synthesis of product **3** was examined (Fig. 2). In most cases, high levels of diastereoselectivity (dr >92:8) could be obtained. An array of functional groups on the 2-aryl ring (for **3c**–**3e**) and indoline framework (for **3h** and **3i**) were incorporated. Both naphthyl (for **3f**) and thiophenyl (for **3g**) motifs were compatible. The reaction of **1j** delivered a pyrrolo[2,3-*b*]pyridine framework (for **3j**), albeit with a slightly decreased diastereoselectivity. A series of alkyl groups could be used as the R^2^ substituent (for **1k**–**1m**) on the nitrogen atom, furnishing desired products in both excellent yields and diastereoselectivity. When the R^2^ substituent was replaced with a phenyl ring, the reaction could produce the expected product **3n** in 82% yield, but with a moderate stereoselectivity (86:14 dr). However, no reaction occurred for the hydrogen substituted one (for **1o**). The present protocol allowed for the construction of a tetracyclic framework (for **3p**) with no detrimental effect on diastereoselectivity. The borylative cascade reactions of **1q** and **1r**, however, became less efficient (<25% yield of cyclized products) and hydroboration of the alkyne moiety^23,45,46^ was observed as a competing reaction pathway. This is presumably due to the disfavored 1,5-hydrogen atom transfer of the corresponding alkenyl radical **I** that makes the reduction by the thiol catalyst possible. To our delight, the desired cascade reactions could be promoted by increasing the reaction temperature to 95 °C with the use of ACCN as the radical initiator and using **2a** as the boryl radical precursor, furnishing products **3q** and **3r** in 83% yield (86:14 dr) and 57% yield (67:33 dr), respectively. In these transformations, **2a** itself also acted as the hydrogen atom donor to maintain the radical chain process^47^. It was also found that the addition of a thiol catalyst was not effective at improving the dr value in this case (see Table S4 in the Supplementary Information). Likewise, the standard reaction protocol of **1s** led to no reaction, while the modified procedure provided the borylated product in 65% yield, albeit with low diastereoselectivity.

### NHC-BH_3_/Lewis acid-controlled stereoselective HAT to acyclic radicals generated via borylative RATC

Next, we questioned whether it is possible to reverse the stereoselectivity of the HAT process by changing the reaction conditions, leading to the preferential formation of diastereomers **4**. Considering that acyclic radical **III** contains some coordinative functionalities, such as methoxycarbonyl group, NHC subunit, and amino group, a range of Lewis acids have been examined. Eventually, it was found that the addition of ZnI_2_ as a chelating reagent and the employment of **2d** as the boryl radical precursor could afford **4aa** in good yield and excellent diastereoselectivity (**3aa**:**4aa** = 6:94, Table 2, entry 1). No thiol catalyst was required in this reaction, implying that acyclic radical **III** should abstract a hydrogen atom from **2d** to propagate the radical chain process^47^. In this case, the formed radical intermediate **III** likely became more electrophilic by coordination of ZnI_2_ to the carbonyl group, thus promoting the HAT process by more polarity matching. Reducing the amount of ZnI_2_ resulted in a dramatic decrease of dr (entries 2 and 3), and the reaction without ZnI_2_ even gave **3aa** as the major product (entry 4). Other Lewis acids such as MgCl_2_, MgI_2_, CuI, ZnCl_2_, Me_3_Al, and MgBr_2_·OEt_2_^48^ were found to be ineffective (entries 5-10). When **2a** was used as the radical precursors instead of **2d**, the dr value was retained, although the yield was decreased to 37% (entry 11). The reaction of **2b** led to only a trace amount of product (entry 12). These results indicate that the ZnI_2_ coordination with the triazole moiety of **2d** may be beneficial to maintain the radical chain propagation, but the contribution in controlling the diastereoselectivity is not significant.

**Table 2:** Examination of reaction parameters for the preferential formation of diastereomer **4aa**^a^.


Entry	Variation of reaction conditions	3aa + 4aa	3aa:4aa
		Yield (%)^b^	dr^c^
1	None	76^d^	6:94
2	ZnI_2_ (0.5 equiv) was used	82	12:88
3	ZnI_2_ (0.2 equiv) was used	62	50:50
4	Without ZnI_2_	57	75:25
5	MgCl_2_ instead of ZnI_2_	48	75:25
6	MgI_2_ instead of ZnI_2_	44	67:33
7	CuI instead of ZnI_2_	40	75:25
8	ZnCl_2_ instead of ZnI_2_	30	75:25
9	AlMe_3_ instead of ZnI_2_	44	75:25
10	MgBr_2_·OEt_2_ instead of ZnI_2_	45	56:44
11	**2a** instead of **2d**	37^e^	7:93
12	**2b** instead of **2d**	Trace	ND

^a^Reaction conditions: **1** (0.2–0.5 mmol), **2** (1.2 equiv), ABVN (0.5 equiv), Lewis acid (0–1 equiv), toluene (2–3 mL), 60 °C for 12–24 h. The yield is recorded for the combination of diastereomers **3** and **4**. dr refers to the ratio of **3**:**4**. ND, not detected.

^b^NMR yield using tetrachloroethane as an internal standard.

^c^dr was determined by ^1^H NMR analysis of the crude reaction mixture.

^d^Isolated yield.

^e^**3aa-Me** and **4aa-Me** were obtained.

**Fig. 3:**
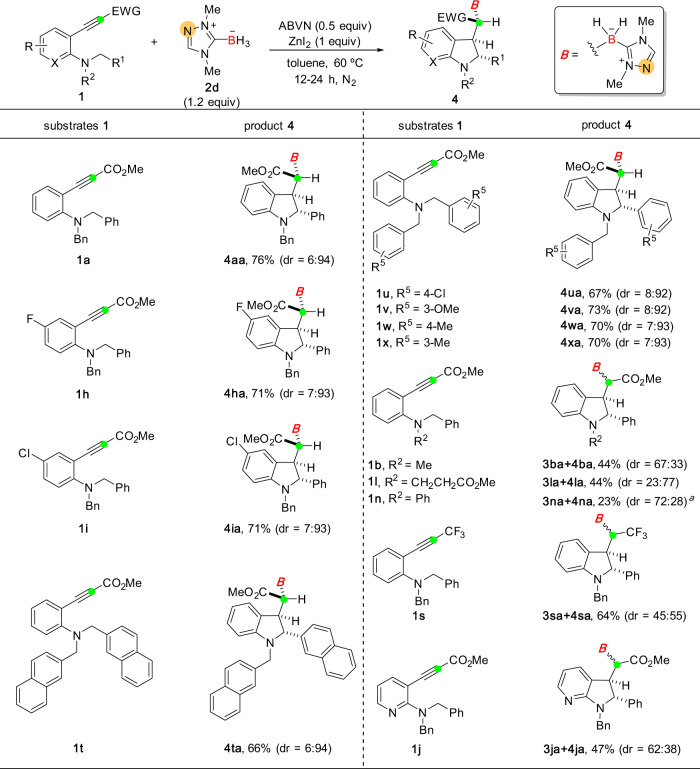
NHC-BH_3_/ZnI_2_-controlled stereoselective synthesis of borylated molecules derived from RATC. Reactions were performed with substrate **1** (0.2–0.5 mmol), **2d** (1.2 equiv), ABVN (0.5 equiv), and ZnI_2_ (1 equiv) in toluene (2–3 mL) at 60 °C for 12–24 h. The yield is recorded for the combination of diastereomers **3** and **4**. dr refers to the ratio of **3**:**4**. ^a^**1n** was recovered in 50% yield.

This NHC-BH_3_/ZnI_2_ chelation-controlled diastereoselective synthesis showed broad substrate scope and functional group tolerance (Fig. 3). In most reactions, both moderate to good yields and high levels of dr (8:92 ~ 6:94) could be obtained (**4aa**, **4ha**, **4ia**, **4ta–4xa**). However, when the R^2^ substituent was changed to other alkyl groups (for **1b** and **1l**) or phenyl group (**1n**), the products were formed with low stereochemical outcomes. This implied that the benzyl group as R^2^ is crucial. In addition, replacing the terminal methoxycarbonyl group with a non-coordinating CF_3_ moiety resulted in a precipitous drop in diastereoselectivity (**3sa**:**4sa** = 45:55), hinting the participation of the carbonyl moiety in the diastereo-determining step. Moreover, the presence of a strong coordinative pyridine ring (for **1j**) also interrupted the chelation, resulting in almost no diastereoselectivity.

### Synthetic applications

**Fig. 4:**
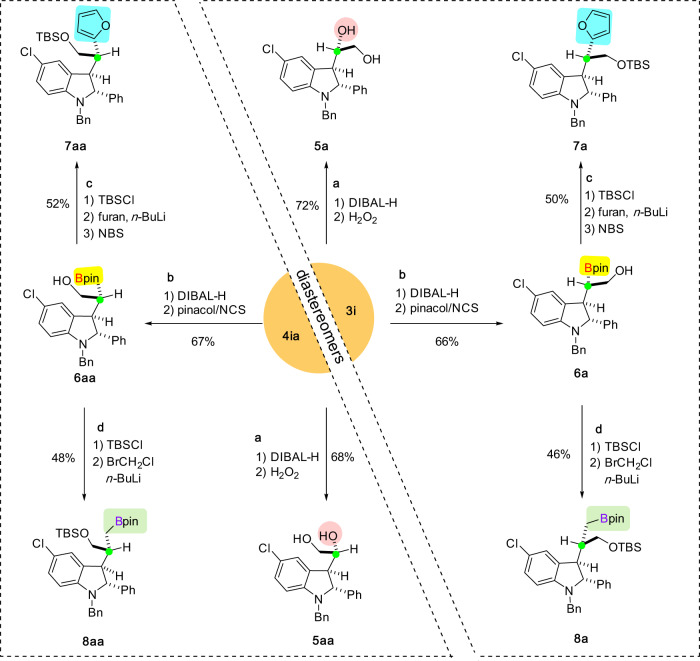
Stereospecific transformations for the synthesis of diastereomeric functional molecules. **a** Synthesis of hydroxylated products **5a** and **5aa**. **b** Synthesis of pinacol boronic esters **6a** and **6aa**. **c** Synthesis of furan-substituted products **7a** and **7aa**. **d** Synthesis of homologated pinacol boronic esters **8a** and **8aa**.

The synthetic utility of this diastereodivergent RATC process was demonstrated (Fig. 4). Diastereomeric α-boryl carbonyls **3i** and **4ia** were converted to a series of useful building blocks based on the versatile chemistry of both the boryl and carbonyl moieties. For example, reduction of the ester group of **3i** and **4ia** followed by oxidation afforded diastereomeric 1,2-diols **5a** and **5aa** in good yields. Instead of oxidation, the treatment with *N*-chlorosuccinimide (NCS) in the presence of pinacol and further protection of the hydroxy group provided useful pinacol boronic esters **6a** and **6aa**, respectively. The subsequent coupling reaction with furan using Aggarwal’s arylation protocol^49^ delivered the corresponding **7a** and **7aa** in good efficiency. Moreover, homologation of **6a** and **6aa** also proceeded, furnishing alkyl boronic esters **8a** and **8aa** in good yields. It is worth mentioning that the starting materials **3i** and **4ia** were both used as a single diastereomer and the stereochemistry was retained in all these transformations, thus the resulting products were obtained with exclusive diastereoselectivity.

## Discussion

### Computational studies of diastereoselective HAT controlled by NHC-BH_3_/thiol catalyst

**Fig. 5:**
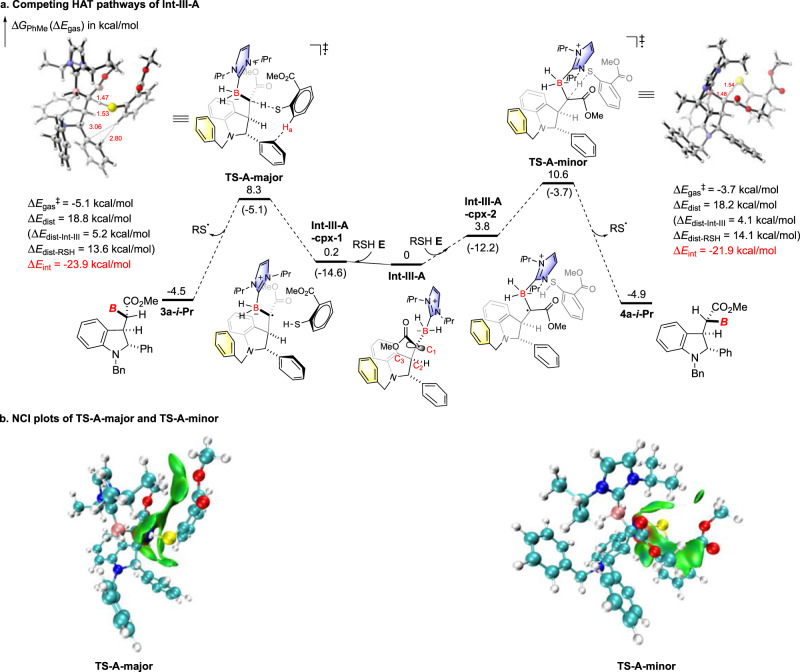
Analysis of the diastereoselectivity in the NHC-BH_3_ 2b/thiol E-controlled HAT process. **a** Competing HAT pathways of **Int-III-A**. **b** NCI plots of **TS-A-major** and **TS-A-minor**.

To elucidate the diastereoselectivity of this HAT process, density functional theory (DFT) calculations were carried out at the (U)B3LYP(D3)/6-311+G(d,p)/SMD(toluene)//(U)B3LYP(D3)/6-31G(d,p) level of theory (Fig. 5). Firstly, the rotational barrier of C_1_-C_2_ bond in **Int-III-A** was roughly estimated by single-point energy calculations of the optimized structures with the frozen dihedral angles of B-C_1_-C_2_-C_3_ (Supplementary Fig. 4). The results reveal that ~7 kcal/mol of energy barrier is required for the free rotation of C_1_–C_2_ bond in **Int-III-A**. This energy is lower than the activation Gibbs energies of HATs, which are 8.3 and 10.6 kcal/mol, respectively. Therefore, the stereochemistry is determined by the relative HAT transition states, based on the Curtin–Hammett principle^50^. Then hydrogen atom transfer to **Int-III-A** was computed by using thiol **E** for simplification. The transition states leading to products **3a-*i*-Pr** and **4a-*i*-Pr** were located as **TS-A-major** and **TS-A-minor**, respectively (Fig. 5). Before reaching these two transition states, two complexes between **Int-III-A** and thiol **E** are formed. **TS-A-major** is 2.3 kcal/mol more favorable than **TS-A-minor** in terms of Gibbs free energy, suggesting that **3a-*i*-Pr** is the major product. This is consistent with the experimental observation (Table 1, entry 7). To find the origin of the stereochemistry preference for **TS-A-major** over **TS-A-minor**, distortion/interaction analysis^51–53^ (DIA) was performed by separating the transition states into two fragments, **Int-III-A** and thiol **E**. The distortion energy for **TS-A-major** is slightly higher than that of **TS-A-minor** (18.8 vs. 18.2 kcal/mol), and a stronger interaction energy is observed in **TS-A-major** (ΔΔ*E*_int_ = −2.0 kcal/mol). These imply the presence of different interaction energies that determines the diastereoselectivity. The interactions between **Int-III-A** and thiol **E** in **TS-A-major** and **TS-A-minor** were further analyzed by noncovalent interaction^54,55^ (NCI) and AIM analysis. The results indicate that the lower interaction energy in **TS-A-major** is likely attributed to the CH/π attraction^56,57^ between H_a_ on thiol **E** and the 2-phenyl moiety, which is absent in the competing **TS-A-minor**. This is further supported by the experimental finding that thiol **G** lacking such *ortho*-C*sp*^2^-H gave inferior diastereoselectivity (Table 1, entry 9).

### Experimental and Computational Studies of Diastereoselective HAT Controlled by NHC-BH_3_/Lewis Acid

**Fig. 6:**
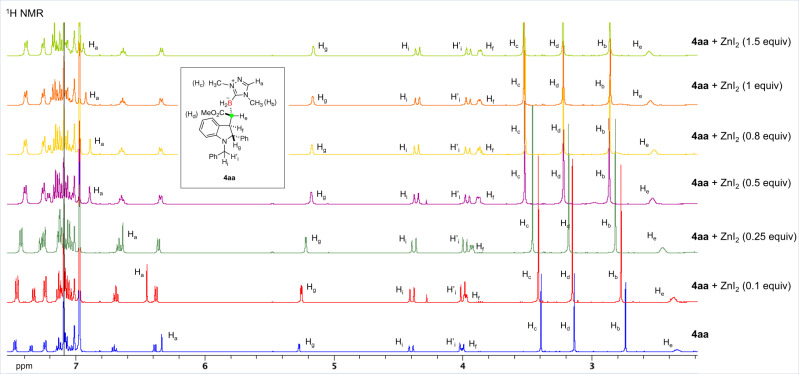
Stacked ^1^H NMR spectra of 4aa with an increasing amount of ZnI_2_. NMR spectra were recorded on a Bruker Avance 500 spectrometers (500 MHz) in toluene-*d*_8_.

Experimental and computational studies were then conducted to study how ZnI_2_-chelation-controlled HAT process takes place. Because **Int-III-B** is assumed to have a chelation with ZnI_2_ similar to that of product **4aa**, we used ^1^H NMR spectroscopy measurements to probe the possible chelation sites employing **4aa** as a model (Fig. 6). Upon addition of ZnI_2_ (0–1.5 equiv), the protons locating on the triazole moiety (H_a_, H_b_ and H_c_) are shifted downfield. In addition, the proton of methoxycarbonyl group (H_d_) and α-carbonyl proton (H_e_) also exhibit a downfield shift. These imply that both the triazole and carbonyl groups are involved in binding with Zn^II^. It should be noted that protons at α-positions of the nitrogen atom (H_g_, H_i_, and H’_i_) have negligible changes, which indicates that the coordination of this aryl-substituted nitrogen atom to the zinc center is weak. Importantly, only a single set of ^1^H NMR spectrum is observed in all cases, suggesting a fast dynamics of complexation/decomplexation between the free **4aa** and Zn^II^-**4aa** chelates. A similar behavior of dynamics between free **2d** and Zn^II^-**2d** was also observed (Supplementary Fig. 3). As such, the following DFT calculations employed **Int-III** bound to one molecular of ZnI_2_ and NHC-BH_3_ **2d** ligated with another one ZnI_2_ as the model substrate and hydrogen atom donor, respectively.

**Fig. 7:**
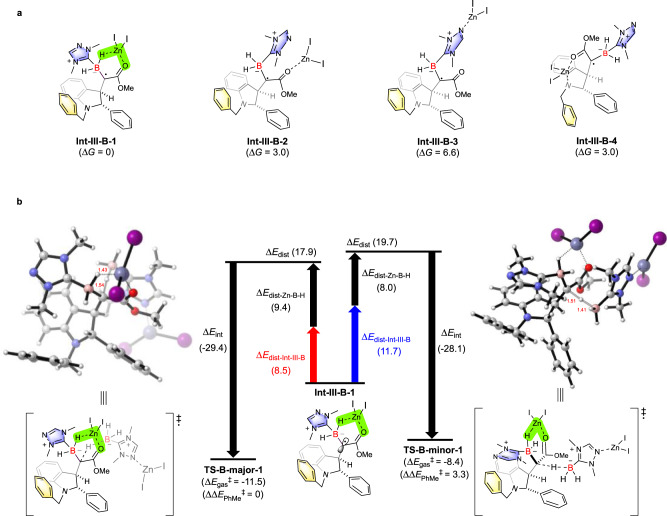
Computational studies of ZnI_2_-mediated reactions. **a Int-III-B** with different chelation modes. **b** Analysis of the diastereoselectivity in the NHC-BH_3_/ZnI_2_-controlled HAT processes.

To investigate the chelation mode of ZnI_2_ with **Int-III**, a variety of possible complexes with different chelation modes were extensively screened. The results revealed that the most favored chelation mode contains a ZnI_2_-chelated six-membered ring, where ZnI_2_ coordinates with both the carbonyl oxygen atom and the B–H bond^58,59^ (**Int-III-B-1** in Fig. 7a). ^1^H NMR spectroscopy of **2a** titrated with ZnI_2_ showed a downfield shift of B–H signals (Supplementary Fig. 2), providing an evidence for the interaction of ZnI_2_ with the B–H bond. Some other chelation models were also considered and computed, such as the coordination of ZnI_2_ with only methoxycarbonyl oxygen atom (**Int-III-B-2**), or with the triazole nitrogen atom (**Int-III-B-3**), or with both the carbonyl group and the indoline nitrogen atom (**Int-III-B-4**). However, all of them are energetically disfavored in comparison with **Int-III-B-1**. The HAT transition states of the aforementioned four possible intermediates were located, and a preference of **Int-III-B-1** was observed (Supplementary Fig. 5). Moreover, our experimental findings showed no evident difference of dr value between **2a** (7:93, Table 2, entry 9) and **2d** (6:94, Table 2, entry 1), hinting that the chelation between ZnI_2_ and the triazole moiety is most likely not the leading factor of determining HAT diastereoselectivity. However, it is worth mentioning that the use of **2d** (Table 2, entry 1) gives a much better product yield than that of **2a** (Table 2, entry 11), meaning that the dynamic complexation between **2d** and ZnI_2_ was probably helpful to accelerate the cascade process, while the detailed mechanism is still unclear and further mechanistic studies are currently under progress in our laboratory. In addition, the optimization studies (Table 2) showed that other Lewis acids, such as MgCl_2_, MgI_2_, CuI, ZnCl_2_, Me_3_Al, and MgBr_2_·OEt_2_, are ineffective in controlling the stereoselectivity, suggesting that both the zinc center and the iodide anions are crucial to form the favored HAT transition state that can lead to high levels of diatereoselectivity. The switch of the anion to chloride might result in a more flexible complexation and the six-membered ring chelation mode was likely not formed, and then inducing no effect of stereocontrol (Table 2, entry 8).

As depicted in Fig. 7b, calculations suggest HAT from **2d**-ZnI_2_ complex to **Int-III-B-1** prefers to occur via **TS-B-major-1** over **TS-B-minor-1** (ΔΔ*G*^‡^ = 3.3 kcal/mol), which correlates well with the selectivity trend observed in the experiment. There are complexes formed between **Int-III-B-1** and ZnI_2_-ligated **2d**, we did not locate them because analyzing the transition states of **TS-B-major-1** and **TS-B-minor-1** can give us answers to stereochemistry. These two transition states **TS-B-major-1** and **TS-B-minor-1** were then subjected to DIA. Based on the DIA results, the preference of **TS-B-major-1** could be mainly ascribed to the lower distortion energy of **Int-III-B-1** (8.5 kcal/mol) than that in **TS-B-minor-1** (11.7 kcal/mol). Careful inspection of the transition state structures reveals the specific Zn-chelated six-membered ring plays a critical role in controlling the diastereoselectivity. In **TS-B-minor-1**, the six-membered ring has to undergo substantial torsion to orient towards *N*-Bn group, thus the steric congestion between these two components is likely the cause of high distortion energy in **Int-III-B-1**. This hypothesis is in line with the poor diastereoselectivities observed in the reactions of **1b**, **1l**, **1n**, where *N*-alkyl and *N*-phenyl groups are employed instead of *N*-benzyl group (Fig. 3). Subsequent DFT calculation and DIA on HAT step of **1b** provided computational support for this hypothesis, finding that replacing *N*-Bn with *N*-Me results in a dramatic decrease of the distortion energy (Supplementary Fig. 6). Computational studies of the HAT from free NHC-BH_3_ **2d** to **Int-III-B** were also conducted, and the results show that the use of free **2d** or **2d**-ZnI_2_ complex as the hydrogen atom donor has a negligible effect on the diastereoselectivity (Supplementary Fig. 7).

In summary, we have developed an NHC-boryl radical triggered radical cascade to assemble heterocyclic molecules anchoring a boron-substituted acyclic stereocenter. The stereochemistry of HAT to acyclic radical intermediates is determined by NHC-BH_3_/thiol catalyst and NHC-BH_3_/ZnI_2_-chelation systems. Experimental and computational studies suggest that these two modes operate in complementary ways. A cooperative control by the NHC unit and a thiol catalyst favor the formation of one diastereomer, while a B–H bond-involved six-membered ring chelation with ZnI_2_ is beneficial for the formation of the other one. Such a stereoselectivity switch enables the divergent synthesis of diastereomeric borylated products. We expect that the present stereoselectivity-switchable HAT strategy and the resulting diastereomeric borylated products would be important for synthetic and medicinal applications.

## Methods

### General procedure for NHC-BH_3_/thiol catalyst-controlled stereoselective synthesis of borylated molecules

To a 25 mL flame-dried Schlenk flask under nitrogen, substrate **1** (0.400 mmol), **2b** (0.480 mmol), ABVN (0. 080 mmol), and phenyl 2-mercaptobenzoate (0.080 mmol) in toluene (4 mL) was stirred at 60 °C for 3–12 h under nitrogen atmosphere. After evaporation of the solvent, the resulting crude residue was purified by flash column chromatography (silica gel; petroleum ether/ethyl acetate) to give the corresponding products.

### General procedure for NHC-BH_3_/ZnI_2_-controlled stereoselective synthesis of borylated molecules

To a 25 mL flame-dried Schlenk flask under nitrogen, substrate **1** (0.400 mmol), **2d** (0.480 mmol), ABVN (0. 200 mmol), and ZnI_2_ (0.400 mmol) in toluene (2 mL) was stirred at 60 °C for 12–24 h under nitrogen atmosphere. The reaction mixture was quenched with NH_4_Cl-NH_3_/H_2_O buffer solutions. The aqueous layer was extracted three times with dichloromethane. The combined extracts were dried over Na_2_SO_4_ and concentrated in *vacuo*. The resulting crude residue was purified by flash column chromatography (silica gel; petroleum ether/ethyl acetate) to give the corresponding products.

### Computational methods

Calculations were carried out with Gaussian 16 software packages. The (U)B3LYP functional was used with an empirical dispersion correction (Grimme-D3(0)) for all the calculations. The geometries of all the stationary points were optimized in the gas phase with the SDD basis set (Stuttgart/Dresden ECP) for Zn and I, and the 6-31G(d,p) basis set for the other atoms. In the computational studies of HAT controlled by NHC-BH_3_/thiol catalyst, the internal six d-type orbitals were used for all elements in geometry optimization part. In the part of diastereoselective HAT controlled by NHC-BH_3_/Lewis acid, the keyword “5D” was used to specify that five d-type orbitals were used for all elements in the calculations. Vibrational frequency analysis was calculated at the same level of theory to validate each structure as either a minimum or a transition state. For each transition state, the intrinsic reaction coordinate (IRC) analysis was conducted to ensure that it connects the right reactant and product. To obtain more accurate energies, single-point energies were calculated with a mixed basis set of SDD for Zn and I, 6-311+G(d,p) for all the other atoms. The distortion/interaction analysis was performed with gas-phase single point energies, while all the other energies reported in the main manuscript were obtained from solution-phase single point energies with SMD solvation model (solvent = toluene). Noncovalent interaction (NCI) analysis was performed with Multiwfn by using the IGM method. The results were visualized by VMD software. 3D structures were generated by CYLview. See Supplementary refs. 22–37.

## Supplementary information

Supplementary Information

Description of Additional Supplementary Files

Supplementary Data 1

## Data Availability

The X-ray crystallographic coordinates for **3a-Me** (CCDC 1994057), **3a-*****i*****-Pr** (CCDC 1994058), **4a-Me** (CCDC 1994059), **3s** (CCDC 1994060), and **4aa** (CCDC 1994061) have been deposited at the Cambridge Crystallographic Data Centre (CCDC). These data can be obtained free of charge from the CCDC via www.ccdc.cam.ac.uk/data_request/cif. The experimental procedures, kinetic studies, computational results, and characterization of all new compounds are provided in the Supplementary Information.
