# Supplementary material for: Stereoselective hydrogen atom transfer to acyclic radicals: a switch enabling diastereodivergent borylative radical cascades
Source: Nat Commun. 2022 Jan 20;13:426. doi: 10.1038/s41467-022-28071-8 (PMC8776760; doi:10.1038/s41467-022-28071-8)
Supplement: Supplementary file 1 — Supplementary Information [file 41467_2022_28071_MOESM1_ESM.pdf]

1. Supplementary Methods

General Information

All reactions were performed by standard Schlenk techniques in oven-dried reaction vessels under nitrogen atmosphere. ^{1}H, ^{11}B, ^{13}C and ^{19}F NMR spectra were recorded on a Bruker Avance 400 or 500 spectrometers (400 MHz or 500 MHz) in $CDCl_3$, DMSO-d_6, or toluene-d_8. The following abbreviations were used to explain the multiplicities: s = singlet, d = doublet, t = triplet, q = quartet, dd = doublet of doublet, ddd = doublet of doublet of doublet, dt = doublet of triplet, m = multiplet, s br = single broad. High-resolution mass spectra were obtained with a Water XEVO G2 Q-Tof (Waters Corporation). X-ray crystallography analysis was performed on Bruker X8 APEX X-ray diffractionmeter. Melting points were uncorrected and were recorded on a Buchi B-54 melting point apparatus. Flash column chromatography was performed using Merck silica gel 60 with distilled solvents. Commercially available reagents were purchased from Energy Chemical, J & K Scientific, Adamas-beta and Sigma-Aldrich Co., Inc.Phenyl 2-mercaptobenzoate,[1] **3o**,[2] NHC−boranes **2a**,[3] **2b**,[4] **2c**[5] and **2d**[4] were known compounds and prepared according to the literature procedures.

Note: In the ^{1}H NMR spectral data, the protons on boron are not listed due to quadrupole broadening and spin–spin coupling with boron.

2. Supplementary Discussions

2.1 Synthesis of starting materials

General procedure A:

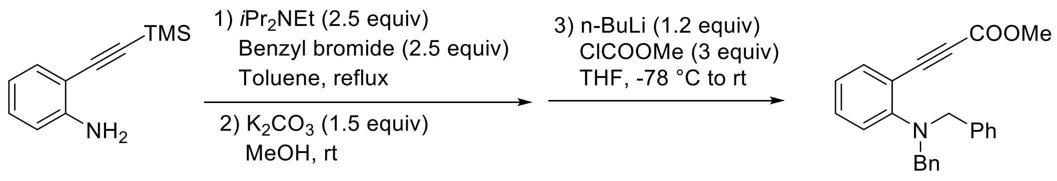

1a

To a solution of 2-((trimethylsilyl)ethynyl)aniline[6] (3.800 g, 20.00 mmol) in toluene was added *i*Pr_2NEt (8.3 mL, 50.22 mmol) and benzyl bromide (5.9 mL, 49.68 mmol). The reaction mixture was refluxed under nitrogen atmosphere for 72 h. The white solid precipitant was filtered off and the solvent was removed under *vacuo* to afford orange crude product. The crude material was dissolved in MeOH and was added K_2CO_3 (4.146 g, 30.00 mmol). The reaction mixture was stirred at room temperature for 1 h and quenched with water. The aqueous layer was extracted with CH_2Cl_2 three times. The combined organic extracts were dried over Na_2SO_4, and concentrated in *vacuo*. The crude residue was dissolved in dry THF and cooled to -78 °C, *n*-BuLi (15 mL, 1.6 M in hexanes) was added. Methyl carbonochloridate (4.7 ml, 60.00 mmol) was added after the reaction mixture was stirred at -78 °C for 30 min, then warmed to room temperature and stirred at room temperature for 30 min. Quenched with water and the aqueous layer was extracted with CH_2Cl_2 three times. The combined organic extracts was dried over Na_2SO_4, and concentrated in *vacuo*. The crude residue was purified by flash column chromatography on silica gel (petroleum ether : ethyl acetate = 50:1) to give **1a** (4.618 g) in 65% yield as orange oil. ^{1}H NMR (400 MHz, $CDCl_3$) δ 3.77 (s, 3H), 4.44 (s, 4H), 6.84-6.90 (m, 2H), 7.19-7.31 (m, 11H), 7.54-7.56 (dd, *J* = 7.6, 1.2 Hz, 1H); ^{13}C NMR (100 MHz, $CDCl_3$) δ 52.5, 56.3, 85.6, 86.3, 112.5, 120.5, 121.1, 127.0, 128.1, 128.2, 131.3, 135.8, 137.9, 154.4, 154.7; ESI-HRMS (*m/z*): $(M+H)^+$ calcd for $C_{24}H_{22}NO_2$, 356.1651; found: 356.1645.

Methyl 3-(2-(dibenzylamino)-5-fluorophenyl)propiolate (1h)

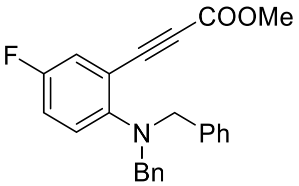

Following general procedure A, **1h** was obtained in 63% yield (1.191 g, 3.19 mmol) from the reaction of 4-fluoro-2-((trimethylsilyl)ethynyl)aniline[7] (1.050 g, 5.06 mmol) as a yellow solid, mp: 80-82 °C; ^{1}H NMR (400 MHz, $CDCl_3$) δ 3.80 (s, 3H), 4.31 (s, 4H), 6.81 (dd, *J* = 8.8, 4.8 Hz, 1H), 6.93 (ddd, *J* = 8.8, 8.8, 2.8 Hz, 1H), 7.20-7.31 (m, 11H); ^{13}C NMR (100 MHz, $CDCl_3$) δ 52.7, 57.0, 84.4 (d, *J* = 2.9 Hz), 85.8, 115.3 (d, *J* = 9.1 Hz), 118.4 (d, *J* = 22.0 Hz), 121.2 (d, *J* = 23.9 Hz), 122.8 (d, *J* = 8.2 Hz), 127.1, 128.3 (overlapped), 137.7, 150.9, 154.4, 157.1 (d, *J* = 241.3 Hz); ^{19}F NMR (376 MHz, $CDCl_3$): δ -121.0 (1F, m); ESI-HRMS (*m/z*): $(M+H)^+$ Calcd for $C_{24}H_{21}FNO_2$, 374.1556; Found: 374.1553.

Methyl 3-(5-chloro-2-(dibenzylamino)phenyl)propiolate (1i)

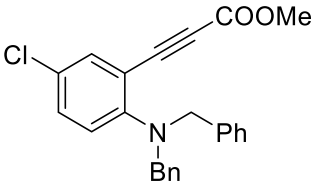

Following general procedure A, **1i** was obtained in 53% yield (1.031 g, 2.65 mmol) from the reaction of 4-chloro-2-((trimethylsilyl)ethynyl)aniline[8] (1.120 g, 5.00 mmol) as a yellow solid, mp: 70-72 °C; ^{1}H NMR (400 MHz, $CDCl_3$) δ 3.76 (s, 3H), 4.41 (s, 4H), 6.76 (d, *J* = 8.8 Hz, 1H), 7.14 (dd, *J* = 8.8, 2.4 Hz, 1H), 7.20-7.28 (m, 10H), 7.50 (d, *J* = 2.4 Hz, 1H); ^{13}C NMR (100 MHz, $CDCl_3$) δ 52.6, 56.5, 84.5, 86.2, 114.0, 121.8, 125.9, 127.2, 128.0, 128.4, 131.3, 134.8, 137.5, 152.8, 154.3; ESI-HRMS (*m/z*): $(M+H)^+$ Calcd for $C_{24}H_{21}ClNO_2$, 390.1261; Found: 390.1262.

Methyl 3-(2-(dibenzylamino)pyridin-3-yl)propiolate (1j)

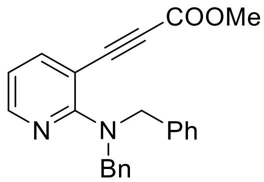

Following general procedure A, **1j** was obtained in 61% yield (1.183 g, 3.32 mmol) from the reaction of 3-((trimethylsilyl)ethynyl)pyridin-2-amine[9] (1.040 g, 5.45 mmol) as a yellow solid, mp: 62-63 °C; ^{1}H NMR (400 MHz, $CDCl_3$) δ 3.65 (s, 3H), 4.93 (s, 4H), 6.68 (dd, *J* = 7.6, 4.8 Hz, 1H), 7.22-7.32 (m, 10H), 7.72 (dd, *J* = 7.6, 1.6 Hz, 1H), 8.24 (dd, *J* = 4.8, 1.6 Hz, 1H); ^{13}C NMR (100 MHz, $CDCl_3$) δ 52.4, 52.5, 85.5, 86.1, 99.8, 113.6, 126.9, 127.5, 128.3, 138.1, 145.5, 150.1, 154.2, 160.4; ESI-HRMS (*m/z*): $(M+H)^+$ Calcd for $C_{23}H_{21}N_2O_2$, 357.1603; Found: 357.1599.

Methyl 3-(2-(benzyl(but-3-en-1-yl)amino)phenyl)propiolate (1k)

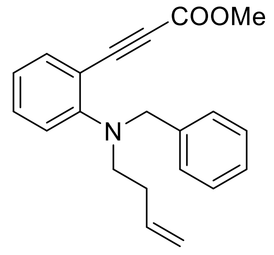

Following general procedure C, **1k** was obtained in 47% yield (0.982.g, 3.07 mmol) from the reaction of *N*-(but-3-en-1-yl)-2-((trimethylsilyl)ethynyl)aniline[10] (1.590 g, 6.53 mmol) as yellow oil; ^{1}H NMR (400 MHz, $CDCl_3$) δ 2.32-2.39 (m, 2H), 3.33-3.37 (m, 2H), 3.79 (s, 3H), 4.50 (s, 2H), 4.95-5.04 (m, 2H), 5.71-5.81 (m, 1H), 6.85 (ddd, *J* = 7.6, 7.2, 0.8 Hz, 1H), 6.90 (dd, *J* = 8.4, 0.4 Hz, 1H), 7.20-7.33 (m, 6H), 7.52 (dd, *J* = 7.6, 1.6 Hz, 1H); ^{13}C NMR (100 MHz, $CDCl_3$) δ 31.9, 51.8, 52.5, 57.0, 85.3, 86.8, 111.7, 116.1, 119.7, 120.5, 126.9, 127.8, 128.3, 131.4, 135.7, 136.1, 138.2, 154.2, 154.7; ESI-HRMS (*m/z*): $(M+H)^+$ Calcd for $C_{21}H_{22}NO_2$, 320.1651; Found: 320.1652.

Methyl 3-(2-(naphthalen-2-yl(naphthalen-2-ylmethyl)amino)phenyl)propiolate (1t)

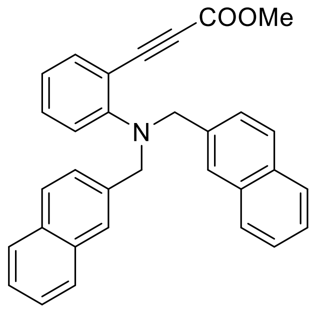

Following general procedure A, **1t** was obtained in 59 % yield (2.480 g, 5.44 mmol) from the reaction of 2-((trimethylsilyl)ethynyl)aniline[6] (1.745 g, 9.22 mmol) and 2-(bromomethyl)naphthalene (5.100 g, 23.07 mmol) as yellow oil; ^{1}H NMR (400 MHz, $CDCl_3$) ^{1}H NMR (400 MHz, $CDCl_3$) δ 3.68 (s, 3H), 4.65 (s, 4H), 6.92 (ddd, *J* = 7.6, 7.6, 1.2 Hz, 1H), 6.97 (d, *J* = 8.0 Hz, 1H), 7.21-7.26 (m, 1H), 7.43-7.49 (m, 6H), 7.60 (dd, *J* = 7.2, 1.6 Hz, 1H), 7.76-7.83 (m, 8H); ^{13}C NMR (100 MHz, $CDCl_3$) δ 52.5, 56.6, 85.7, 86.4, 112.7, 120.7, 121.4, 125.6, 125.9, 126.3, 126.9, 127.6, 127.8, 128.0, 131.4, 132.7, 133.3, 135.5, 135.9, 154.6, 154.7; ESI-HRMS (*m/z*): $(M+H)^+$ Calcd for $C_{32}H_{26}NO_2$, 456.1964; Found: 456.1964.

Methyl 3-(2-(bis(4-chlorobenzyl)amino)phenyl)propiolate (1u)

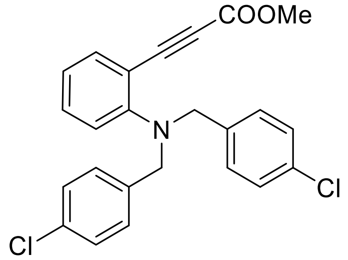

Following general procedure A, **1u** was obtained in 61% yield (2.069 g, 4.88 mmol) from the reaction of 2-((trimethylsilyl)ethynyl)aniline[6] (1.510 g, 8.00 mmol) and 1-(bromomethyl)-4-chlorobenzene (4.109 g, 20.00 mmol) as a yellow solid, mp: 112-114 °C; ^{1}H NMR (400 MHz, $CDCl_3$) δ 3.79 (s, 3H), 4.33 (s, 4H), 6.83 (d, *J* = 8.0 Hz, 1H), 6.93 (ddd, *J* = 7.6, 7.6, 1.2 Hz, 1H), 7.21-7.26 (m, 9H), 7.55 (dd, *J* = 7.6, 1.2 Hz, 1H); ^{13}C NMR (100 MHz, $CDCl_3$) δ 52.6, 55.9, 85.7, 85.8, 113.4, 120.8, 122.0, 128.5, 129.5, 131.4, 132.8, 135.8, 136.2, 153.9, 154.5; ESI-HRMS (*m/z*): $(M+H)^+$ Calcd for $C_{24}H_{20}Cl_2NO_2$, 424.0871; Found: 424.0869.

Methyl 3-(2-(bis(3-methoxybenzyl)amino)phenyl)propiolate (1v)

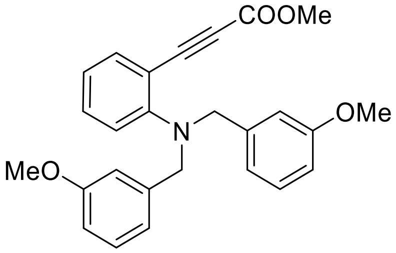

Following general procedure A, **1v** was obtained in 42% yield (1.082 g, 2.60 mmol) from the reaction of 2-((trimethylsilyl)ethynyl)aniline[6] (1.174 g, 6.20 mmol) and 1-(bromomethyl)-3-methoxybenzene (3.116 g, 15.5 mmol) as yellow oil; ^{1}H NMR (400 MHz, $CDCl_3$) δ 3.75 (s, 6H), 3.77 (s, 3H), 4.41 (s, 4H), 6.75 (dd, J = 2.4, 0.8 Hz, 1H), 6.77 (dd, J = 2.8, 0.8 Hz, 1H), 6.84-6.91 (m, 6H), 7.16-7.25 (m, 3H), 7.55 (dd, J = 8.0, 2.4 Hz, 1H); ^{13}C NMR (100 MHz, $CDCl_3$) δ 52.5, 55.0, 56.3, 85.6, 86.3, 112.7, 112.9, 113.2, 120.4, 120.6, 121.3, 129.2, 131.4, 135.8, 139.6, 154.5, 154.7, 159.7; ESI-HRMS (*m/z*): $(M+Na)^+$ Calcd for $C_{26}H_{25}NNaO_4$, 438.1681; Found: 438.1681.

Methyl 3-(2-(bis(4-methylbenzyl)amino)phenyl)propiolate (1w)

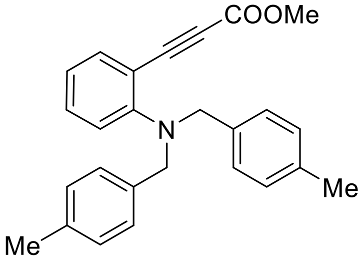

Following general procedure A, **1w** was obtained in 71% yield (2.440 g, 6.35 mmol) from the reaction of 2-((trimethylsilyl)ethynyl)aniline[6] (1.700 g, 8.98 mmol) and 1-(bromomethyl)-4-methylbenzene (4.155 g, 22.45 mmol) as a yellow solid, mp: 58-59 °C; ^{1}H NMR (400 MHz, $CDCl_3$) δ 2.32 (s, 6H), 3.79 (s, 3H), 4.39 (s, 4H), 6.84-6.90 (m, 2H), 7.09 (d, J = 8.4 Hz, 4H), 7.17 (d, J = 8.4 Hz, 4H), 7.22 (ddd, J = 8.4, 7.6, 1.6 Hz, 1H), 7.55 (dd, J = 7.6, 1.6 Hz 1H); ^{13}C NMR (100 MHz, $CDCl_3$) δ 21.0, 52.5, 55.9, 85.6, 86.6, 112.5, 120.6, 121.0, 128.1, 128.9, 131.3, 134.8, 135.8, 136.5, 154.6, 154.7; ESI-HRMS (*m/z*): $(M+H)^+$ Calcd for $C_{26}H_{26}NO_2$, 384.1964; Found: 384.1959.

Methyl 3-(2-(bis(3-methylbenzyl)amino)phenyl)propiolate (1x)

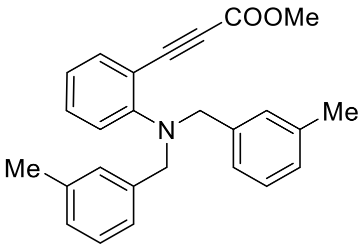

Following general procedure A, **1x** was obtained in 61% yield (1.032 g, 3.04 mmol) from the reaction of 2-((trimethylsilyl)ethynyl)aniline[6] (1.300 g, 6.60 mmol) and 1-(bromomethyl)-3-methylbenzene (3.000 g, 16.21 mmol) as yellow oil; ^{1}H NMR (400 MHz, $CDCl_3$) δ 2.30 (s, 6H), 3.76 (s, 3H), 4.39 (s, 4H), 6.85-6.89 (m, 2H), 7.01-7.24 (m, 9H), 7.54 (dd, *J* = 1.6, 0.4 Hz, 1H); ^{13}C NMR (100 MHz, $CDCl_3$) δ 21.4, 52.5, 56.3, 85.5, 86.5, 112.3, 120.4, 121.0, 125.1, 127.7, 128.1, 128.8, 131.3, 135.8, 137.8, 137.9, 154.6, 154.7; ESI-HRMS (*m/z*): $(M+H)^+$ Calcd for $C_{26}H_{26}NO_2$, 384.1964; Found: 384.1960.

General procedure B:

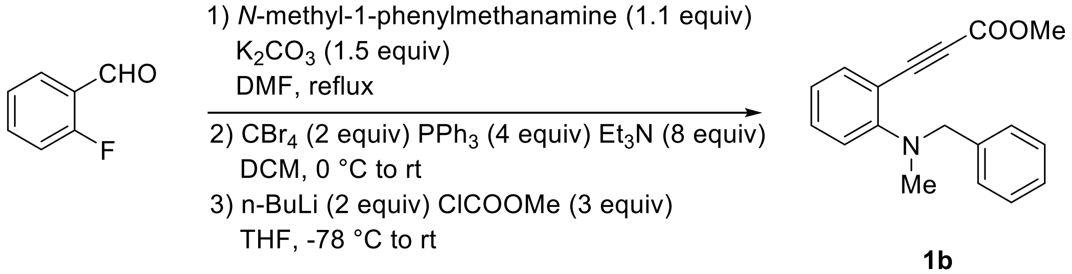

1b

To a solution of 2-fluorobenzaldehyde (2.500 g, 20.14 mmol) in DMF (100 mL) was added *N*-methyl-1-phenylmethanamine (2.670 g, 22.00 mmol) and K_2CO_3 (4.146 g, 30.00 mmol) under nitrogen atmosphere. The reaction mixture was refluxed for 48 h and was then quenched with water. The aqueous layer was extracted with CH_2Cl_2 three times. The combined organic extracts were dried over Na_2SO_4, and concentrated in *vacuo* to afford crude product. To a solution of CBr_4 (13.27 g, 40.00 mmol) in CH_2Cl_2 (20 mL) was added dropwise a solution of PPh_3 (21.00 g, 80.00 mmol) in CH_2Cl_2 (20 mL) at 0 °C. A solution of the crude mixture in CH_2Cl_2 (10 mL) and Et_3N

(22.4 mL, 160.00 mmol) was added dropwise after the reaction mixture was stirred at 0 °C for 30 min, then warmed to room temperature and stirred for 1 h. Quenched with water and the aqueous layer was extracted with CH_2Cl_2 three times. The combined organic extracts were dried over Na_2SO_4, and concentrated in *vacuo* to afford the brown crude product. The crude residue was purified by a short flash column chromatography on silica gel eluted with petroleum ether to get rid of the side-product triphenylphosphine oxide which had large polarity. The collected product was dissolved in dry THF and cooled to -78 °C, n-BuLi (25 mL, 1.6 M in hexanes) was added dropwise. Methyl carbonochloridate (4.7 mL, 60.00 mmol) was added after the reaction mixture was stirred at -78 °C for 30 min, then warmed to room temperature and stirred at room temperature for 30 min. Quenched with water and the aqueous layer was extracted with CH_2Cl_2 three times. The combined organic extracts were dried over Na_2SO_4, and concentrated in *vacuo*. The crude residue was purified by flash column chromatography on silica gel (petroleum ether : ethyl acetate = 50:1) to give **1b** (2.312 g, 8.26 mmol) in 41% yield as yellow oil. ^1H NMR (400 MHz, $CDCl_3$) δ 2.84 (s, 3H), 3.74 (s, 3H), 4.52 (s, 2H), 6.85-6.91 (m, 2H), 7.24-7.35 (m, 6H), 7.53 (dd, J = 7.6, 1.6 Hz, 1H); ^{13}C NMR (100 MHz, $CDCl_3$) δ 39.6, 52.5, 60.2, 85.7, 86.7, 110.3, 118.0, 120.2, 127.1, 128.0, 128.3, 131.7, 136.0, 138.0, 154.7, 155.7; ESI-HRMS (*m/z*): $(M+H)^+$ Calcd for $C_{18}H_{18}NO_2$, 280.1338; Found: 280.1340.

Methyl 3-(2-((4-chlorobenzyl)(methyl)amino)phenyl)propiolate (1c)

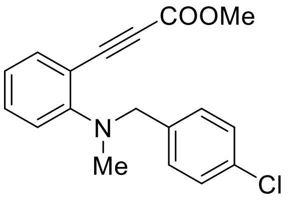

Following general procedure B, **1c** was obtained in 35% yield (2.213 g, 7.05 mmol) from the reaction of 2-fluorobenzaldehyde (2.500 g, 20.14 mmol) and 1-(4-chlorophenyl)-N-methylmethanamine[11] (3.450 g, 22.17 mmol) as yellow oil; ^1H NMR (400 MHz, $CDCl_3$) δ 2.80 (s, 3H), 3.74 (s, 3H), 4.46 (s, 2H), 6.88-6.93 (m, 2H), 7.28-7.36 (m, 5H), 7.54 (dd, J = 7.6, 1.6 Hz, 1H); ^{13}C NMR (100 MHz, $CDCl_3$) δ 39.5,

52.6, 59.8, 85.8, 86.4, 110.8, 118.0, 120.6, 128.4, 129.3, 131.8, 132.7, 136.0, 136.7, 154.6, 155.5; ESI-HRMS (*m/z*): $(M+H)^+$ Calcd for $C_{18}H_{17}ClNO_2$, 314.0948; Found: 314.0947.

Methyl 3-(2-((3-fluorobenzyl)(methyl)amino)phenyl)propiolate (1d)

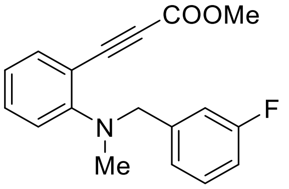

Following general procedure B, **1d** was obtained in 31% yield (1.859 g, 6.24 mmol) from the reaction of 2-fluorobenzaldehyde (2.500 g, 20.14 mmol) and 1-(3-fluorophenyl)-*N*-methylmethanamine[12] (3.100 g, 22.27 mmol) as orange oil; ^{1}H NMR (400 MHz, $CDCl_3$) δ 2.82 (s, 3H), 3.74 (s, 3H), 4.48 (s, 2H), 6.89-6.98 (m, 3H), 7.12 (d, J = 7.6 Hz, 1H), 7.18 (d, J = 10.0 Hz, 1H), 7.26-7.37 (m, 2H), 7.55 (dd, J = 7.6, 1.6 Hz, 1H); ^{13}C NMR (100 MHz, $CDCl_3$) δ 39.5, 52.5, 60.1, 85.8, 86.2, 110.7, 113.9 (d, J = 79.7 Hz), 114.8 (d, J = 81.6 Hz), 118.0, 120.6, 123.4, 129.6 (d, J = 30.5 Hz), 131.8, 136.0, 141.0 (d, J = 25.6 Hz), 154.6, 155.6, 163.0 (d, J = 243.8 Hz); ^{19}F NMR (376 MHz, $CDCl_3$) δ -113.6 (1F, m); ESI-HRMS (*m/z*): $(M+H)^+$ Calcd for $C_{18}H_{17}FNO_2$, 298.1243; Found: 298.1238.

Methyl 3-(2-((4-methoxybenzyl)(methyl)amino)phenyl)propiolate (1e)

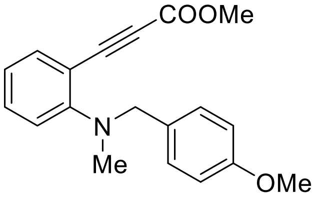

Following general procedure B, **1e** was obtained in 33% yield (2.060 g, 6.65 mmol) from the reaction of 2-fluorobenzaldehyde (2.500 g, 20.14 mmol) and 1-(4-methoxyphenyl)-*N*-methylmethanamine[13] (3.500 g, 23.15 mmol) as orange oil; ^{1}H NMR (400 MHz, $CDCl_3$) δ 2.80 (s, 3H), 3.77 (s, 3H), 3.80 (s, 3H), 4.45 (s, 2H), 6.84-6.90 (m, 4H), 7.25-7.27 (m, 2H), 7.32 (ddd, J = 8.8, 7.6, 1.6 Hz, 1H), 7.54 (dd, J = 7.6, 1.6 Hz, 1H); ^{13}C NMR (100 MHz, $CDCl_3$) δ 39.3, 52.6, 55.2, 59.7, 85.7, 86.8,

110.6, 113.6, 118.1, 120.3, 129.3, 130.0, 131.7, 136.0, 154.8, 155.8, 158.7; ESI-HRMS (*m/z*): $(M+H)^+$ Calcd for $C_{19}H_{20}NO_3$, 310.1443; Found: 310.1440.

Methyl 3-(2-(methyl(naphthalen-1-ylmethyl)amino)phenyl)propiolate (1f)

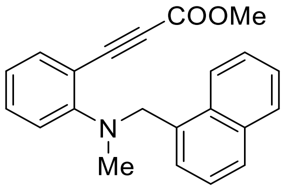

Following general procedure B, **1f** was obtained in 39% yield (2.592 g, 7.85 mmol) from the reaction of 2-fluorobenzaldehyde (2.500 g, 20.14 mmol) and *N*-methyl-1-(naphthalen-1-yl)methanamine[13] (3.800 g, 22.19 mmol) as yellow oil; ^1H NMR (400 MHz, $CDCl_3$) δ 3.02 (s, 3H), 3.56 (s, 3H), 5.01 (s, 2H), 6.86 (dd, *J* = 7.6, 7.6 Hz, 1H), 6.95 (d, *J* = 8.4 Hz, 1H), 7.29-7.34 (m, 1H), 7.44-7.58 (m, 5H), 7.80 (d, *J* = 8.0 Hz, 1H), 7.88-7.90 (m, 1H), 7.97-8.00 (m, 1H); ^{13}C NMR (100 MHz, $CDCl_3$) δ 40.4, 52.3, 57.3, 85.3, 87.0, 109.1, 117.3, 119.6, 123.1, 125.2, 125.4, 125.5, 125.9, 127.5, 128.6, 131.5, 131.7, 133.2, 133.7, 136.2, 154.6, 155.6; ESI-HRMS (*m/z*): $(M+H)^+$ Calcd for $C_{22}H_{20}NO_2$, 330.1494; Found: 330.1496.

Methyl 3-(2-(methyl(thiophen-2-ylmethyl)amino)phenyl)propiolate (1g)

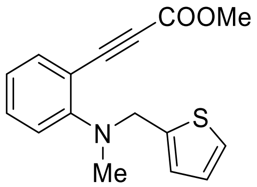

Following general procedure B, **1g** was obtained in 25% yield (1.440 g, 5.04 mmol) from the reaction of 2-fluorobenzaldehyde (2.500 g, 20.14 mmol) and *N*-methyl-1-(thiophen-2-yl)methanamine[14] (2.900 g, 22.80 mmol) as yellow oil; ^1H NMR (400 MHz, $CDCl_3$) δ 2.85 (s, 3H), 3.80 (s, 3H), 4.73 (s, 2H), 6.89-6.95 (m, 4H), 7.20 (dd, *J* = 4.8, 1.6 Hz, 1H), 7.33 (ddd, *J* = 8.8, 8.0, 1.6 Hz, 1H), 7.55 (dd, *J* = 7.6, 1.6 Hz, 1H); ^{13}C NMR (100 MHz, $CDCl_3$) δ 39.3, 52.6, 54.8, 85.9, 86.3, 111.2, 118.7, 120.9, 124.8, 126.1, 126.4, 131.6, 135.9, 140.7, 154.69, 154.75 ; ESI-HRMS (*m/z*): $(M-H)^+$ Calcd for $C_{16}H_{16}NO_2S$, 286.0902; Found: 286.0901.

Methyl 3-(2-(benzyl(phenethyl)amino)phenyl)propiolate (1m)

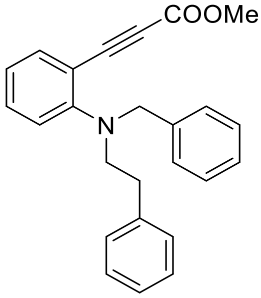

Following general procedure B, **1m** was obtained in 29% yield (2.160 g, 5.85 mmol) from the reaction of 2-fluorobenzaldehyde (2.500 g, 20.14 mmol) and *N*-benzyl-2-phenylethan-1-amine (4.700 g, 22.24 mmol) as orange oil; ^{1}H NMR (400 MHz, $CDCl_3$) δ 2.85-2.89 (m, 2H), 3.47-3.51 (m, 2H), 3.77 (s, 3H), 4.50 (s, 2H), 6.89 (ddd, J = 7.6, 7.6, 0.8 Hz, 1H), 6.96 (dd, J = 8.0, 0.8 Hz, 1 H), 7.10-7.12 (m, 2H), 7.14-7.31 (m, 9H), 7.55 (dd, J = 8.0, 1.6 Hz, 1H); ^{13}C NMR (100 MHz, $CDCl_3$) δ 33.8, 52.5, 53.8, 57.5, 85.4, 86.7, 112.2, 119.9, 120.8, 126.0, 127.0, 127.9, 128.26, 128.30, 128.8, 131.4, 136,1, 138.2, 139.6, 154.2, 154.7; ESI-HRMS (*m/z*): $(M+H)^+$ Calcd for $C_{25}H_{24}NO_2$, 370.1807; Found: 370.1778.

General procedure C:

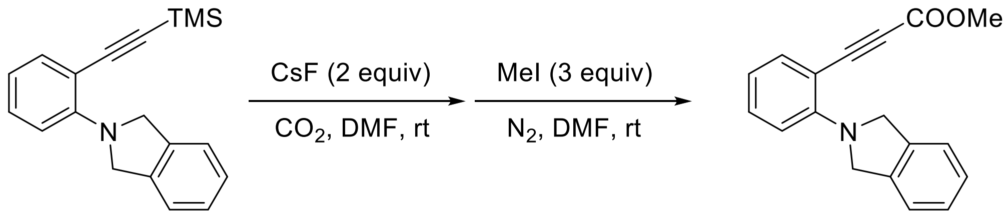

1p

To a solution of 2-(2-((trimethylsilyl)ethynyl)phenyl)isoindoline[15] (1.470 g, 5.05 mmol) in DMF was added CsF (1.533 g, 10.09 mmol) under CO_2 atmosphere, and the reaction mixture stirred at room temperature for 4 h. MeI (1.0 mL, 16.06 mmol) was added under nitrogen atmosphere and the reaction mixture was stirred at room temperature overnight. Quenched with water and the aqueous layer was extracted with ethyl acetate three times. The combined organic extracts were washed with water three times and brine, dried over Na_2SO_4, and concentrated in *vacuo*. The crude

residue was purified by flash column chromatography on silica gel (petroleum ether : ethyl acetate = 50:1) to give **1p** (926.2 mg) in 66% yield as a yellow solid, mp: 70-72 °C; ^{1}H NMR (400 MHz, $CDCl_3$) δ 3.85 (s, 3H), 5.06 (s, 4H), 6.68-6.72 (m, 2H), 7.29-7.36 (m, 5H), 7.50 (dd, J = 8.0, 2.0 Hz, 1H); ^{13}C NMR (100 MHz, $CDCl_3$) δ 52.5, 55.9, 83.9, 90.5, 102.2, 114.1, 116.6, 122.4, 127.2, 132.3, 136.9, 137.7, 149.9, 155.1; ESI-HRMS (*m/z*): $(M+H)^+$ Calcd for $C_{18}H_{16}NO_2$, 278.1181; Found: 278.1180.

Methyl 3-(2-(benzyl(3-methoxy-3-oxopropyl)amino)phenyl)propiolate (1l)

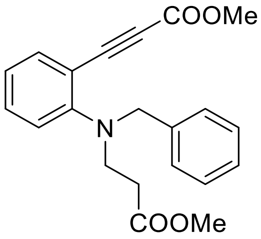

Following general procedure C, **1l** was obtained in 52% yield (571.9 mg, 1.63 mmol) from the reaction of methyl 3-(benzyl(2-((trimethylsilyl)ethynyl)phenyl)amino)propanoate (1.145 g, 3.13 mmol) (prepared from the reaction of methyl 3-((2-iodophenyl)amino)propanoate[16] (3.566 g, 11.69 mmol) following general procedure A) as a yellow solid, mp: 55-56 °C; ^{1}H NMR (400 MHz, $CDCl_3$) δ 2.59-2.62 (m, 2H), 3.58-3.62 (m, 5H), 3.80 (s, 3H), 4.46 (s, 2H), 6.89-6.94 (m, 2H), 7.22-7.32 (m, 6H), 7.54 (dd, J = 7.6, 1.6 Hz, 1H); ^{13}C NMR (100 MHz, $CDCl_3$) δ 32.7, 47.9, 51.6, 52.6, 57.4, 85.3, 86.3, 112.7, 120.1, 121.4, 127.2, 127.9, 128.3, 131.5, 136.0, 137.8, 153.8, 154.7, 172.5; ESI-HRMS (*m/z*): $(M+H)^+$ Calcd for $C_{21}H_{22}NO_4$, 352.1549; Found: 352.1545.

General procedure D:

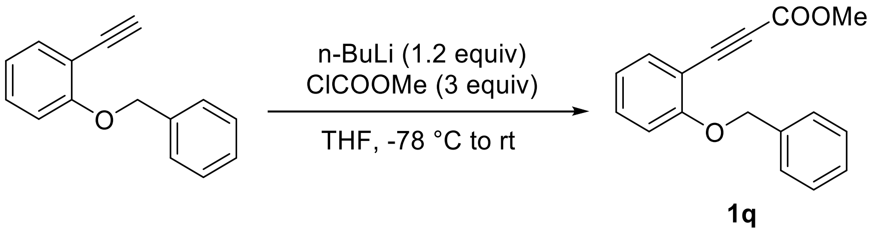

1q

To a solution of 1-(benzyloxy)-2-ethynylbenzene[17] (1.041 g, 5.00 mmol) in dry THF was added *n*-BuLi (3.8 mL, 1.6 M in hexanes) dropwise at -78 °C under nitrogen

atmosphere. Methyl carbonochloridate (1.1 mL, 15.00 mmol) was added after the reaction mixture was stirred at -78 °C for 30 min, then warmed to room temperature and stirred at room temperature for 30 min. The reaction mixture was quenched with water and the aqueous layer was extracted with ethyl acetate three times. The combined organic extracts were washed with brine, dried over Na_2SO_4, and concentrated in *vacuo*. The crude residue was purified by flash column chromatography on silica gel (petroleum ether : ethyl acetate = 50:1) to give **1q** (1.131 g) in 85% yield as a yellow solid, mp: 44-46 °C; ^{1}H NMR (400 MHz, $CDCl_3$) δ 3.84 (s, 3H), 5.20 (s, 2H), 6.92-6.97 (m, 2H), 7.30-7.41 (m, 4H), 7.48-7.50 (m, 2H), 7.54 (dd, *J* = 7.6, 1.6 Hz, 1H); ^{13}C NMR (100 MHz, $CDCl_3$) δ 52.7, 70.3, 83.6, 84.5, 109.6, 112.8, 120.9, 126.7, 127.8, 128.6, 132.2, 134.8, 136.4, 154.7, 160.7; ESI-HRMS (*m/z*): $(M+H)^+$ Calcd for $C_{17}H_{15}O_3$, 267.1021; Found: 267.1021.

Methyl 3-(2-(benzylthio)phenyl)propiolate (1r)

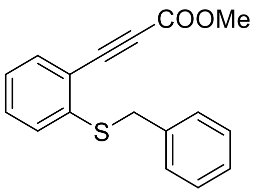

Following general procedure D, **1r** was obtained in 78% yield (1.178 g, 4.17 mmol) from the reaction of benzyl(2-ethynylphenyl)sulfane[18] (1.200 g, 5.35 mmol) as a yellow solid, mp: 90-92 °C; ^{1}H NMR (400 MHz, $CDCl_3$) δ 3.84 (s, 3H), 4.19 (s, 2H), 7.15 (ddd, *J* = 7.6, 7.2, 1.2 Hz, 1H), 7.24-7.36 (m, 7H), 7.54 (dd, *J* = 7.6, 1.2 Hz, 1H); ^{13}C NMR (100 MHz, $CDCl_3$) δ 37.7, 52.8, 83.9, 85.6, 119.6, 125.7, 127.3, 128.2, 128.5, 128.9, 130.8, 134.3, 136.2, 141.6, 154.3 ; ESI-HRMS (*m/z*): $(M+Na)^+$ Calcd for $C_{17}H_{14}NaO_2S$, 305.0612; Found: 305.0611.

Synthesis of methyl 3-(2-(benzyl(phenyl)amino)phenyl)propiolate (1n)

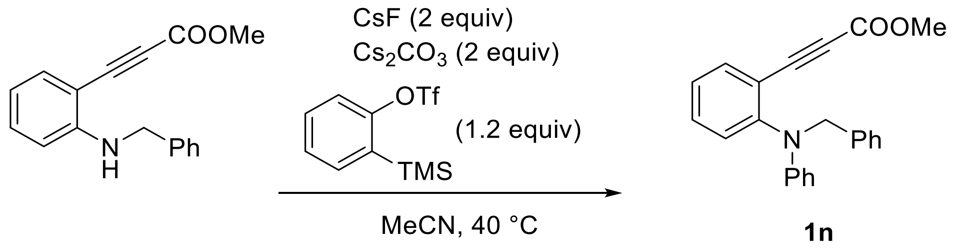

To a solution of methyl 3-(2-(benzylamino)phenyl)propiolate[2] (1.400 g, 5.28 mmol) in dry MeCN was added CsF (1.500 g, 10.57 mmol) and Cs_2CO_3 (3.500 g, 10.74 mmol) under N_2 atmosphere, then 2-(trimethylsilyl)phenyl trifluoromethanesulfonate (1.900 g, 6.37 mmol) was added and the reaction mixture stirred at 40 °C overnight. The reaction mixture was quenched with water and the aqueous layer was extracted with ethyl acetate three times. The combined organic extracts were washed with brine, dried over Na_2SO_4, and concentrated in *vacuo*. The crude residue was purified by flash column chromatography on silica gel (petroleum ether : ethyl acetate = 50:1) to give **1n** (1.370 g) in 76% yield as a yellow solid, mp: 55-57 °C; ^{1}H NMR (400 MHz, $CDCl_3$) δ 3.73 (s, 3H), 5.03 (s, 2H), 6.74-6.76 (m, 2H), 6.81 (t, J = 7.2 Hz, 1H), 7.13-7.17 (m, 3H), 7.22 (dd, J = 7.2, 7.2 Hz, 1H), 7.28-7.32 (m, 3H), 7.39-7.43 (m, 3H), 7.61 (dd, J = 8.0, 1.6 Hz, 1H); ^{13}C NMR (100 MHz, $CDCl_3$) δ 52.6, 56.6, 84.5, 85.3, 116.7, 117.8, 119.4, 124.9, 126.80, 126.83, 127.9, 128.4, 128.9, 131.9, 135.5, 138.7, 148.4, 151.4, 154.3; ESI-HRMS (*m/z*): $(M+Na)^+$ Calcd for $C_{23}H_{19}NNaO_2$, 364.1313; Found: 364.1302.

Synthesis of *N,N*-dibenzyl-2-(3,3,3-trifluoroprop-1-yn-1-yl)aniline (1s)

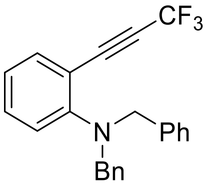

According to the literature[19], **1s** was obtained in 66% yield (485.6 mg, 1.33 mmol) from the reaction of CuI (76.10 mg, 0.40 mmol), Togni's reagent (1.000 g, 3.03 mmol), 1,10-Phen (145.0 mg, 0.80 mmol), $KHCO_3$ (80.6 mg, 0.81 mmol) and *N,N*-dibenzyl-2-ethynylaniline (600.0 mg, 2.02 mmol) as yellow oil; ^{1}H NMR (400 MHz, $CDCl_3$) δ 4.39 (s, 4H), 6.86-6.92 (m, 2H), 7.20-7.30 (m, 11H), 7.50 (dd, J = 7.6,

1.6 Hz, 1H); ^{13}C NMR (100 MHz, $CDCl_3$) δ 56.3, 80.4 (q, J = 51.6 Hz), 86.6 (q, J = 6.4 Hz), 111.7, 115.1 (q, J = 255.1 Hz), 120.9, 121.4, 127.1, 128.0, 128.3, 131.5, 135.2, 137.7, 154.3; ^{19}F NMR (376 MHz, $CDCl_3$) δ -49.4 (s, 3F); ESI-HRMS (*m/z*): $(M+H)^+$ Calcd for $C_{23}H_{19}FN_3$, 366.1470; Found: 366.1459.

2.2 NHC-BH_3/thiol Catalyst-Controlled Stereoselective Synthesis of Borylated Molecules Derived from RATC

General procedure E:

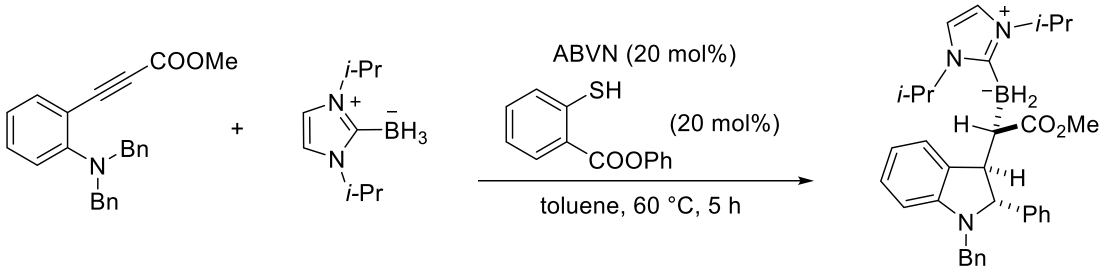

A solution of **1a** (150.0 mg, 0.422 mmol), **2b** (84.0 mg, 0.506 mmol), ABVN (20.9 mg, 0. 084 mmol) and phenyl 2-mercaptobenzoate (20.0 mg, 0.087 mmol) in toluene (4 mL) was stirred at 60 °C for 5 h under nitrogen atmosphere. After evaporation of solvent, the resulting crude residue was subjected to ^{1}H NMR analysis and the result showed that dr > 95:5. The crude material was purified by flash column chromatography (silica gel; petroleum ether : ethyl acetate = 2 : 1) to give product **3a-*i*-Pr** (205.0 mg) in 93% yield as a white solid; Recrystallization from petroleum ether/ethyl acetate gave colorless crystals.

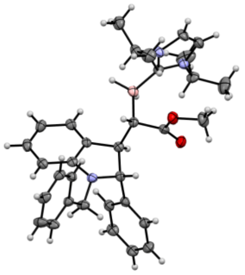

CCDC 1994058; mp: 60-61 °C; ^{1}H NMR (400 MHz, $CDCl_3$) δ 1.33 (d, J = 6.4 Hz, 6H), 1.34 (d, J = 6.8 Hz, 6H), 2.25-2.28 (m, BH_2CH, 1H), 3.28 (s, 3H), 3.65 (dd, J = 7.2, 5.6 Hz, 1H), 4.03 (d, J = 16.0 Hz, 1H), 4.37 (d, J = 16.0 Hz, 1H), 4.59 (d, J = 5.6 Hz, 1H), 4.89-4.95 (m, 2H), 6.28 (d, J = 7.6 Hz, 1H), 6.63 (dd, J = 7.6, 7.2 Hz, 1H),

6.92 (s, 2H), 7.00 (dd, J = 7.6, 7.6 Hz, 1H), 7.18-7.27 (m, 10H), 7.49 (d, J = 7.2 Hz, 1H); ^{13}C NMR (100 MHz, $CDCl_3$) δ 22.8, 23.3, 49.1, 50.0, 50.1, 53.4, 75.1, 104.9, 115.4, 116.1, 126.4, 126.8, 126.98, 127.02, 127.3, 127.5, 128.09, 128.14, 132.2, 139.3, 143.9, 151.9, 181.0; ^{11}B NMR (128.4 MHz, $CDCl_3$) δ -26.8 (t, J = 88.1 Hz, 1B); ESI-HRMS (*m/z*): $(M+H)^+$ Calcd for $C_{33}H_{41}{}^{11}BN_3O_2$, 522.3292; Found: 522.3300.

Supplementary Table 1. Crystal data and structure refinement for **3a-*i*-Pr**.

Empirical formula	$C_{33}H_{40}BN_3O_2$
Formula weight	521.49
Temperature/K	100.00(10)
Crystal system	monoclinic
Space group	$P2_1/c$
a/Å	30.9633(12)
b/Å	16.4655(6)
c/Å	11.8001(5)
α/°	90
β/°	94.550(4)
γ/°	90
Volume/Å^3	5997.0(4)
Z	8
ρ_{calc}g/cm^3	1.155
μ/mm^{-1}	0.071
F(000)	2240.0
Crystal size/mm^3	0.13 × 0.12 × 0.11
Radiation	MoKα (λ = 0.71073)
2Θ range for data collection/°	3.958 to 50
Index ranges	$-36 \le h \le 29$, $-19 \le k \le 15$, $-14 \le l \le 12$
Reflections collected	29484
Independent reflections	10546 [R_{int} = 0.0692, R_{sigma} = 0.0739]
Data/restraints/parameters	10546/0/729
Goodness-of-fit on F^2	1.051
Final R indexes [I>=2σ (I)]	R_1= 0.0569, wR_2 = 0.1311
Final R indexes [all data]	R_1 = 0.0842, wR_2 = 0.1525
Largest diff. peak/hole / e Å^{-3}	0.25/-0.28

((*S)-1-((2*R**,3*R**)-1-benzyl-2-phenylindolin-3-yl)-2-methoxy-2-oxoethyl)(1,3-dimethyl-1*H*-imidazol-3-ium-2-yl)dihydroborate (3a-Me)**

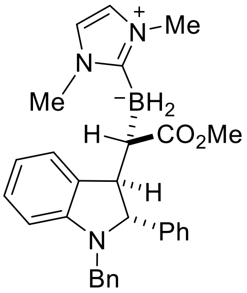

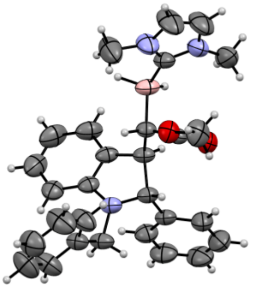

According to the optimization (Table 1, entry 1), the reaction of **1a** (150.0 mg, 0.422 mmol), **2a** (55.6 mg, 0.506 mmol), TBHN (15.0 mg, 0.086 mmol) in toluene (4 mL) was stirred at 50 °C for 4 h under nitrogen atmosphere. ^{1}H NMR analysis of the crude product showed 81% yield using the 1,1,2,2-tetrachloroethane as the internal standard and dr = 75:25. The two diastereomers were difficult to be separated by the flash column chromatography on silica gel but could be separated slightly by running the Pre-TLC (diethyl ether : petroleum ether = 10:1) several times. Recrystallization from petroleum ether/ethyl acetate gave colorless crystals; CCDC: 1994057; mp: 75-77 °C; ^{1}H NMR (400 MHz, $CDCl_3$) δ 2.25-2.28 (m, BH_2CH, 1H), 3.30 (s, 3H), 3.59 (s, 6H), 3.68 (dd, *J* = 6.4, 6.4 Hz, 1H), 3.98 (d, *J* = 16.0 Hz, 1H), 4.36 (d, *J* = 16.0 Hz, 1H), 4.60 (d, *J* = 6.8 Hz, 1H), 6.31 (d, *J* = 7.6 Hz, 1H), 6.59 (dd, *J* = 7.6, 7.2 Hz, 1H), 6.76 (s, 2H), 6.99 (dd, *J* = 7.6, 7.6 Hz, 1H), 7.19-7.34 (m, 10H), 7.40 (d, *J* = 7.2 Hz, 1H); ^{13}C NMR (100 MHz, $CDCl_3$) δ 35.8, 50.2, 50.3, 53.2, 74.5, 105.5, 116.4, 120.3, 126.50, 126.54, 126.9, 127.0, 127.5, 127.9, 128.1, 128.2, 132.3, 139.0, 143.8, 151.9, 181.4; ^{11}B NMR (128.4 MHz, $CDCl_3$) δ -27.0 (t, *J* = 87.5 Hz, 1B); ESI-HRMS (*m/z*): $(M+H)^+$ Calcd for $C_{29}H_{33}{}^{11}BN_3O_2$, 466.2666; Found: 466.2676.

Supplementary Table 2. Crystal data and structure refinement for **3a-Me**.

Empirical formula	$C_{29}H_{32}BN_3O_2$
Formula weight	465.38
Temperature/K	292(2)
Crystal system	monoclinic
Space group	$P2_1/c$
a/Å	11.0277(2)
b/Å	8.4429(2)
c/Å	28.3272(4)
α/°	90

β/°	95.649(2)
γ/°	90
Volume/Å^3	2624.62(9)
Z	4
ρ_{calc}g/cm^3	1.178
μ/mm^{-1}	0.579
F(000)	992.0
Crystal size/mm^3	0.250 × 0.220 × 0.180
Radiation	CuKα (λ = 1.54184)
2Θ range for data collection/°	8.056 to 142.612
Index ranges	$-11 \leq h \leq 13$, $-9 \leq k \leq 10$, $-34 \leq l \leq 33$
Reflections collected	9836
Independent reflections	4940 [R_{int} = 0.0157, R_{sigma} = 0.0169]
Data/restraints/parameters	4940/0/327
Goodness-of-fit on F^2	1.046
Final R indexes [I>=2σ (I)]	R_1 = 0.0501, wR_2 = 0.1409
Final R indexes [all data]	R_1 = 0.0586, wR_2 = 0.1492
Largest diff. peak/hole / e Å^{-3}	0.17/-0.18

((*R)-1-((2*R**,3*R**)-1-benzyl-2-phenylindolin-3-yl)-2-methoxy-2-oxoethyl)(1,3-dimethyl-1*H*-imidazol-3-ium-2-yl)dihydroborate (4a-Me)**

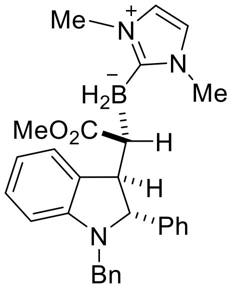

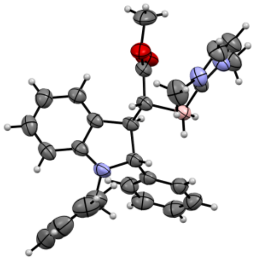

Recrystallization from petroleum ether/ethyl acetate gave colorless crystals, CCDC: 1994059; mp: 150-153 °C; ^{1}H NMR (400 MHz, $CDCl_3$) δ 2.17-2.20 (m, BH_2CH, 1H), 3.34 (s, 3H), 3.61-3.64 (m, 7H), 4.02 (d, *J* = 16.0 Hz, 1H), 4.47 (d, *J* = 16.0 Hz, 1H), 4.98 (d, *J* = 3.6 Hz, 1H), 6.32 (d, *J* = 7.6 Hz, 1H), 6.52 (dd, *J* = 7.6, 7.2 Hz, 1H), 6.76 (s, 2H), 7.00 (dd, *J* = 7.6, 7.6 Hz, 1H), 7.04 (d, *J* = 7.2 Hz, 1H), 7.17-7.30 (m, 10H); ^{13}C NMR (100 MHz, $CDCl_3$) δ 35.8, 49.0, 50.1, 52.9, 70.6, 104.3, 116.2, 120.3, 124.5, 126.5, 126.6, 127.3 (overlapped), 127.4, 128.1, 128.2, 134.1, 139.4, 144.3, 151.1, 180.9; ^{11}B NMR (128.4 MHz, $CDCl_3$) δ -26.5 (t, *J* = 87.2 Hz, 1B); ESI-HRMS (*m/z*): $(M+H)^+$ Calcd for $C_{29}H_{33}{}^{11}BN_3O_2$, 466.2666; Found: 466.2674.

Supplementary Table 3. Crystal data and structure refinement for **4a-Me**.

Empirical formula	$C_{29}H_{32}BN_3O_2$
Formula weight	465.38
Temperature/K	291(2)
Crystal system	triclinic
Space group	P-1
a/Å	9.7741(3)
b/Å	11.3557(4)
c/Å	12.9061(3)
α/°	74.599(3)
β/°	83.319(2)
γ/°	71.395(3)
Volume/Å^3	1308.06(8)
Z	2
ρ_{calc}g/cm^3	1.182
μ/mm^{-1}	0.580
F(000)	496.0
Crystal size/mm^3	0.21 × 0.019 × 0.016
Radiation	CuKα (λ = 1.54184)
2Θ range for data collection/°	7.108 to 142.594
Index ranges	$-11 \leq h \leq 11$, $-13 \leq k \leq 13$, $-15 \leq l \leq 9$
Reflections collected	8676
Independent reflections	4914 [R_{int} = 0.0122, R_{sigma} = 0.0142]
Data/restraints/parameters	4914/0/328
Goodness-of-fit on F^2	1.047
Final R indexes [I>=2σ (I)]	R_1 = 0.0415, wR_2 = 0.1188
Final R indexes [all data]	R_1 = 0.0454, wR_2 = 0.1230
Largest diff. peak/hole / e Å^{-3}	0.17/-0.15

(1,3-Diisopropyl-1*H*-imidazol-3-ium-2-yl)((*S)-2-methoxy-1-((2*R**,3*R**)-1-methyl-2-phenylindolin-3-yl)-2-oxoethyl)dihydroborate (3b)**

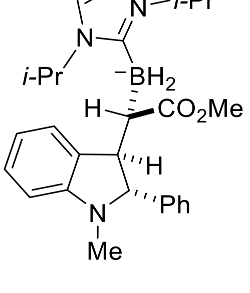

According to the general procedure E, the reaction of methyl 3-(2-(benzyl(methyl)amino)phenyl)propiolate (103.4 mg, 0.370 mmol), **2b** (73.7 mg, 0.444 mmol), ABVN (19.0 mg, 0.077 mmol) and phenyl 2-mercaptobenzoate (17.3 mg, 0.075 mmol) in toluene (4 mL) was stirred at 60 °C for 3 h under nitrogen atmosphere. ^{1}H NMR analysis of the crude product showed that dr = 94:6. Flash column chromatography on silica gel (petroleum ether : ethyl acetate = 3 : 1) afforded 156.6 mg (95%) **3b** as a grey solid; Recrystallization from petroleum ether/ethyl acetate gave colorless crystals; mp: 135-136 °C; ^{1}H NMR (400 MHz, $CDCl_3$) δ 1.33 (d, *J* = 6.8 Hz, 6H), 1.34 (d, *J* = 6.8 Hz, 6H), 2.25-2.26 (m, BH_2CH, 1H), 2.63 (s, 3H), 3.23 (s, 3H), 3.60 (dd, *J* = 6.8, 6.4 Hz, 1H), 4.42 (d, *J* = 6.4 Hz, 1H), 4.90-4.99 (m, 2H), 6.39 (d, *J* = 7.6 Hz, 1H), 6.63 (ddd, *J* = 7.6, 7.2, 0.8 Hz, 1H), 6.90 (s, 2H), 7.08 (dd, *J* = 7.6, 7.6 Hz, 1H), 7.18-7.33 (m, 5H), 7.48 (d, *J* = 7.2 Hz, 1H); ^{13}C NMR (100 MHz, $CDCl_3$) δ 22.8, 23.3, 33.2, 49.1, 49.8, 53.1, 76.6, 104.9, 115.4, 116.2, 126.5, 126.8, 127.0, 127.5, 128.0, 132.4, 143.9, 152.7, 180.9; ^{11}B NMR (128.4 MHz, $CDCl_3$) δ -26.9 (t, *J* = 89.7 Hz, 1B); ESI-HRMS (*m/z*): $(M+H)^+$ Calcd for $C_{27}H_{37}{}^{11}BN_3O_2$, 446.2979; Found: 446.2985.

((*S)-1-((2*R**,3*R**)-2-(4-chlorophenyl)-1-methylindolin-3-yl)-2-methoxy-2-oxoethyl)(1,3-diisopropyl-1*H*-imidazol-3-ium-2-yl)dihydroborate (3c)**

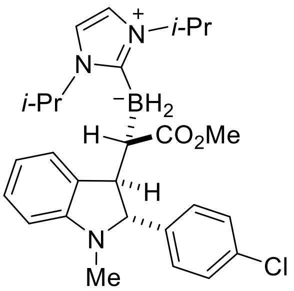

According to the general procedure E, the reaction of methyl 3-(2-((4-chlorobenzyl)(methyl)amino)phenyl)propiolate (123.6 mg, 0.394 mmol), **2b** (78.6 mg, 0.473 mmol), ABVN (20.4 mg, 0.082 mmol) and phenyl 2-mercaptobenzoate (18.4 mg, 0.080 mmol) in toluene (4 mL) was stirred at 60 °C for 3h under nitrogen. ^{1}H NMR analysis of the crude product showed that dr = 94:6. Flash column chromatography on silica gel (petroleum ether : ethyl acetate = 3 : 1)

afforded 164.0 mg (87%) **3c** as a grey solid; Recrystallization from petroleum ether/ethyl acetate gave colorless crystals; mp: 142-143 °C; ^{1}H NMR (400 MHz, $CDCl_3$) δ 1.35 (d, *J* = 6.8 Hz, 12H), 2.27-2.29 (m, BH_2CH, 1H), 2.63 (s, 3H), 3.22 (s, 3H), 3.52 (dd, *J* = 6.8, 6.4 Hz, 1H), 4.47 (d, *J* = 6.0 Hz, 1H), 4.91-4.98 (m, 2H), 6.40 (d, *J* = 8.0 Hz, 1H), 6.64 (dd, *J* = 7.2, 7.2 Hz, 1H), 6.93 (s, 2H), 7.09 (dd, *J* = 7.6, 7.6 Hz, 1H), 7.22-7.28 (m, 4H), 7.42 (d, *J* = 7.6 Hz, 1H); ^{13}C NMR (100 MHz, $CDCl_3$) δ 22.7, 23.3, 33.2, 49.1, 49.8, 53.4, 75.5, 104.9, 115.4, 116.3, 126.1, 127.1, 128.1, 128.9, 132.21, 132.22, 142.9, 152.4, 180.7; ^{11}B NMR (128.4 MHz, $CDCl_3$) δ -26.8 (t, *J* = 89.2 Hz, 1B); ESI-HRMS (*m/z*): $(M+H)^+$ Calcd for $C_{27}H_{36}{}^{11}BClN_3O_2$, 480.2589; Found: 480.2585.

(1,3-Diisopropyl-1*H*-imidazol-3-ium-2-yl)((*S)-1-((2*R**,3*R**)-2-(3-fluorophenyl)-1-methylindolin-3-yl)-2-methoxy-2-oxoethyl)dihydroborate (3d)**

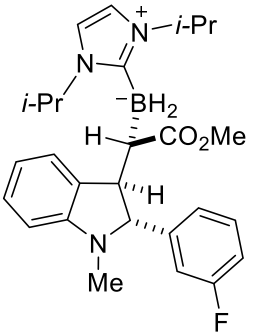

According to the general procedure E, the reaction of methyl 3-(2-((3-fluorobenzyl)(methyl)amino)phenyl)propiolate (123.3 mg, 0.415 mmol), **2b** (82.9 mg, 0.499 mmol), ABVN (20.7 mg, 0.083 mmol) and phenyl 2-mercaptobenzoate (20.2 mg, 0.088 mmol) in toluene (4 mL) was stirred at 60 °C for 4 h under nitrogen atmosphere. ^{1}H NMR analysis of the crude product showed that dr = 94:6. Flash column chromatography on silica gel (petroleum ether : ethyl acetate = 2 : 1) afforded 182.3 mg (95%) **3d** as a grey solid; Recrystallization from petroleum ether/ethyl acetate gave colorless crystals; mp: 126-128 °C; ^{1}H NMR (400 MHz, $CDCl_3$) δ 1.36 (d, *J* = 6.8 Hz, 6H), 1.37 (d, *J* = 6.8 Hz, 6H), 2.26-2.29 (m, BH_2CH, 1H), 2.66 (s, 3H), 3.25 (s, 3H), 3.56 (dd, *J* = 6.8, 6.4 Hz, 1H), 4.49 (d, *J* = 6.4 Hz, 1H), 4.93-5.00 (m, 2H), 6.41 (d, *J* = 7.6 Hz, 1H), 6.64 (ddd, *J* = 7.6, 7.2, 0.8 Hz, 1H),

6.87-6.93 (m, 3H), 7.03-7.14 (m, 3H), 7.20-7.24 (m, 1H), 7.44 (d, *J* = 7.6 Hz, 1H); ^{13}C NMR (100 MHz, $CDCl_3$) δ 22.8, 23.3, 33.3, 49.2, 49.9, 53.3, 75.9, 105.0, 113.6 (d, *J* = 21.1 Hz), 114.3 (d, *J* = 21.1 Hz), 115.5, 116.4, 123.2, 126.3, 127.2, 129.4 (d, *J* = 32.0 Hz), 132.3, 147.2 (d, *J* = 6.2 Hz), 152.5, 162.7 (d, *J* = 243.3 Hz), 180.8; ^{11}B NMR (128.4 MHz, $CDCl_3$) δ -26.8 (t, *J* = 87.6 Hz, 1B); ^{19}F NMR (376 MHz, $CDCl_3$): δ -113.8-113.7 (1F, m); ESI-HRMS (*m/z*): $(M+H)^+$ Calcd for $C_{27}H_{36}{}^{11}BFN_3O_2$, 464.2885; Found: 464.2888.

(1,3-Diisopropyl-1*H*-imidazol-3-ium-2-yl)((*S)-2-methoxy-1-((2*R**,3*R**)-2-(4-methoxyphenyl)-1-methylindolin-3-yl)-2-oxoethyl)dihydroborate (3e)**

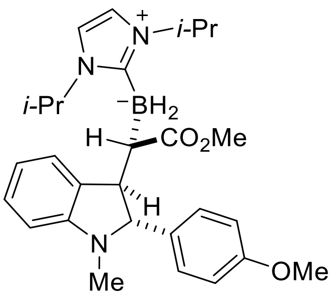

According to the general procedure E, the reaction of methyl 3-(2-((4-methoxybenzyl)(methyl)amino)phenyl)propiolate (124.2 mg, 0.402 mmol), **2b** (80.7 mg, 0.486 mmol), ABVN (21.4 mg, 0.086 mmol) and phenyl 2-mercaptobenzoate (19.1 mg, 0.083 mmol) in toluene (4 mL) was stirred at 60 °C for 4 h under nitrogen atmosphere. ^{1}H NMR analysis of the crude product showed that dr = 94:6. Flash column chromatography on silica gel (petroleum ether : ethyl acetate = 5 : 1) afforded 160.7 mg (85%) **3e** as a grey solid; Recrystallization from petroleum ether/ethyl acetate gave colorless crystals; mp: 126-128 °C; ^{1}H NMR (400 MHz, $CDCl_3$) δ 1.35 (d, *J* = 6.8 Hz, 6H), 1.36 (d, *J* = 6.8 Hz, 6H), 2.21-2.26 (m, BH_2CH, 1H), 2.61 (s, 3H), 3.23 (s, 3H), 3.57 (dd, *J* = 6.8, 6.8 Hz, 1H), 3.78 (s, 3H), 4.36 (d, *J* = 6.8 Hz, 1H), 4.93-5.00 (m, 2H), 6.39 (d, *J* = 7.6 Hz, 1H), 6.63 (ddd, *J* = 7.6, 7.2, 0.8 Hz, 1H), 6.81 (d, *J* = 8.8 Hz, 2H), 6.92 (s, 2H), 7.08 (dd, *J* = 7.6, 7.6 Hz, 1H), 7.24 (d, *J* = 8.8 Hz, 2H), 7.48 (d, *J* = 7.2 Hz, 1H); ^{13}C NMR (100 MHz, $CDCl_3$) δ 22.9, 23.4, 33.3, 49.2, 49.9, 53.1, 55.2, 76.2, 105.1, 113.4, 115.4, 116.3, 126.5, 127.0, 128.7,

132.6, 136.1, 152.7, 158.5, 181.0; ^{11}B NMR (128.4 MHz, $CDCl_3$) δ -27.0 (t, *J* = 88.5 Hz, 1B); ESI-HRMS (*m/z*): $(M+H)^+$ Calcd for $C_{28}H_{39}{}^{11}BN_3O_3$, 476.3084; Found: 476.3093.

(1,3-Diisopropyl-1*H*-imidazol-3-ium-2-yl)((*S)-2-methoxy-1-((2*R**,3*R**)-1-methyl-2-(naphthalen-1-yl)indolin-3-yl)-2-oxoethyl)dihydroborate (3f)**

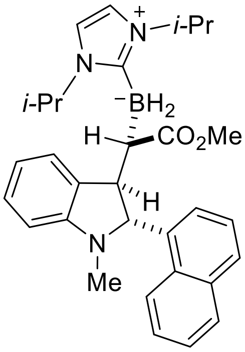

According to the general procedure E, the reaction of 5-(((2-(3-methoxy-3-oxoprop-1-yn-1-yl)phenyl)(methyl)amino)methyl)naphthalen-2-ylium (124.5 mg, 0.378 mmol), **2b** (75.8 mg, 0.456 mmol), ABVN (18.7 mg, 0.075 mmol) and phenyl 2-mercaptobenzoate (17.4 mg, 0.076 mmol) in toluene (4 mL) was stirred at 60 °C for 4 h under nitrogen atmosphere. ^{1}H NMR analysis of the crude product showed that dr = 92:8. Flash column chromatography on silica gel (petroleum ether : ethyl acetate = 2 : 1) afforded 131.0 mg (70%) **3f** as a grey solid; Recrystallization from petroleum ether/ethyl acetate gave colorless crystals; mp: 81-82 °C; ^{1}H NMR (400 MHz, $CDCl_3$) δ 1.27 (d, *J* = 6.8 Hz, 6H), 1.32 (d, *J* = 6.8 Hz, 6H), 2.27-2.28 (m, BH_2CH, 1H), 2.61 (s, 3H), 3.07 (s, 3H), 4.02-4.06 (m, 1H), 4.88-4.95 (m, 2H), 5.18 (s br, 1H), 6.48 (d, *J* = 7.6 Hz, 1H), 6.72 (ddd, *J* = 7.6, 7.2, 1.2 Hz, 1H), 6.88 (s, 2H), 7.14 (dd, *J* = 7.6, 7.6 Hz, 1H), 7.37-7.46 (m, 3H), 7.59 (dd, *J* = 7.2, 6.4 Hz, 2H), 7.76 (d, *J* = 8.4 Hz, 1H), 7.85 (dd, *J* = 8.0 Hz, 1.6 Hz, 1H), 8.43 (d, *J* = 8.4 Hz, 1H); ^{13}C NMR (100 MHz, $CDCl_3$) δ 22.8, 23.2, 33.9, 49.1, 49.8, 76.7, 105.9, 115.4 (overlapped), 116.7, 125.1, 125.2, 125.3, 126.5, 127.2, 127.8, 128.7, 132.3, 132.7, 134.1, 139.5, 152.8, 180.8; ^{11}B NMR (128.4 MHz, $CDCl_3$) δ -27.3 (t, *J* = 88.6 Hz, 1B); ESI-HRMS (*m/z*): $(M+H)^+$ Calcd for $C_{31}H_{39}{}^{11}BN_3O_2$, 496.3135; Found: 496.3143.

(1,3-Diisopropyl-1*H*-imidazol-3-ium-2-yl)((*S)-2-methoxy-1-((2*R**,3*R**)-1-methyl-2-(thiophen-2-yl)indolin-3-yl)-2-oxoethyl)dihydroborate (3g)**

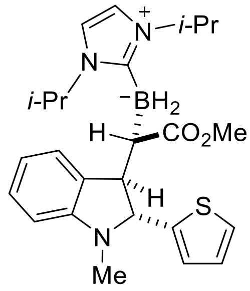

According to the general procedure E, the reaction of methyl 3-(2-(methyl(thiophen-2-ylmethyl)amino)phenyl)propiolate (119.0 mg, 0.417 mmol), **2b** (83.4 mg, 0.502 mmol), ABVN (21.3 mg, 0.086 mmol) and phenyl 2-mercaptobenzoate (20.4 mg, 0.089 mmol) in toluene (4 mL) was stirred at 60 °C for 4 h under nitrogen atmosphere. ^{1}H NMR analysis of the crude product showed that dr = 94:6. Flash column chromatography on silica gel (petroleum ether : ethyl acetate = 2 : 1) afforded 150.9 mg (80%) **3g** as a grey solid; Recrystallization from petroleum ether/ethyl acetate gave colorless crystals; mp: 135-136 °C; ^{1}H NMR (400 MHz, $CDCl_3$) δ 1.36 (d, *J* = 6.8 Hz, 6H), 1.37 (d, *J* = 6.8 Hz, 6H), 2.27-2.29 (m, BH_2CH, 1H), 2.69 (s, 3H), 3.29 (s, 3H), 3.65 (dd, *J* = 6.8, 6.4 Hz, 1H), 4.73 (d, *J* = 6.4 Hz, 1H), 4.94-5.00 (m, 2H), 6.44 (d, *J* = 7.6 Hz, 1H), 6.68 (ddd, *J* = 7.6, 7.2, 1.2 Hz, 1H), 6.90-6.92 (m, 3H), 6.98 (dd, *J* = 3.6, 1.2 Hz, 1H), 7.08-7.11 (m, 1H), 7.14-7.15 (m, 1H), 7.49 (d, *J* = 7.2 Hz, 1H); ^{13}C NMR (100 MHz, $CDCl_3$) δ 22.8, 23.3, 33.7, 49.2, 50.0, 53.7, 72.0, 106.1, 115.4, 117.0, 123.8, 124.9, 126.0, 126.4, 127.1, 132.4, 148.1, 152.0, 180.9; ^{11}B NMR (128.4 MHz, $CDCl_3$) δ -27.0 (t, *J* = 88.2 Hz, 1B); ESI-HRMS (*m/z*): $(M+H)^+$ Calcd for $C_{25}H_{35}{}^{11}BN_3O_2S$, 452.2543; Found: 452.2538.

((*S)-1-((2*R**,3*R**)-1-benzyl-5-fluoro-2-phenylindolin-3-yl)-2-methoxy-2-oxoethyl)(1,3-diisopropyl-1*H*-imidazol-3-ium-2-yl)dihydroborate (3h)**

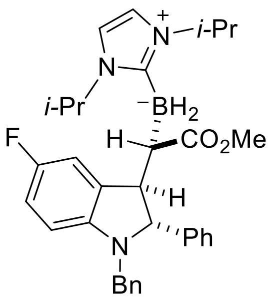

According to the general procedure E, the reaction of methyl 3-(2-(dibenzylamino)-5-fluorophenyl)propiolate (131.2 mg, 0.351 mmol), **2b** (69.3 mg, 0.417 mmol), ABVN (17.7 mg, 0.071 mmol) and phenyl 2-mercaptobenzoate (18.0 mg, 0.078 mmol) in toluene (4 mL) was stirred at 60 °C for 3 h under nitrogen. ^{1}H NMR analysis of the crude product showed that dr = 93:7. Flash column chromatography on silica gel (petroleum ether : ethyl acetate = 2 : 1) afforded 171.4 mg (90%) **3h** as a grey solid; Recrystallization from petroleum ether/ethyl acetate gave colorless crystals; mp: 68-70 °C; ^{1}H NMR (400 MHz, $CDCl_3$) δ 1.34 (d, *J* = 6.4 Hz, 6H), 1.34 (d, *J* = 6.4 Hz, 6H), 2.16-2.20 (m, BH_2CH, 1H), 3.29 (s, 3H), 3.65 (dd, *J* = 6.8, 6.8 Hz, 1H), 3.99 (d, *J* = 16.0 Hz, 1H), 4.29 (d, *J* = 16.0 Hz, 1H), 4.59 (d, *J* = 6.4 Hz, 1H), 4.86-4.95 (m, 2H), 6.12 (dd, *J* = 8.4, 4.4 Hz, 1H), 6.66 (ddd, *J* = 9.2, 8.8, 2.4 Hz, 1H), 6.92 (s, 2H), 7.16-7.32 (m, 11H); ^{13}C NMR (100 MHz, $CDCl_3$) δ 22.8, 23.3, 49.2, 50.1, 51.2, 53.2, 75.7, 105.0 (d, *J* = 8.0 Hz), 112.4 (d, *J* = 22.8 Hz), 114.8 (d, *J* = 24.2 Hz), 115.5, 126.5, 127.1, 127.3, 127.8, 128.17, 128.22, 134.0 (d, *J* = 8.0 Hz), 139.2, 143.5, 148.3, 155.9 (d, *J* = 230.7 Hz), 180.9; ^{11}B NMR (128.4 MHz, $CDCl_3$) δ -27.1 (t, *J* = 88.2 Hz, 1B); ^{19}F NMR (376 MHz, $CDCl_3$) δ -129.2-129.1 (1F, m); ESI-HRMS (*m/z*): $(M+H)^+$ Calcd for $C_{33}H_{40}{}^{11}BFN_3O_2$, 540.3198; Found: 540.3193.

((*S)-1-((2*R**,3*R**)-1-benzyl-5-chloro-2-phenylindolin-3-yl)-2-methoxy-2-oxoethyl)(1,3-diisopropyl-1*H*-imidazol-3-ium-2-yl)dihydroborate (3i)**

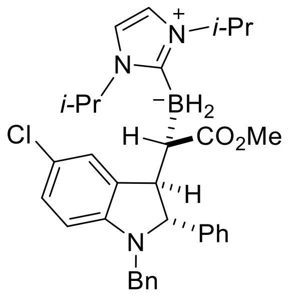

According to the general procedure E, the reaction of methyl 3-(5-chloro-2-(dibenzylamino)phenyl)propiolate (180.5 mg, 0.463 mmol), **2b** (80.0 mg, 0.482 mmol), ABVN (20.3 mg, 0.082 mmol) and phenyl 2-mercaptobenzoate (19.8 mg, 0.086 mmol) in toluene (4 mL) was stirred at 60 °C for 2 h under nitrogen atmosphere. ^{1}H NMR analysis of the crude product showed that dr > 95:5. Flash column chromatography on silica gel (petroleum ether : ethyl acetate = 2 : 1) afforded 231.5 mg (90%) **3i** as a grey solid; Recrystallization from petroleum ether/ethyl acetate gave colorless crystals; mp: 166-167 °C; ^{1}H NMR (400 MHz, $CDCl_3$) δ 1.33 (d, *J* = 7.2 Hz, 6H), 1.35 (d, *J* = 7.2 Hz, 6H), 2.19-2.20 (m, BH_2CH, 1H), 3.31 (s, 3H), 3.60 (dd, *J* = 6.4, 6.4 Hz, 1H), 4.01 (d, *J* = 16.0 Hz, 1H), 4.33 (d, *J* = 16.0 Hz, 1H), 4.62 (d, *J* = 5.2 Hz, 1H), 4.85-4.92 (m, 2H), 6.16 (d, *J* = 8.4 Hz, 1H), 6.92-6.95 (m, 3H), 7.18-7.26 (m, 9H), 7.46 (d, *J* = 1.2 Hz, 1H); ^{13}C NMR (100 MHz, $CDCl_3$) δ 22.8, 23.3, 49.2, 50.11, 50.12, 53.2, 75.1, 105.5, 115.5, 120.7, 126.6, 126.7, 127.10, 127.11, 127.2, 127.4, 128.2, 128.3, 134.2, 138.8, 143.4, 150.5, 180.9; ^{11}B NMR (128.4 MHz, $CDCl_3$) δ -27.0 (t, *J* = 92.2 Hz, 1B); ESI-HRMS (*m/z*): $(M+H)^+$ Calcd for $C_{33}H_{40}{}^{11}BClN_3O_2$, 556.2902; Found: 556.2907.

((*S)-1-((2*R**,3*R**)-1-benzyl-2-phenyl-2,3-dihydro-1*H*-pyrrolo[2,3-b]pyridin-3-yl)-2-methoxy-2-oxoethyl)(1,3-diisopropyl-1*H*-imidazol-3-ium-2-yl)dihydroborate (3j)**

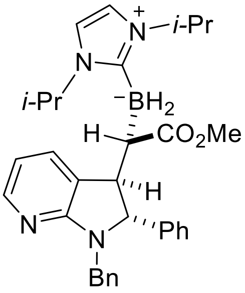

According to the general procedure E, the reaction of methyl 3-(2-(dibenzylamino)pyridin-3-yl)propiolate (141.0 mg, 0.396 mmol), **2b** (79.2 mg, 0.477 mmol), ABVN (20.5 mg, 0.083 mmol) and phenyl 2-mercaptobenzoate (19.7 mg, 0.086 mmol) in toluene (4 mL) was stirred at 60 °C for 3 h under nitrogen atmosphere. ^{1}H NMR analysis of the crude product showed that dr = 89:11. Flash column chromatography on silica gel (petroleum ether : ethyl acetate = 2 : 1) afforded 186.0 mg (90%) **3j** as a grey solid; Recrystallization from petroleum ether/ethyl acetate gave colorless crystals; mp: 130-132 °C; NMR data shown below was only given for the major one. ^{1}H NMR (400 MHz, $CDCl_3$) δ 1.32 (d, *J* = 6.4 Hz, 6H), 1.32 (d, *J* = 6.8 Hz, 6H), 2.08-2.11 (m, BH_2CH, 1H), 3.25 (s, 3H), 3.53 (dd, *J* = 7.6, 4.8 Hz, 1H), 3.83 (d, *J* = 15.6 Hz, 1H), 4.42 (d, *J* = 4.4 Hz, 1H), 4.81-4.87 (m, 2H), 5.20 (d, *J* = 15.6 Hz, 1H), 6.48 (dd, *J* = 7.2, 5.2 Hz, 1H), 6.91 (s, 2H), 7.14-7.28 (m, 10H), 7.63 (ddd, *J* = 7.2, 1.6, 1.2 Hz, 1H), 7.93 (ddd, *J* = 5.2, 1.6, 0.8 Hz, 1H); ^{13}C NMR (100 MHz, $CDCl_3$) δ 22.8, 23.3, 45.6, 49.2, 50.0, 51.3, 70.4, 111.7, 115.5, 125.4, 126.5, 127.08, 127.12, 127.9, 128.1, 128.3, 133.8, 138.4, 142.8, 145.5, 162.4, 180.7; ^{11}B NMR (128.4 MHz, $CDCl_3$) δ -27.0 (t, *J* = 87.6 Hz, 1B); ESI-HRMS (*m/z*): $(M+H)^+$ Calcd for $C_{32}H_{40}{}^{11}BN_4O_2$, 523.3244; Found: 523.3245.

((*S)-1-((2*R**,3*R**)-1-(but-3-en-1-yl)-2-phenylindolin-3-yl)-2-methoxy-2-oxoethyl) (1,3-diisopropyl-1*H*-imidazol-3-ium-2-yl)dihydroborate (3k)**

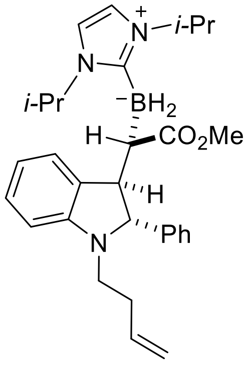

According to the general procedure E, the reaction of methyl 3-(2-(benzyl(but-3-en-1-yl)amino)phenyl)propiolate (112.4 mg, 0.352 mmol), **2b** (70.2 mg, 0.423 mmol), ABVN (17.9 mg, 0.072 mmol) and phenyl 2-mercaptobenzoate (17.0 mg, 0.074 mmol) in toluene (4 mL) was stirred at 60 °C for 4 h under nitrogen atmosphere. ^{1}H NMR analysis of the crude product showed that dr > 95:5. Flash column chromatography on silica gel (petroleum ether : ethyl acetate = 2 : 1) afforded 137.9 mg (81%) **3k** as a grey solid; Recrystallization from petroleum ether/ethyl acetate gave colorless crystals; mp: 29-30 °C; ^{1}H NMR (400 MHz, $CDCl_3$) δ 1.32 (d, *J* = 6.8 Hz, 6H), 1.33 (d, *J* = 6.4 Hz, 6H), 2.19-2.27 (m, 3H), 2.92-3.00 (m, 1H), 3.23-3.30 (m, 4H), 3.52 (dd, *J* = 5.6, 5.2 Hz, 1H), 4.62 (d, *J* = 4.8 Hz, 1H), 4.88-5.02 (m, 4H), 5.71-5.81 (m, 1H), 6.39 (d, *J* = 8.0 Hz, 1H), 6.58 (dd, *J* = 7.2, 7.2 Hz, 1H), 6.89 (s, 2H), 7.06 (dd, *J* = 7.6, 7.6 Hz, 1H), 7.16-7.29 (m, 5H), 7.41 (d, *J* = 7.2 Hz, 1H); ^{13}C NMR (100 MHz, $CDCl_3$) δ 22.7, 23.2, 31.5, 45.3, 49.0, 49.9, 53.5, 73.7, 104.0, 115.4, 115.5, 115.6, 126.6, 126.7, 127.0, 127.2, 128.0, 131.9, 136.5, 144.5, 151.5, 180.9; ^{11}B NMR (128.4 MHz, $CDCl_3$) δ -26.8 (t, *J* = 86.5 Hz, 1B); ESI-HRMS (*m/z*): $(M+H)^+$ Calcd for $C_{30}H_{41}{}^{11}BN_3O_2$, 486.3292; Found: 486.3294.

(1,3-Diisopropyl-1*H*-imidazol-3-ium-2-yl)((*S)-2-methoxy-1-((2*R**,3*R**)-1-(3-methoxy-3-oxopropyl)-2-phenylindolin-3-yl)-2-oxoethyl)dihydroborate (3l)**

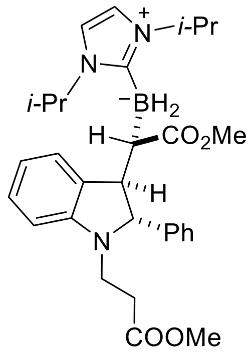

According to the general procedure E, the reaction of methyl 3-(2-(benzyl(3-methoxy-3-oxopropyl)amino)phenyl)propiolate (147.2 mg, 0.419 mmol), **2b** (83.4 mg, 0.502 mmol), ABVN (21.3 mg, 0.086 mmol) and phenyl 2-mercaptobenzoate (19.4 mg, 0.084 mmol) in toluene (4 mL) was stirred at 60 °C for 4 h under nitrogen atmosphere. ^{1}H NMR analysis of the crude product showed that dr > 95:5. Flash column chromatography on silica gel (petroleum ether : ethyl acetate = 2 : 1) afforded 180.5 mg (83%) **3l** as a yellow solid; Recrystallization from petroleum ether/ethyl acetate gave colorless crystals; mp: 92-93 °C; ^{1}H NMR (400 MHz, $CDCl_3$) δ 1.33 (d, *J* = 6.8 Hz, 6H), 1.34 (d, *J* = 6.8 Hz, 6H), 2.20-2.23 (m, BH_2CH, 1H), 2.42-2.58 (m, 2H), 3.24-3.32 (m, 4H), 3.48-3.53 (m, 2H), 3.60 (s, 3H), 4.64 (d, *J* = 5.2 Hz, 1H), 4.89-4.94 (m, 2H), 6.44 (d, *J* = 8.0 Hz, 1H), 6.62 (dd, *J* = 7.2, 7.2 Hz, 1H), 6.91 (s, 2H), 7.08 (dd, *J* = 7.6, 7.6 Hz, 1H), 7.19-7.30 (m, 5H), 7.39 (d, *J* = 7.2 Hz, 1H); ^{13}C NMR (100 MHz, $CDCl_3$) δ 22.7, 23.2, 31.9, 42.0, 49.1, 49.8, 51.3, 53.5, 73.6, 104.4, 115.4, 116.1, 126.5, 126.7, 127.0, 127.3, 128.0, 132.1, 144.5, 150.9, 172.7, 180.7; ^{11}B NMR (128.4 MHz, $CDCl_3$) δ -26.9 (t, *J* = 88.7 Hz, 1B); ESI-HRMS (*m/z*): $(M+H)^+$ Calcd for $C_{30}H_{41}{}^{11}BN_3O_4$, 518.3190; Found: 518.3193.

(1,3-Diisopropyl-1*H*-imidazol-3-ium-2-yl)((*S)-2-methoxy-2-oxo-1-((2*R**,3*R**)-1-phenethyl-2-phenylindolin-3-yl)ethyl)dihydroborate (3m)**

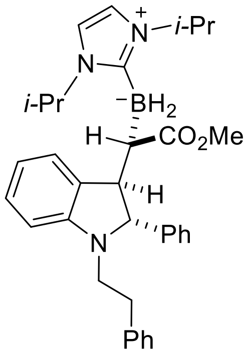

According to the general procedure E, the reaction of methyl 3-(2-(benzyl(phenethyl)amino)phenyl)propiolate (171.6 mg, 0.465 mmol), **2b** (93.2 mg, 0.561 mmol), ABVN (23.5 mg, 0.095 mmol) and phenyl 2-mercaptobenzoate (21.3 mg, 0.093 mmol) in toluene (4 mL) was stirred at 60 °C for 3 h under nitrogen atmosphere. ^{1}H NMR analysis of the crude product showed that dr = 95:5. Flash column chromatography on silica gel (petroleum ether : ethyl acetate = 2 : 1) afforded 223.8 mg (90%) **3m** as a yellow solid; Recrystallization from petroleum ether/ethyl acetate gave colorless crystals; mp: 150-153 °C; ^{1}H NMR (400 MHz, $CDCl_3$) δ 1.34 (d, *J* = 6.8 Hz, 6H), 1.34 (d, *J* = 6.8 Hz, 6H), 2.25-2.26 (m, BH_2CH, 1H), 2.68-2.75 (m, 1H), 2.79-2.86 (m, 1H), 3.06-3.14 (m, 1H), 3.26 (s, 3H), 3.37-3.45 (m, 1H), 3.57 (dd, *J* = 8.0, 6.0 Hz, 1H), 4.61 (d, *J* = 5.6 Hz, 1H), 4.91-4.98 (m, 2H), 6.42 (d, *J* = 7.6 Hz, 1H), 6.61 (dd, *J* = 7.2, 7.2 Hz, 1H), 6.91 (s, 2H), 7.07-7.29 (m, 11H), 7.44 (d, *J* = 7.2 Hz, 1H); ^{13}C NMR (100 MHz, $CDCl_3$) δ 22.9, 23.3, 33.3, 47.7, 49.2, 49.9, 53.5, 74.0, 104.1, 115.4, 115.7, 125.8, 126.77, 126.82, 127.1, 127.4, 128.1, 128.2, 128.7, 132.2, 140.3, 144.5, 151.4, 180.9; ^{11}B NMR (128.4 MHz, $CDCl_3$) δ -26.8 (t, *J* = 90.4 Hz, 1B); ESI-HRMS (*m/z*): $(M+H)^+$ Calcd for $C_{30}H_{43}{}^{11}BN_3O_4$, 536.3448; Found: 536.3471.

(1,3-Diisopropyl-1*H*-imidazol-3-ium-2-yl)((*S)-1-((2*R**,3*R**)-1,2-diphenylindolin-3-yl)-methoxy-2-oxoethyl)dihydroborate (3n)**

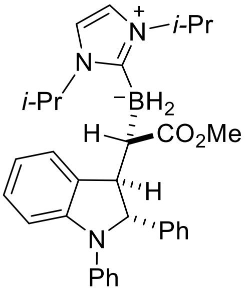

According to the general procedure E, the reaction of methyl 3-(2-(benzyl(phenyl)amino)phenyl)propiolate (151.0 mg, 0.442 mmol), **2b** (88.0 mg, 0.530 mmol), ABVN (22.4 mg, 0.090 mmol), and phenyl 2-mercaptobenzoate (20.6 mg, 0.089 mmol) in toluene (4 mL) was stirred at 60 °C for 4 h under nitrogen. ^{1}H NMR analysis of the crude product showed that dr = 86:14. Flash column chromatography on silica gel (petroleum ether : ethyl acetate = 6 : 1) afforded 181.8 mg (82%) **3n** as a grey solid; Recrystallization from petroleum ether/ethyl acetate gave colorless crystals; mp: 150-152 °C; NMR datas shown below were only given for the major one. ^{1}H NMR (400 MHz, $CDCl_3$) δ 1.30 (d, *J* = 6.8 Hz, 6H), 1.32 (d, *J* = 6.8 Hz, 6H), 2.20-2.23 (m, BH_2CH, 1H), 3.30 (s, 3H), 3.40 (dd, *J* = 9.6 Hz, 1H), 4.85-4.88 (m, 2H), 4.92 (s, 1H), 6.76 (ddd, *J* = 7.6, 7.2, 0.8 Hz, 1H), 6.80-6.84 (m, 1H), 6.89 (s, 2H), 7.12-7.26 (m, 8H), 7.35-7.39 (m, 3H), 7.46 (d, *J* = 7.2 Hz, 1H); ^{13}C NMR (100 MHz, $CDCl_3$) δ 22.8, 23.3, 49.1, 50.1, 54.7, 74.6, 109.1, 115.5, 116.2, 118.6, 119.5, 125.9, 126.4, 126.8, 128.3, 128.4, 128.8, 134.1, 144.29, 144.32, 146.0, 180.8; ^{11}B NMR (128.4 MHz, $CDCl_3$) δ -26.4 (t, *J* = 88.2 Hz, 1B); ESI-HRMS (*m/z*): $(M+H)^+$ Calcd for $C_{32}H_{39}{}^{11}BN_3O_2$, 508.3135; Found: 508.3133.

((*S)-1-((10b*R**,11*R**)-10b,11-dihydro-6*H*-isoindolo[2,1-*a*]indol-11-yl)-2-methoxy-2-oxoethyl)(1,3-diisopropyl-1*H*-imidazol-3-ium-2-yl)dihydroborate (3p)**

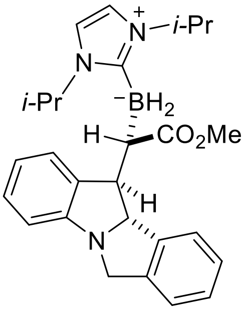

According to the general procedure E, the reaction of methyl 3-(2-(isoindolin-2-yl)phenyl)propiolate (116.2 mg, 0.419 mmol), **2b** (83.6 mg, 0.503 mmol), ABVN (21.4 mg, 0.086 mmol) and phenyl 2-mercaptobenzoate (21.0 mg, 0.091 mmol) in toluene (4 mL) was stirred at 60 °C for 4 h under nitrogen atmosphere. ^{1}H NMR analysis of the crude product showed that dr > 95:5. Flash column chromatography on silica gel (petroleum ether : ethyl acetate = 2 : 1) afforded 103.1 mg (56%) **3p** as a grey solid; Recrystallization from petroleum ether/ethyl acetate gave colorless crystals; mp: 85-87 °C; ^{1}H NMR (400 MHz, $CDCl_3$) δ 1.39 (d, *J* = 6.4 Hz, 6H), 1.39 (d, *J* = 6.4 Hz, 6H), 2.39-2.44 (m, BH_2CH, 1H), 3.28 (s, 3H), 3.97 (dd, *J* = 9.2, 2.0 Hz, 1H), 4.48 (d, *J* = 14.8 Hz, 1H), 4.61 (d, *J* = 14.8 Hz, 1H), 5.02-5.09 (m, 3H), 6.72-6.78 (m, 2H), 6.96 (s, 2H), 7.08 (dd, *J* = 8.0, 7.6 Hz, 1H), 7.14-7.24 (m, 3H), 7.44 (d, *J* = 7.6 Hz, 1H), 7.67 (d, *J* = 7.6 Hz, 1H); ^{13}C NMR (100 MHz, $CDCl_3$) δ 22.9, 23.4, 49.3, 49.9, 51.9, 59.0, 76.7, 111.3, 115.5, 119.3, 122.1, 123.5, 127.00, 127.03, 127.05, 127.2, 134.6, 139.6, 144.9, 155.1, 181.1; ^{11}B NMR (128.4 MHz, $CDCl_3$) δ -26.0 (t, *J* = 90.6 Hz, 1B); ESI-HRMS (*m/z*): $(M+H)^+$ Calcd for $C_{27}H_{35}{}^{11}BN_3O_2$, 444.2822; Found: 444.2822.

(1,3-Dimethyl-1*H*-imidazol-3-ium-2-yl)(2-methoxy-2-oxo-1-((2*R,3*R**)-2-phenyl-2,3-dihydrobenzofuran-3-yl)ethyl)dihydroborate (3q)**

Supplementary Table 4. Optimization of reaction conditions*[a]*

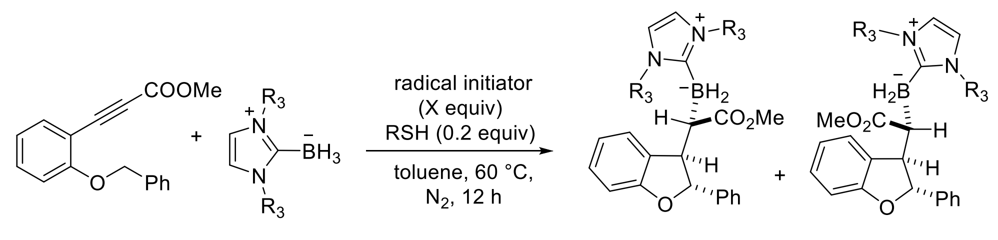

1q **2** (1.2 equiv) **3q** **4q**

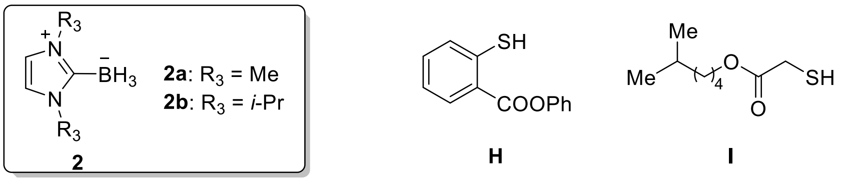

entry	2	initiator (X equiv)	RSH	**3q**+**4q** yield (%)[b]	**3q**:**4q** (dr)[c]
1	**2b**	ABVN (0.2 equiv)	**H**	15	6:1(**1q** : 37%)
2[d]	**2b**	TBHN (0.2 equiv)	**H**	9	7:1(**1q** : 67%)
3[e]	**2b**	ACCN (0.2 equiv)	**H**	24	6:1(**1q** : 10%)
4	**2b**	ABVN (0.2 equiv)	---	trace	---
5[d]	**2a**	TBHN (0.2 equiv)	**I**	36	5:1(**1q** : 10%)
6[d]	**2a**	TBHN (0.2 equiv)	**H**	18	5:1(**1q** : 20%)
7[e]	**2a**	ACCN (0.5 equiv)	---	83	6:1

[a] Reaction conditions: **1q** (0.2-0.3 mmol), **2** (1.2 equiv), initiator (X equiv), RSH (0.2 equiv), toluene (1-2 mL), 60 °C for 12 h. [b] NMR yield using tetrachloroethane as an internal standard. [c] dr was determined by ^{1}H NMR analysis of the crude reaction mixture.[d] The reaction was run at 50 °C. [e] The reaction was run at 95 °C.

General procedure F:

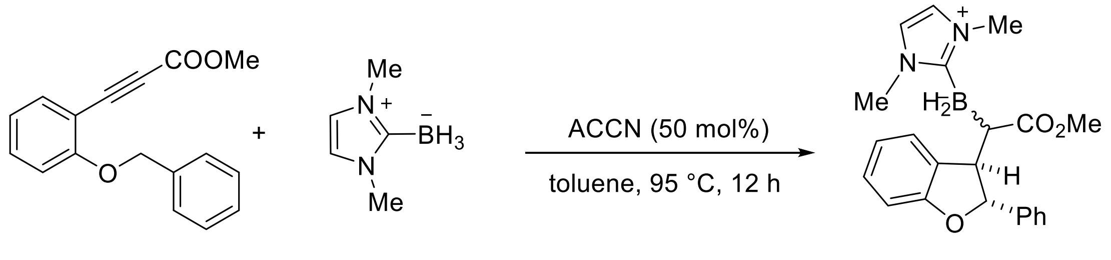

1q **2a (1.2 equiv)** **3q**

According to the general procedure F, the reaction of methyl 3-(2-(benzyloxy)phenyl)propiolate (77.7 mg, 0.292 mmol), **2a** (38.2 mg, 0.347 mmol), and ACCN (35.7 mg, 0.146 mmol) in toluene (3 mL) was stirred at 95 °C for 8 h under nitrogen atmosphere. ^{1}H NMR analysis of the crude product showed that dr = 86:14. Flash column chromatography on silica gel (petroleum ether : ethyl acetate = 2 : 1) afforded 91.2 mg (83%) **3q** as an inseparable mixture of diastereomers (dr = 4.3:1); ^{1}H NMR (400 MHz, $CDCl_3$) δ 2.19-2.24 (m, BH_2CH, 1Hx1), 2.28-2.33 (m, BH_2CH,

1Hx4.3), 3.33 (s, 3Hx4.3), 3.39 (s, 3Hx1), 3.65 (s, 6Hx4.3), 3.68 (s, 6Hx1), 3.74 (dd, *J* = 7.6, 4.0 Hz, 1Hx4.3), 3.83 (dd, *J* = 8.8, 3.6 Hz, 1Hx1), 5.68 (d, *J* = 4.0 Hz, 1Hx4.3), 6.04 (d, *J* = 3.6 Hz, 1Hx1), 6.75-6.69 (m, 1Hx1+2Hx1+2Hx4.3), 6.82 (ddd, *J* = 7.6, 7.6, 1.2 Hz, 1Hx4.3), 6.87 (d, *J* = 8.0 Hz, 1Hx4.3), 7.08-7.15 (m, 2Hx1+1Hx4.3), 7.20-7.24 (m, 1Hx1+1Hx4.3), 7.27-7.31 (m, 2Hx1+2Hx4.3), 7.44-7.51 (m, 3Hx1+3Hx4.3); ^{13}C NMR (100 MHz, $CDCl_3$) δ 35.79, 35.83, 50.21, 50.24, 52.9, 53.7, 87.3, 89.5, 108.7, 108.9, 119.6, 120.2, 120.3, 120.4, 124.9, 125.7, 125.8, 126.97, 126.99, 127.1, 127.6, 127.7, 128.1 (overlapped), 130.8, 132.4, 143.6, 143.9, 159.4, 159.9, 180.7, 180.8; ^{11}B NMR (128.4 MHz, $CDCl_3$) δ -26.6 (t, *J* = 90.5 Hz, 1B); ESI-HRMS (*m/z*): $(M+Na)^+$ Calcd for $C_{22}H_{25}{}^{11}BN_2NaO_3$, 399.1856; Found: 399.1859.

(1,3-Dimethyl-1*H*-imidazol-3-ium-2-yl)(2-methoxy-2-oxo-1-((2*R*,3*R*)-2-phenyl-2,3-dihydrobenzo[*b*]thiophen-3-yl)ethyl)dihydroborate (3r)

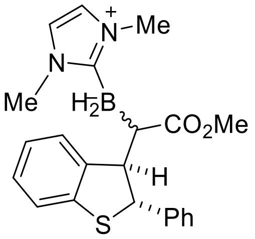

According to the general procedure F, the reaction of methyl 3-(2-(benzylthio)phenyl)propiolate (85.0 mg, 0.301 mmol), **2a** (39.7 mg, 0.361 mmol), and ACCN (36.8 mg, 0.151 mmol) in toluene (3 mL) was stirred at 95 °C for 8 h under nitrogen atmosphere. ^{1}H NMR analysis of the crude product showed that dr = 4:1. Flash column chromatography on silica gel (petroleum ether : ethyl acetate = 2 : 1) afforded 67.3 mg (57%) **3r** as an inseparable mixture of diastereomers (dr =67:33); ^{1}H NMR (400 MHz, $CDCl_3$) δ 2.42-2.48 (m, BH_2CH, 1Hx1+1Hx2.3), 3.23 (s, 3Hx1), 3.41 (s, 3Hx2.3), 3.58 (s, 6Hx2.3), 3.72 (s, 6Hx1), 3.93-3.95 (m, 1Hx1+1Hx2.3), 4.57 (d, *J* = 2.4 Hz, 1Hx2.3), 5.30 (s br, 1Hx1), 6.74 (s, 2Hx2.3), 6.79 (s, 2Hx1), 6.93 (ddd, *J* = 7.6, 7.6, 1.2 Hz, 1Hx1), 6.98 (ddd, *J* = 7.6, 7.2, 1.2 Hz, 1Hx2.3), 7.06-7.23 (m, 1Hx1+5Hx1+5Hx2.3), 7.32-7.36 (m, 2Hx1+2Hx2.3), 7.45 (d, *J* = 7.6 Hz, 1Hx2.3); ^{13}C NMR (100 MHz, $CDCl_3$) δ 35.7, 35.9, 50.0, 50.4, 55.5, 59.4, 59.7, 59.9, 120.3,

120.4, 121.4, 121.8, 123.0, 124.0, 125.1, 126.6, 126.69, 126.72, 126.8, 127.0, 127.1, 128.1, 128.21, 128.25, 140.2, 140.7, 142.0, 144.6, 144.7, 145.7, 180.3, 181.2; ^{11}B NMR (128.4 MHz, $CDCl_3$) δ -25.9 (t, *J* = 86.4 Hz, 1B), -26.6 (t, *J* = 87.4 Hz, 1B); ESI-HRMS (*m/z*): $(M+H)^+$ Calcd for $C_{22}H_{26}{}^{11}BN_2O_2S$, 393.1808; Found: 393.1815.

(1-((2*R,3*R**)-1-benzyl-2-phenylindolin-3-yl)-2,2,2-trifluoroethyl)(1,3-dimethyl-1*H*-imidazol-3-ium-2-yl)dihydroborate** (**3s**+**4s**)

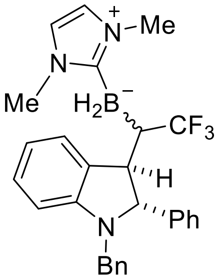

According to the general procedure F, the reaction of *N,N*-dibenzyl-2-(3,3,3-trifluoroprop-1-yn-1-yl)aniline (153.1 mg, 0.419 mmol), **2a** (55.3 mg, 0.503 mmol), and ACCN (51.2 mg, 0.210 mmol) in toluene (4 mL) was stirred at 95 °C for 8 h under nitrogen atmosphere. ^{1}H NMR analysis of the crude product showed that dr = 45:55. The two diastereomers could be separated by flash column chromatography (silica gel; petroleum ether : ethyl acetate = 1 : 1) afforded **3s** (58.9 mg, 0.124 mmol) and **4s** (70.6 mg, 0.149 mmol) in 65% combined yield.

Diastereomer 1:

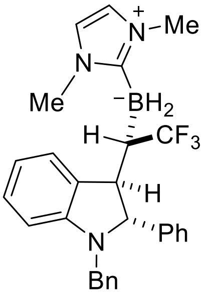

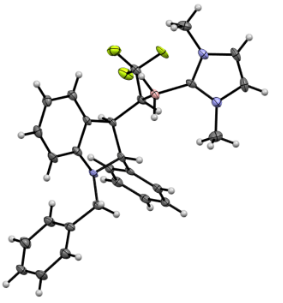

Recrystallization from petroleum ether/ethyl acetate gave colorless crystals; CCDC: 1994060, mp: 226-229 °C; ^{1}H NMR (500 MHz, DMSO-d_6) δ 1.37-1.45 (m, 1H), 3.45 (s, 6H), 3.50 (s, 6H), 3.53 (d, *J* = 10.0 Hz, 1H), 3.79 (d, *J* = 16.0 Hz, 1H), 4.24 (d, *J* = 10.0 Hz, 1H), 4.26 (d, *J* = 16.0 Hz, 1H), 6.35 (d, *J* = 8.0 Hz, 1H), 6.58 (dd, *J* = 7.5,

7.5 Hz, 1H), 6.91 (dd, J = 7.5, 7.5 Hz, 1H), 7.15-7.19 (m, 4H), 7.23-7.26 (m, 2H), 7.29 (s, 2H), 7.30-3.32 (m, 1H), 7.37-7.38 (m, 4H); ^{13}C NMR (125 MHz, DMSO-d_6) δ 35.1, 50.2, 50.5 (q, J = 3.6 Hz), 73.6, 106.7, 117.1, 121.3, 125.5, 126.7, 126.8, 127.5, 127.9, 128.1, 128.2, 128.6, 130.4, 138.0, 141.9, 151.7; ^{11}B NMR (128.4 MHz, $CDCl_3$) δ -30.5 (t, J = 90.7 Hz, 1B); ^{19}F NMR (376 MHz, $CDCl_3$): δ -60.7 (d, J = 14.6 Hz, 3F); ESI-HRMS (*m/z*): $(M+H)^+$ Calcd for $C_{28}H_{30}{}^{11}BF_3N_3$, 476.2485; Found: 476.2491.

Supplementary Table 5. Crystal data and structure refinement for **3s**

.

Empirical formula	$C_{28}H_{29}BF_3N_3$
Formula weight	475.35
Temperature/K	100.01(10)
Crystal system	monoclinic
Space group	$P2_1/c$
a/Å	10.3123(5)
b/Å	14.2293(10)
c/Å	16.9478(13)
α/°	90.006(6)
β/°	73.282(6)
γ/°	90.005(5)
Volume/Å^3	2381.8(3)
Z	4
ρ_{calc}g/cm^3	1.326
μ/mm^{-1}	0.094
F(000)	1000.0
Crystal size/mm^3	0.14 × 0.12 × 0.1
Radiation	MoKα (λ = 0.71073)
2Θ range for data collection/°	3.804 to 49.998
Index ranges	$-12 \le h \le 10$, $-16 \le k \le 16$, $-19 \le l \le 20$
Reflections collected	10758
Independent reflections	4196 [R_{int} = 0.0544, R_{sigma} = 0.0615]
Data/restraints/parameters	4196/0/326
Goodness-of-fit on F^2	1.042
Final R indexes [I>=2σ (I)]	R_1 = 0.0526, wR_2= 0.1305
Final R indexes [all data]	R_1 = 0.0688, wR_2 = 0.1460
Largest diff. peak/hole / e Å^{-3}	0.26/-0.31

Diastereomer 2:

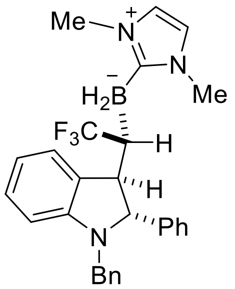

Pale yellow oil; ^{1}H NMR (400 MHz, $CDCl_3$) δ 1.70-1.79 (m, 1H), 3.45 (s, 6H), 3.76 (d, J = 6.4 Hz, 1H), 4.08 (d, J = 16.0 Hz, 1H), 4.32 (d, J = 16.0 Hz, 1H), 5.11 (d, J = 6.4 Hz, 1H), 6.24 (d, J = 7.6 Hz, 1H), 6.52 (dd, J = 7.6, 7.2 Hz, 1H), 6.68 (s, 2H), 6.91 (d, J = 7.2 Hz, 1H), 6.95 (dd, J = 8.0, 7.6 Hz, 1H), 7.13-7.36 (m, 10H); ^{13}C NMR (100 MHz, $CDCl_3$) δ 35.6, 50.8, 51.1 (q, J = 3.2 Hz), 71.4, 105.4, 116.2, 120.2, 123.2, 126.5, 126.9, 127.3, 127.6, 128.02, 128.04, 128.1, 132.1, 132.4 (q, J = 278.8 Hz), 139.0, 144.2, 152.2; ^{11}B NMR (128.4 MHz, $CDCl_3$) δ -30.4 (t, J = 88.5 Hz, 1B); ^{19}F NMR (376 MHz, $CDCl_3$): δ -61.4 (d, J = 13.2 Hz, 3F); ESI-HRMS (*m/z*): $(M+H)^+$ Calcd for $C_{28}H_{30}{}^{11}BF_3N_3$, 476.2485; Found: 476.2491.

2.3 ZnI_2-mediated Radical Borylative Cascades for Stereoselective Synthesis of Boron-Tethered Heterocycles

General procedure G:

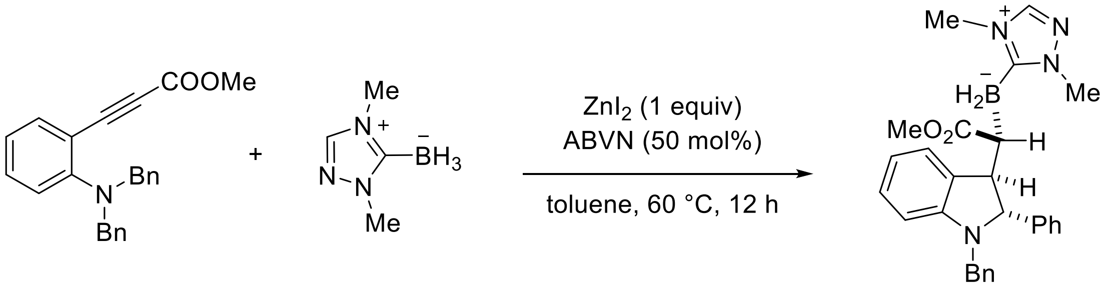

1a **2d (1.2 equiv)** **4aa**

A solution of **1a** (150.0 mg, 0.422 mmol), **2d** (56.0 mg, 0.505 mmol), ABVN (52.4 mg, 0. 211 mmol), and ZnI_2 (135.0 mg, 0.423 mmol) in toluene (2 mL) was stirred at 60 °C for 12 h under nitrogen atmosphere. The reaction mixture was quenched with NH_4Cl-NH_3/H_2O buffer solutions (pH = 7-8). The aqueous layer was extracted three times with dichloromethane. The combined extracts were dried over Na_2SO_4 and concentrated in *vacuo*. The resulting crude residues was subjected to ^{1}H NMR

analysis and the result showed that **3aa**:**4aa** = 6:94 (dr). The crude material was purified by flash column chromatography (silica gel; petroleum ether : ethyl acetate = 2 : 1) to give product **4aa** (149.0 mg) in 76% yield as a grey solid; Recrystallization from petroleum ether/ethyl acetate gave colorless crystals; mp: 120-121 °C.

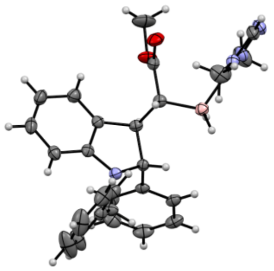

CCDC 1994061: ^{1}H NMR (400 MHz, $CDCl_3$) δ 2.14-2.17 (m, BH_2CH, 1H), 3.36 (s, 3H), 3.57 (s, 3H), 3.64 (dd, *J* = 9.2, 3.6 Hz, 1H), 3.81 (s, 3H), 4.00 (d, *J* = 16.0 Hz, 1H), 4.46 (d, *J* = 16.0 Hz, 1H), 4.94 (d, *J* = 3.6 Hz, 1H), 6.33 (d, *J* = 7.6 Hz, 1H), 6.52 (dd, *J* = 7.6, 7.2 Hz 1H), 6.98-7.02 (m, 2H), 7.18-7.30 (m, 10H), 7.79 (s, 1H); ^{13}C NMR (100 MHz, $CDCl_3$) δ 33.5, 38.0, 49.0, 50.2, 52.6, 70.6, 104.4, 116.3, 124.4, 126.6, 126.8, 127.3, 127.46, 127.50, 128.17, 128.20, 133.6, 139.3, 141.3, 144.1, 151.1, 180.5; ^{11}B NMR (128.4 MHz, $CDCl_3$) δ -27.1 (t, *J* = 88.2 Hz, 1B); ESI-HRMS (*m/z*): $(M+H)^+$ Calcd for $C_{28}H_{32}{}^{11}BN_4O_2$, 467.2618; Found: 467.2630.

Supplementary Table 6. Crystal data and structure refinement for **4aa**

Empirical formula	$C_{28}H_{31}BN_4O_2$
Formula weight	466.38
Temperature/K	150.00(10)
Crystal system	triclinic
Space group	P-1
a/Å	10.7610(14)
b/Å	11.3103(16)
c/Å	11.4757(12)
α/°	84.448(10)
β/°	79.344(10)
γ/°	68.223(13)
Volume/Å^3	1274.1(3)
Z	2
ρ_{calc}g/cm^3	1.216

μ/mm^{-1}	0.077
F(000)	496.0
Crystal size/mm^3	0.12 × 0.11 × 0.08
Radiation	MoKα (λ = 0.71073)
2Θ range for data collection/°	4.13 to 49.998
Index ranges	-12 ≤ h ≤ 12, -12 ≤ k ≤ 13, -13 ≤ l ≤ 11
Reflections collected	8317
Independent reflections	4492 [R_{int} = 0.0231, R_{sigma} = 0.0420]
Data/restraints/parameters	4492/0/327
Goodness-of-fit on F^2	1.043
Final R indexes [I>=2σ (I)]	R_1 = 0.0466, wR_2 = 0.1036
Final R indexes [all data]	R_1 = 0.0614, wR_2 = 0.1132
Largest diff. peak/hole / e Å^{-3}	0.18/-0.20

((*R)-1-((2*R**,3*R**)-1-benzyl-5-fluoro-2-phenylindolin-3-yl)-2-methoxy-2-oxoethyl)(1,4-dimethyl-4*H*-1,2,4-triazol-1-ium-5-yl)dihydroborate (4ha)**

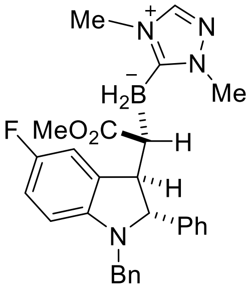

According to the general procedure G, the reaction of methyl 3-(2-(dibenzylamino)-5-fluorophenyl)propiolate (150.8 mg, 0.402 mmol), **2d** (53.5 mg, 0.482 mmol), ABVN (50.0 mg, 0. 201 mmol) and ZnI_2 (128.3 mg, 0.402 mmol) in toluene (2 mL) was stirred at 60 °C for 12 h under nitrogen atmosphere. ^{1}H NMR analysis of the crude product showed that dr = 7:93. Flash column chromatography on silica gel (petroleum ether : ethyl acetate = 2 : 1) afforded 138.4 mg (71%) **4ha** as a grey solid; Recrystallization from petroleum ether/ethyl acetate gave colorless crystals; mp: 149-151 °C; ^{1}H NMR (400 MHz, $CDCl_3$) δ 2.14-2.19 (m, BH_2CH, 1H), 3.37 (s, 3H), 3.59 (dd, *J* = 8.8, 3.6 Hz, 1H), 3.62 (s, 3H), 3.83 (s, 3H), 3.99 (d, *J* = 15.6 Hz, 1H), 4.38 (d, *J* = 15.6 Hz, 1H), 4.90 (d, *J* = 4.0 Hz, 1H), 6.17 (dd, *J* = 8.4, 4.4 Hz, 1H), 6.68 (ddd, *J* = 9.2, 8.8, 2.8 Hz, 1H), 6.82 (ddd, *J* = 8.8, 2.8, 0.8 Hz, 1H), 7.19-7.28 (m, 10H), 7.85 (s, 1H); ^{13}C NMR (100 MHz, $CDCl_3$) δ 33.5, 38.1, 49.8, 50.3, 52.7, 71.6,

104.2 (d, J = 8.8 Hz), 112.4 (d, J = 24.2 Hz), 112.8 (d, J = 22.9 Hz), 126.7, 126.9, 127.3, 127.5, 128.24, 128.27, 135.3 (d, J = 7.7 Hz), 139.2, 141.4, 143.8, 147.5, 155.8 (d, J = 230.6 Hz), 180.2; ^{11}B NMR (128.4 MHz, $CDCl_3$) δ -27.0 (t, J = 88.2 Hz, 1B); ^{19}F NMR (376 MHz, $CDCl_3$): δ -129.4-129.3 (m, 1F); ESI-HRMS (*m/z*): $(M+H)^+$ Calcd for $C_{28}H_{31}{}^{11}BFN_4O_2$, 485.2524; Found: 485.2526.

((*R)-1-((2*R**,3*R**)-1-benzyl-5-chloro-2-phenylindolin-3-yl)-2-methoxy-2-oxoethyl)(1,4-dimethyl-4*H*-1,2,4-triazol-1-ium-5-yl)dihydroborate (4ia)**

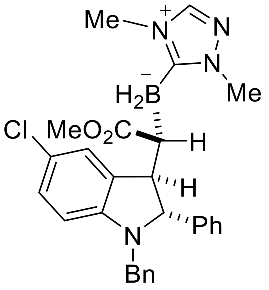

According to the general procedure G, the reaction of methyl 3-(5-chloro-2-(dibenzylamino)phenyl)propiolate (156.7 mg, 0.402 mmol), **2d** (53.5 mg, 0.482 mmol), ABVN (52.2 mg, 0. 210 mmol) and ZnI_2 (129.6 mg, 0.406 mmol) in toluene (2 mL) was stirred at 60 °C for 12 h under nitrogen atmosphere. ^{1}H NMR analysis of the crude product showed that dr = 7:93. Flash column chromatography on silica gel (petroleum ether : ethyl acetate = 2 : 1) afforded 143.0 mg (71%) **4ia** as a grey solid; Recrystallization from petroleum ether/ethyl acetate gave colorless crystals; mp: 149-150 °C; ^{1}H NMR (400 MHz, $CDCl_3$) δ 2.10-2.13 (m, BH_2CH, 1H), 3.38 (s, 3H), 3.57-3.60 (m, 4H), 3.82 (s, 3H), 4.00 (d, J = 16.0 Hz, 1H), 4.41 (d, J = 16.0 Hz, 1H), 4.96 (d, J = 3.6 Hz, 1H), 6.21 (d, J = 8.0 Hz, 1H), 6.94 (dd, J = 8.4, 2.0 Hz, 1H), 6.98 (d, J = 1.6 Hz, 1H), 7.21-7.28 (m, 10H), 7.84 (s, 1H); ^{13}C NMR (100 MHz, $CDCl_3$) δ 33.5, 38.0, 49.1, 50.3, 52.5, 70.9, 104.9, 120.5, 124.7, 126.8, 126.99, 127.05, 127.1, 127.4, 128.3 (overlapped), 135.6, 138.8, 141.4, 143.6, 149.8, 180.2; ^{11}B NMR (128.4 MHz, $CDCl_3$) δ -27.1 (t, J = 88.2 Hz, 1B); ESI-HRMS (*m/z*): $(M+H)^+$ Calcd for $C_{28}H_{31}{}^{11}BClN_4O_2$, 501.2229; Found: 501.2225.

(1,4-Dimethyl-4*H*-1,2,4-triazol-1-ium-5-yl)((*R)-2-methoxy-1-((2*R**,3*R**)-2-(naph**

thalen-2-yl)-1-(naphthalen-2-ylmethyl)indolin-3-yl)-2-oxoethyl)dihydroborate (4ta)

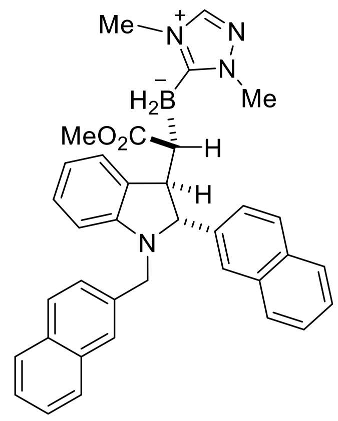

According to the general procedure G, the reaction of methyl 3-(2-(bis(naphthalen-2-ylmethyl)amino)phenyl)propiolate (190.7 mg, 0.419 mmol), **2d** (55.8 mg, 0.503 mmol), ABVN (52.0 mg, 0. 209 mmol) and ZnI_2 (136.0 mg, 0.426 mmol) in toluene (2 mL) was stirred at 60 °C for 12 h under nitrogen atmosphere. ^{1}H NMR analysis of the crude product showed that dr = 6:94. Flash column chromatography on silica gel (petroleum ether : ethyl acetate = 2 : 1) afforded 156.0 mg (66%) **4ta** as a grey solid; Recrystallization from petroleum ether/ethyl acetate gave colorless crystals; mp: 168-170 °C; ^{1}H NMR (400 MHz, $CDCl_3$) δ 2.15-2.22 (m, BH_2CH, 1H), 3.37 (s, 3H), 3.45 (s, 3H), 3.74 (s, 3H), 3.78-3.80 (m, 1H), 4.18 (d, *J* = 16.0 Hz, 1H), 4.70 (d, *J* = 16.0 Hz, 1H), 5.14 (d, *J* = 3.6 Hz, 1H), 6.49 (d, *J* = 8.0 Hz, 1H), 6.60 (dd, *J* = 7.6, 7.2 Hz, 1H), 7.07 (dd, *J* = 6.0, 5.6 Hz, 2H), 7.42-7.45 (m, 5H), 7.52 (d, *J* = 8.4 Hz, 1 H), 7.72-7.82 (m, 9H); ^{13}C NMR (100 MHz, $CDCl_3$) δ 33.3, 37.9, 49.2, 50.2, 52.5, 70.5, 104.6, 116.4, 124.5, 125.3, 125.4, 125.67, 125.75, 125.9, 126.1 (overlapped), 126.3, 127.5, 127.6 (overlapped), 127.7, 127.9 (overlapped), 128.1, 132.6, 132.8, 133.3, 133.4, 133.7, 136.8, 141.3, 141.6, 151.2, 180.5; ^{11}B NMR (128.4 MHz, $CDCl_3$) δ -27.1 (t, *J* = 88.2 Hz, 1B); ESI-HRMS (*m/z*): $(M+H)^+$ Calcd for $C_{36}H_{36}{}^{11}BN_4O_2$, 567.2931; Found: 567.2935.

((*R)-1-((2*R**,3*R**)-1-(4-chlorobenzyl)-2-(4-chlorophenyl)indolin-3-yl)-2-methoxy -2-oxoethyl)(1,4-dimethyl-4*H*-1,2,4-triazol-1-ium-5-yl)dihydroborate (4ua)**

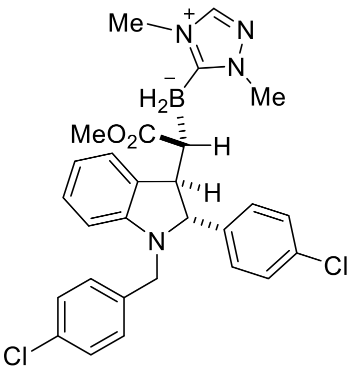

According to the general procedure G, the reaction of methyl 3-(2-(bis(4-chlorobenzyl)amino)phenyl)propiolate (168.9 mg, 0.398 mmol), **2d** (53.0 mg, 0.478 mmol), ABVN (49.4 mg, 0. 199 mmol) and ZnI_2 (130.0 mg, 0.407 mmol) in toluene (2 mL) was stirred at 60 °C for 12 h under nitrogen atmosphere. ^{1}H NMR analysis of the crude product showed that dr = 8:92. Flash column chromatography on silica gel (petroleum ether : ethyl acetate = 2 : 1) afforded 142.0 mg (67%) **4ua** as a grey solid; Recrystallization from petroleum ether/ethyl acetate gave colorless crystals; mp: 160-162 °C; ^{1}H NMR (400 MHz, $CDCl_3$) δ 2.09-2.14 (m, BH_2CH, 1H), 3.36 (s, 3H), 3.61 (s, 3H), 3.62-3.64 (m, 1H), 3.83 (s, 3H), 3.94 (d, J = 16.0 Hz, 1H), 4.41 (d, J = 16.0 Hz, 1H), 4.88 (d, J = 4.4 Hz, 1H), 6.33 (d, J = 7.6 Hz, 1H), 6.57 (ddd, J = 7.6, 7.6, 1.2 Hz, 1H), 6.99-7.04 (m, 2H), 7.18-7.26 (m, 8H), 7.85 (s, 1H); ^{13}C NMR (100 MHz, $CDCl_3$) δ 33.5, 38.0, 48.7, 50.3, 52.6, 69.9, 104.7, 116.8, 124.3, 127.6, 128.3, 128.4, 128.8, 129.0, 132.39, 132.43, 133.4, 137.5, 141.3, 142.7, 150.8, 180.5; ^{11}B NMR (128.4 MHz, $CDCl_3$) δ -27.3 (t, J = 88.2 Hz, 1B); ESI-HRMS (*m/z*): $(M+H)^+$ Calcd for $C_{28}H_{30}{}^{11}BCl_2N_4O_2$, 535.1839; Found: 535.1832.

(1,4-Dimethyl-4*H*-1,2,4-triazol-1-ium-5-yl)((*R)-2-methoxy-1-((2*R**,3*R**)-1-(3-methoxybenzyl)-2-(3-methoxyphenyl)indolin-3-yl)-2-oxoethyl)dihydroborate (4va)**

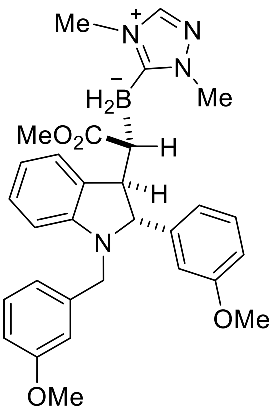

According to the general procedure G, the reaction of methyl 3-(2-(bis(3-methoxybenzyl)amino)phenyl)propiolate (156.9 mg, 0.378 mmol), **2d** (50.3 mg, 0.454 mmol), ABVN (47.0 mg, 0. 189 mmol) and ZnI_2 (120.5 mg, 0.378 mmol) in toluene (2 mL) was stirred at 60 °C for 12 h under nitrogen atmosphere. ^{1}H NMR analysis of the crude product showed that dr = 8:92. Flash column chromatography on silica gel (petroleum ether : ethyl acetate = 2 : 1) afforded 145.0 mg (73%) **4va** as a grey solid; Recrystallization from petroleum ether/ethyl acetate gave colorless crystals; mp: 43-45 °C; ^{1}H NMR (400 MHz, $CDCl_3$) δ 2.16-2.21 (m, BH_2CH, 1H), 3.37 (s, 3H), 3.62 (s, 3H), 3.64 (dd, *J* = 9.2, 3.6 Hz, 1H), 3.73 (s, 3H), 3.74 (s, 3H), 3.84 (s, 3H), 4.02 (d, *J* = 16.0 Hz, 1H), 4.44 (d, *J* = 16.0 Hz, 1H), 4.94 (d, *J* = 4.0 Hz, 1H), 6.33 (d, *J* = 7.6 Hz, 1H), 6.52 (ddd, *J* = 7.6, 7.2, 1.2 Hz, 1H), 6.74-6.77 (m, 2H), 6.86-6.89 (m, 4H), 6.98-7.02 (m, 2H), 7.16-7.20 (m, 2H), 7.83 (s, 1H); ^{13}C NMR (100 MHz, $CDCl_3$) δ 33.5, 38.0, 49.2, 50.2, 52.6, 55.06, 55.09, 70.7, 104.5, 112.1, 112.2, 112.86, 112.90, 116.3, 119.7, 119.8, 124.3, 127.5, 129.1, 129.2, 133.7, 141.2, 141.3, 145.9, 151.1, 159.5, 159.7, 180.5; ^{11}B NMR (128.4 MHz, $CDCl_3$) δ -27.1 (t, *J* = 88.2 Hz, 1B); ESI-HRMS (*m/z*): $(M+H)^+$ Calcd for $C_{30}H_{36}{}^{11}BN_4O_4$, 527.2830; Found: 527.2828.

(1,4-Dimethyl-4*H*-1,2,4-triazol-1-ium-5-yl)((*R)-2-methoxy-1-((2*R**,3*R**)-1-(4-methylbenzyl)-2-(p-tolyl)indolin-3-yl)-2-oxoethyl)dihydroborate (4wa)**

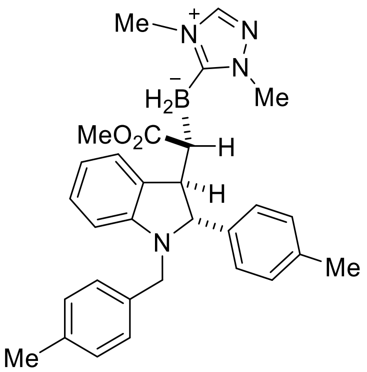

According to the general procedure G, the reaction of methyl 3-(2-(bis(4-methylbenzyl)amino)phenyl)propiolate (141.2 mg, 0.368 mmol), **2d** (49.0 mg, 0.442 mmol), ABVN (46.3 mg, 0. 186 mmol) and ZnI_2 (118.0 mg, 0.370 mmol) in toluene (2 mL) was stirred at 60 °C for 12 h under nitrogen atmosphere. ^{1}H NMR analysis of the crude product showed that dr = 7:93. Flash column chromatography on silica gel (petroleum ether : ethyl acetate = 2 : 1) afforded 127.0 mg (70%) **4wa** as a grey solid; Recrystallization from petroleum ether/ethyl acetate gave colorless crystals; mp: 119-120 °C; ^{1}H NMR (400 MHz, $CDCl_3$) δ 2.14-2.21 (m, BH_2CH, 1H), 2.32 (s, 6H), 3.38 (s, 3H), 3.60 (s, 3H), 3.63 (dd, *J* = 8.8, 3.6 Hz, 1H), 3.82 (s, 3H), 3.97 (d, *J* = 15.6 Hz, 1H), 4.41 (d, *J* = 15.6 Hz, 1H), 4.91 (d, *J* = 3.6 Hz, 1H), 6.32 (d, *J* = 8.0 Hz, 1H), 6.52 (ddd, *J* = 7.6, 7.2, 0.8 Hz, 1H), 6.98-7.02 (m, 2H), 7.07-7.08 (m, 4H), 7.16-7.20 (m, 4H), 7.82 (s, 1H); ^{13}C NMR (100 MHz, $CDCl_3$) δ 21.0, 21.1, 33.5, 38.0, 48.6, 50.2, 52.6, 70.4, 104.4, 116.1, 124.3, 127.3, 127.4 (overlapped), 128.85, 128.87, 133.7, 136.0, 136.2, 136.3, 141.1, 141.3, 151.2, 180.6; ^{11}B NMR (128.4 MHz, $CDCl_3$) δ -27.1 (t, *J* = 88.2 Hz, 1B); ESI-HRMS (*m/z*): $(M+H)^+$ Calcd for $C_{30}H_{36}{}^{11}BN_4O_2$, 495.2931; Found: 495.2937.

(1,4-Dimethyl-4*H*-1,2,4-triazol-1-ium-5-yl)((*R)-2-methoxy-1-((2*R**,3*R**)-1-(3-methylbenzyl)-2-(m-tolyl)indolin-3-yl)-2-oxoethyl)dihydroborate (4xa)**

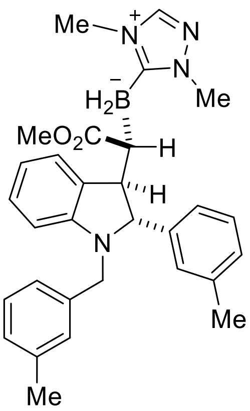

According to the general procedure G, the reaction of methyl 3-(2-(bis(3-methylbenzyl)amino)phenyl)propiolate (151.7 mg, 0.386 mmol), **2d** (53.0 mg, 0.478 mmol), ABVN (49.2 mg, 0. 198 mmol) and ZnI_2 (130.0 mg, 0.407 mmol) in toluene (2 mL) was stirred at 60 °C for 12 h under nitrogen atmosphere. ^{1}H NMR analysis of the crude product showed that dr = 7:93. Flash column chromatography on silica gel (petroleum ether : ethyl acetate = 2 : 1) afforded 133.0 mg (70%) **4xa** as a grey solid; Recrystallization from petroleum ether/ethyl acetate gave colorless crystals; mp: 41-42 °C; ^{1}H NMR (400 MHz, $CDCl_3$) δ 2.14-2.21 (m, BH_2CH, 1H), 2.29 (s, 3H), 2.30 (s, 3H), 3.37 (s, 3H), 3.61 (s, 3H), 3.64 (dd, *J* = 9.2, 3.6 Hz, 1H), 3.82 (s, 3H), 3.98 (d, *J* = 16.0 Hz, 1H), 4.40 (d, *J* = 16.0 Hz, 1H), 4.92 (d, *J* = 3.6 Hz, 1H), 6.32 (d, *J* = 7.6 Hz, 1H), 6.51 (ddd, *J* = 7.6, 7.2, 1.2 Hz, 1H), 6.99-7.16 (m, 10H), 7.83 (s, 1H); ^{13}C NMR (100 MHz, $CDCl_3$) δ 21.4, 21.5, 33.5, 38.0, 49.1, 50.2, 52.6, 70.7, 104.4, 116.1, 124.3, 124.4, 124.5, 127.3, 127.46, 127.55, 128.0, 128.07, 128.09, 128.3, 133.7, 137.66, 137.72, 139.4, 141.3, 144.2, 151.2, 180.6; ^{11}B NMR (128.4 MHz, $CDCl_3$) δ -27.1 (t, *J* = 88.2 Hz, 1B); ESI-HRMS (*m/z*): $(M+H)^+$ Calcd for $C_{30}H_{36}{}^{11}BN_4O_2$, 495.2931; Found: 495.2931.

(1,4-Dimethyl-4*H*-1,2,4-triazol-1-ium-5-yl)(2-methoxy-1-((2*R,3*R**)-1-methyl-2-phenylindolin-3-yl)-2-oxoethyl)dihydroborate (3ba+4ba)**

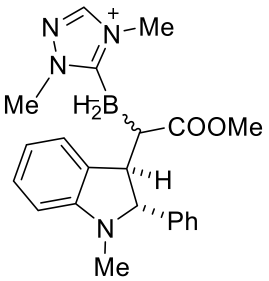

According to the general procedure G, the reaction of methyl 3-(2-(benzyl(methyl)amino)phenyl)propiolate (114.6 mg, 0.410 mmol), **2d** (54.2 mg, 0.489 mmol), ABVN (51.2 mg, 0.206 mmol) and ZnI_2 (130.7 mg, 0.410 mmol) in toluene (2 mL) was stirred at 60 °C for 12 h under nitrogen atmosphere. ^{1}H NMR analysis of the crude product showed that dr = 67:33. Flash column chromatography on silica gel (petroleum ether : ethyl acetate = 2 : 1) afforded 70.5 mg in 44% yield as an inseparable mixture of diastereomers (dr = 1.9:1); ^{1}H NMR (400 MHz, $CDCl_3$) δ 2.19-2.27 (m, BH_2CH, 1Hx1+1Hx1.9), 2.61 (s, 3Hx1.9), 2.65 (s, 3Hx1), 3.34 (s, 3Hx1.9), 3.78 (s, 3Hx1), 3.60-3.69 (1Hx1+3Hx1+1Hx1.9+3Hx1.9), 3.85 (s, 3Hx1.9), 3.86 (s, 3Hx1), 4.36 (d, J = 8.0 Hz, 1Hx1.9), 4.71 (d, J = 5.2 Hz, 1Hx1), 6.34 (d, J = 7.6 Hz, 1Hx1), 6.43 (d, J = 7.6 Hz, 1Hx1.9), 6.53 (dd, J = 7.2, 7.2 Hz, 1Hx1), 6.59 (dd, J = 7.6, 7.2 Hz, 1Hx1.9), 6.97 (d, J = 7.2 Hz, 1Hx1), 7.04-7.10 (m, 1Hx1+1x1.9), 7.22-7.37 (m, 5Hx1+6Hx1.9), 7.81-7.82 (m, 1Hx1+1Hx1.9); ^{13}C NMR (100 MHz, $CDCl_3$) δ 32.5, 33.5, 33.59, 33.64, 38.07, 38.11, 50.3, 50.4, 52.3, 52.7, 73.5, 76.6, 104.5, 105.6, 116.2, 116.7, 123.8, 126.1, 126.8, 127.1, 127.2, 127.5, 127.6, 127.8, 128.0, 128.2, 132.1, 133.8, 141.3 (overlapped), 143.5, 144.1, 152.1, 152.9, 180.8, 181.2; ^{11}B NMR (160.5 MHz, $CDCl_3$) δ -27.6 (t, J = 89.6 Hz, 1B), -27.4 (t, J = 84.6 Hz, 1B); ESI-HRMS (m/z): $(M+H)^+$ Calcd for $C_{22}H_{28}{}^{11}BN_4O_2$, 391.2305; Found: 391.2307.

(1,4-Dimethyl-4*H*-1,2,4-triazol-1-ium-5-yl)((*R)-2-methoxy-1-((2*R**,3*R**)-1-(3-methoxy-3-oxopropyl)-2-phenylindolin-3-yl)-2-oxoethyl)dihydroborate (3la+4la)**

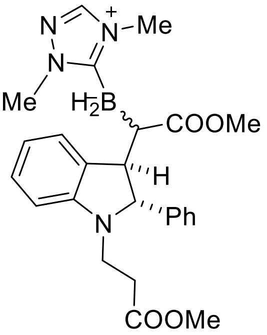

According to the general procedure G, the reaction of methyl 3-(2-(benzyl(3-methoxy-3-oxopropyl)amino)phenyl)propiolate (123.8 mg, 0.352 mmol), **2d** (47.5 mg, 0.428 mmol), ABVN (43.6 mg, 0.176 mmol) and ZnI_2 (114.0 mg, 0.357 mmol) in toluene (2 mL) was stirred at 60 °C for 12 h under nitrogen atmosphere. ^{1}H NMR analysis of the crude product showed that dr = 23:77. Flash column chromatography on silica gel (petroleum ether : ethyl acetate = 2 : 1) afforded 72.0 mg in 44% yield as an inseparable mixture of diastereomers (1:3.2); ^{1}H NMR (400 MHz, $CDCl_3$) δ 2.14-2.15 (m, BH_2CH, 1Hx3.2), 2.23-2.26 (m, BH_2CH, 1Hx1), 2.45-2.56 (m, 2Hx1+2Hx3.2), 3.26-3.39 (m, 1Hx1+3Hx1+1Hx3.2+3Hx3.2), 3.52-3.63 (m, 1Hx1+1Hx1+3Hx1+3Hx1+1Hx3.2+1Hx3.2+3Hx3.2), 3.67 (s, 3Hx3.2), 3.86-3.87 (m, 3Hx1+3Hx3.2), 4.61 (d, J = 6.4 Hz, 1Hx1), 4.94 (d, J = 4.0 Hz, 1Hx3.2), 6.41 (d, J = 8.0 Hz, 1Hx3.2), 6.45 (d, J = 8.0 Hz, 1Hx1), 6.52 (dd, J = 7.2, 7.2 Hz, 1Hx3.2), 6.58 (dd, J = 7.6, 7.2 Hz, 1Hx1), 6.95 (d, J = 7.6 Hz, 1Hx3.2), 7.04-7.09 (m, 2Hx1+1Hx3.2), 7.20-7.32 (m, 5Hx1+5Hx3.2), 7.83-7.84 (m, 1Hx1+1Hx3.2); ^{13}C NMR (100 MHz, $CDCl_3$) δ 31.7, 32.5, 33.5, 33.6, 38.1 (overlapped), 41.5, 42.1, 50.2, 50.3, 51.5 (overlapped), 52.7, 53.2, 70.5, 73.5, 104.2, 104.9, 116.3, 116.5, 124.2, 126.2, 126.8, 127.1, 127.27, 127.31, 127.5, 127.6, 128.18, 128.24, 131.9, 133.5, 141.3 (overlapped), 144.1, 144.7, 150.7, 151.0, 172.7, 172.8, 180.7, 180.9; ^{11}B NMR (128.4 MHz, $CDCl_3$) δ -27.4 (t, J = 90.6 Hz, 1B); ESI-HRMS (m/z): $(M+H)^+$ Calcd for $C_{25}H_{32}{}^{11}BN_4O_4$, 463.2517; Found: 463.2521.

(1,4-Dimethyl-4*H*-1,2,4-triazol-1-ium-5-yl)(1-((2*R,3*R**)-1,2-diphenylindolin-3-yl)-2-methoxy-2-oxoethyl)dihydroborate (3na+4na)**

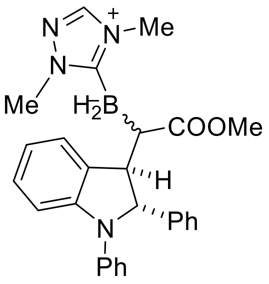

According to the general procedure G, the reaction of methyl 3-(2-(benzyl(phenyl)amino)phenyl)propiolate (103.5 mg, 0.303 mmol), **2d** (40.1 mg, 0.361 mmol), ABVN (37.6 mg, 0.151 mmol) and ZnI_2 (97.0 mg, 0.304 mmol) in toluene (1.5 mL) was stirred at 60 °C for 12 h under nitrogen atmosphere. ^{1}H NMR analysis of the crude product showed that dr = 72:28. Flash column chromatography on silica gel (petroleum ether : ethyl acetate = 2 : 1) afforded 31.5 mg in 23% yield as an inseparable mixture of diastereomers (1:1.7); ^{1}H NMR (400 MHz, $CDCl_3$) δ 2.04-2.07 (m, BH_2CH, 1Hx1), 2.23-2.27 (m, BH_2CH, 1Hx1.7), 3.33 (s, 3Hx1.7), 3.40-3.46 (m, 1Hx1+3Hx1+1Hx1.7), 3.54 (s, 3Hx1), 3.61 (s, 3Hx1.7), 3.80 (s, 3Hx1), 3.86 (s, 3Hx1.7), 4.95 (d, J = 1.2 Hz, 1Hx1.7), 5.49 (s br, 1Hx1), 6.68-6.76 (m, 1Hx1+1Hx1.7), 6.82-6.85 (1Hx1+1Hx1.7), 7.09-7.44 (m, 12Hx1+12Hx1.7), 7.77 (s, 1Hx1), 7.80 (s, 1Hx1.7); ^{13}C NMR (100 MHz, $CDCl_3$) δ 33.5, 33.6, 38.06, 38.12, 50.3, 50.4, 53.6, 54.3, 71.3, 74.3, 109.3, 109.9, 116.3, 116.4, 118.7, 119.3, 119.80, 119.83, 125.5, 125.9, 126.5,125.89, 125.94, 126.5, 126.6, 127.0, 127.1, 128.0, 128.4, 128.9, 133.7, 135.9, 141.3 (overlapped), 144.2, 144.3, 144.4, 144.7, 145.9, 146.1, 180.5, 180.6; ^{11}B NMR (128.4 MHz, $CDCl_3$) δ -27.6—27.2 (m, 1Bx1+1Bx1.9); ESI-HRMS (*m/z*): $(M+H)^+$ Calcd for $C_{27}H_{30}{}^{11}BN_4O_2$, 453.2462; Found: 453.2466.

(1-((2*R,3*R**)-1-benzyl-2-phenylindolin-3-yl)-2,2,2-trifluoroethyl)(1,4-dimethyl-4*H*-1,2,4-triazol-1-ium-5-yl)dihydroborate (3sa+4sa)**

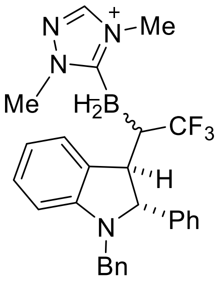

According to the general procedure G, the reaction of *N*,*N*-dibenzyl-2-(3,3,3-trifluoroprop-1-yn-1-yl)aniline (153.9 mg, 0.421 mmol), **2d** (56.1 mg, 0.506 mmol), ABVN (52.8 mg, 0.213 mmol) and ZnI_2 (138.0 mg, 0.432 mmol) in toluene (2 mL) was stirred at 60 °C for 12 h under nitrogen atmosphere. ^{1}H NMR analysis of the crude product showed that dr = 45:55. The two diastereomers could be separated by flash column chromatography (silica gel; petroleum ether : ethyl acetate = 1 : 1) afforded 128.3 mg (64%). afforded **3sa+4sa** (**diastereomer 1**, 58.3 mg, 0.122 mmol; **diastereomer 2**, 70.0 mg, 0.147 mmol) in 64% combined yield.

Diastereomer 1:

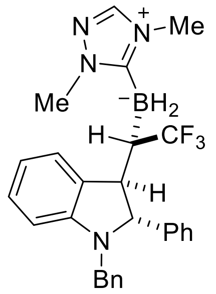

White solid, mp: 223-225 °C; ^{1}H NMR (500 MHz, DMSO-*d*$_6$) δ 1.44-1.52 (m, 1H), 3.53-3.56 (m, 4H), 3.73 (s, 3H), 3.82 (d, *J* = 16.0 Hz, 1H), 4.34 (d, *J* = 16.0 Hz, 1H), 4.40 (d, *J* = 10.0 Hz, 1H), 6.39 (d, *J* = 8.0 Hz, 1H), 6.58 (dd, *J* = 8.0, 7.5 Hz, 1H), 6.94 (dd, *J* = 8.0, 7.5 Hz, 1H), 7.13 (d, *J* = 7.5 Hz, 1H), 7.18-7.27 (m, 5H), 7.32 (dd, *J* = 7.0, 7.0 Hz, 1H), 7.37-7.43 (m, 4H), 8.70 (s, 1H); ^{13}C NMR (125 MHz, DMSO-*d*$_6$) δ 33.2, 37.5, 50.0, 50.4 (q, *J* = 4.9 Hz), 73.4, 106.8, 117.1, 126.8, 126.9, 127.6, 127.9, 128.1, 128.2 (overlapped), 128.7, 130.0, 137.9, 141.9, 143.6, 151.7; ^{11}B NMR (160.5 MHz, DMSO-*d*$_6$) δ -30.6 (s br, 1B); ^{19}F NMR (470 MHz, DMSO-*d*$_6$): δ -60.9 (d, *J* = 14.1 Hz, 3F); ESI-HRMS (*m/z*): ESI-HRMS (*m/z*): $(M+H)^+$ Calcd for $C_{27}H_{29}{}^{11}BF_3N_4$, 477.2437; Found: 477.2441.

Diastereomer 2:

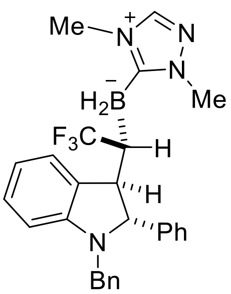

Yellow oil; ^{1}H NMR (400 MHz, $CDCl_3$) δ 1.75-1.78 (m, 1H), 3.45 (s, 3H), 3.74 (s, 3H), 3.78 (d, J = 5.6 Hz, 1H), 4.06 (d, J = 15.6 Hz, 1H), 4.32 (d, J = 15.6 Hz, 1H), 5.12 (d, J = 6.0 Hz, 1H), 6.25 (d, J = 7.6 Hz, 1H), 6.50 (dd, J = 7.2, 7.2 Hz, 1H), 6.84 (d, J = 7.2 Hz, 1H), 6.97 (dd, J = 7.6, 7.6 Hz, 1H), 7.12-7.35 (m, 10H), 7.73 (s, 1H); ^{13}C NMR (125 MHz, $CDCl_3$) δ 33.3, 37.9, 50.4, 50.7 (q, J = 3.2 Hz), 70.8, 105.3, 116.0, 122.9, 126.6, 127.1, 127.5, 127.6, 127.9, 128.1 (overlapped), 131.8, 132.0 (q, J = 278.4 Hz), 138.8, 141.3, 143.9, 152.1; ^{11}B NMR (160.5 MHz, $CDCl_3$) δ -30.8 (t, J = 88.5 Hz, 1B); ^{19}F NMR (376 MHz, $CDCl_3$): δ -61.9 (d, J = 12.8 Hz, 3F); ESI-HRMS (*m/z*): $(M+H)^+$ Calcd for $C_{27}H_{29}{}^{11}BF_3N_4$, 477.2437; Found: 477.2447.

(1-((*2R,*3R**)-1-benzyl-2-phenyl-2,3-dihydro-1*H*-pyrrolo[2,3-*b*]pyridin-3-yl)-2-methoxy-2-oxoethyl)(1,4-dimethyl-4*H*-1,2,4-triazol-1-ium-5-yl)dihydroborate (3ja+4ja)**

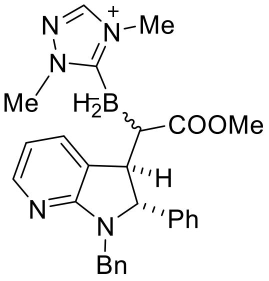

According to the general procedure G, the reaction of methyl 3-(2-(dibenzylamino)pyridin-3-yl)propiolate (143.5 mg, 0.403 mmol), **2d** (53.4 mg, 0.481 mmol), ABVN (49.9 mg, 0.199 mmol) and ZnI_2 (129.0 mg, 0.404 mmol) in toluene (2 mL) was stirred at 60 °C for 12 h under nitrogen atmosphere. ^{1}H NMR analysis of the crude product showed that dr = 62:38. Flash column chromatography on silica gel (petroleum ether : ethyl acetate = 1 : 3) afforded 72.0 mg in 47% yield as

an inseparable mixture of diastereomers (5.4:1); ^{1}H NMR (400 MHz, $CDCl_3$) δ 2.02-2.07 (m, BH_2CH, 1Hx1+1Hx5.4), 3.33 (s, 3Hx5.4), 3.36 (s, 3Hx1), 3.49-3.53 (m, 1Hx1+3Hx1+3Hx5.4), 3.57 (dd, J = 6.0, 6.0 Hz, 1Hx5.4), 3.75-3.80 (m, 1Hx1+3Hx1+1Hx5.4+3Hx5.4), 4.41 (d, J = 6.0 Hz, 1Hx5.4), 4.84 (d, J = 2.4 Hz, 1Hx1), 5.20 (d, J = 15.2 Hz, 1Hx5.4), 5.29 (d, J = 15.2 Hz, 1Hx1), 6.38 (dd, J = 6.8, 5.2 Hz, 1Hx1), 6.46 (dd, J = 7.2, 5.6 Hz, 1Hx5.4), 7.16-7.33 (m, 11Hx1H+10Hx5.4), 7.52 (ddd, J = 7.2, 2.8, 1.6 Hz, 1Hx5.4), 7.82 (s, 1Hx5.4) 7.84 (s, 1Hx1), 7.90 (d, J = 5.2 Hz, 1Hx1), 7.93 (d, J = 5.2 Hz, 1Hx5.4); ^{13}C NMR (100 MHz, $CDCl_3$) δ 33.5, 38.0, 45.7, 50.5, 50.7, 69.7, 111.9, 125.3, 126.7, 126.8, 127.1, 127.41, 127.44, 128.1, 128.21, 128.25, 128.45, 128.52, 133.4, 138.0, 141.3, 142.6, 142.9, 145.6, 162.4, 180.8, 183.8; ^{11}B NMR (160.5 MHz, $CDCl_3$) δ -27.8 (t, J = 91.0 Hz, 1B); ESI-HRMS (*m/z*): $(M+H)^+$ Calcd for $C_{27}H_{31}{}^{11}BN_5O_2$, 468.2571; Found: 468.2576.

2.4 Synthetic applications

2.4.1 Synthesis of alcohol 5a and 5aa

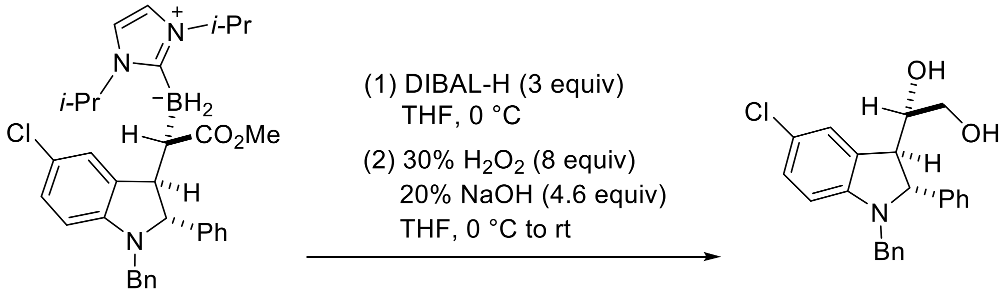

3i **5a**

To a solution of **3i** (278.0 mg, 0.500 mmol) in anhydrous THF (5 ml) was slowly added DIBAL-H (0.95 mL, 1.500 mmol) at 0 °C. The reaction mixture was stirred at this temperature for 30 min. The reaction was quenched with potassium sodium tartrate salts solutions followed by filtration through celite and washed with ethyl acetate (50 ml). The solvent was evaporated and the residue was dissolved in MeOH/CH_3CN (1:1, 10 mL), then H_2O_2 (30% aq, 0.44 ml) and NaOH (20% aq, 0.44 ml) was added at room temperature. The mixture was stirred at 40 °C overnight. The reaction was then quenched with saturated NH_4Cl (aq). The reaction mixture was extracted three times with dichloromethane. The combined extracts were dried over

Na_2SO_4 and concentrated in *vacuo*. The residue was purified by flash column chromatography (silica gel; petroleum ether : ethyl acetate = 2 : 1) to give **5a** (136.8 mg, 0.360 mmol) in 72% yield as a white solid, mp: 128-130 °C; ^{1}H NMR (400 MHz, $CDCl_3$) δ 1.78 (s br, 1H), 2.31 (s br, 1H), 3.41 (dd, *J* = 6.4, 6.0 Hz, 1H), 3.55-3.65 (m, 2H), 3.89-3.93 (m, 2H), 4.36 (d, *J* = 15.6 Hz, 1H), 4.51 (d, *J* = 6.0 Hz, 1H), 6.32 (d, *J* = 8.0 Hz, 1H), 7.05 (dd, *J* = 8.4, 2.0 Hz, 1H), 7.14-7.19 (m, 3H), 7.23-7.33 (m, 8H); ^{13}C NMR (100 MHz, $CDCl_3$) δ 49.6, 53.3, 64.3, 70.1, 74.0, 106.9, 121.8, 125.7, 127.2, 127.3, 127.4, 128.1, 128.4, 128.6, 128.8, 128.9, 137.5, 141.5, 150.6; ESI-HRMS (*m/z*): $(M+H)^+$ Calcd for $C_{23}H_{23}ClNO_2$, 380.1417; Found: 380.1412.

(*R)-1-((2*R**,3*R**)-1-benzyl-5-chloro-2-phenylindolin-3-yl)ethane-1,2-diol (5aa)**

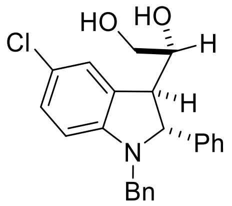

According to the same procedure as shown above , the reaction of **4ia** (139.1 mg, 0.278 mmol) afforded **5aa** (71.8 mg, 0.189 mmol) in 68% yield as a white solid, mp: 139-140 °C; ^{1}H NMR (400 MHz, $CDCl_3$) δ 1.95 (s br, 1H), 2.37 (s br, 1H), 3.41 (dd, *J* = 5.6, 5.6 Hz, 1H), 3.52-3.63 (m, 2H), 3.94 (d, *J* = 16.0 Hz, 1H), 4.01-4.05 (m, 1H), 4.34 (d, *J* = 16.0 Hz, 1H), 4.74 (d, *J* = 6.4 Hz, 1H), 6.27 (d, *J* = 8.4 Hz, 1H), 7.02 (ddd, *J* = 8.4, 2.0, 0.4 Hz, 1H), 7.07 (dd, *J* = 2.0, 0.8 Hz, 1H), 7.15-7.17 (m, 2H), 7.21-7.31 (m, 8H); ^{13}C NMR (100 MHz, $CDCl_3$) δ 49.6, 53.5, 64.0, 68.8, 73.0, 106.7, 121.7, 124.7, 127.1, 127.3, 127.4, 128.0, 128.4, 128.5, 128.8, 128.9, 137.6, 142.0, 150.7; ESI-HRMS (*m/z*): $(M+H)^+$ Calcd for $C_{23}H_{23}ClNO_2$, 380.1417; Found: 380.1414.

2.4.2 Synthesis of pinacol boronic ester 6a and 6aa

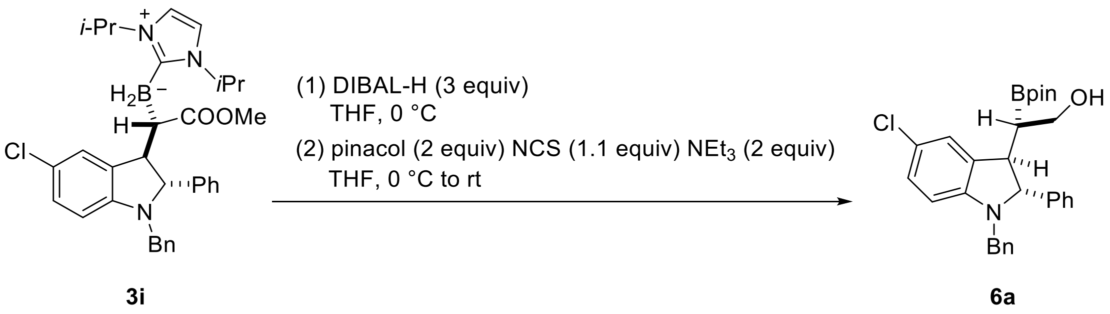

To a solution of **3i** (280 mg, 0.500 mmol) in anhydrous THF (5 ml) was slowly added DIBAL-H (0.95 mL, 1.500 mmol) at 0 °C. The reaction mixture was stirred at this temperature for 30 min. The reaction was quenched with potassium sodium tartrate salts solutions followed by filtration through celite and washed with ethyl acetate (50 ml). The solvent was evaporated and the residue was dissolved in 5ml toluene. NCS (73.0 mg, 0.550 mmol) and pinacol (119.0 mg, 1.000 mmol) and NEt_3 (0.14 ml, 1.000 mmol) were added. The mixture was stirred at 40 °C for 18 h. After evaporation, the residue was purified by flash column chromatography (silica gel; petroleum ether : ethyl acetate = 5 : 1) to give **6a** (161.7 mg, 0.330 mmol) in 66% yield as colorless oil; ^{1}H NMR (400 MHz, $CDCl_3$) δ 0.98 (s, 6H), 1.13 (s, 6H), 1.61-1.65 (m, BPinC*H,* 1H), 2.20 (s br, 1H), 3.40 (dd, *J* = 9.2, 2.8 Hz, 1H), 3.67 (dd, *J* = 10.0, 5.2 Hz, 1H), 3.87-3.92 (m, 2H), 4.28 (d, *J* = 15.6 Hz, 1H), 4.60 (d, *J* = 9.2 Hz, 1H), 6.22 (d, *J* = 8.4 Hz, 1H), 6.94 (dd, *J* = 8.4, 1.6 Hz, 1H), 7.11 (s, 1H), 7.18-7.34 (m, 8H), 7.40-7.42 (m, 2H); ^{13}C NMR (100 MHz, $CDCl_3$) δ 24.5, 24.9, 49.7, 50.5, 63.2, 74.1, 83.6, 107.4, 122.0, 124.2, 126.9, 127.3, 127.5, 127.9, 128.2, 128.3, 128.6, 133.2, 137.8, 141.3, 150.6; ^{11}B NMR (128.4 MHz, $CDCl_3$): δ 33.7 (1B, s br); ESI-HRMS (*m/z*): $(M+H)^+$ Calcd for $C_{29}H_{34}{}^{11}BClNO_3$, 490.2320; Found: 490.2320.

(*R)-2-((2*R**,3*R**)-1-benzyl-5-chloro-2-phenylindolin-3-yl)-2-(4,4,5,5-tetramethyl -1,3,2-dioxaborolan-2-yl)ethan-1-ol (6aa)**

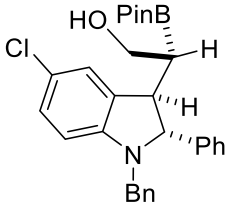

According to the same procedure as shown above, the reaction of **4ia** (404.0 mg, 0.806 mmol) afforded **6aa** (264.6 mg, 0.540 mmol) in 67% yield as colorless oil; ^{1}H NMR (400 MHz, $CDCl_3$) δ 1.20 (s, 6H), 1.21 (s, 6H), 1.73-1.77 (m, BPinC*H,* 1H), 2.00 (s br, 1H), 3.58 (dd, *J* = 7.2, 4.8 Hz, 1H), 3.63-3.74 (m, 2H), 3.89 (d, *J* = 16.0 Hz, 1H), 4.30 (d, *J* = 16.0 Hz, 1H), 4.59 (d, *J* = 7.2 Hz, 1H), 6.25 (d, *J* = 8.4 Hz, 1H), 6.97 (dd, *J* = 8.4, 1.6 Hz, 1H), 7.07 (s, 1H), 7.16-7.34 (m, 10H); ^{13}C NMR (100 MHz, $CDCl_3$) δ 24.7, 24.8, 48.9, 50.3, 60.9, 73.7, 83.8, 107.2, 121.9, 124.5, 127.0, 127.4, 127.5, 127.7, 127.9, 128.4, 128.6, 132.2, 137.7, 141.8, 150.4; ^{11}B NMR (128.4 MHz, $CDCl_3$): δ 34.0 (1B, s br); ESI-HRMS (*m/z*): $(M+H)^+$ Calcd for $C_{29}H_{34}{}^{11}BClNO_3$, 490.2320; Found: 490.2318.

2.4.3 Synthesis of furan-substituted indoline 7a and 7aa

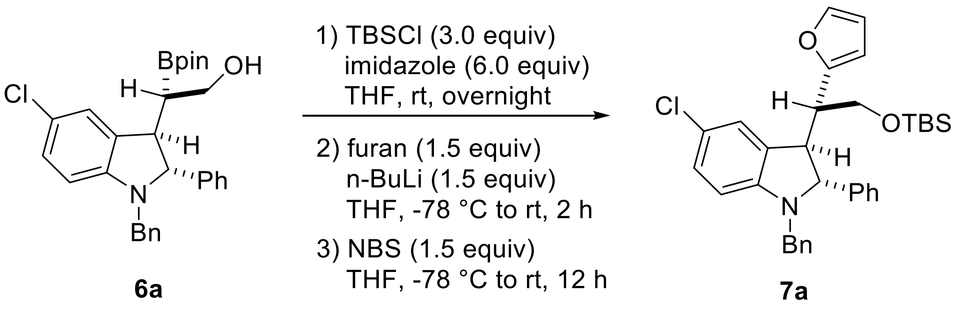

To a solution of **6a** (98.0 mg, 0.200 mmol) and imidazole (81.0 mg, 1.200 mmol) in anhydrous THF (1 ml) was slowly added the solution of TBSCl (90.4 mg, 0.600 mmol) in anhydrous THF (1 ml). The reaction mixture was stirred overnight at room temperature. After filtration to remove white precipitates, the filtrate was evaporated under *vacuo* giving TBS-protected boronic ester. A solution of furan (20.6 mg, 0.300 mmol) in THF (1 mL) was slowly added *n*-BuLi (0.6 mL, 1.6 M in hexanes) at –78 °C and then the cooling bath was removed. The mixture was stirred at room temperature for 2 h. The mixture was re-cooled to –78 °C and a solution of TBS-protected boronic

ester in THF (0.5 mL) was added dropwise. The mixture was stirred at –78 °C overnight and then a solution of NBS (54.0 mg, 0.300 mmol) in THF (0.5 mL) was added dropwise. The reaction was stirred at –78 °C for 12 h followed by quenching with saturated $Na_2S_2O_3$ (aq). The reaction mixture was allowed to warm to room temperature and was diluted with ethyl acetate. The aqueous layer was extracted with ethyl acetate three times. The combined organic layers were dried over anhydrous Na_2SO_4, filtered and concentrated under *vacuo*. The crude residue was purified by flash column chromatography on silica gel (petroleum ether : ethyl acetate = 100:1) to give **7a** (54.0 mg) in 50% yield as yellow oil. ^{1}H NMR (400 MHz, $CDCl_3$) δ -0.19 (s, 3H), -0.13 (s, 3H), 0.69 (s, 9H), 3.12 (ddd, *J* = 6.4, 6.4, 6.4 Hz, 1H), 3.67 (dd, *J* = 6.4, 5.6 Hz, 1H), 3.79-3.82 (m, 3H), 4.21 (d, *J* = 16.4 Hz, 1H), 4.48 (d, *J* = 6.4 Hz, 1H), 5.87 (d, *J* = 2.4 Hz, 1H), 6.13 (d, *J* = 8.4 Hz, 1H), 6.20 (s br, 1H), 6.66 (s, 1H), 6.90 (d, *J* = 8.4 Hz, 1H), 6.97-6.98 (m, 2H), 7.11-7.22 (m, 9H); ^{13}C NMR (100 MHz, $CDCl_3$) δ -5.6, 18.1, 25.8, 45.6, 49.6, 51.2, 63.1, 71.5, 106.3, 107.8, 110.2, 121.3, 125.3, 126.9, 127.2, 127.3, 127.67, 127.75, 128.4, 128.6, 130.6, 137.7, 140.9, 142.1, 150.5, 154.1; ESI-HRMS (*m/z*): $(M+H)^+$ Calcd for $C_{33}H_{39}ClNO_2Si$, 544.2439; Found: 544.2434.

(2*R,3*R**)-1-benzyl-3-((*R**)-2-((tert-butyldimethylsilyl)oxy)-1-(furan-2-yl)ethyl)-5-chloro-2-phenylindoline (7aa)**

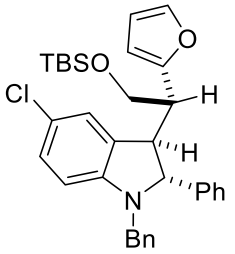

According to the same procedure as shown above, the reaction of **6aa** (100.0 mg, 0.204 mmol) afforded **7aa** (57.7 mg, 0.106 mmol) in 52% yield as yellow oil; ^{1}H NMR (400 MHz, $CDCl_3$) δ -0.16 (s, 3H), -0.09 (s, 3H), 0.78 (s, 9H), 3.24-3.29 (m, 1H), 3.75-3.90 (m, 4H), 4.26 (d, *J* = 16.4 Hz, 1H), 4.51 (d, *J* = 5.6 Hz, 1H), 5.97 (d, *J* = 3.2 Hz, 1H), 6.19 (d, *J* = 8.4 Hz, 1H), 6.28 (dd, *J* = 3.2, 2.0 Hz, 1H), 6.98 (dd, *J* = 8.4, 2.0 Hz, 1H), 7.05-7.07 (m, 3H), 7.10-7.13 (m, 2H), 7.20-7.29 (m, 7H); ^{13}C NMR

(100 MHz, $CDCl_3$) δ -5.7, 18.1, 25.8, 44.9, 49.4, 50.6, 62.1, 71.4, 106.3, 107.6, 110.1, 121.3, 125.3, 126.9, 127.2 (overlapped), 127.7 (overlapped), 128.4, 128.6, 131.1, 137.9, 141.1, 142.2, 150.4, 154.4; ESI-HRMS (*m/z*): $(M+H)^+$ Calcd for $C_{33}H_{39}ClNO_2Si$, 544.2439; Found: 544.2434.

2.4.4 Synthesis of homologated pinacol boronic ester 8a and 8aa

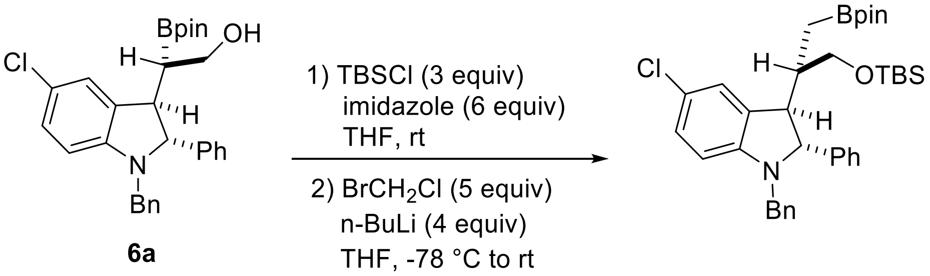

To a solution of **6a** (100.0 mg, 0.204 mmol) and imidazole (80.0 mg, 1.175 mmol) in anhydrous THF (1 ml) was slowly added the solution of TBSCl (92.4 mg, 0.613 mmol) in anhydrous THF (1 ml). The reaction mixture was stirred overnight at room temperature. After filtration to remove white precipitates, the filtrate was evaporated under *vacuo* giving TBS-protected boronic ester. A solution of the TBS-protected boronic ester in THF (1 mL) was slowly added $BrCH_2Cl$ (132.0 mg, 1.020 mmol) and *n*-BuLi (0.5 mL, 1.6 M in hexanes) at –78 °C. The reaction mixture was warmed to reflux overnight 30 min later. The reaction was quenched with water and the aqueous layer was extracted with ethyl acetate three times. The combined organic extracts were washed with brine, dried over Na_2SO_4, and concentrated in *vacuo*. The crude residue was purified by flash column chromatography on silica gel to give **8a (**58.0 mg) in 46% yield as yellow oil; ^1H NMR (400 MHz, $CDCl_3$) δ -0.08 (s, 3H), -0.02 (s, 3H), 0.79 (m, 11H), 1.14 (s, 6H), 1.15 (s, 6H), 2.11-2.16 (m, 1H), 3.52-3.60 (m, 3H), 3.90 (d, J = 16.0 Hz, 1H), 4.31 (d, J = 16.0 Hz, 1H), 4.46 (d, J = 6.8 Hz, 1H), 6.21 (d, J = 8.4 Hz, 1H), 6.96 (dd, J = 8.4, 2.0 Hz, 1H), 7.05 (s, 1H), 7.16-7.17 (m, 2H), 7.21-7.29 (m, 8H); ^{13}C NMR (100 MHz, $CDCl_3$) δ -5.40, -5.38, 18.2, 24.7, 24.8, 25.9, 40.1, 50.0, 52.5, 65.2, 71.4, 83.0, 106.3, 121.4, 125.1, 126.9, 127.2, 127.4, 127.5 (overlapped), 128.4, 128.5, 131.6, 138.0, 142.8, 150.8; ^{11}B NMR (128.4 MHz, $CDCl_3$): δ 34.3 (1B, s br); ESI-HRMS (*m/z*): $(M+H)^+$ Calcd for $C_{36}H_{50}{}^{11}BClNO_3Si$,

618.3342; Found: 618.3341.

(2*R,3*R**)-1-benzyl-3-((*R**)-1-((tert-butyldimethylsilyl)oxy)-3-(4,4,5,5-tetramethyl-1,3,2-dioxaborolan-2-yl)propan-2-yl)-5-chloro-2-phenylindoline (8aa)**

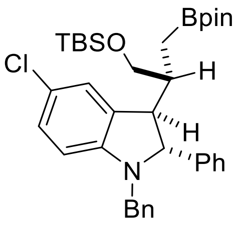

According to the same procedure as shown above, the reaction of **6aa** (100.0 mg, 0.204 mmol) afforded **8aa** (60.7 mg, 0.098 mmol) in 48% yield as yellow oil; ^{1}H NMR (400 MHz, $CDCl_3$) δ -0.19 (s, 3H), -0.09 (s, 3H), 0.73-0.84 (m, 11H), 1.15 (s, 6H), 1.17 (s, 6H), 2.25-2.31 (m, 1H), 3.35 (dd, *J* = 9.6, 8.4 Hz, 1H), 3.50 (dd, *J* = 9.6, 5.2 Hz, 1H), 3.68 (dd, *J* = 6.8, 3.6 Hz, 1H), 3.89 (d, *J* = 16.0 Hz, 1H), 4.29 (d, *J* = 16.0 Hz, 1H), 4.53 (d, *J* = 6.8 Hz, 1H), 6.19 (d, *J* = 8.4 Hz, 1H), 6.95 (dd, *J* = 8.4, 2.0 Hz, 1H), 7.05 (s, 1H), 7.15-7.17 (m, 2H), 7.20-7.27 (m, 8H); ^{13}C NMR (100 MHz, $CDCl_3$) δ -5.63, -5.56, 18.1, 24.6, 25.0, 25.8, 40.1, 49.8, 51.5, 64.7, 69.9, 83.0, 106.2, 121.4, 124.4, 126.9, 127.3, 127.4, 127.5, 127.6, 128.4, 128.5, 132.5, 138.0, 142.8, 150.7; ^{11}B NMR (128.4 MHz, $CDCl_3$): δ 34.3 (1B, s br); ESI-HRMS (*m/z*): $(M+H)^+$ Calcd for $C_{36}H_{50}{}^{11}BClNO_3Si$, 618.3342; Found: 618.3341.

2.5 ^{1}H NMR experiments for investigation of ZnI_2-chelation mode

Because **Int-III-B** is assumed to have a chelation with ZnI_2 similar to that of product **4aa**, we used ^{1}H NMR spectroscopy measurements to probe the possible chelation sites employing **4aa** as a model. Different equivalents of ZnI_2 (0.1 equiv, 0.25 equiv, 0.5 equiv, 0.8 equiv, 1 equiv, 1.5 equiv) was added separately into a 0.028 M solution of **4aa** (dissolved in 1.0 mL toluene-d_8) in a 10 mL sealed tube under nitrogen atmosphere and the mixture was stirred at 60 °C for 1 h. Then 0.5 mL supernatant solvent was transferred into an NMR tube and was measured immediately on a Bruker Avance 500 spectrometer. The ^{1}H NMR spectra were stacked and shown below. Upon addition of ZnI_2 (0-1.5 equiv), the protons locating on the triazole moiety (H'$_a$, H'$_b$ and H'$_c$) are shifted downfield. In addition, the proton of methoxycarbonyl group (H'$_d$)

also exhibits a downfield shift. These imply that both the triazole and carbonyl group are involved in binding with Zn^{II}. Moreover, only a single set of ^{1}H NMR spectrum is observed in all cases, which indicates a fast dynamics of complexation/ decomplexation between the free **4aa** and Zn^{II}-**4aa** chelators.

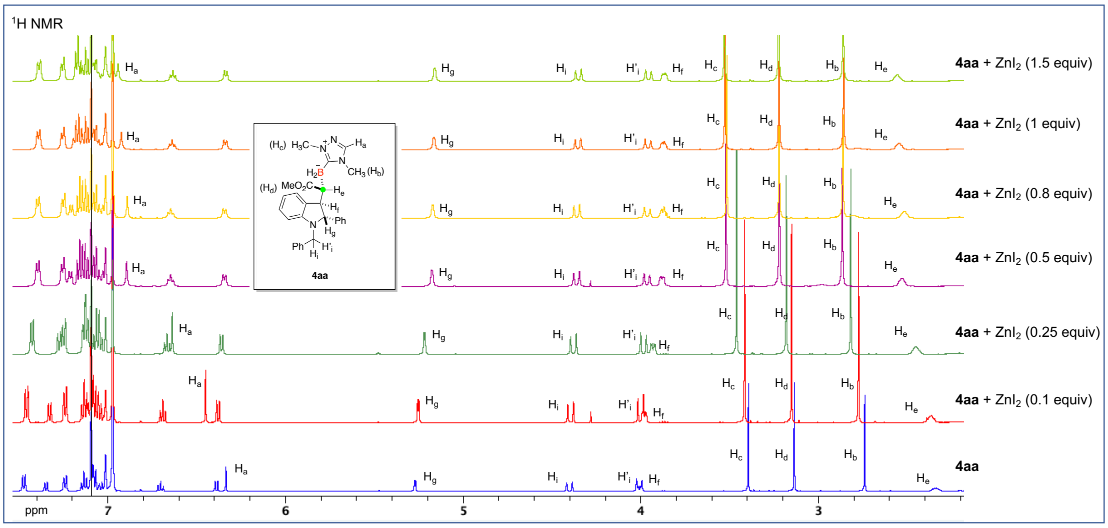

Supplementary Figure 1. Stacked ^{1}H NMR (500 MHz, toluene-d_8) spectra of **4aa** with increasing amount of ZnI$_2$

The chelation mode of ZnI$_2$ with NHC-BH$_3$ was also studied with ^{1}H NMR spectroscopy measurements. Different equivalents of ZnI$_2$ was added into a 0.127 M solution of **2a** (dissolved in 1.0 mL toluene-d_8) in a 10 mL sealed tube under nitrogen atmosphere and the mixture was stirred at 60 °C for 1 h. Then 0.5 mL supernatant solvent was transferred into an NMR tube and was measured immediately on a Bruker Avance 500 spectrometer. The ^{1}H NMR spectra were stacked and shown below. Upon addition of ZnI$_2$ (0-1.0 equiv), the protons on boron (H$_a$) exhibit an obvious downfield shift. Similarly, the protons locating on the triazole moiety (H$_b$ and H$_c$) also shifted downfield. Based on these results and previous reports on the coordination modes of NHC-BH$_3$ with transition metals,[20,21] it is highly possible that ZnI$_2$ has an interaction with the B-H bond in NHC-BH$_3$. Moreover, only a single set of ^{1}H NMR spectrum is observed in all cases, which indicates a fast dynamics of complexation/ decomplexation between the free **2a** and Zn^{II}-**2a** chelators.

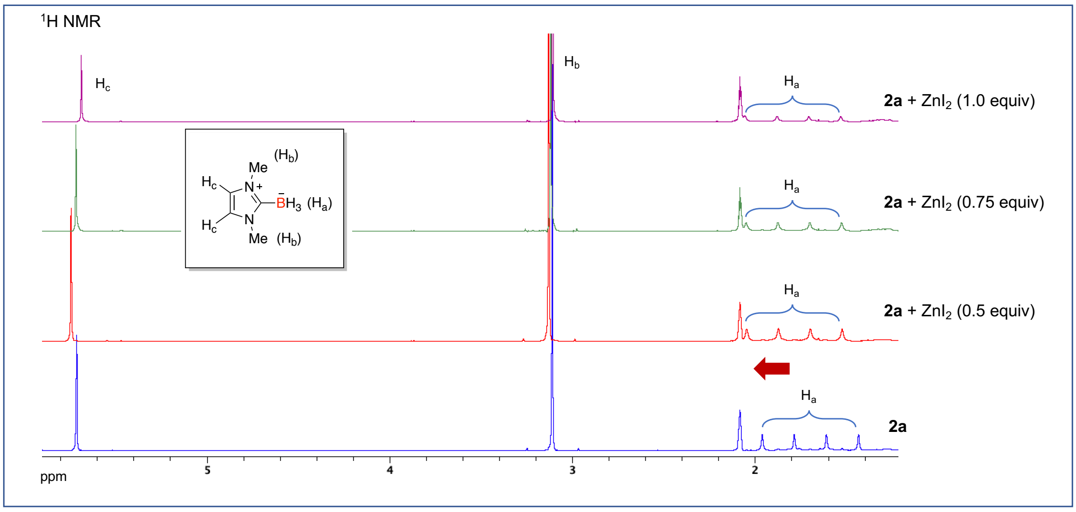

Supplementary Figure 2. Stacked ^{1}H NMR (400 MHz, toluene-d_8) spectra of **2a** with increasing amount of ZnI_2

Different equivalents of ZnI_2 was added into a 0.127 M solution of **2d** (dissolved in 1.0 mL toluene-d_8) in a 10 mL sealed tube under nitrogen atmosphere and the mixture was stirred at 60 °C for 1 h. Then 0.5 mL supernatant solvent was transferred into a NMR tube and was measured immediately on a Bruker Avance 500 spectrometer. Only a single set of ^{1}H NMR spectrum is observed in all cases, which indicates a fast dynamics of complexation/ decomplexation between the free **2d** and Zn^{II}-**2d** chelators.

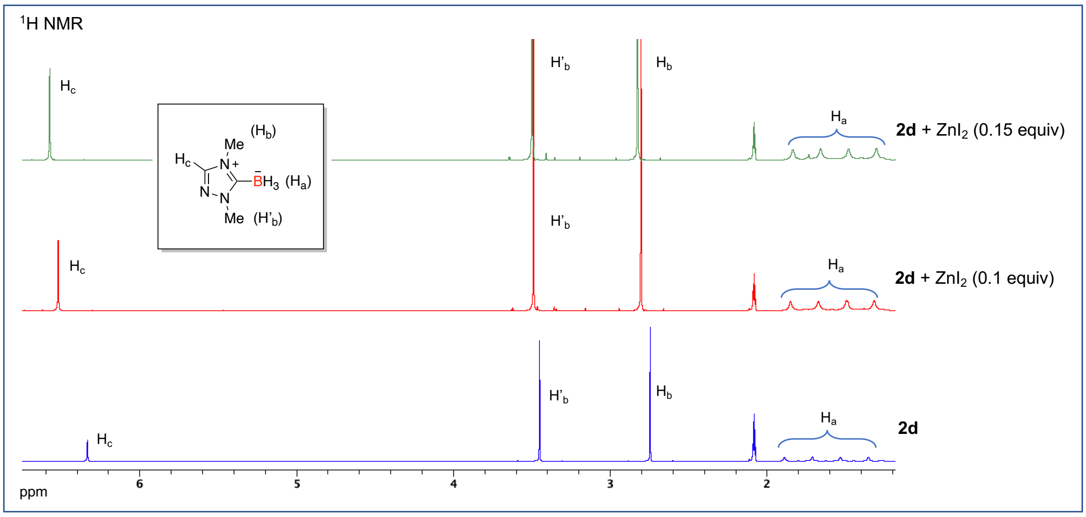

Supplementary Figure 3. Stacked ^{1}H NMR (400 MHz, toluene-d_8) spectra of **2d** with increasing amount of ZnI_2

2.6 Computational studies

2.6.1 Computational Methods

Calculations were carried out with Gaussian 16 software packages.[22] The (U)B3LYP functional[23,24] was used with an empirical dispersion correction (Grimme-D3(0))[25] for all the calculations. The geometries of all the stationary points were optimized in gas-phase with the SDD basis set[26] (Stuttgart/Dresden ECP) for Zn and I, and the 6-31G(d,p) basis set[27] for the other atoms. In the computational studies of HAT controlled by NHC-BH_3/thiol catalyst, the internal six d-type orbitals were used for all elements in geometry optimization part. In the computational studies of diastereoselective HAT controlled by NHC-BH_3/Lewis acid, the keyword "5D" was used to specify that five d-type orbitals were used for all elements in the calculations. Vibrational frequency analysis was calculated at the same level of theory to validate each structure as either a minimum or a transition state. For each transition state, the intrinsic reaction coordinate (IRC) analysis[28] was conducted to ensure that it connects the right reactant and product. To obtain more accurate energies, single point energies were calculated with a mixed basis set of SDD for Zn and I, 6-311+G(d,p)[27] for all the other atoms. The distortion/interaction analysis[29-31] was performed with gas-phase single point energies, while all the other energies reported in the main manuscript were obtained from solution-phase single point energies with SMD solvation model[32] (solvent = toluene). Noncovalent interaction (NCI) analysis[33] was performed with Multiwfn[34] by using IGM method.[35] The results were visualized by VMD software.[36] 3D structures were generated by CYLview.[37]

Supplementary Table 7. Single point energies (E, hartree) for **Int-III-A** with varied frozen dihedral angles of B-C_1-C_2-C_3

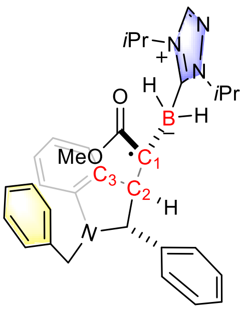

Int-III-A

ϕ = D(B-C$_1$-C$_2$-C$_3$)

ϕ	E[a]	ϕ	E[a]
-179.5°	-1621.090807	0.1°	-1621.097253
-150°	-1621.097546	30°	-1621.100218
-120°	-1621.099974	60°	-1621.098284
-90°	-1621.097132	90°	-1621.100713
-60°	-1621.091900	120°	-1621.096324
-30°	-1621.090500	150°	-1621.089526

[a] Computed at the SMD(toulene)/(U)B3LYP-D3/6-311+G(d,p)//(U)B3LYP-D3/6-31G(d,p) level (the internal six d-type orbitals were used)

Supplementary Table 8. Thermal correction to Gibbs free energy (TCG) and Single point energies (E) in hartree for all the other species in the computational studies of HAT controlled by NHC-BH_3/thiol catalyst

Compounds	TCG[a]	E[b]	E[c]
**Int-III-A**	0.587514	-1621.099976	-1621.068319
**RSH E**	0.106378	-858.496603	-858.4856705
**Int-III-A -cpx-1**	0.716537	-2479.615908	-2479.577248
**TS-A-major**	0.714728	-2479.601156	-2479.562076
**3a-i-Pr**	0.600723	-1621.748963	
**Int-III-A -cpx-2**	0.717905	-2479.611494	-2479.573476
**TS-A-minor**	0.715689	-2479.598536	-2479.559922
**4a-i-Pr**	0.601665	-1621.750517	
**RS• E**	0.097230	-857.858810	

[a] Computed at the (U)B3LYP-D3/6-31G(d,p) level (the internal six d-type orbitals were used)

[b] Computed at the SMD(toulene)/(U)B3LYP-D3/6-311+G(d,p)//(U)B3LYP-D3/6-31 G(d,p) level
[c] Computed at the (U)B3LYP-D3/6-311+G(d,p)//(U)B3LYP-D3/6-31G(d,p) level

Supplementary Table 9. Thermal correction to Gibbs free energy (TCG) and Single point energies (E) in hartree for all the species in the computational studies of diastereoselective HAT controlled by NHC-BH_3/Lewis acid

Compounds	TCG[a]	E[b]	E[c]
**Int-III-B-1**	0.459957	-1729.971910	-1729.931191
**Int-III-B-2**	0.459957	-1729.96712	
**Int-III-B-3**	0.457278	-1729.958690	
**Int-III-B-4**	0.465243	-1729.972362	
**2d**-ZnI_2 complex	0.103582	-597.795933	-597.7642901
**TS-B-major-1**	0.591867	-2327.777595	-2327.713783
**TS-B-major-2**	0.587573	-2327.764551	
**TS-B-major-3**	0.594712	-2327.766701	
**TS-B-major-4**	0.592896	-2327.768348	
**TS-B-minor-1**	0.589165	-2327.769598	-2327.708742
**TS-B-minor-2**	0.586020	-2327.764600	
**TS-B-minor-3**	0.588914	-2327.758402	
**TS-B-minor-4**	0.593022	-2327.769142	
**Int-III-B-Me**	0.383377	-1498.843842	-1498.806723
**TS-B-major-Me**	0.515119	-2096.64872	-2096.589834
**TS-B-minor-Me**	0.512882	-2096.646680	-2096.588658
**2d**	0.114909	-347.651530	-347.6360599
**TS-B-major'**	0.599224	-2077.627908	-2077.580197
**TS-B-minor'**	0.598977	-2077.62162	-2077.576158
**TS-B-major-Me’**	0.520640	-1846.497533	-1846.454346
**TS-B-minor-Me'**	0.52274	-1846.498418	-1846.456131

[a] Computed at the (U)B3LYP-D3/6-31G(d,p) level (the keyword “5D” was used)
[b] Computed at the SMD(toulene)/(U)B3LYP-D3/SDD-6-311+G(d,p)//(U)B3LYP-D3/SDD-6-31G(d,p) level
[c] Computed at the (U)B3LYP-D3/SDD-6-311+G(d,p)//(U)B3LYP-D3/SDD-6-31 G(d,p) level

2.5.2 Additional Computational Results

To verify whether the C_1-C_2 bond in **Int-III-A** is free to rotate, we firstly made efforts to locate the C_1-C_2 bond rotation transition state with various methods.

However, all of these trials failed. Then we turned to estimate the rotation barrier by single point energy calculations of the optimized structures with the frozen dihedral angles of B-C_1-C_2-C_3. Twelve dihedral angles varied from -179.5° to 150° were selected, and the single point energies of corresponding structures were shown below. The results reveal that ~7 kcal/mol of energy barrier is required for the free rotation of C_1-C_2 bond in **Int-III-A**. This energy is lower than the activation Gibbs energies of HATs, which are 8.3 and 10.6 kcal/mol, respectively. These results indicate the stereochemistry is determined by the relative HAT transition states, based on the Curtin-Hammett principle.

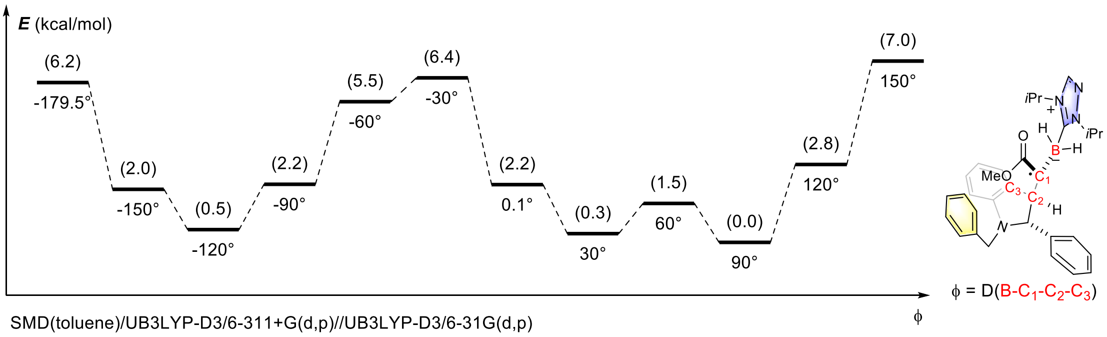

Supplementary Figure 4. Estimation of rotational barrier of C_1-C_2 bond in **Int-III-A**

The major and minor transition states of HAT step with different chelation modes were located. The results imply that **TS-B-major-1** and **TS-B-minor-1**, in which the **Int-III** adopts ZnI_2-chelated six-membered ring chelation mode are the most favorable, respectively.

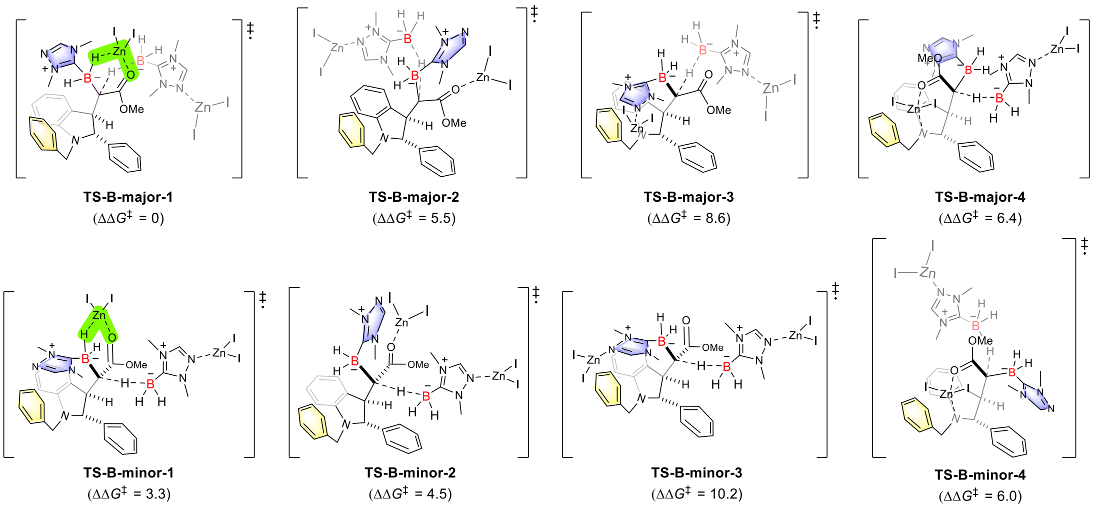

Supplementary Figure 5. Comparison of **TS-B-major** and **TS-B-minor** with

different chelation modes

In the part of NHC-BH3/Lewis acid-controlled stereoselective synthesis of borylated molecules, the existence of the *N*-Bn group on the substrates is crucial for the good diastereoselectivity. Poor diastereoselectivities were observed in the reactions of **1b**, **1l**, **1n**, where *N*-alkyl and *N*-phenyl groups are employed instead of N-benzyl group. To explain this phenomenon, HAT process of **1b** in the presence of ZnI_2 were computed. As shown below, the major and minor transition states possess comparable Gibbs free energies, which is in line with the experimental results. Further DIA shows that replacing *N*-Bn with *N*-Me results in a dramatic decrease of the distortion energy of **Int-III-B**, thus resulting in the complete abolish of diastereoselectivity. The alleviation of distortion energy can be attributed to less steric congestion between then Zn-chelated six-membered ring and the *N*-Me moiety compared with *N*-Bn group.

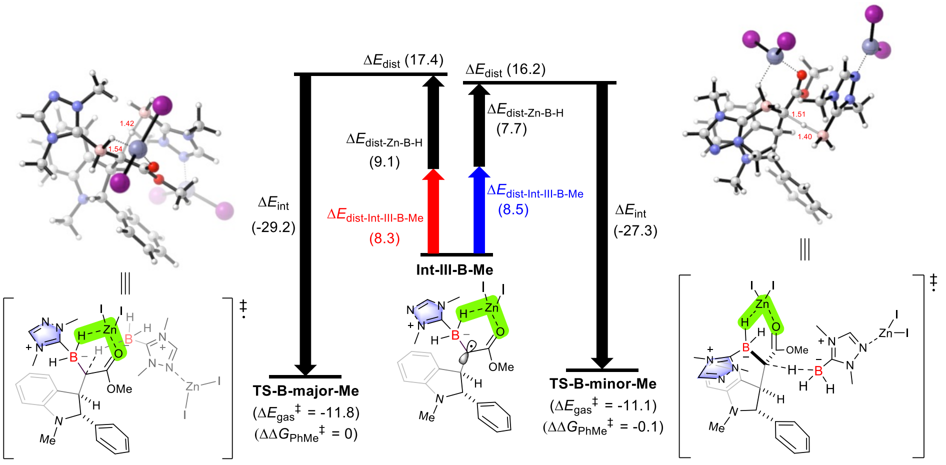

Supplementary Figure 6. Analysis of the diastereoselectivity in the NHC-BH_3/ZnI_2-controlled HAT process of **Int-III-B-Me**

Computational studies of the HAT from free NHC-BH_3 **2d** to **Int-III-B-1** were also conducted. The results show that the use of free **2d** or **2d**-ZnI_2 complex as the hydrogen atom donor has negligible effect on the diastereoselectivity.

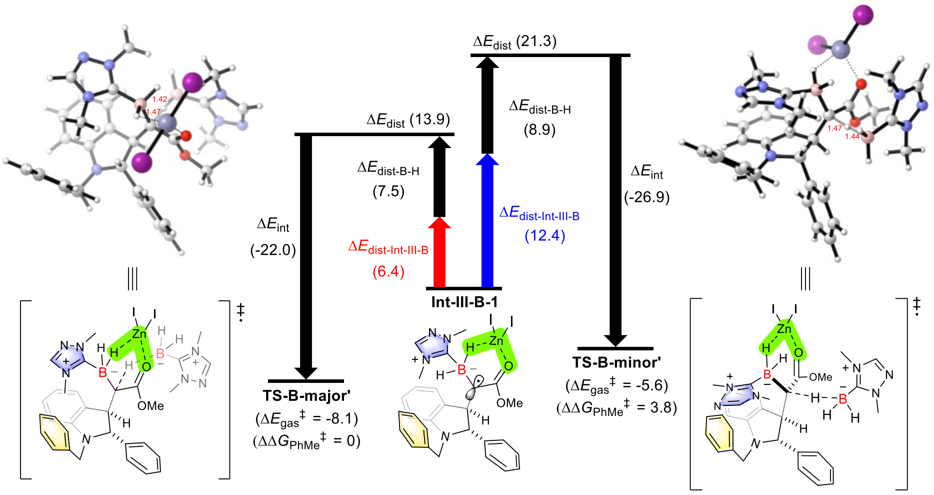

Supplementary Figure 7. HAT from **2d** to **Int-III-B**

Computational studies of the HAT from **2d** to **Int-III-B-Me** were also conducted. Similar to the HAT from **2d**-ZnI_2 complex to **Int-III-B-Me**, the poor diastereoselectivity of **1b** is due to a dramatic decrease of the distortion energy of **Int-III-B**, which originates from the less steric congestion between then Zn-chelated six-membered ring and the *N*-Me moiety.

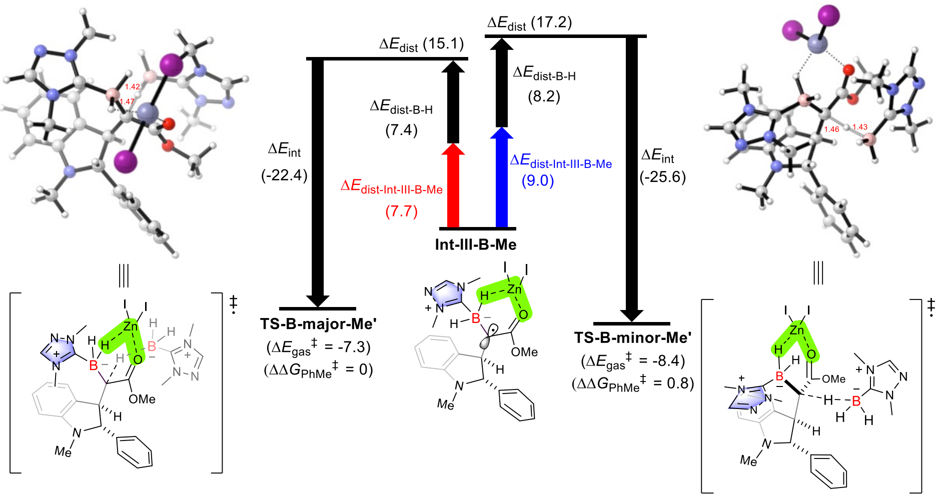

Supplementary Figure 8. HAT from **2d** to **Int-III-B-Me**

3. Supplementary References

1 Black, M., Cadogan, J. I. G. & Mcnab, H. Formation of dibenzofurans by flash vacuum pyrolysis of aryl 2-(allyloxy)benzoates and related reactions. *Org. Biomol. Chem.* **8**, 2961 (2010).

2 Murayama, T., Shibuya, M. & Yamamoto, Y. Synthesis of 3,4-Fused

2-Quinolones from an (ortho-Aminophenyl)propiolate via Sequential Cycloaddition/Lactam Formation. *Adv. Synth. Catal.* **357**, 690 (2015).
3 Brahmi, M. M., Monot, J., Desage-El Murr, M., Curran, D. P., Fensterbank, L., Lacôte, E. & Malacria, M. Preparation of NHC Borane Complexes by Lewis Base Exchange with Amine− and Phosphine−Boranes. *J. Org. Chem.* **75**, 6983 (2010).
4 Gardner, S., Kawamoto, T. & Curran, D. P. Synthesis of 1,3-Dialkylimidazol-2-ylidene Boranes from 1,3-Dialkylimidazolium Iodides and Sodium Borohydride. *J. Org. Chem.* **80**, 9794 (2015).
5 Ueng, S.-H., Makhlouf Brahmi, M., Derat, É., Fensterbank, L., Lacôte, E., Malacria, M. & Curran, D. P. Complexes of Borane and N-Heterocyclic Carbenes: A New Class of Radical Hydrogen Atom Donor. *J. Am. Chem. Soc.* **130**, 10082 (2008).
6 Jonckers, T. H. M., Van Miert, S., Cimanga, K., Bailly, C., Colson, P., De Pauw-Gillet, M.-C., Van Den Heuvel, H., Claeys, M., Lemière, F., Esmans, E. L., Rozenski, J., Quirijnen, L., Maes, L., Dommisse, R., Lemière, G. L. F., Vlietinck, A. & Pieters, L. Synthesis, Cytotoxicity, and Antiplasmodial and Antitrypanosomal Activity of New Neocryptolepine Derivatives. *J. Med. Chem.* **45**, 3497 (2002).
7 Sakai, N., Annaka, K. & Konakahara, T. Direct Synthesis of Polysubstituted Quinoline Derivatives by $InBr_3$-Promoted Dimerization of 2-Ethynylaniline Derivatives. *J. Org. Chem.* **71**, 3653 (2006).
8 Trost, B. M. & Mcclory, A. Rhodium-Catalyzed Cycloisomerization: Formation of Indoles, Benzofurans, and Enol Lactones. *Angew. Chem. Int. Ed.* **46**, 2074 (2007).
9 He, M., Chen, N., Zhou, T., Li, Q., Li, H., Lang, M., Wang, J. & Peng, S. Copper-Catalyzed Tandem Cross-Coupling/[2 + 2] Cycloaddition of 1,6-Allenynes with Diazo Compounds to 3-Azabicyclo[5.2.0] Ring Systems. *Org. Lett.* **21**, 9559 (2019).
10 Rosillo, M., Domínguez, G., Casarrubios, L., Amador, U. & Pérez-Castells, J. Tandem Enyne Metathesis-Diels−Alder Reaction for Construction of Natural Product Frameworks. *J. Org. Chem.* **69**, 2084 (2004).
11 Jiménez, J., Kim, B.-S. & Walsh, P. J. Tandem C(sp^3)−H Arylation/Oxidation and Arylation/Allylic Substitution of Isoindolinones. *Adv. Synth. Catal.* **358**, 2829 (2016).
12 Pechulis, A. D., Beck, J. P., Curry, M. A., Wolf, M. A., Harms, A. E., Xi, N., Opalka, C., Sweet, M. P., Yang, Z., Vellekoop, A. S., Klos, A. M., Crocker, P. J., Hassler, C., Laws, M., Kitchen, D. B., Smith, M. A., Olson, R. E., Liu, S. & Molino, B. F. 4-Phenyl tetrahydroisoquinolines as dual norepinephrine and dopamine reuptake inhibitors. *Bioorg. Med. Chem. Lett.* **22**, 7219 (2012).
13 Adachi, S., Onozuka, M., Yoshida, Y., Ide, M., Saikawa, Y. & Nakata, M. Smooth Isoindolinone Formation from Isopropyl Carbamates via Bischler–Napieralski-Type Cyclization. *Org. Lett.* **16**, 358 (2014).
14 Pyun, S. Y., Lee, D. C., Seung, Y. J. & Cho, B. R. Elimination Reactions of

N-Alkyl-N-chlorothenylamines Promoted by MeONa−MeOH and Et2NH−MeCN. Effect of the β-Aryl Group on the Imine-Forming Transition State. *J. Org. Chem.* **70**, 5327 (2005).
15 Chen, D.-F., Han, Z.-Y., He, Y.-P., Yu, J. & Gong, L.-Z. Metal-Free Oxidation/C(sp3)-H Functionalization of Unactivated Alkynes Using Pyridine-N-Oxide as the External Oxidant. *Angew. Chem. Int. Ed.* **51**, 12307 (2012).
16 Solé, D. & Serrano, O. Palladium-Catalyzed Intramolecular Nucleophilic Substitution at the Alkoxycarbonyl Group. *Angew. Chem. Int. Ed.* **46**, 7270 (2007).
17 Shen, H., Fu, J., Yuan, H., Gong, J. & Yang, Z. Synthesis of 2,3-Disubstituted Indoles and Benzofurans by the Tandem Reaction of Rhodium(II)-Catalyzed Intramolecular C–H Insertion and Oxygen-Mediated Oxidation. *J. Org. Chem.* **81**, 10180 (2016).
18 Gao, Y., Zhang, P., Li, G. & Zhao, Y. Cascade Annulation of 2-Alkynylthioanisoles with Unsaturated alpha-Bromocarbonyls Leading to Thio-Benzobicyclic Skeletons. *J. Org. Chem.* **83**, 13726 (2018).
19 Weng, Z., Li, H., He, W., Yao, L.-F., Tan, J., Chen, J., Yuan, Y. & Huang, K.-W. Mild copper-catalyzed trifluoromethylation of terminal alkynes using an electrophilic trifluoromethylating reagent. *Tetrahedron* **68**, 2527 (2012).
20 Bissinger, P., Braunschweig, H., Kupfer, T. & Radacki, K. Monoborane NHC Adducts in the Coordination Sphere of Transition Metals. *Organometallics* **29**, 3987 (2010).
21 Saha, K., Roy, D. K., Dewhurst, R. D., Ghosh, S. & Braunschweig, H. Recent Advances in the Synthesis and Reactivity of Transition Metal σ-Borane/Borate Complexes. *Acc. Chem. Res.* **54**, 1260 (2021).
22 Gaussian 16 Rev. C.01 (Wallingford, CT, 2016).
23 Lee, C., Yang, W. & Parr, R. G. Development of the Colle-Salvetti correlation-energy formula into a functional of the electron density. *Phys. Rev. B* **37**, 785 (1988).
24 Becke, A. D. Density-functional thermochemistry. III. The role of exact exchange. *J. Chem. Phys.* **98**, 5648 (1993).
25 Grimme, S., Antony, J., Ehrlich, S. & Krieg, H. A consistent and accurate ab initio parametrization of density functional dispersion correction (DFT-D) for the 94 elements H-Pu. *J. Chem. Phys.* **132**, 154104 (2010).
26 Andrae, D., Häußermann, U., Dolg, M., Stoll, H. & Preuß, H. Energy-adjustedab initio pseudopotentials for the second and third row transition elements. *Theor. Chim. Acta* **77**, 123 (1990).
27 Hehre, W. J., Radom, L., Schleyer, P. v. R. & Pople, J. A. *Ab Initio Molecular Orbital Theory*. (Wiley, 1986).
28 Fukui, K. The path of chemical reactions - the IRC approach. *Acc. Chem. Res.* **14**,

363 (1981).
29 Bickelhaupt, F. M. & Houk, K. N. Analyzing Reaction Rates with the Distortion/Interaction-Activation Strain Model. *Angew. Chem. Int. Ed.* **56**, 10070 (2017).
30 Fernández, I. & Bickelhaupt, F. M. The activation strain model and molecular orbital theory: understanding and designing chemical reactions. *Chem. Soc. Rev.* **43**, 4953 (2014).
31 Van Zeist, W.-J. & Bickelhaupt, F. M. The activation strain model of chemical reactivity. *Org. Biomol. Chem.* **8**, 3118 (2010).
32 Marenich, A. V., Cramer, C. J. & Truhlar, D. G. Universal Solvation Model Based on Solute Electron Density and on a Continuum Model of the Solvent Defined by the Bulk Dielectric Constant and Atomic Surface Tensions. *J. Phys. Chem. B* **113**, 6378 (2009).
33 Johnson, E. R., Keinan, S., Mori-Sánchez, P., Contreras-García, J., Cohen, A. J. & Yang, W. Revealing Noncovalent Interactions. *J. Am. Chem. Soc.* **132**, 6498 (2010).
34 Lu, T. & Chen, F. Multiwfn: A multifunctional wavefunction analyzer. *J. Comput. Chem.* **33**, 580 (2012).
35 Lefebvre, C., Rubez, G., Khartabil, H., Boisson, J.-C., Contreras-García, J. & Hénon, E. Accurately extracting the signature of intermolecular interactions present in the NCI plot of the reduced density gradient versus electron density. *Phys. Chem. Chem. Phys.* **19**, 17928 (2017).
36 Humphrey, W., Dalke, A. & Schulten, K. VMD: Visual molecular dynamics. *J. Mol. Graph.* **14**, 33 (1996).
37 Legault, C. Y. CYLview, 1.0b (Université de Sherbrooke, 2009); www.cylview.org.

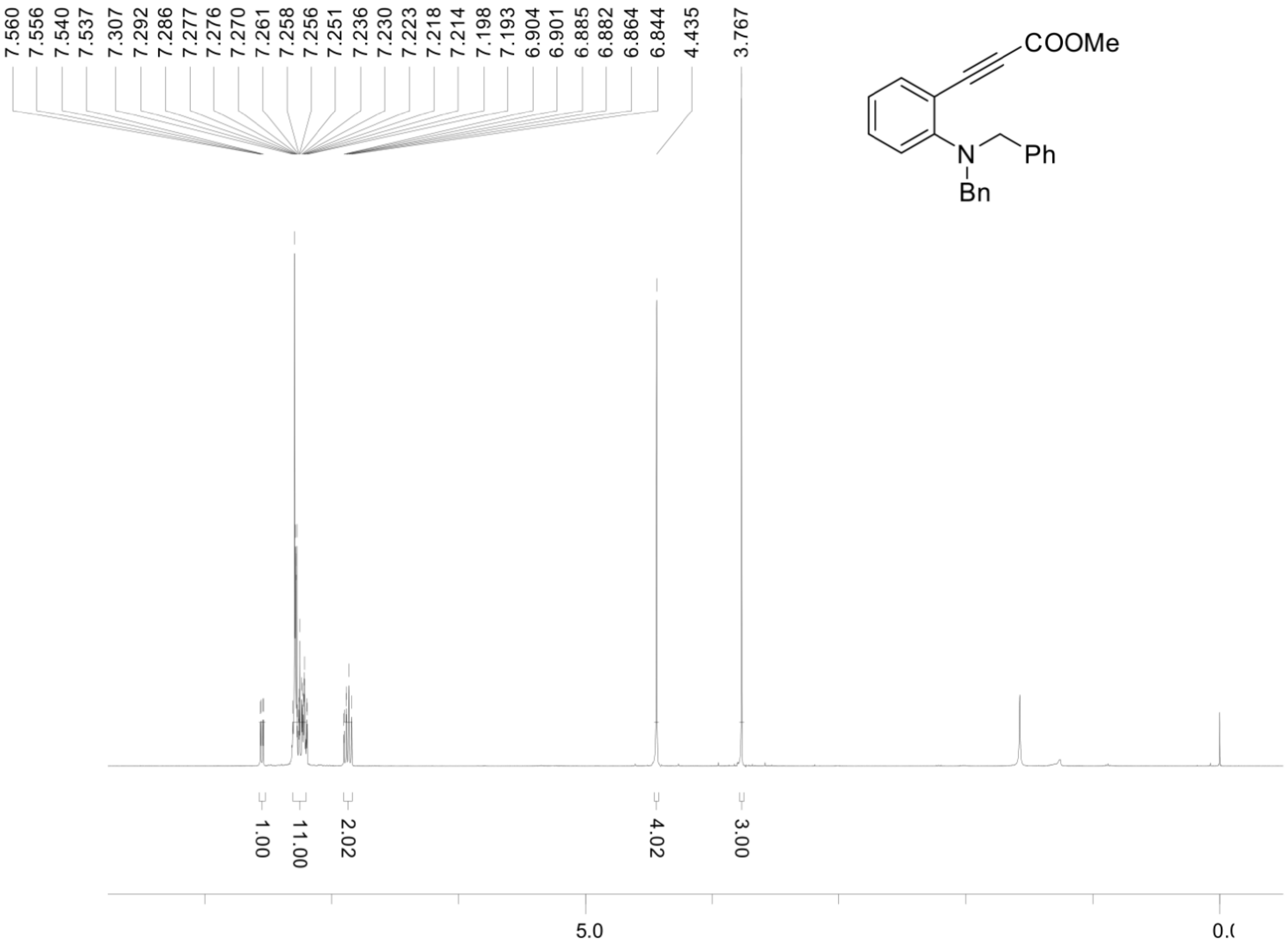

Supplementary Figure 9. ^{1}H NMR spectrum of **1a** (400 MHz, $CDCl_3$)

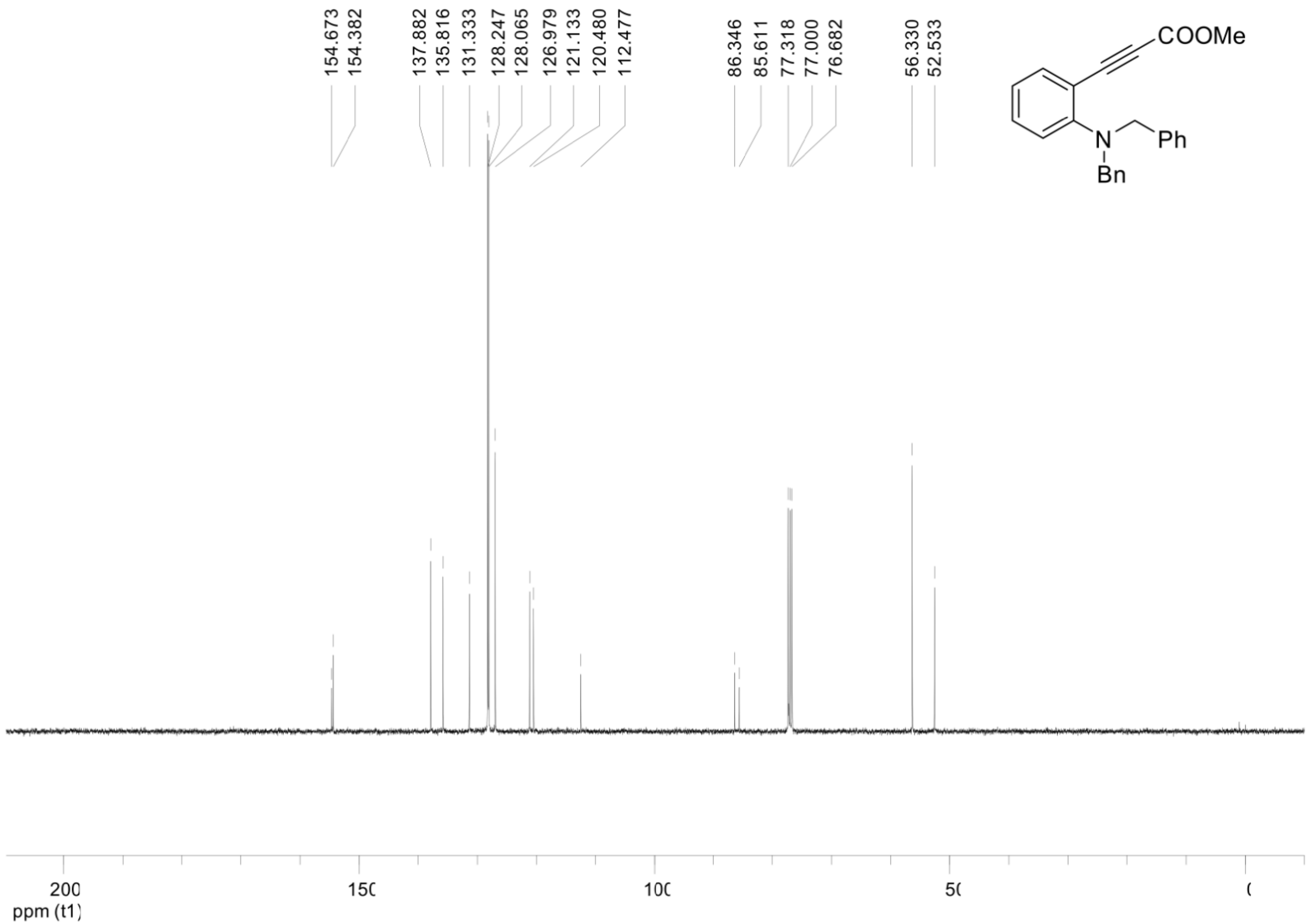

Supplementary Figure 10. ^{13}C NMR spectrum of **1a** (100 MHz, $CDCl_3$)

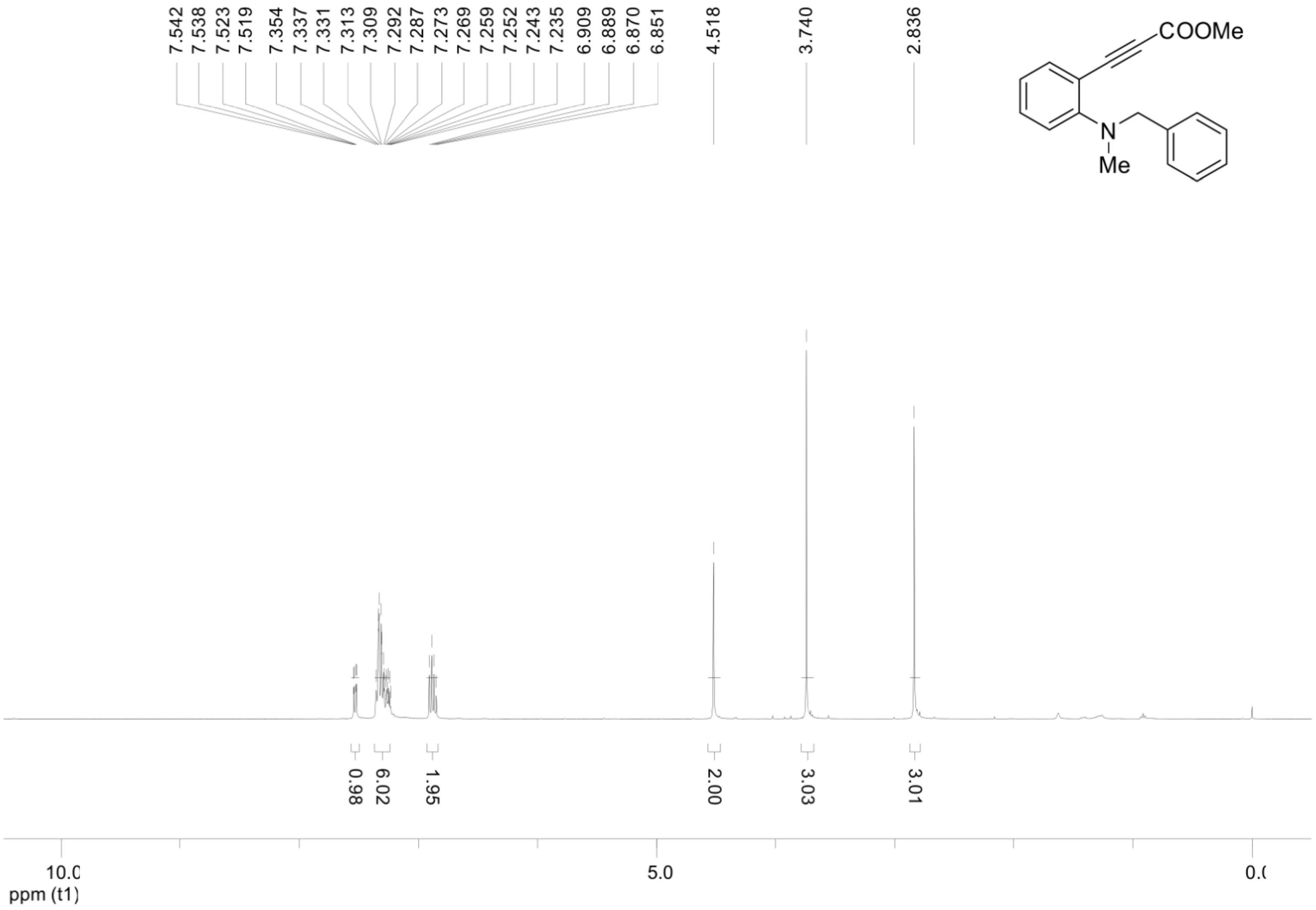

Supplementary Figure 11. ^{1}H NMR spectrum of **1b** (400 MHz, $CDCl_3$)

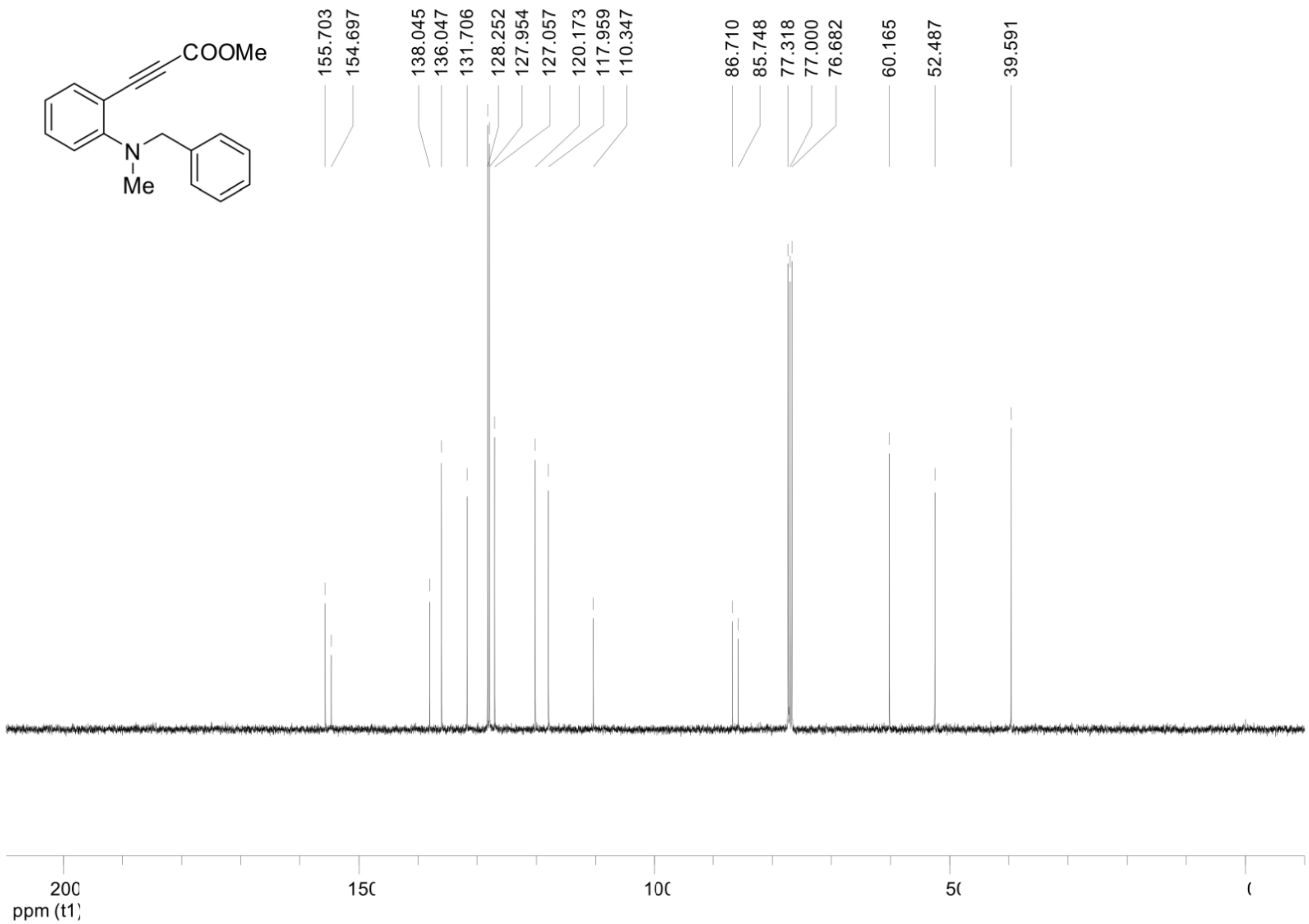

Supplementary Figure 12. ^{13}C NMR spectrum of **1b** (100 MHz, $CDCl_3$)

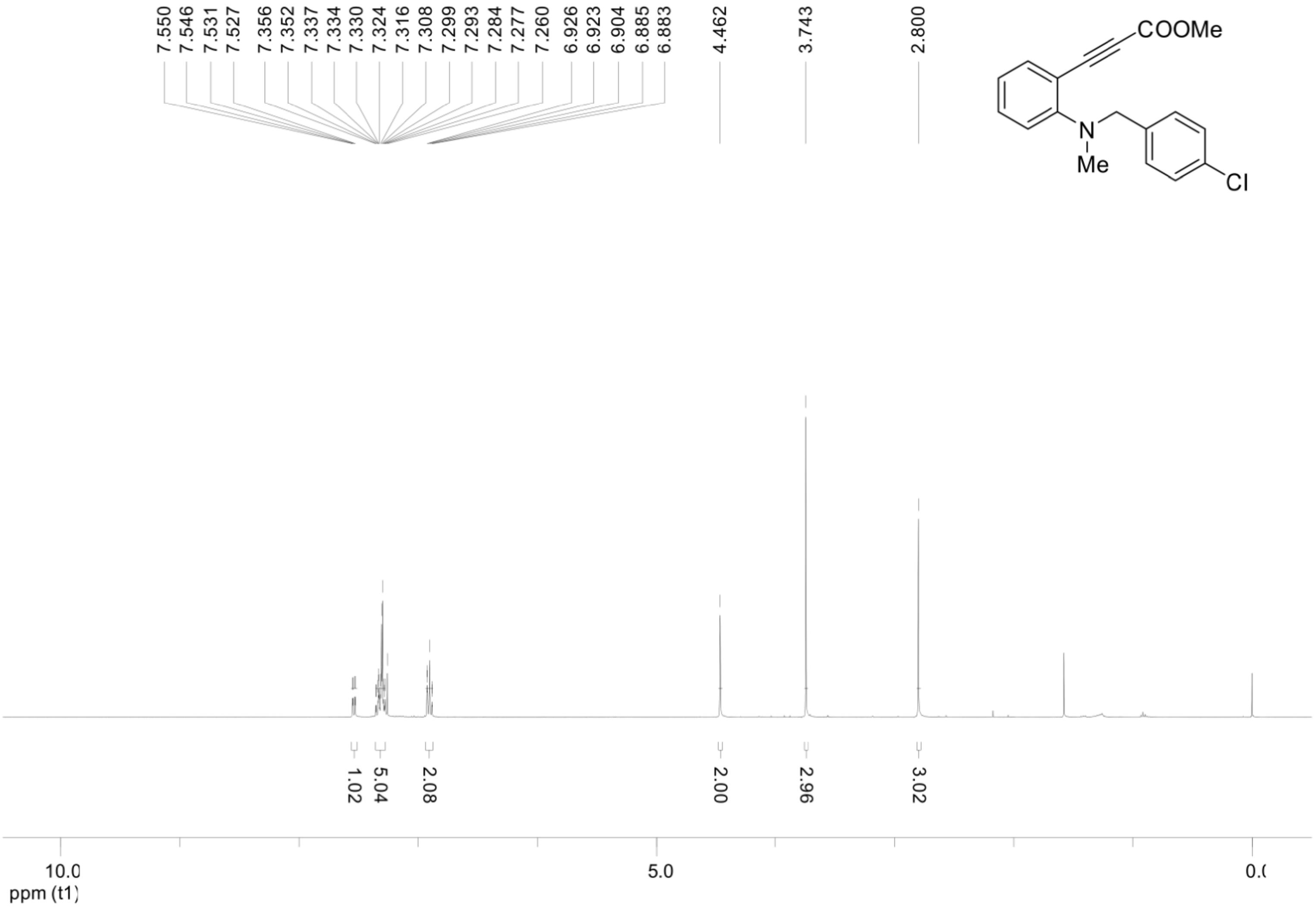

Supplementary Figure 13. ^{1}H NMR spectrum of **1c** (400 MHz, $CDCl_3$)

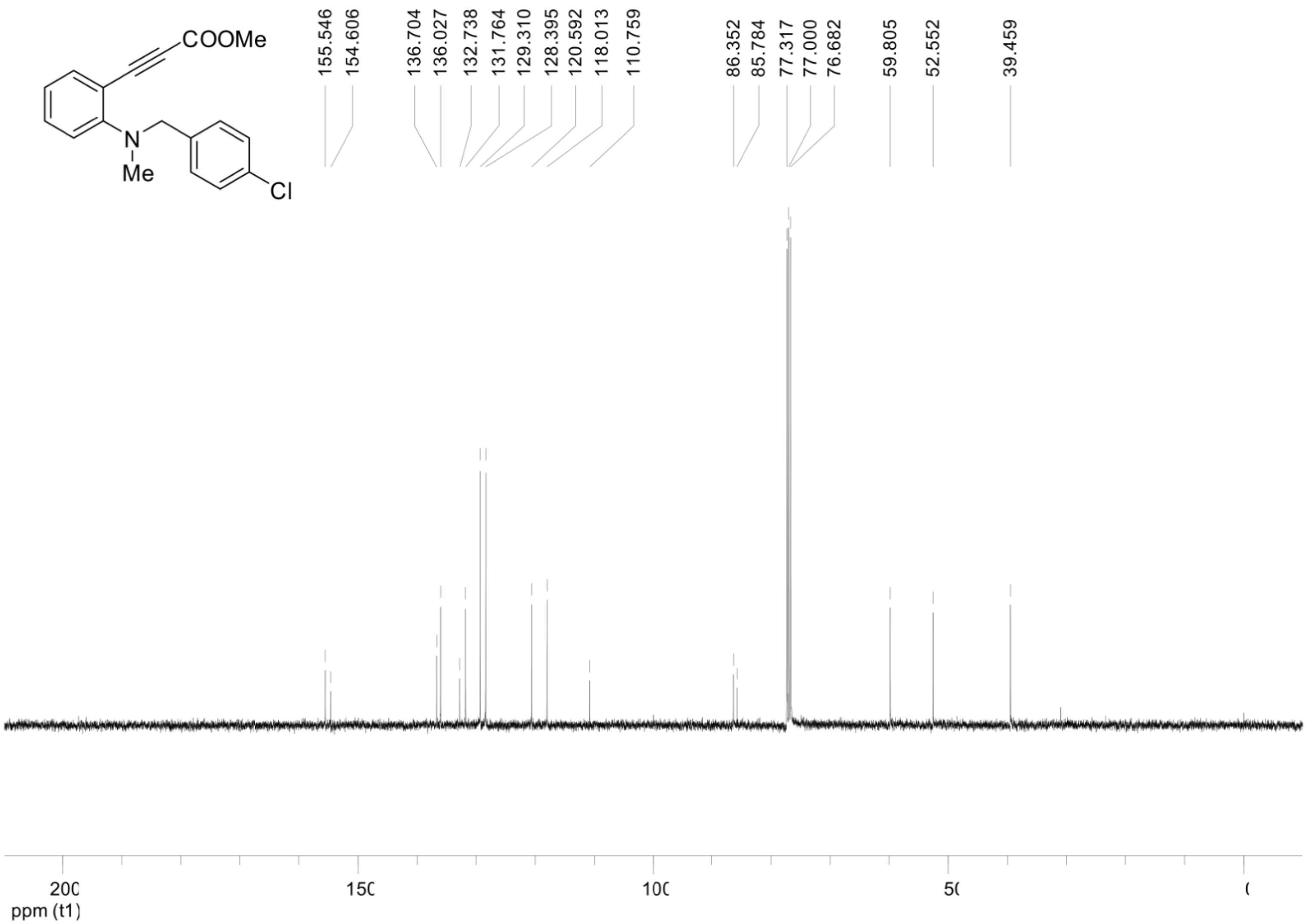

Supplementary Figure 14. ^{13}C NMR spectrum of **1c** (100 MHz, $CDCl_3$)

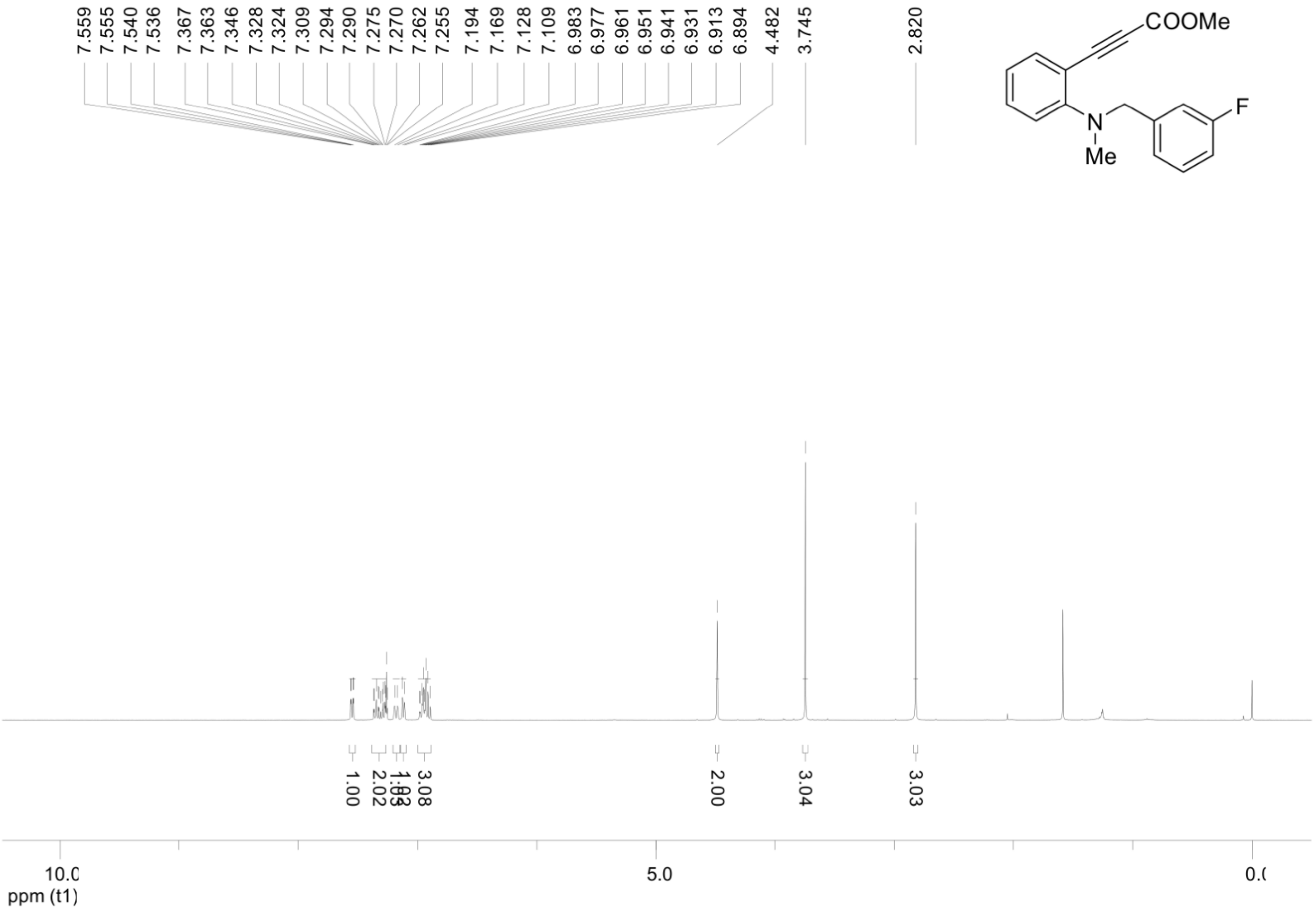

Supplementary Figure 15. ^{1}H NMR spectrum of **1d** (400 MHz, $CDCl_3$)

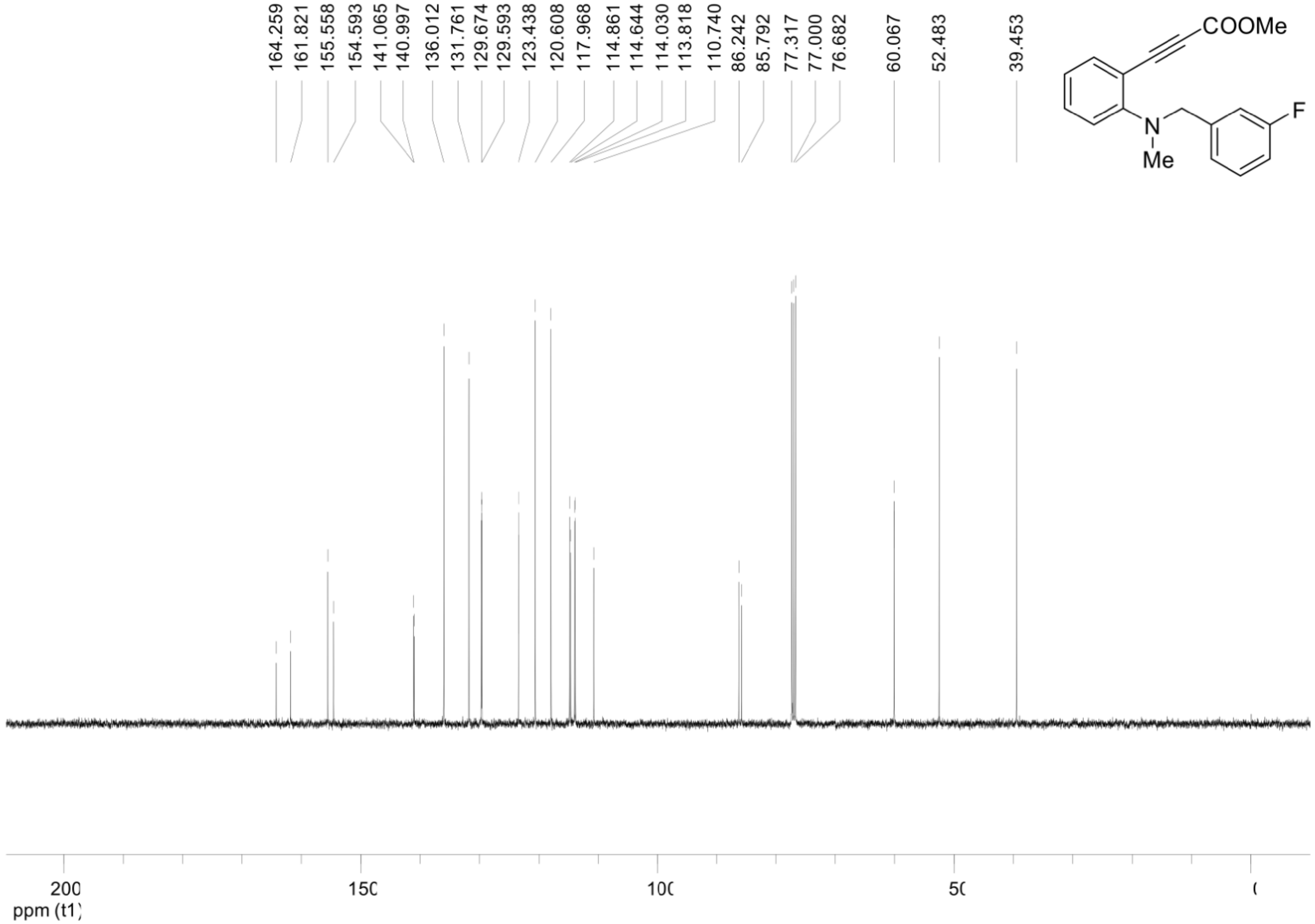

Supplementary Figure 16. ^{13}C NMR spectrum of **1d** (100 MHz, $CDCl_3$)

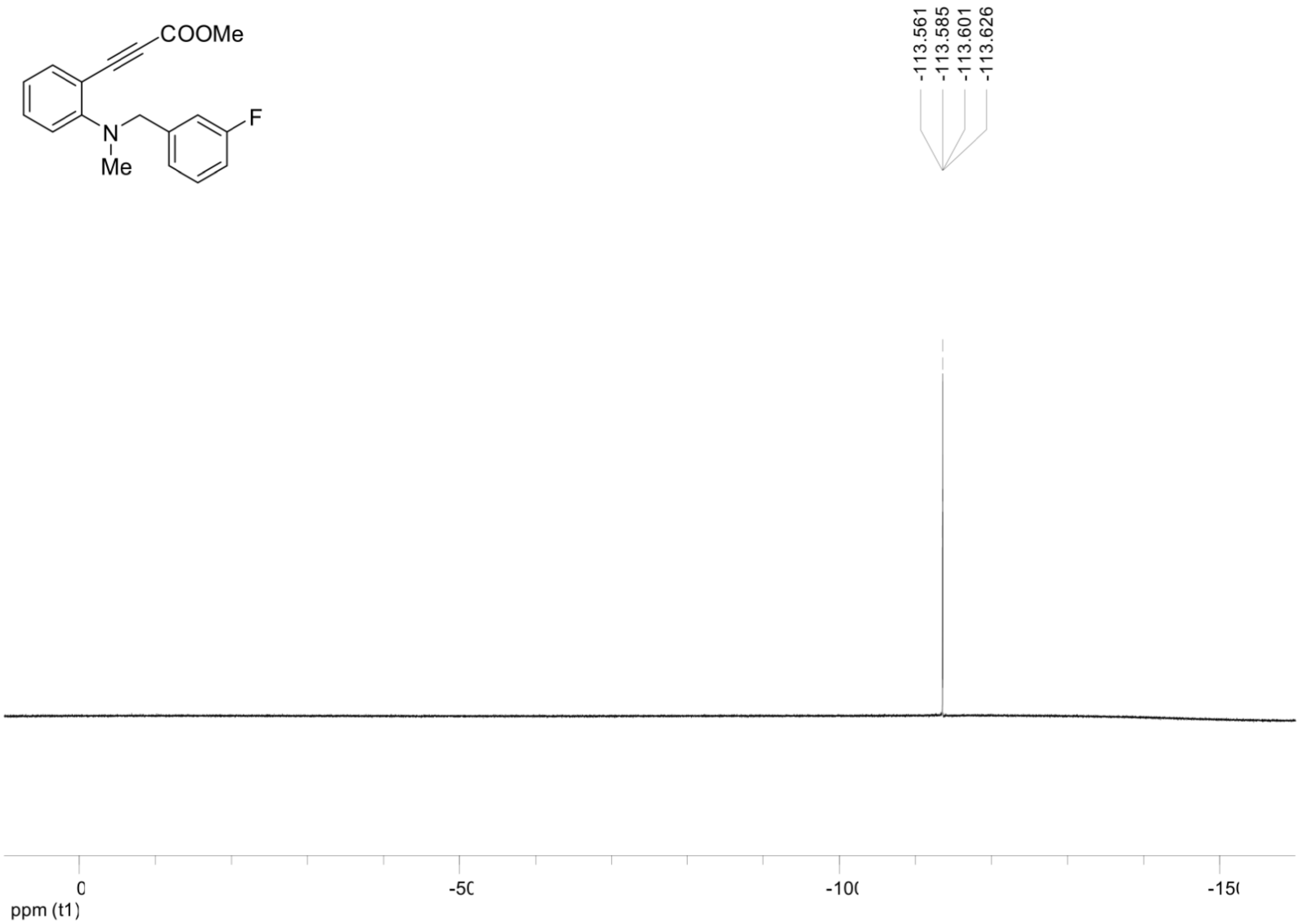

Supplementary Figure 17. ^{19}F NMR spectrum of **1d** (376 MHz, $CDCl_3$)

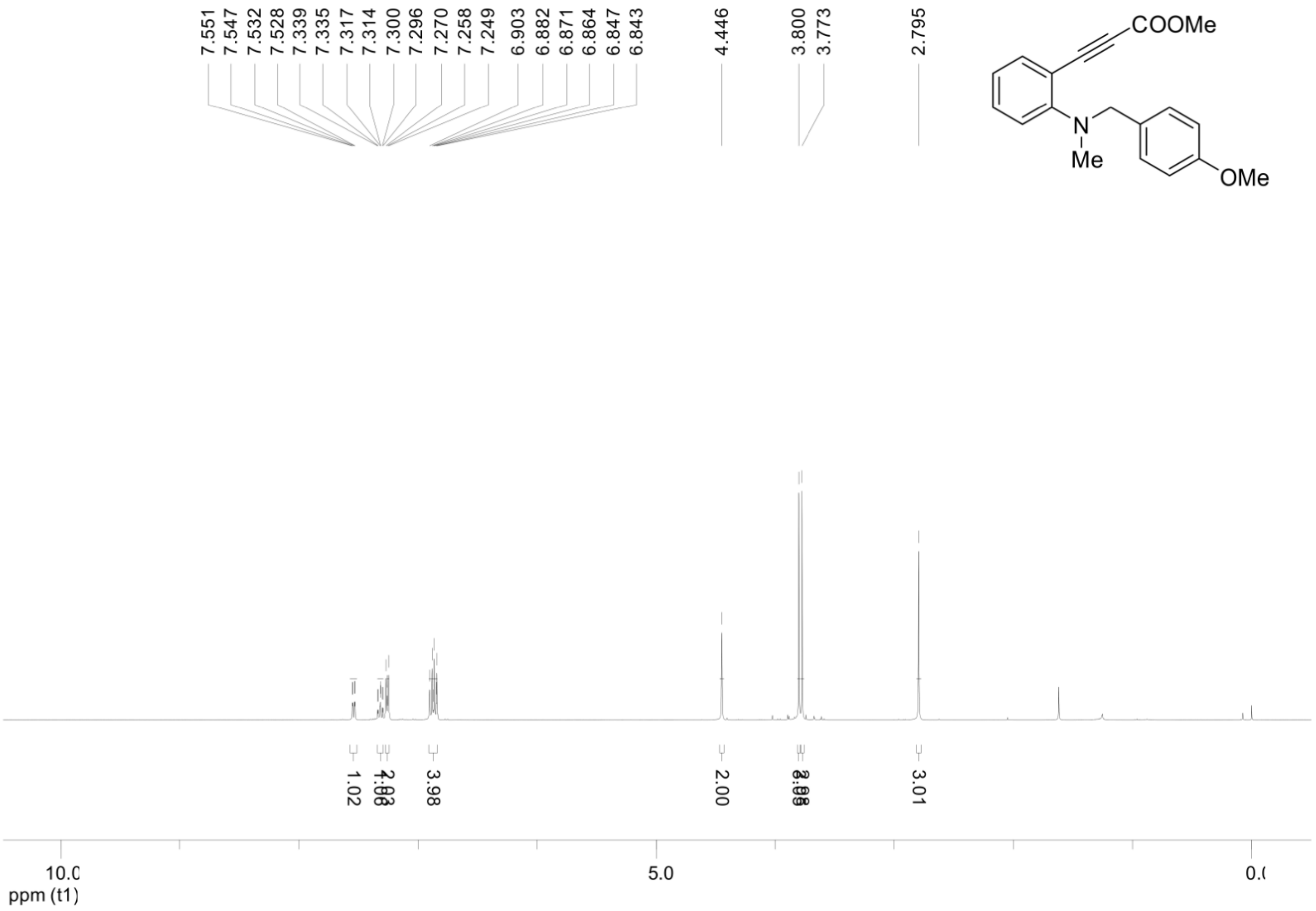

Supplementary Figure 18. ^{1}H NMR spectrum of **1e** (400 MHz, $CDCl_3$)

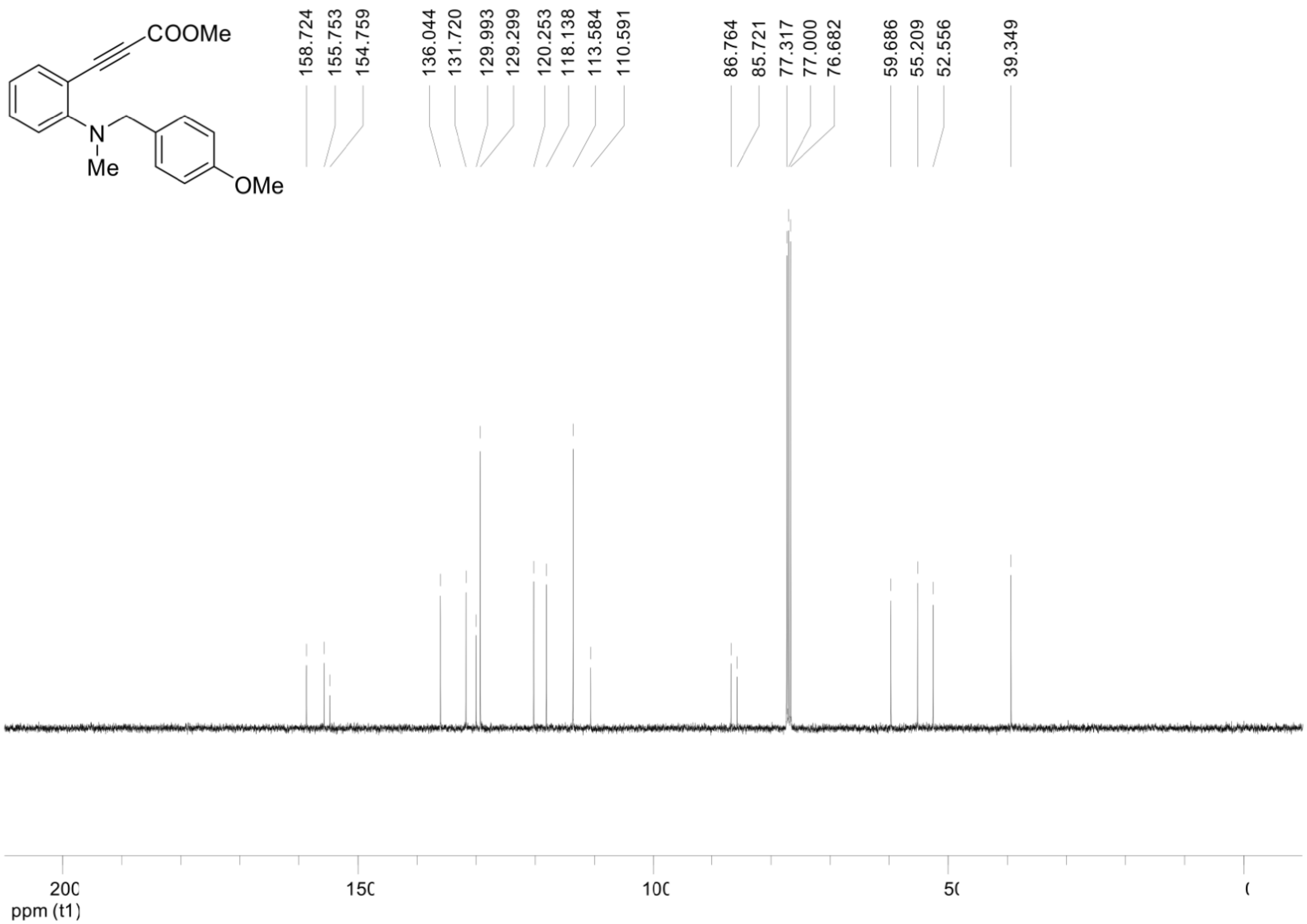

Supplementary Figure 19. ^{13}C NMR spectrum of **1e** (100 MHz, $CDCl_3$)

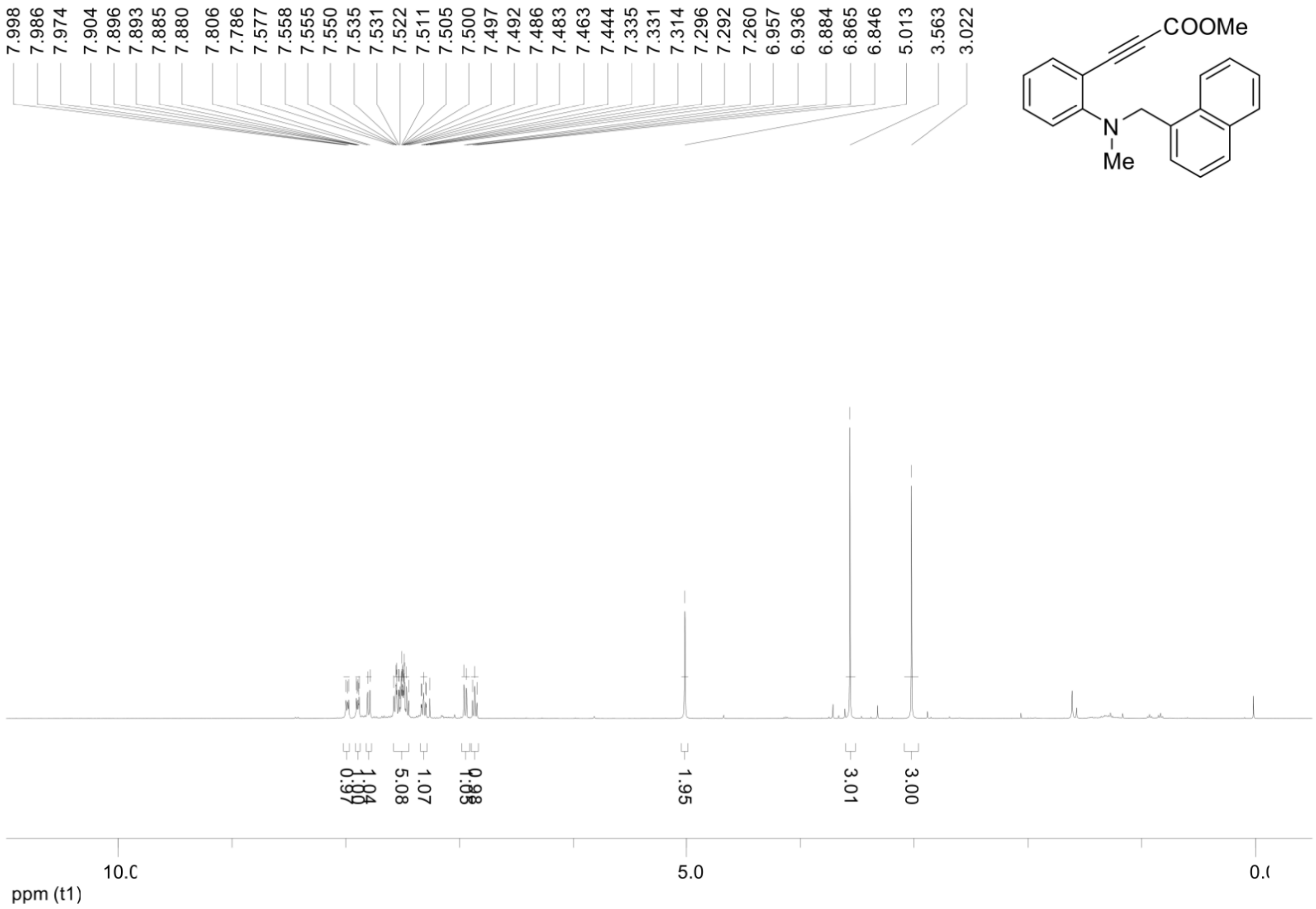

Supplementary Figure 20. ^{1}H NMR spectrum of **1f** (400 MHz, $CDCl_3$)

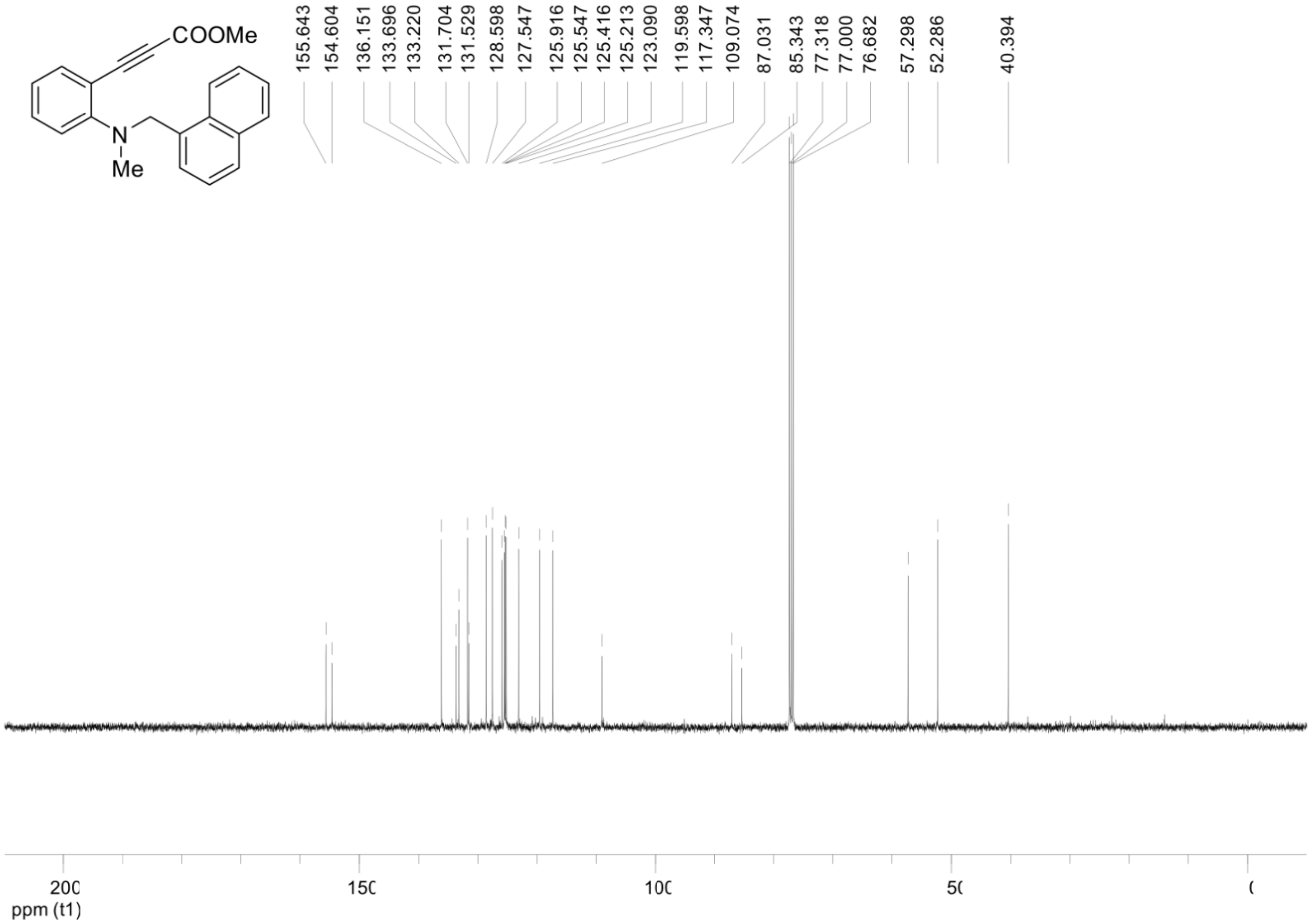

Supplementary Figure 21. ^{13}C NMR spectrum of **1f** (100 MHz, $CDCl_3$)

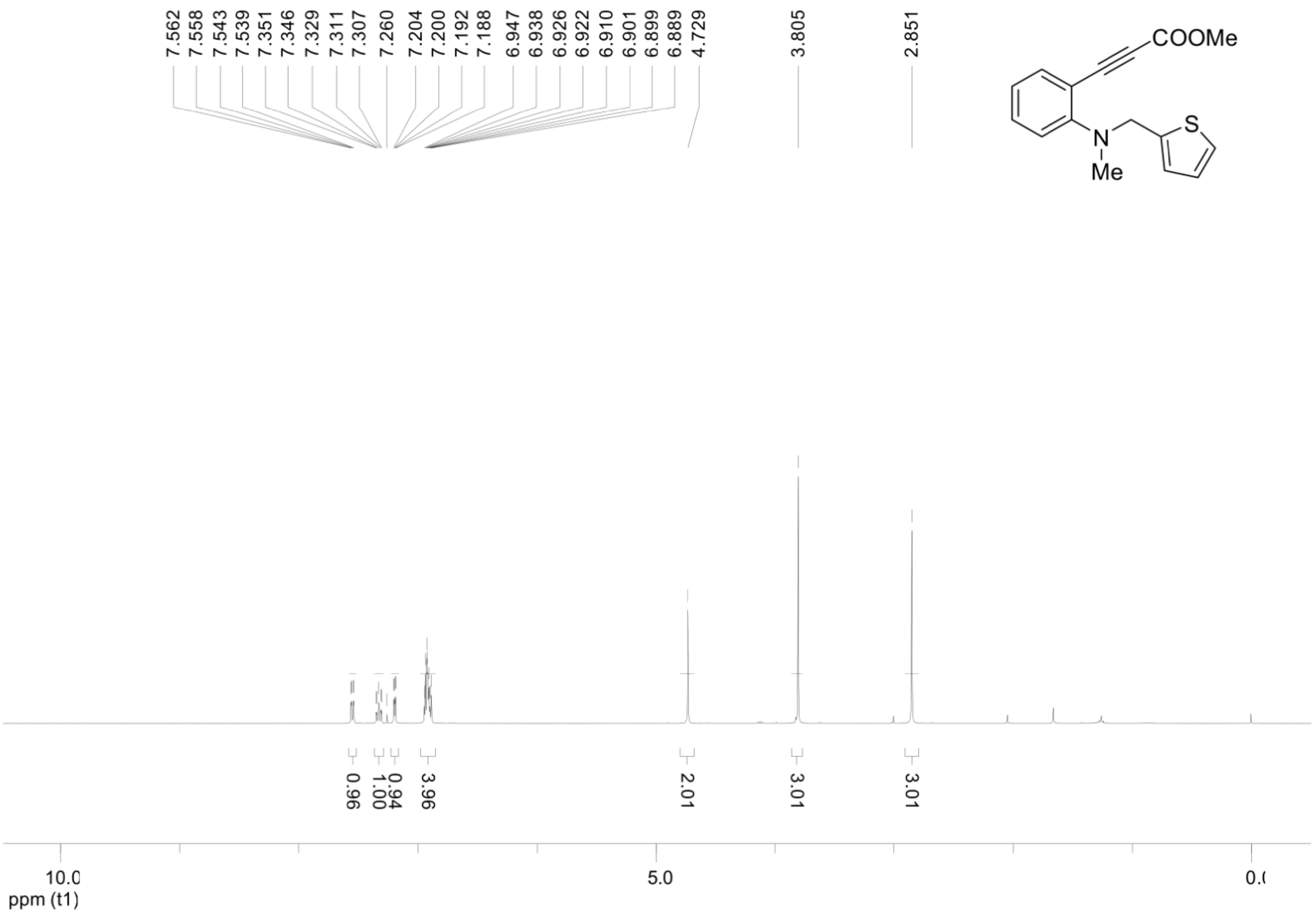

Supplementary Figure 22. ^{1}H NMR spectrum of **1g** (400 MHz, $CDCl_3$)

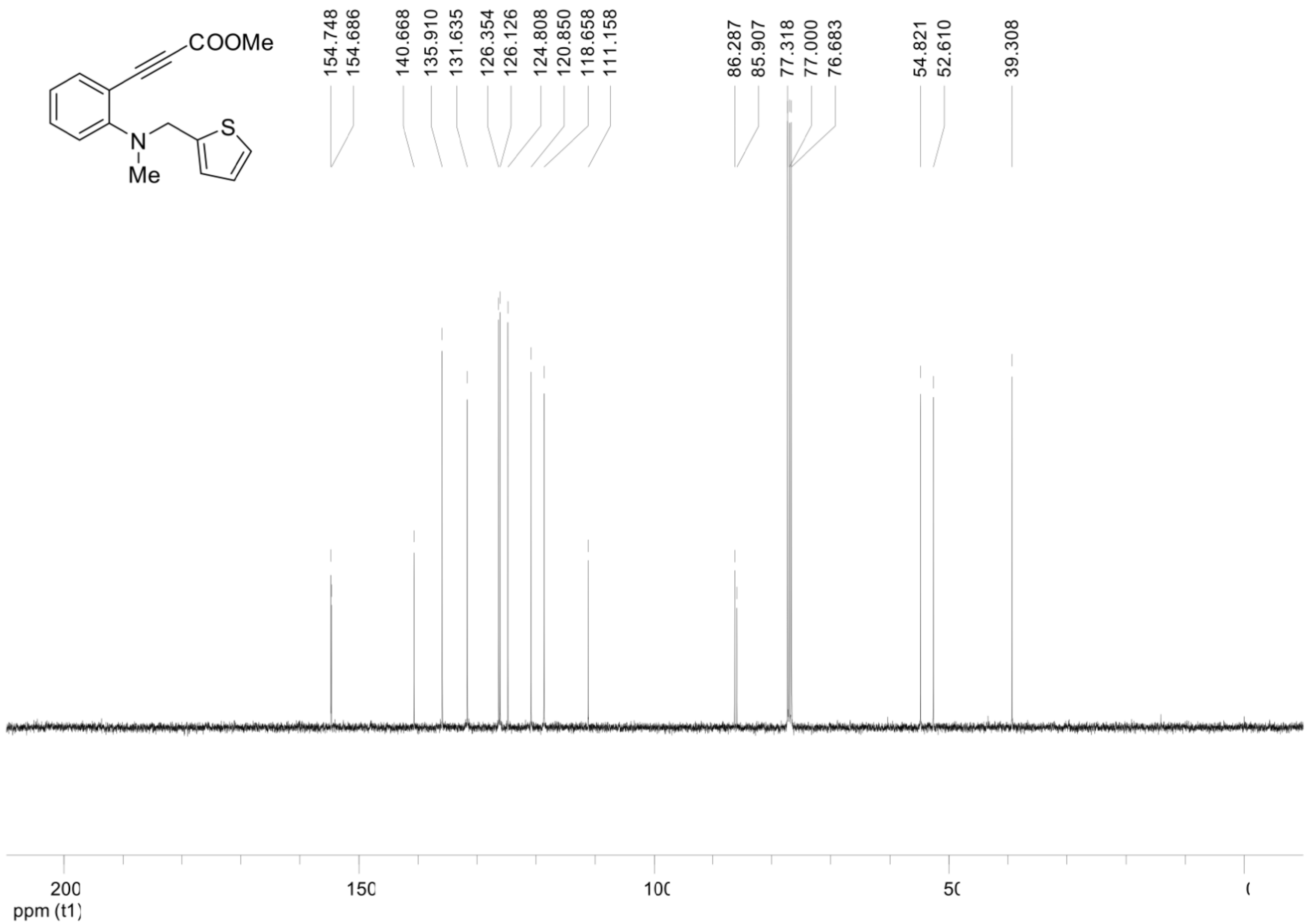

Supplementary Figure 23. ^{13}C NMR spectrum of **1g** (100 MHz, $CDCl_3$)

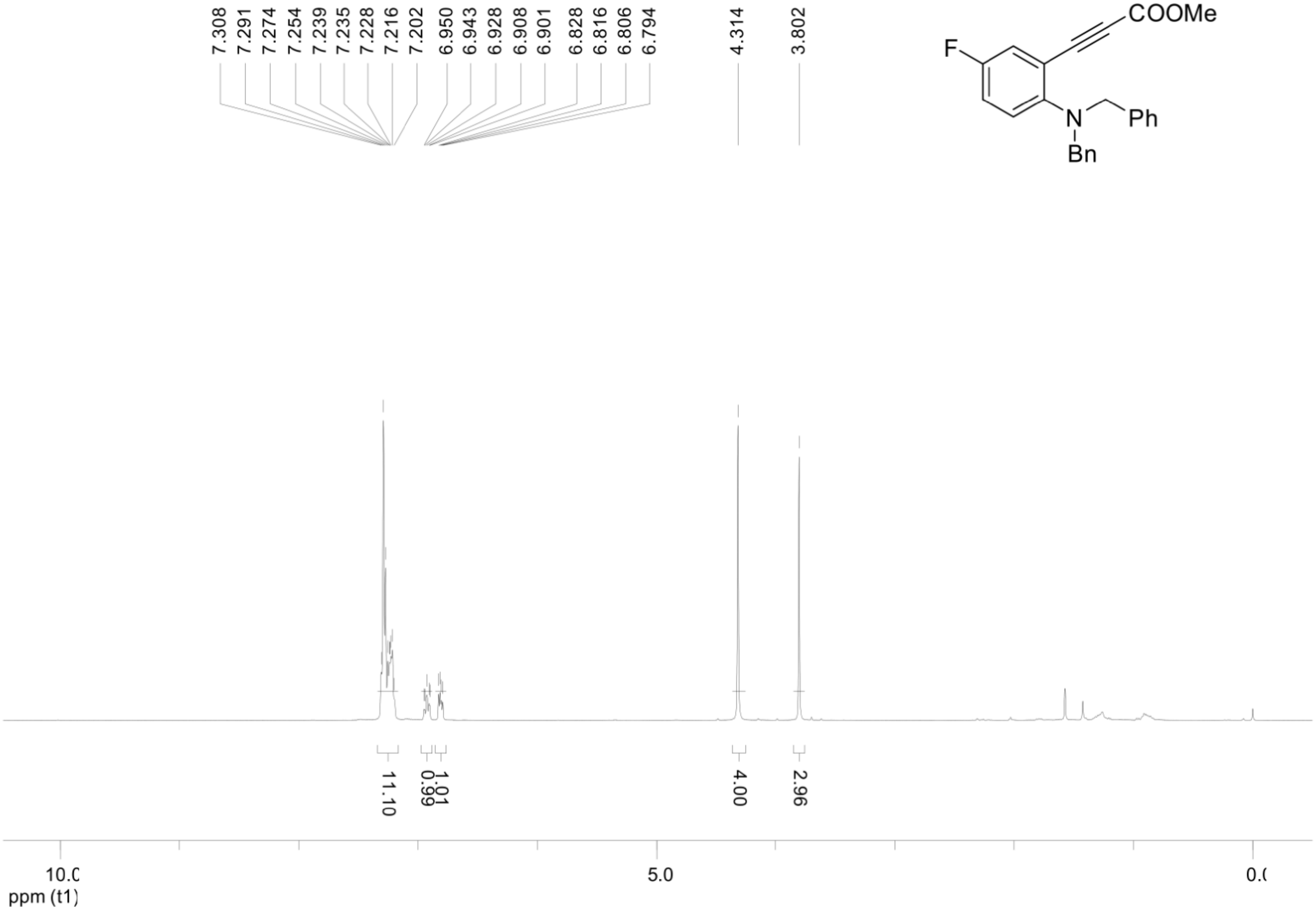

Supplementary Figure 24. ^{1}H NMR spectrum of **1h** (400 MHz, $CDCl_3$)

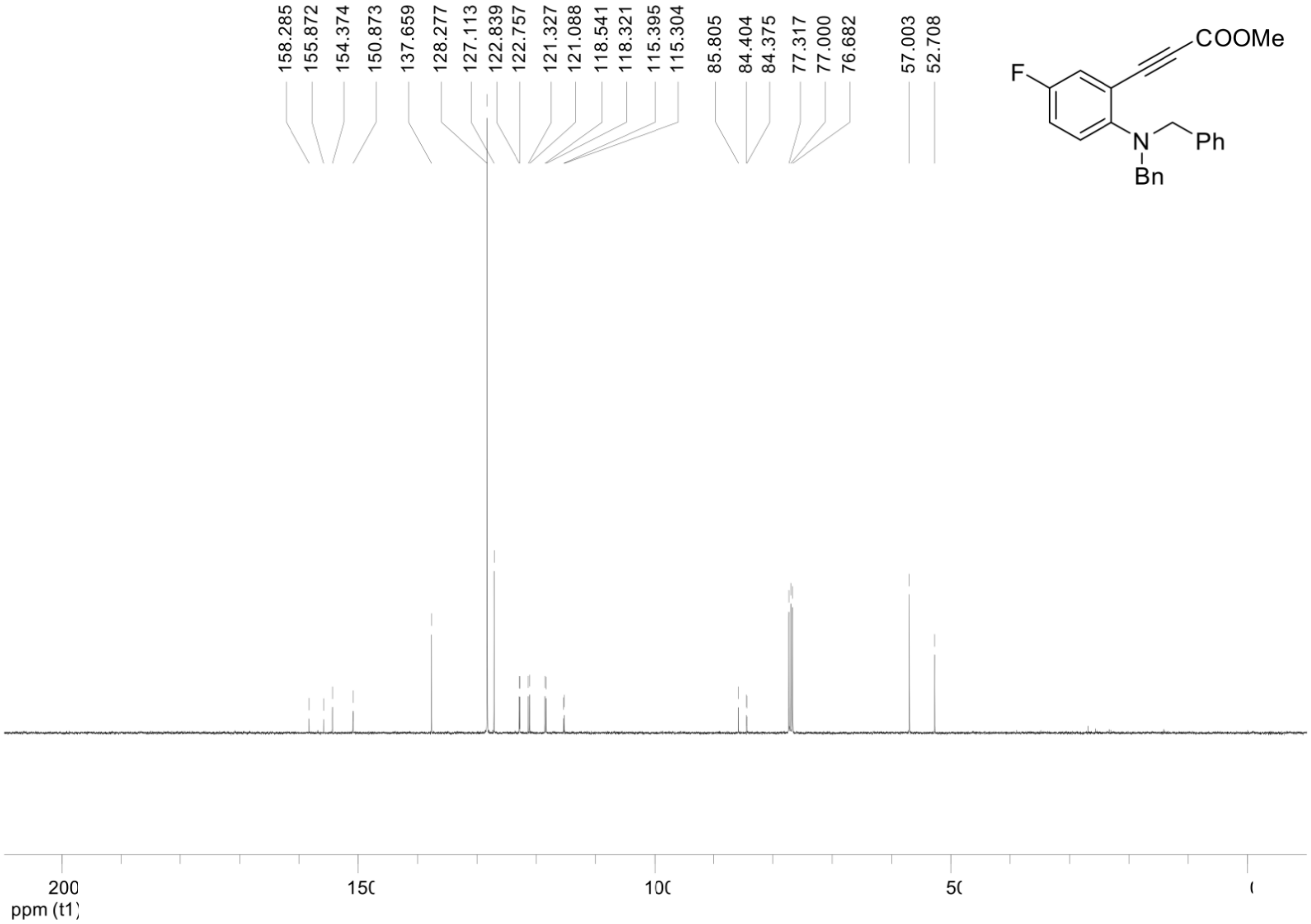

Supplementary Figure 25. ^{13}C NMR spectrum of **1h** (100 MHz, $CDCl_3$)

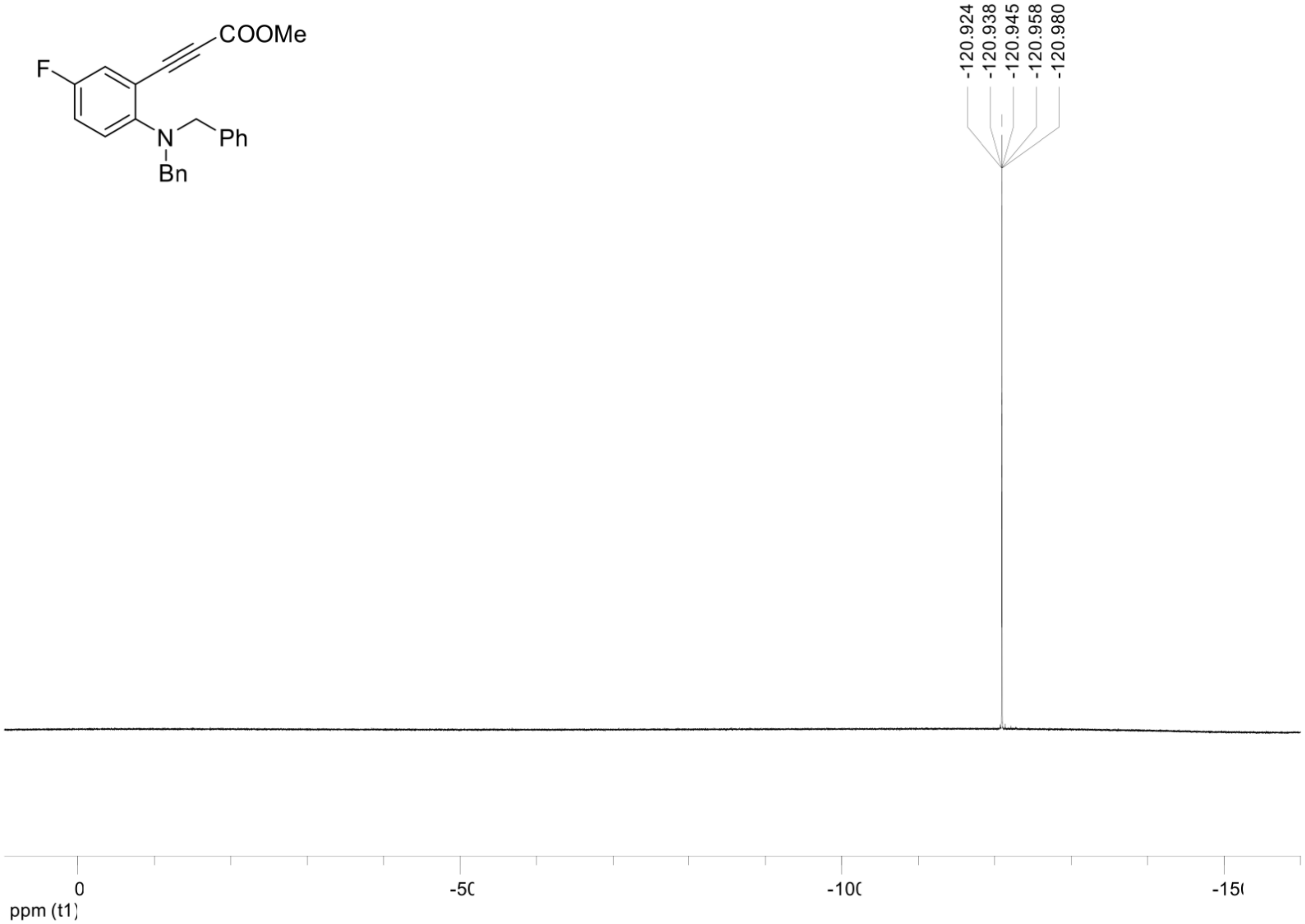

Supplementary Figure 26. ^{19}F NMR spectrum of **1h** (376 MHz, $CDCl_3$)

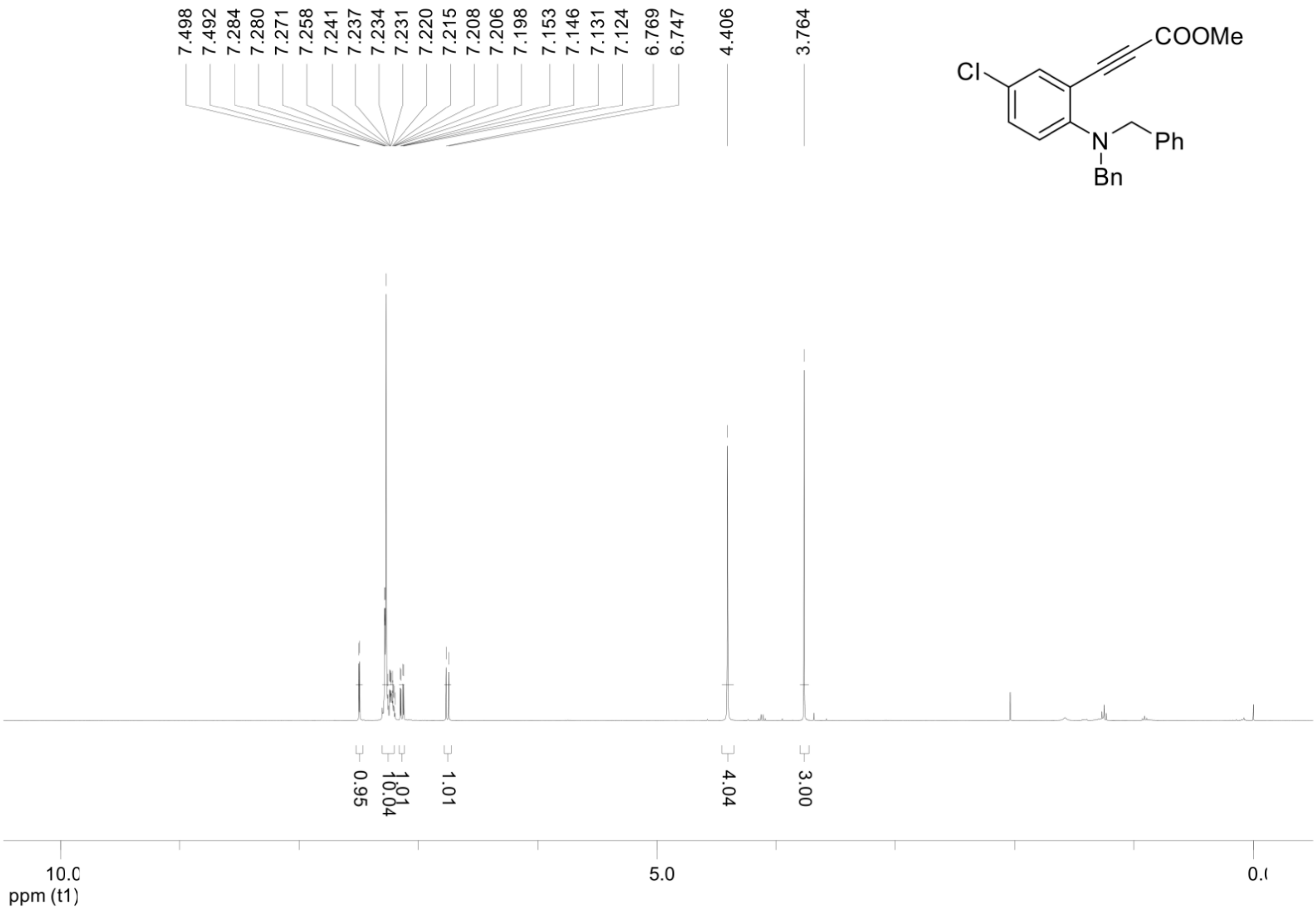

Supplementary Figure 27. ^{1}H NMR spectrum of **1i** (400 MHz, $CDCl_3$)

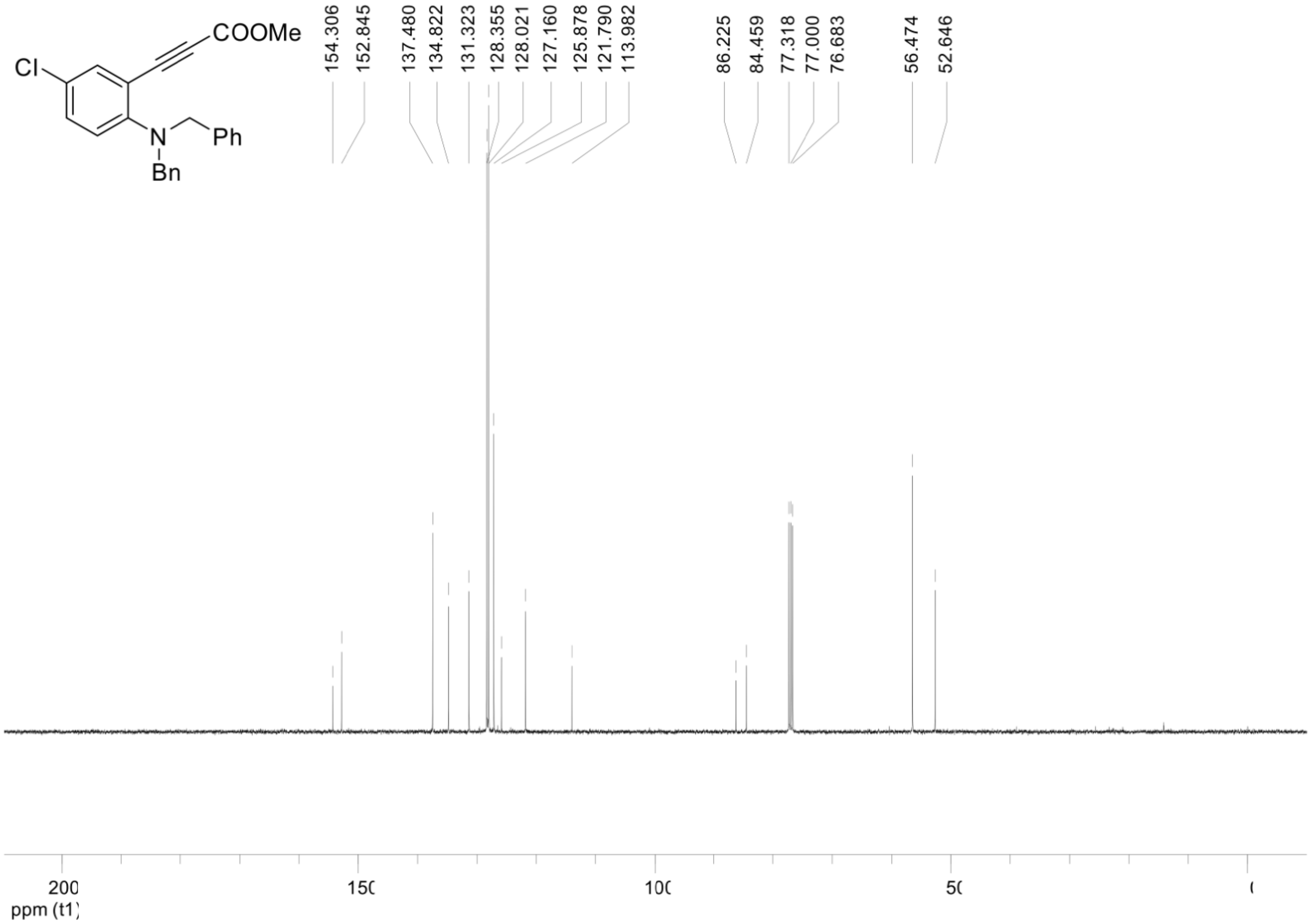

Supplementary Figure 28. ^{13}C NMR spectrum of **1i** (100 MHz, $CDCl_3$)

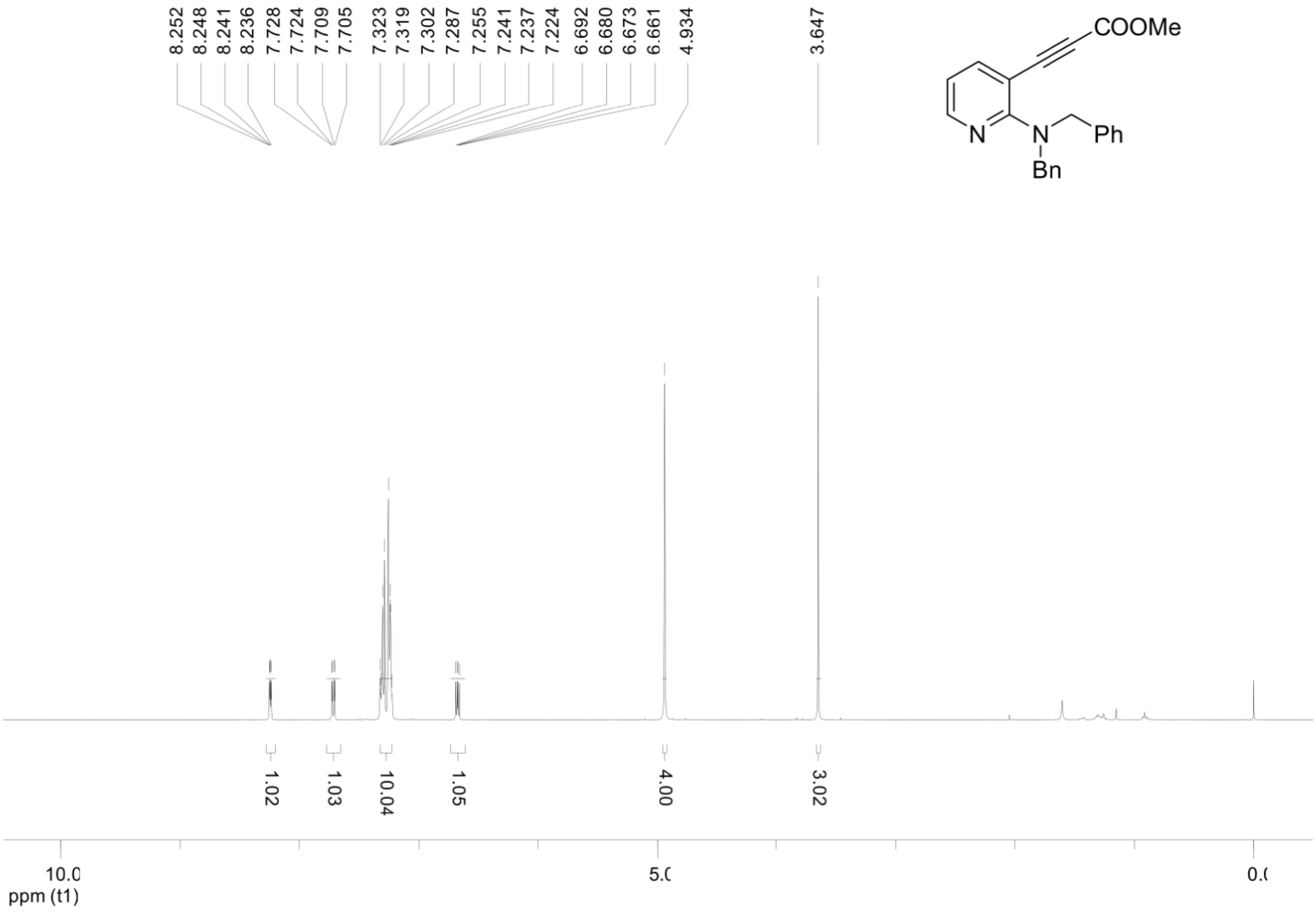

Supplementary Figure 29. ^{1}H NMR spectrum of **1j** (400 MHz, $CDCl_3$)

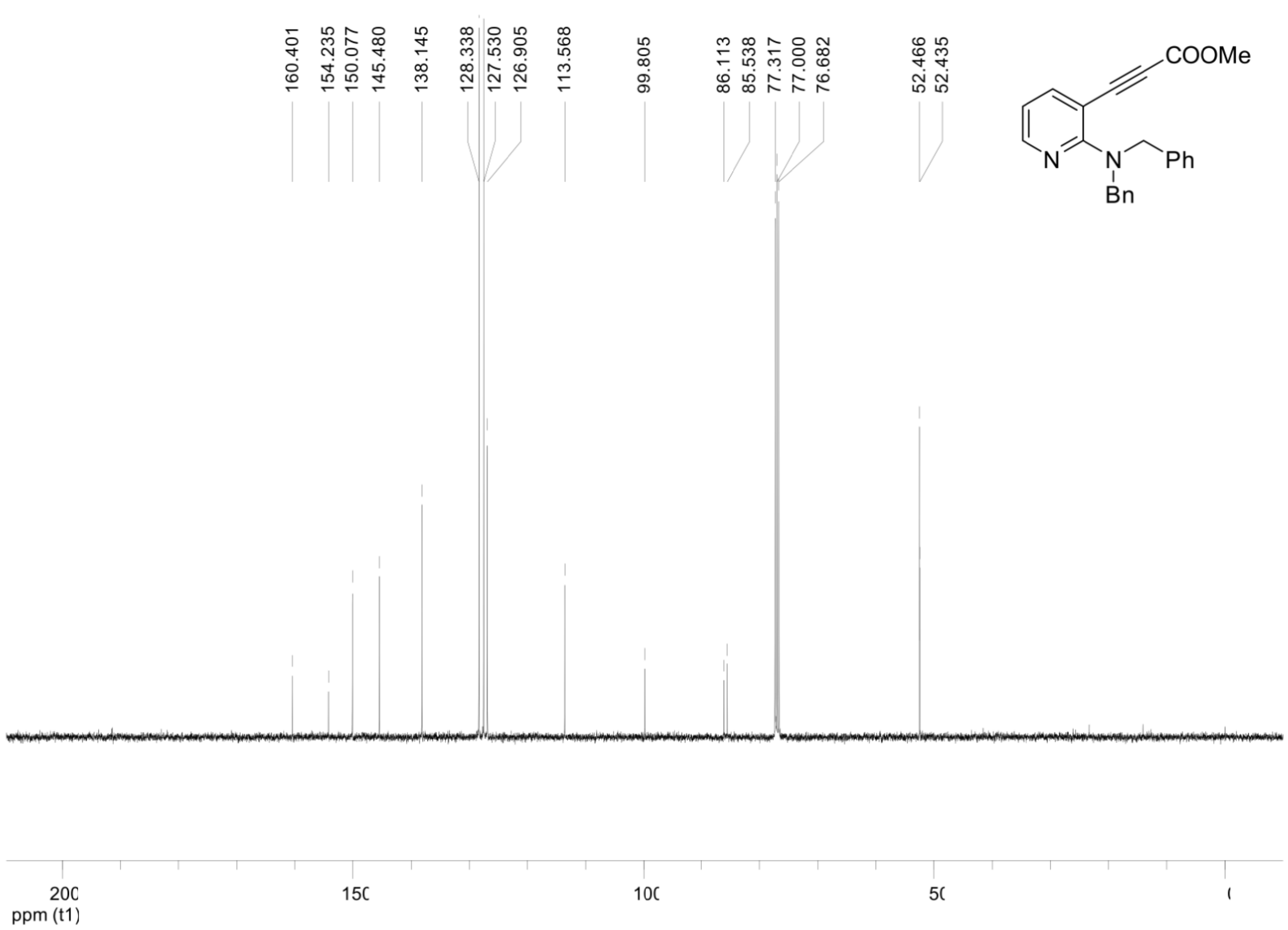

Supplementary Figure 30. ^{13}C NMR spectrum of **1j** (100 MHz, $CDCl_3$)

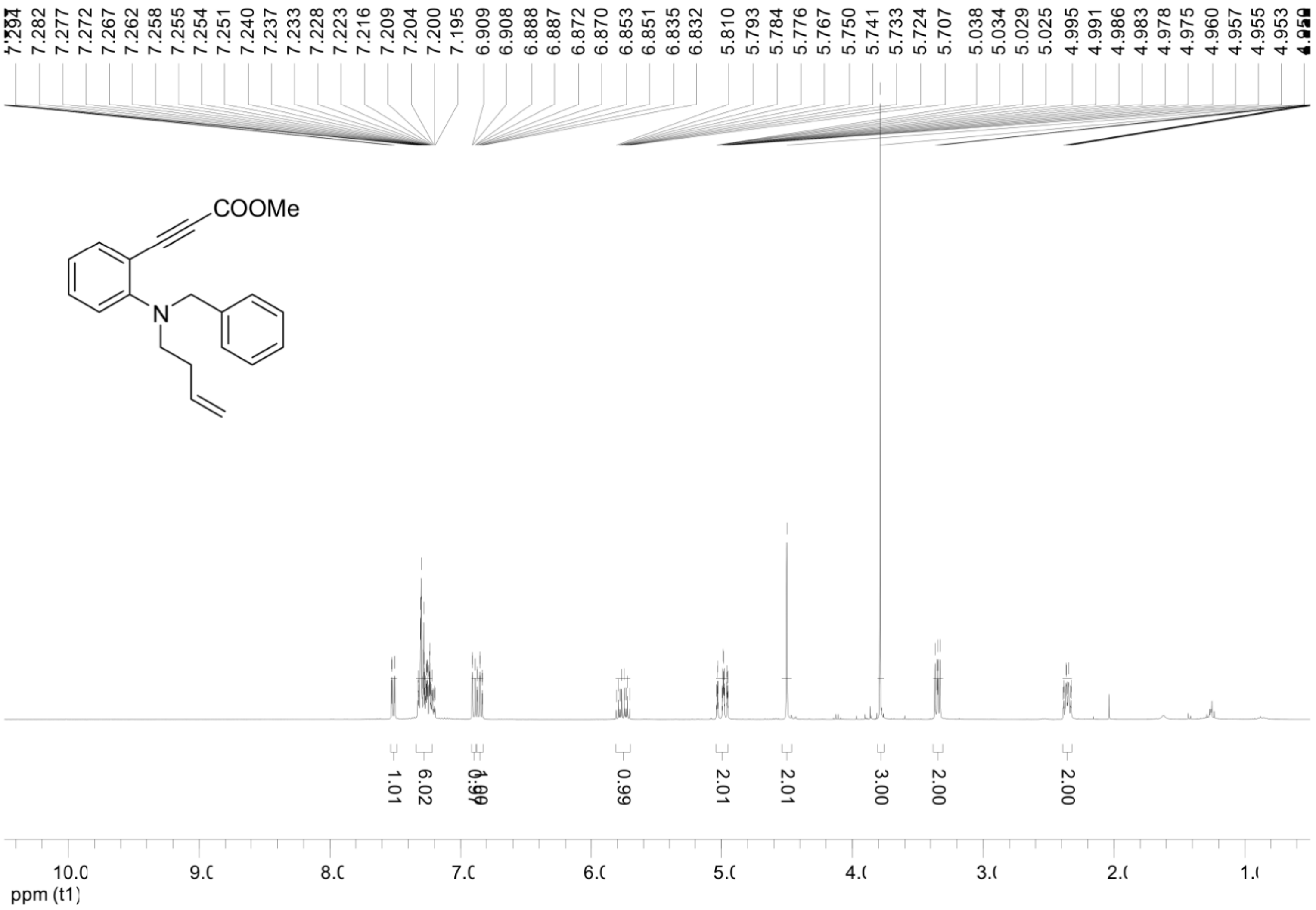

Supplementary Figure 31. ^{1}H NMR spectrum of **1k** (400 MHz, $CDCl_3$)

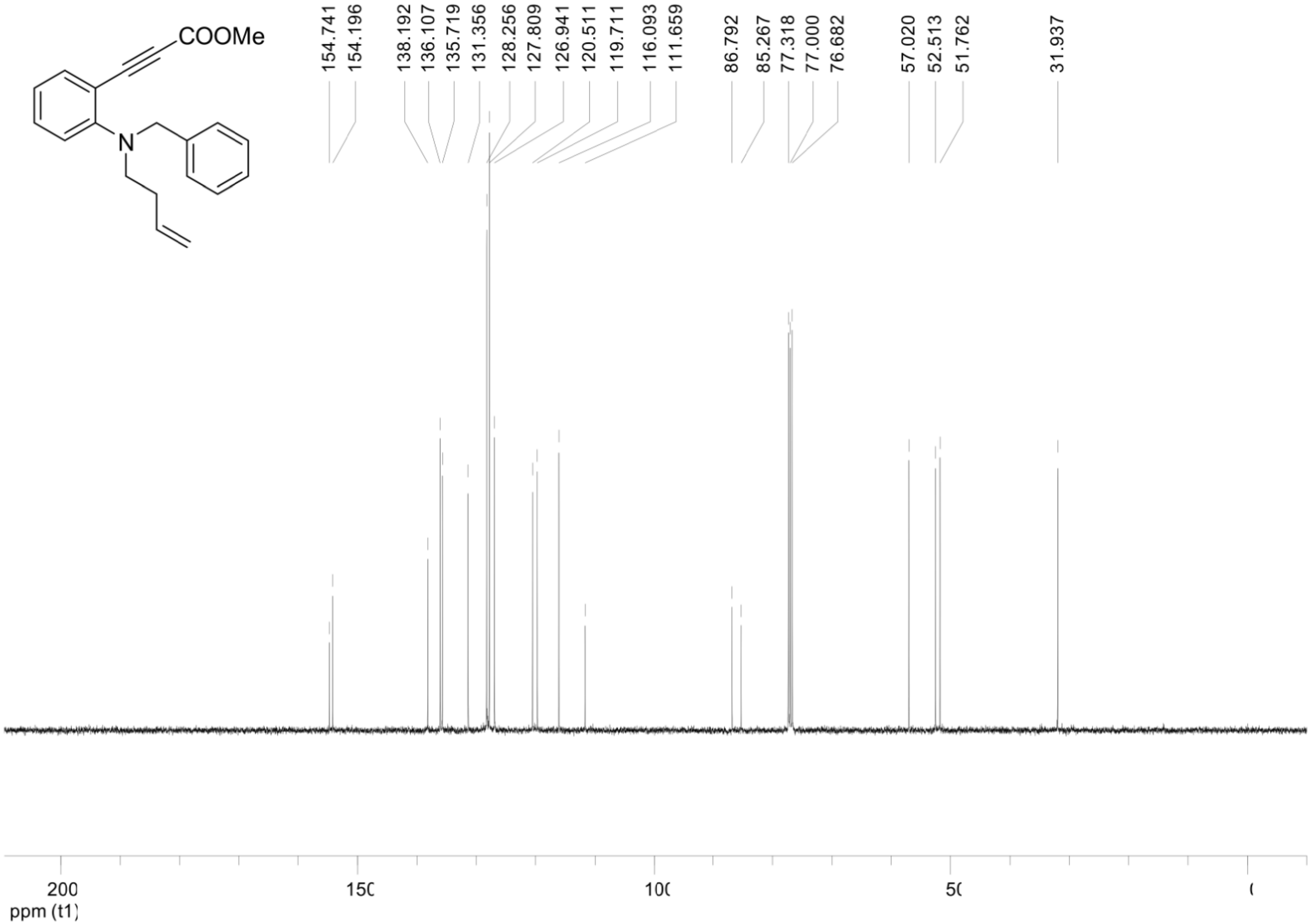

Supplementary Figure 32. ^{13}C NMR spectrum of **1k** (100 MHz, $CDCl_3$)

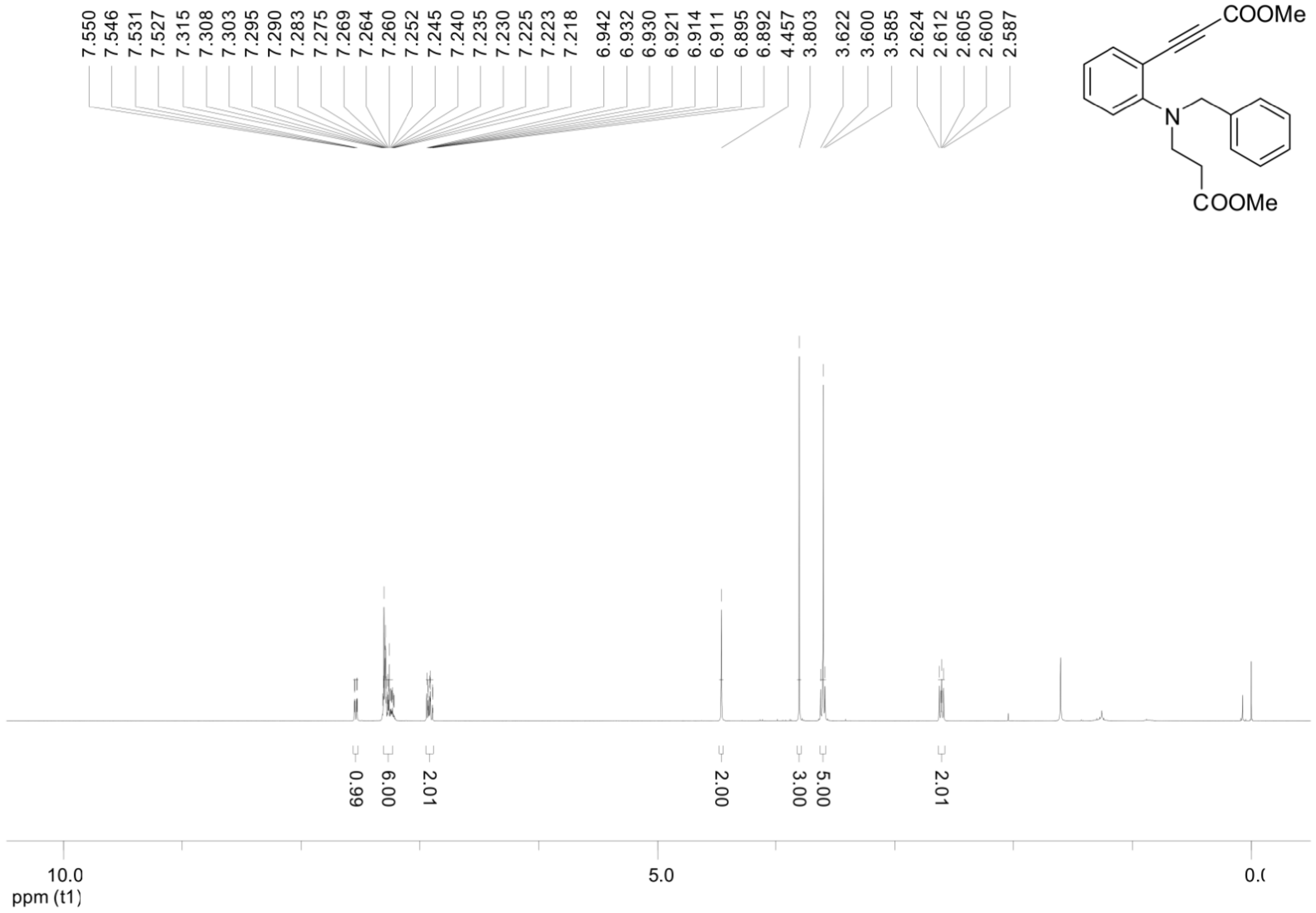

Supplementary Figure 33. ^{1}H NMR spectrum of **1l** (400 MHz, $CDCl_3$)

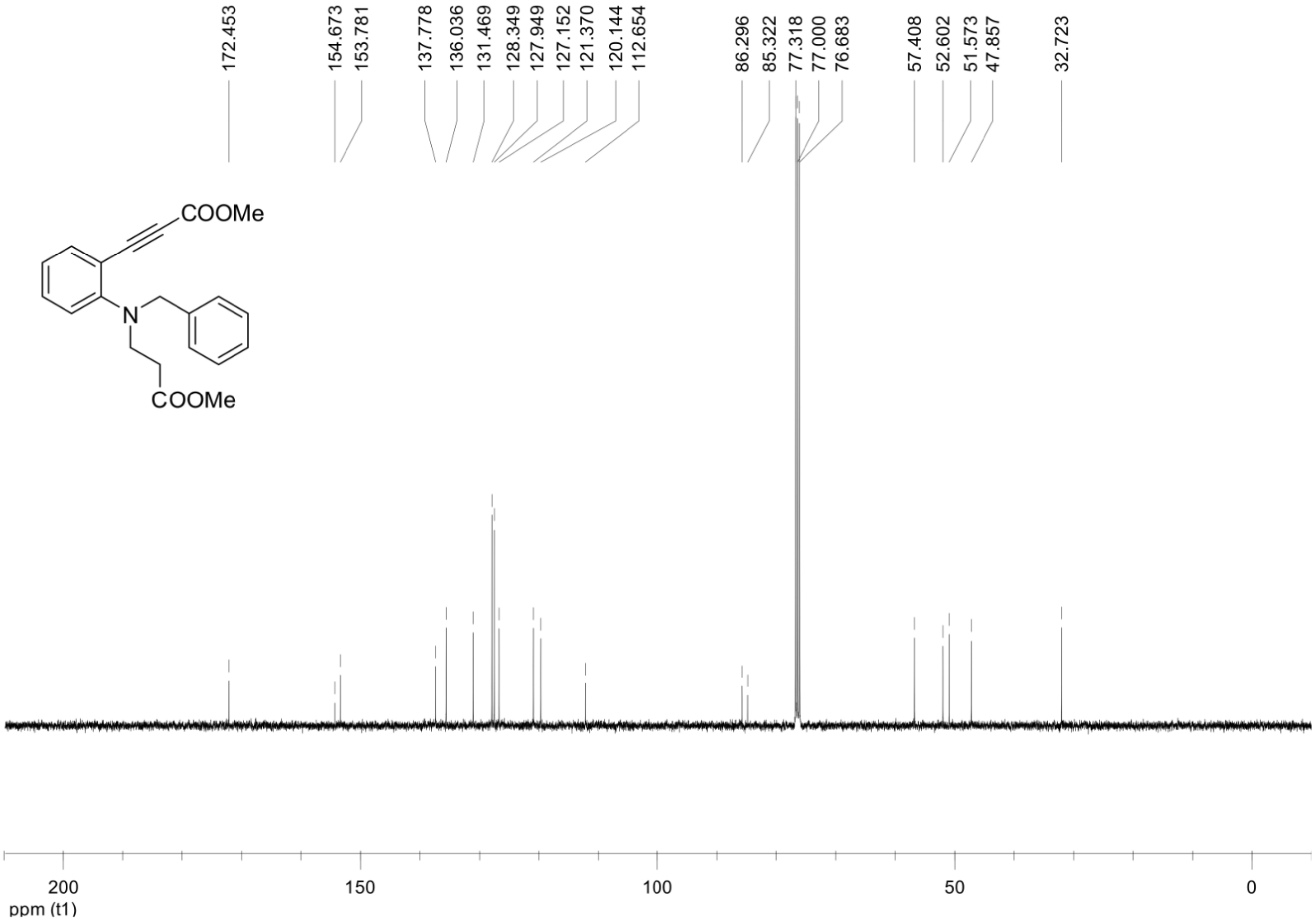

Supplementary Figure 34. ^{13}C NMR spectrum of **1l** (100 MHz, $CDCl_3$)

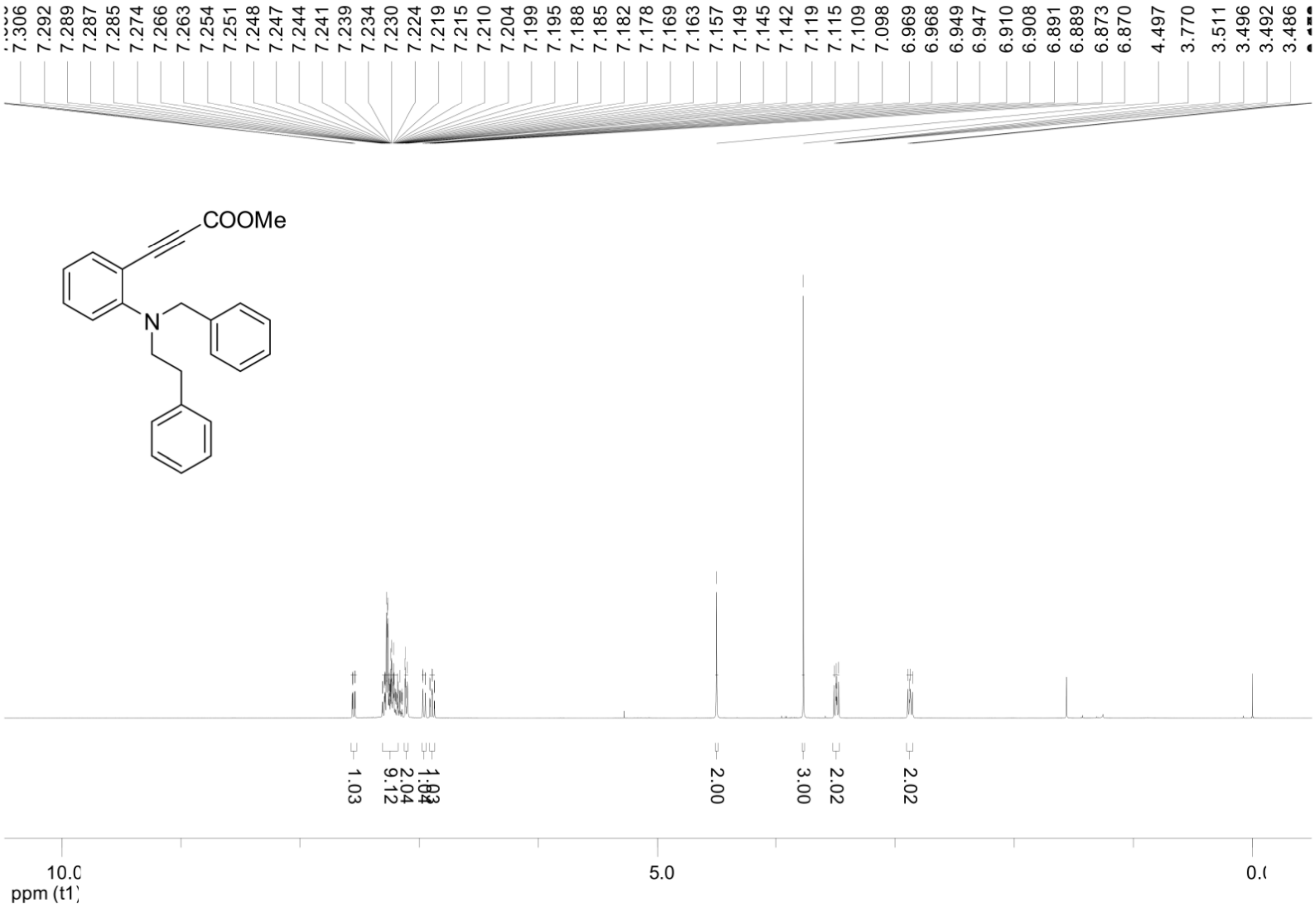

Supplementary Figure 35. ^{1}H NMR spectrum of **1m** (400 MHz, $CDCl_3$)

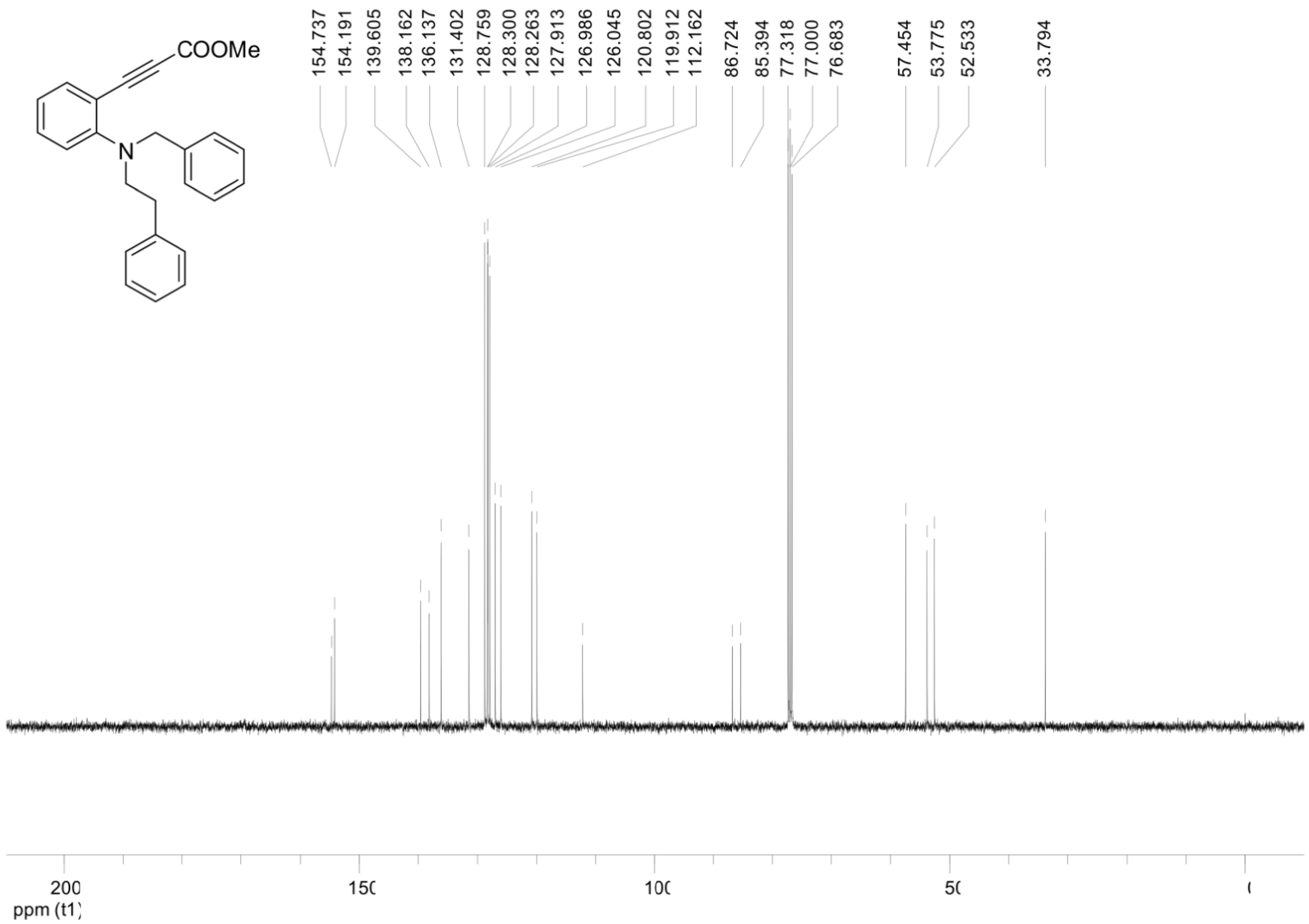

Supplementary Figure 36. ^{13}C NMR spectrum of **1m** (100 MHz, $CDCl_3$)

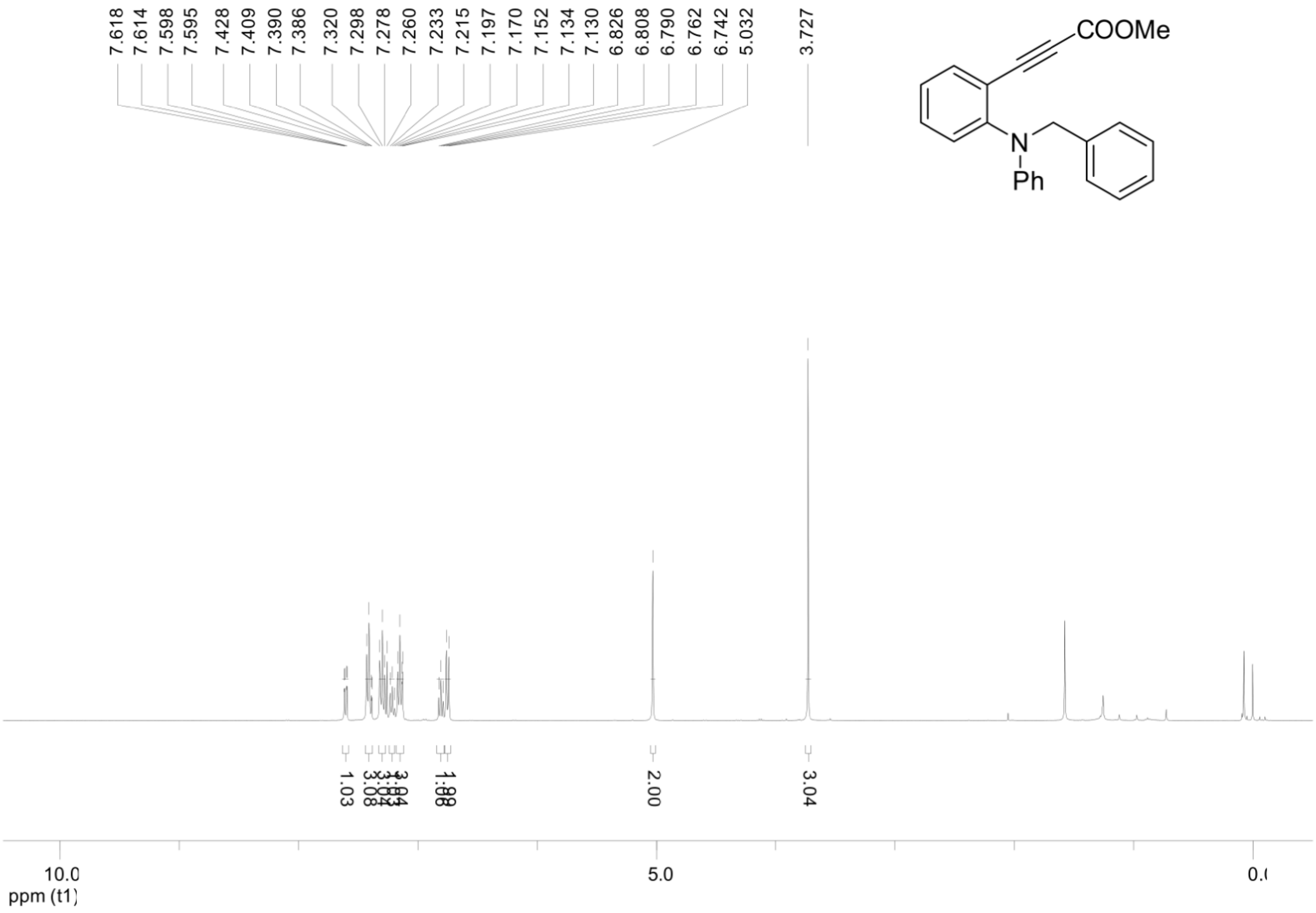

Supplementary Figure 37. ^{1}H NMR spectrum of **1n** (400 MHz, $CDCl_3$)

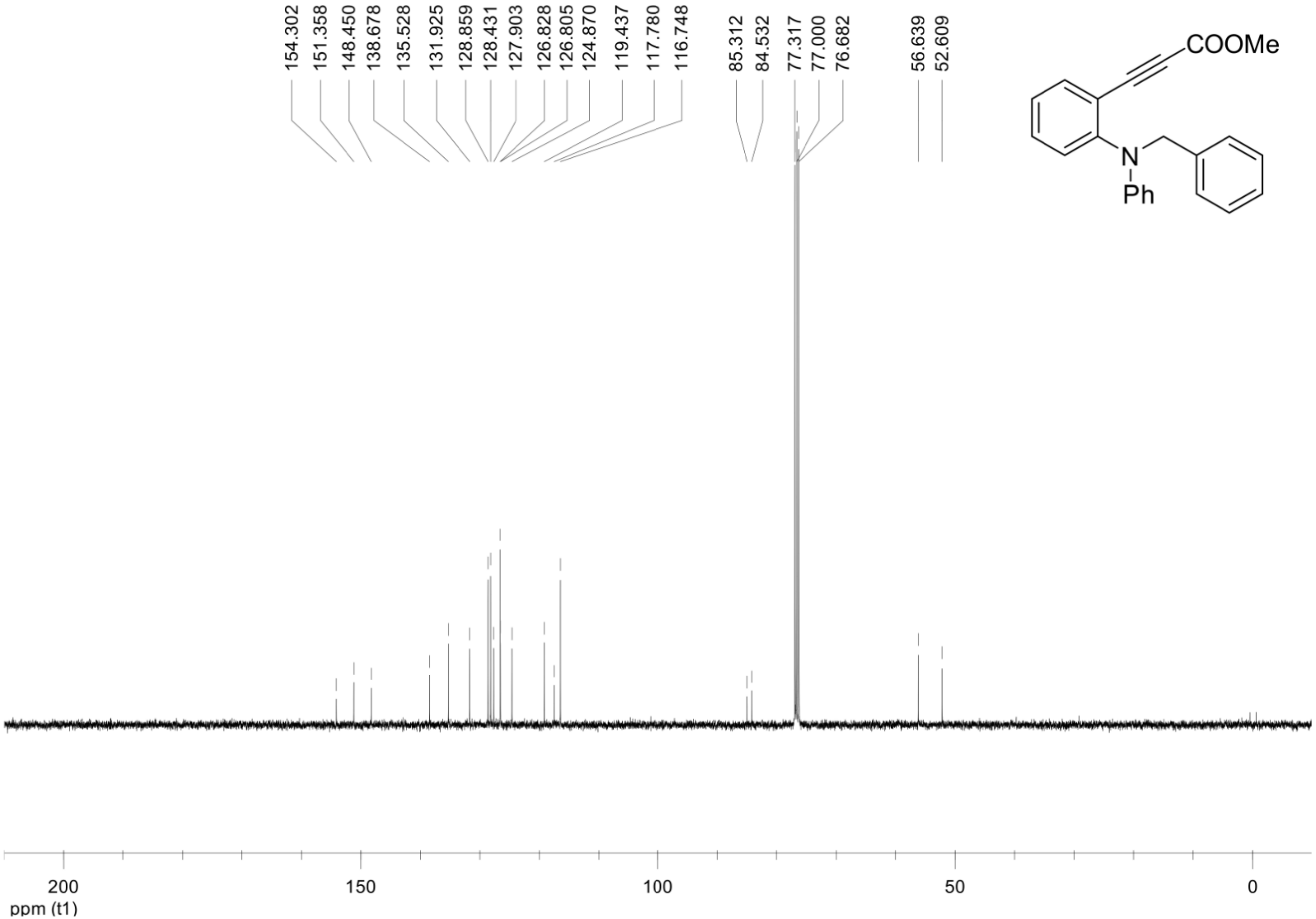

Supplementary Figure 38. ^{13}C NMR spectrum of **1n** (100 MHz, $CDCl_3$)

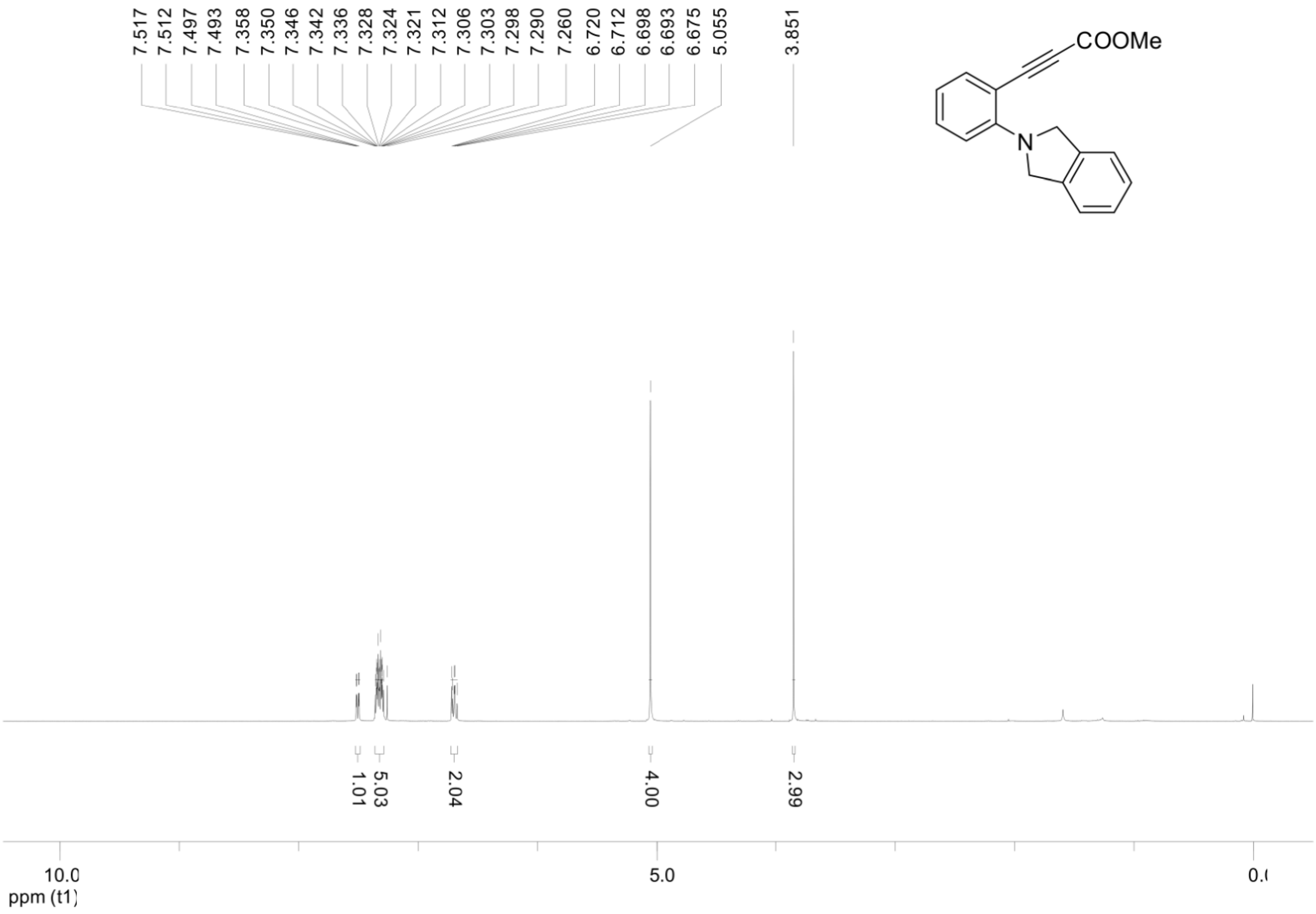

Supplementary Figure 39. ^{1}H NMR spectrum of **1p** (400 MHz, $CDCl_3$)

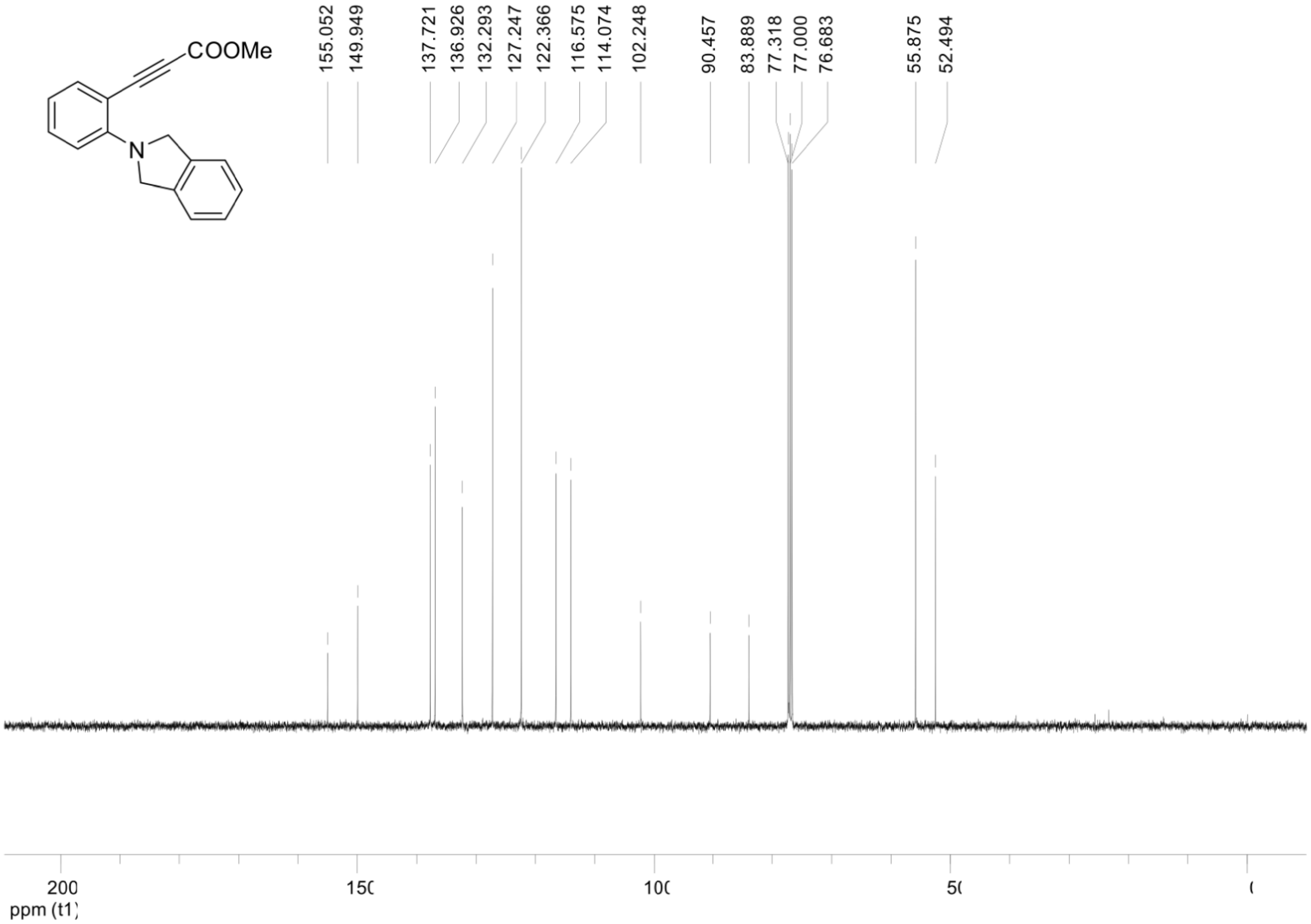

Supplementary Figure 40. ^{13}C NMR spectrum of **1p** (100 MHz, $CDCl_3$)

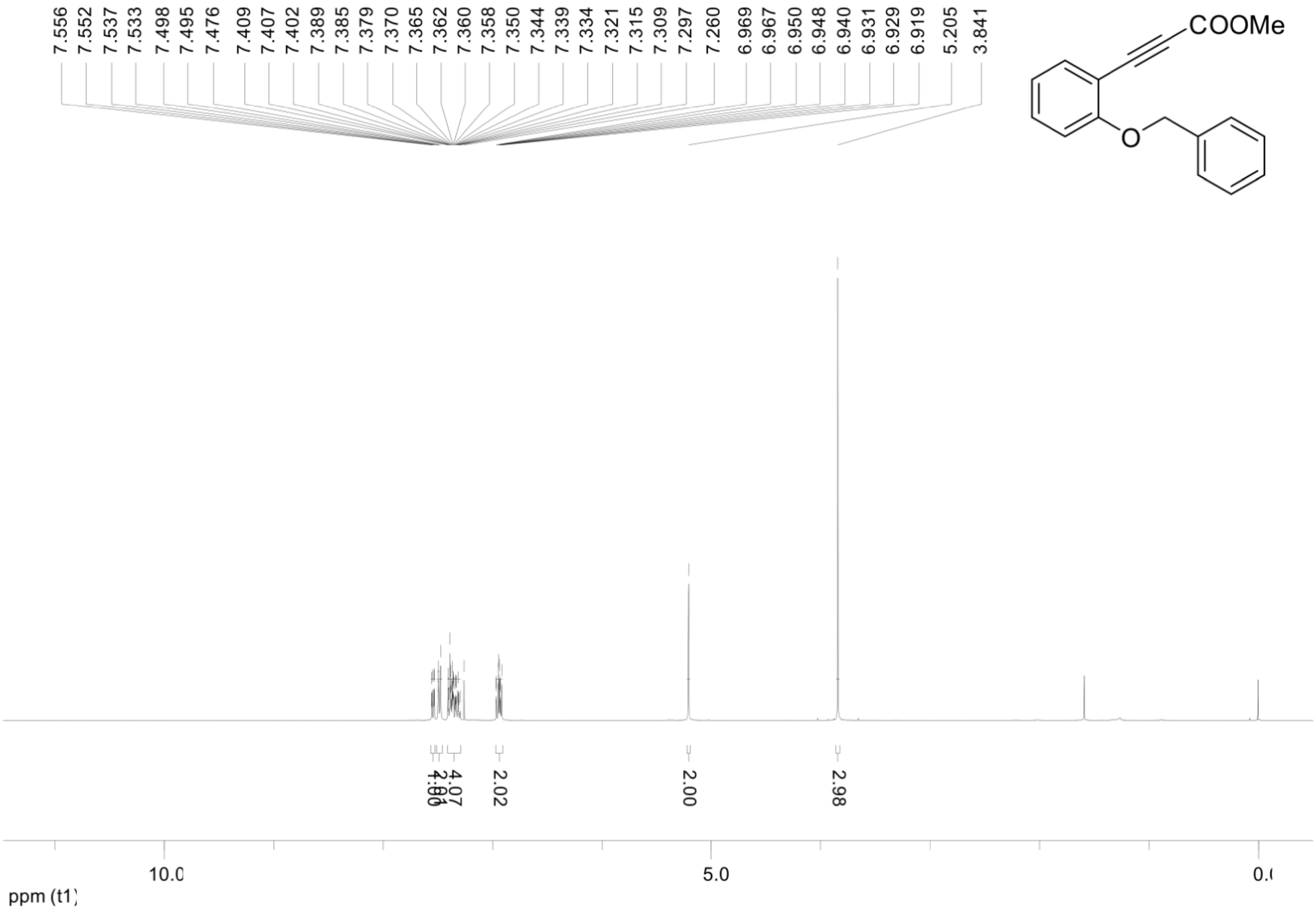

Supplementary Figure 41. ^{1}H NMR spectrum of **1q** (400 MHz, $CDCl_3$)

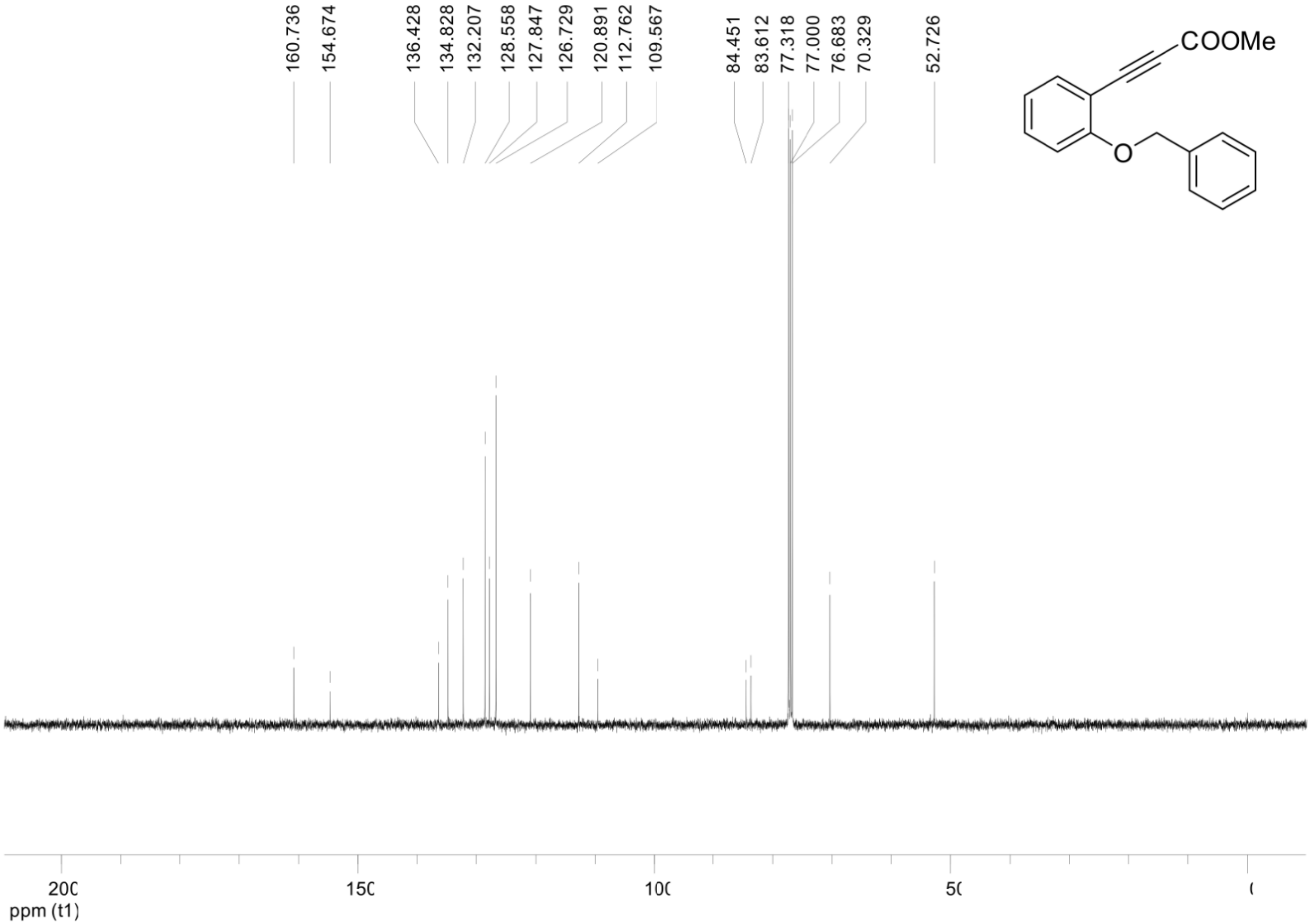

Supplementary Figure 42. ^{13}C NMR spectrum of **1q** (100 MHz, $CDCl_3$)

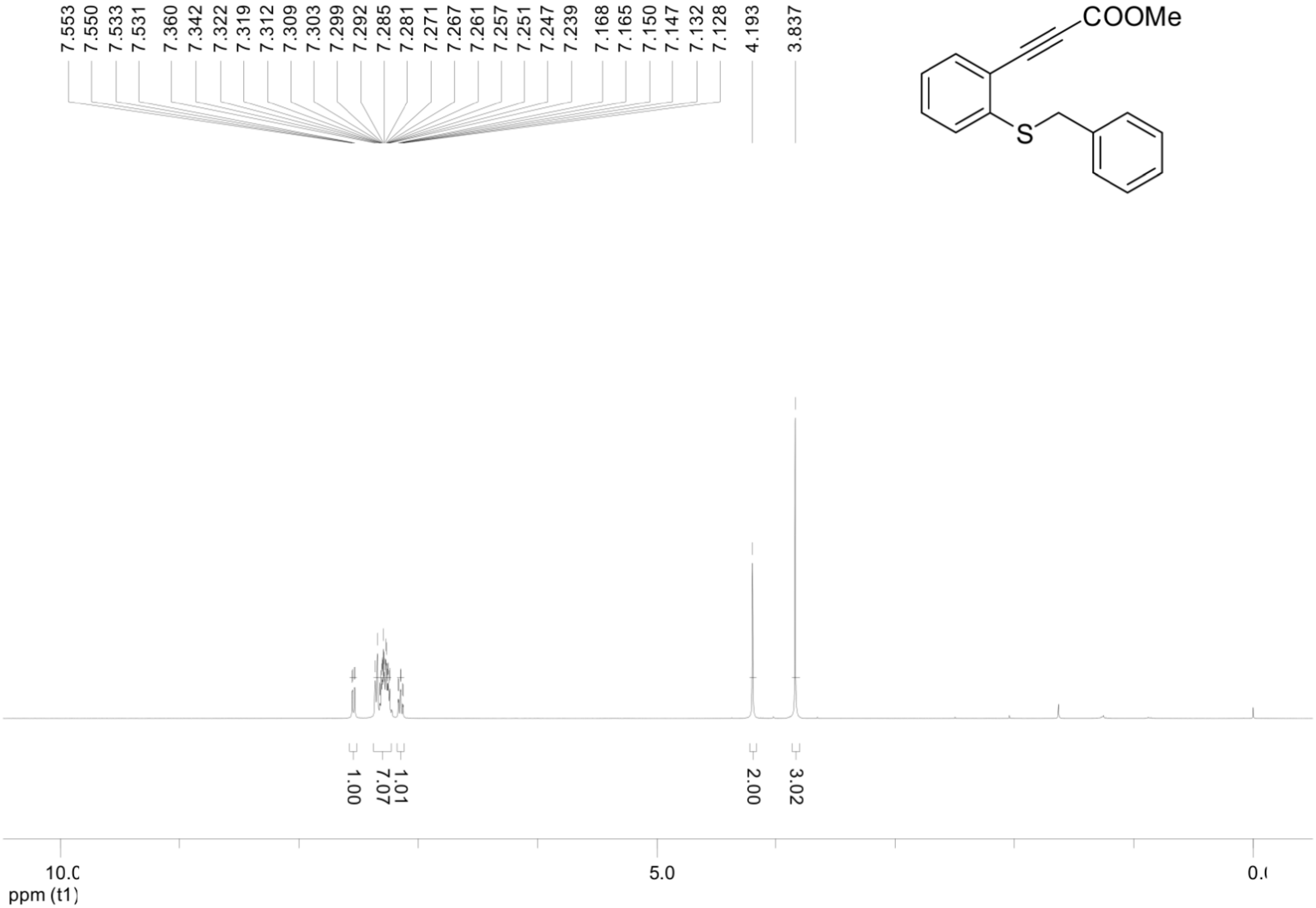

Supplementary Figure 43. ^{1}H NMR spectrum of **1r** (400 MHz, $CDCl_3$)

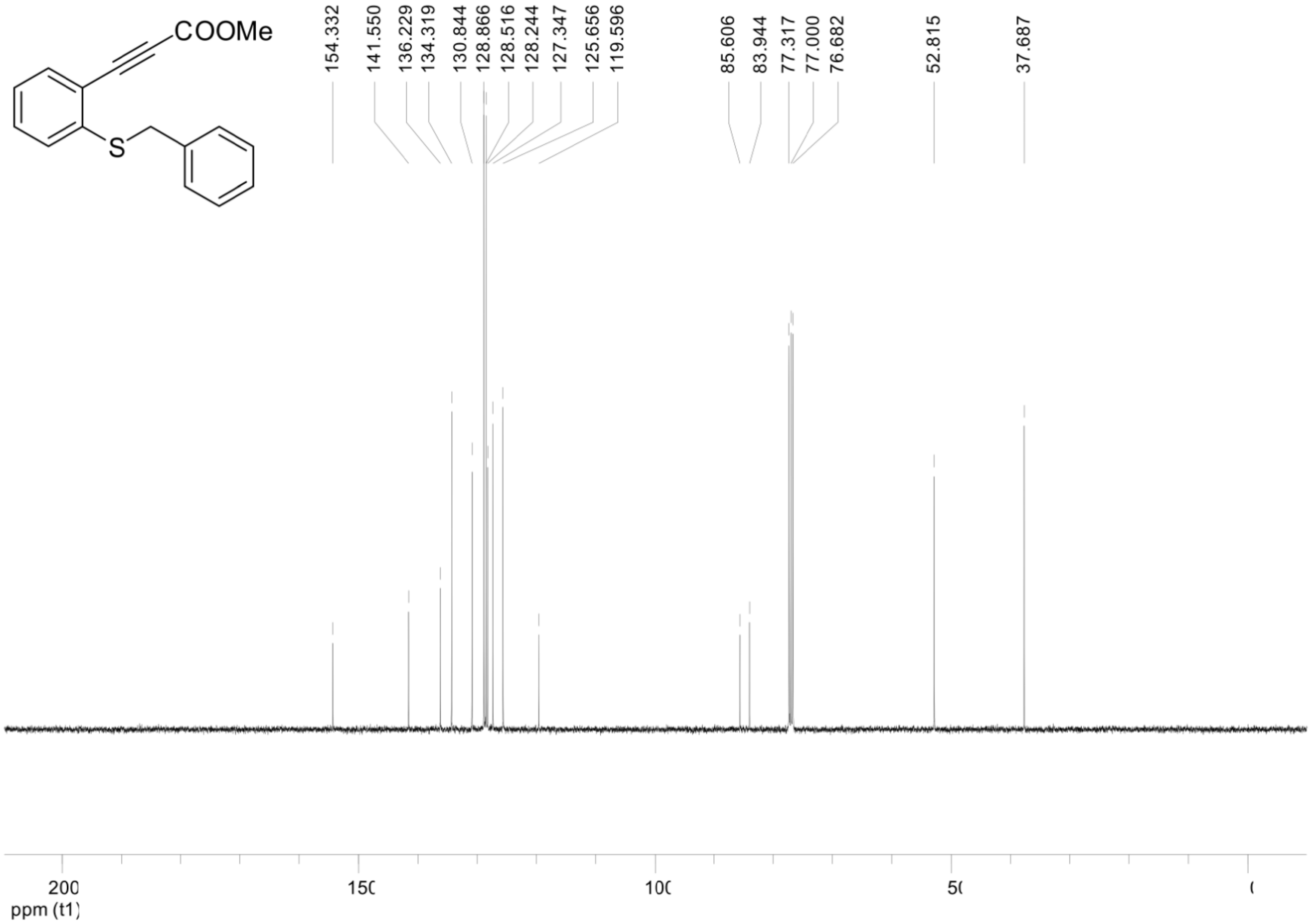

Supplementary Figure 44. ^{13}C NMR spectrum of **1r** (100 MHz, $CDCl_3$)

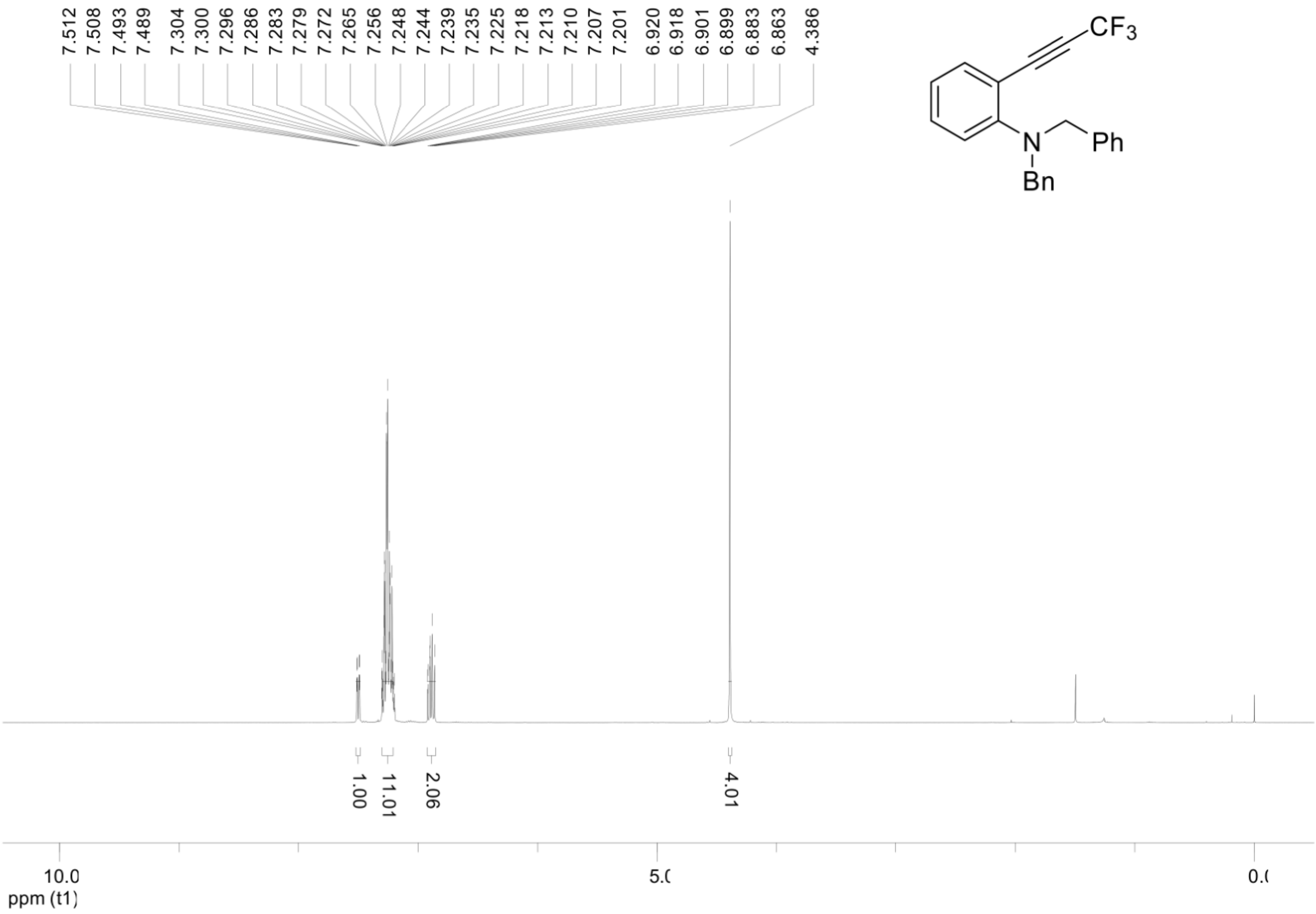

Supplementary Figure 45. ^{1}H NMR spectrum of **1s** (400 MHz, $CDCl_3$)

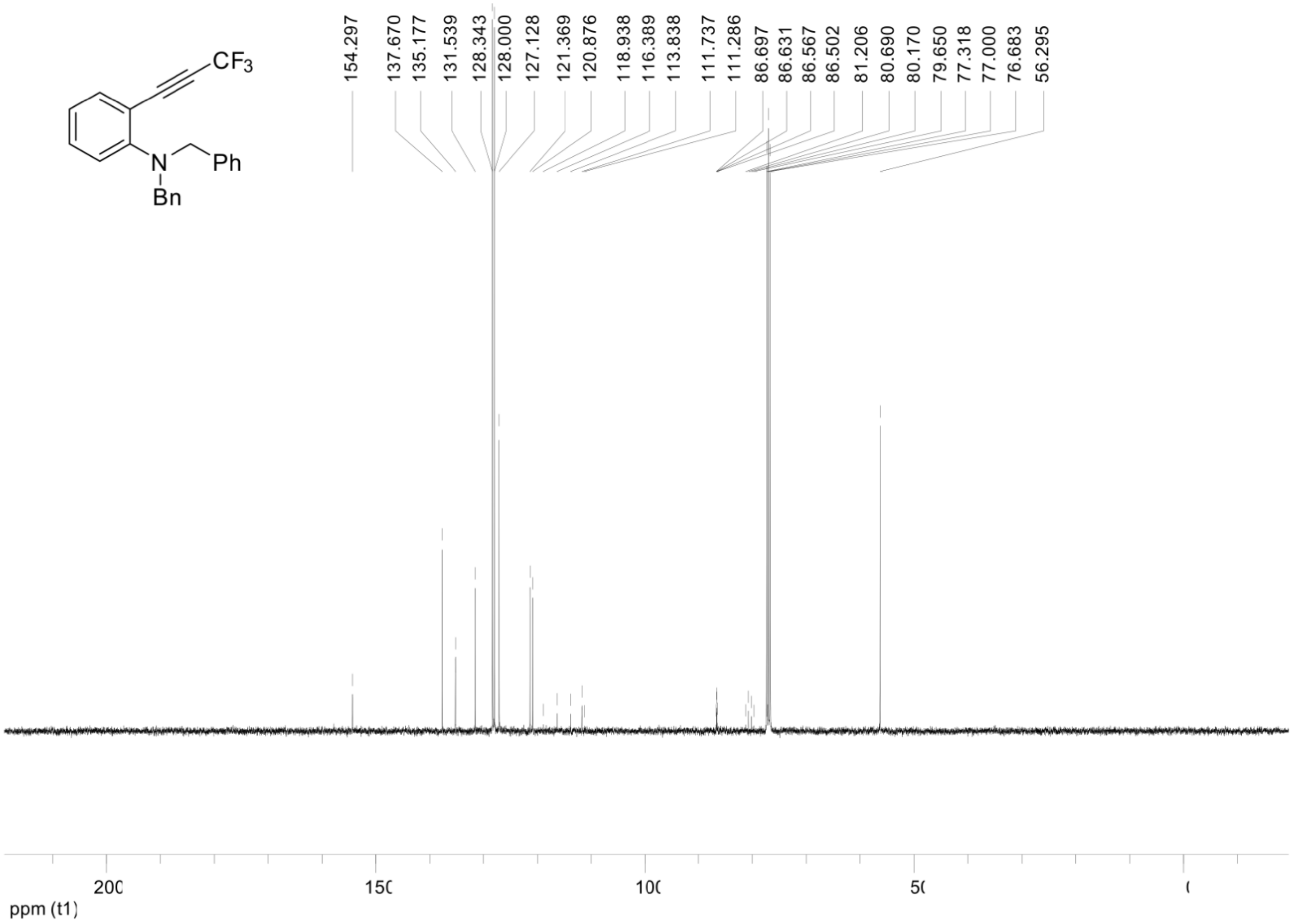

Supplementary Figure 46. ^{13}C NMR spectrum of **1s** (100 MHz, $CDCl_3$)

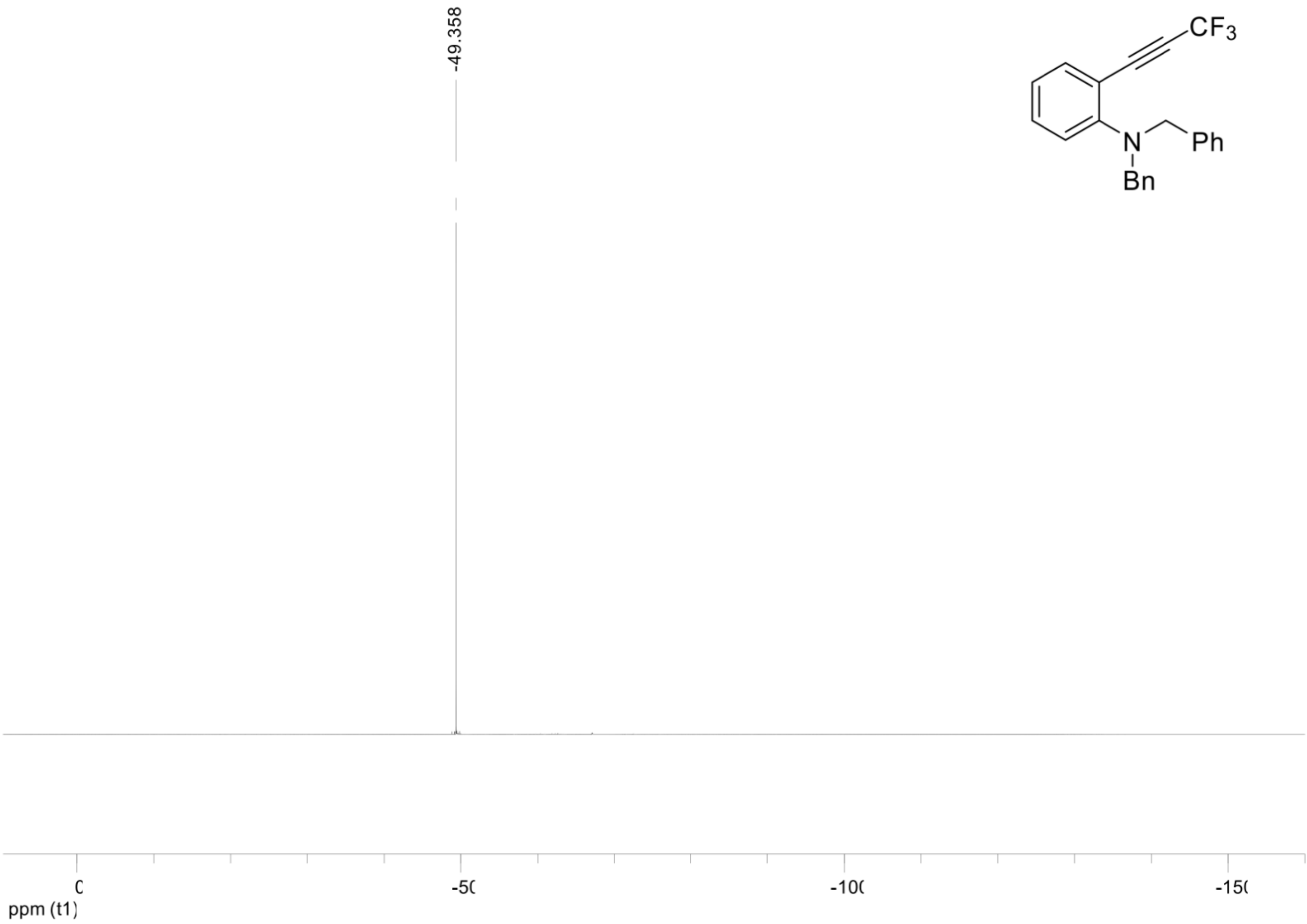

Supplementary Figure 47. ^{19}F NMR spectrum of **1s** (376 MHz, $CDCl_3$)

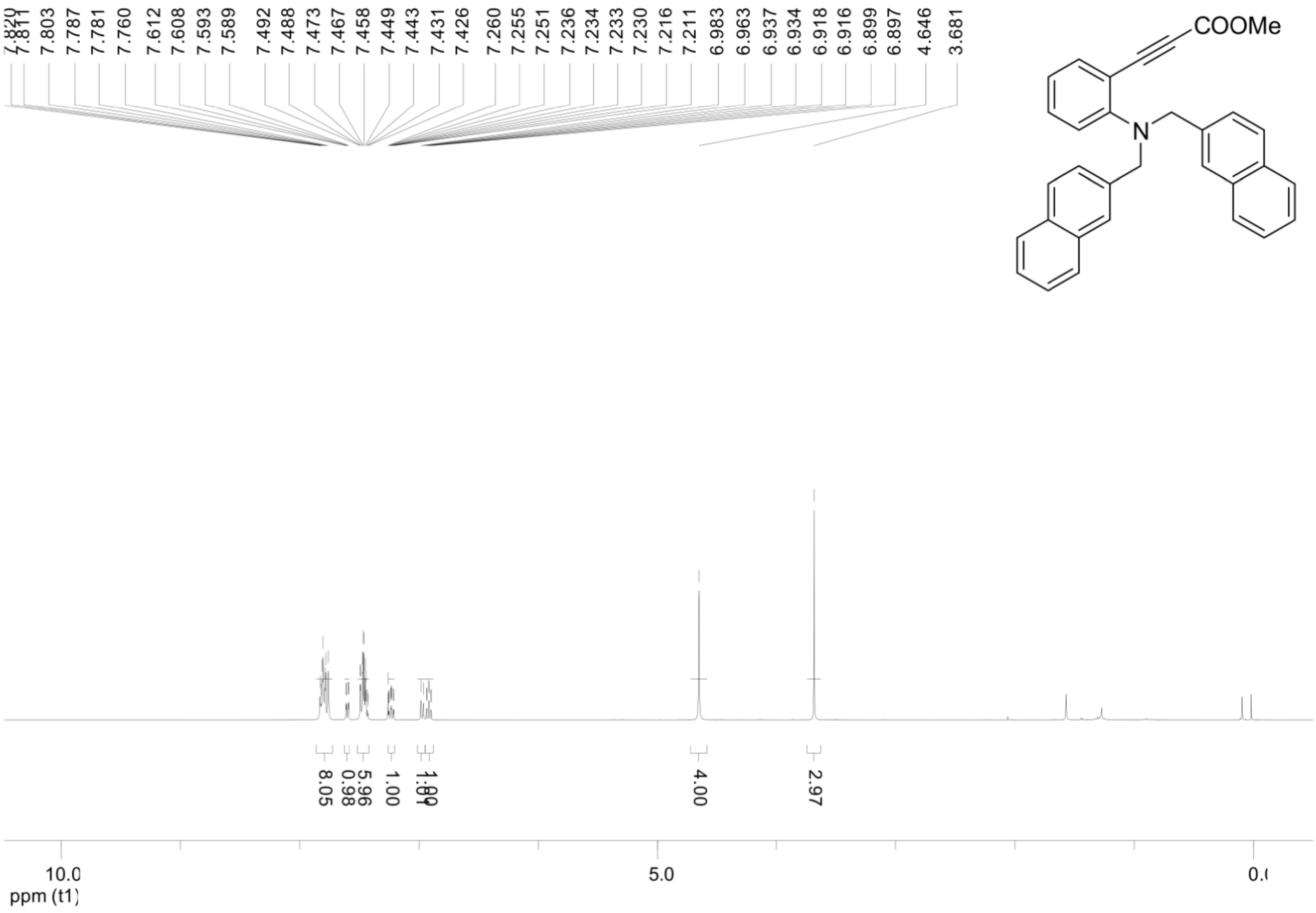

Supplementary Figure 48. ^{1}H NMR spectrum of **1t** (400 MHz, $CDCl_3$)

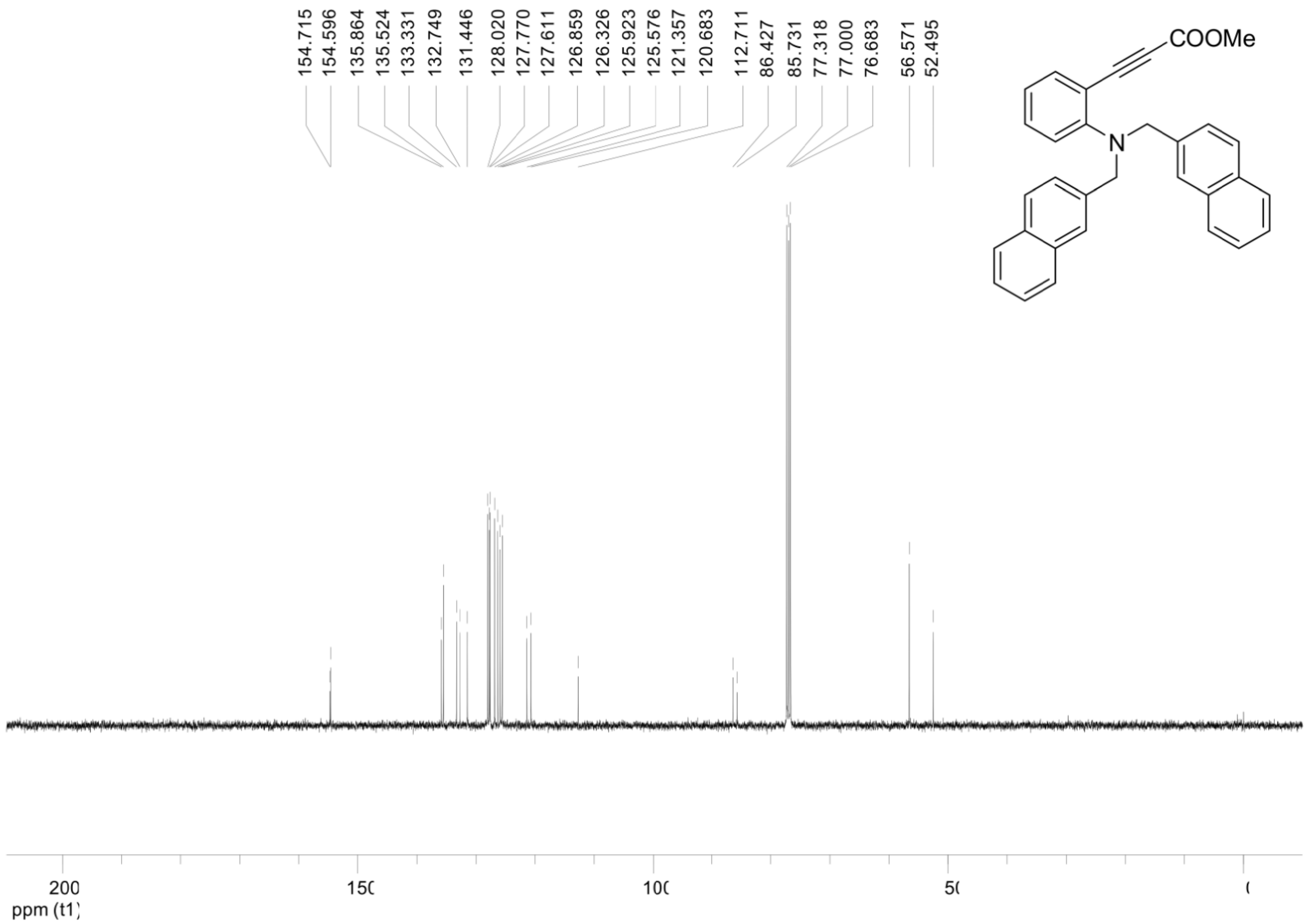

Supplementary Figure 49. ^{13}C NMR spectrum of **1t** (100 MHz, $CDCl_3$)

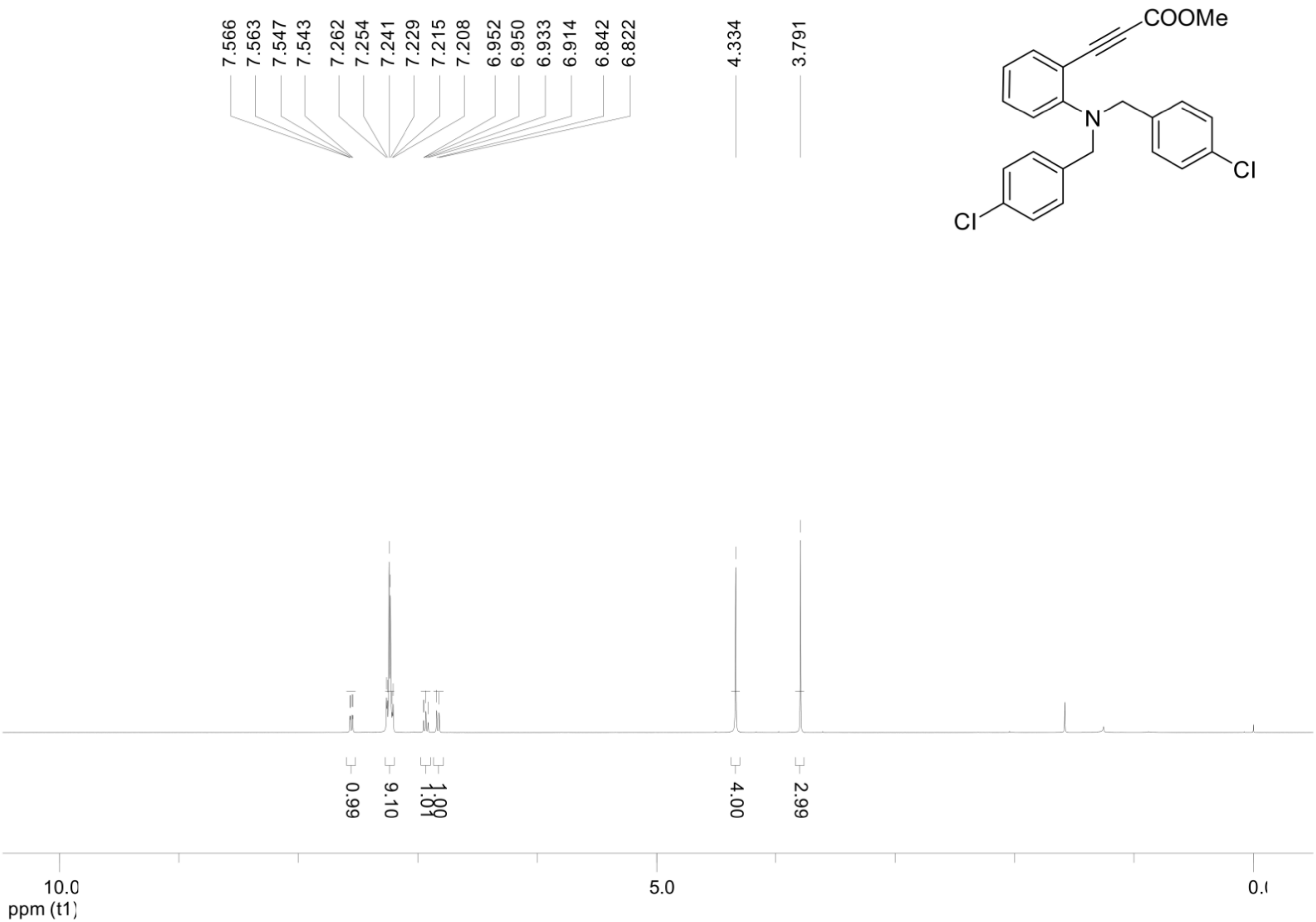

Supplementary Figure 50. ^{1}H NMR spectrum of **1u** (400 MHz, $CDCl_3$)

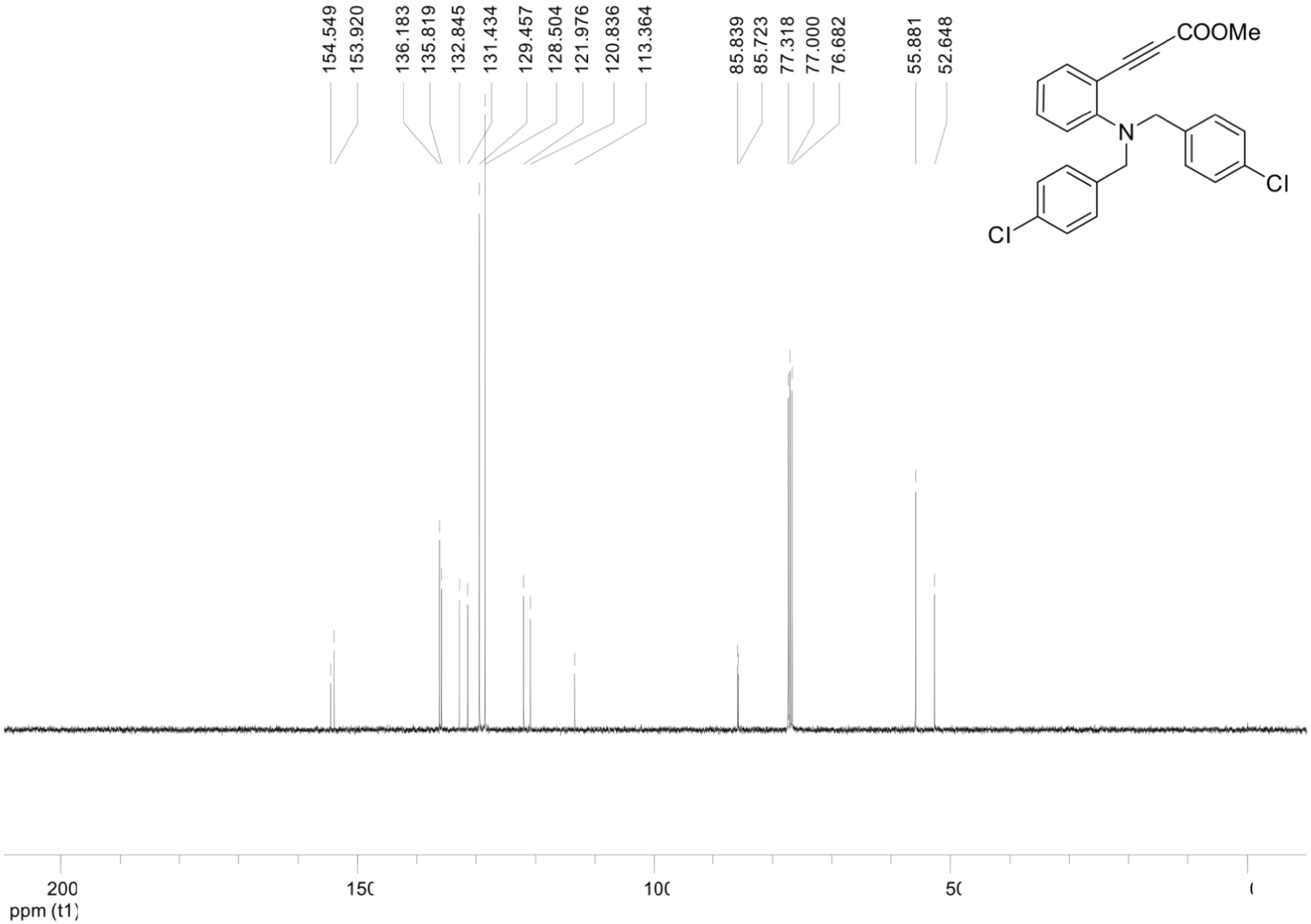

Supplementary Figure 51. ^{13}C NMR spectrum of **1u** (100 MHz, $CDCl_3$)

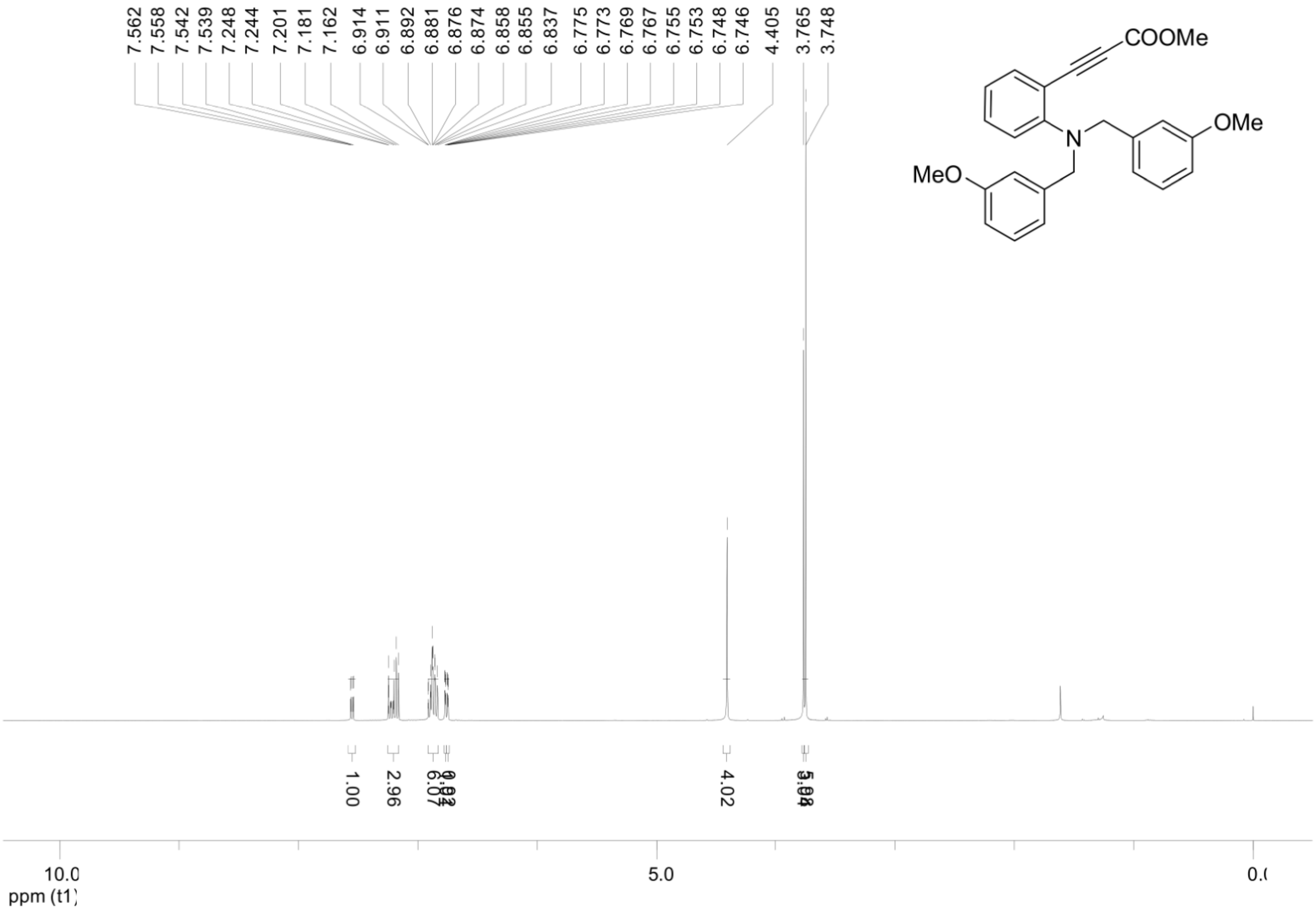

Supplementary Figure 52. ^{1}H NMR spectrum of **1v** (400 MHz, $CDCl_3$)

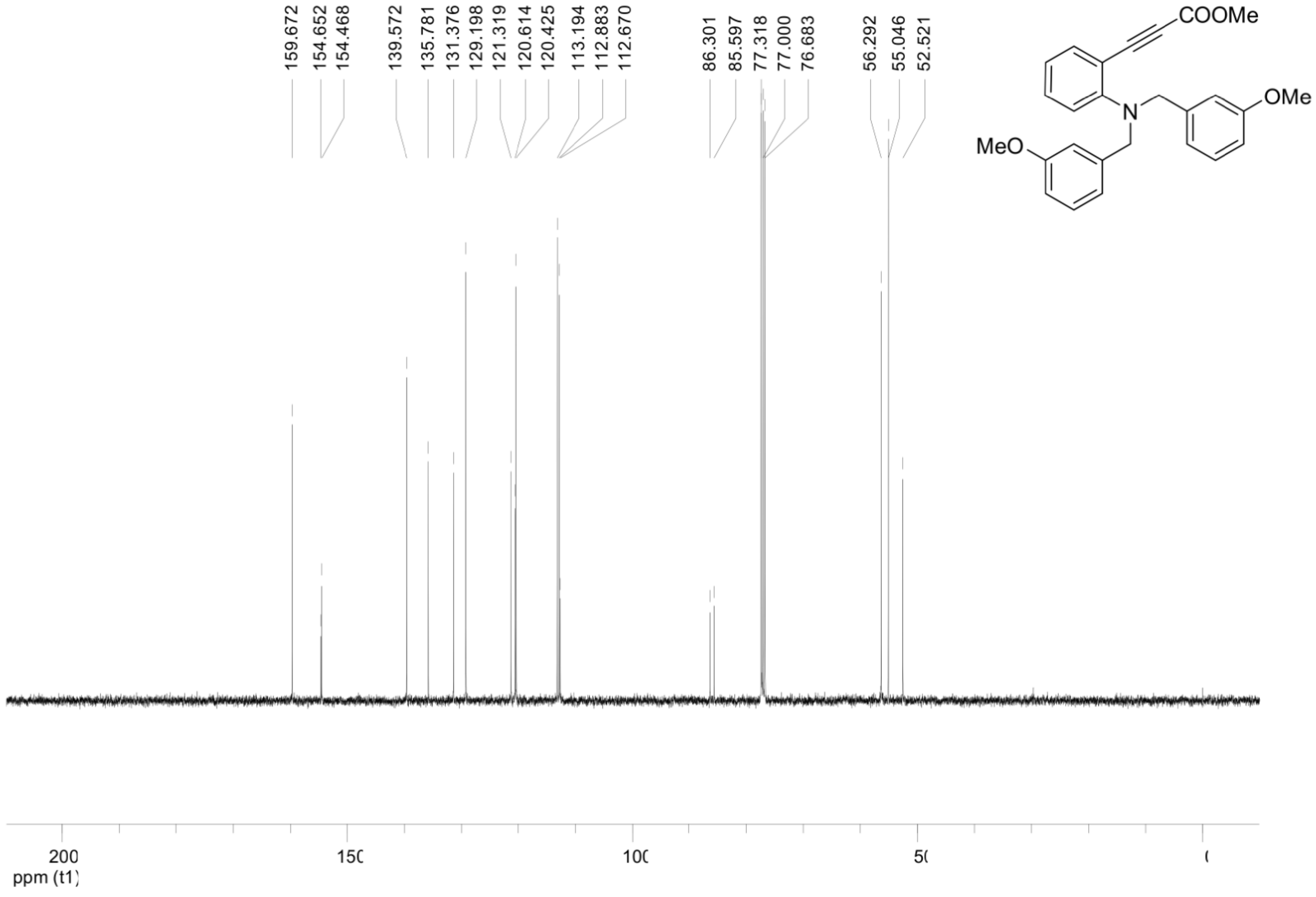

Supplementary Figure 53. ^{13}C NMR spectrum of **1v** (100 MHz, $CDCl_3$)

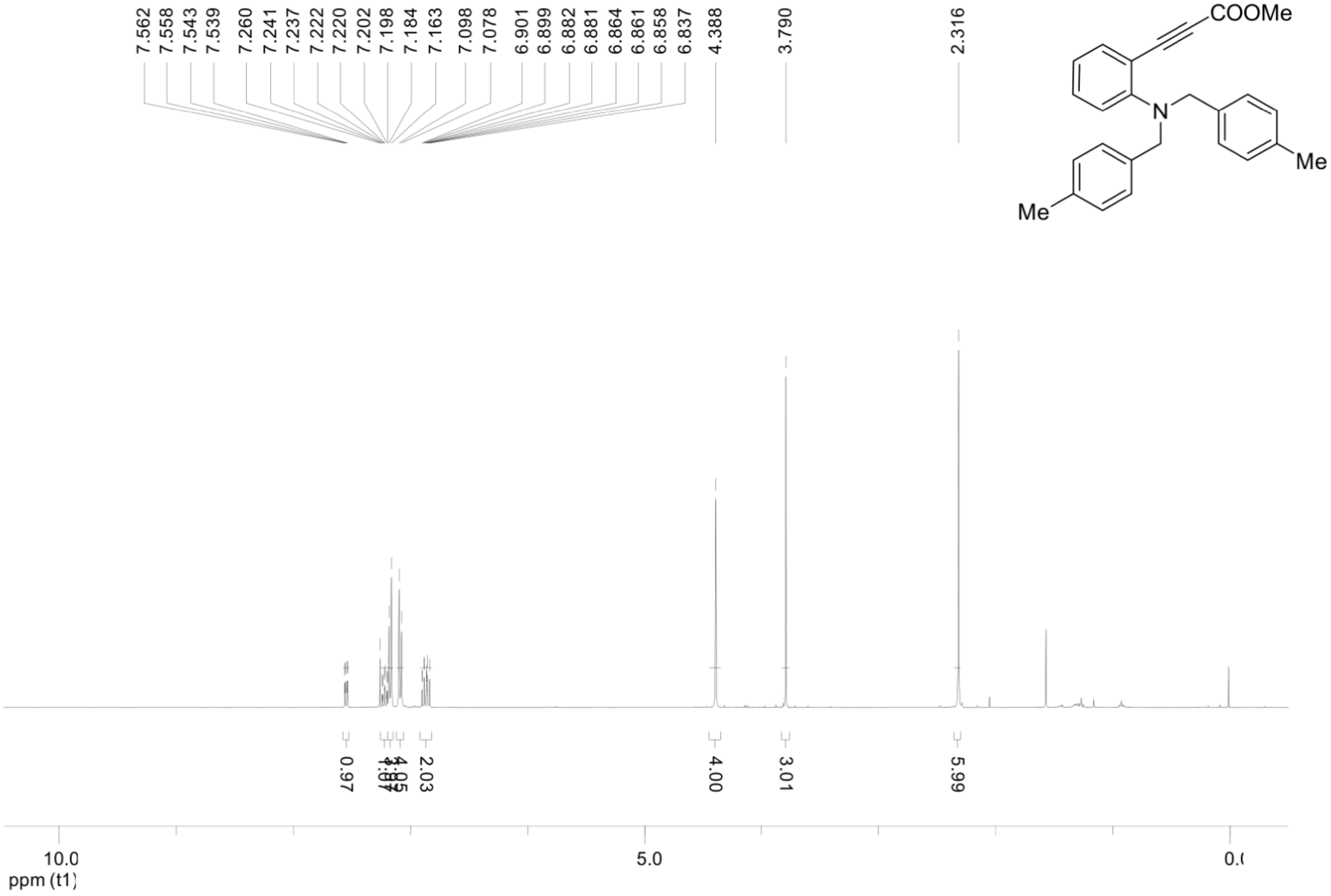

Supplementary Figure 54. ^{1}H NMR spectrum of **1w** (400 MHz, $CDCl_3$)

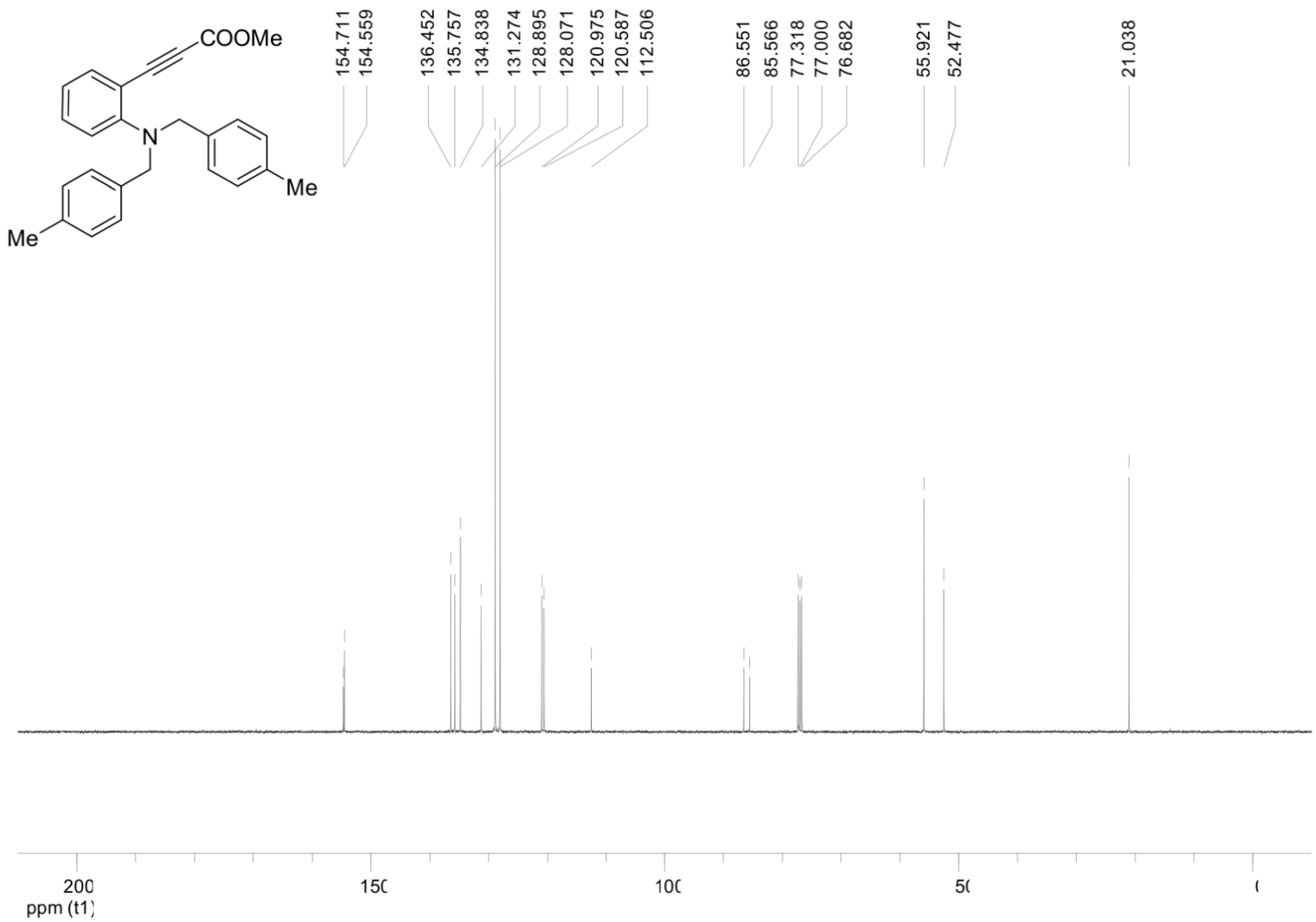

Supplementary Figure 55. ^{13}C NMR spectrum of **1w** (100 MHz, $CDCl_3$)

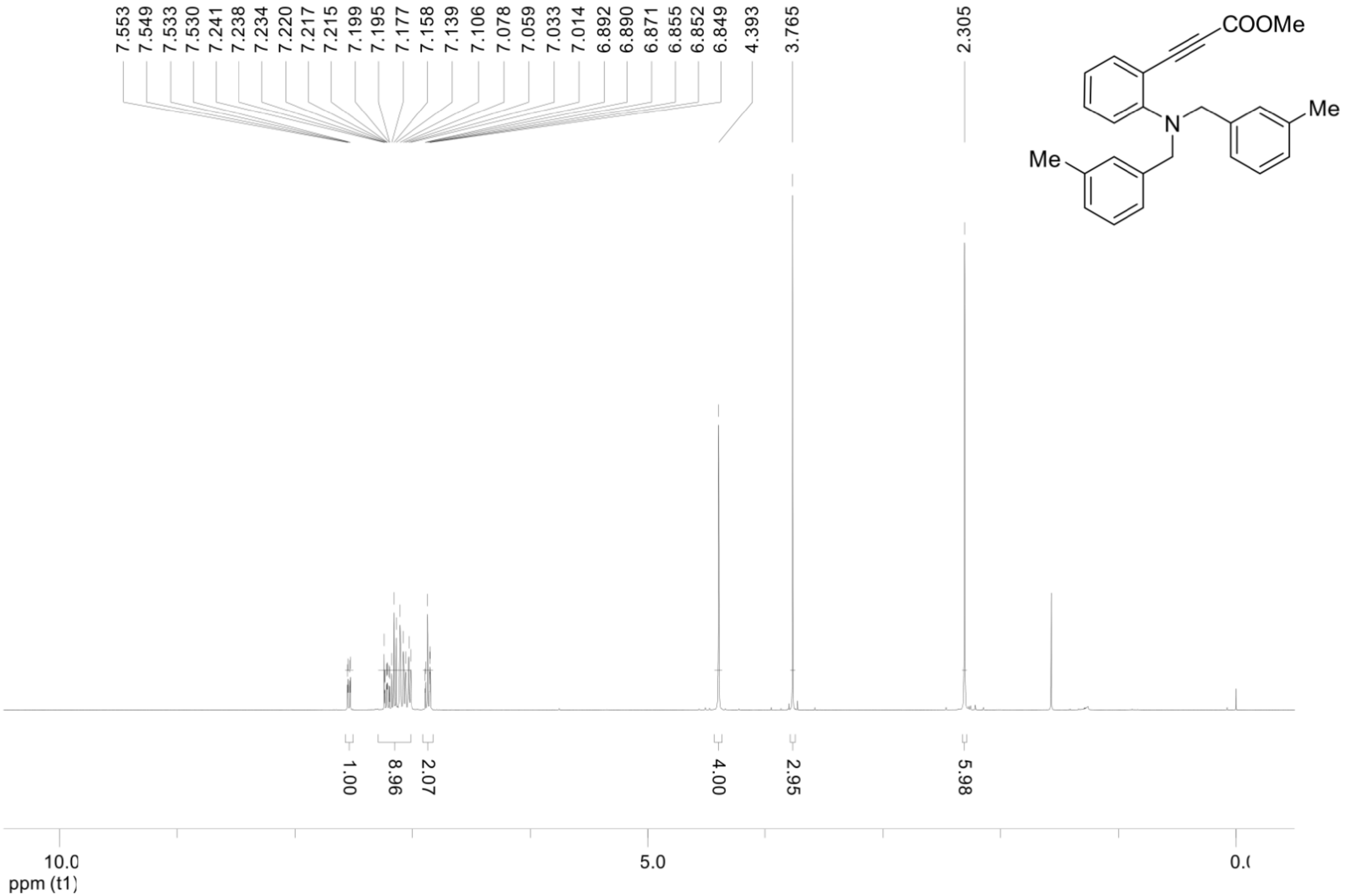

Supplementary Figure 56. ^{1}H NMR spectrum of **1x** (400 MHz, $CDCl_3$)

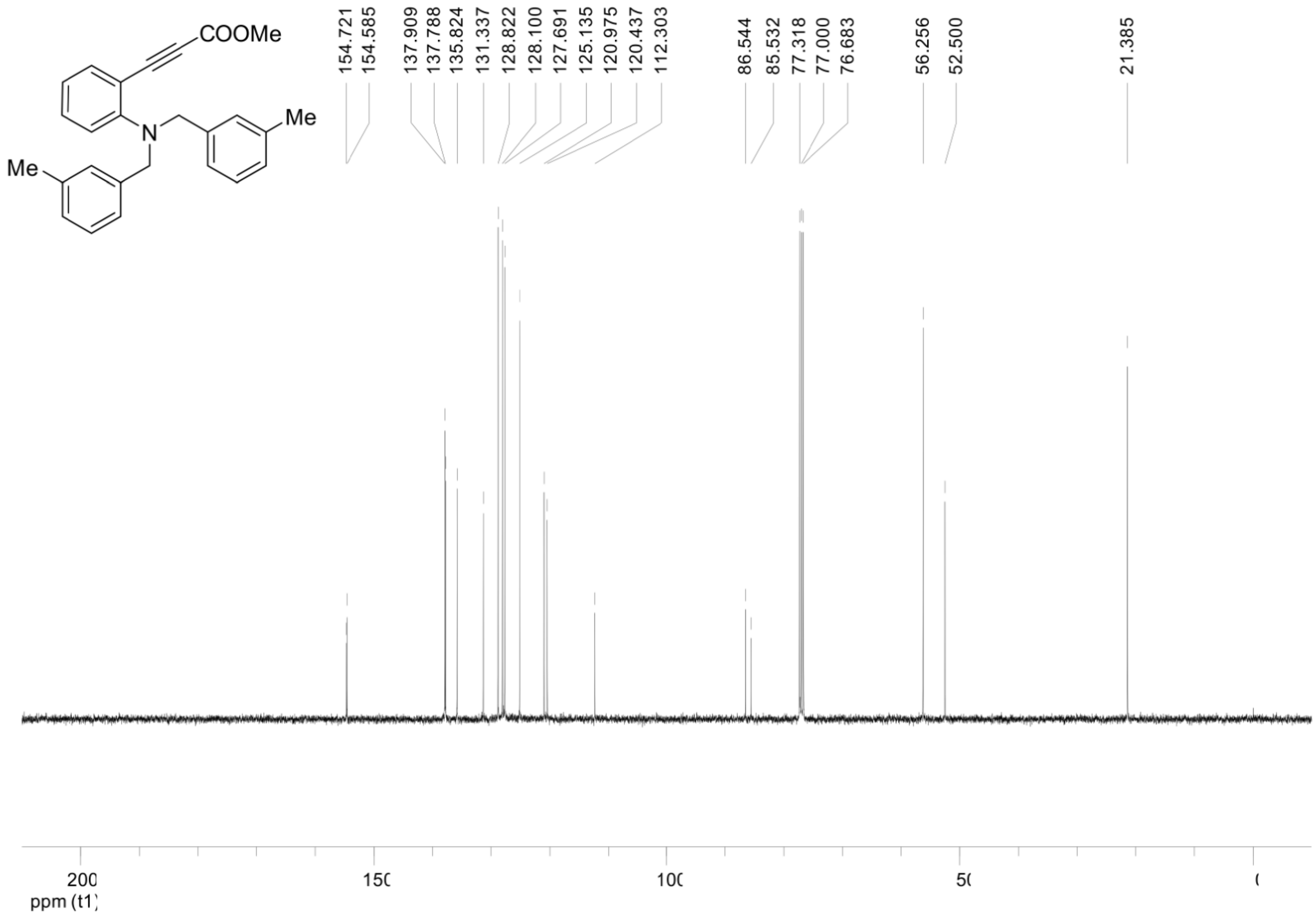

Supplementary Figure 57. ^{13}C NMR spectrum of **1x** (100 MHz, $CDCl_3$)

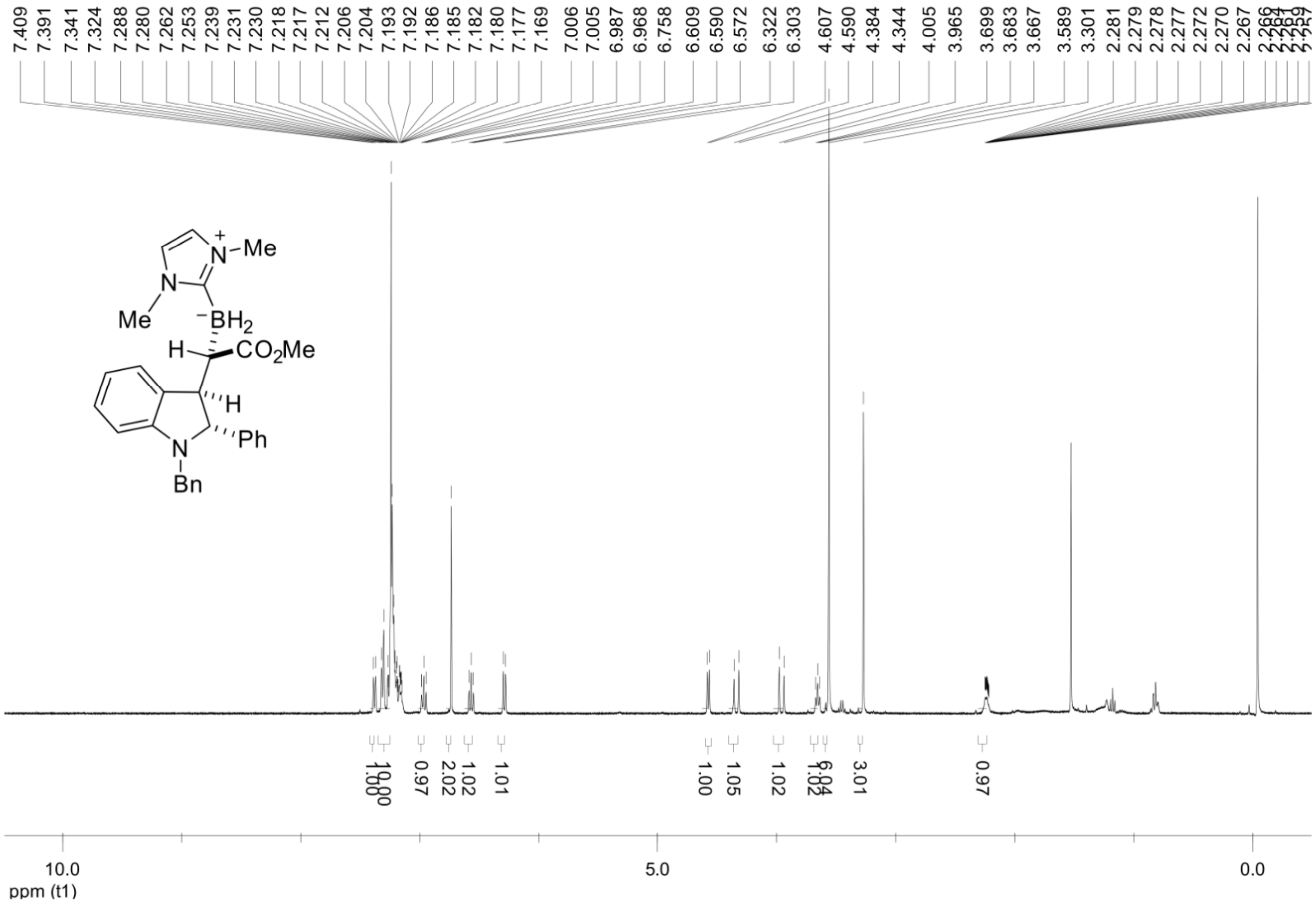

Supplementary Figure 58. ^{1}H NMR spectrum of **3a-Me** (400 MHz, $CDCl_3$)

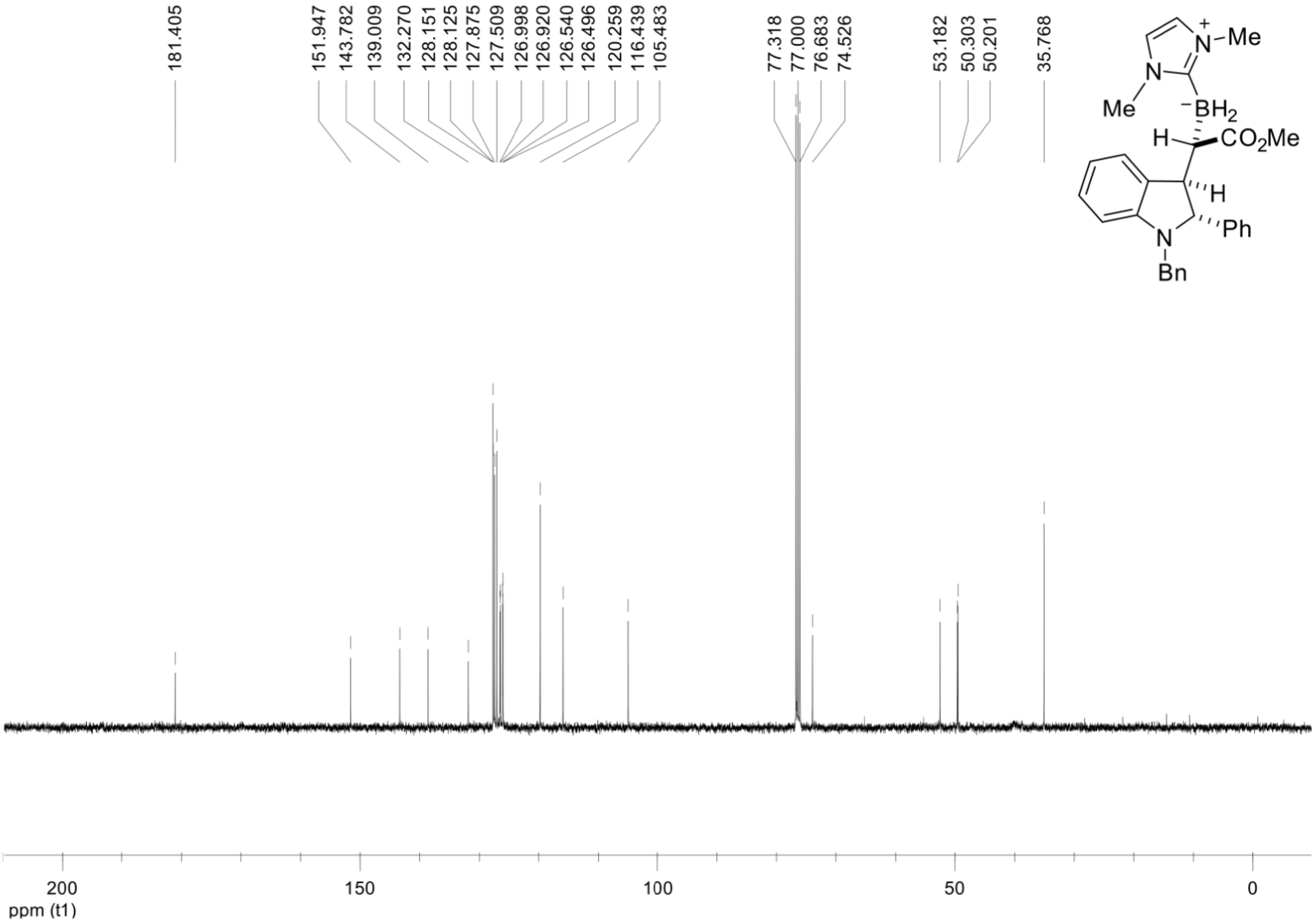

Supplementary Figure 59. ^{13}C NMR spectrum of **3a-Me** (100 MHz, $CDCl_3$)

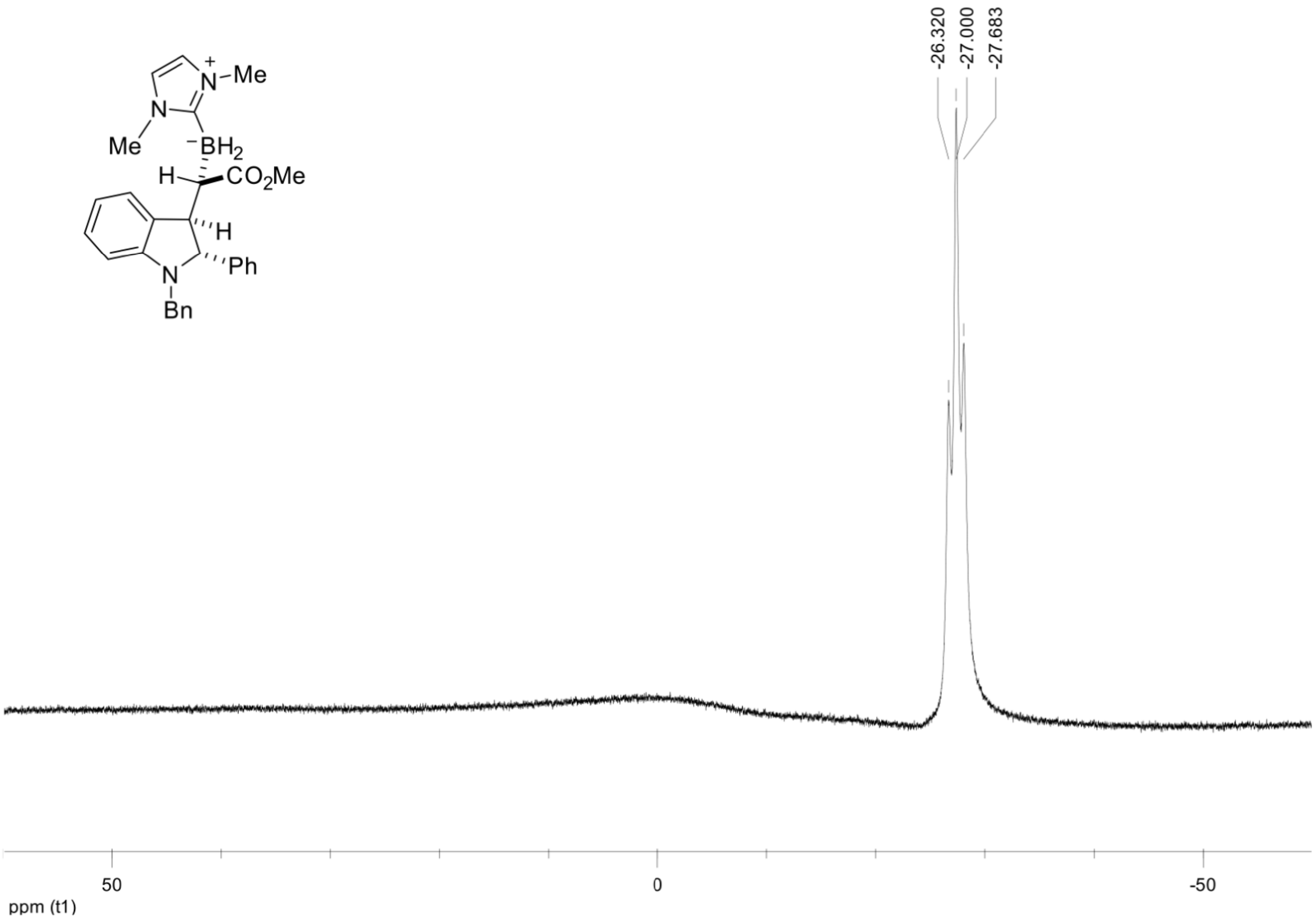

Supplementary Figure 60. ^{11}B NMR spectrum of **3a-Me** (128.4 MHz, $CDCl_3$)

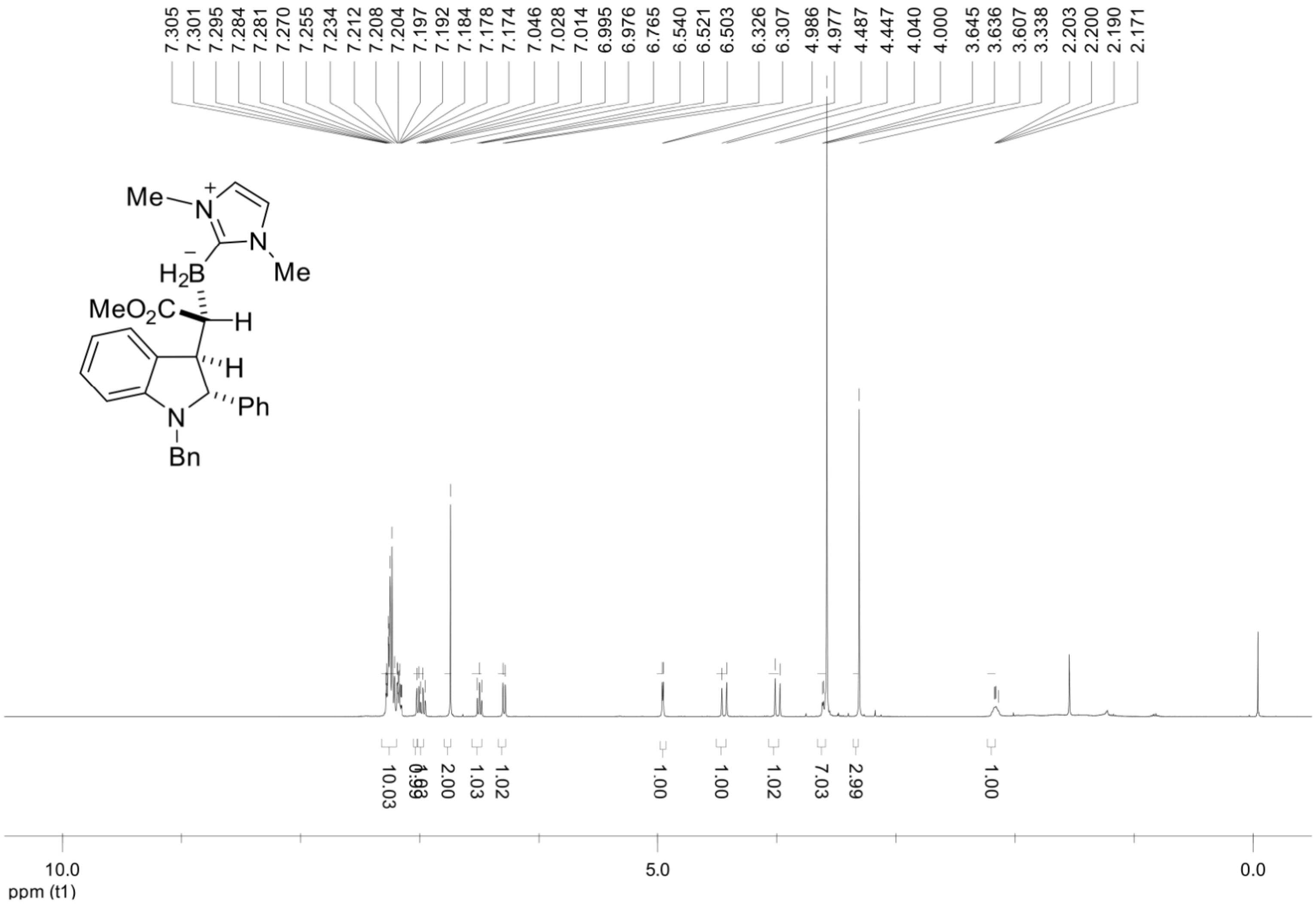

Supplementary Figure 61. ^{1}H NMR spectrum of **4a-Me** (400 MHz, $CDCl_3$)

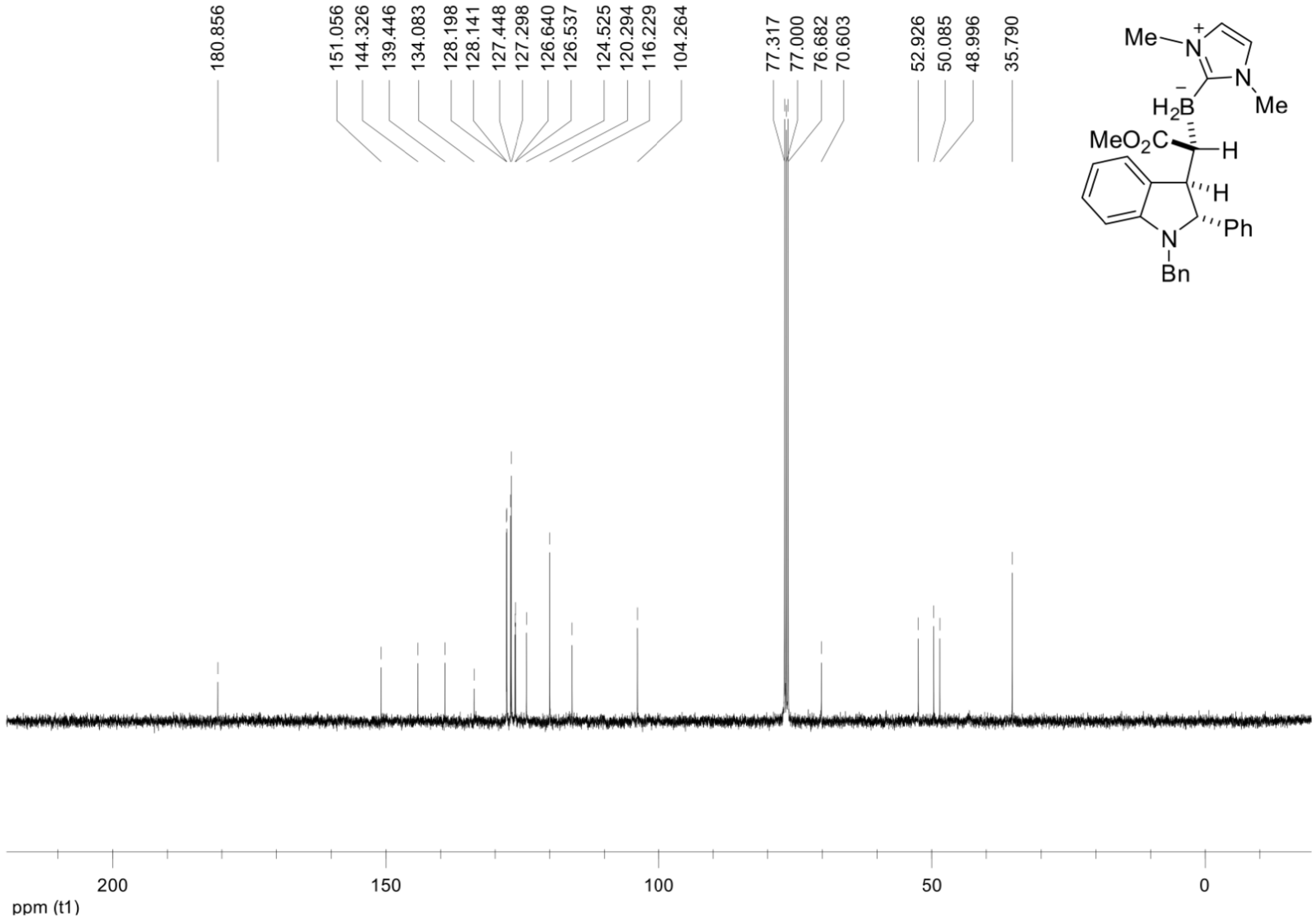

Supplementary Figure 62. ^{13}C NMR spectrum of **4a-Me** (100 MHz, $CDCl_3$)

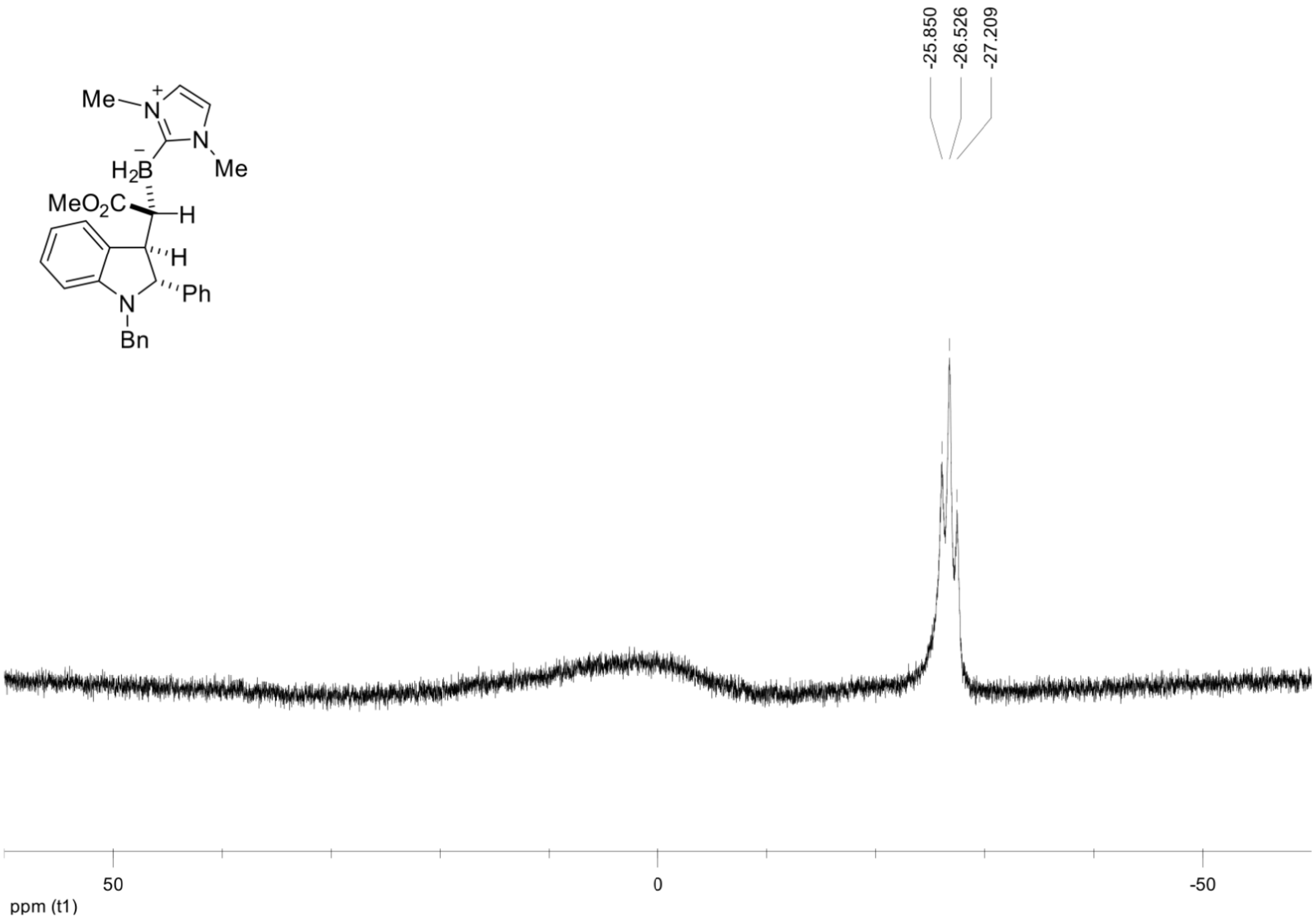

Supplementary Figure 63. ^{11}B NMR spectrum of **4a-Me** (128.4 MHz, $CDCl_3$)

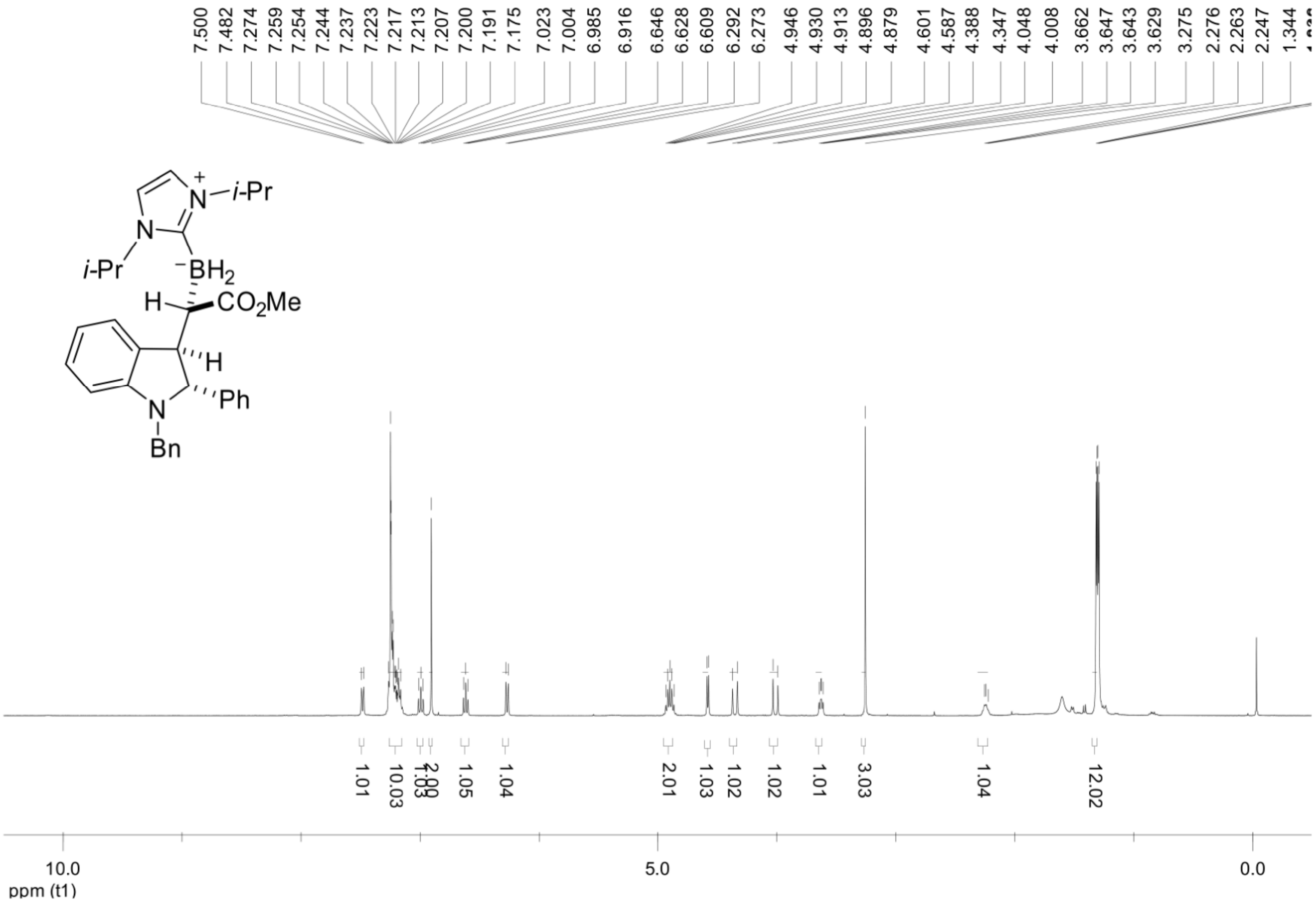

Supplementary Figure 64. ^{1}H NMR spectrum of **3a-*i*-Pr** (400 MHz, $CDCl_3$)

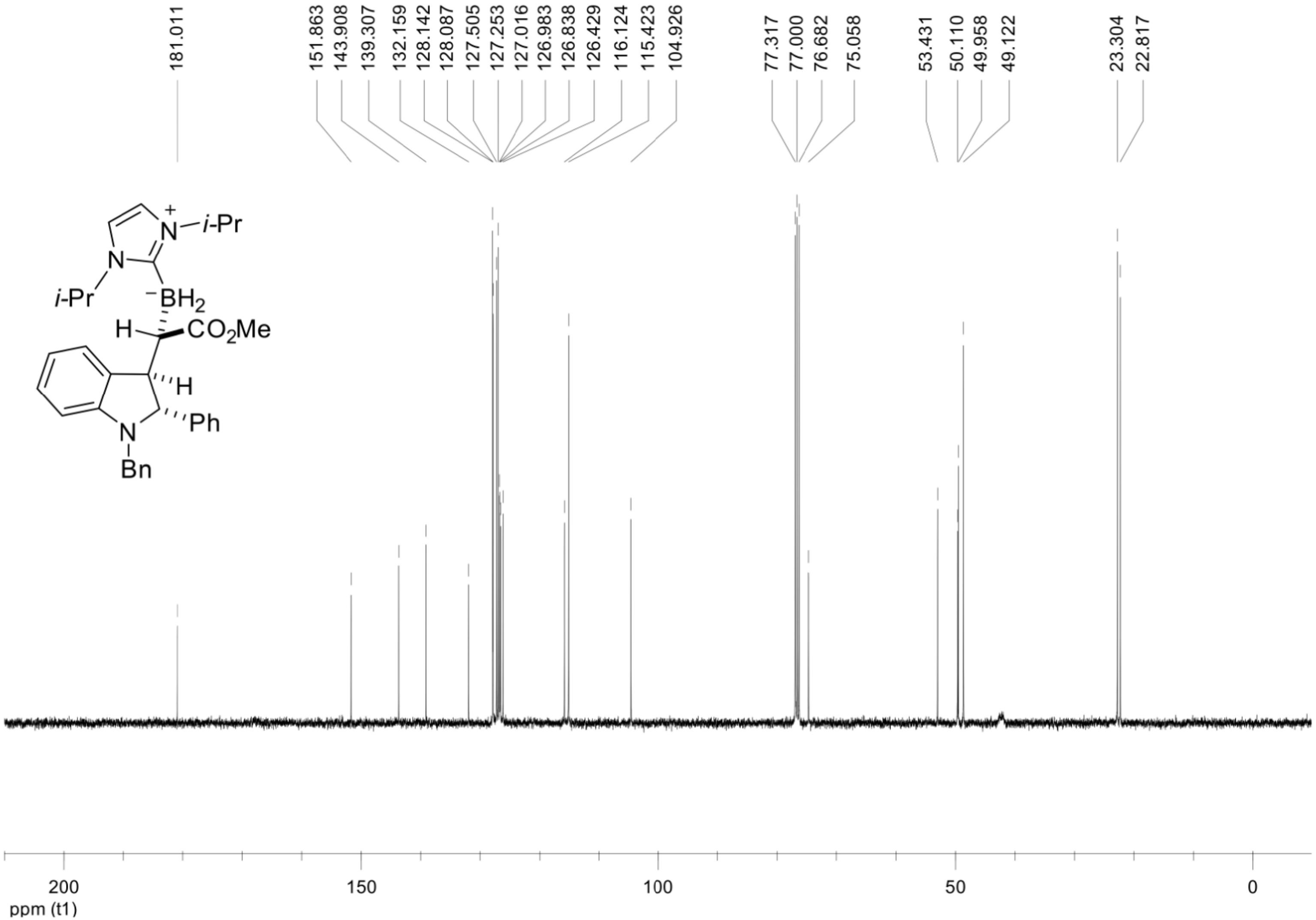

Supplementary Figure 65. ^{13}C NMR spectrum of **3a-*i*-Pr** (100 MHz, $CDCl_3$)

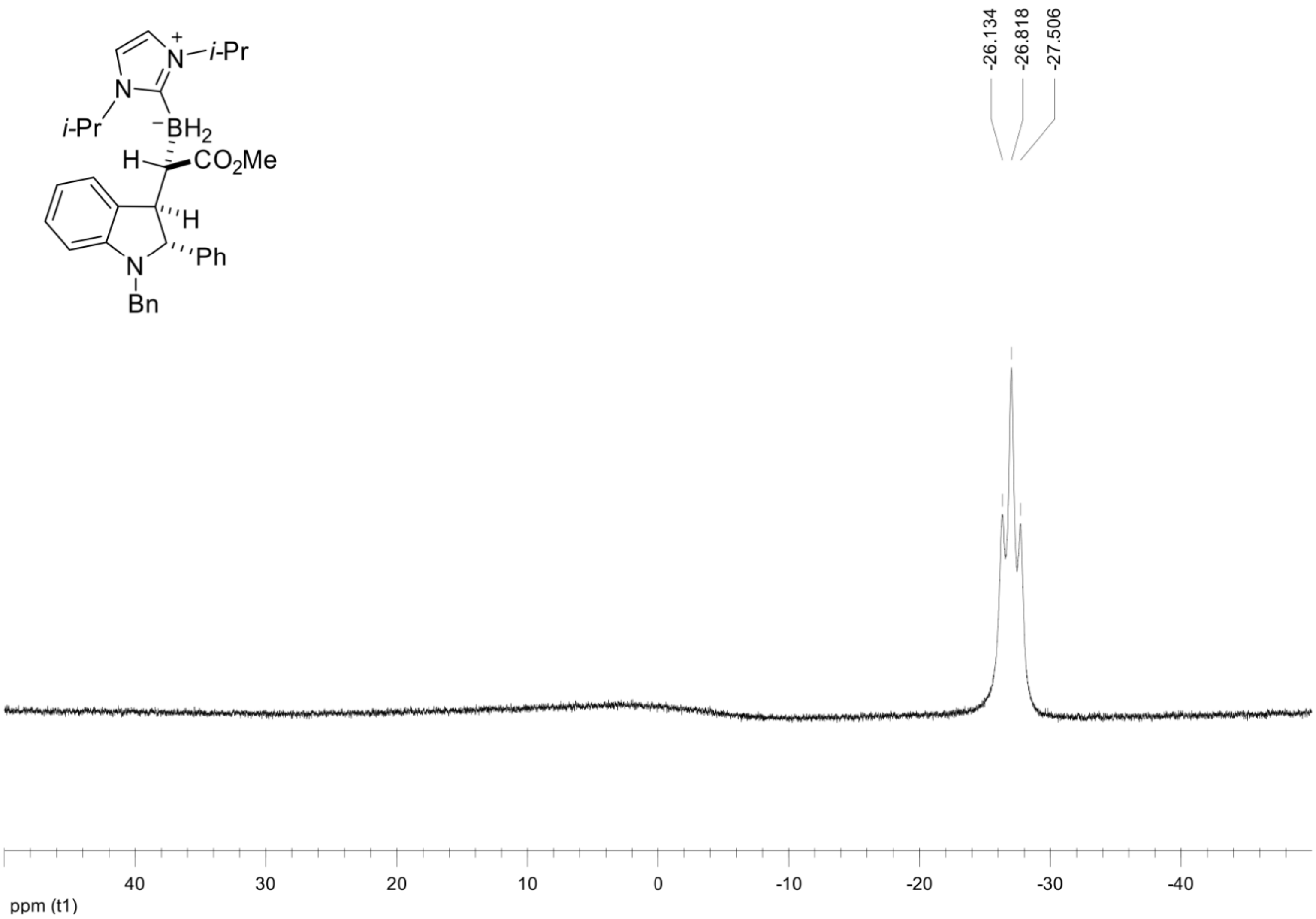

Supplementary Figure 66. ^{11}B NMR spectrum of **3a-*i*-Pr** (128.4 MHz, $CDCl_3$)

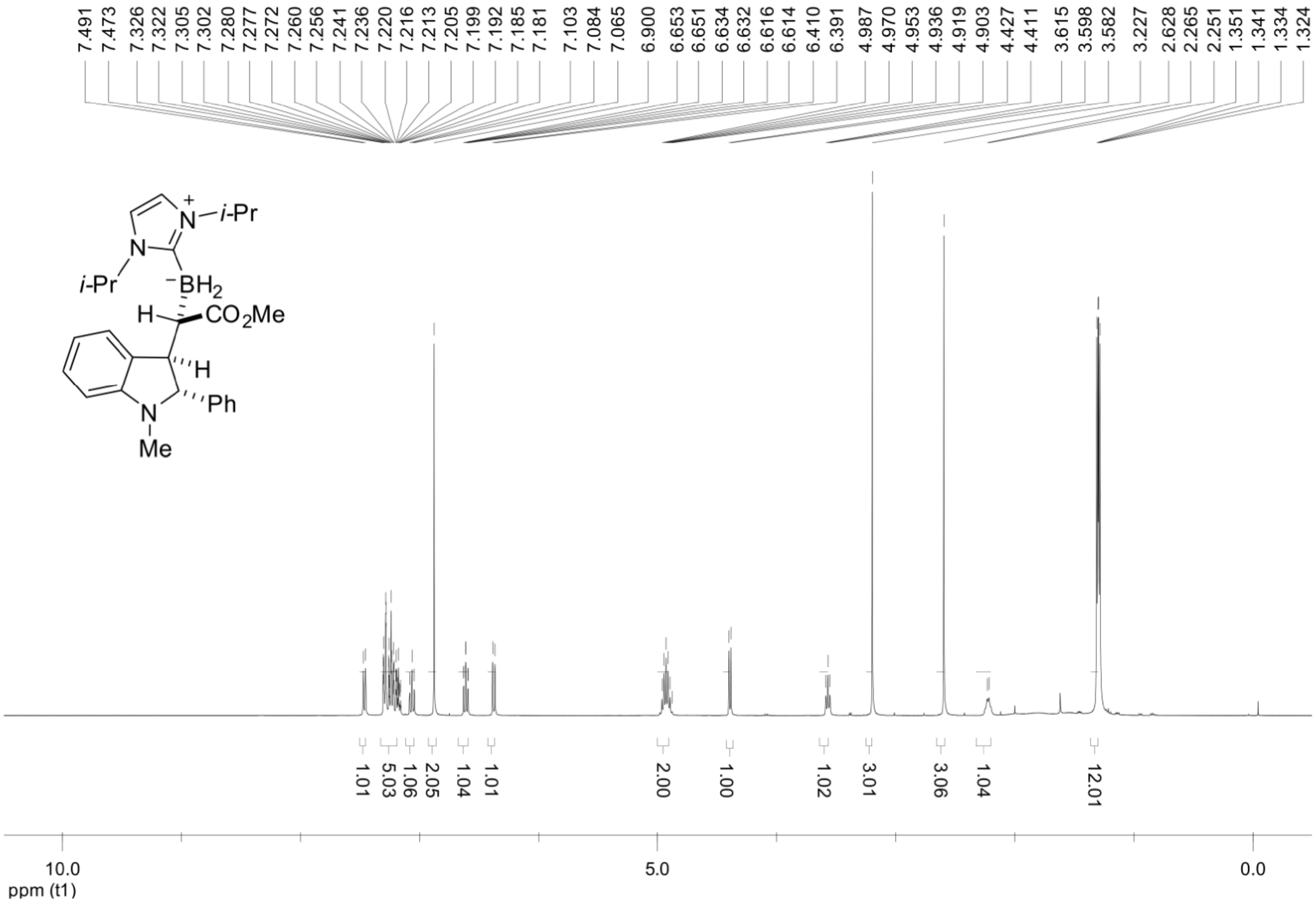

Supplementary Figure 67. ^{1}H NMR spectrum of **3b** (400 MHz, $CDCl_3$)

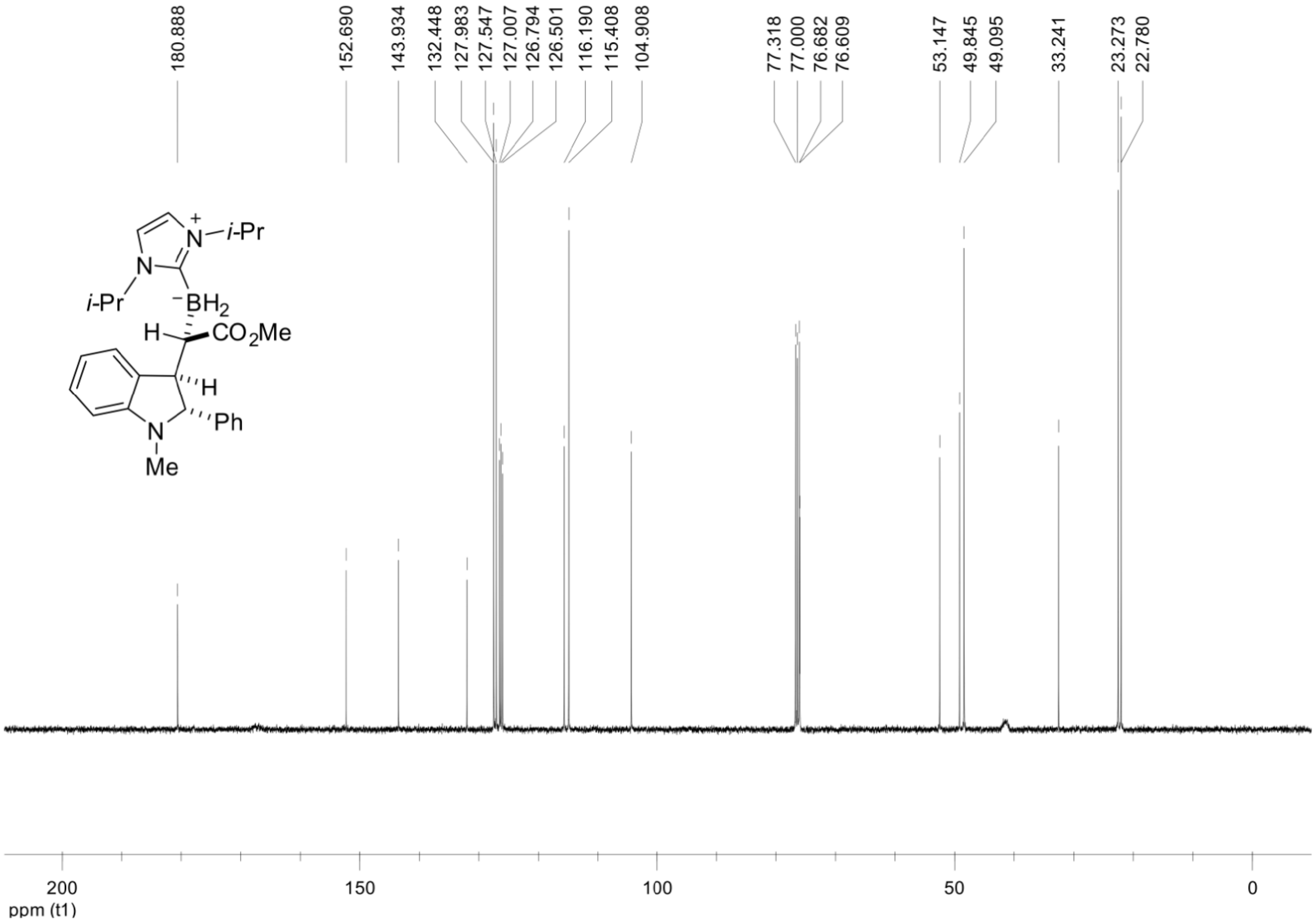

Supplementary Figure 68. ^{13}C NMR spectrum of **3b** (100 MHz, $CDCl_3$)

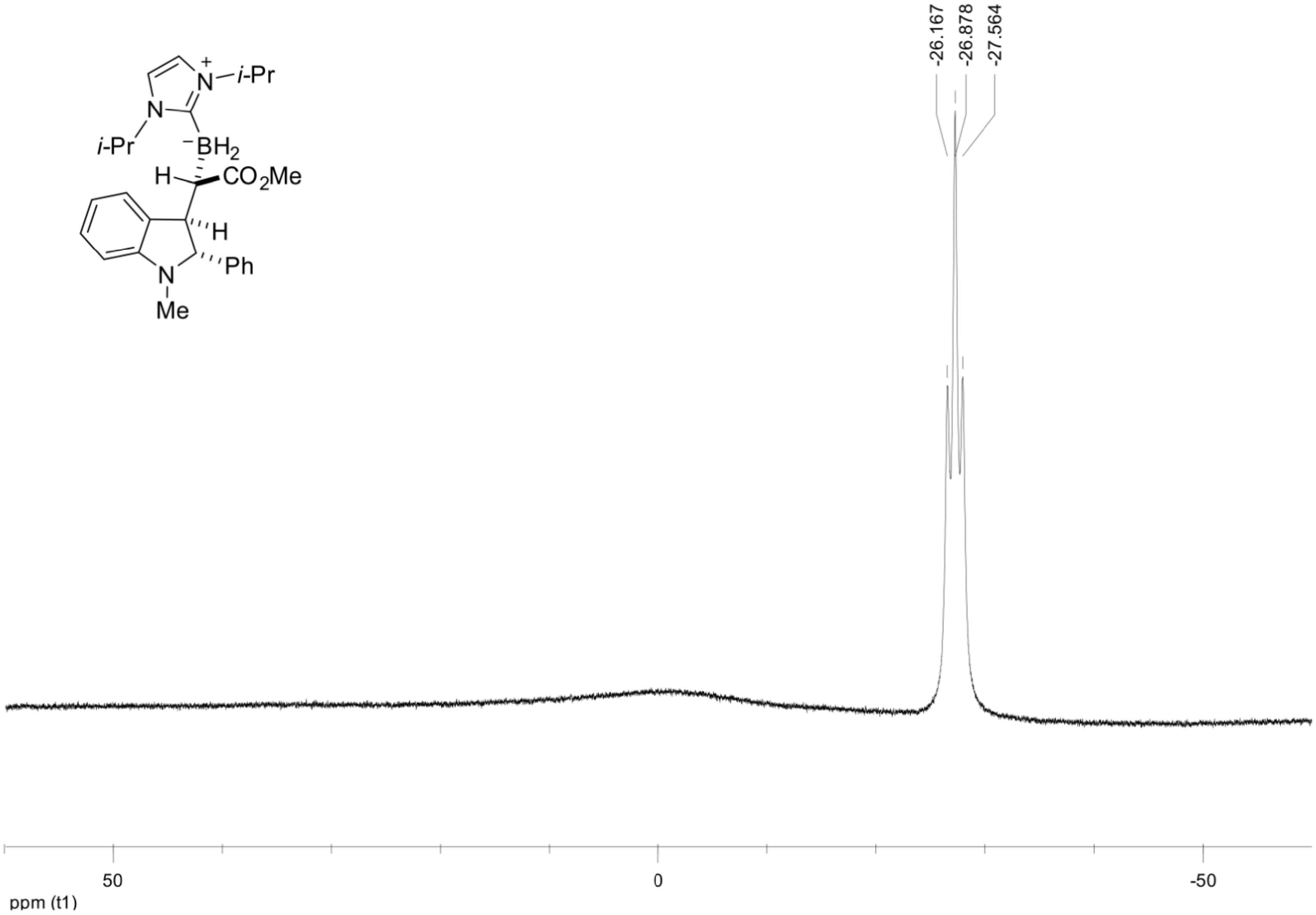

Supplementary Figure 69. ^{11}B NMR spectrum of **3b** (128.4 MHz, $CDCl_3$)

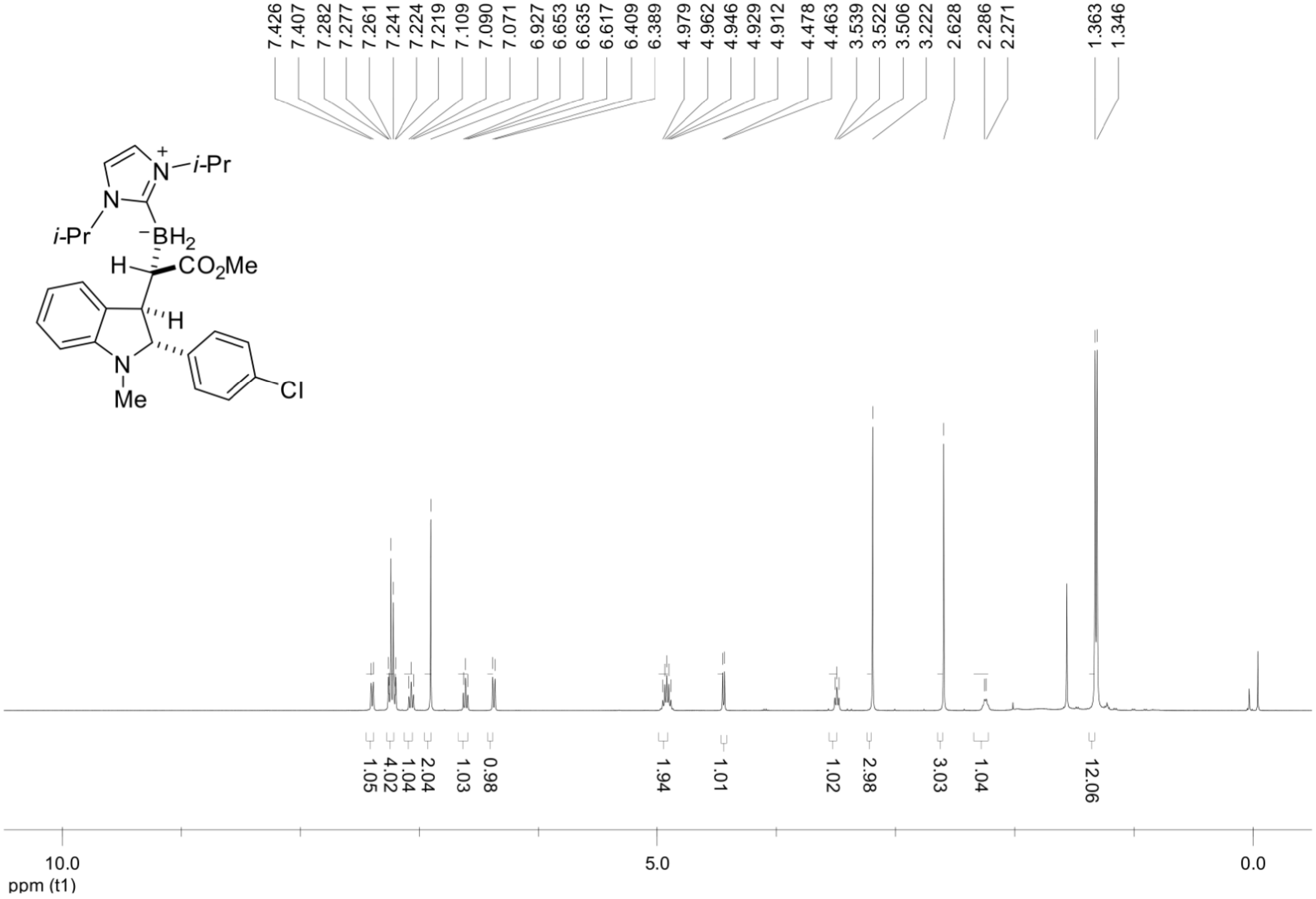

Supplementary Figure 70. ^{1}H NMR spectrum of **3c** (400 MHz, $CDCl_3$)

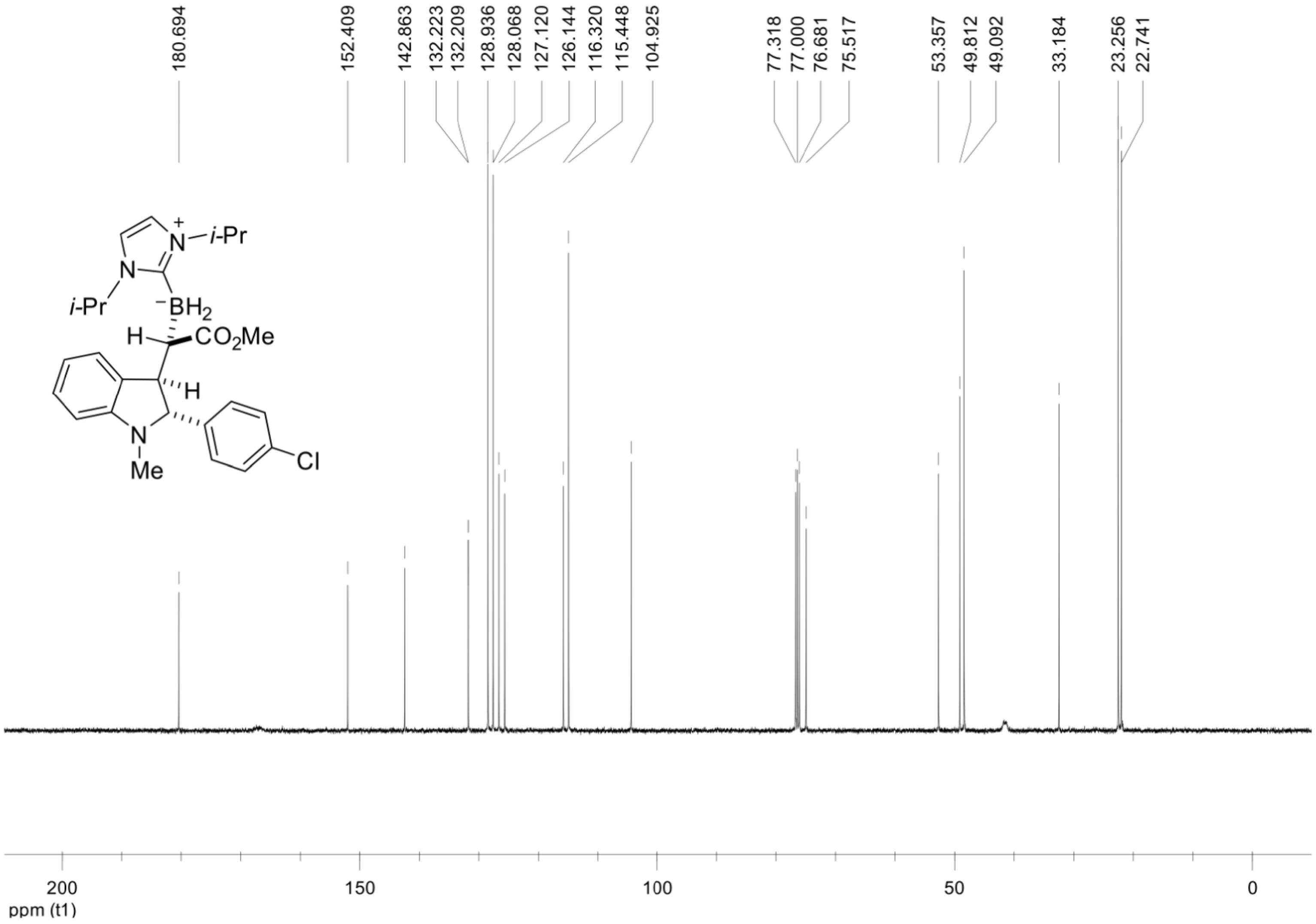

Supplementary Figure 71. ^{13}C NMR spectrum of **3c** (100 MHz, $CDCl_3$)

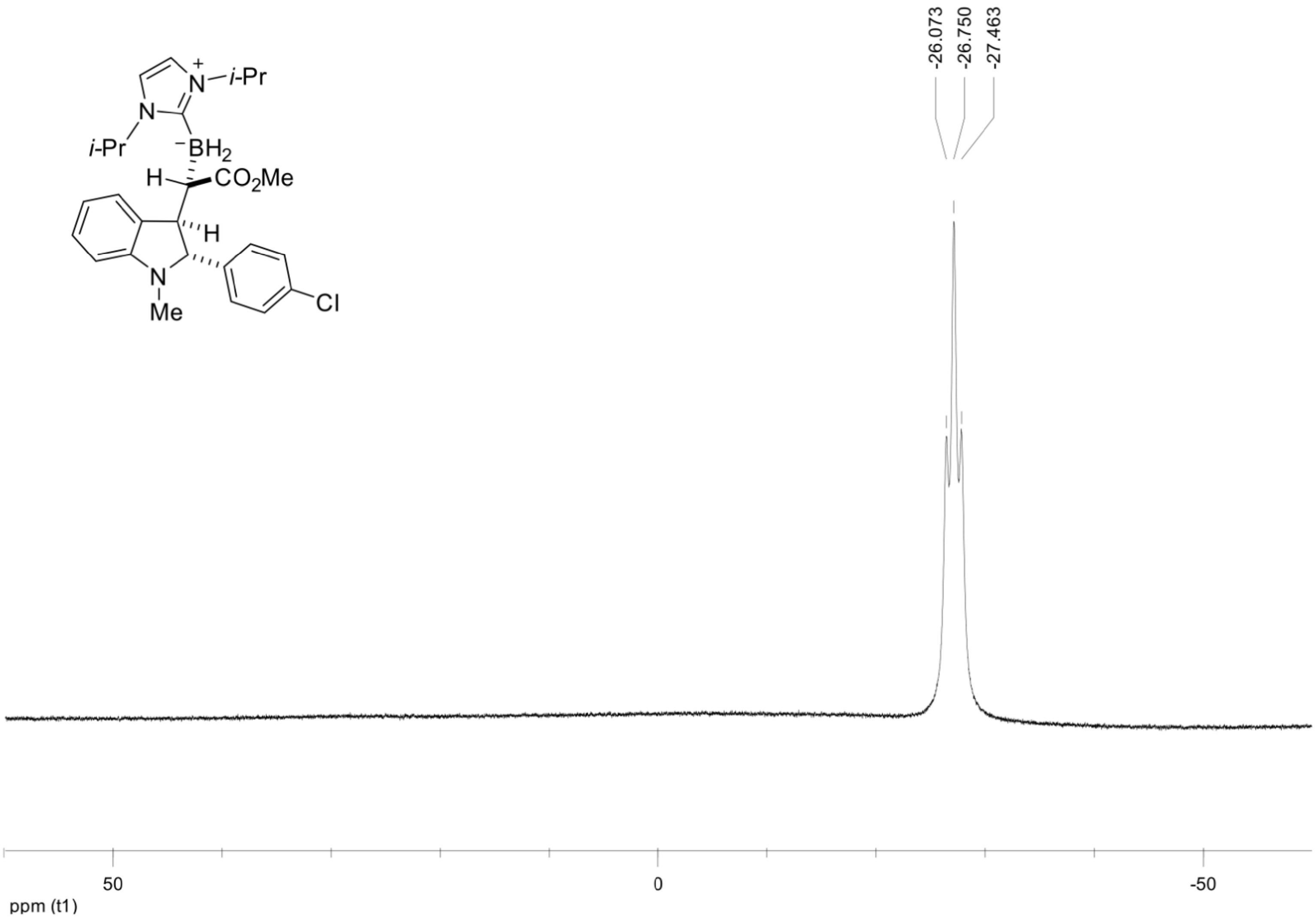

Supplementary Figure 72. ^{11}B NMR spectrum of **3c** (128.4 MHz, $CDCl_3$)

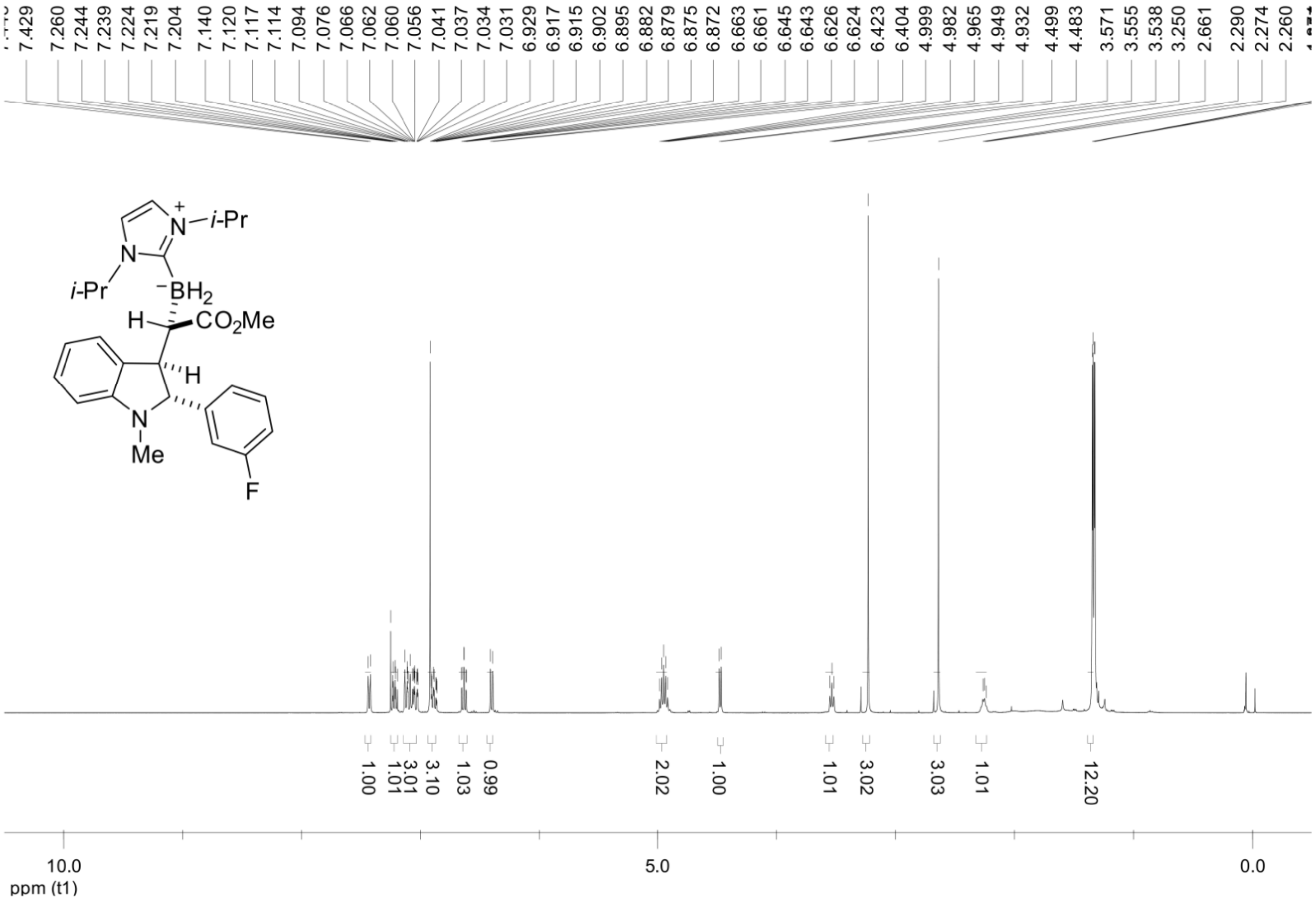

Supplementary Figure 73. ^{1}H NMR spectrum of **3d** (400 MHz, $CDCl_3$)

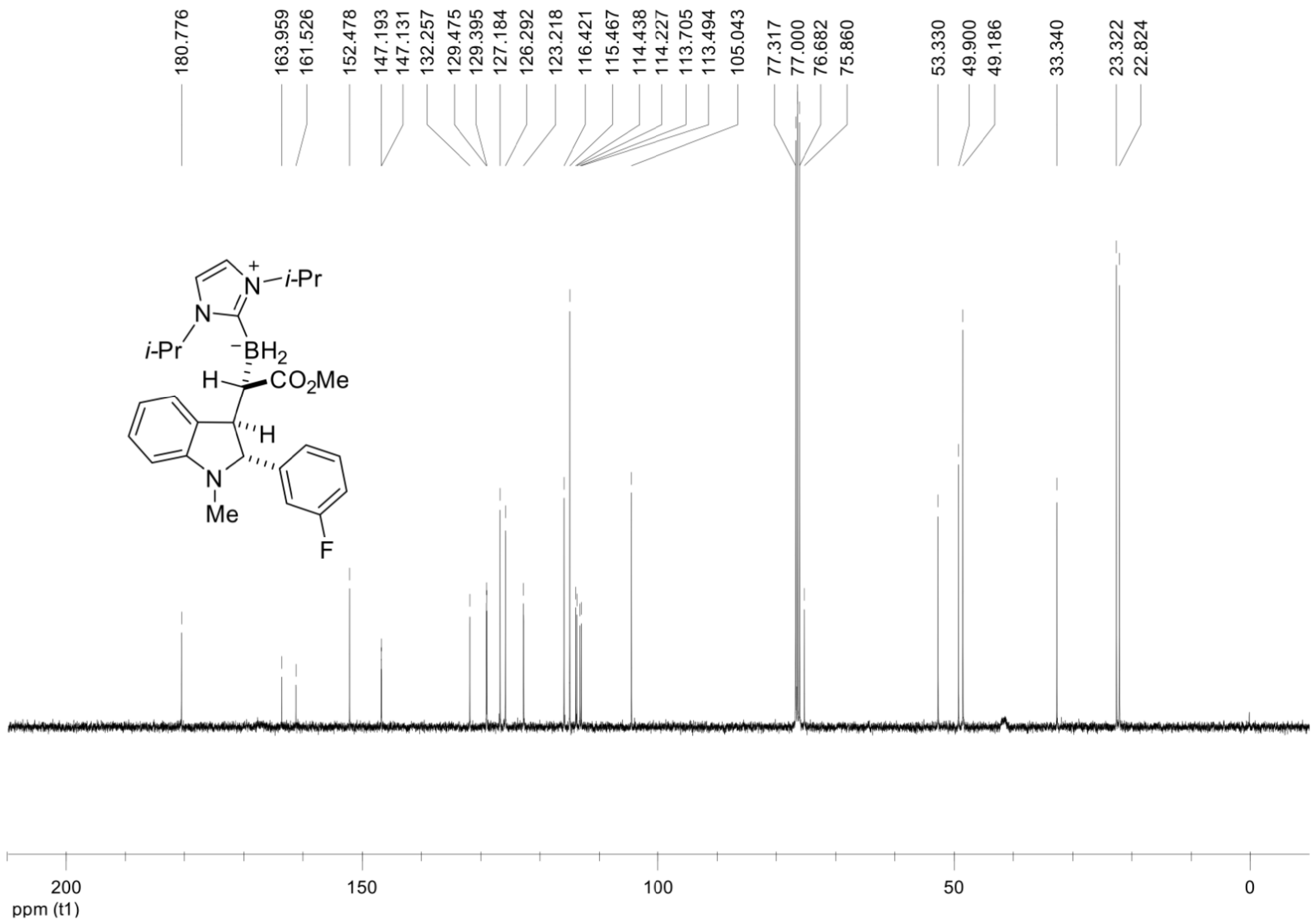

Supplementary Figure 74. ^{13}C NMR spectrum of **3d** (100 MHz, $CDCl_3$)

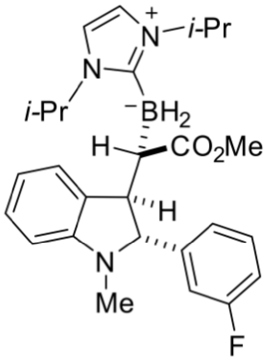

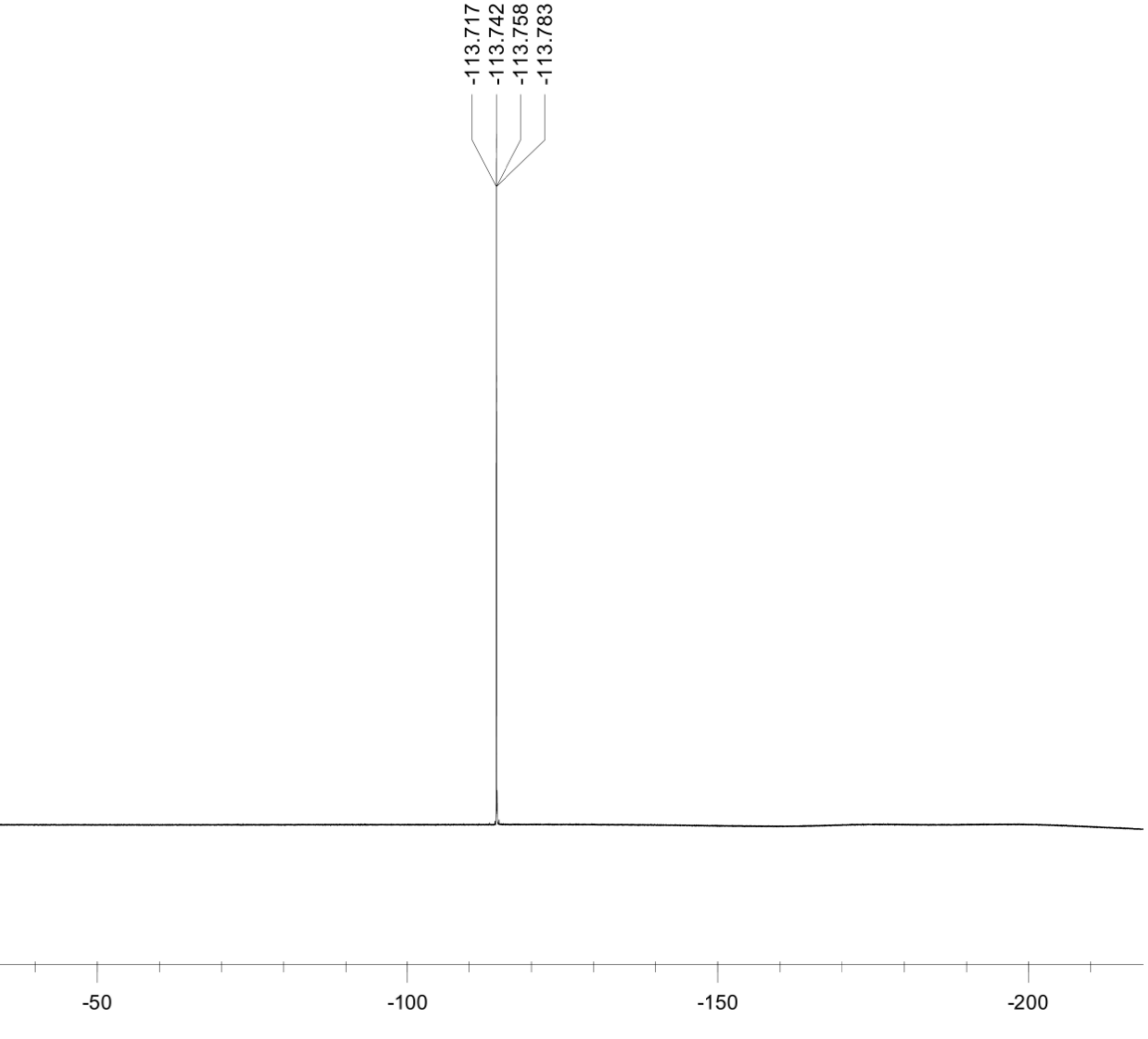

Supplementary Figure 75. ^{19}F NMR spectrum of **3d** (376 MHz, $CDCl_3$)

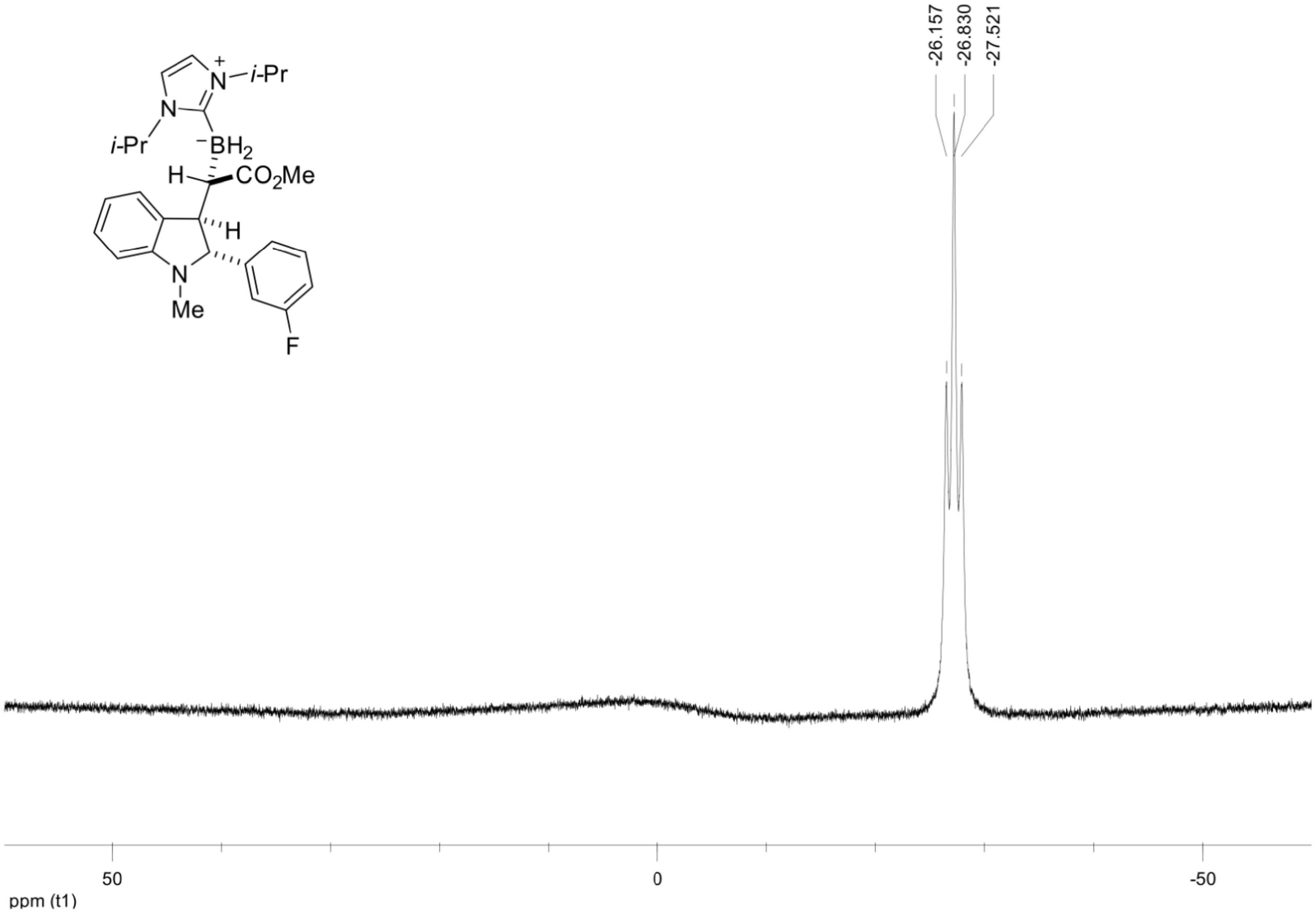

Supplementary Figure 76. ^{11}B NMR spectrum of **3d** (128.4 MHz, $CDCl_3$)

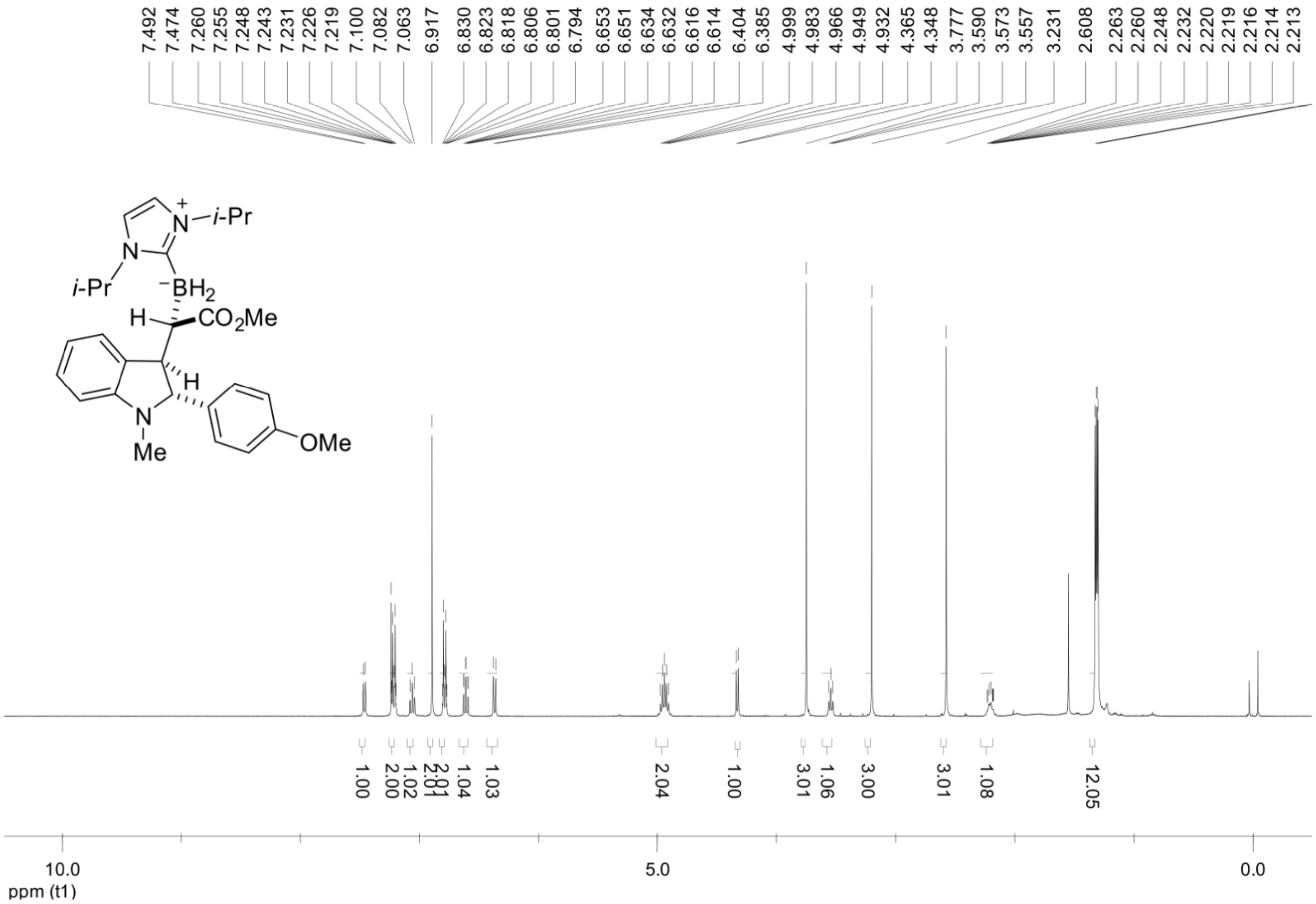

Supplementary Figure 77. ^{1}H NMR spectrum of **3e** (400 MHz, $CDCl_3$)

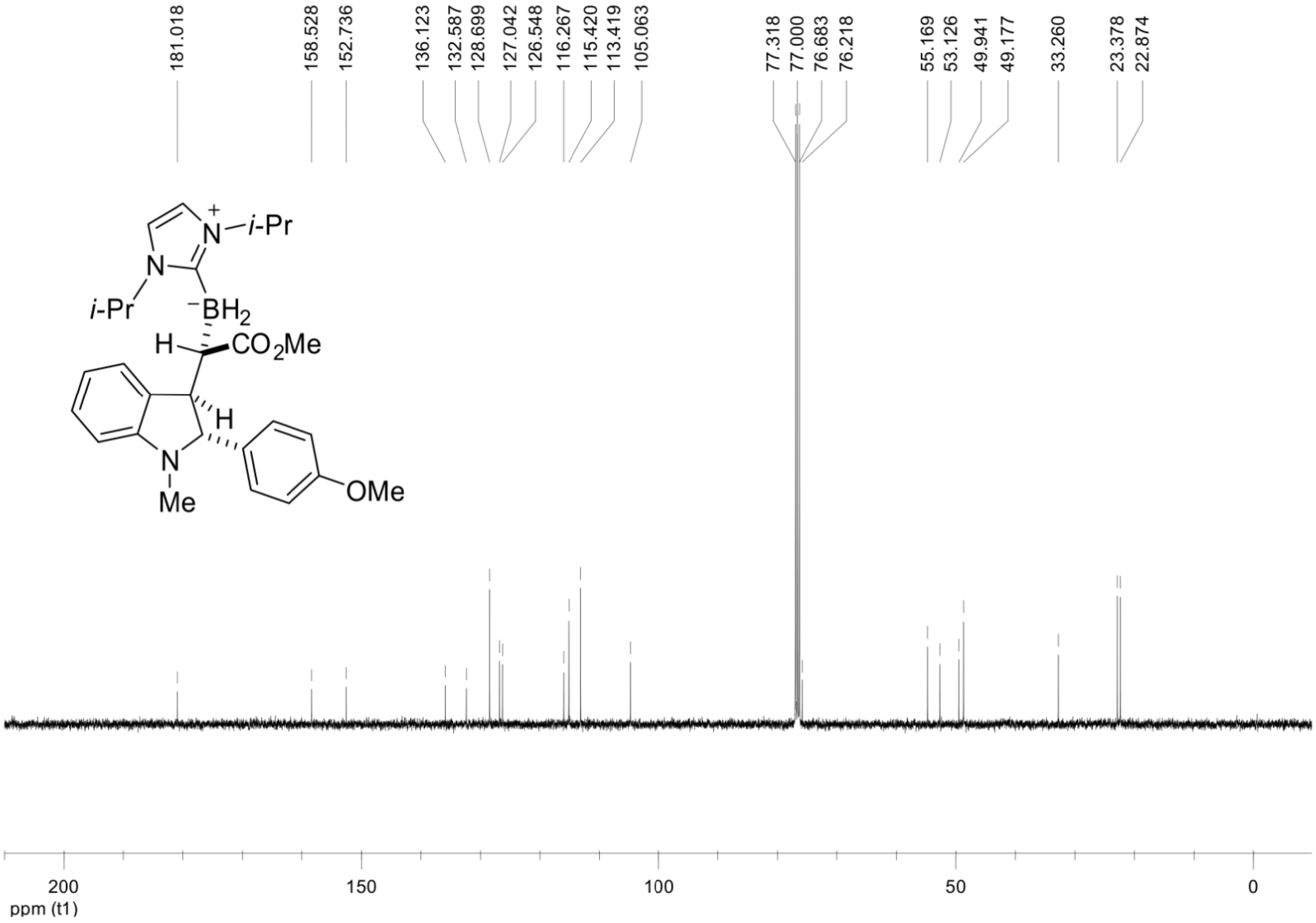

Supplementary Figure 78. ^{13}C NMR spectrum of **3e** (100 MHz, $CDCl_3$)

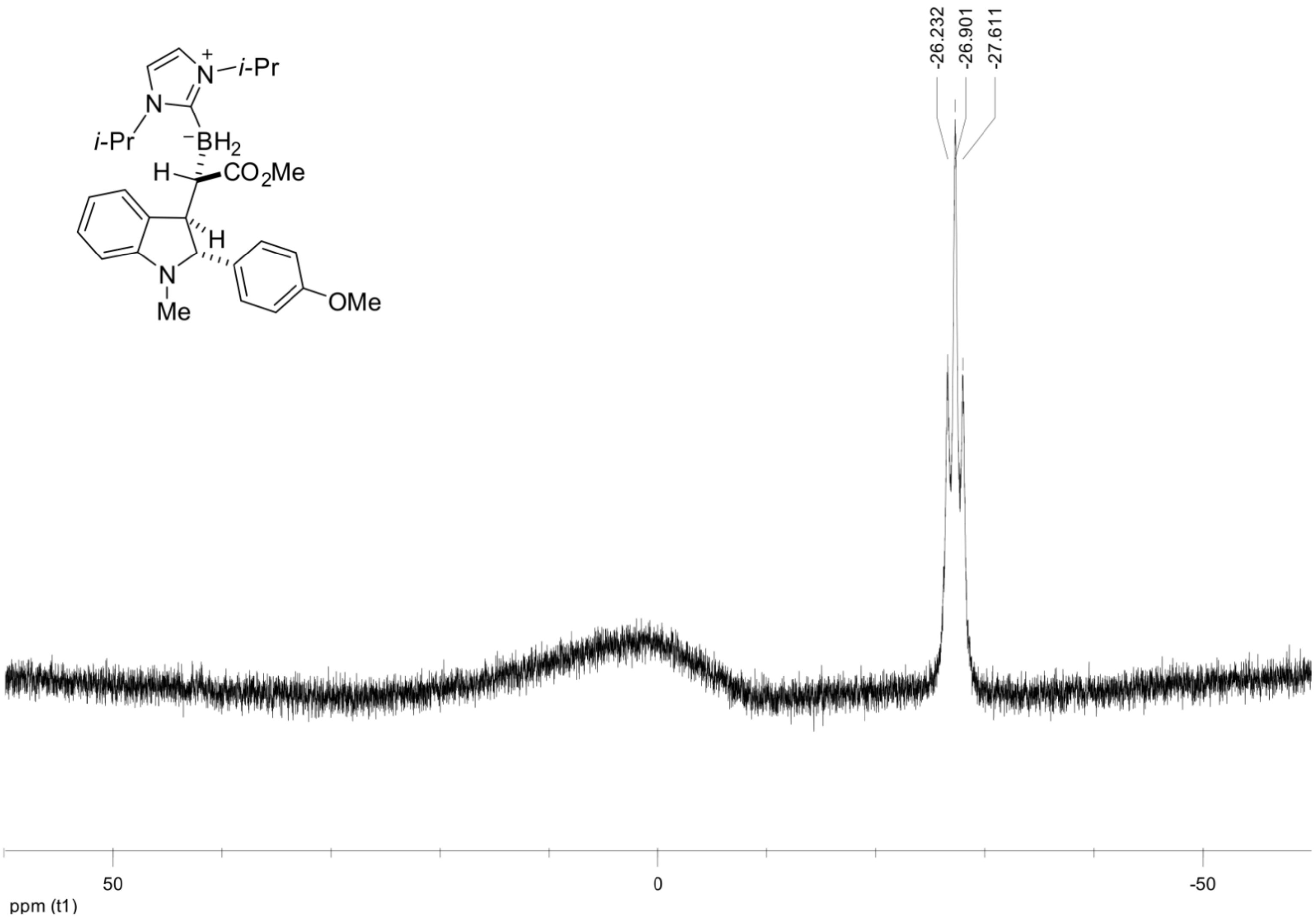

Supplementary Figure 79. ^{11}B NMR spectrum of **3e** (128.4 MHz, $CDCl_3$)

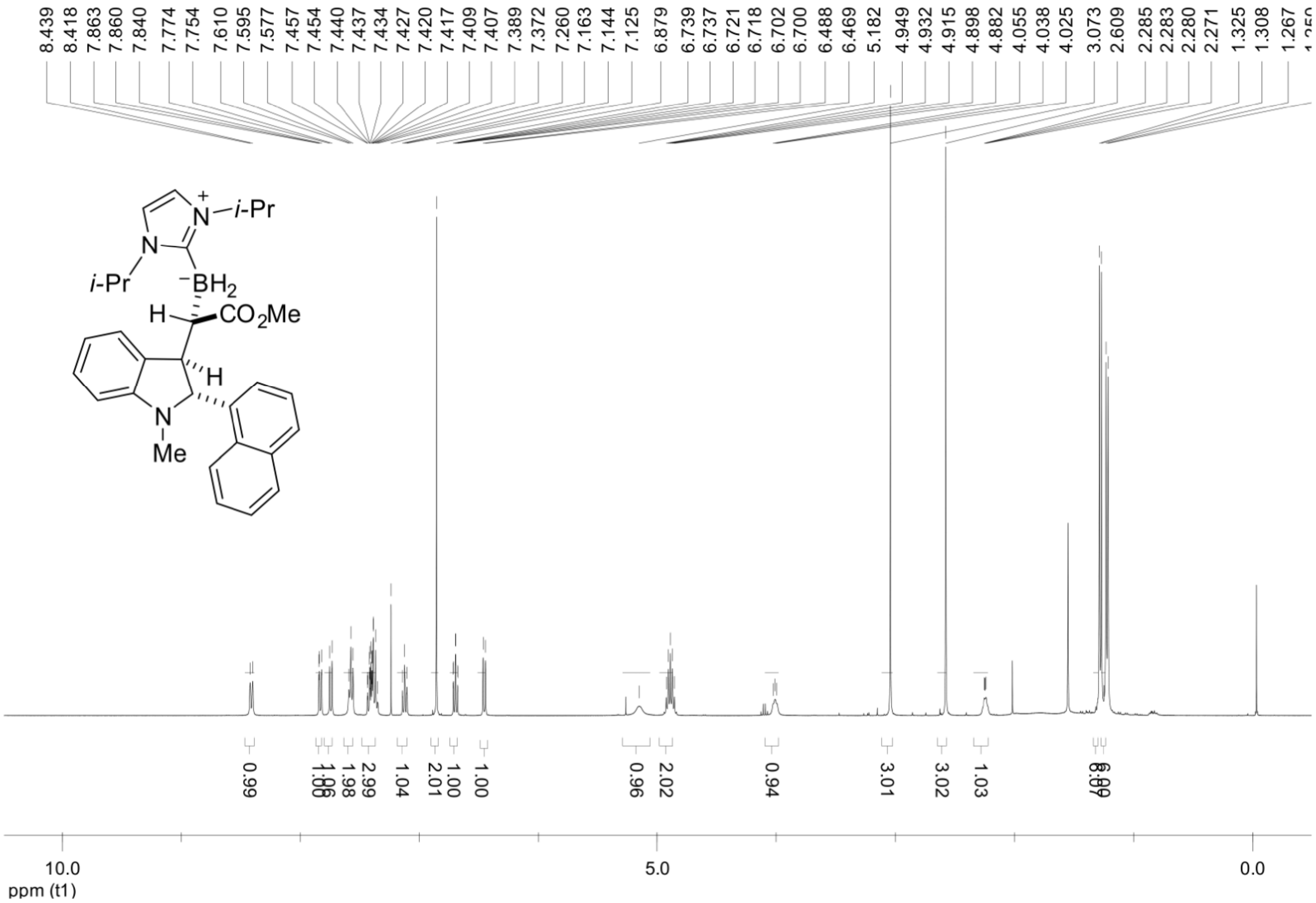

Supplementary Figure 80. ^{1}H NMR spectrum of **3f** (400 MHz, $CDCl_3$)

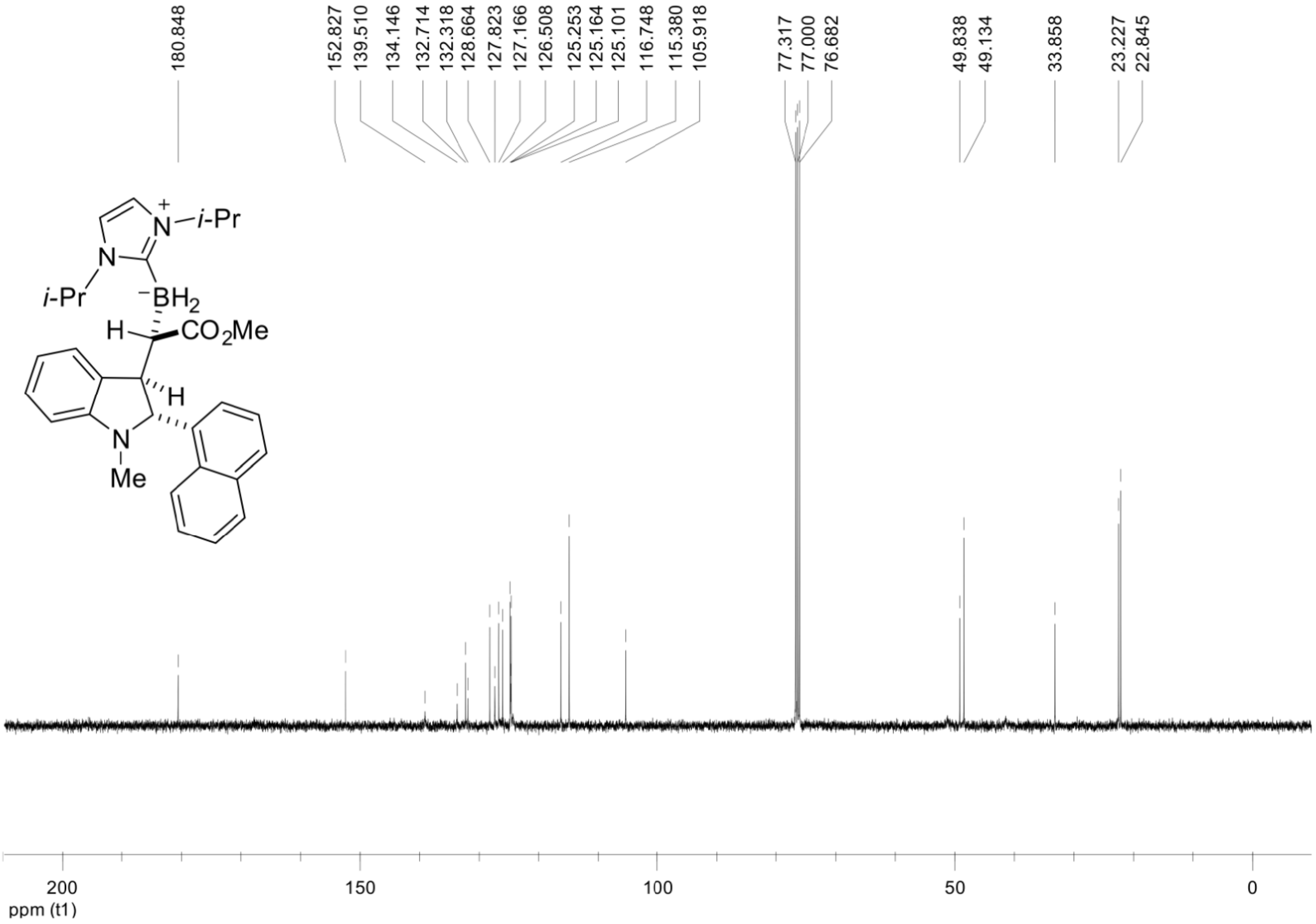

Supplementary Figure 81. ^{13}C NMR spectrum of **3f** (100 MHz, $CDCl_3$)

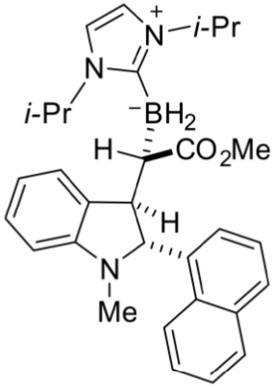

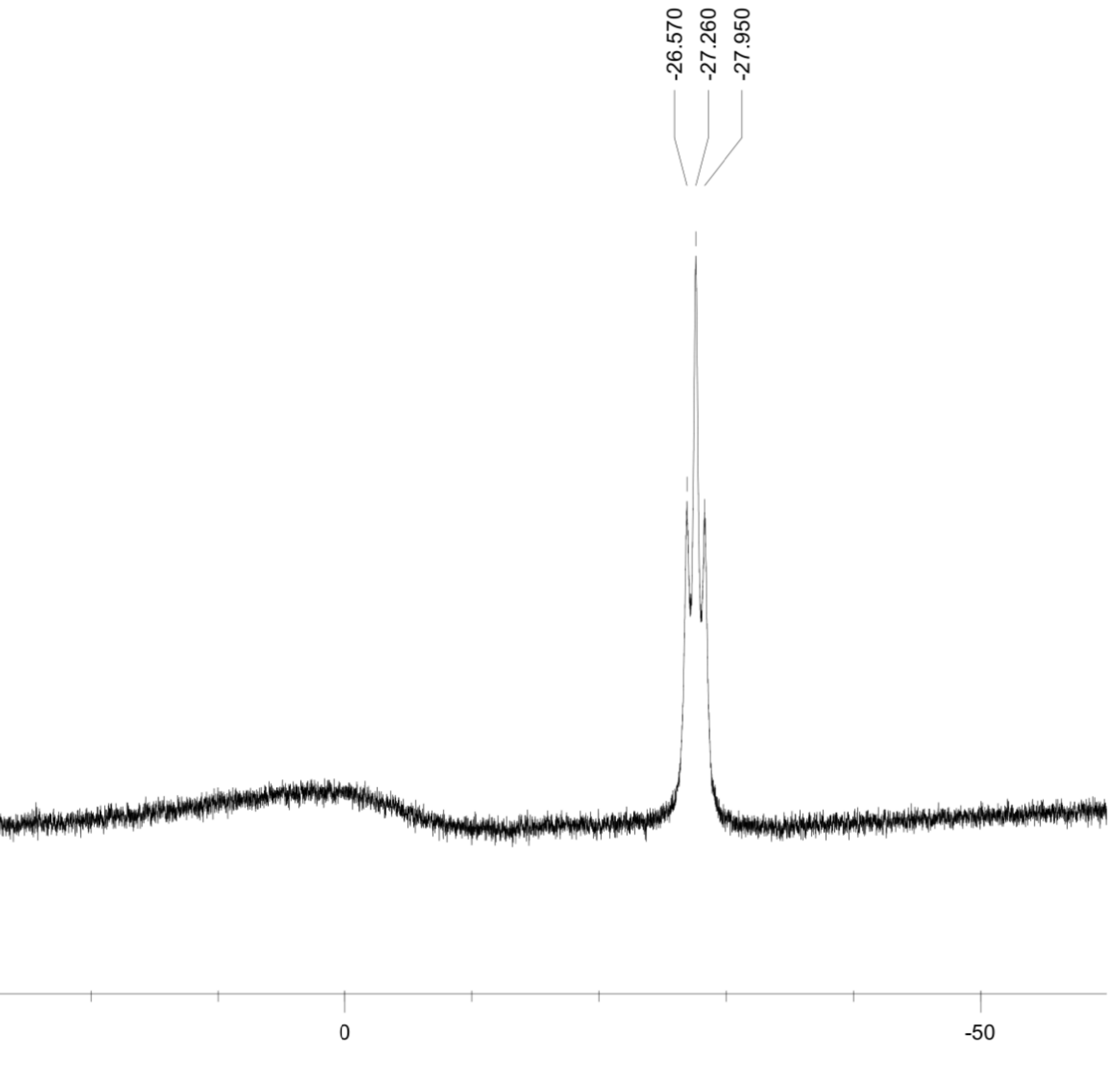

Supplementary Figure 82. ^{11}B NMR spectrum of **3f** (128.4 MHz, $CDCl_3$)

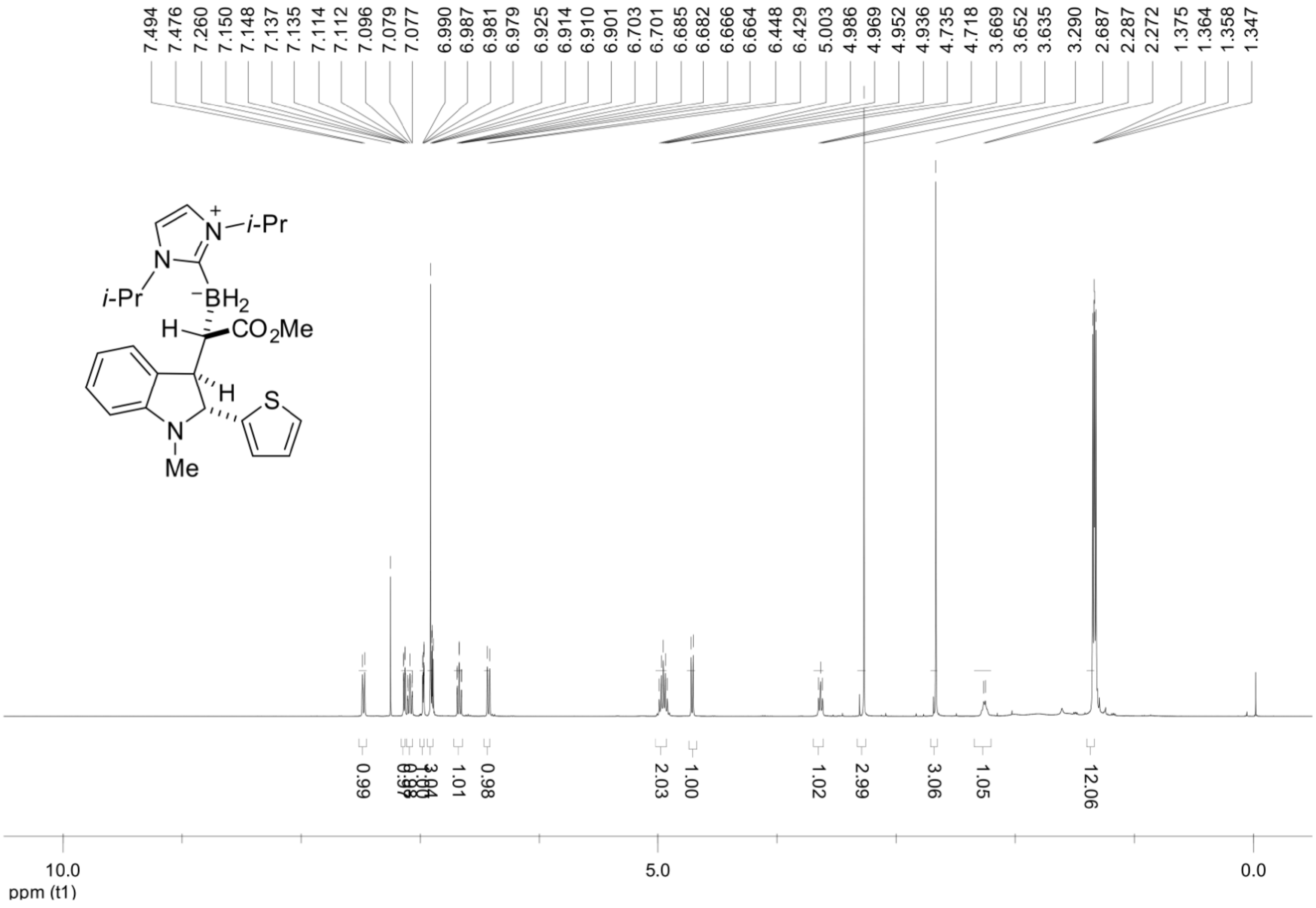

Supplementary Figure 83. ^{1}H NMR spectrum of **3g** (400 MHz, $CDCl_3$)

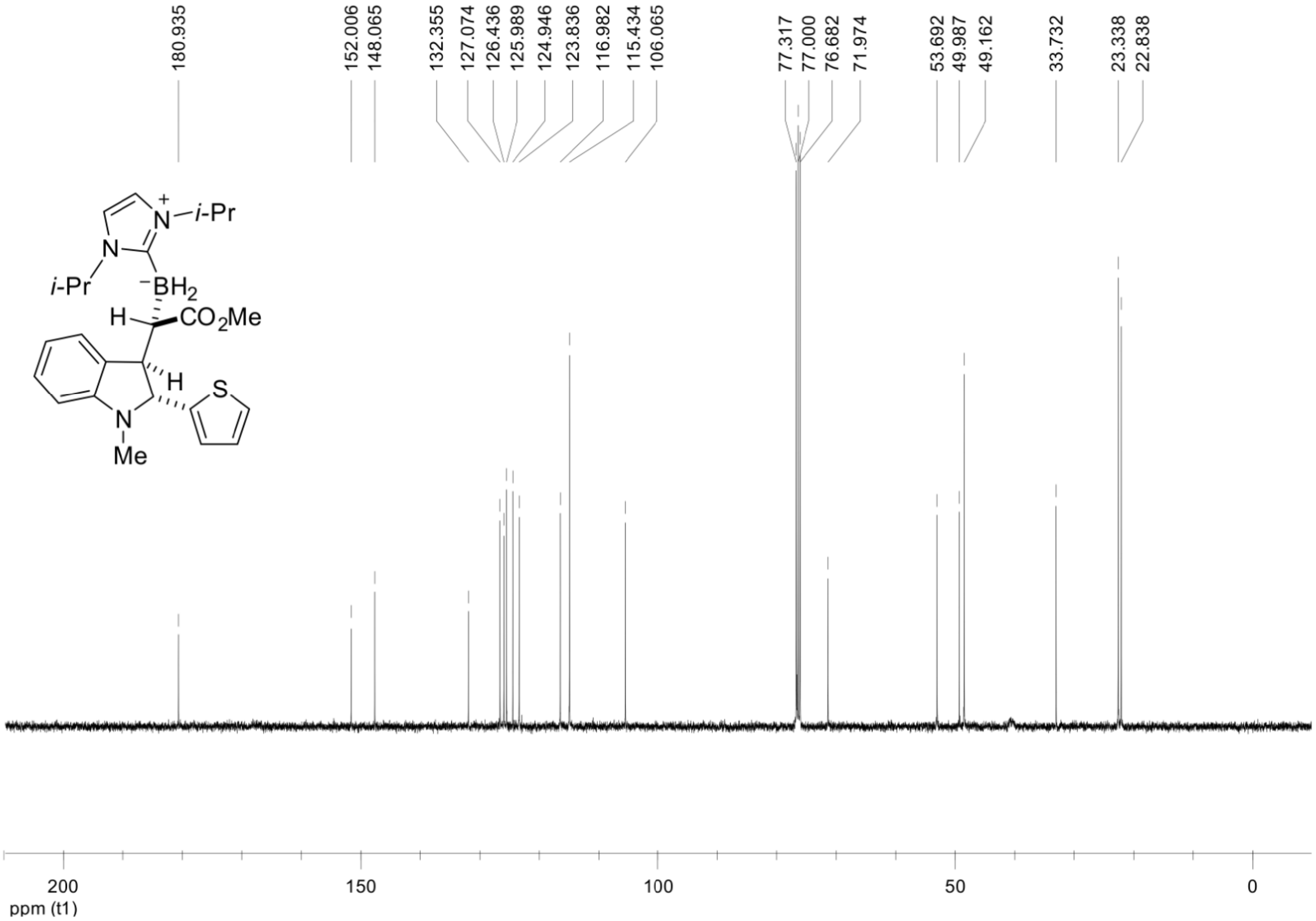

Supplementary Figure 84. ^{13}C NMR spectrum of **3g** (100 MHz, $CDCl_3$)

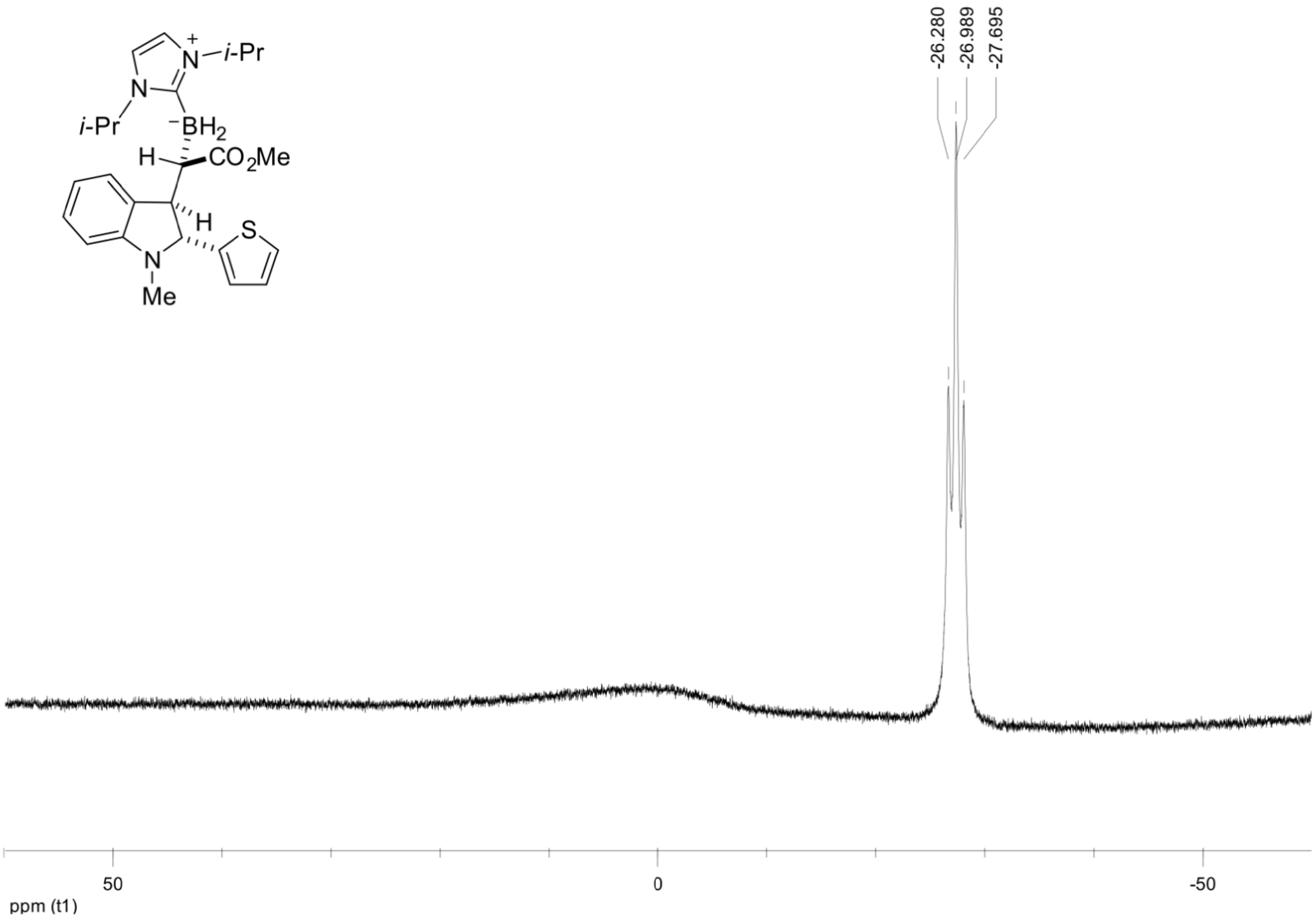

Supplementary Figure 85. ^{11}B NMR spectrum of **3g** (128.4 MHz, $CDCl_3$)

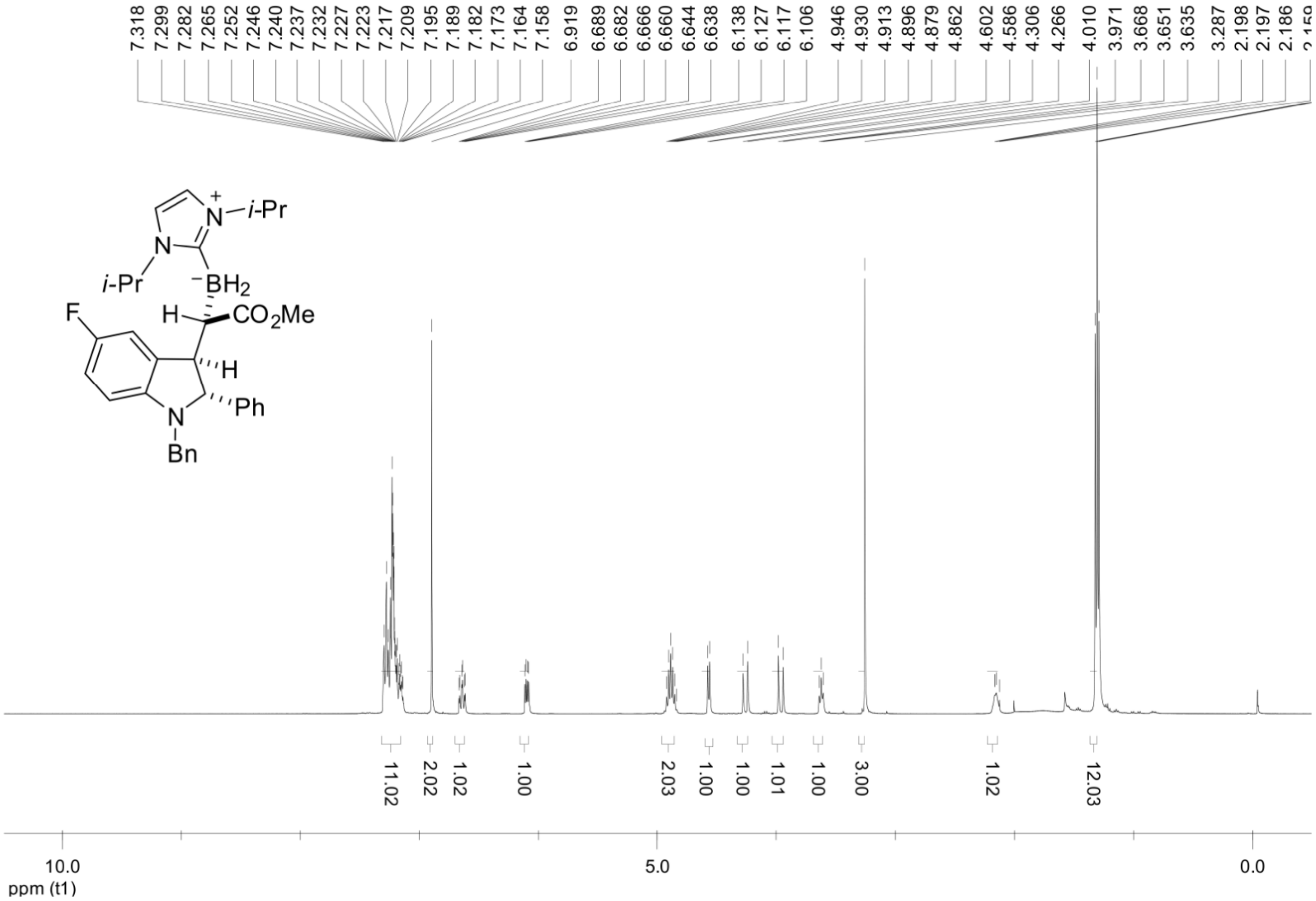

Supplementary Figure 86. ^{1}H NMR spectrum of **3h** (400 MHz, $CDCl_3$)

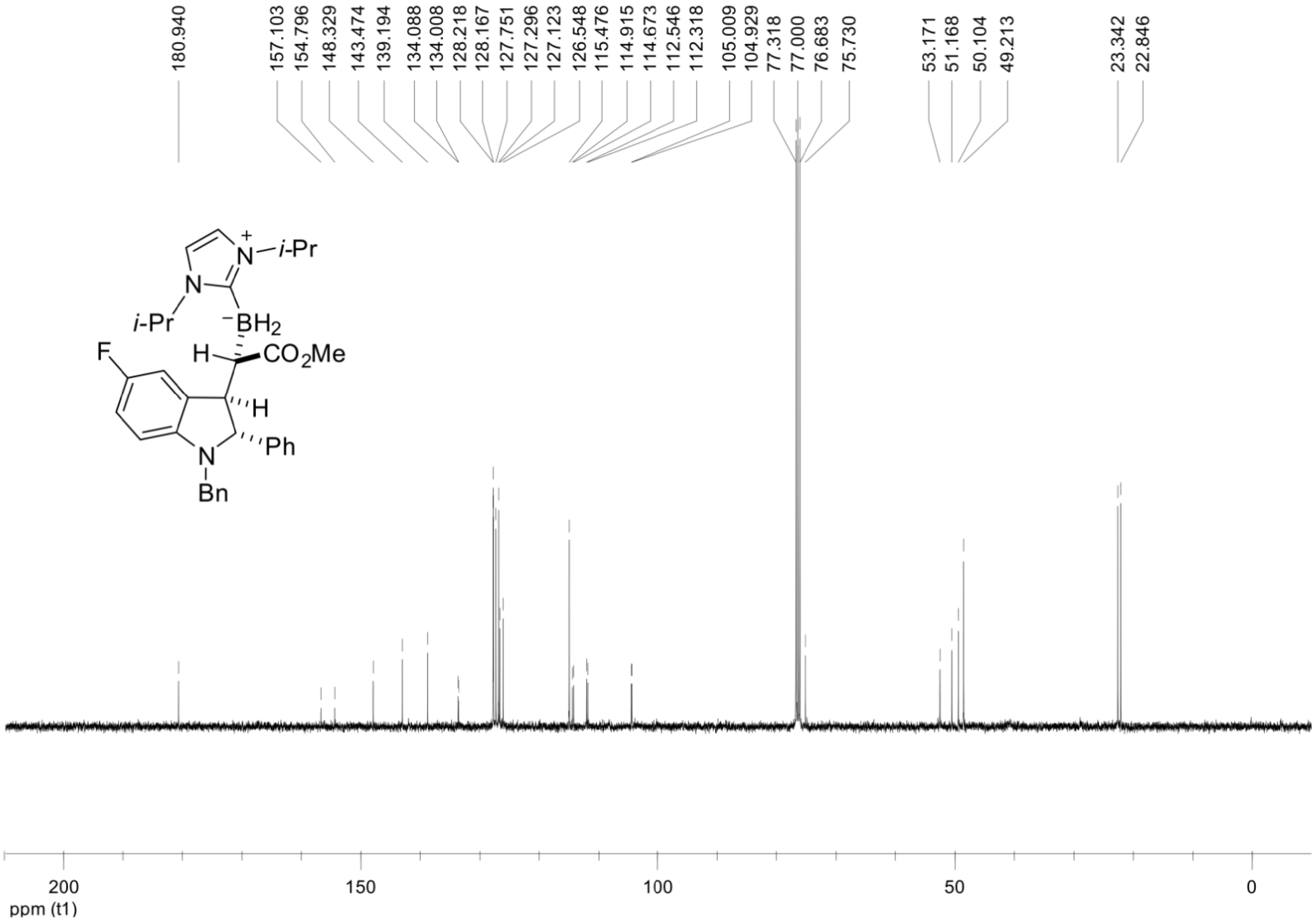

Supplementary Figure 87. ^{13}C NMR spectrum of **3h** (100 MHz, $CDCl_3$)

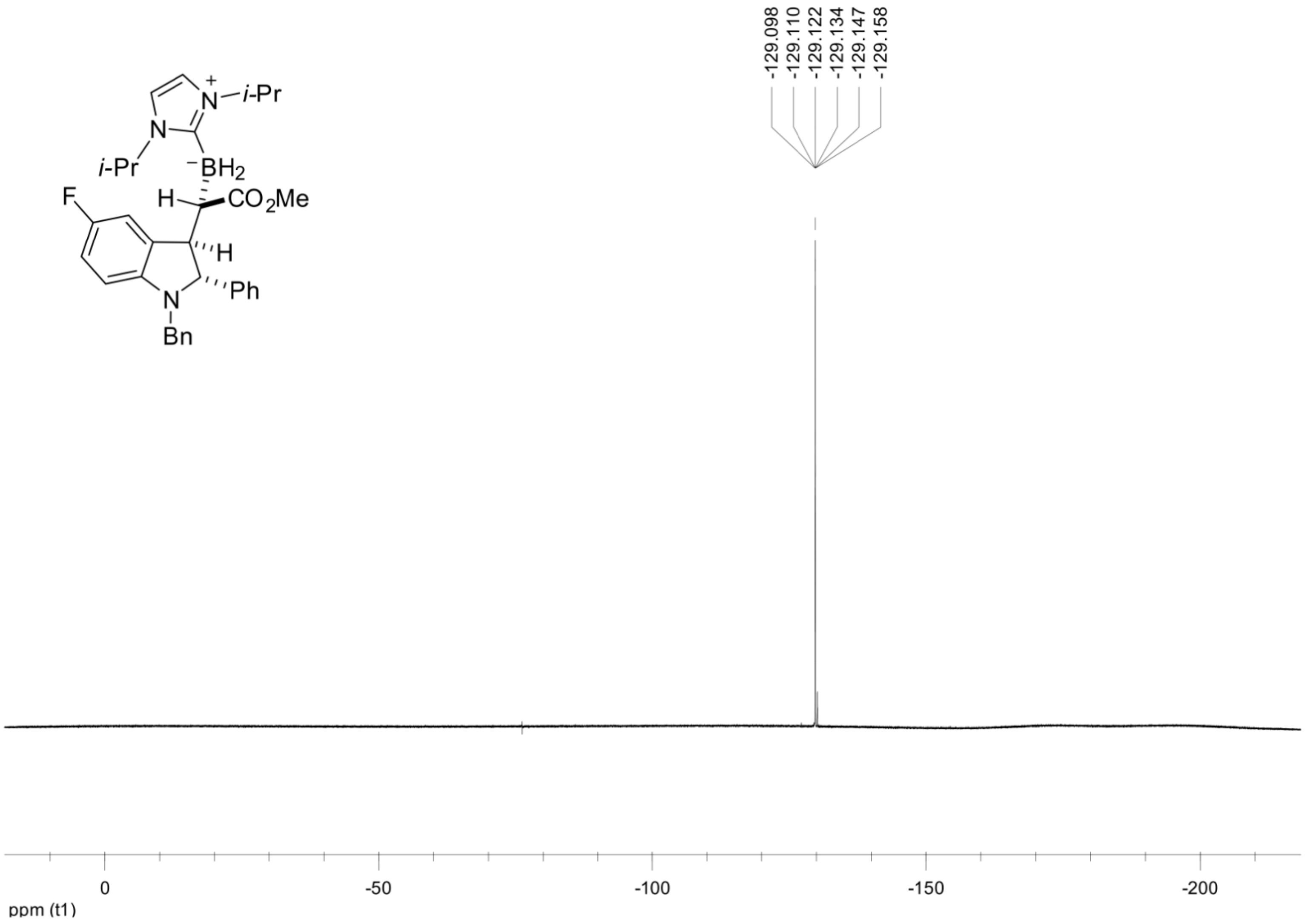

Supplementary Figure 88. ^{19}F NMR spectrum of **3h** (376 MHz, $CDCl_3$)

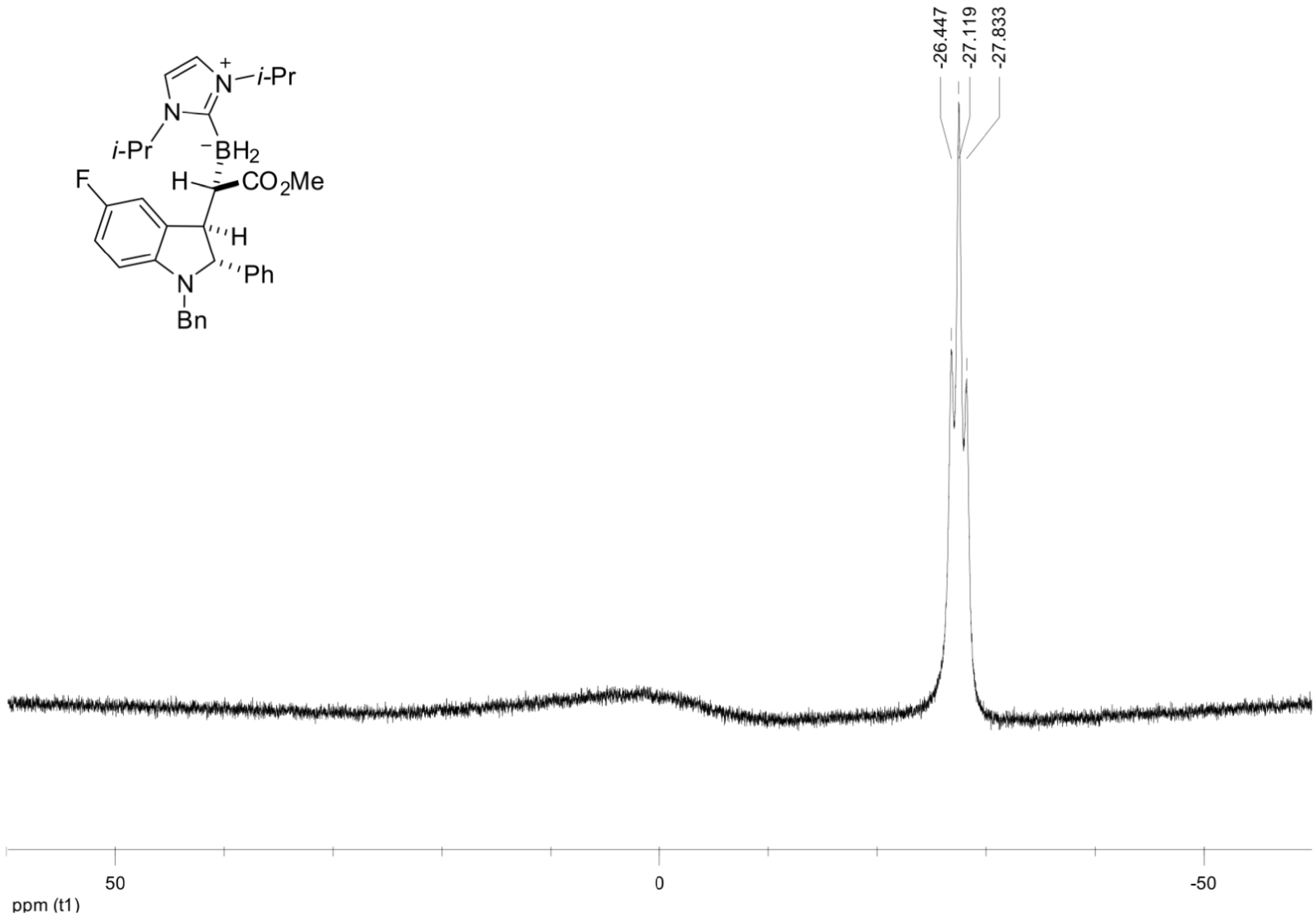

Supplementary Figure 89. ^{11}B NMR spectrum of **3h** (128.4 MHz, $CDCl_3$)

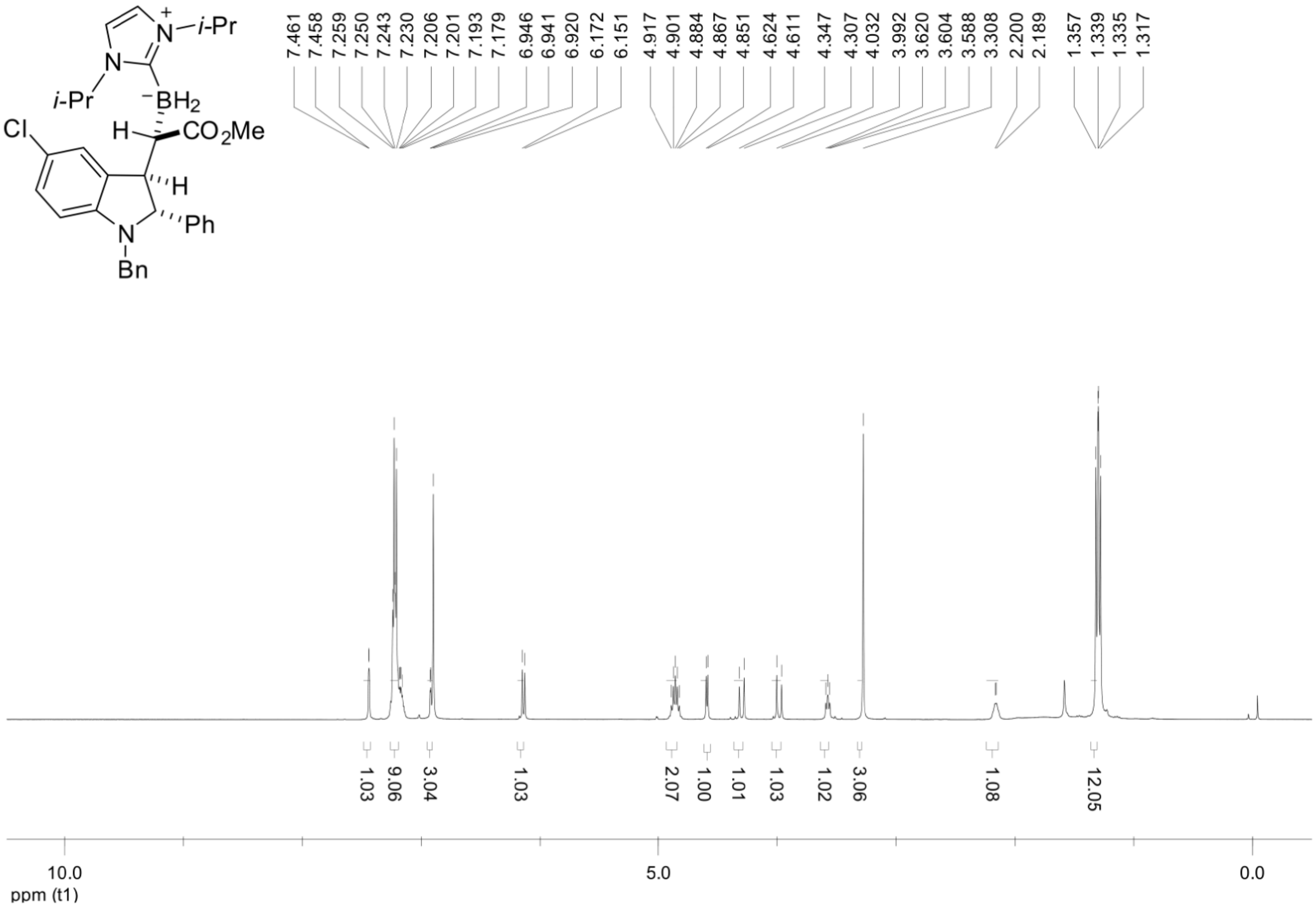

Supplementary Figure 90. ^{1}H NMR spectrum of **3i** (400 MHz, $CDCl_3$)

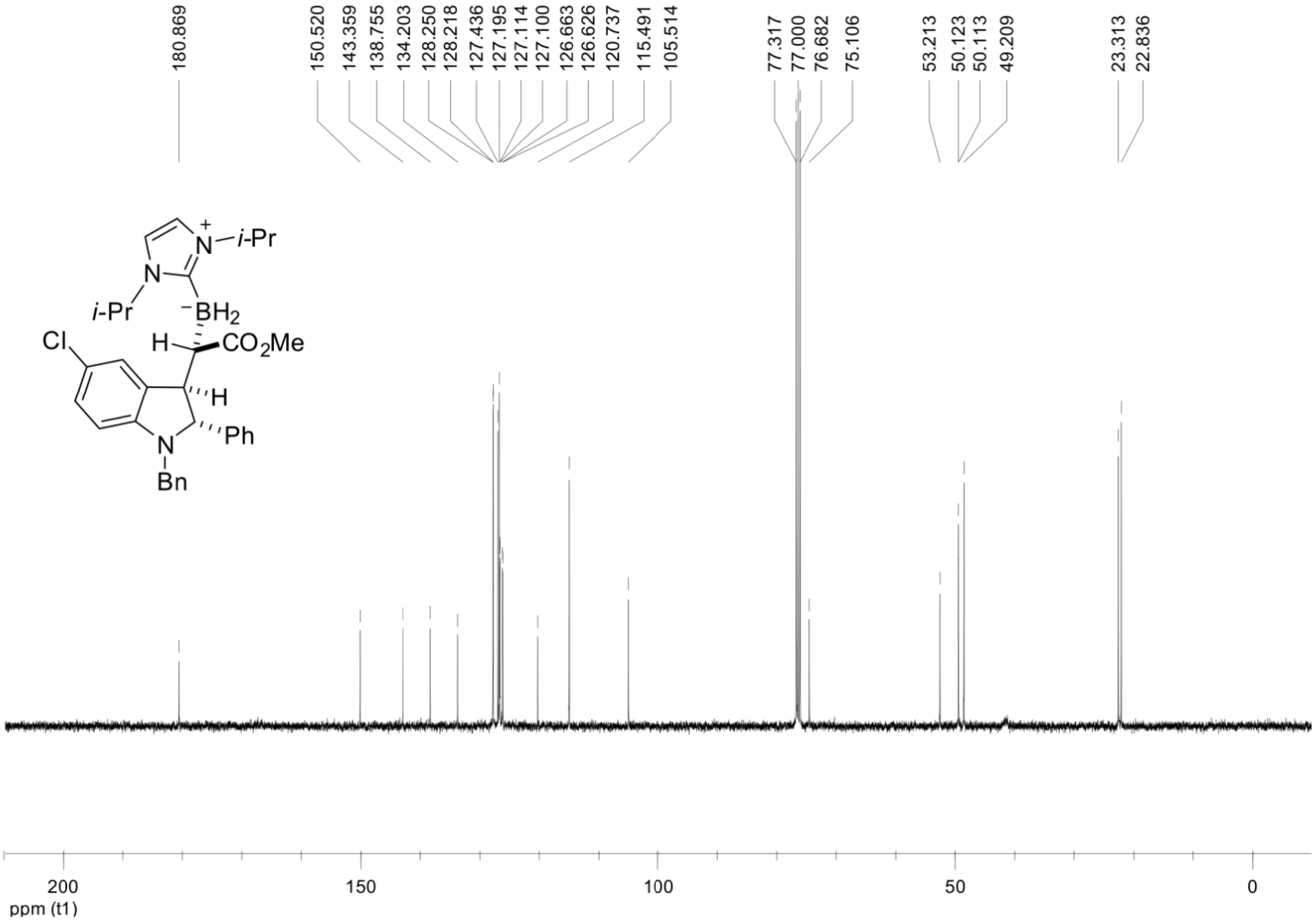

Supplementary Figure 91. ^{13}C NMR spectrum of **3i** (100 MHz, $CDCl_3$)

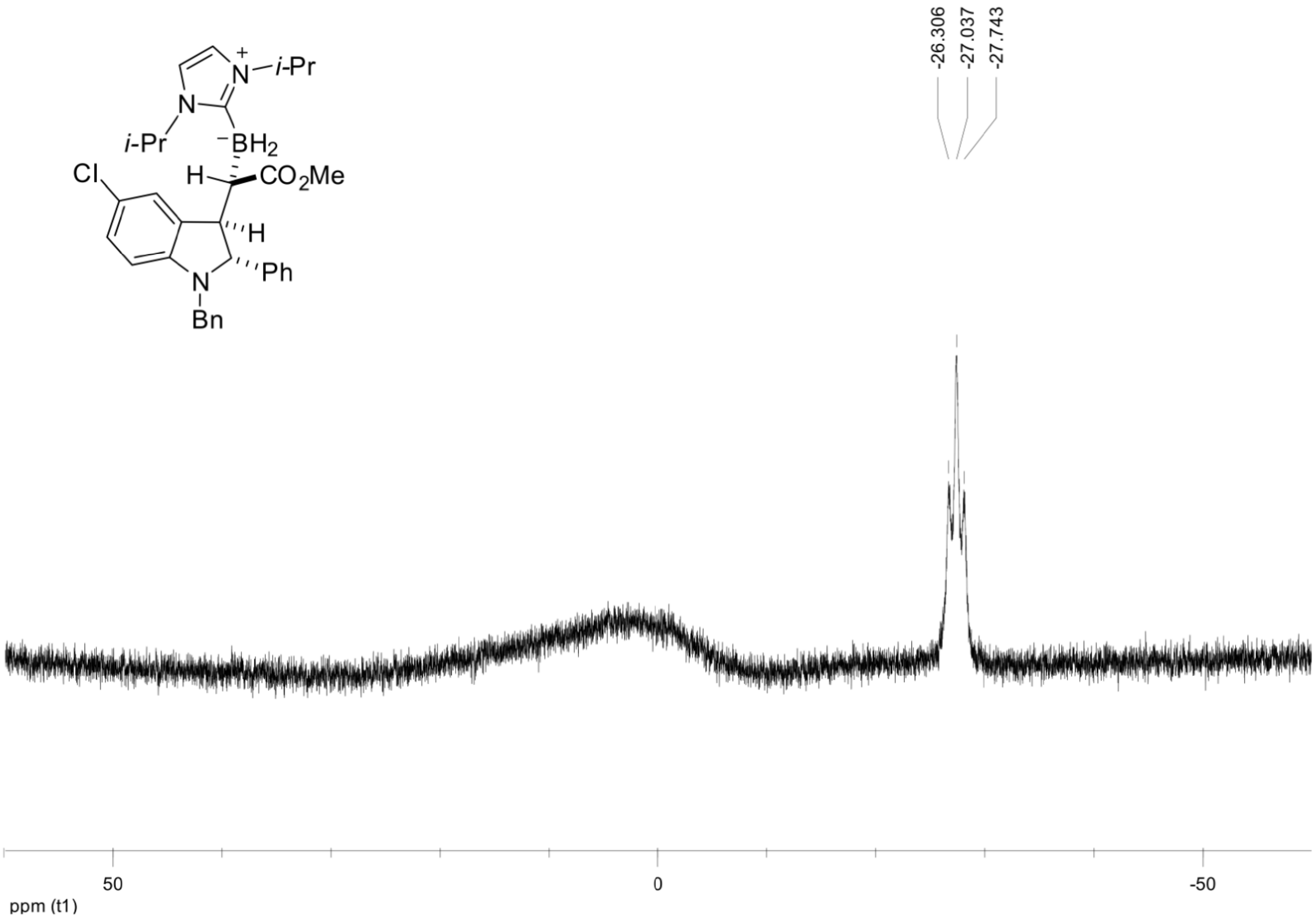

Supplementary Figure 92. ^{11}B NMR spectrum of **3i** (128.4 MHz, $CDCl_3$)

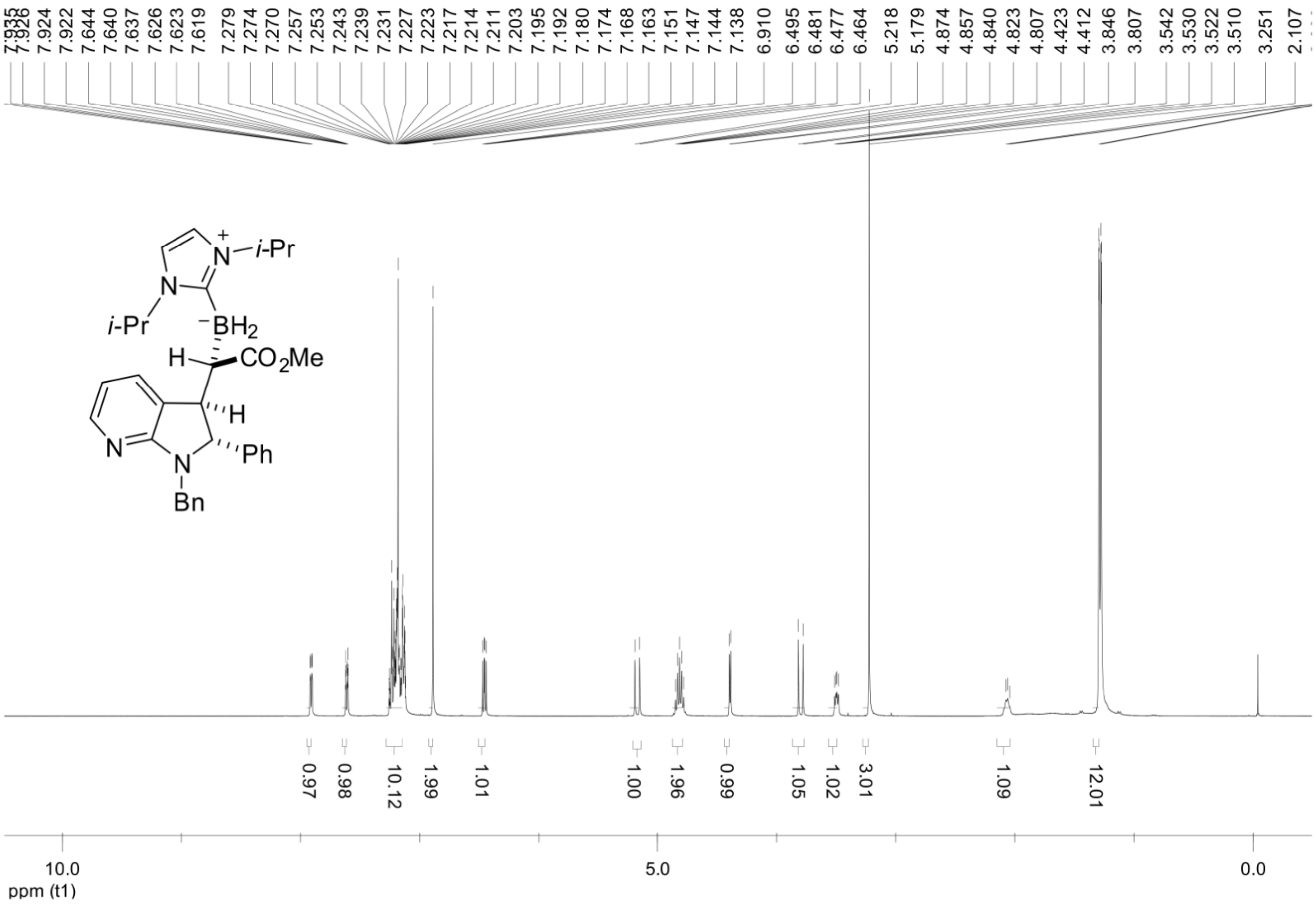

Supplementary Figure 93. ^{1}H NMR spectrum of **3j** (400 MHz, $CDCl_3$)

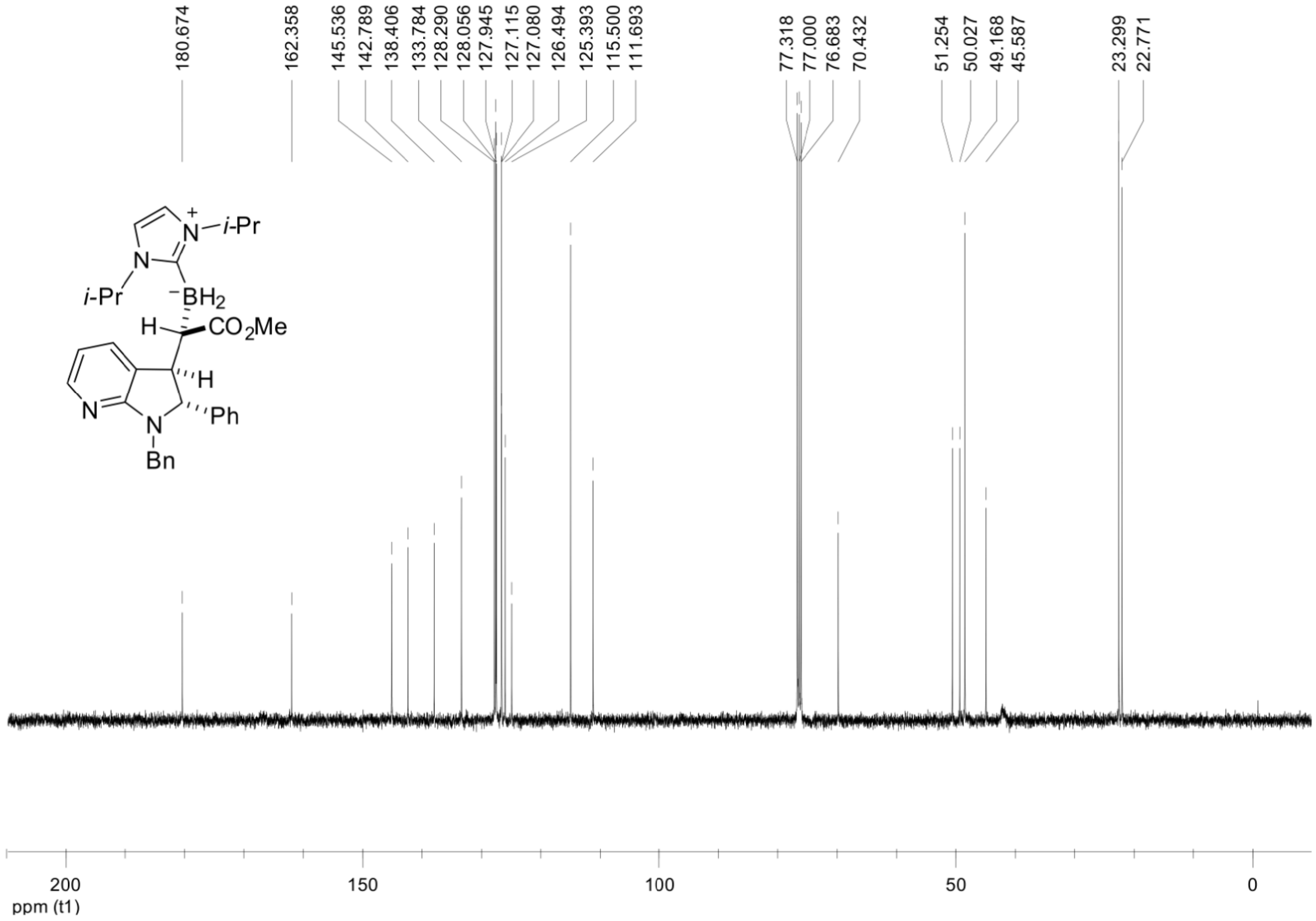

Supplementary Figure 94. ^{13}C NMR spectrum of **3j** (100 MHz, $CDCl_3$)

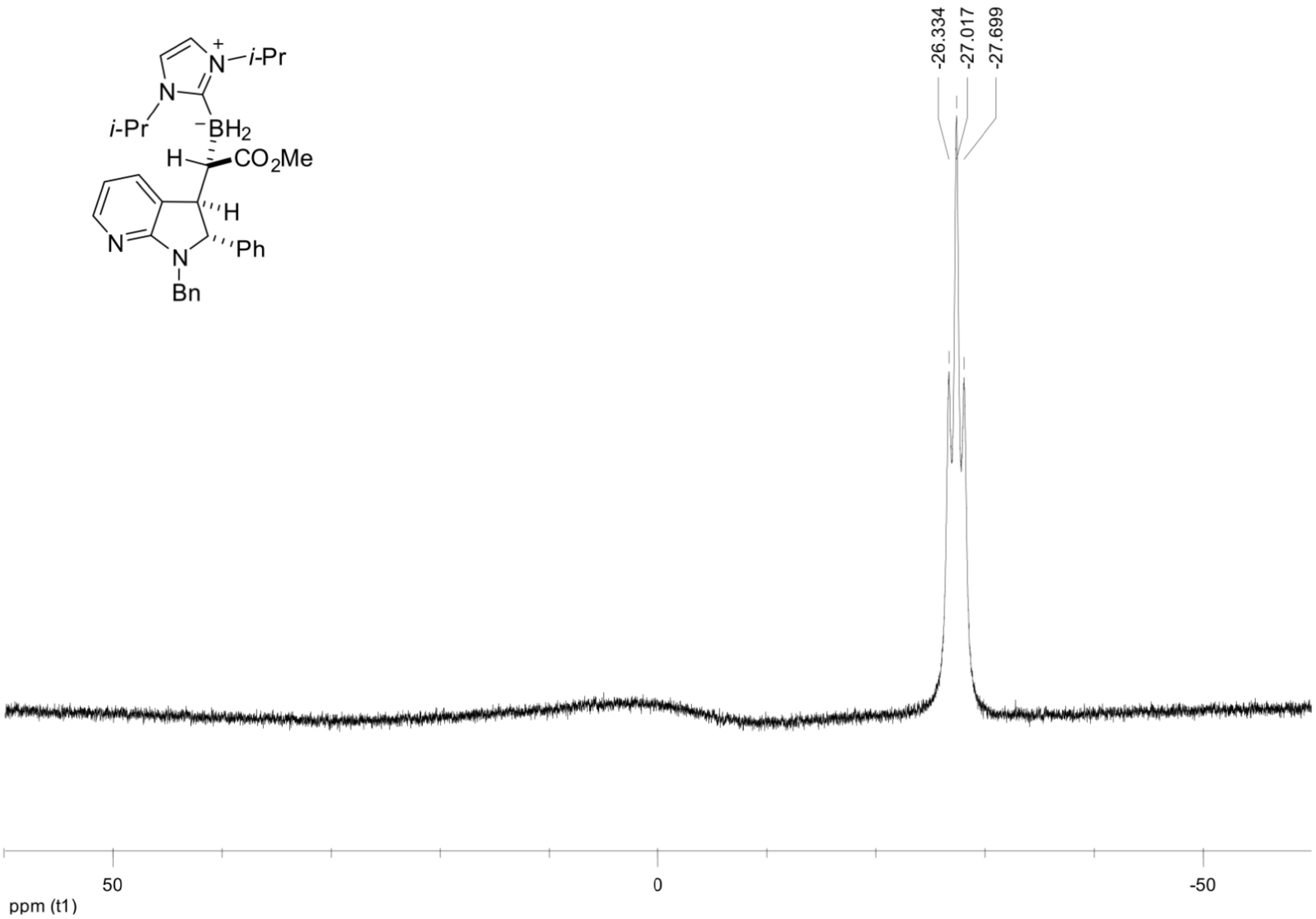

Supplementary Figure 95. ^{11}B NMR spectrum of **3j** (128.4 MHz, $CDCl_3$)

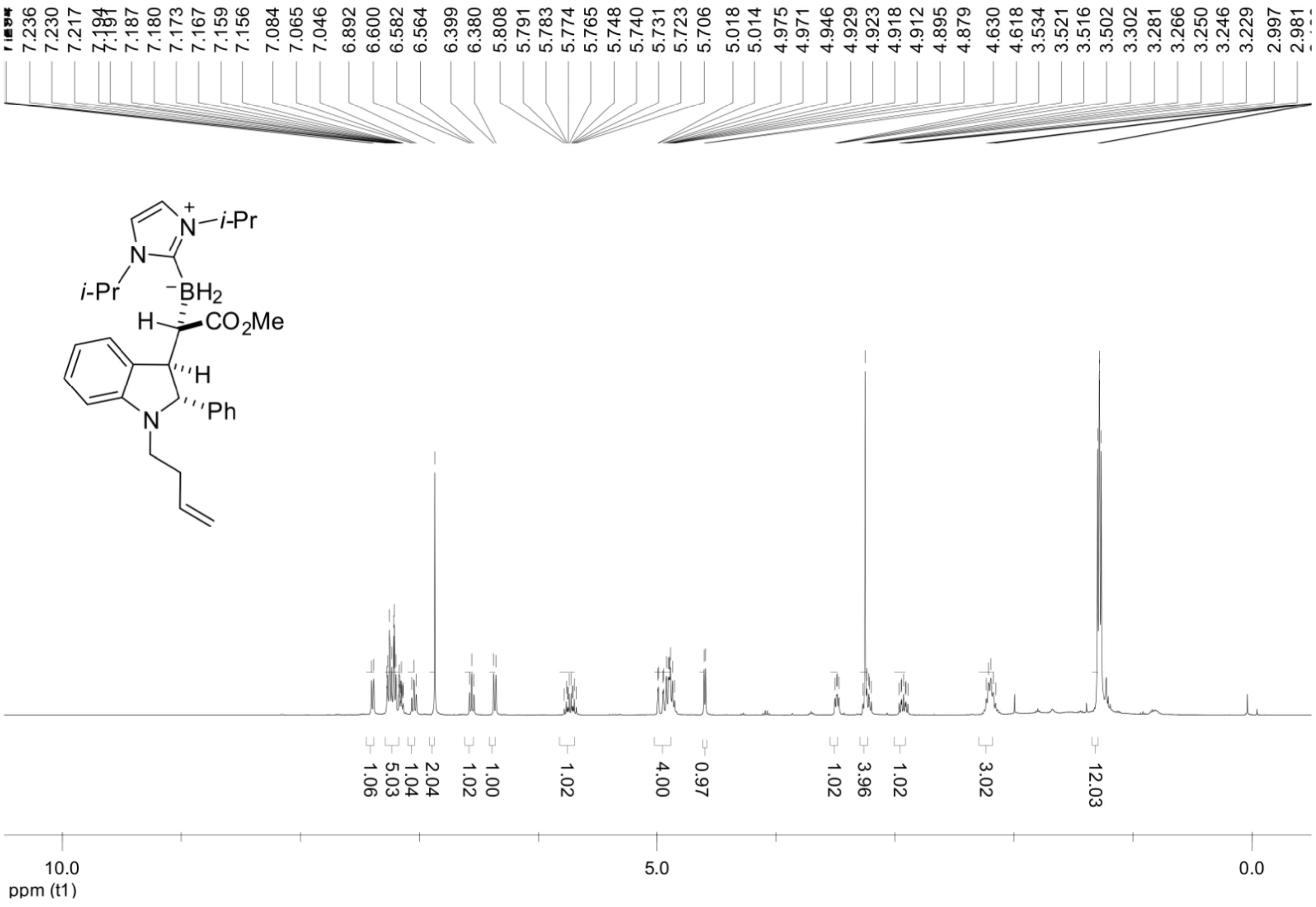

Supplementary Figure 96. ^{1}H NMR spectrum of **3k** (400 MHz, $CDCl_3$)

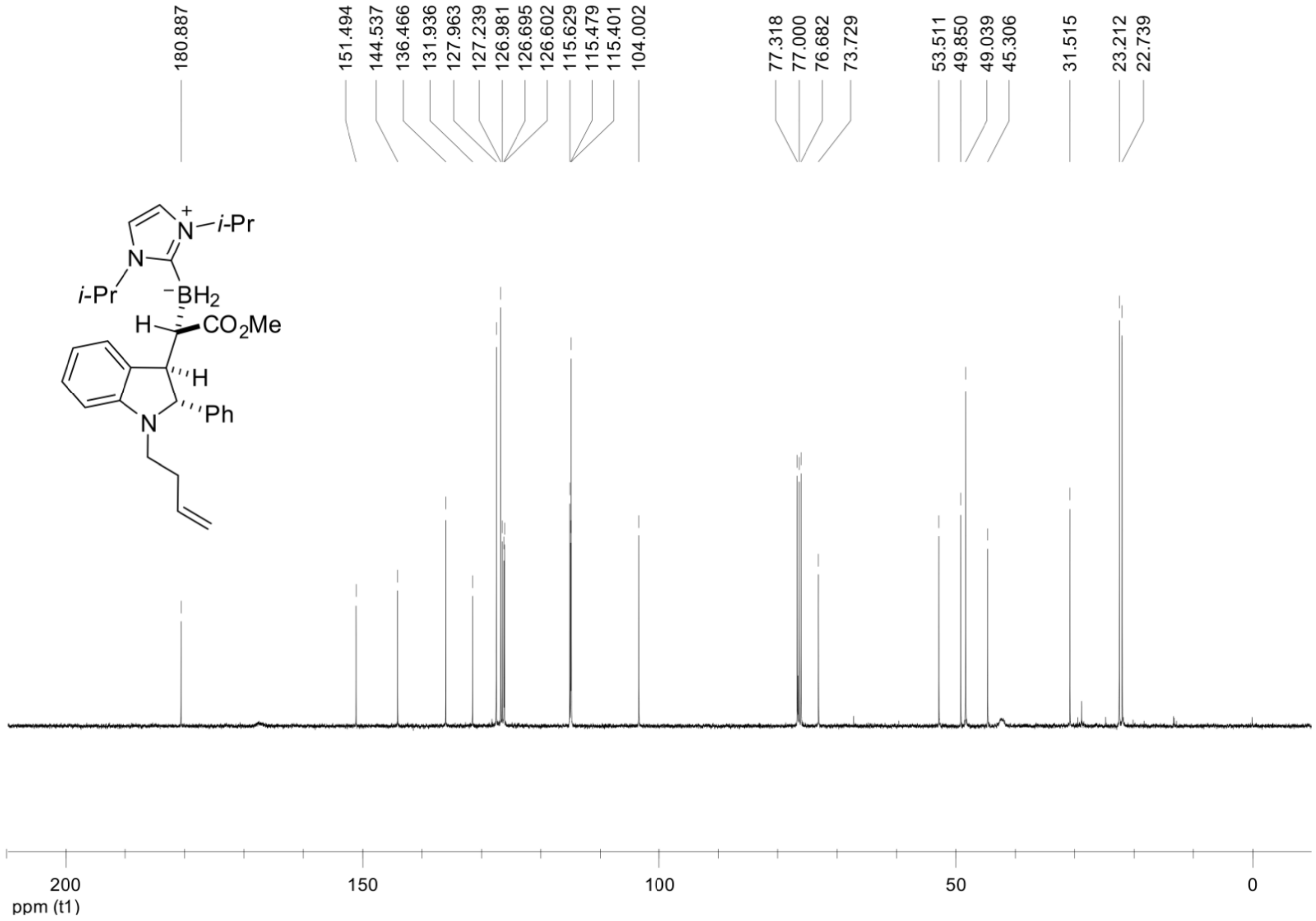

Supplementary Figure 97. ^{13}C NMR spectrum of **3k** (100 MHz, $CDCl_3$)

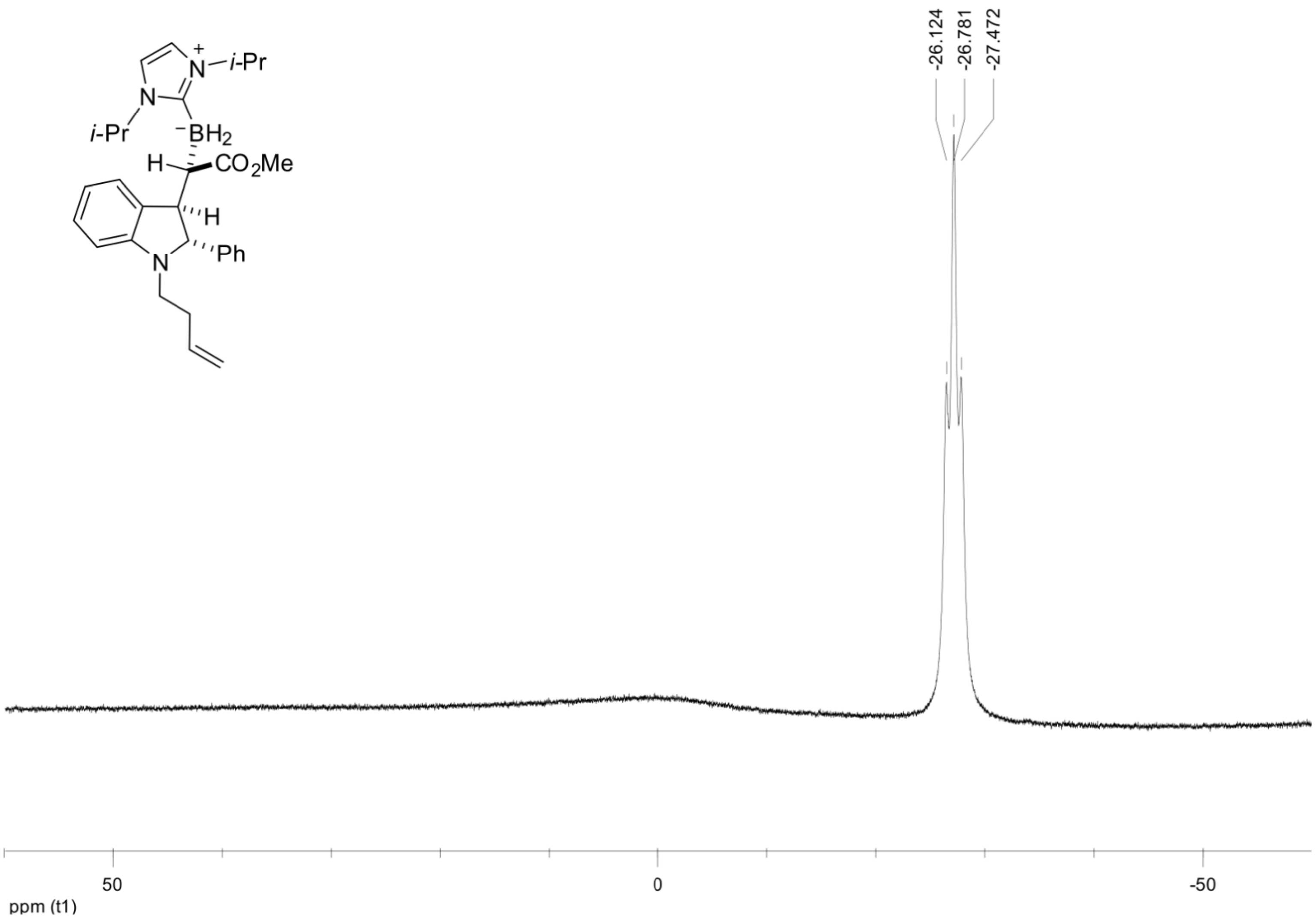

Supplementary Figure 98. ^{11}B NMR spectrum of **3k** (128.4 MHz, $CDCl_3$)

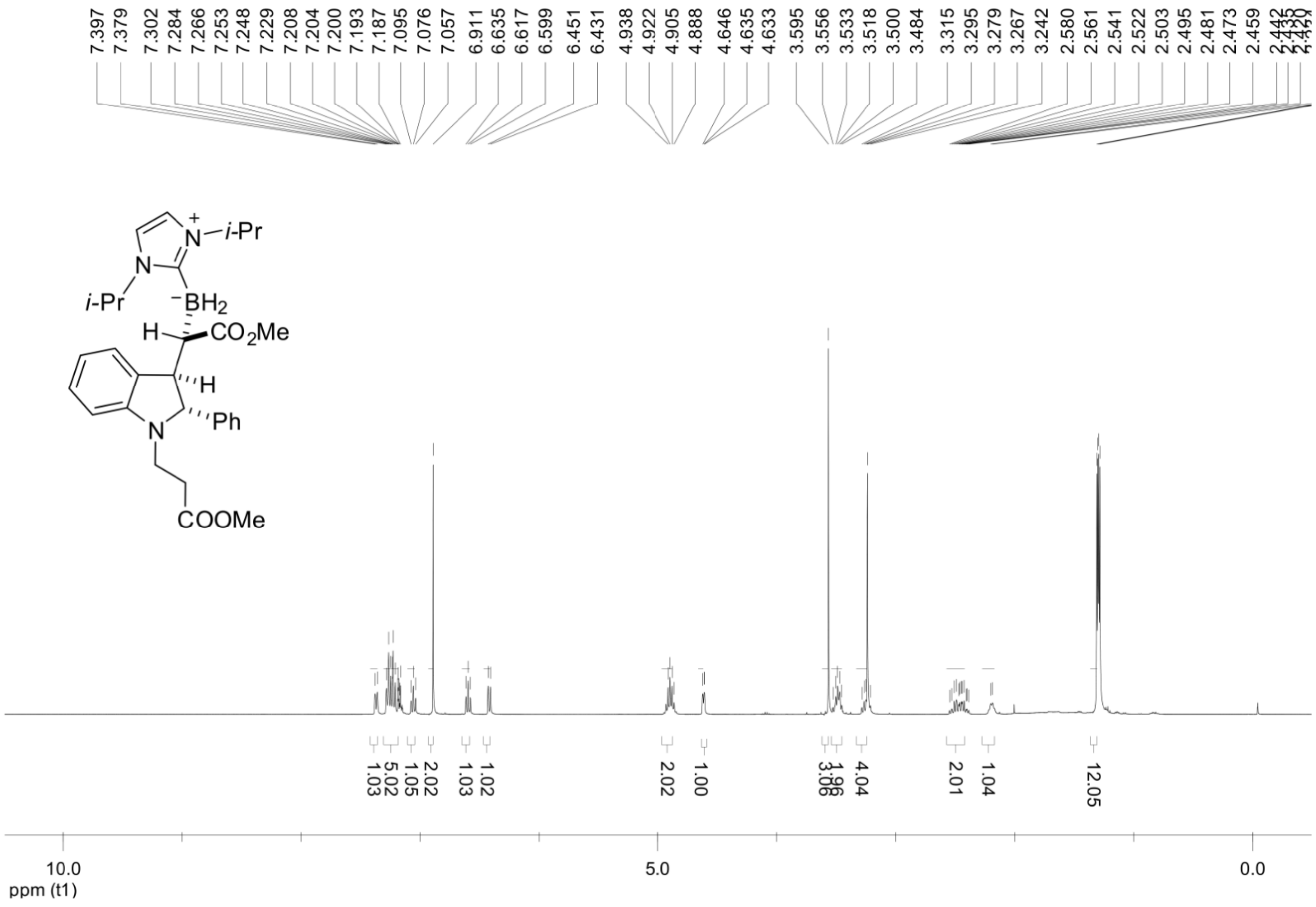

Supplementary Figure 99. ^{1}H NMR spectrum of **3l** (400 MHz, $CDCl_3$)

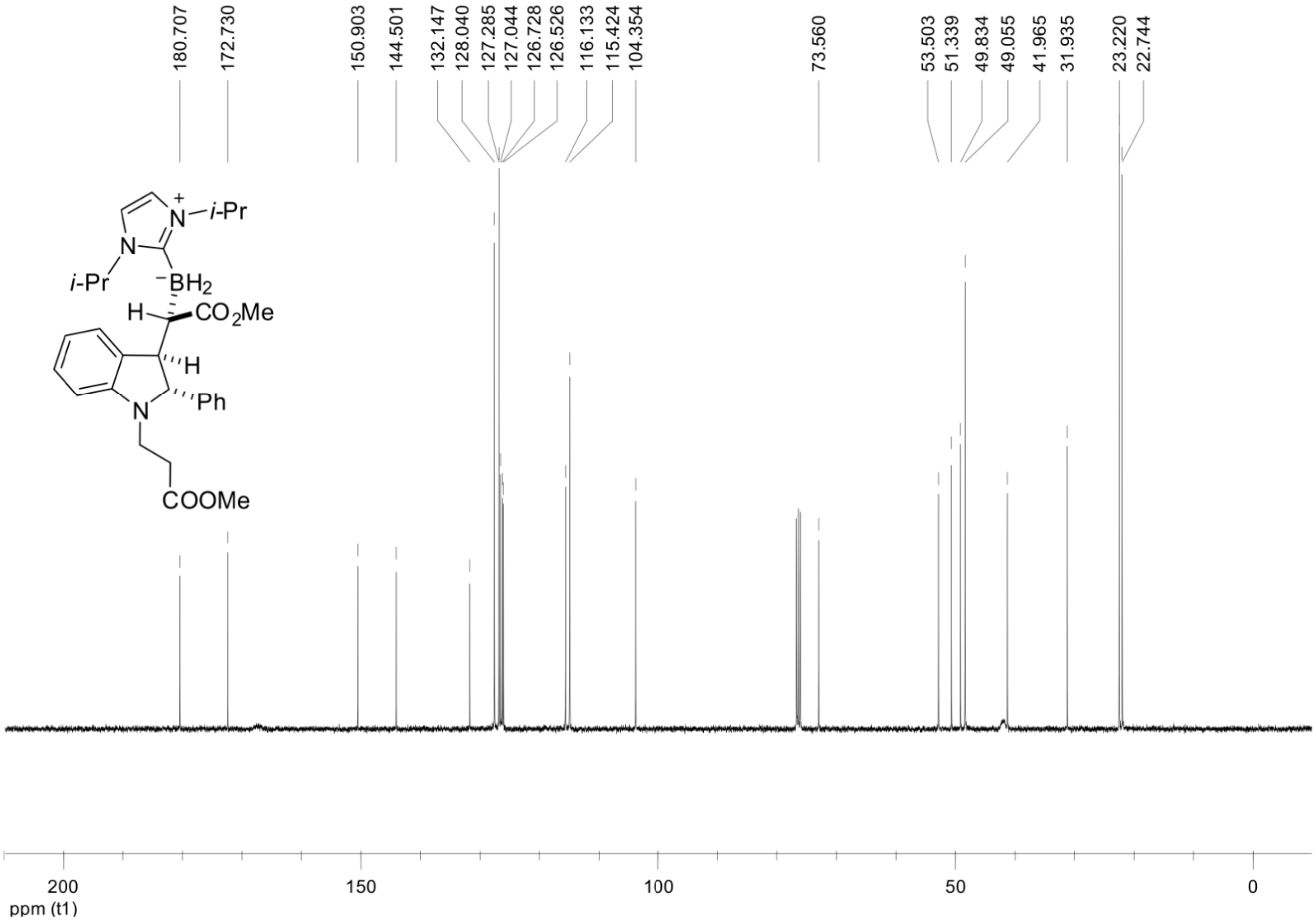

Supplementary Figure 100. ^{13}C NMR spectrum of **3l** (100 MHz, $CDCl_3$)

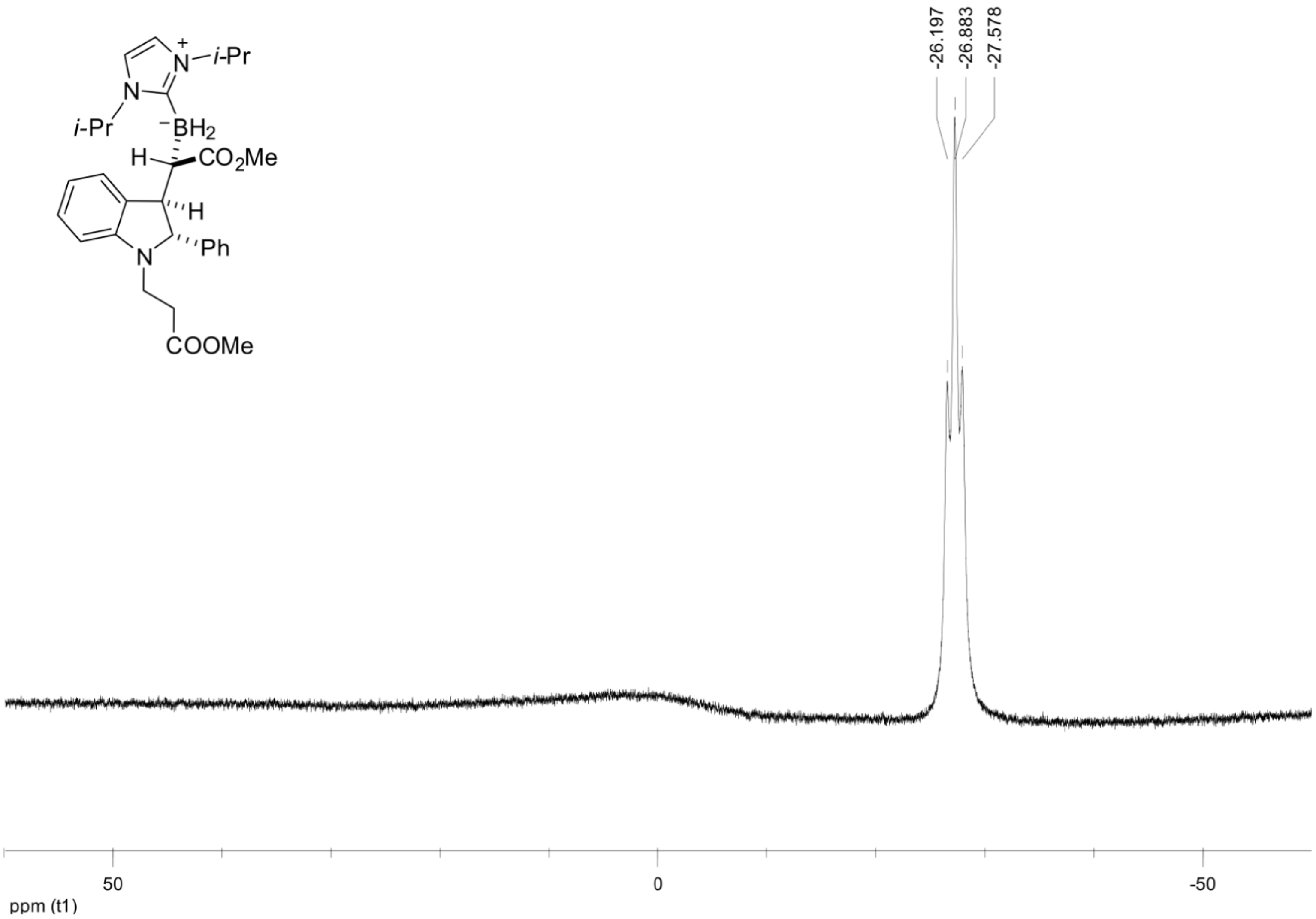

Supplementary Figure 101. ^{11}B NMR spectrum of **3l** (128.4 MHz, $CDCl_3$)

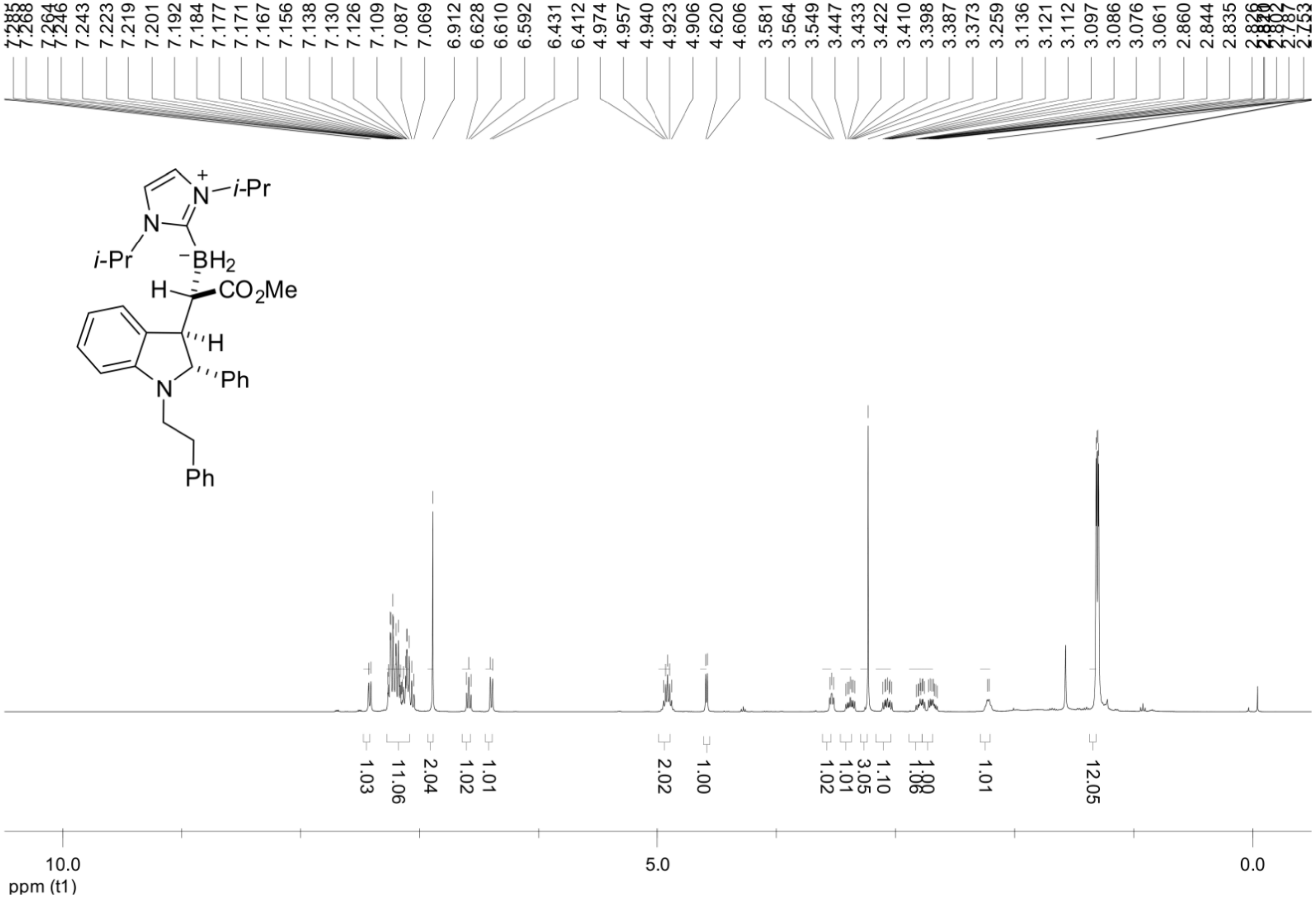

Supplementary Figure 102. ^{1}H NMR spectrum of **3m** (400 MHz, $CDCl_3$)

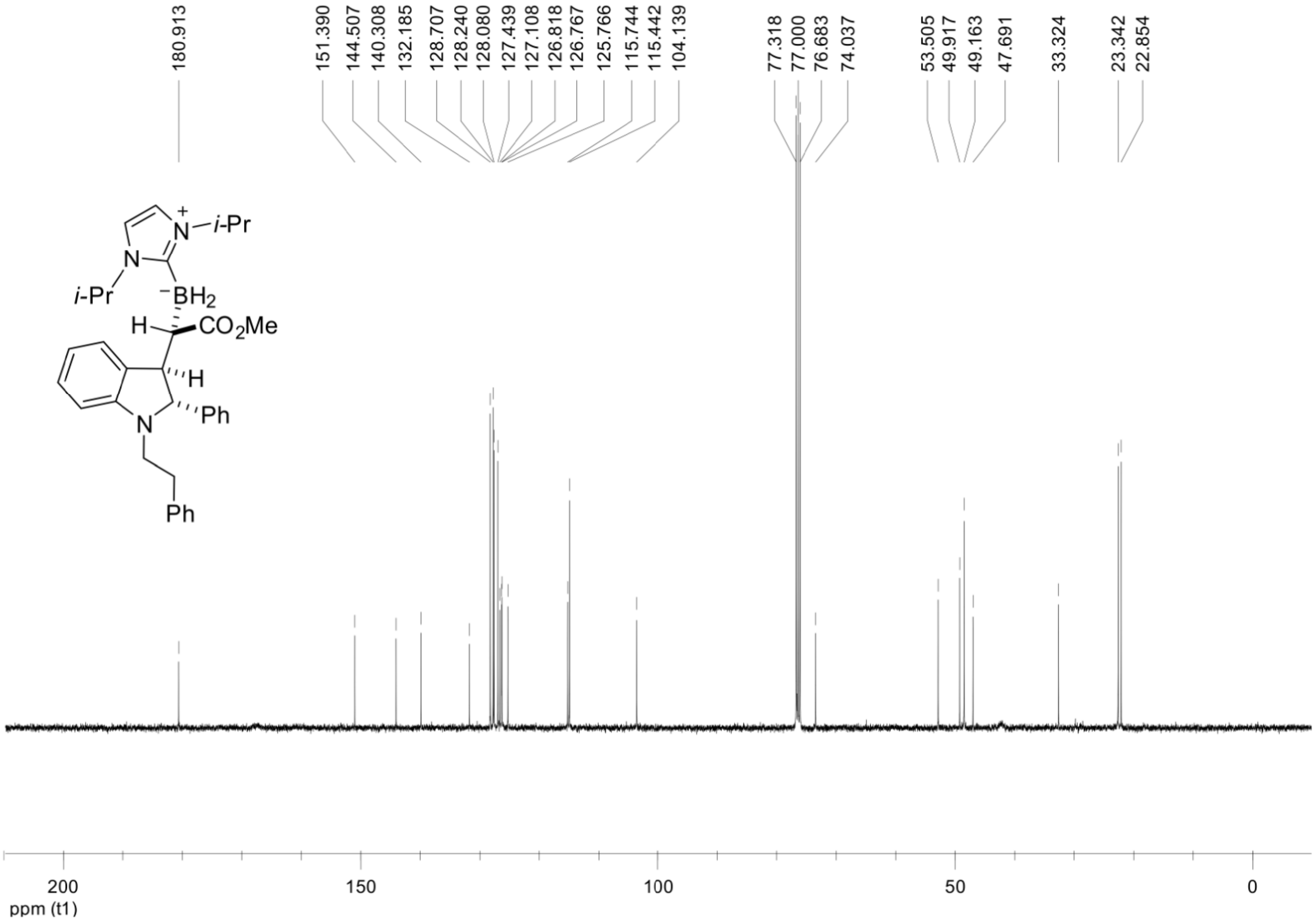

Supplementary Figure 103. ^{13}C NMR spectrum of **3m** (100 MHz, $CDCl_3$)

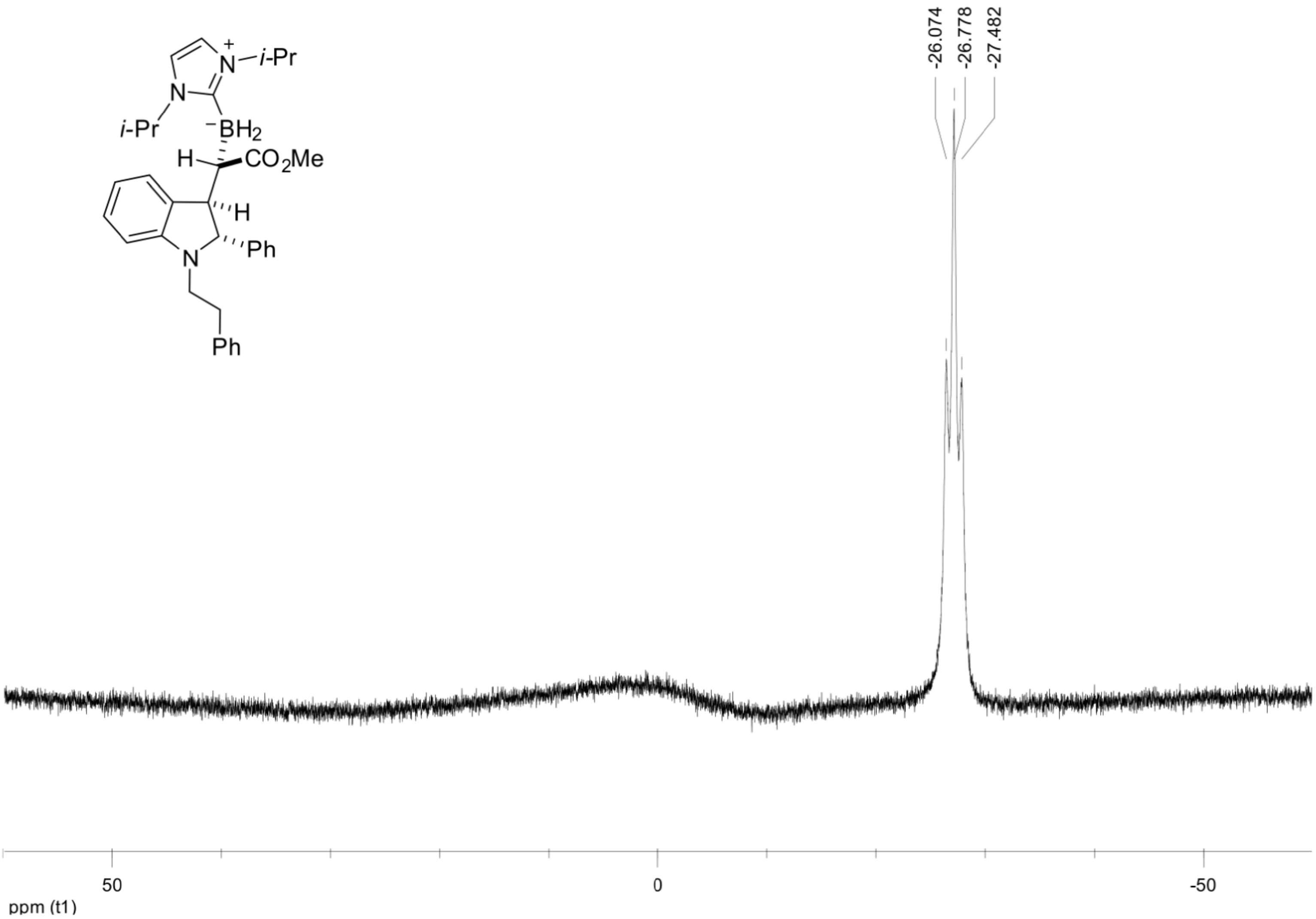

Supplementary Figure 104. ^{11}B NMR spectrum of **3m** (128.4 MHz, $CDCl_3$)

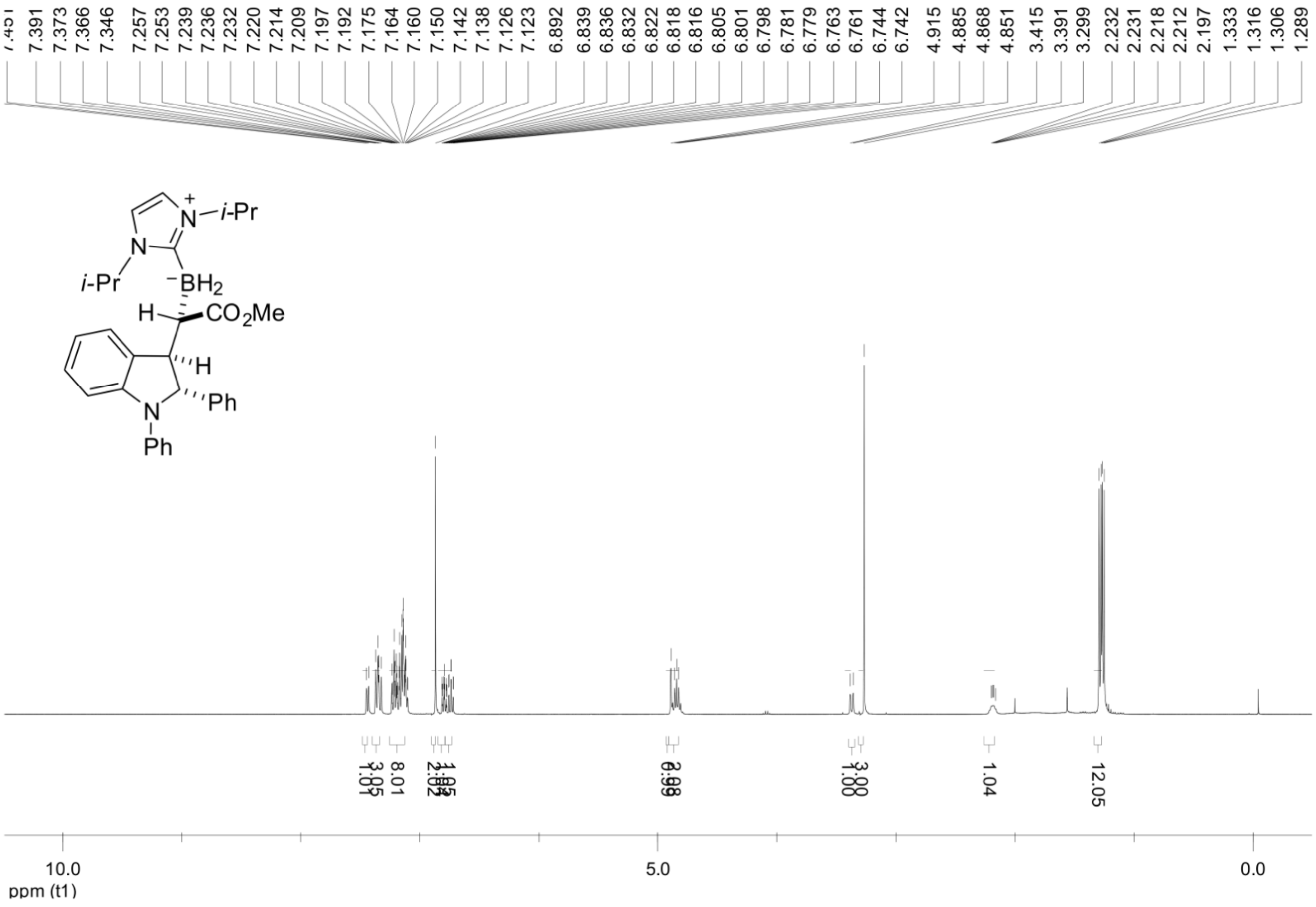

Supplementary Figure 105. ^{1}H NMR spectrum of **3n** (400 MHz, $CDCl_3$)

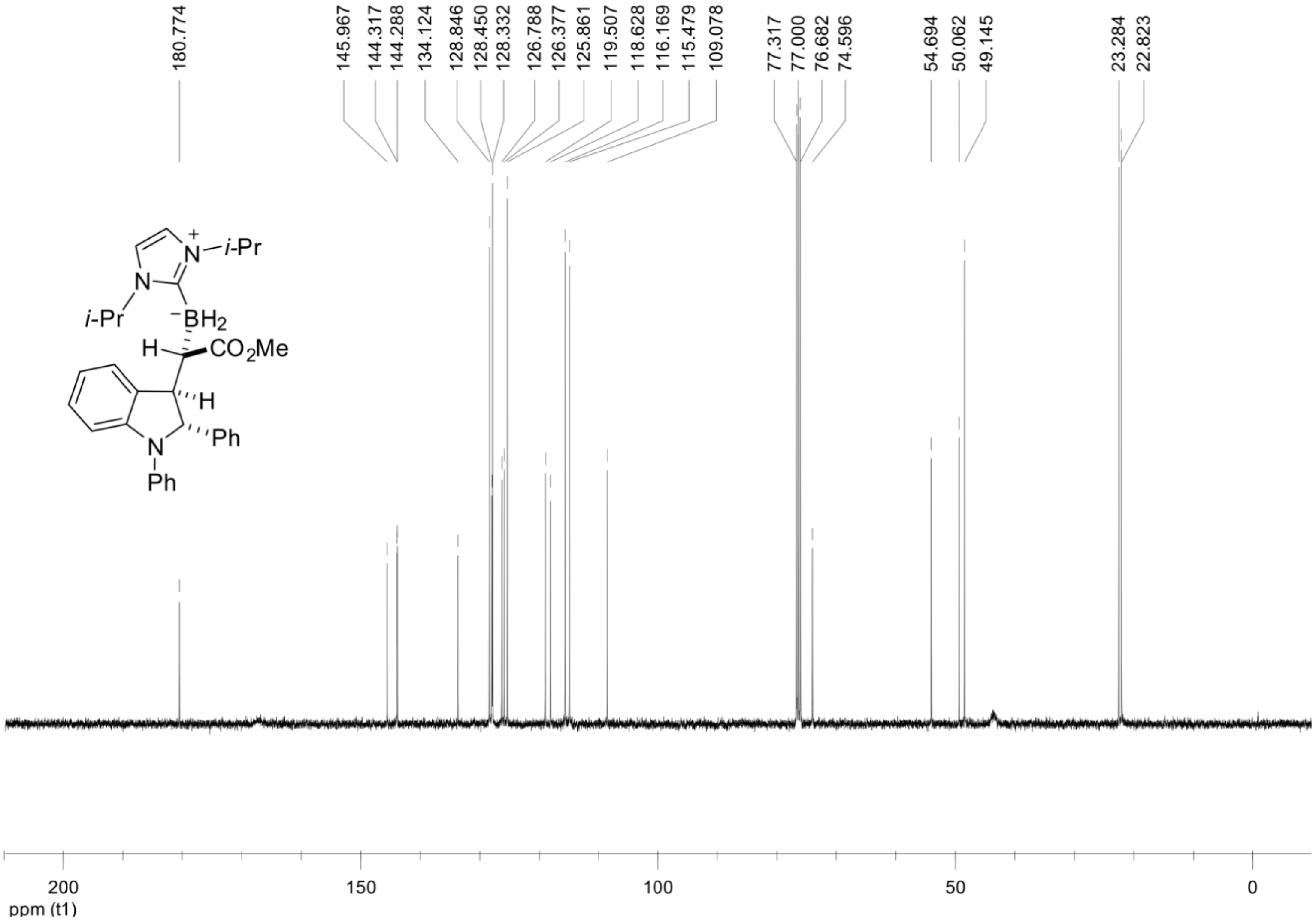

Supplementary Figure 106. ^{13}C NMR spectrum of **3n** (100 MHz, $CDCl_3$)

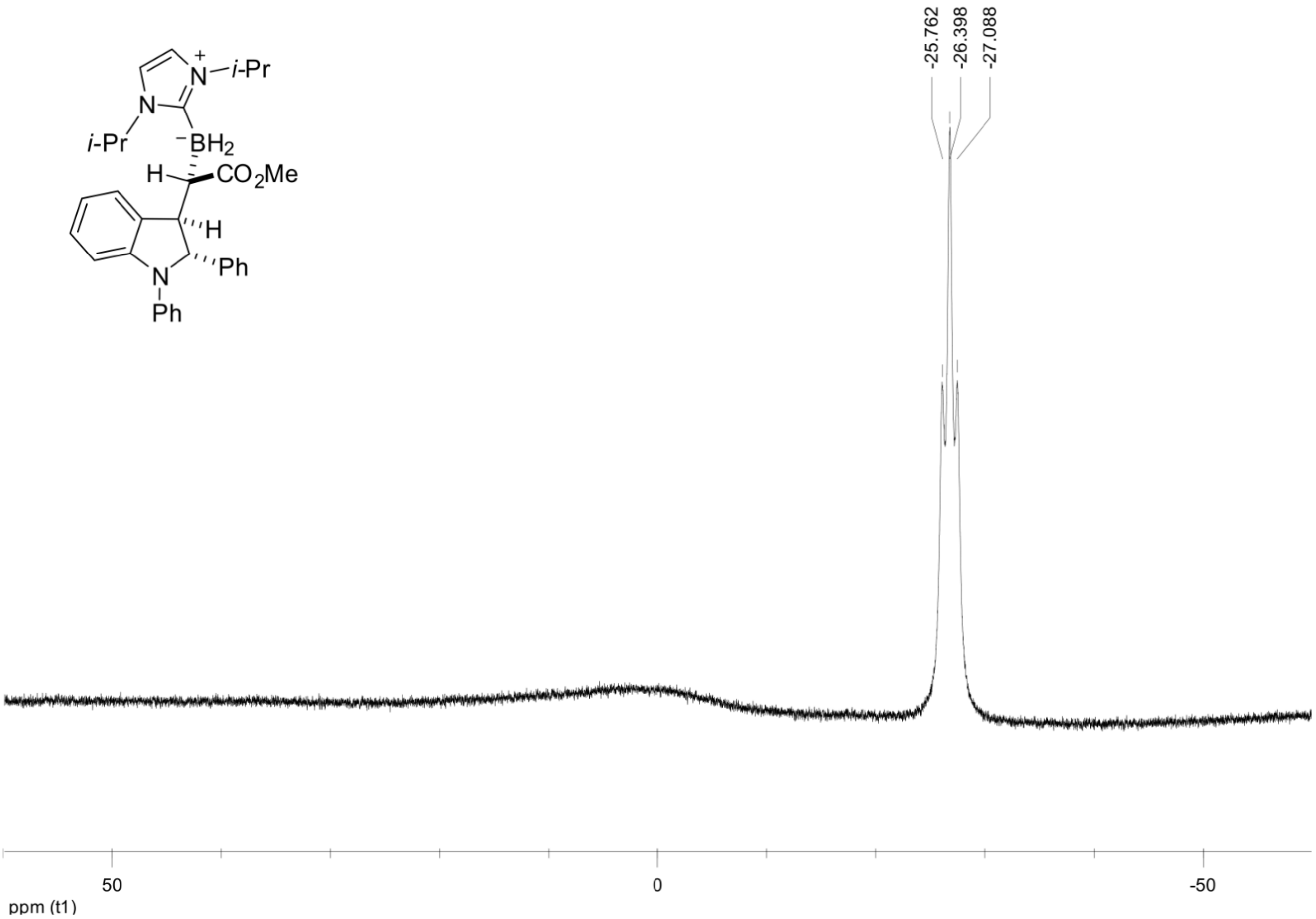

Supplementary Figure 107. ^{11}B NMR spectrum of **3n** (128.4 MHz, $CDCl_3$)

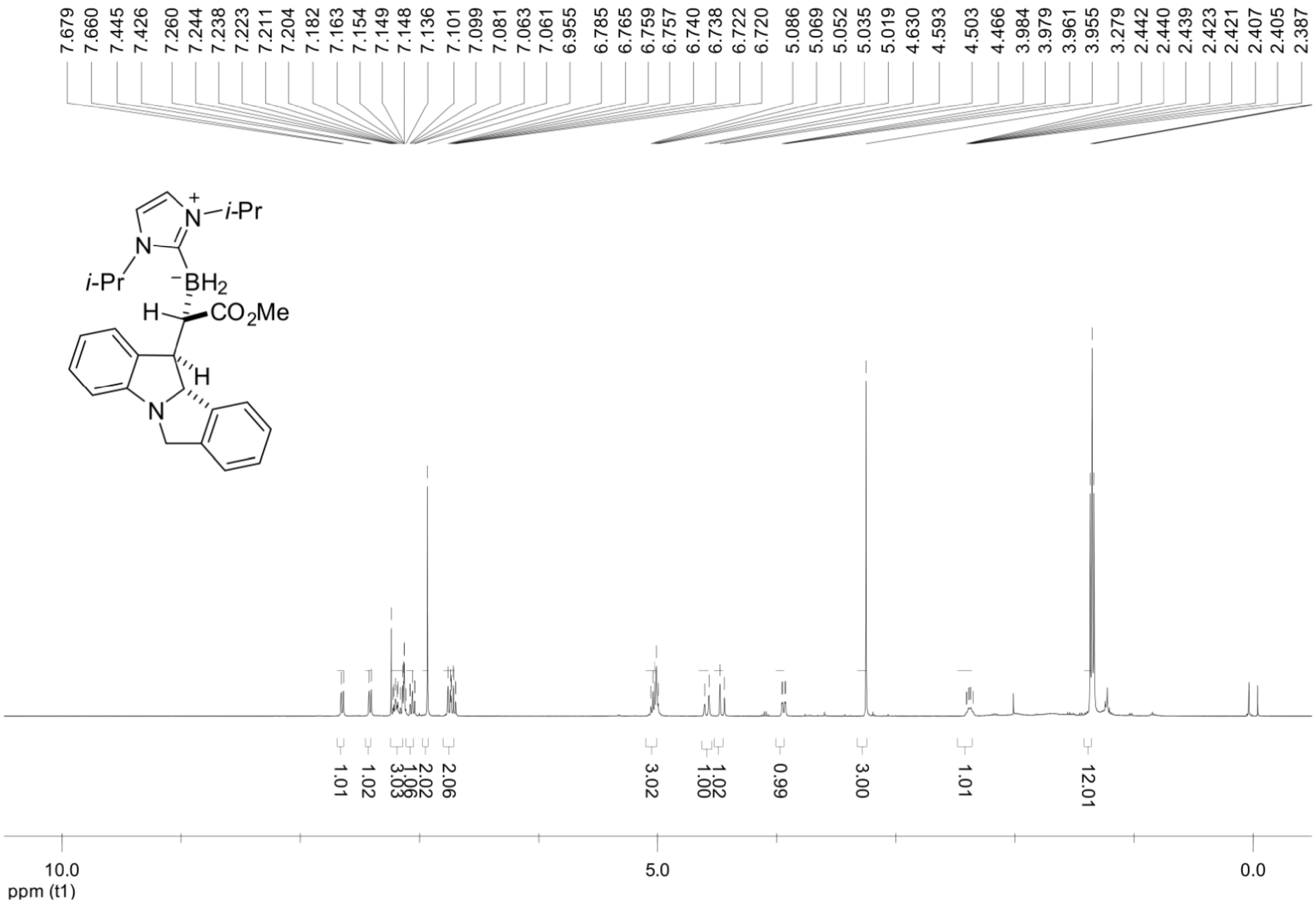

Supplementary Figure 108. ^{1}H NMR spectrum of **3p** (400 MHz, $CDCl_3$)

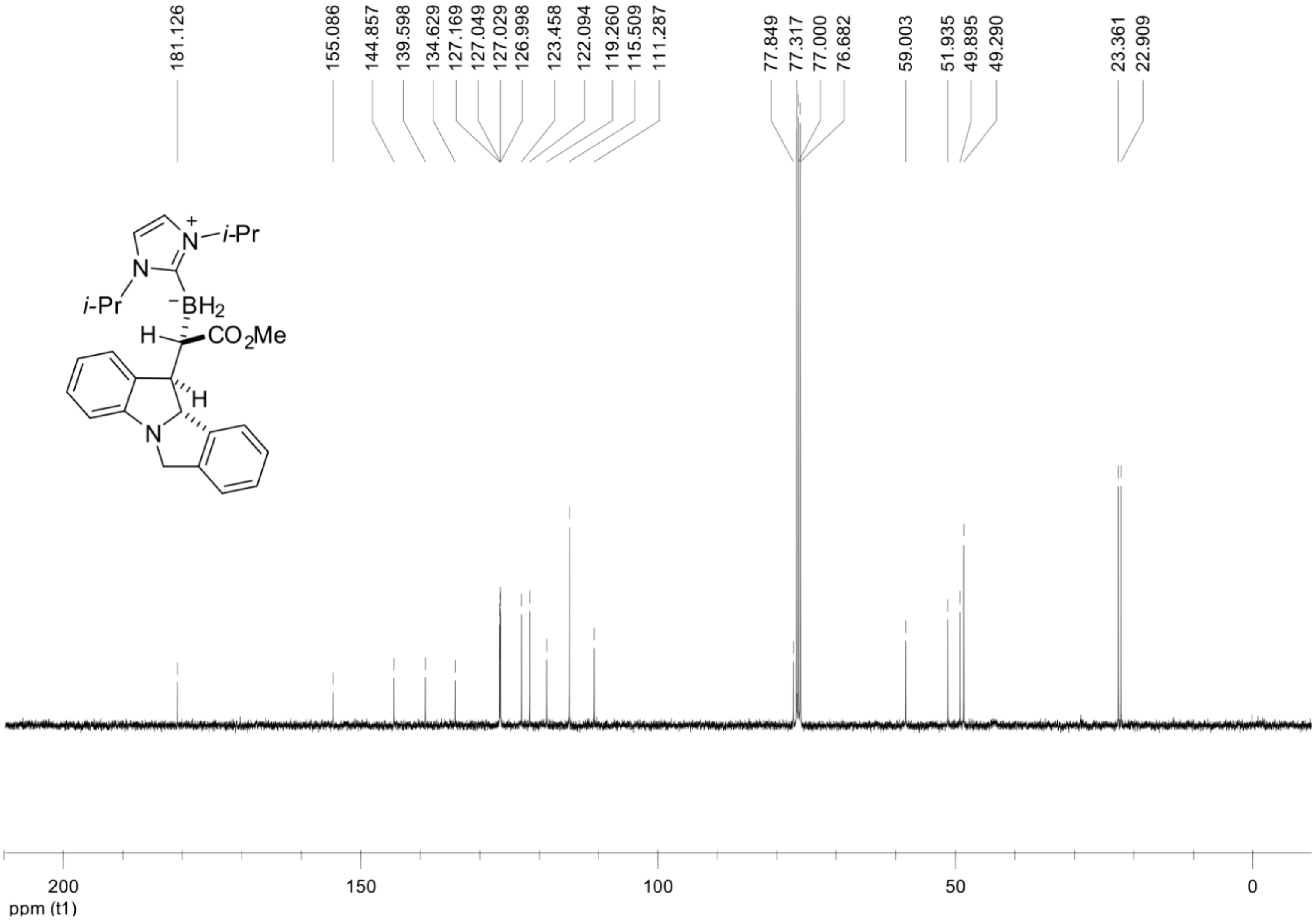

Supplementary Figure 109. ^{13}C NMR spectrum of **3p** (100 MHz, $CDCl_3$)

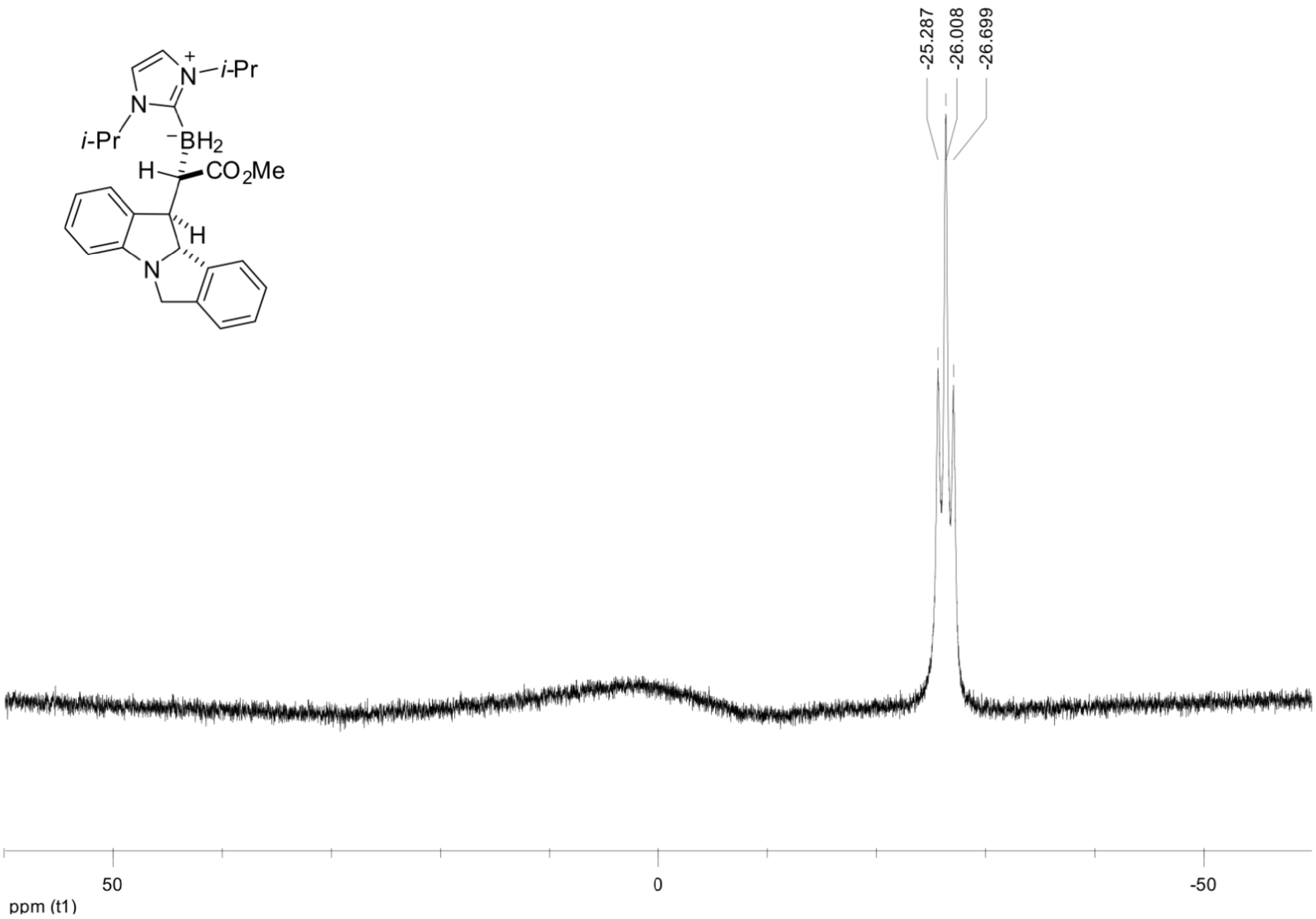

Supplementary Figure 110. ^{11}B NMR spectrum of **3p** (128.4 MHz, $CDCl_3$)

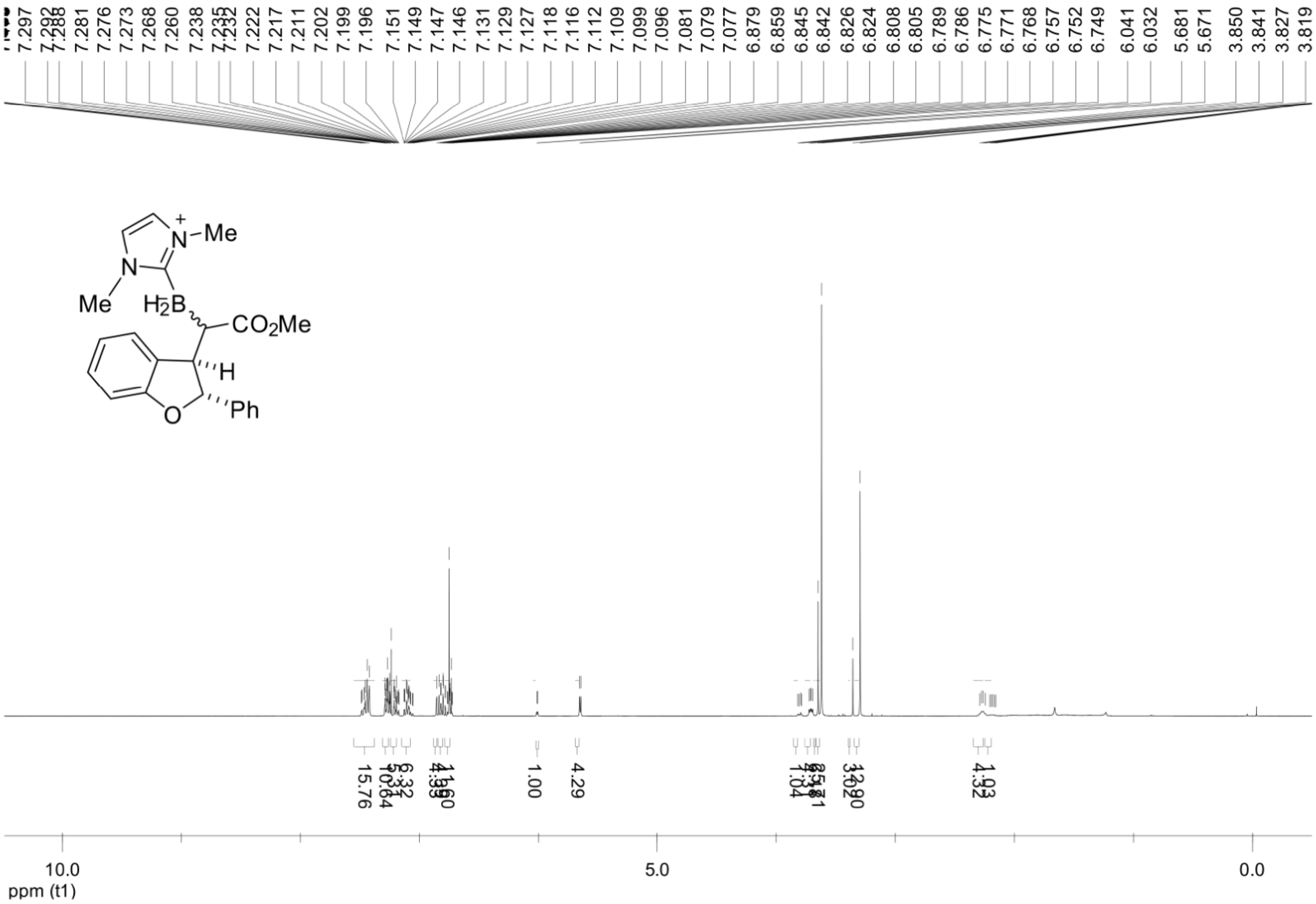

Supplementary Figure 111. ^{1}H NMR spectrum of **3q** (400 MHz, $CDCl_3$)

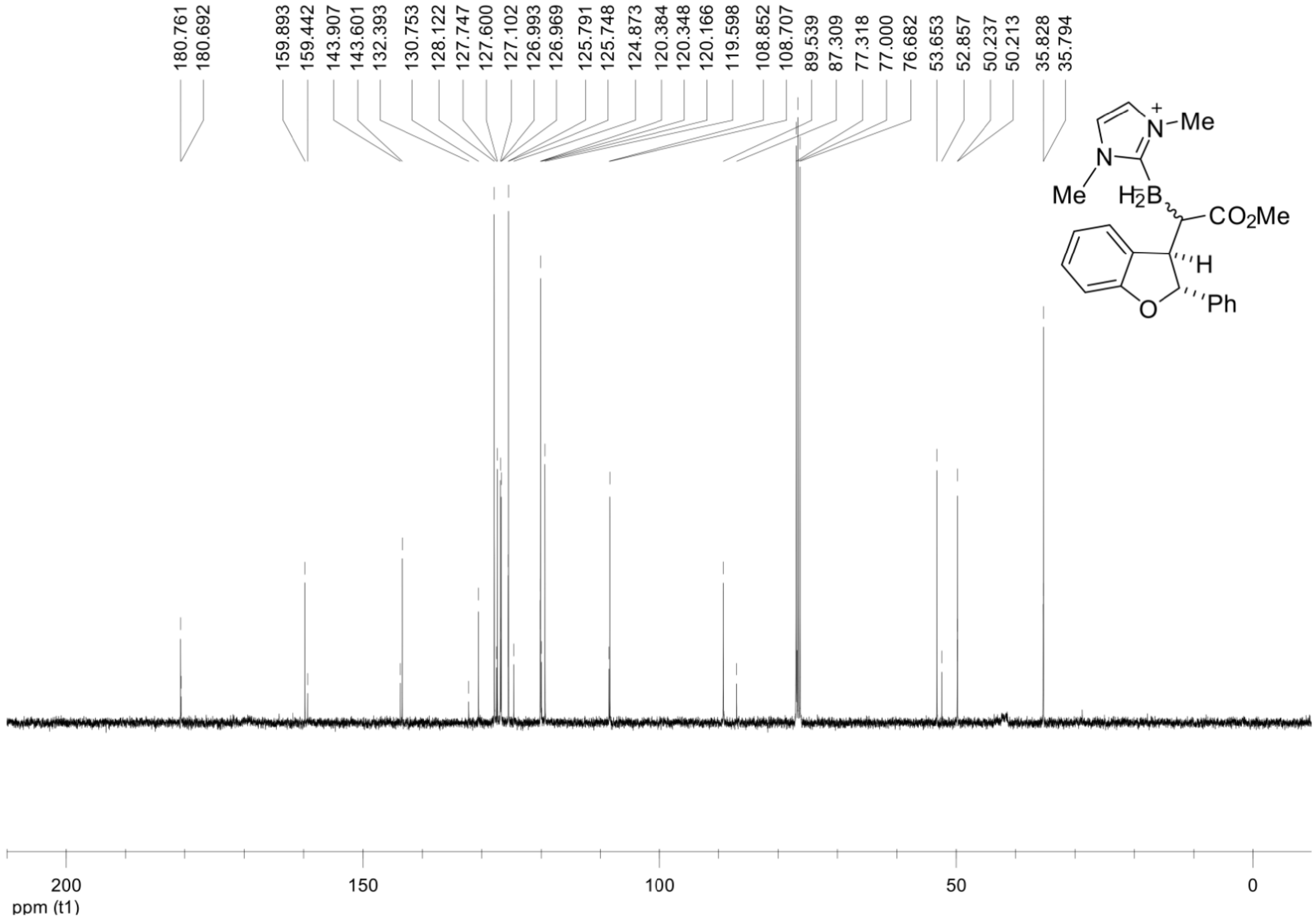

Supplementary Figure 112. ^{13}C NMR spectrum of **3q** (100 MHz, $CDCl_3$)

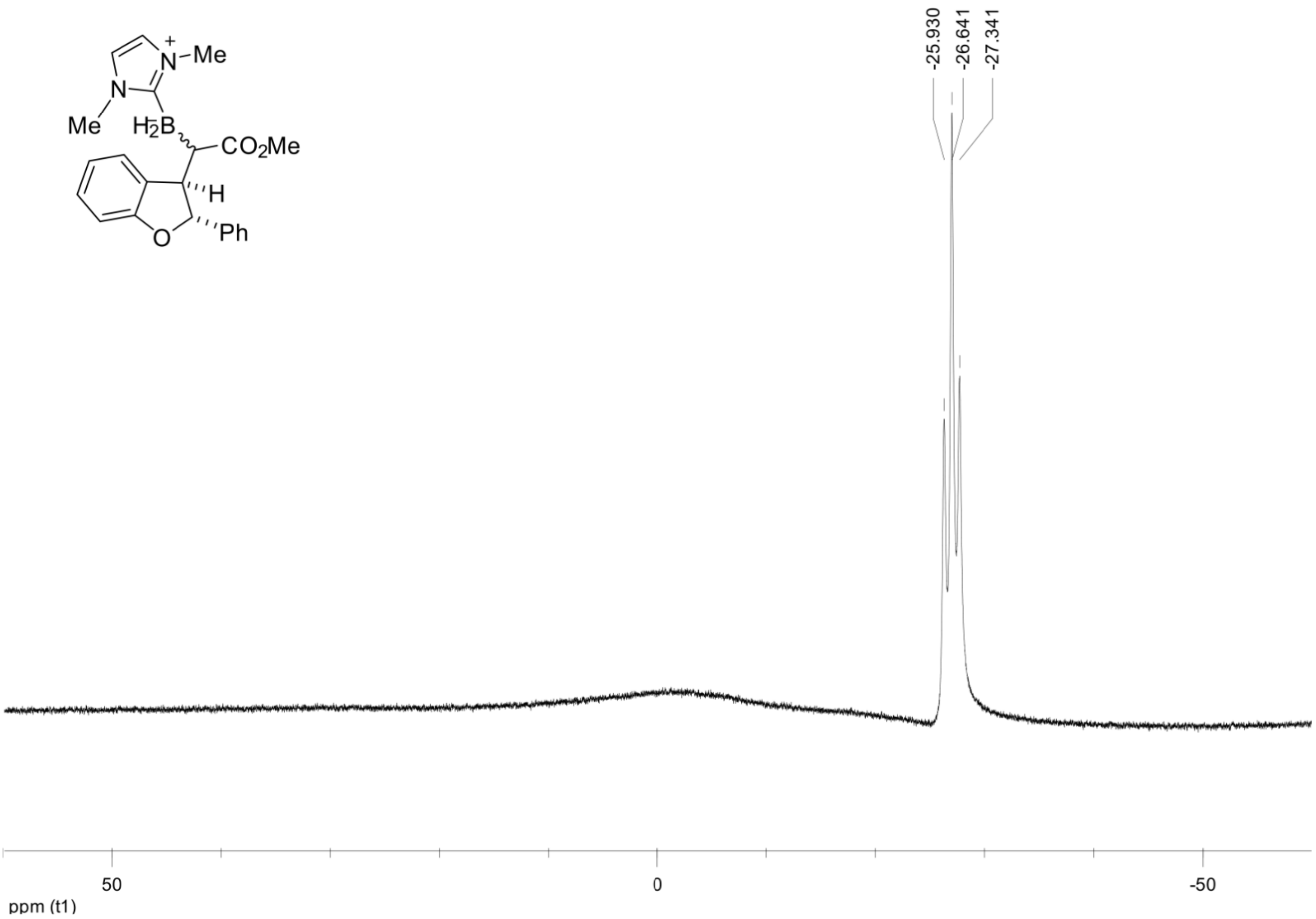

Supplementary Figure 113. ^{11}B NMR spectrum of **3q** (128.4 MHz, $CDCl_3$)

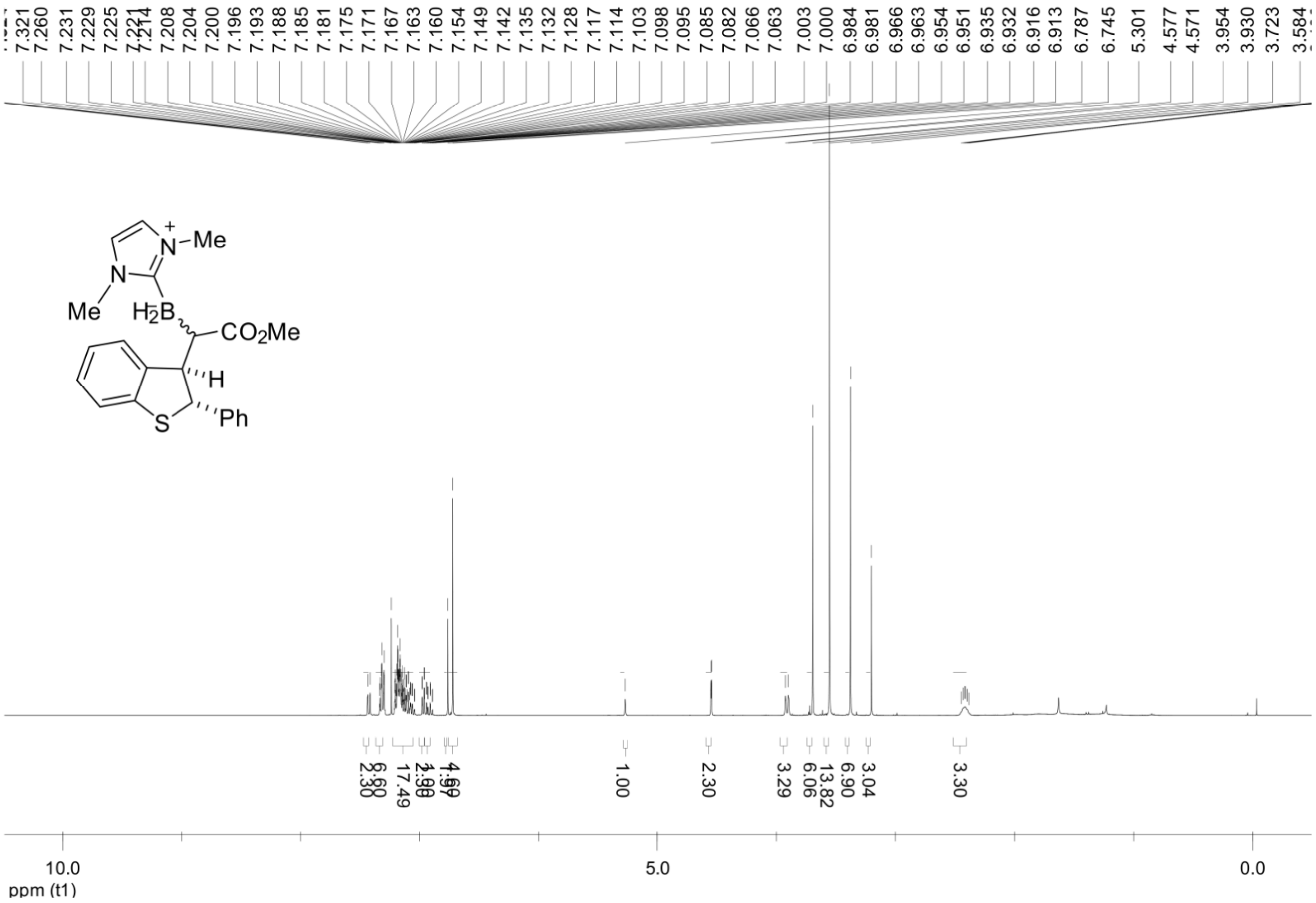

Supplementary Figure 114. ^{1}H NMR spectrum of **3r** (400 MHz, $CDCl_3$)

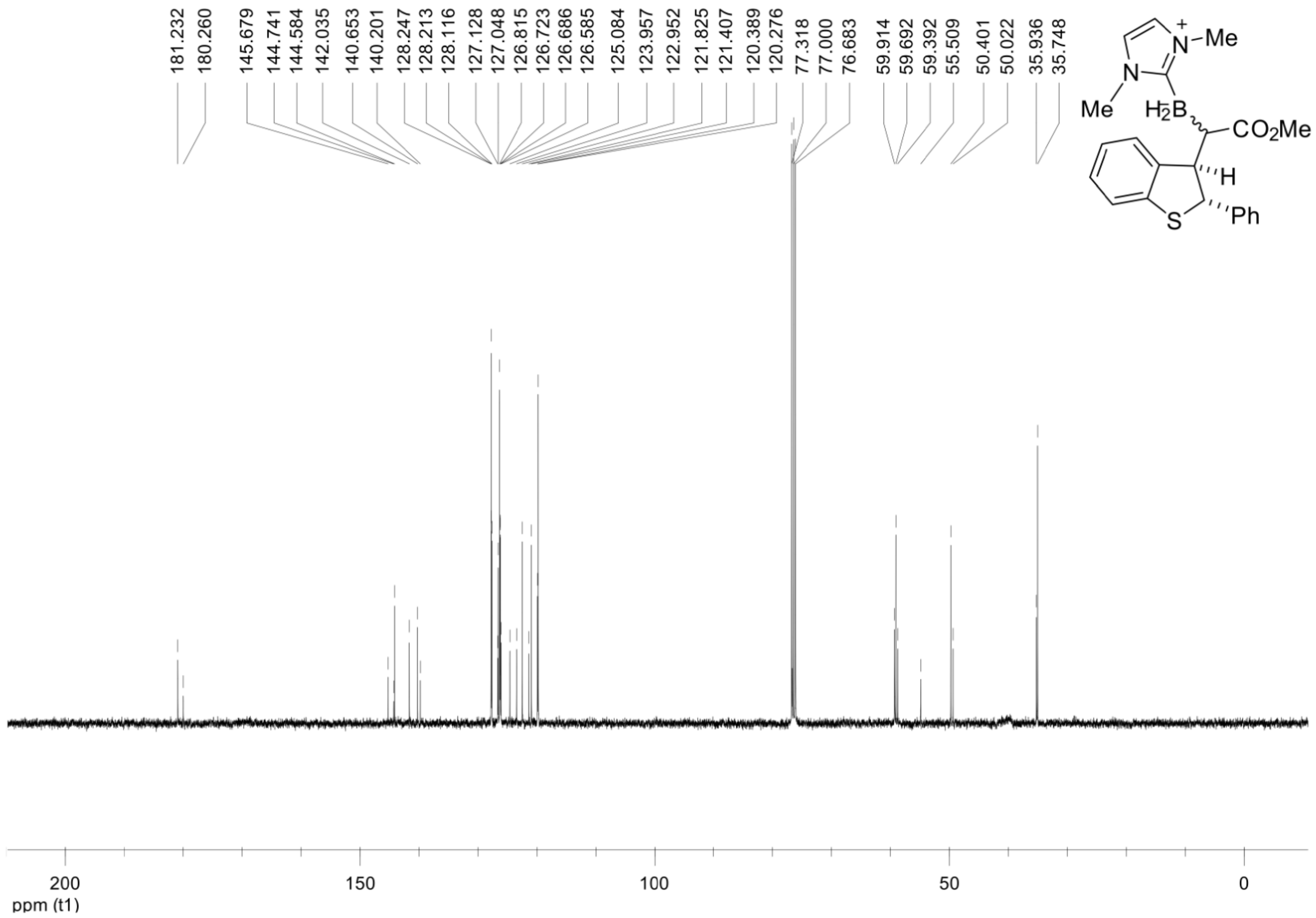

Supplementary Figure 115. ^{13}C NMR spectrum of **3r** (100 MHz, $CDCl_3$)

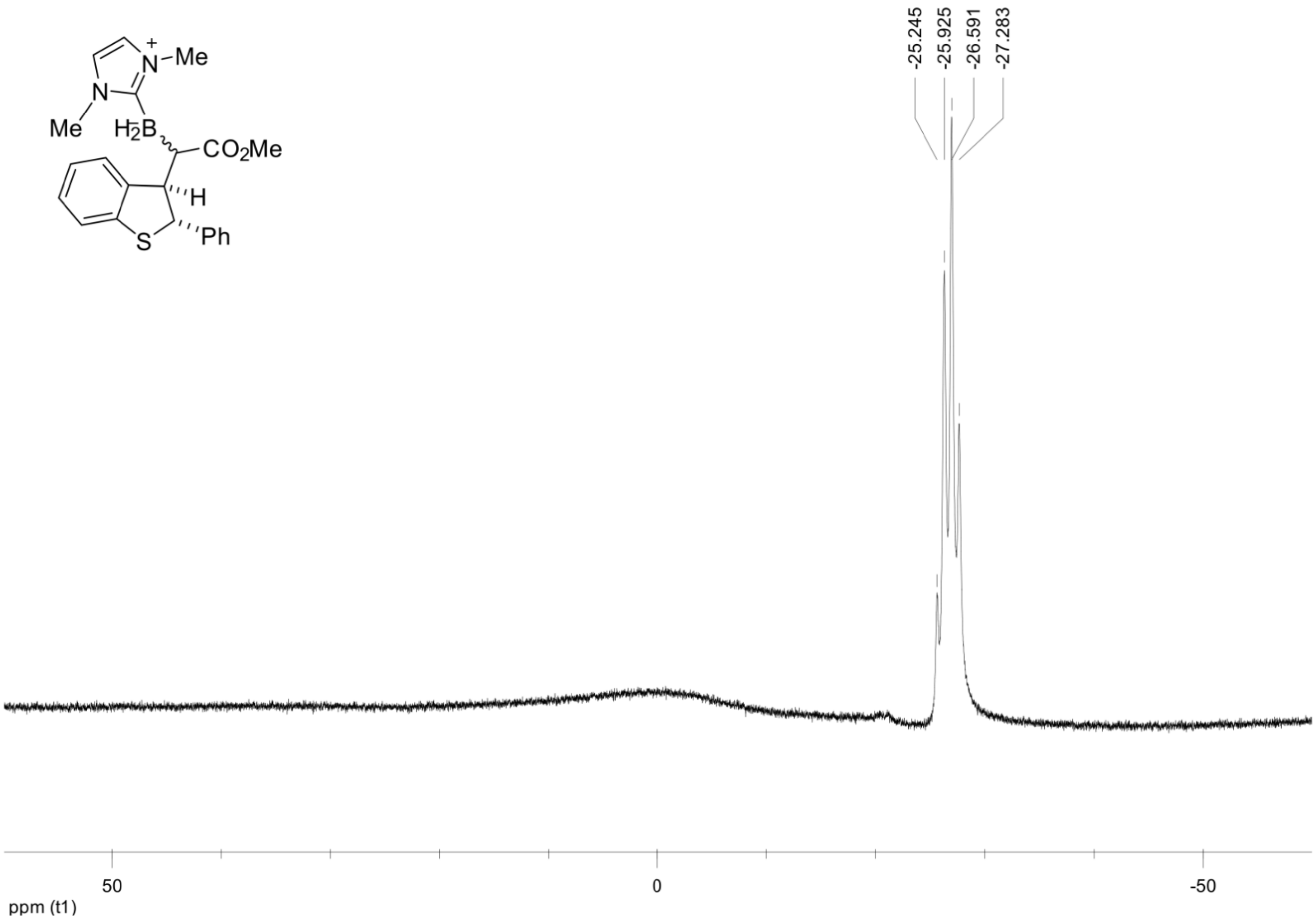

Supplementary Figure 116. ^{11}B NMR spectrum of **3r** (128.4 MHz, $CDCl_3$)

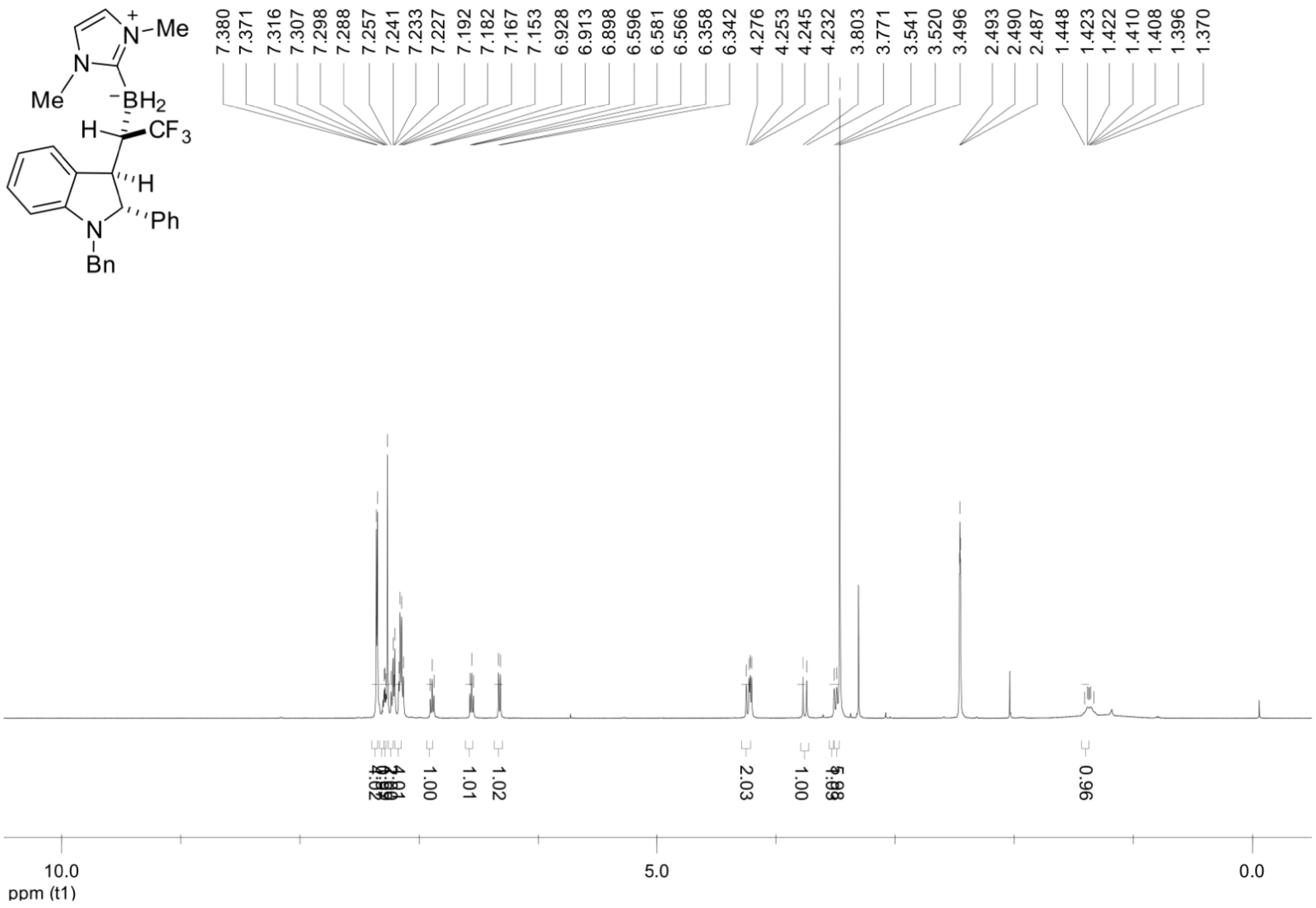

Supplementary Figure 117. ^{1}H NMR spectrum of **3s** (500 MHz, DMSO-d_6)

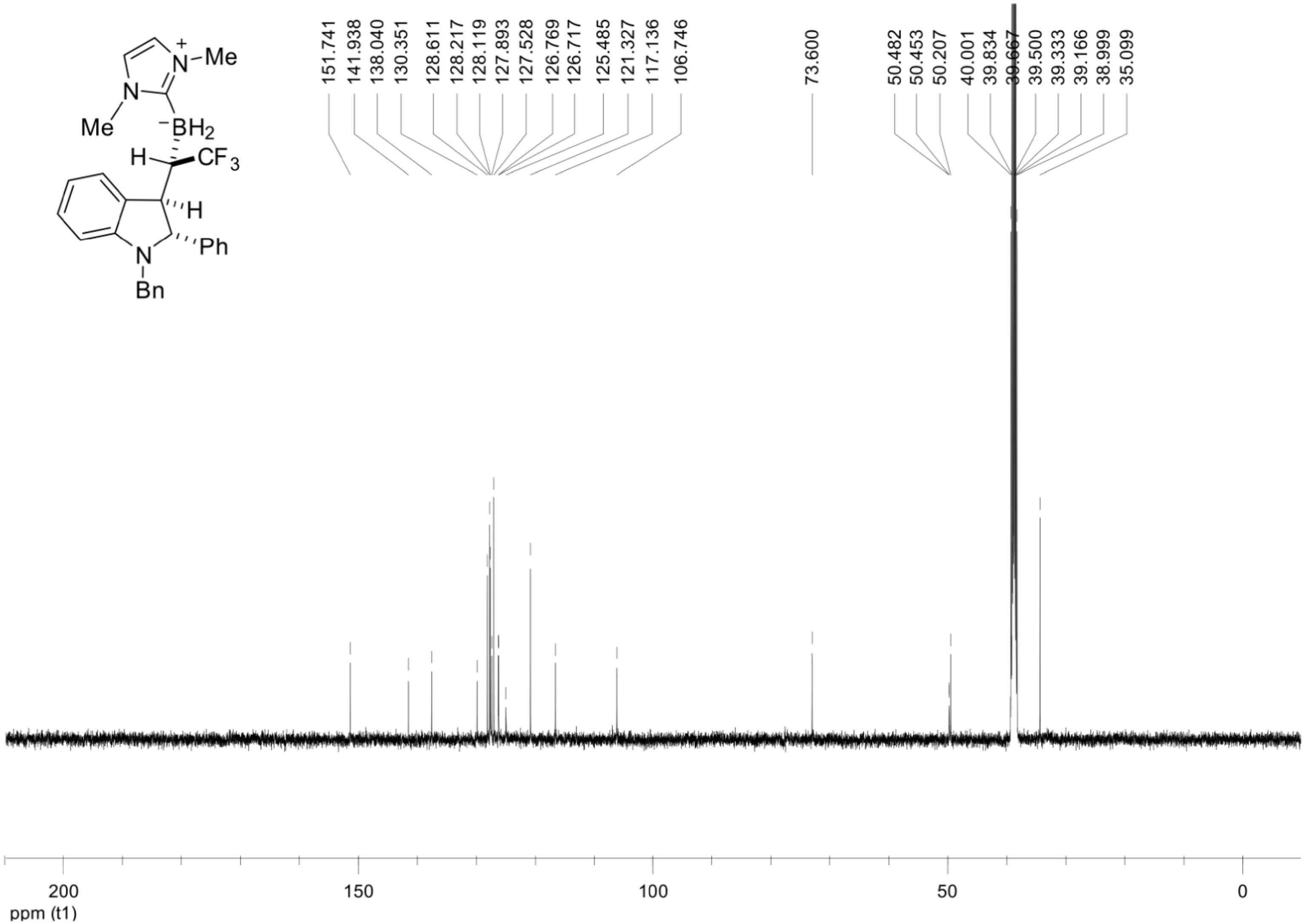

Supplementary Figure 118. ^{13}C NMR spectrum of **3s** (125 MHz, DMSO-d_6)

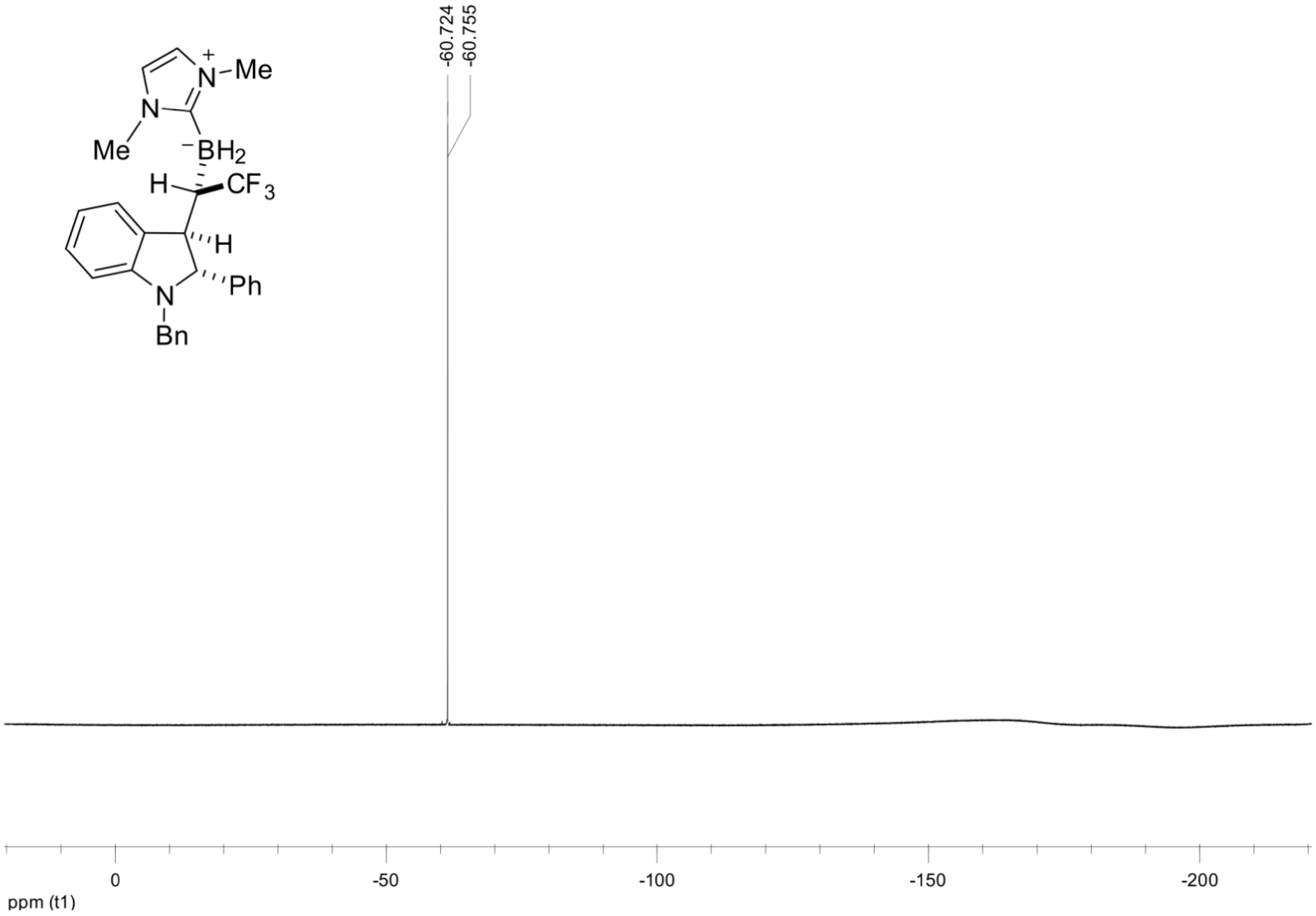

Supplementary Figure 119. ^{19}F NMR spectrum of **3s** (376 MHz, $CDCl_3$)

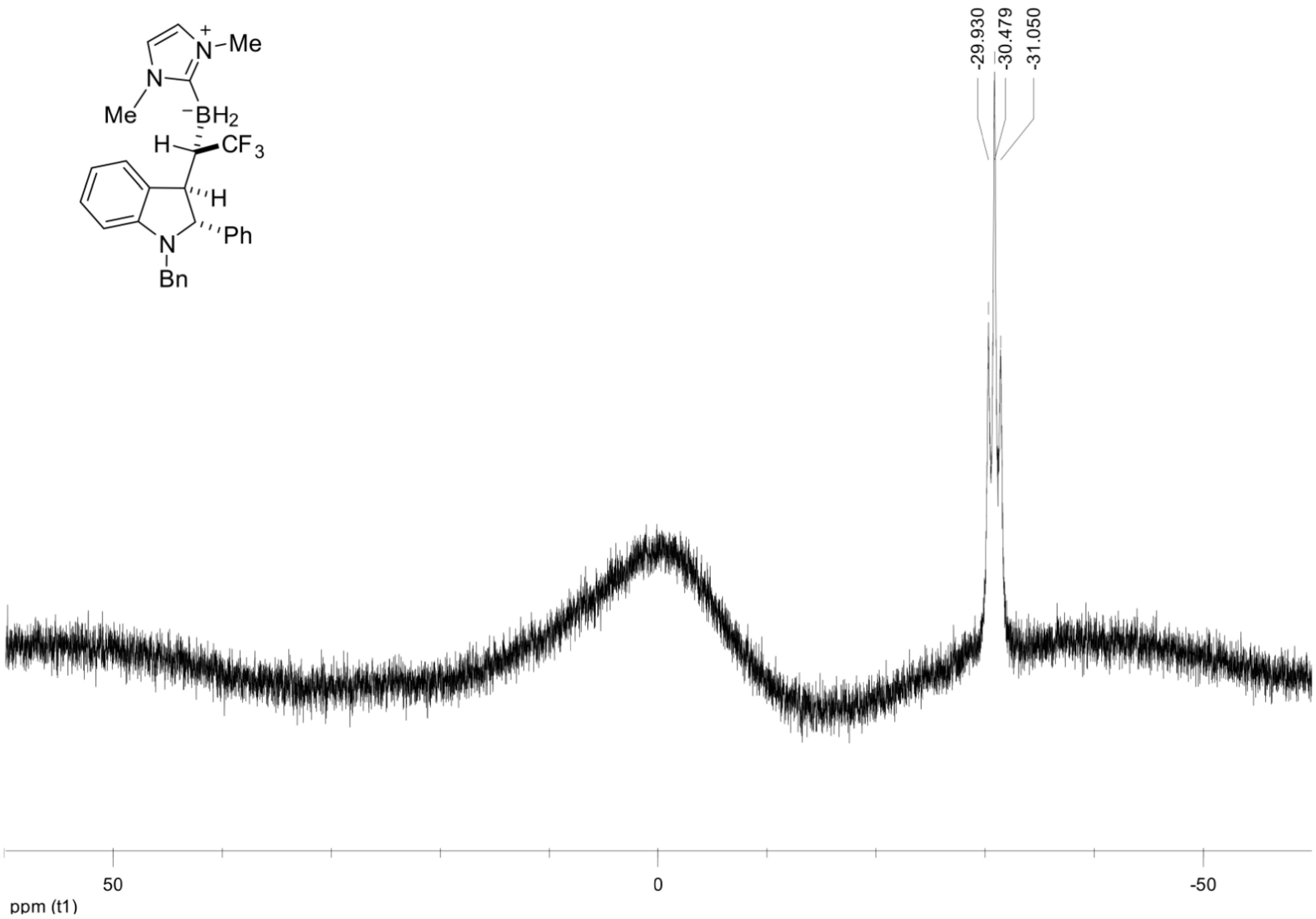

Supplementary Figure 120. ^{11}B NMR spectrum of **3s** (128.4 MHz, $CDCl_3$)

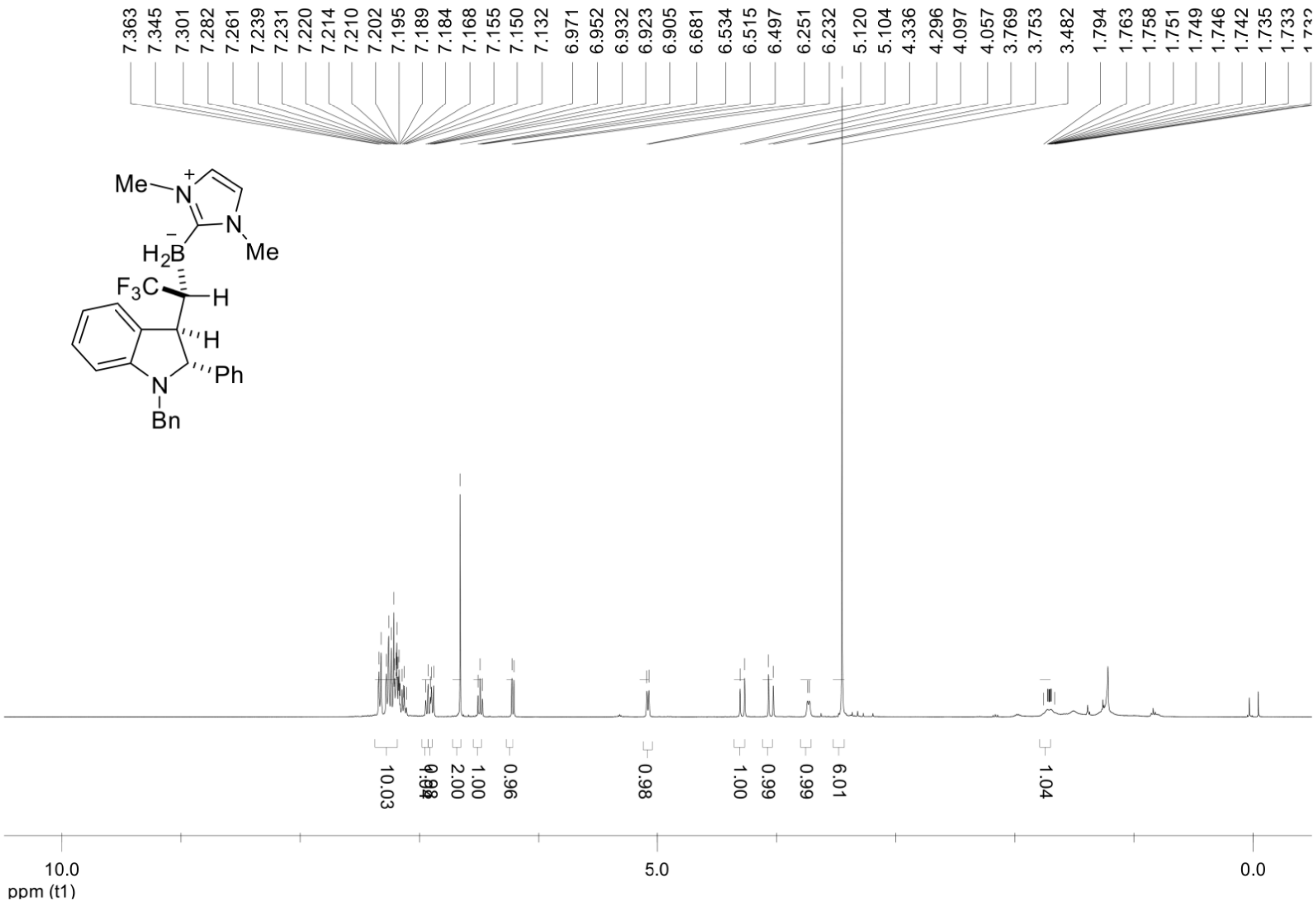

Supplementary Figure 121. ^{1}H NMR spectrum of **4s** (400 MHz, $CDCl_3$)

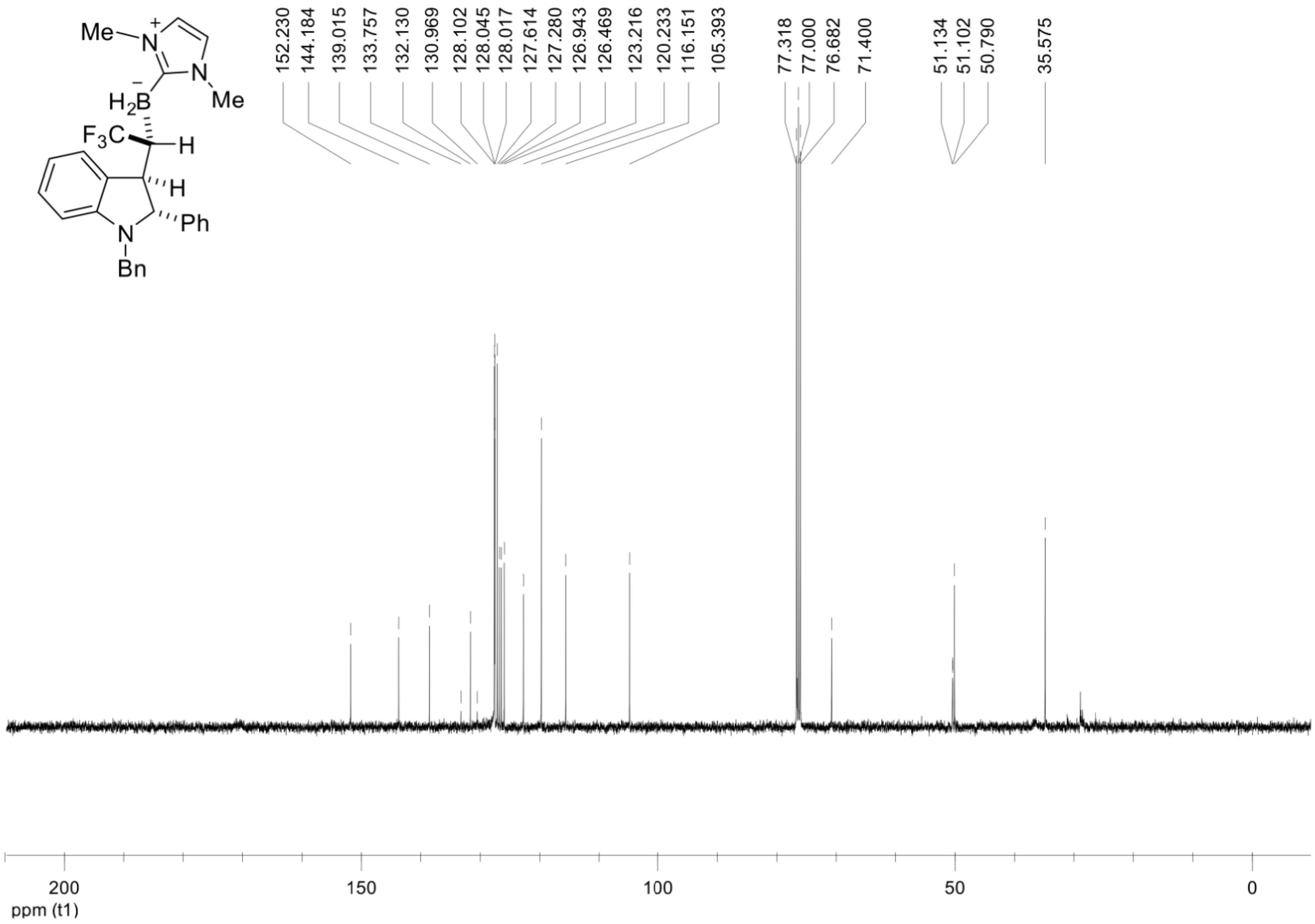

Supplementary Figure 122. ^{13}C NMR spectrum of **4s** (100 MHz, $CDCl_3$)

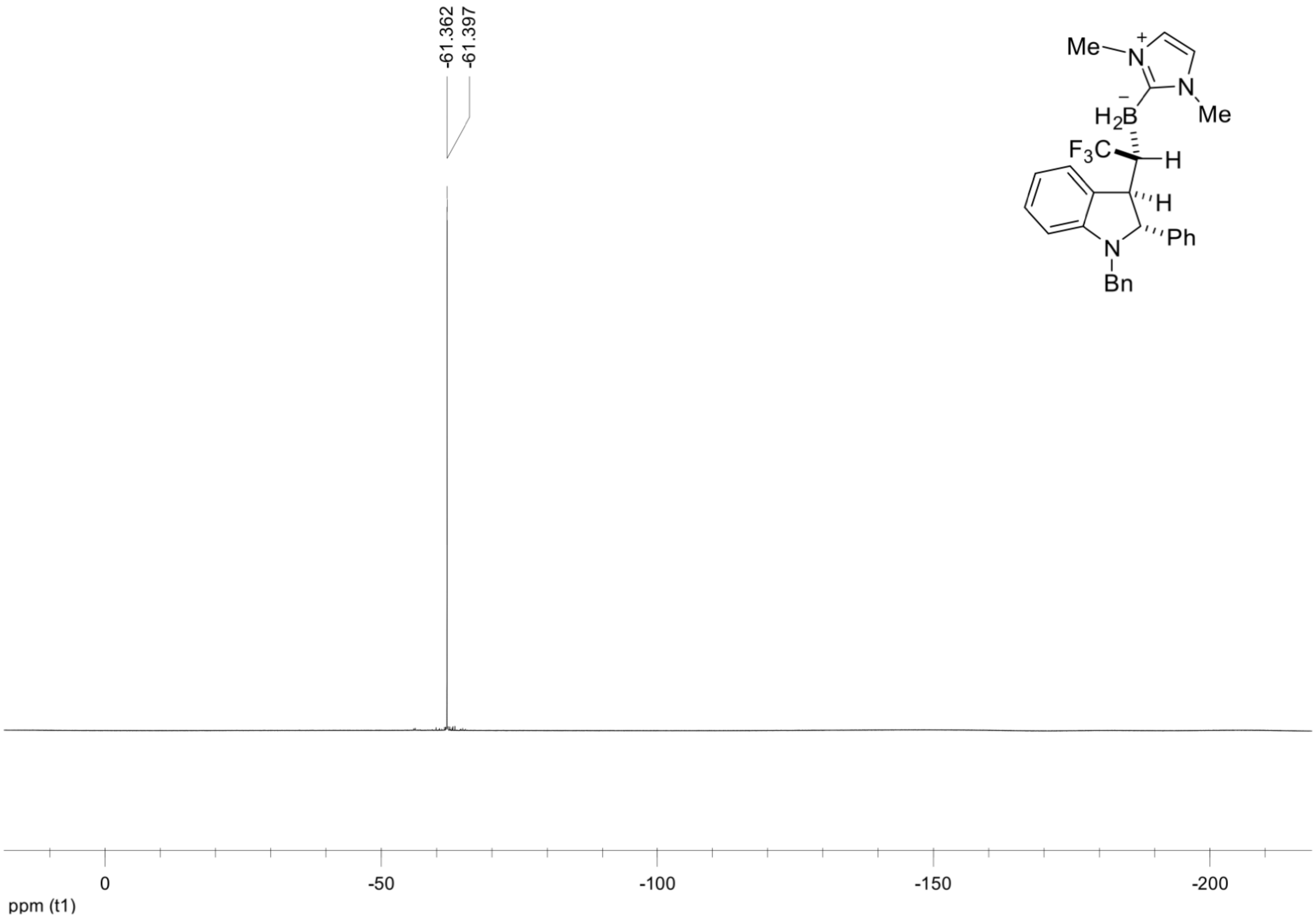

Supplementary Figure 123. ^{19}F NMR spectrum of **4s** (376 MHz, $CDCl_3$)

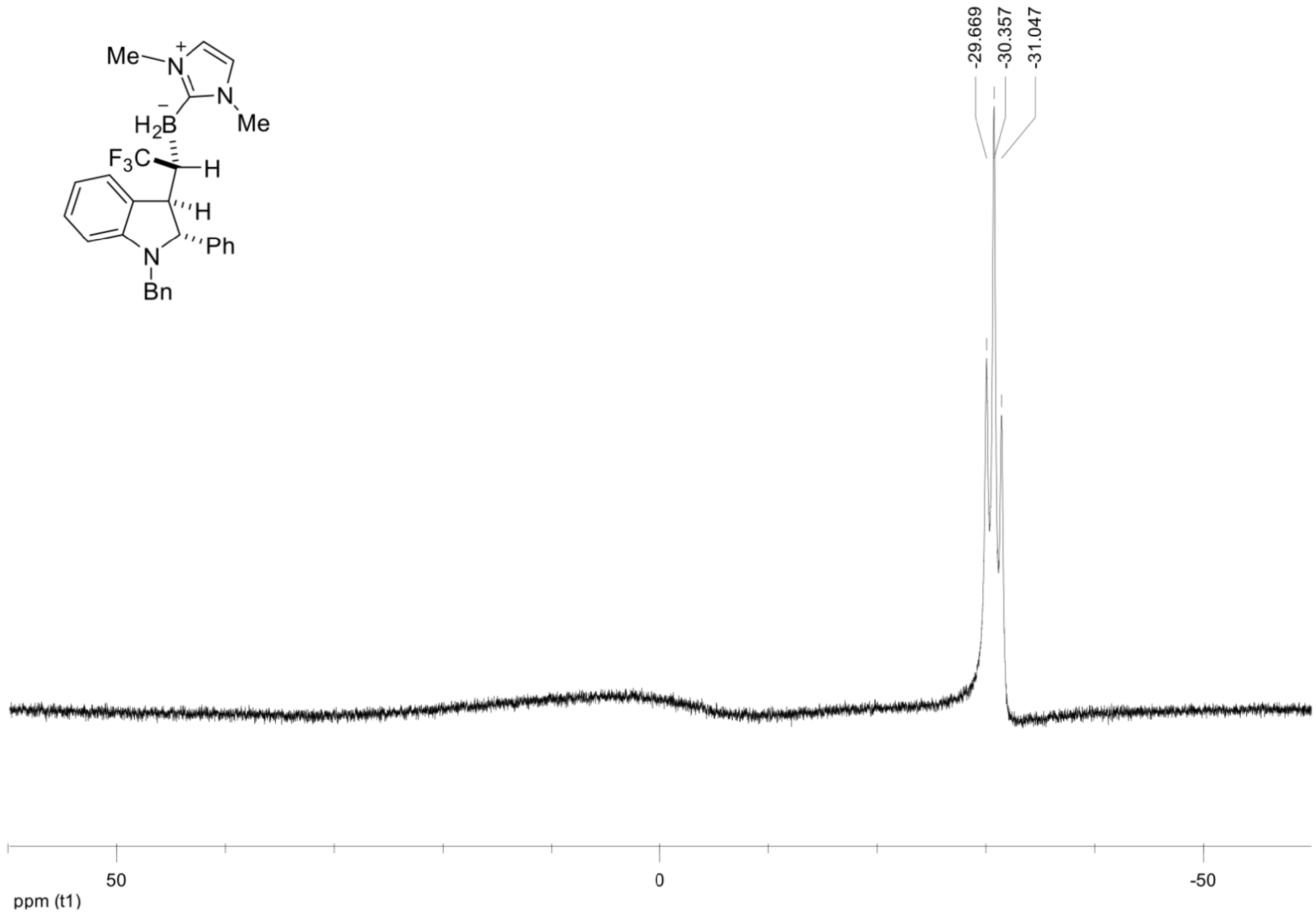

Supplementary Figure 124. ^{11}B NMR spectrum of **4s** (128.4 MHz, $CDCl_3$)

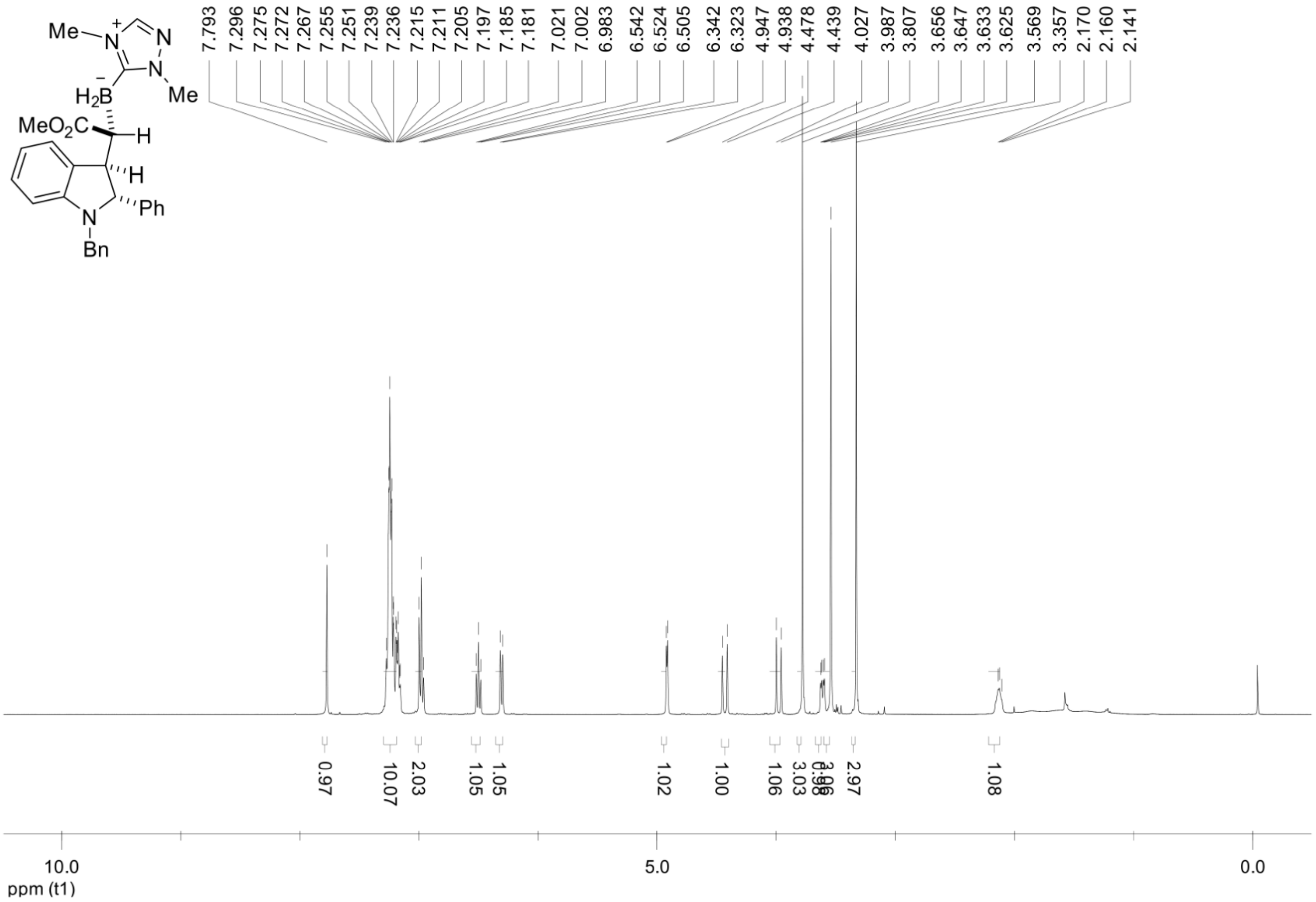

Supplementary Figure 125. ^{1}H NMR spectrum of **4aa** (400 MHz, $CDCl_3$)

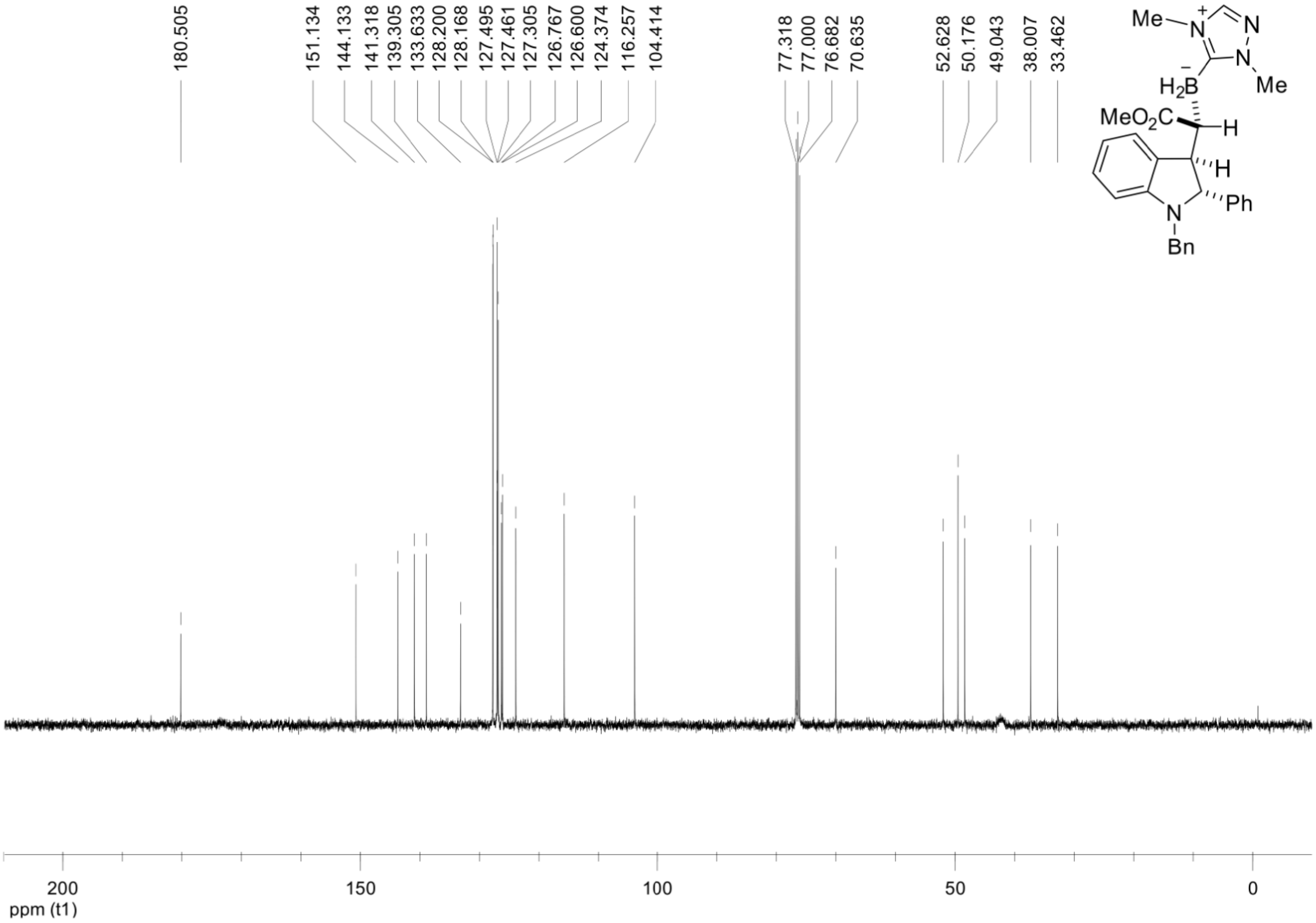

Supplementary Figure 126. ${}^{13}C$ NMR spectrum of **4aa** (100 MHz, $CDCl_3$)

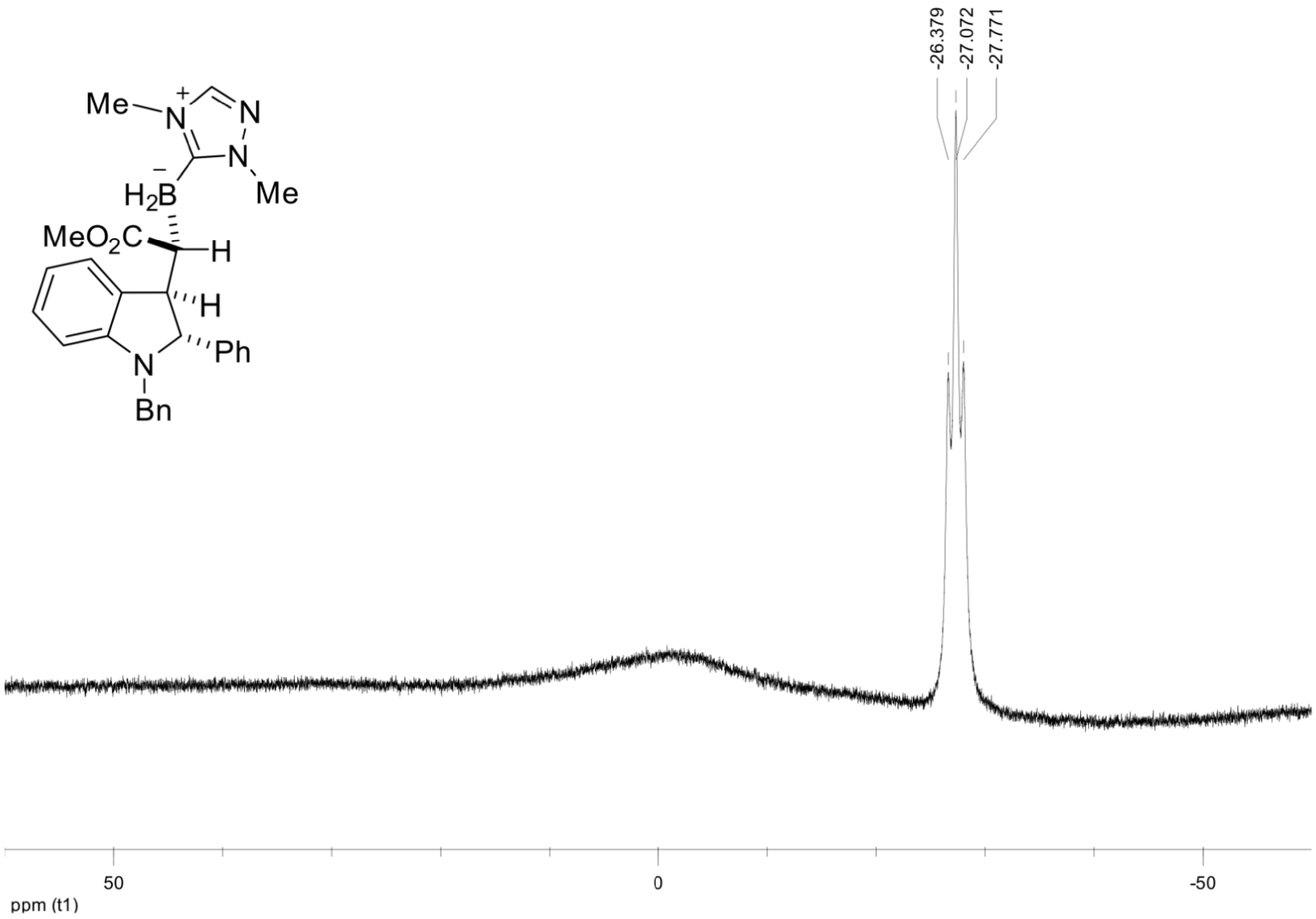

Supplementary Figure 127. ^{11}B NMR spectrum of **4aa** (128.4 MHz, $CDCl_3$)

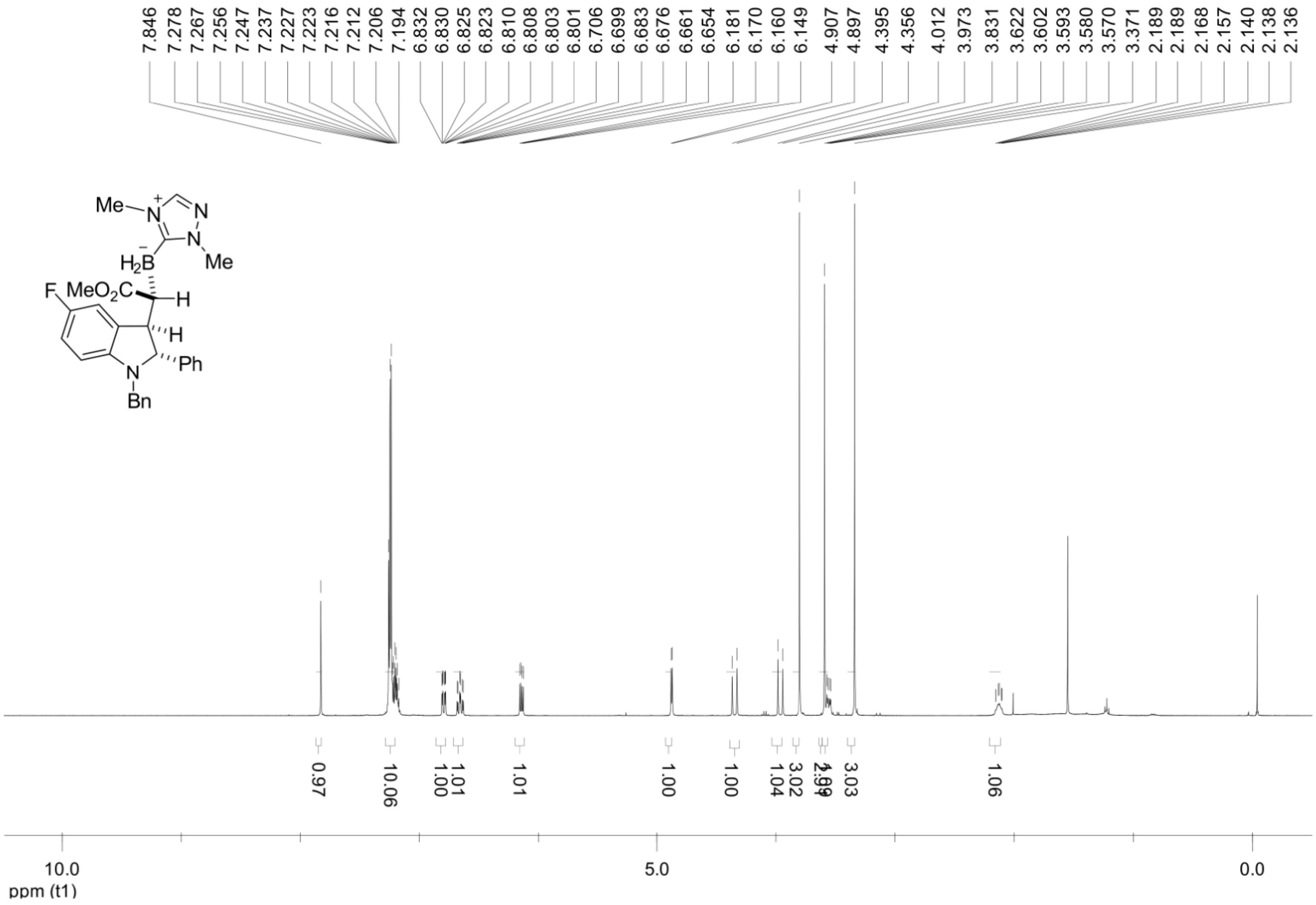

Supplementary Figure 128. ^{1}H NMR spectrum of **4ha** (400 MHz, $CDCl_3$)

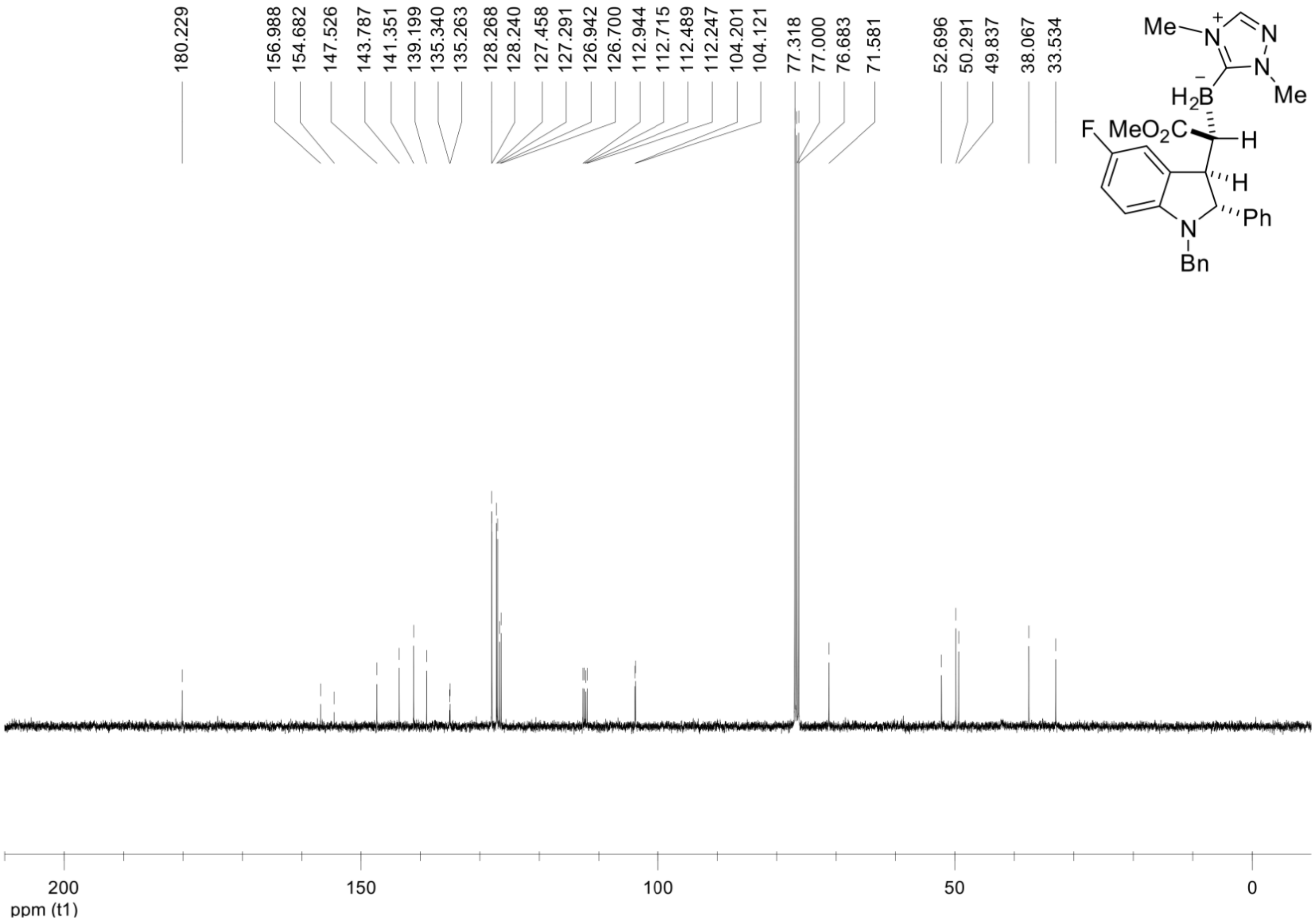

Supplementary Figure 129. ^{13}C NMR spectrum of **4ha** (100 MHz, $CDCl_3$)

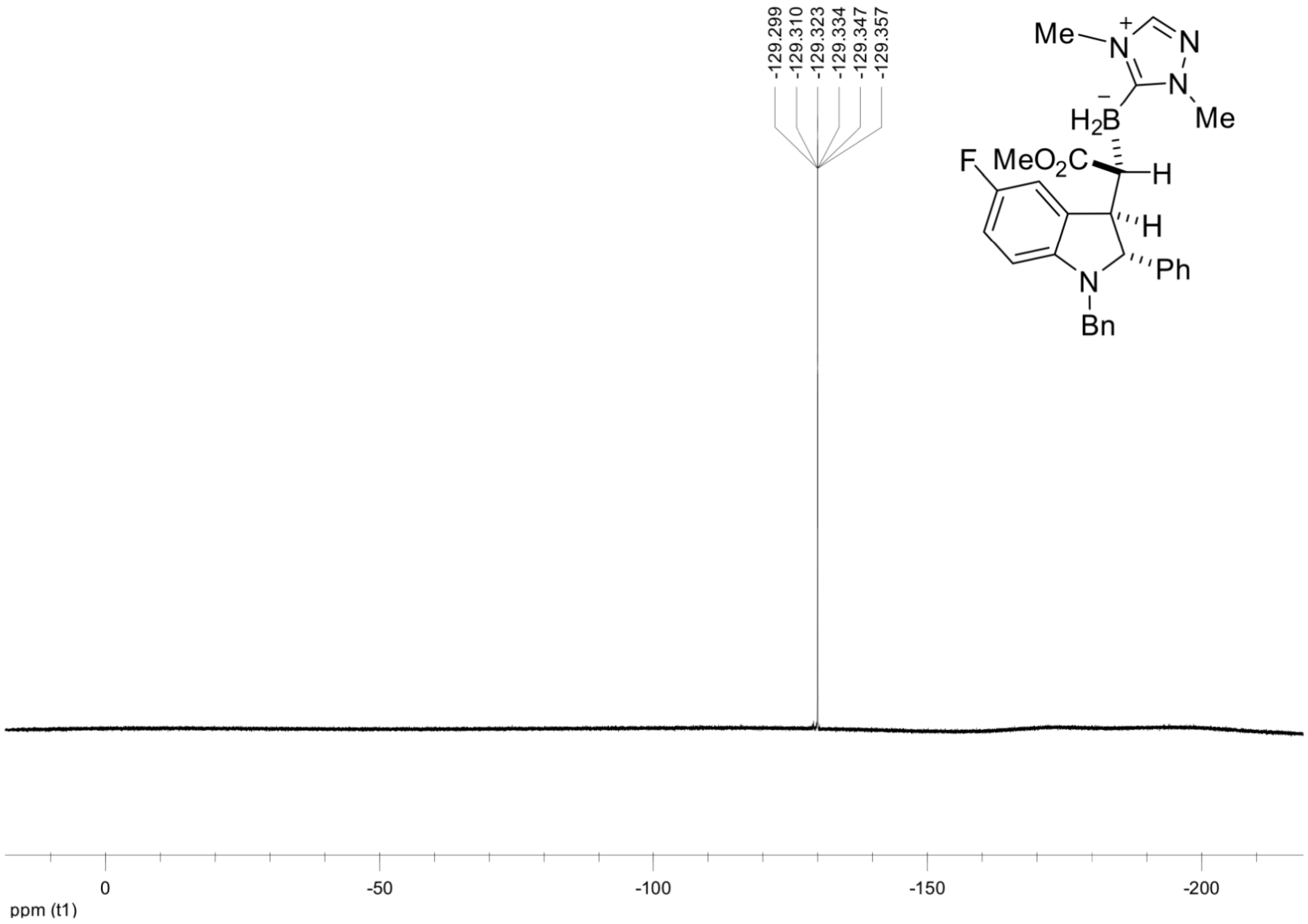

Supplementary Figure 130. ^{19}F NMR spectrum of **4ha** (376 MHz, $CDCl_3$)

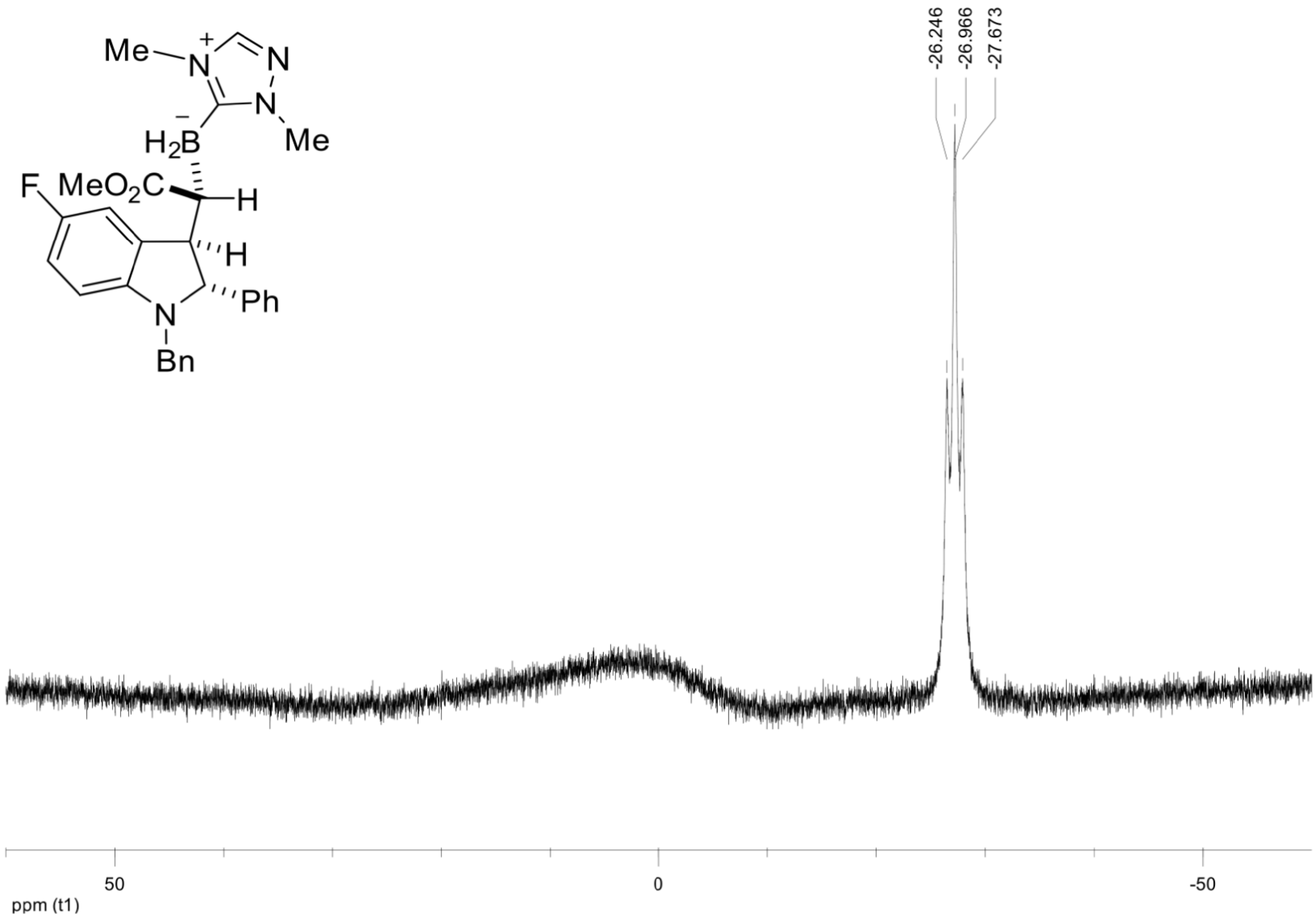

Supplementary Figure 131. ^{11}B NMR spectrum of **4ha** (128.4 MHz, $CDCl_3$)

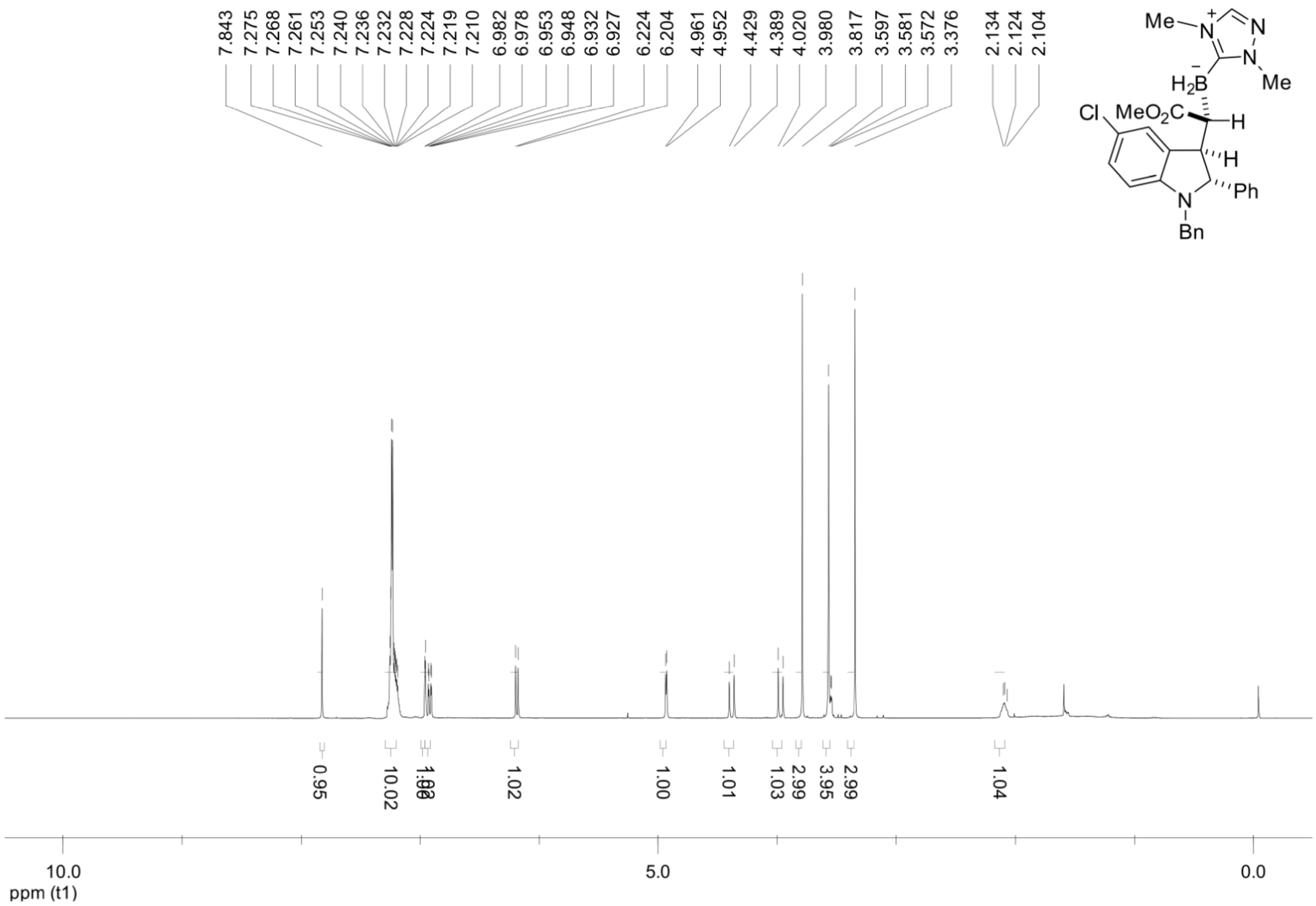

Supplementary Figure 132. ^{1}H NMR spectrum of **4ia** (400 MHz, $CDCl_3$)

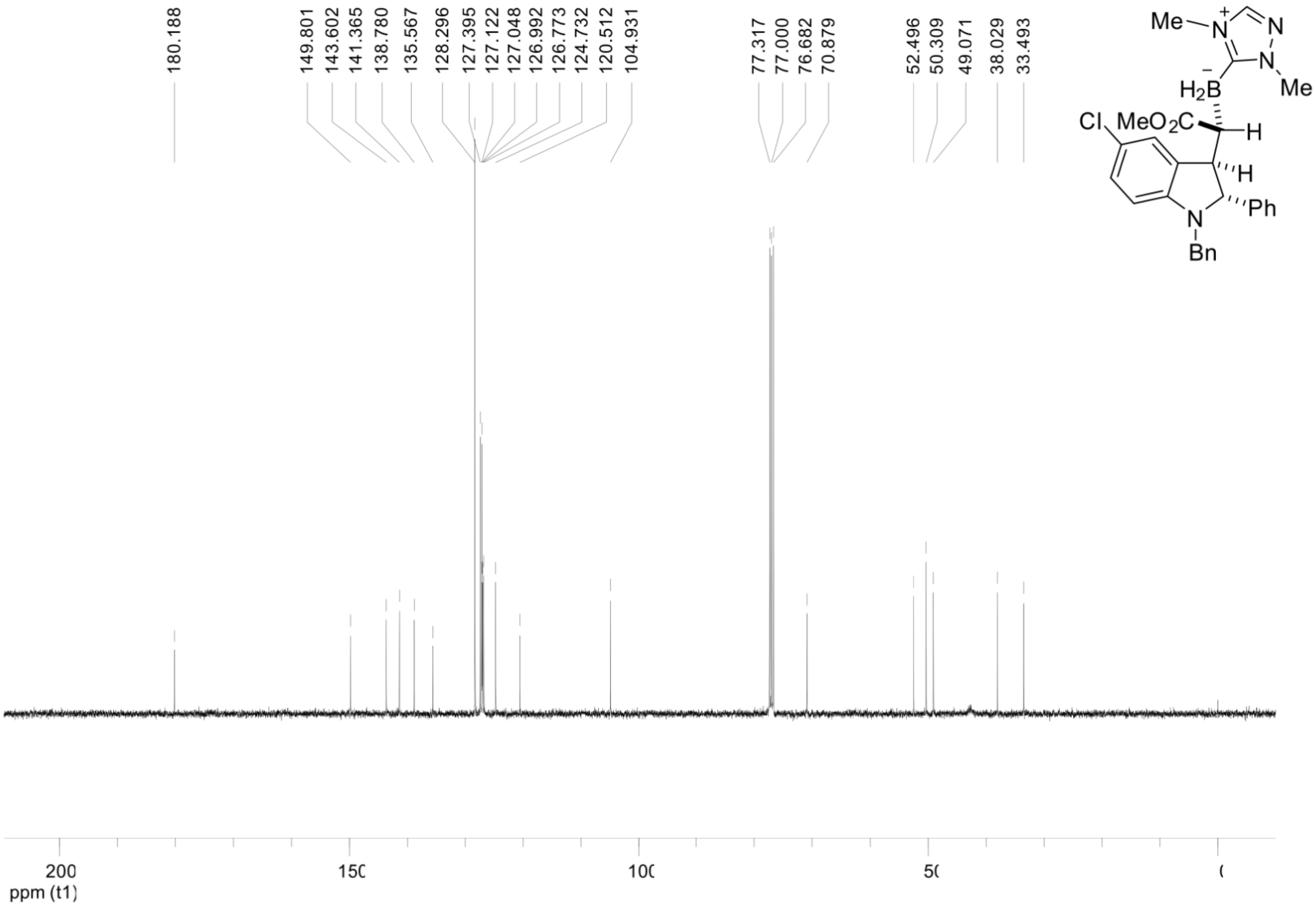

Supplementary Figure 133. ^{13}C NMR spectrum of **4ia** (100 MHz, $CDCl_3$)

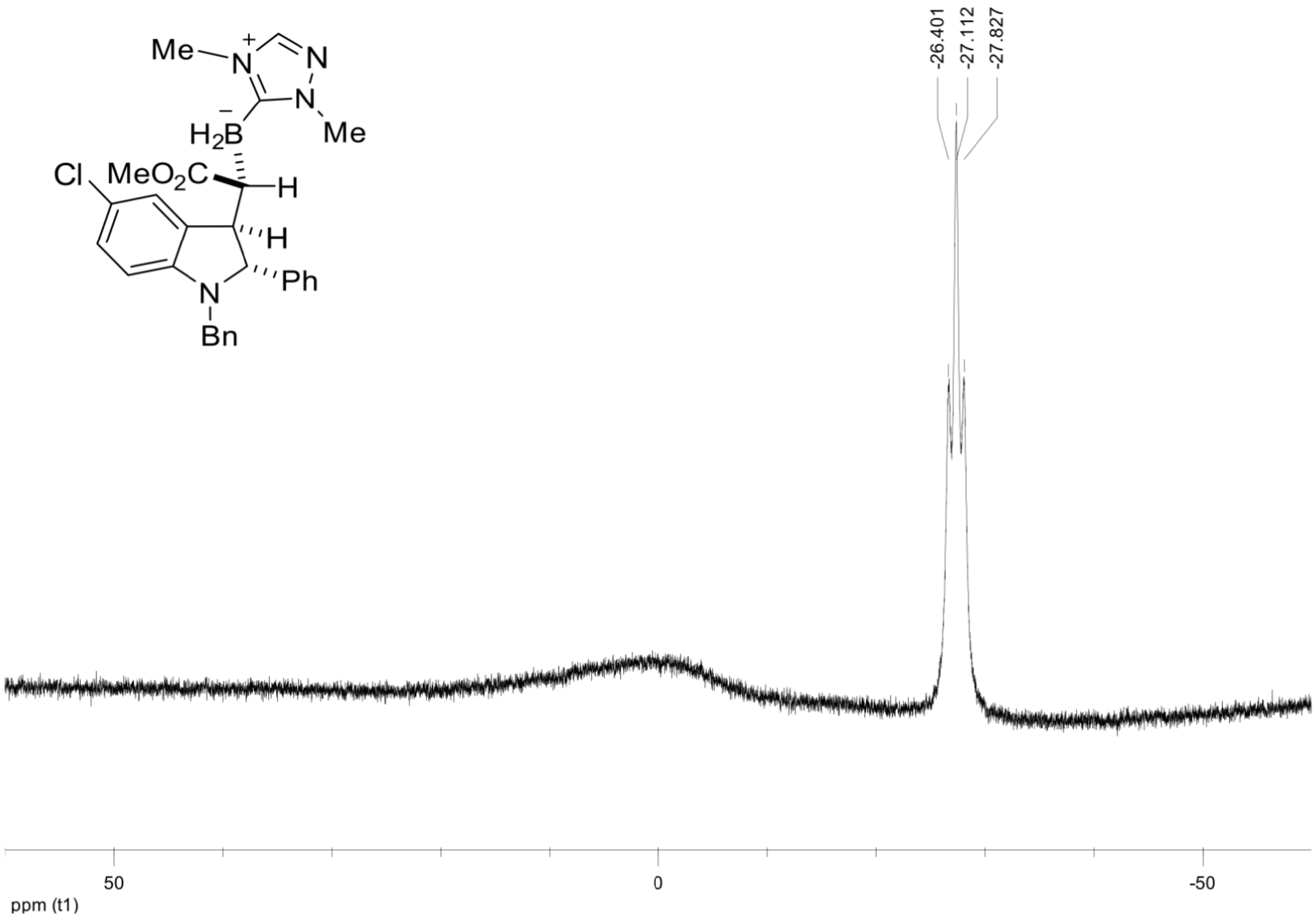

Supplementary Figure 134. ^{11}B NMR spectrum of **4ia** (128.4 MHz, $CDCl_3$)

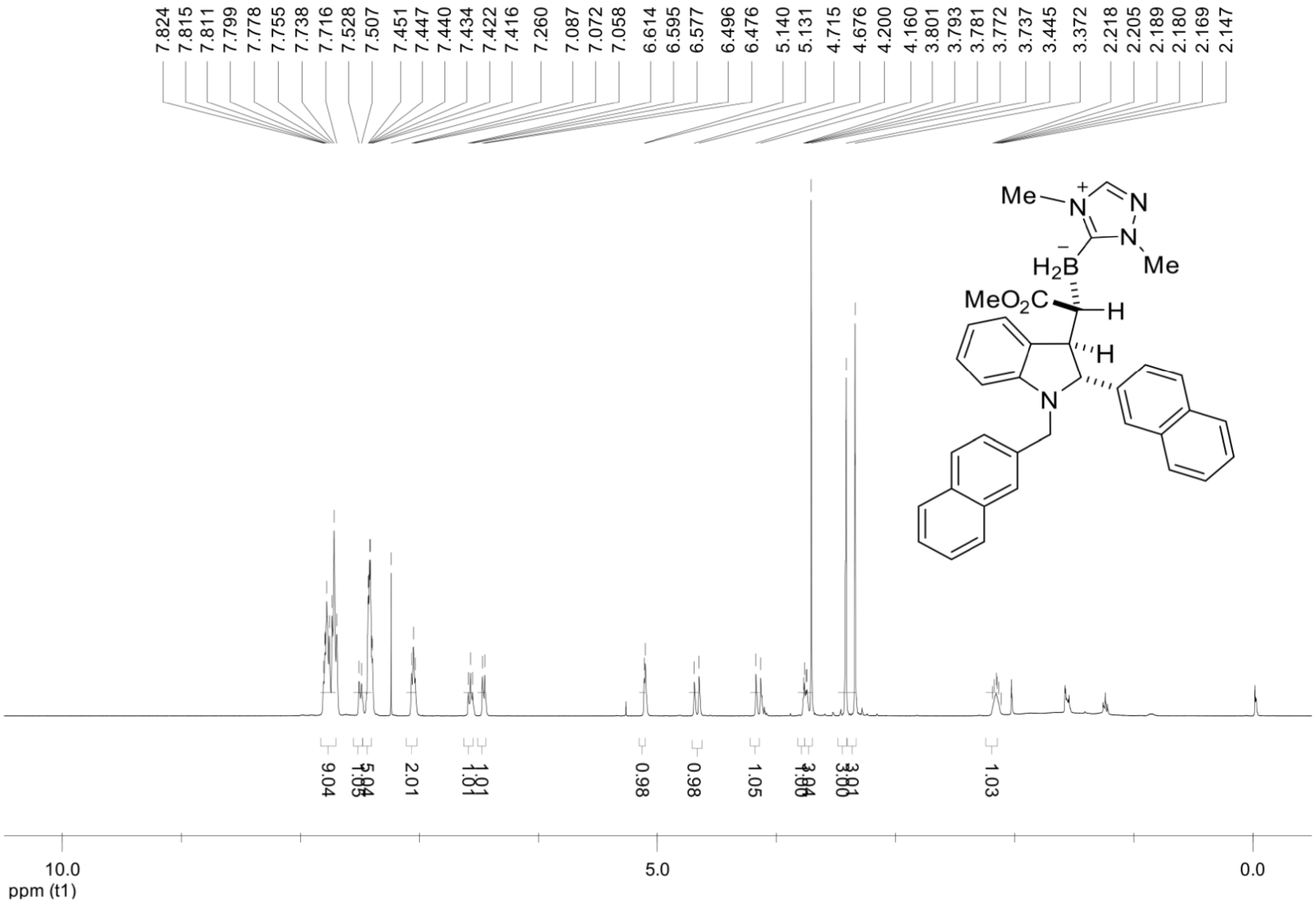

Supplementary Figure 135. ^{1}H NMR spectrum of **4ta** (400 MHz, $CDCl_3$)

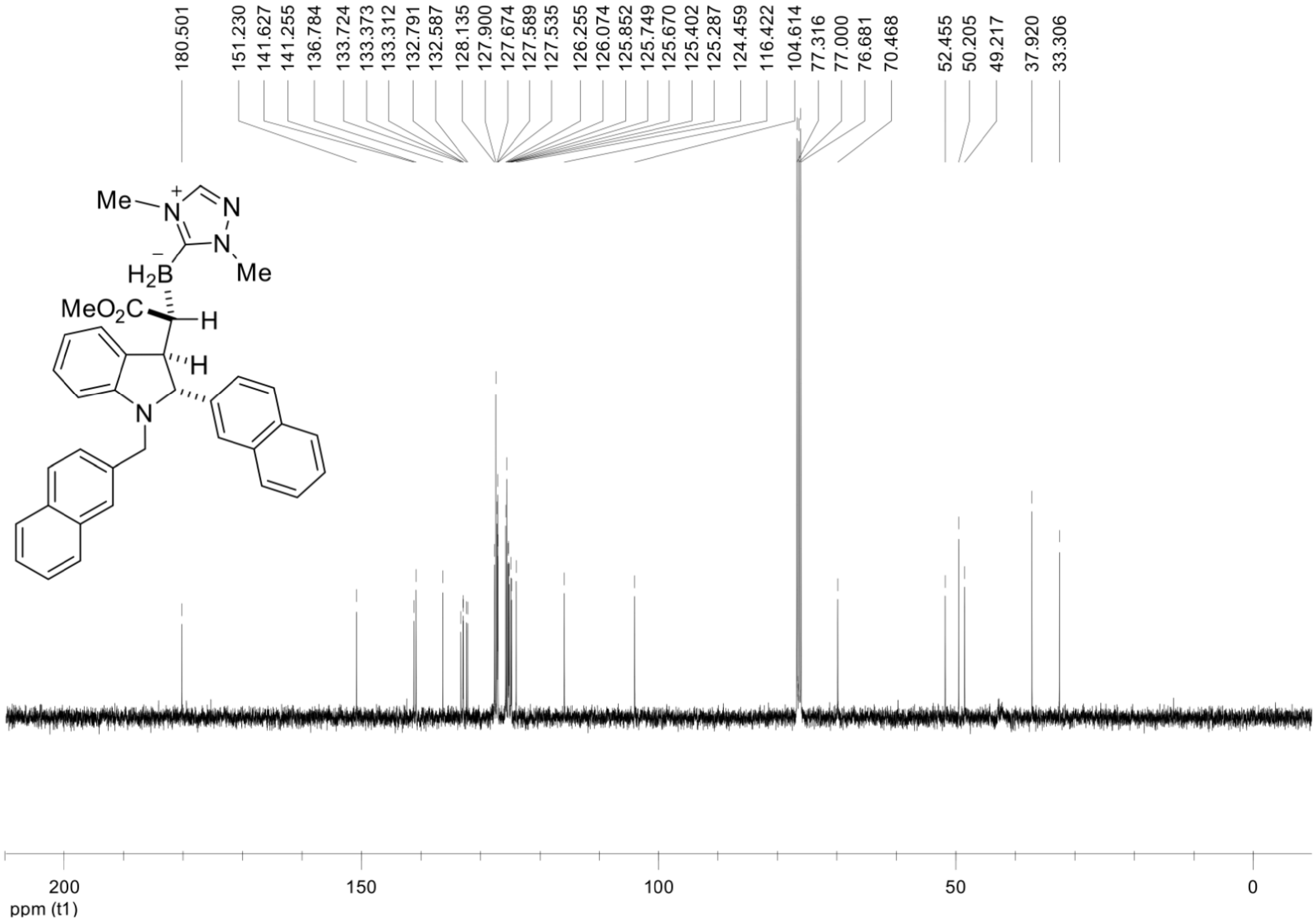

Supplementary Figure 136. ^{13}C NMR spectrum of **4ta** (100 MHz, $CDCl_3$)

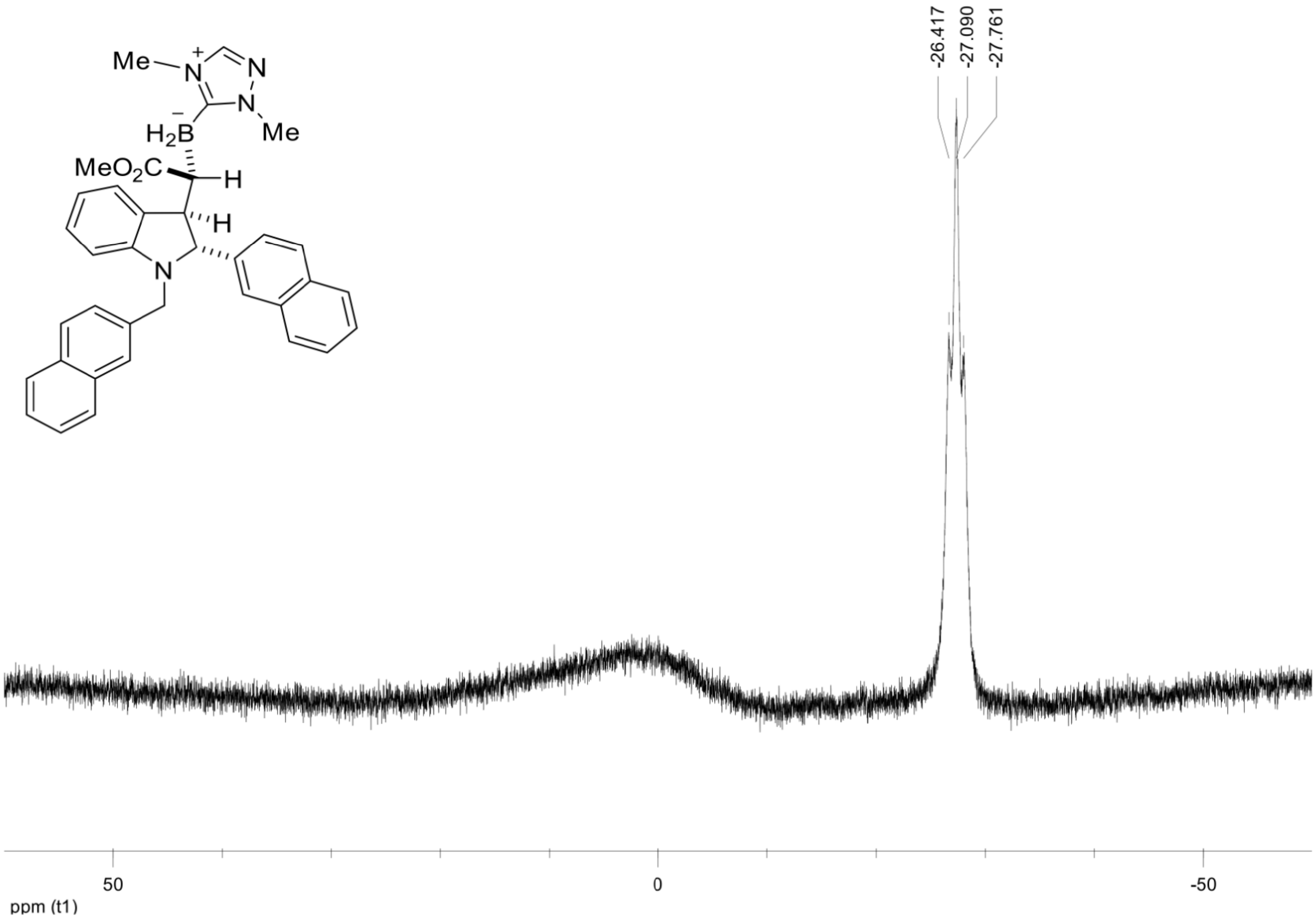

Supplementary Figure 137. ${}^{11}B$ NMR spectrum of **4ta** (128.4 MHz, $CDCl_3$)

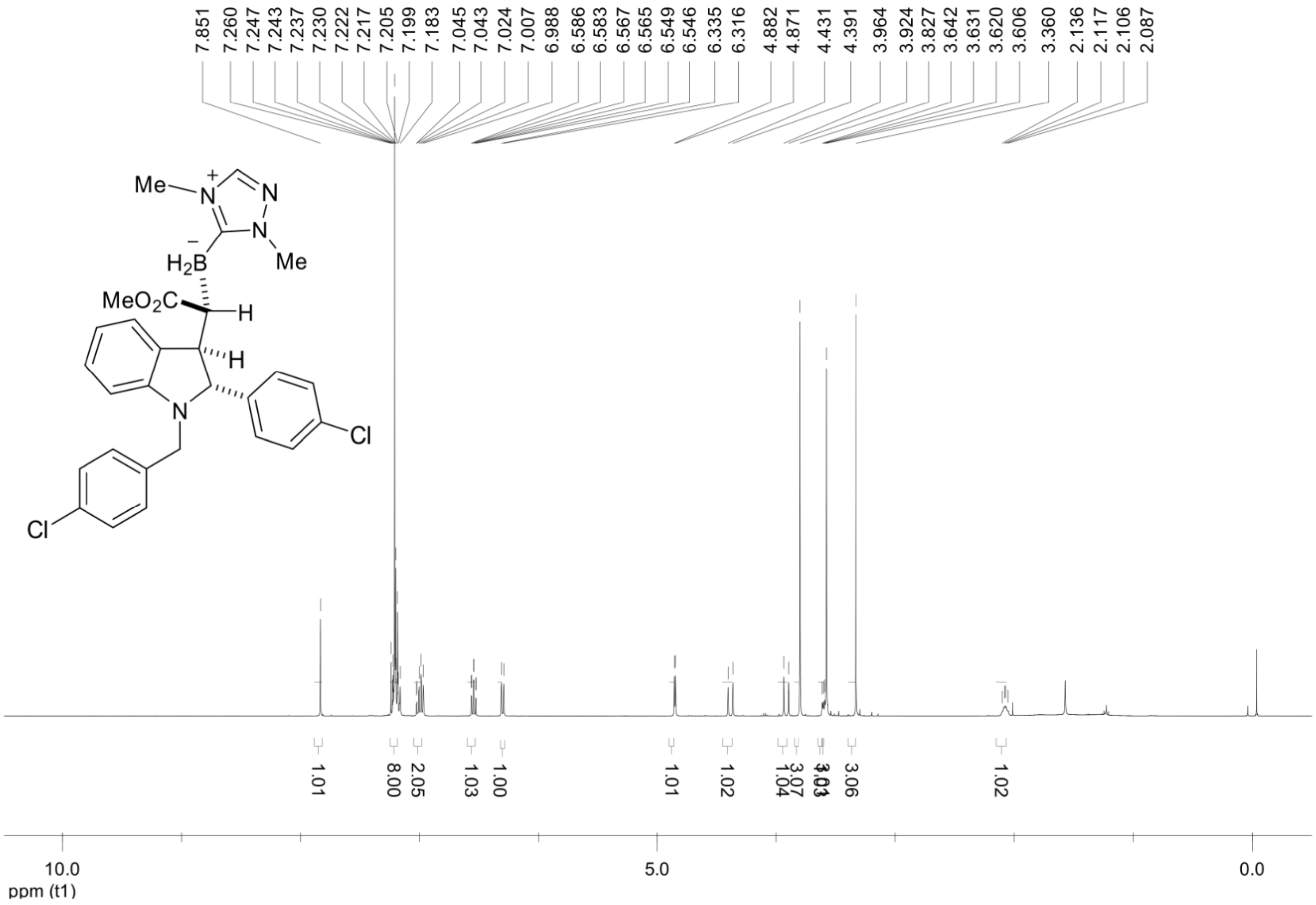

Supplementary Figure 138. ^{1}H NMR spectrum of **4ua** (400 MHz, $CDCl_3$)

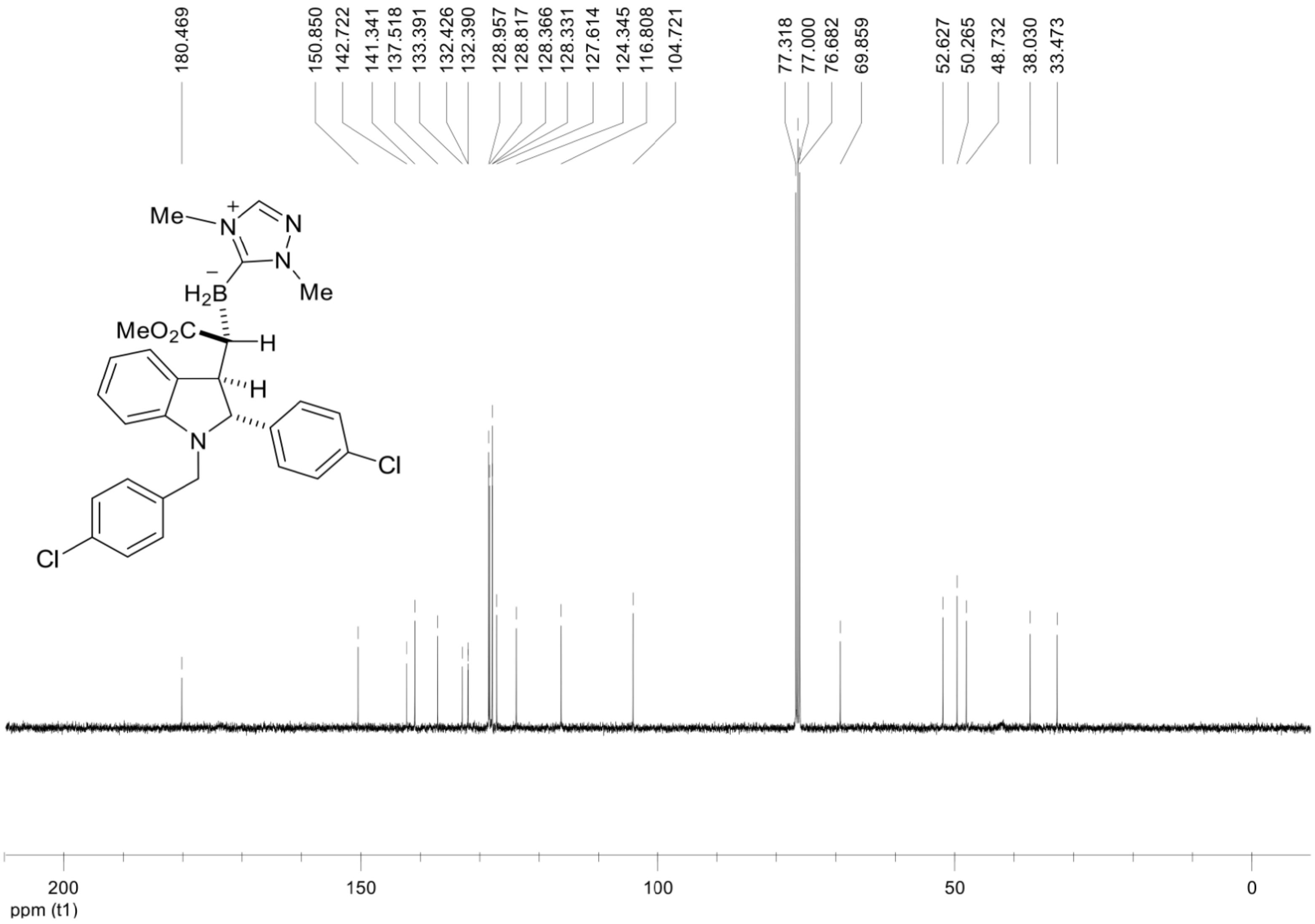

Supplementary Figure 139. ^{13}C NMR spectrum of **4ua** (100 MHz, $CDCl_3$)

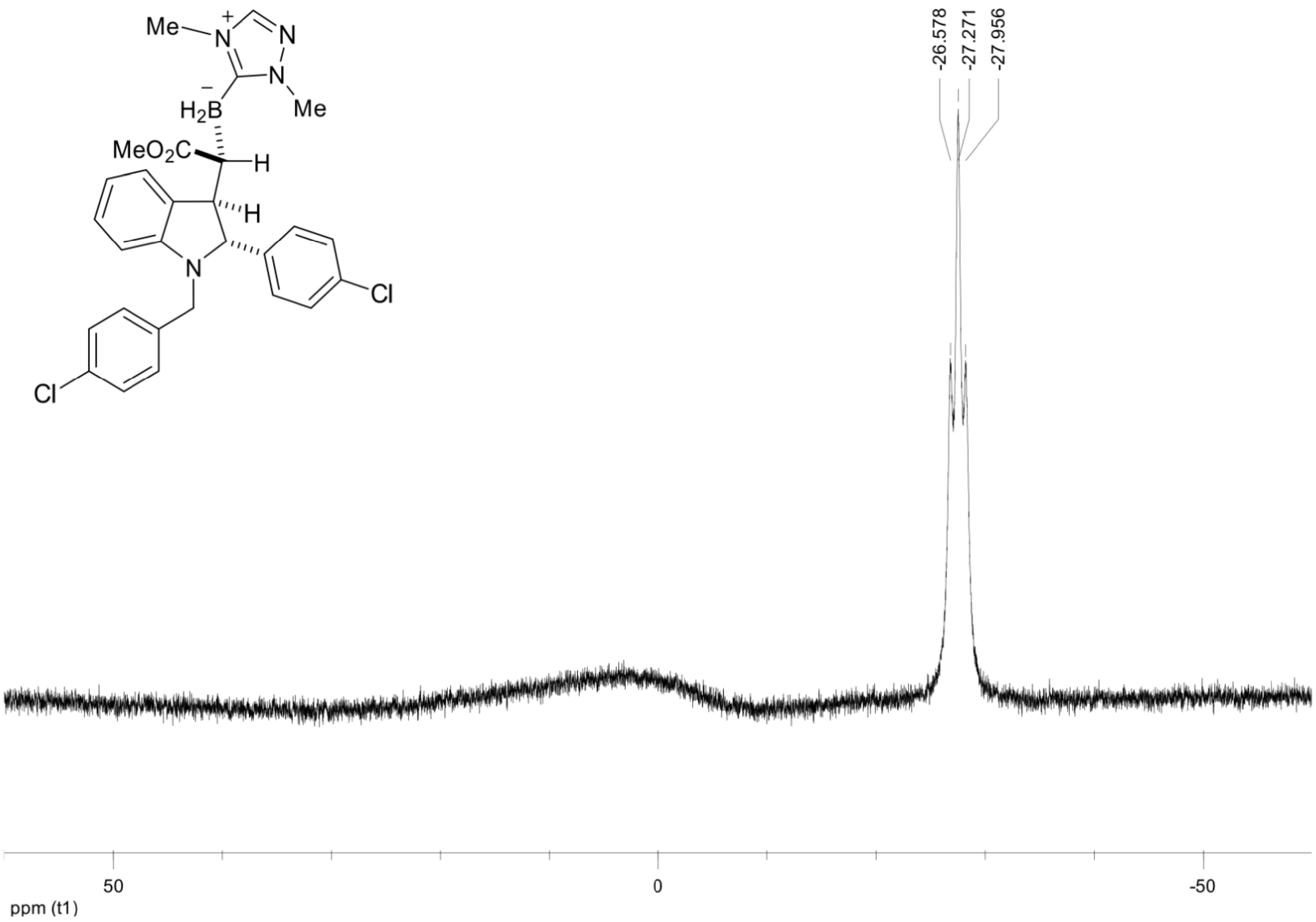

Supplementary Figure 140. ^{11}B NMR spectrum of **4ua** (128.4 MHz, $CDCl_3$)

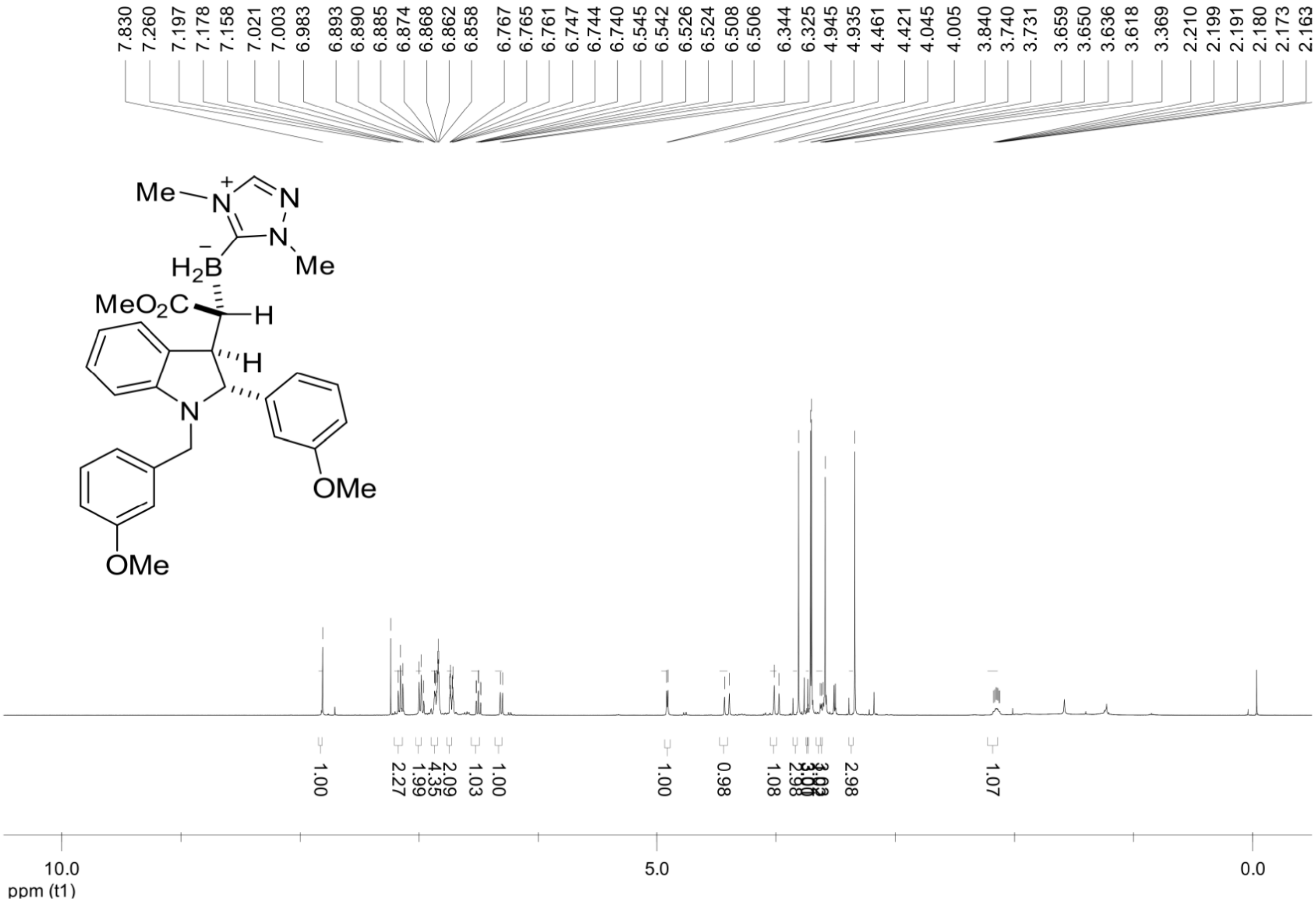

Supplementary Figure 141. ^{1}H NMR spectrum of **4va** (400 MHz, $CDCl_3$)

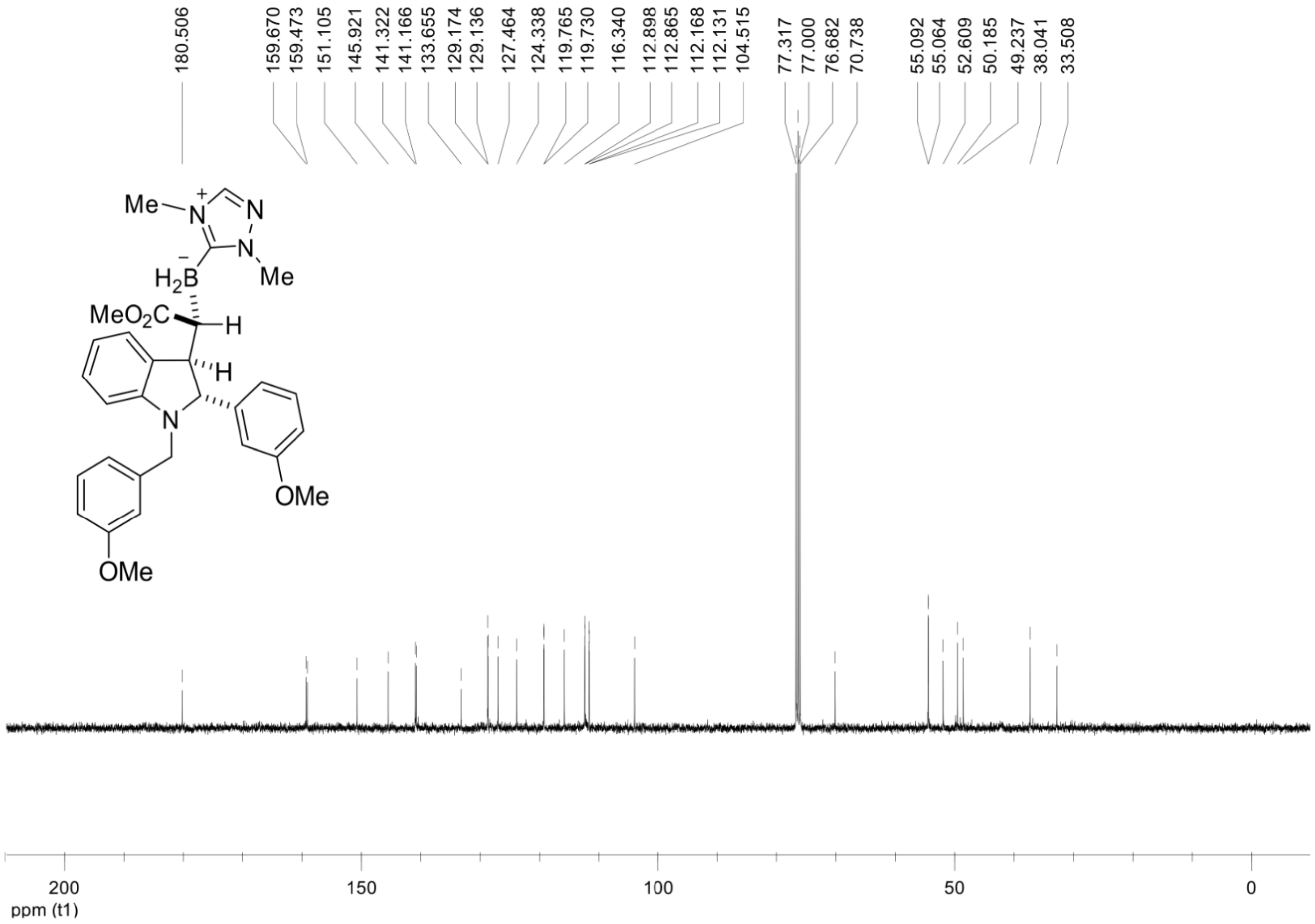

Supplementary Figure 142. ^{13}C NMR spectrum of **4va** (100 MHz, $CDCl_3$)

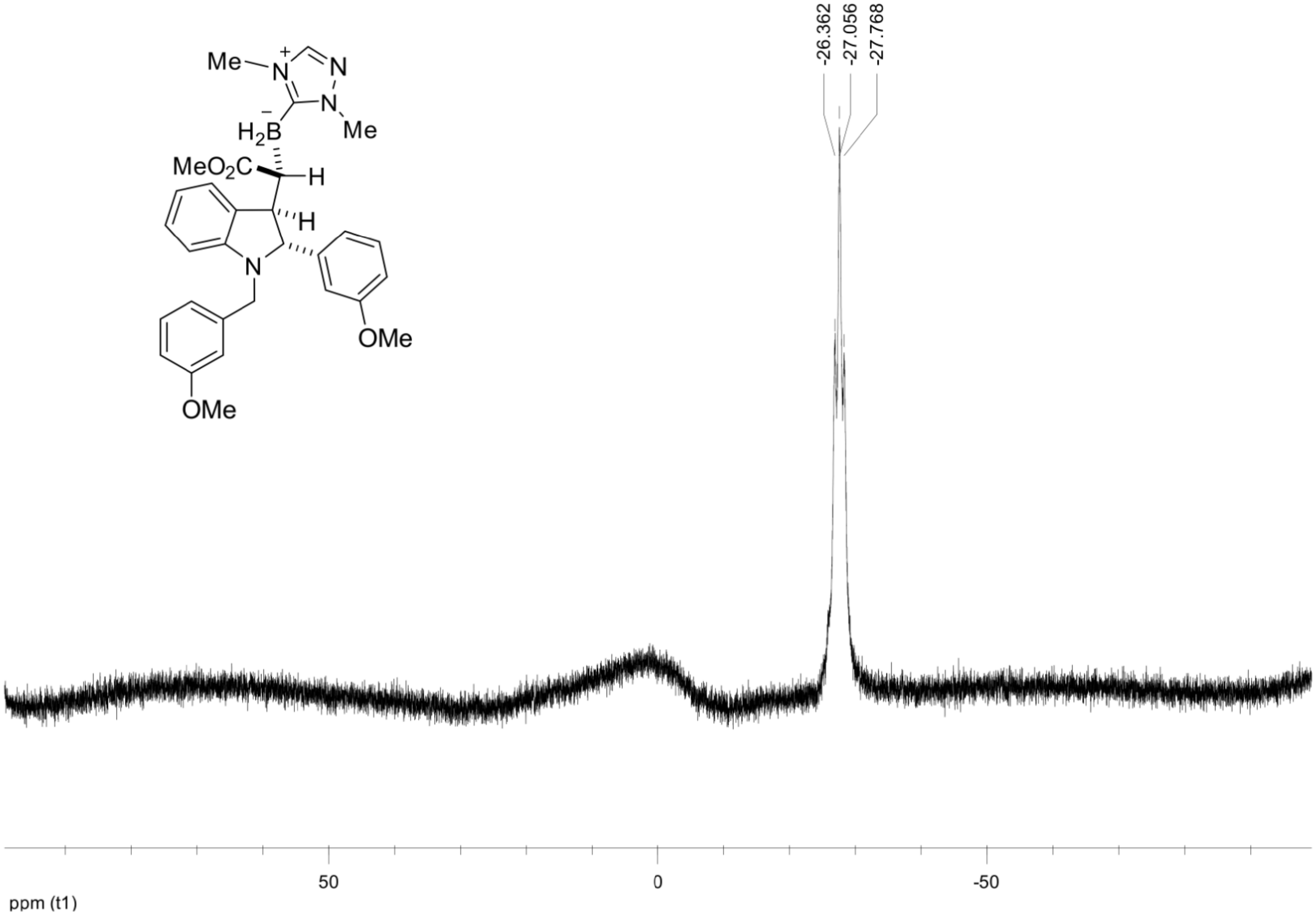

Supplementary Figure 143. ^{11}B NMR spectrum of **4va** (128.4 MHz, $CDCl_3$)

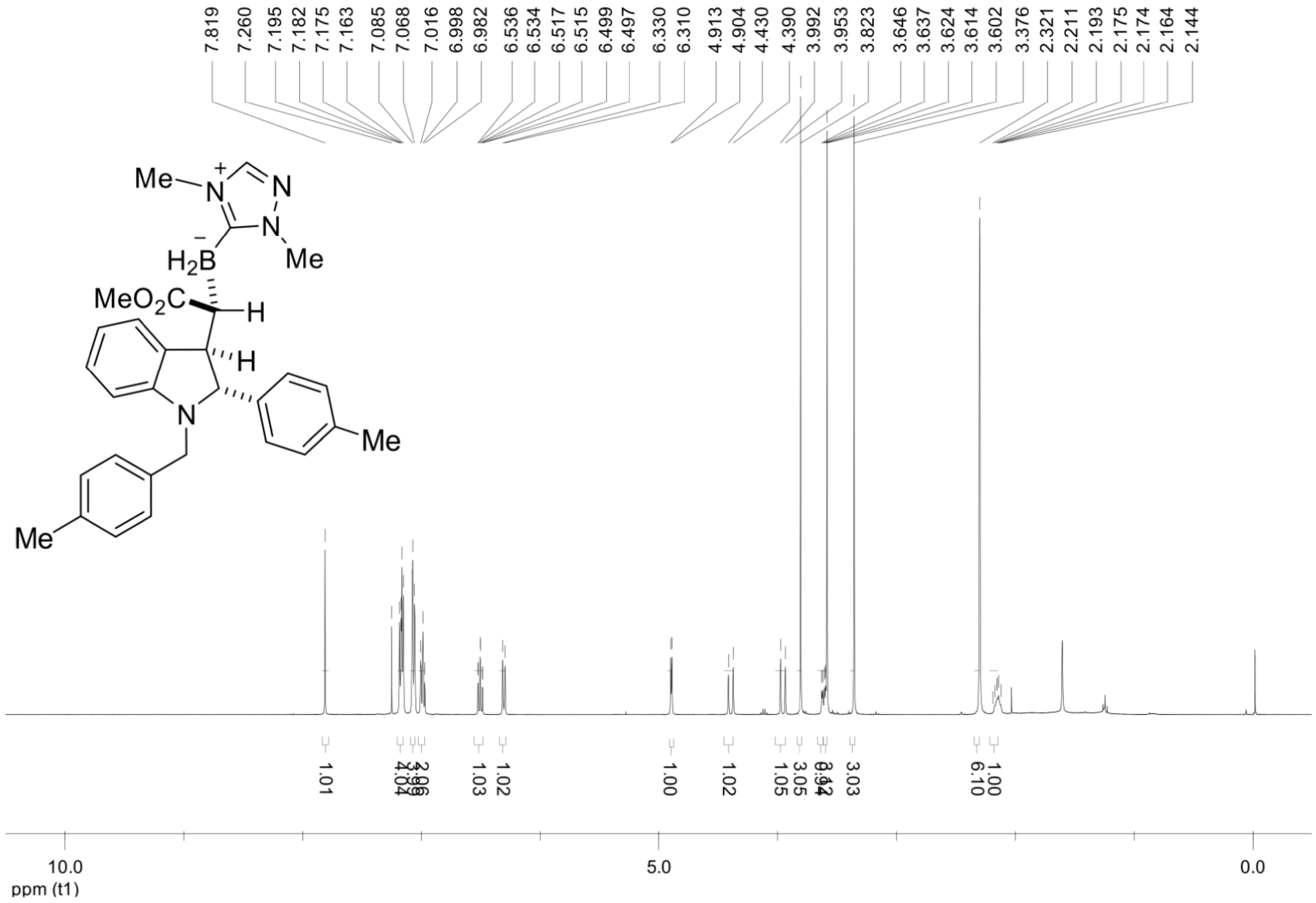

Supplementary Figure 144. ^{1}H NMR spectrum of **4wa** (400 MHz, $CDCl_3$)

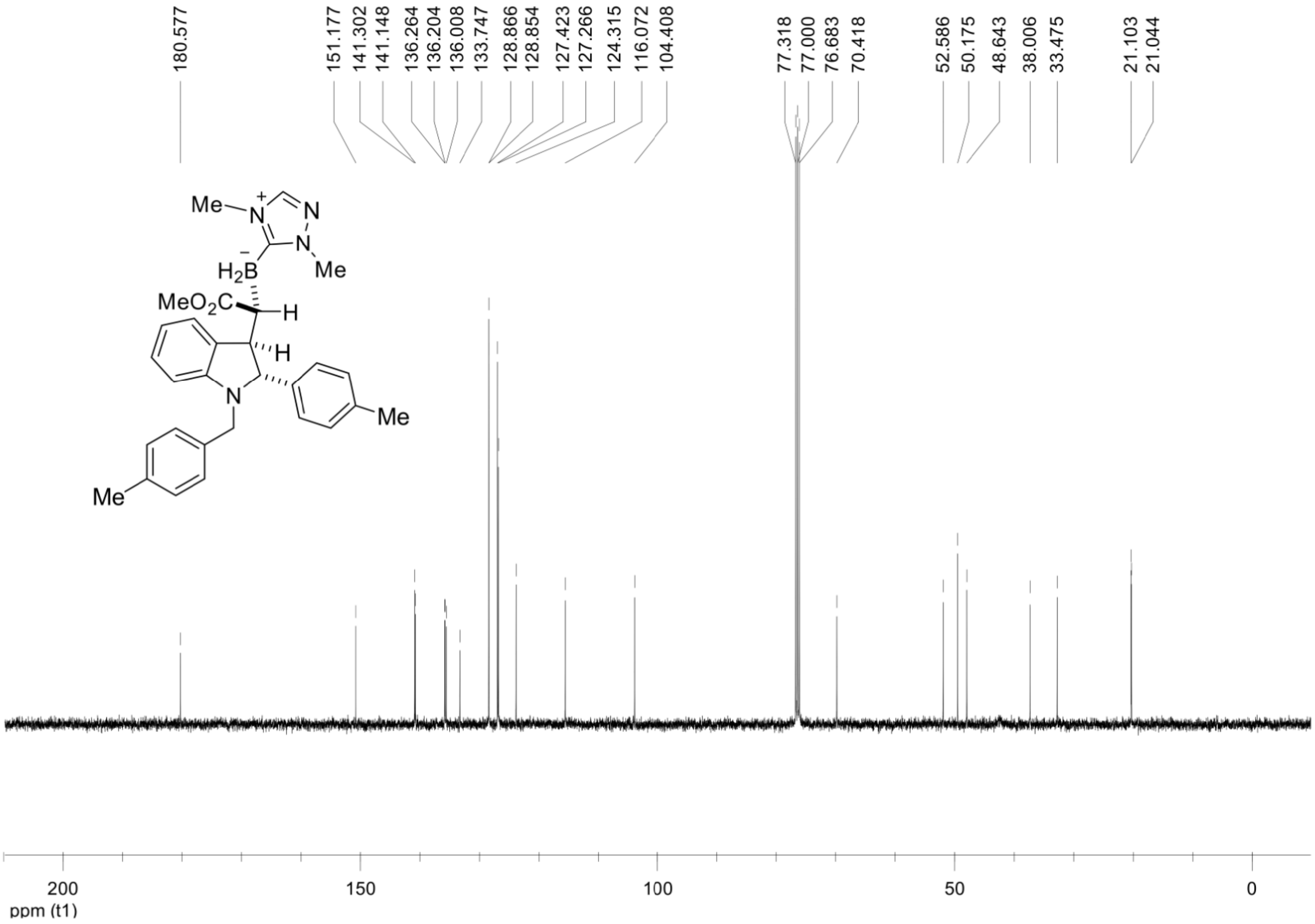

Supplementary Figure 145. ^{13}C NMR spectrum of **4wa** (100 MHz, $CDCl_3$)

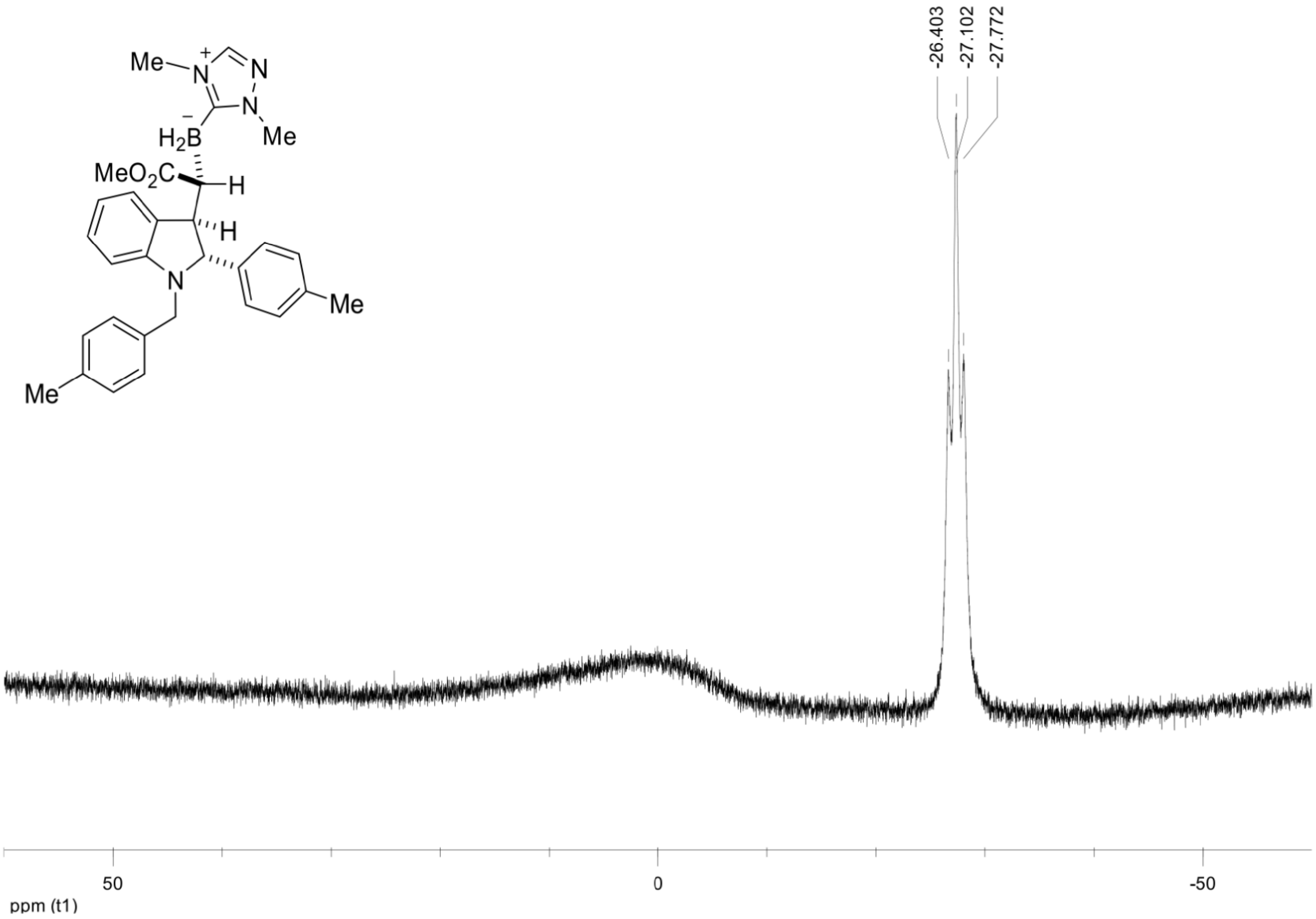

Supplementary Figure 146. ^{11}B NMR spectrum of **4wa** (128.4 MHz, $CDCl_3$)

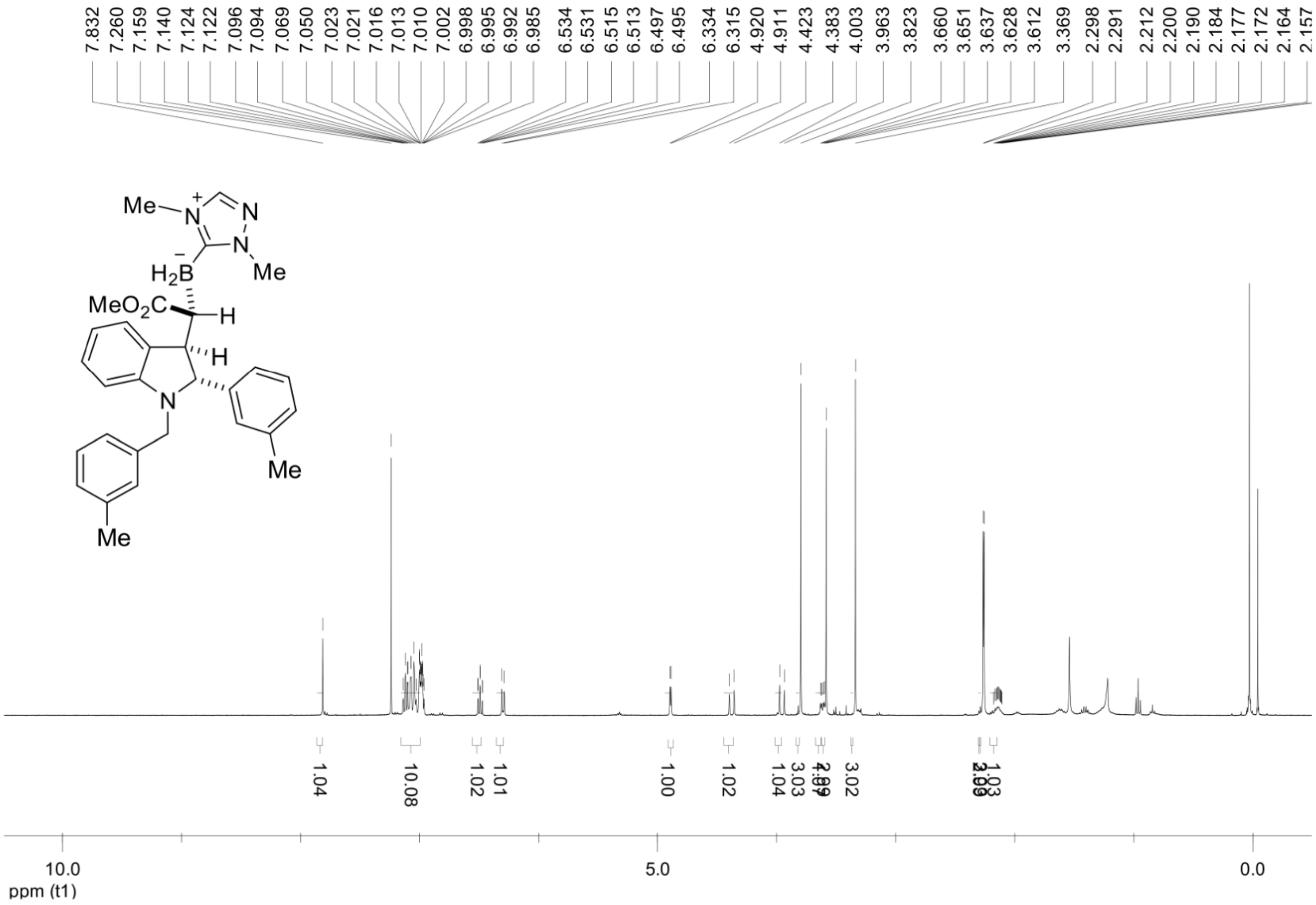

Supplementary Figure 147. ^{1}H NMR spectrum of **4xa** (400 MHz, $CDCl_3$)

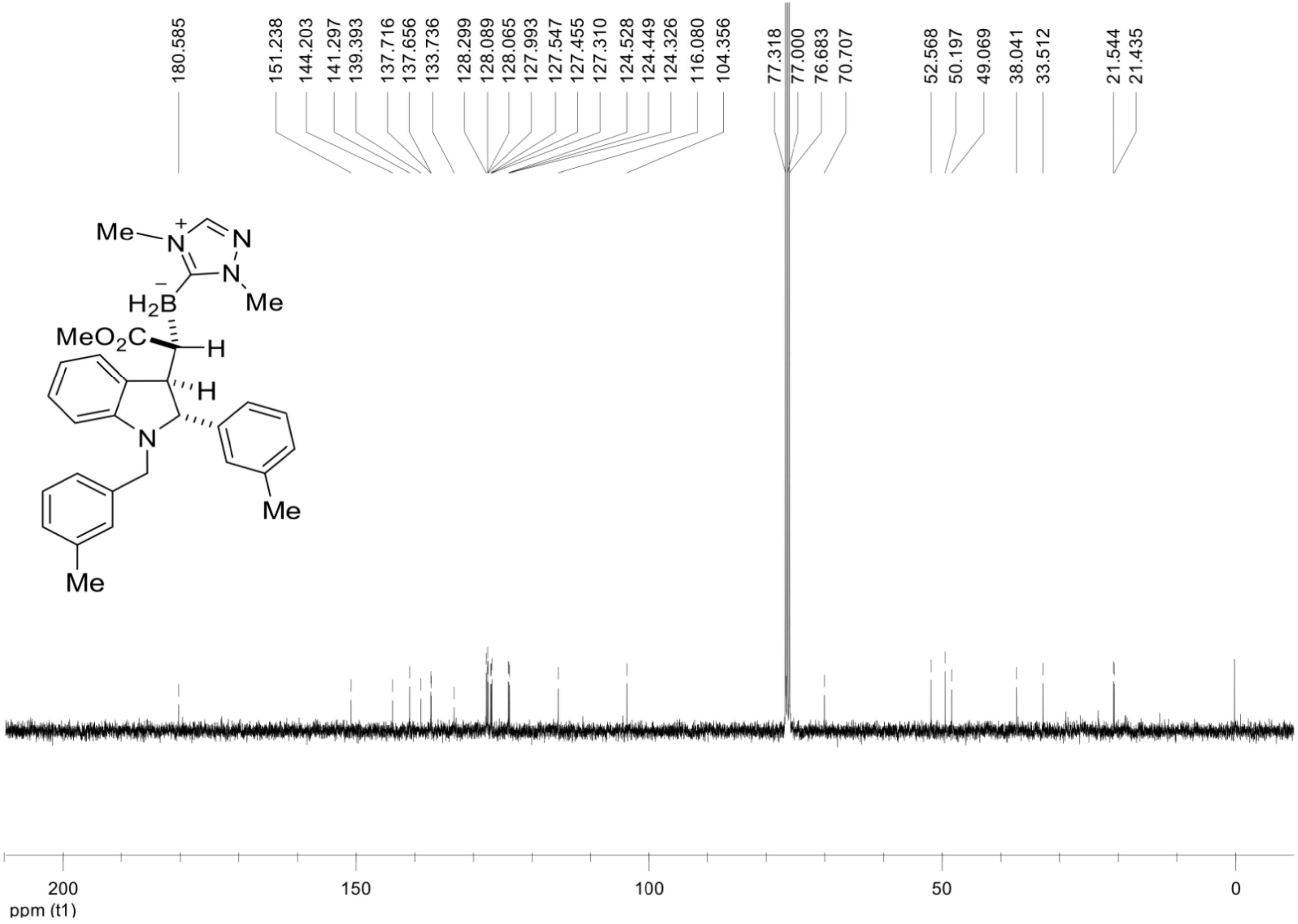

Supplementary Figure 148. ^{13}C NMR spectrum of **4xa** (100 MHz, $CDCl_3$)

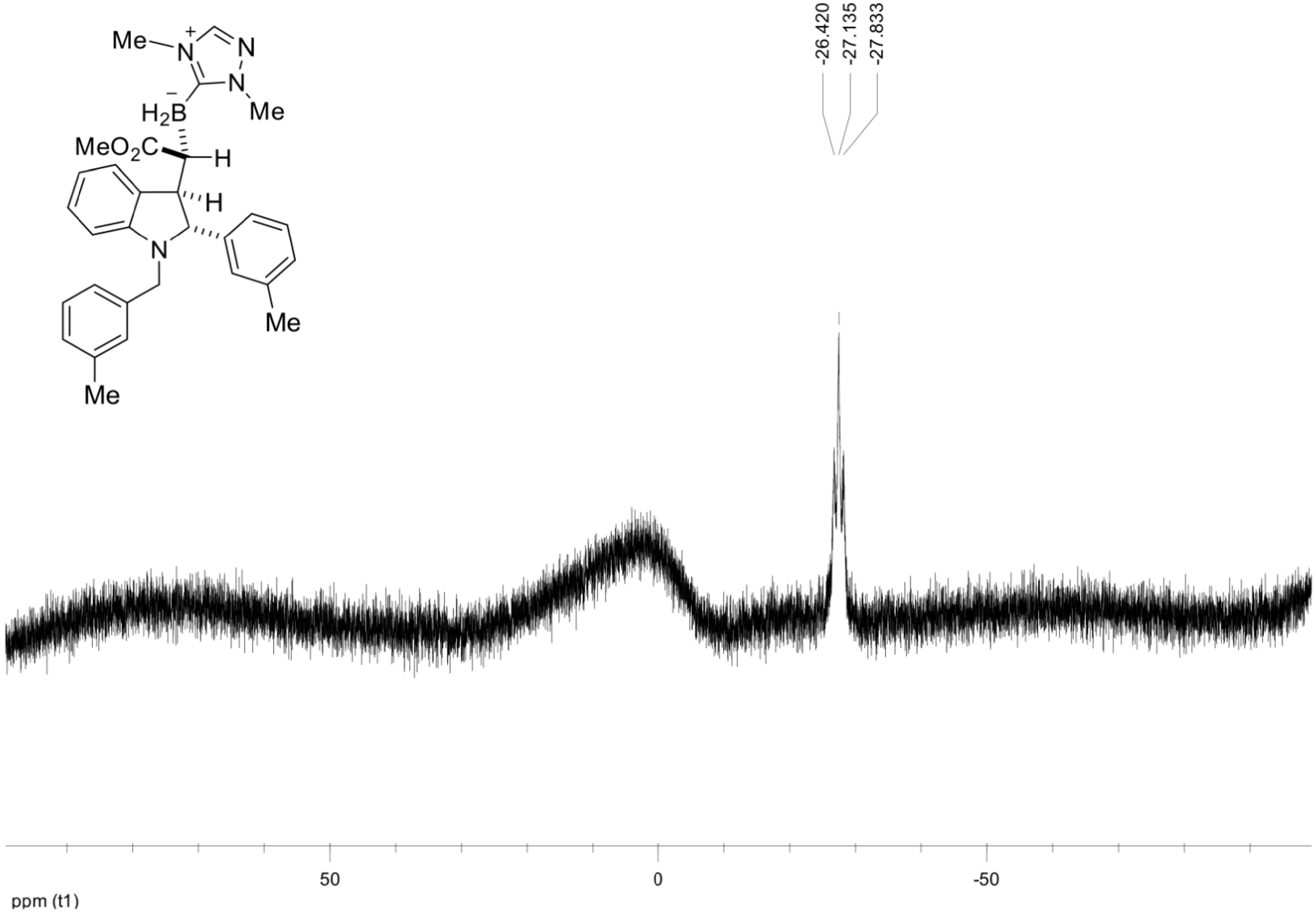

Supplementary Figure 149. ^{11}B NMR spectrum of **4xa** (128.4 MHz, $CDCl_3$)

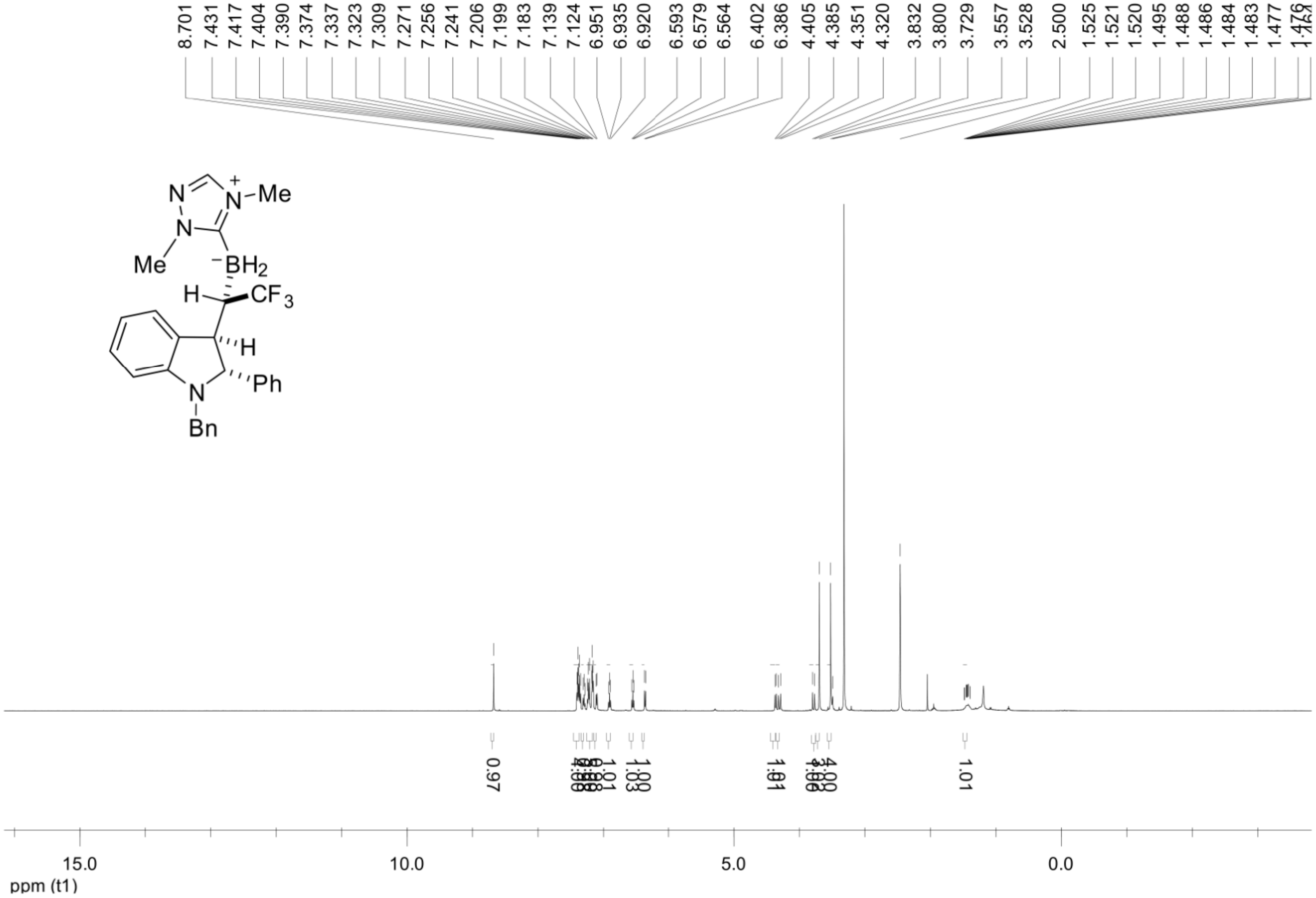

Supplementary Figure 150. ^{1}H NMR spectrum of **3sa** (500 MHz, DMSO-d_6)

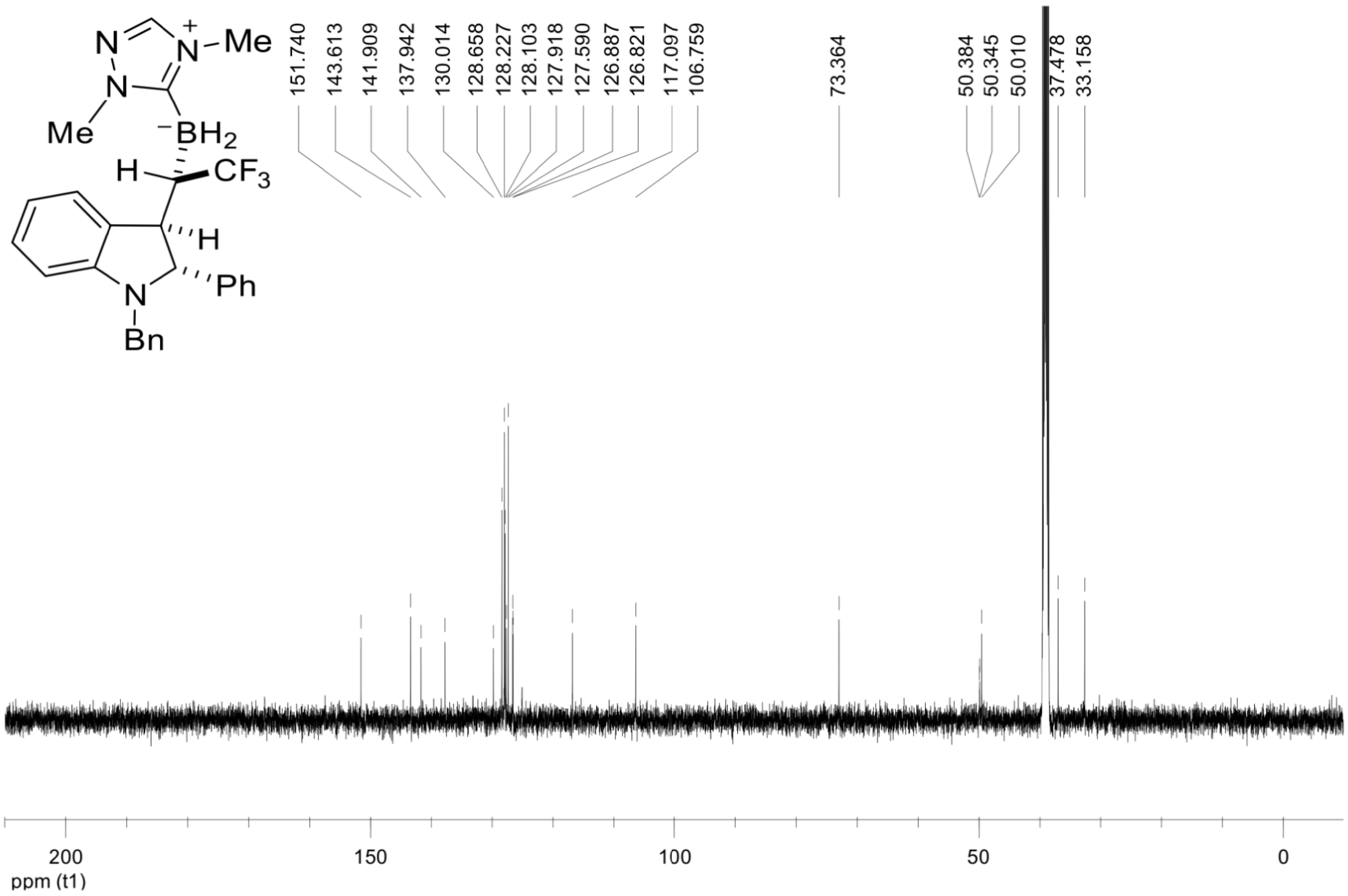

Supplementary Figure 151. ^{13}C NMR spectrum of **3sa** (125 MHz, DMSO-d_6)

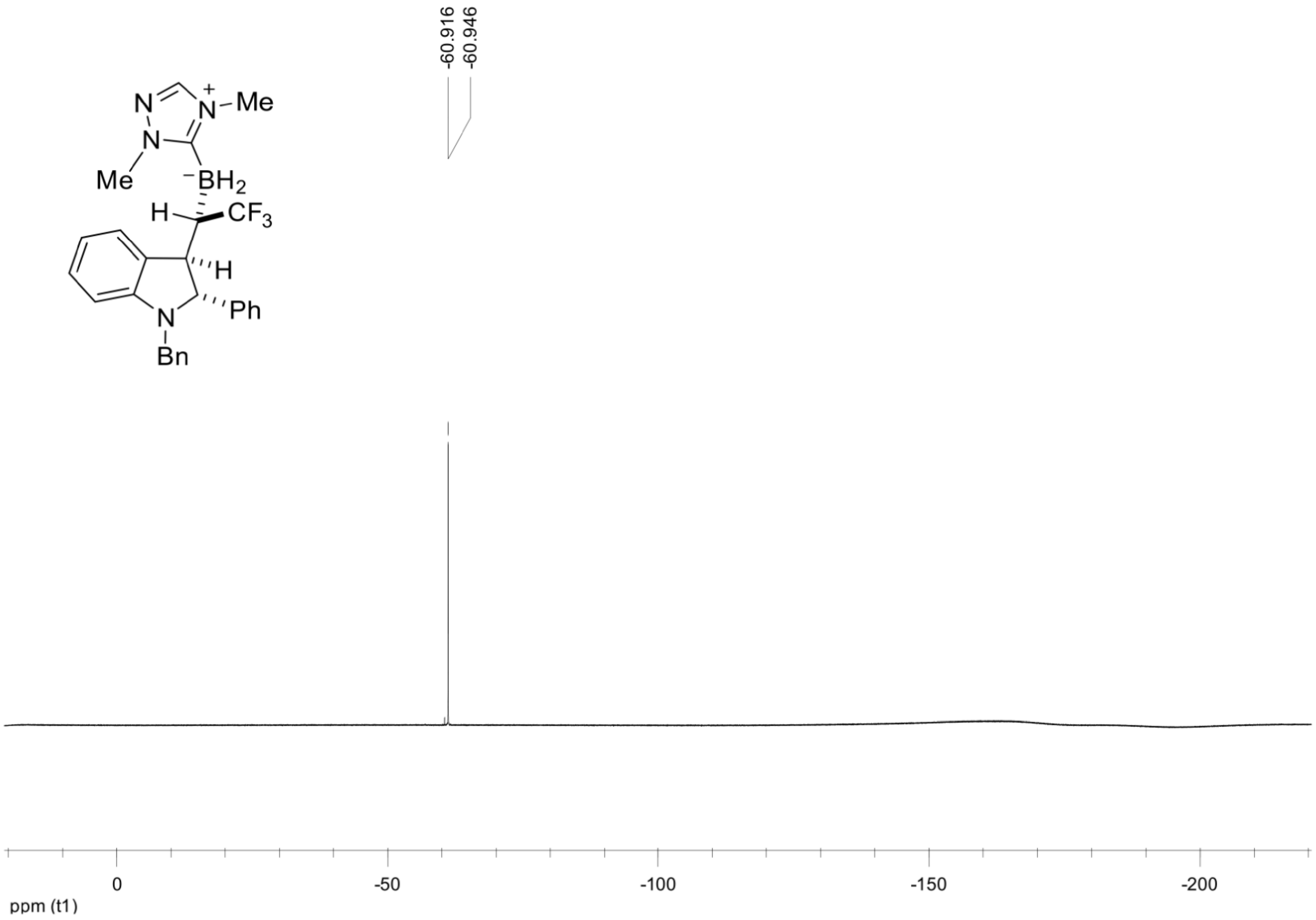

Supplementary Figure 152. ^{19}F NMR spectrum of **3sa** (470 MHz, DMSO-d_6)

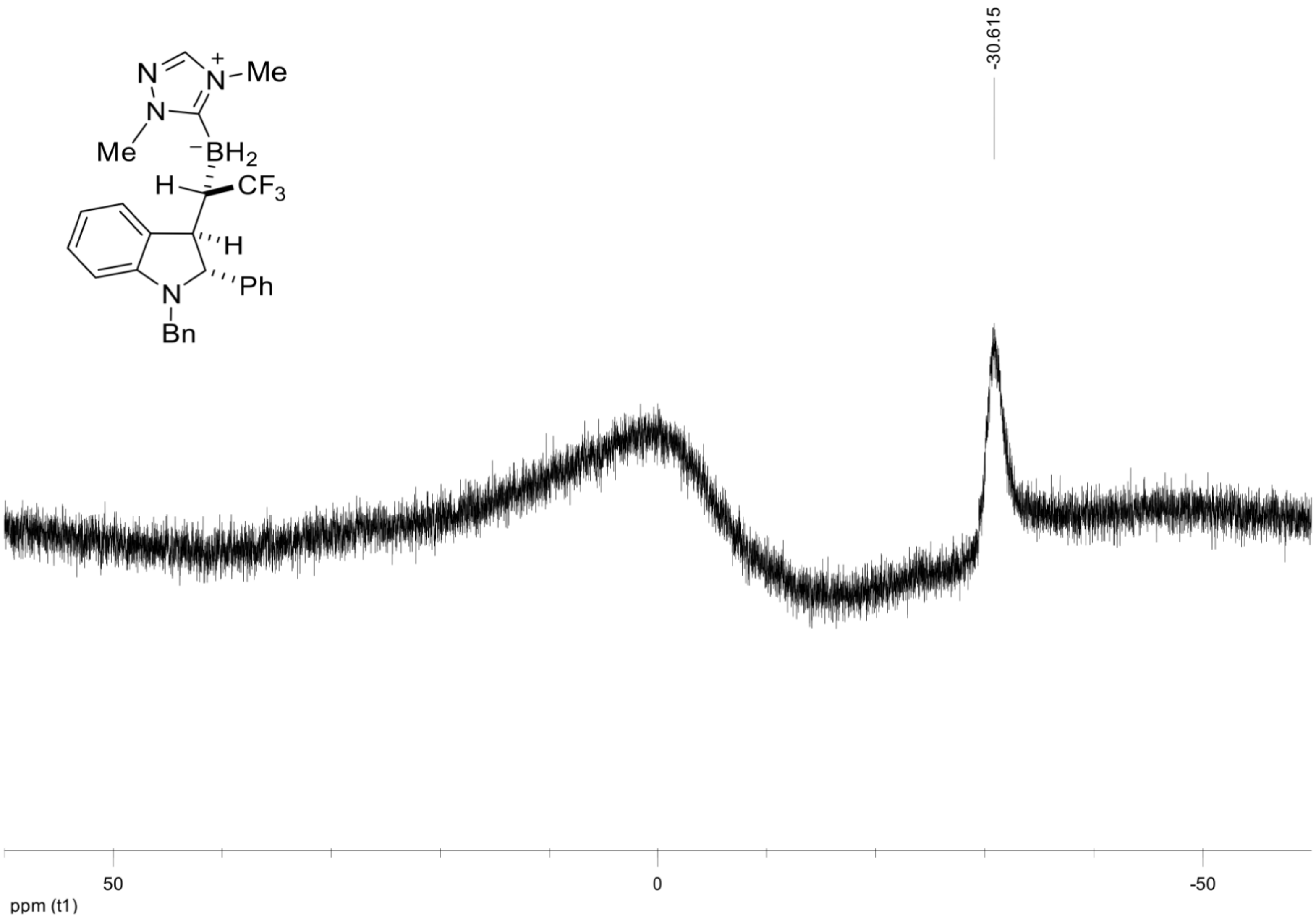

Supplementary Figure 153. ^{11}B NMR spectrum of **3sa** (160.5 MHz, DMSO-d_6)

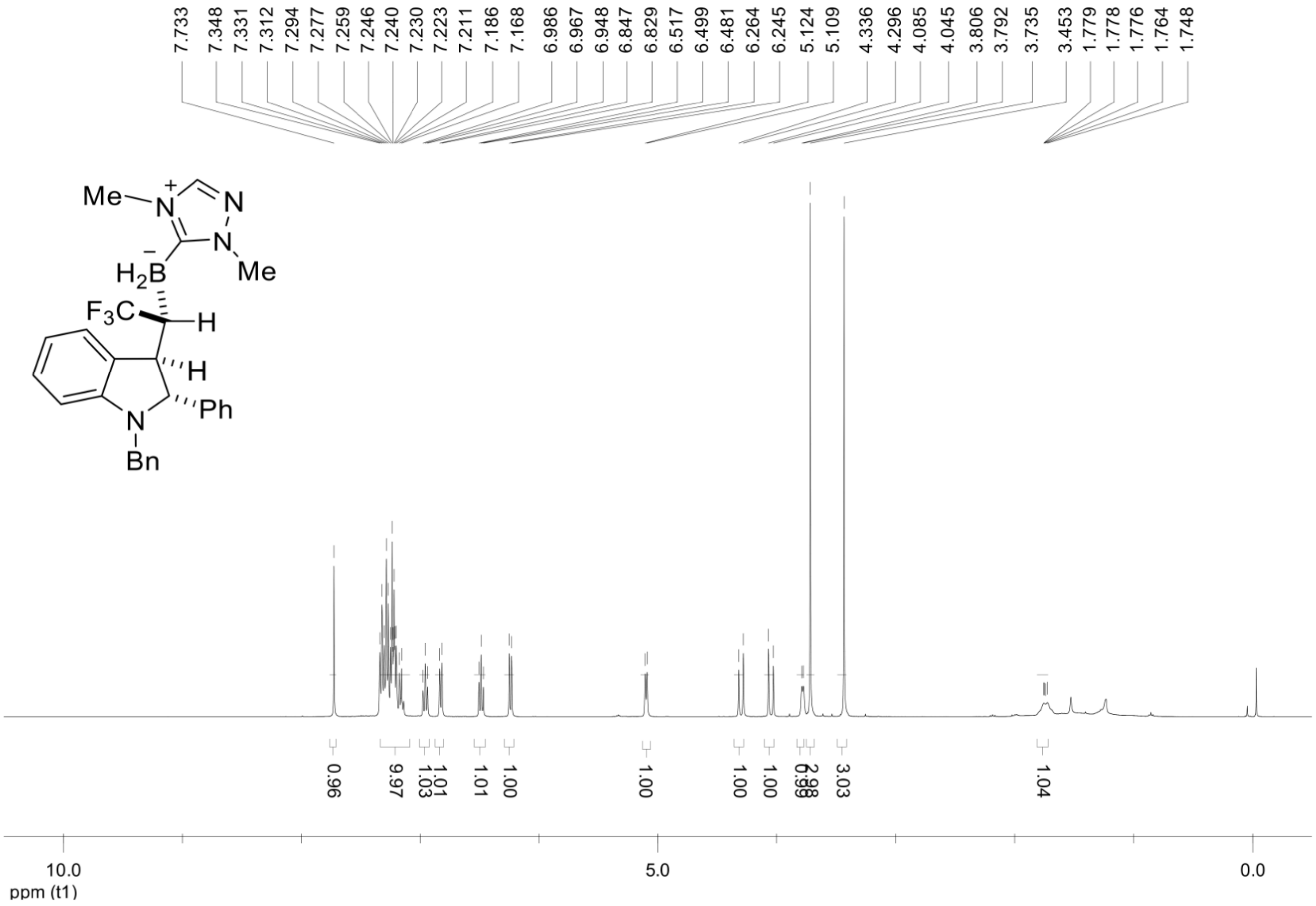

Supplementary Figure 154. ^{1}H NMR spectrum of **4sa** (400 MHz, $CDCl_3$)

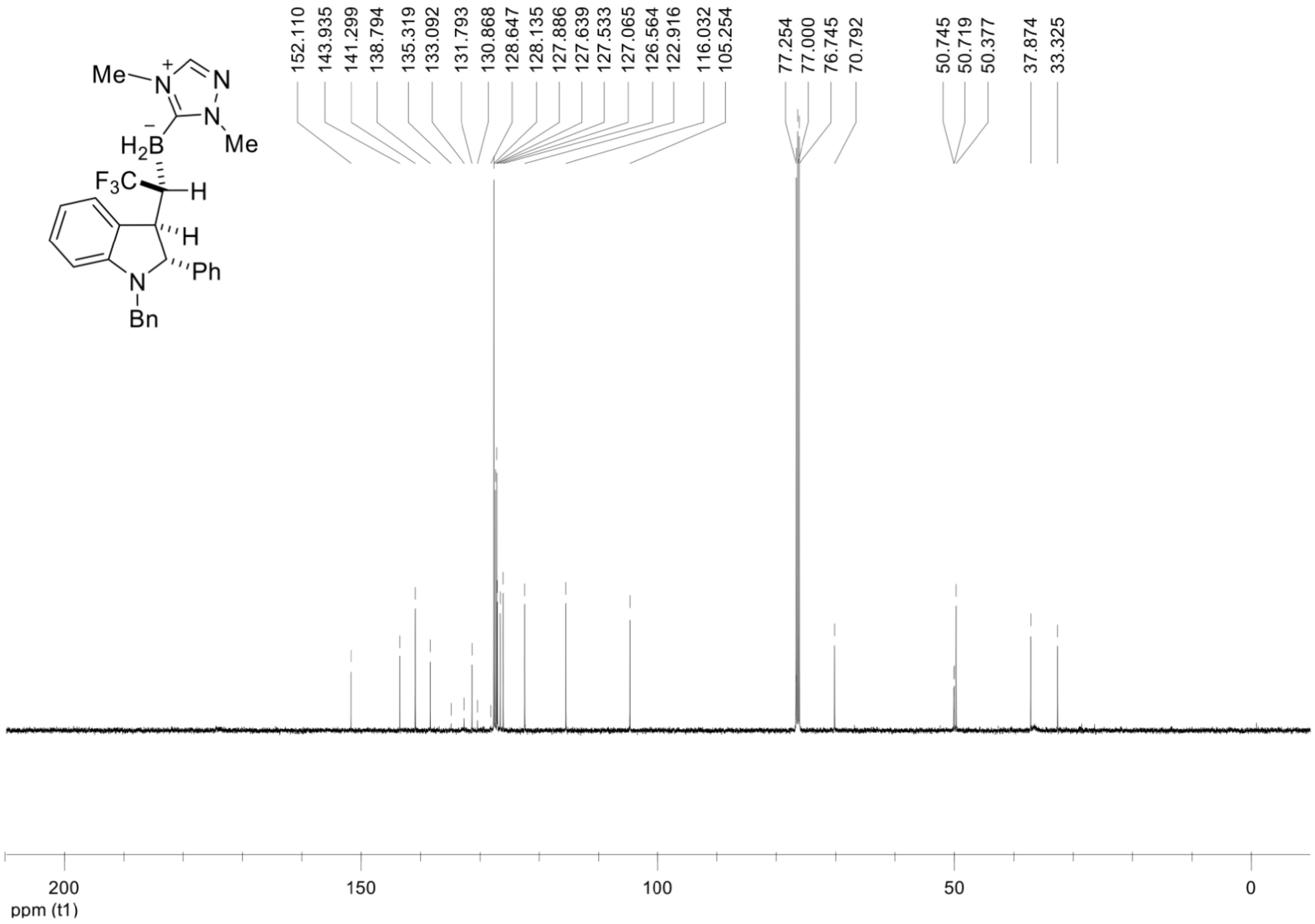

Supplementary Figure 155. ^{13}C NMR spectrum of **4sa** (125 MHz, $CDCl_3$)

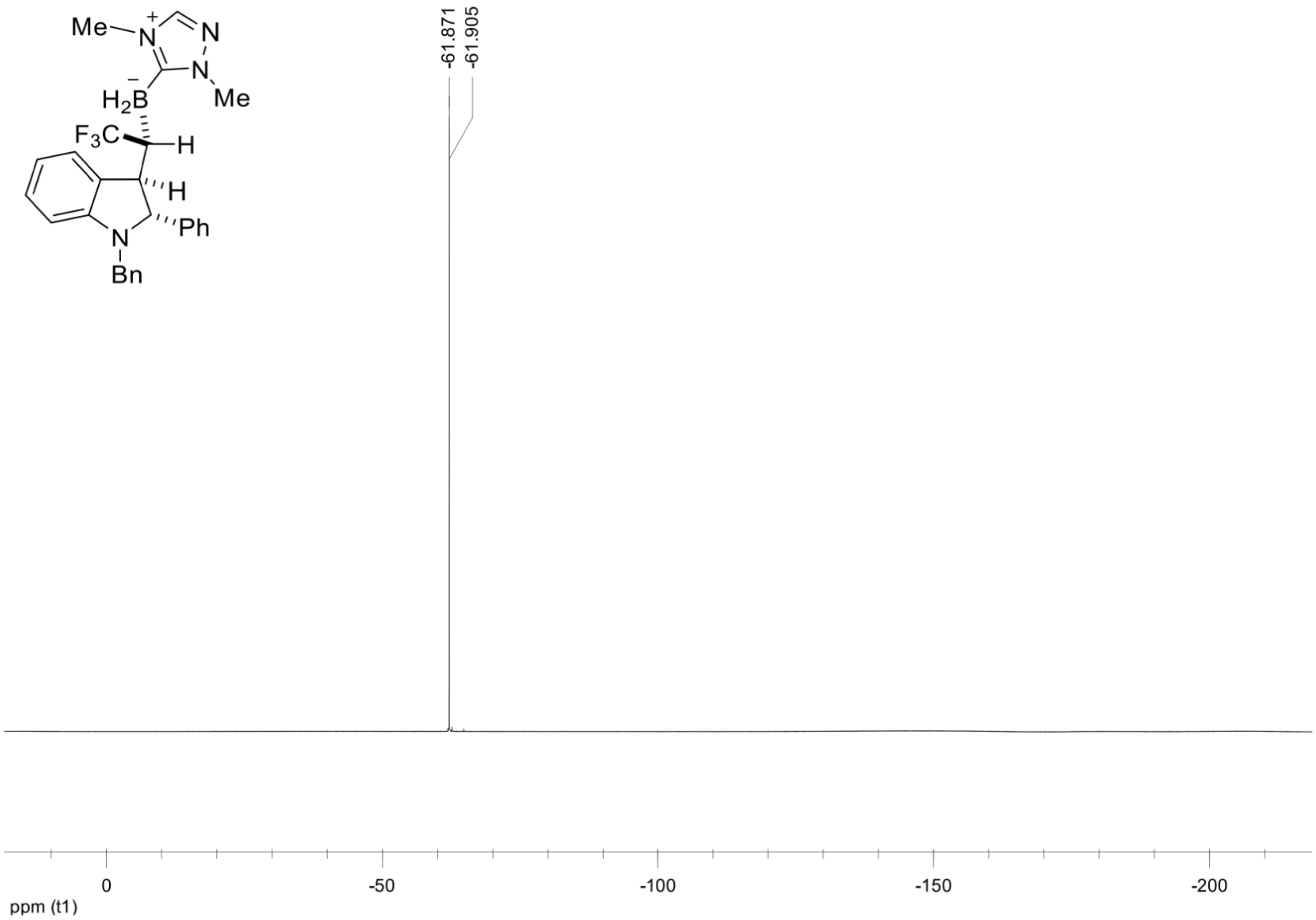

Supplementary Figure 156. ^{19}F NMR spectrum of **4sa** (376 MHz, $CDCl_3$)

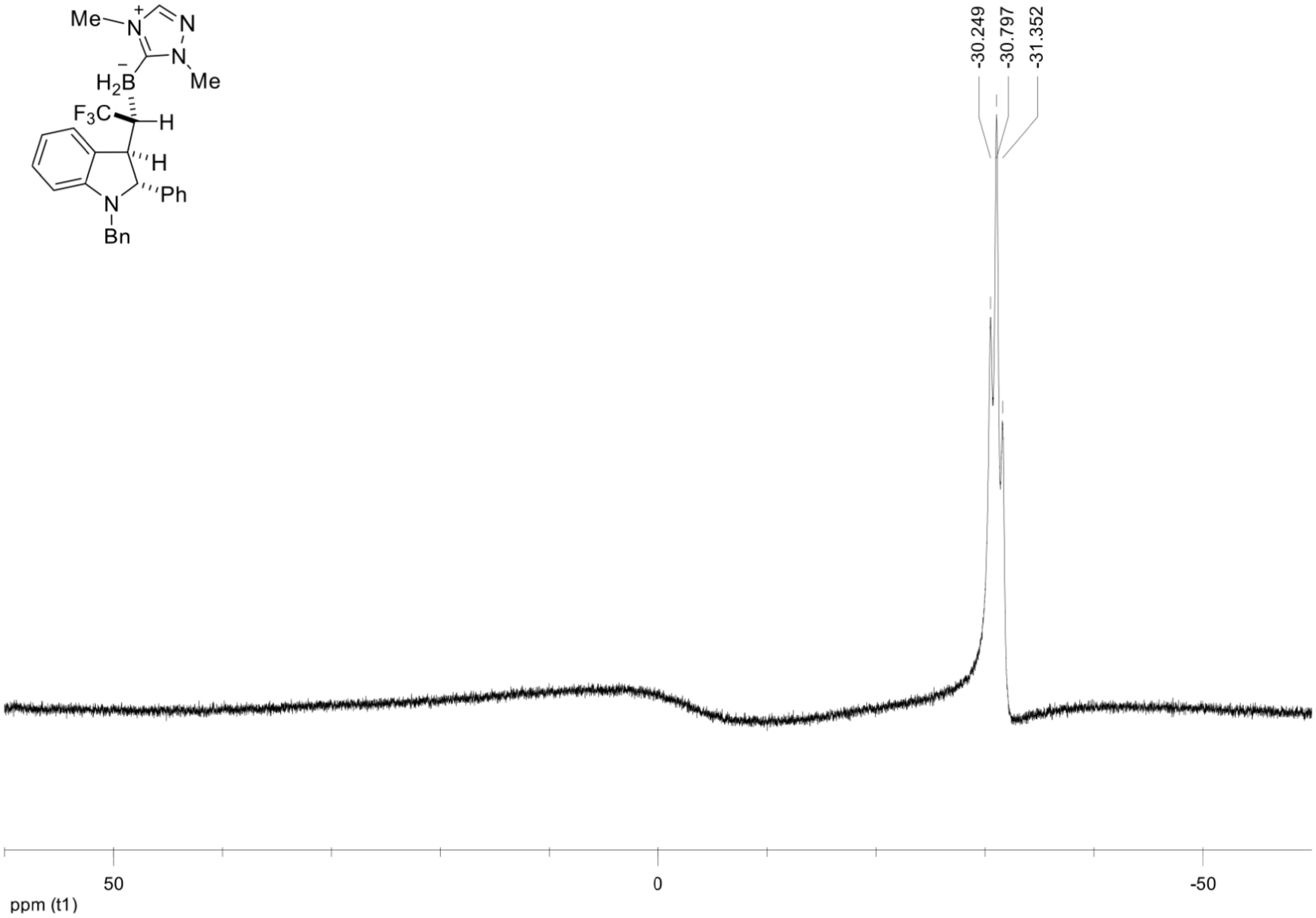

Supplementary Figure 157. ^{11}B NMR spectrum of **4sa** (160.5 MHz, $CDCl_3$)

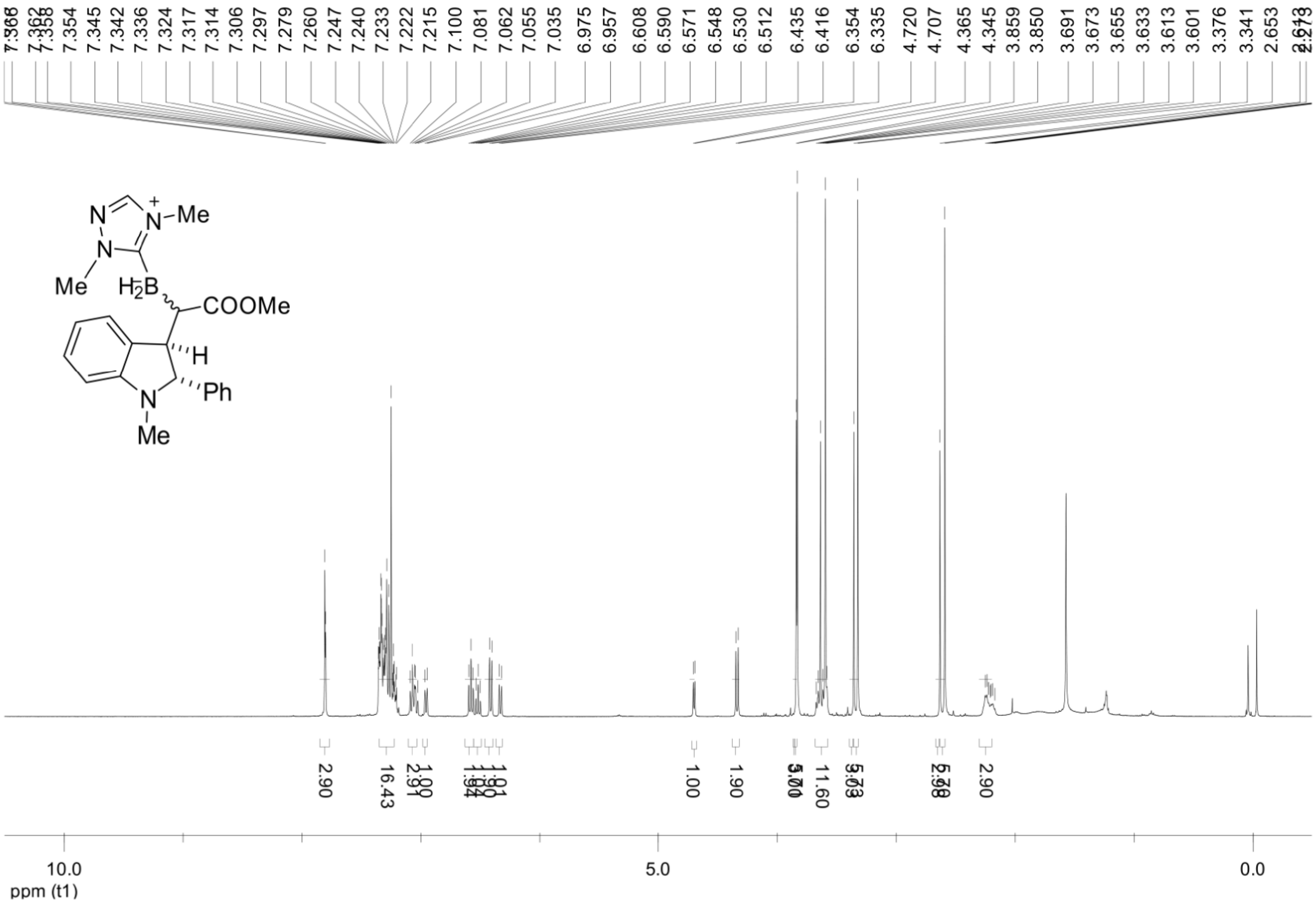

Supplementary Figure 158. ^{1}H NMR spectrum of **3ba+4ba** (400 MHz, $CDCl_3$)

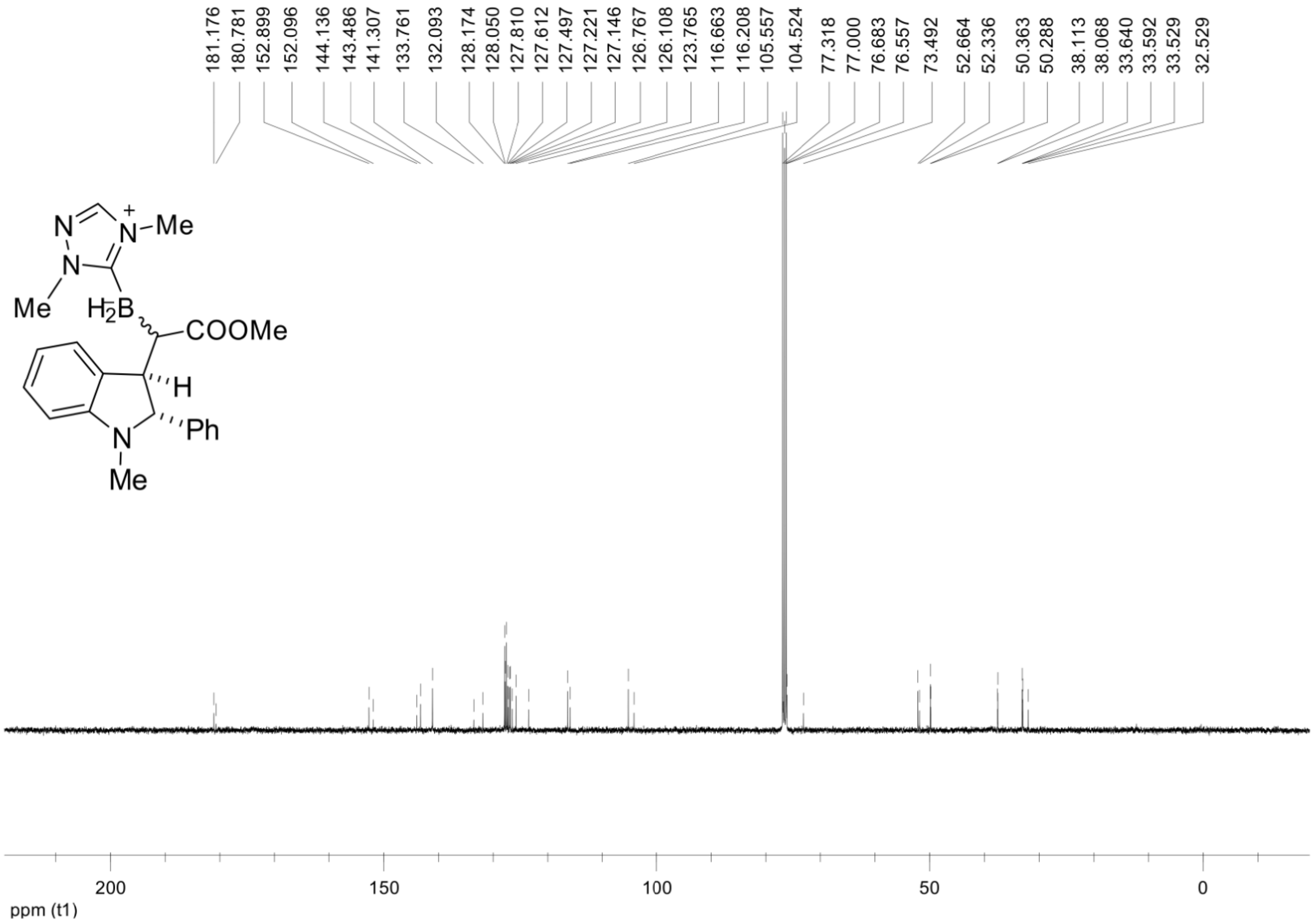

Supplementary Figure 159. ^{13}C NMR spectrum of **3ba+4ba** (100 MHz, $CDCl_3$)

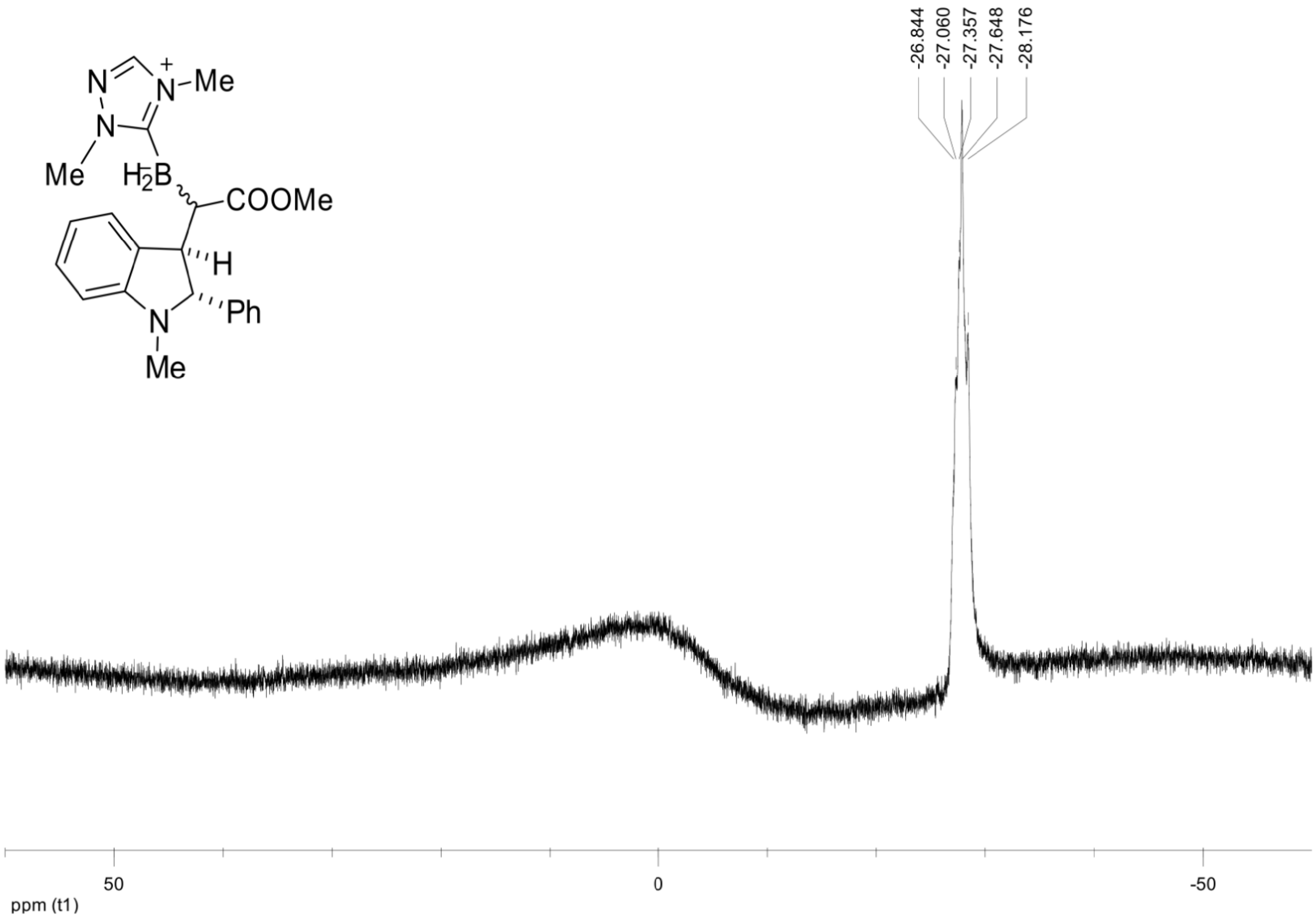

Supplementary Figure 160. ^{11}B NMR spectrum of **3ba+4ba** (128.4 MHz, $CDCl_3$)

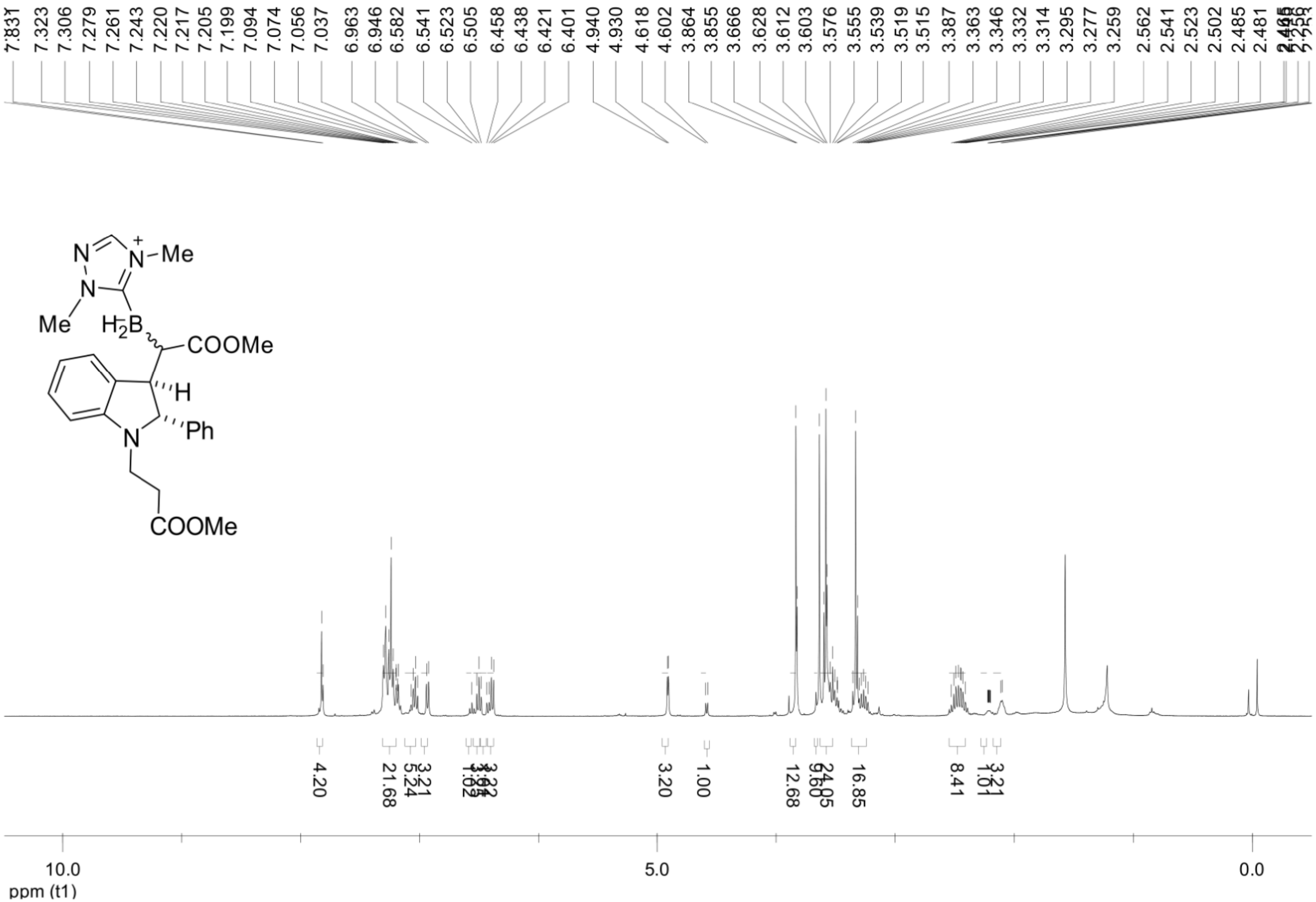

Supplementary Figure 161. ^{1}H NMR spectrum of **3la+4la** (400 MHz, $CDCl_3$)

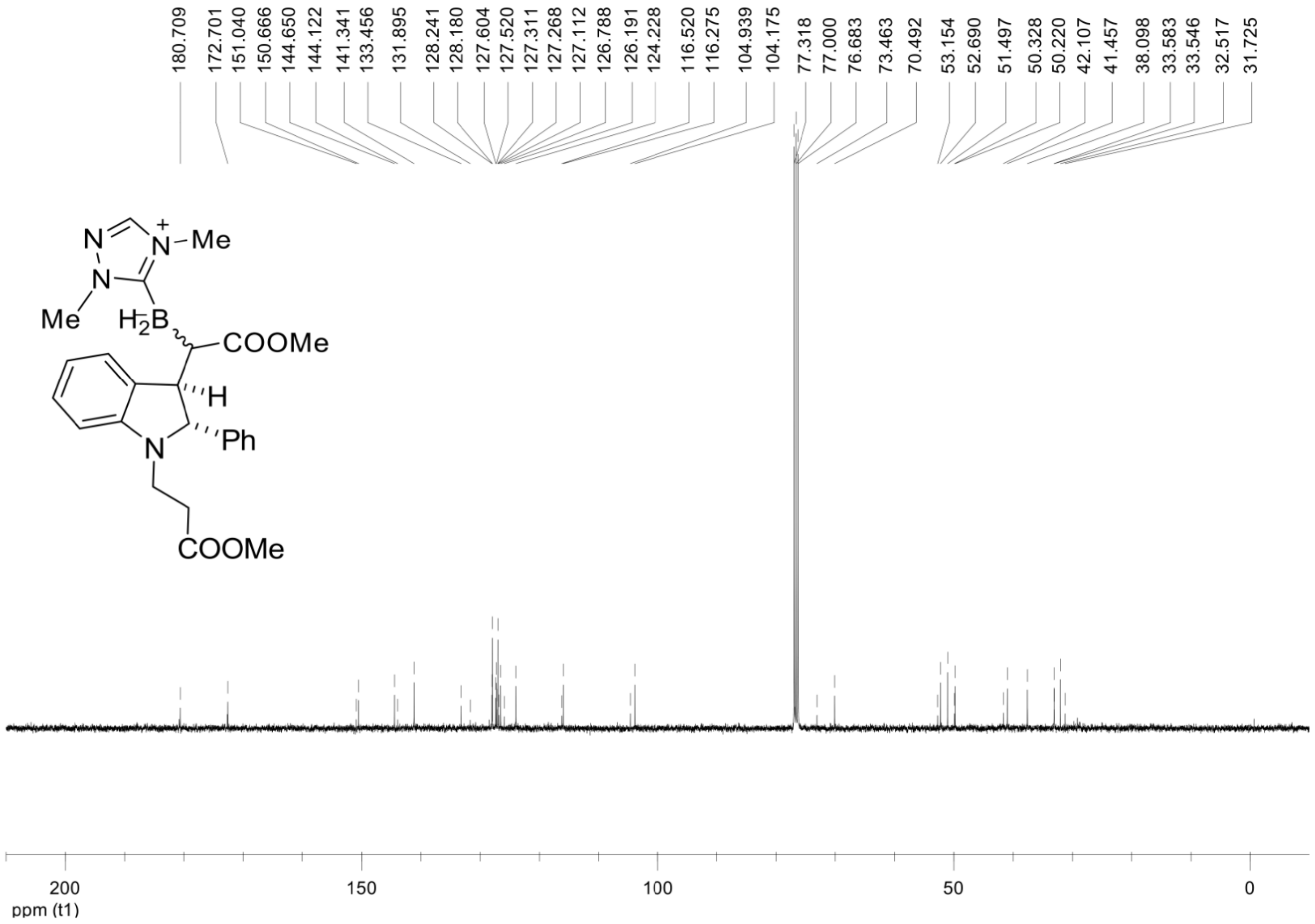

Supplementary Figure 162. ^{13}C NMR spectrum of **3la+4la** (100 MHz, $CDCl_3$)

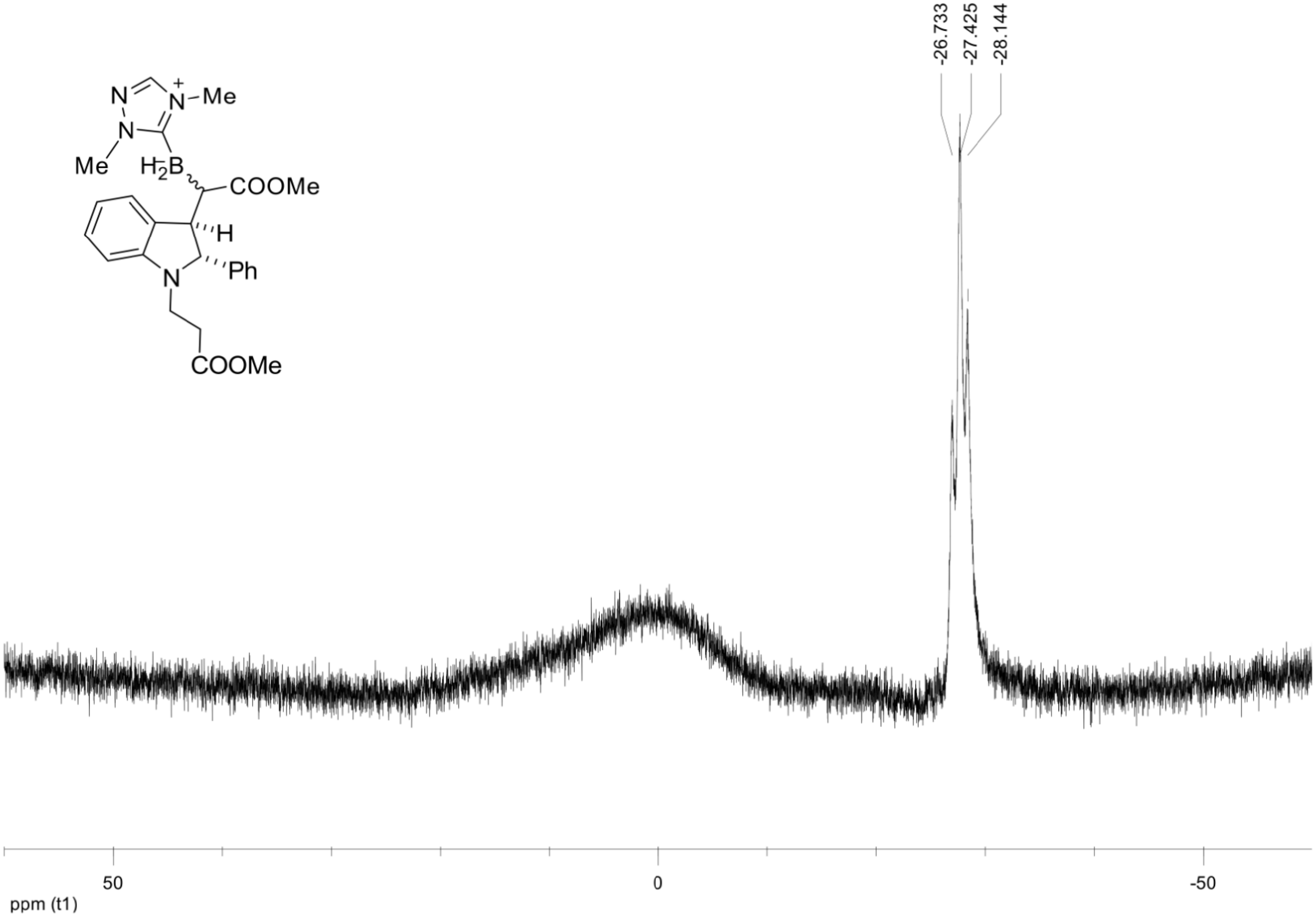

Supplementary Figure 163. ^{11}B NMR spectrum of **3la+4la** (128.4 MHz, $CDCl_3$)

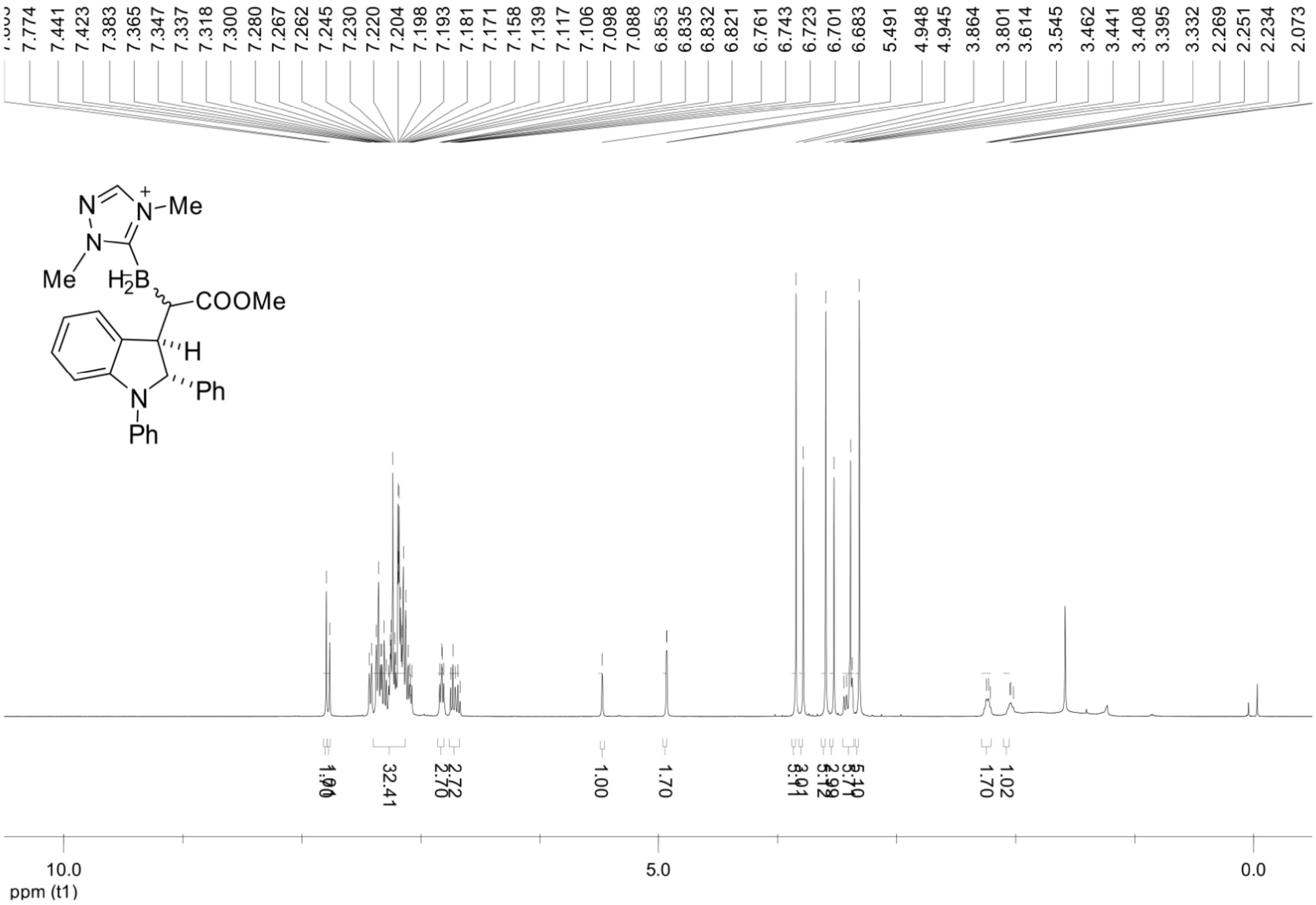

Supplementary Figure 164. ^{1}H NMR spectrum of **3na+4na** (400 MHz, $CDCl_3$)

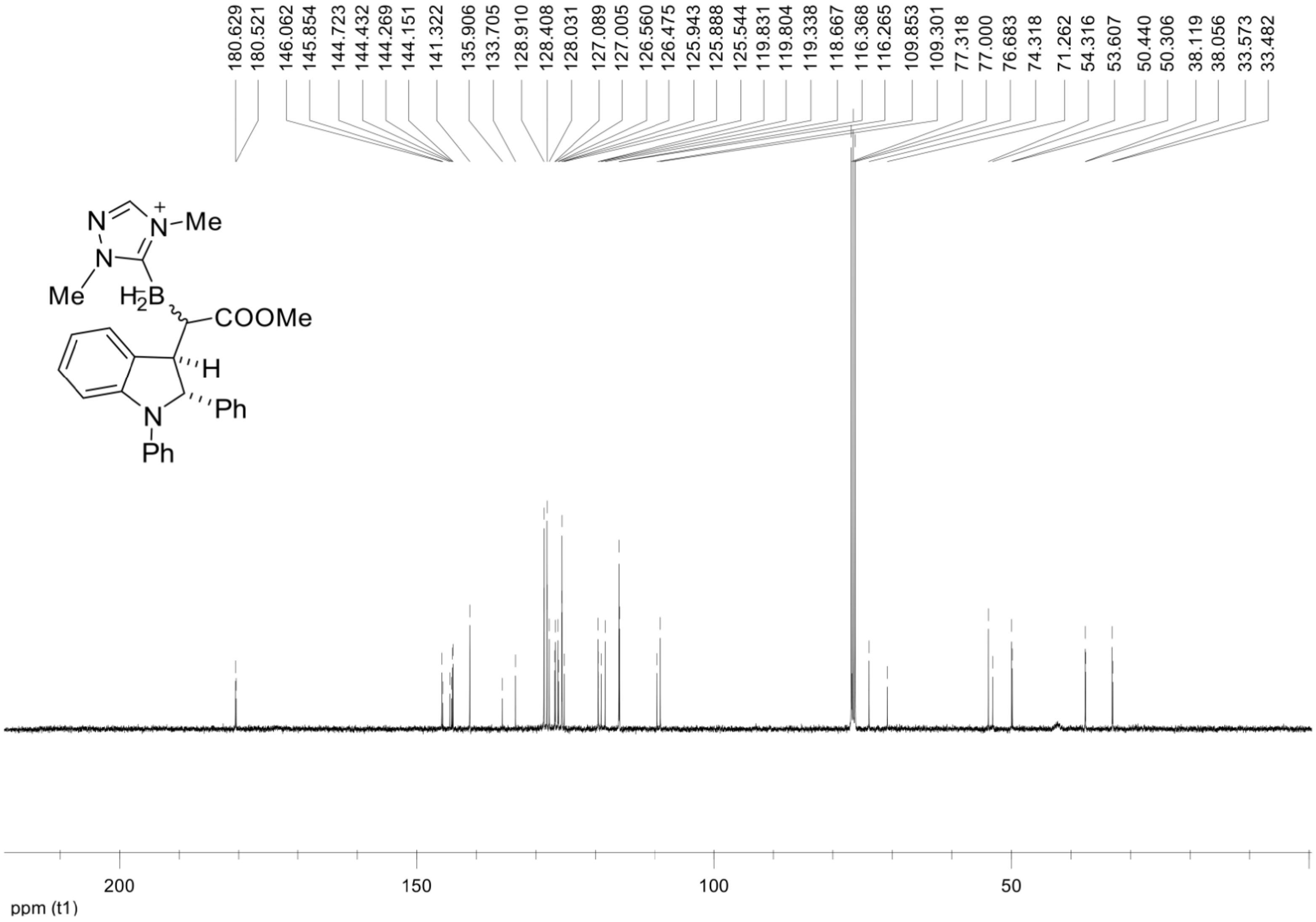

Supplementary Figure 165. ^{13}C NMR spectrum of **3na+4na** (100 MHz, $CDCl_3$)

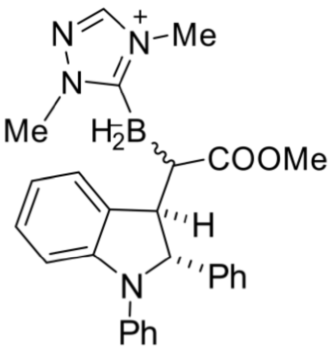

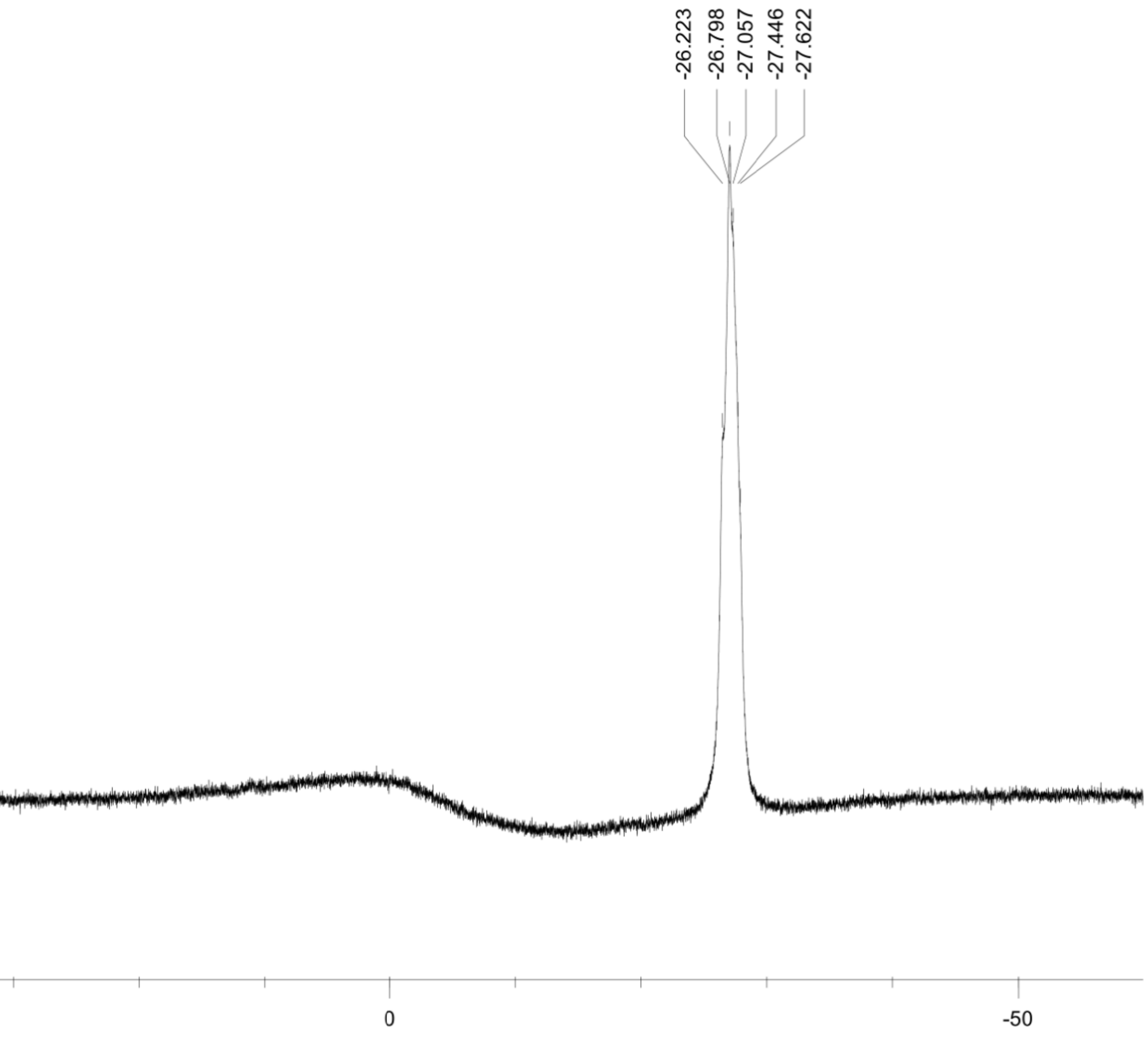

Supplementary Figure 166. ^{11}B NMR spectrum of **3na+4na** (128.4 MHz, $CDCl_3$)

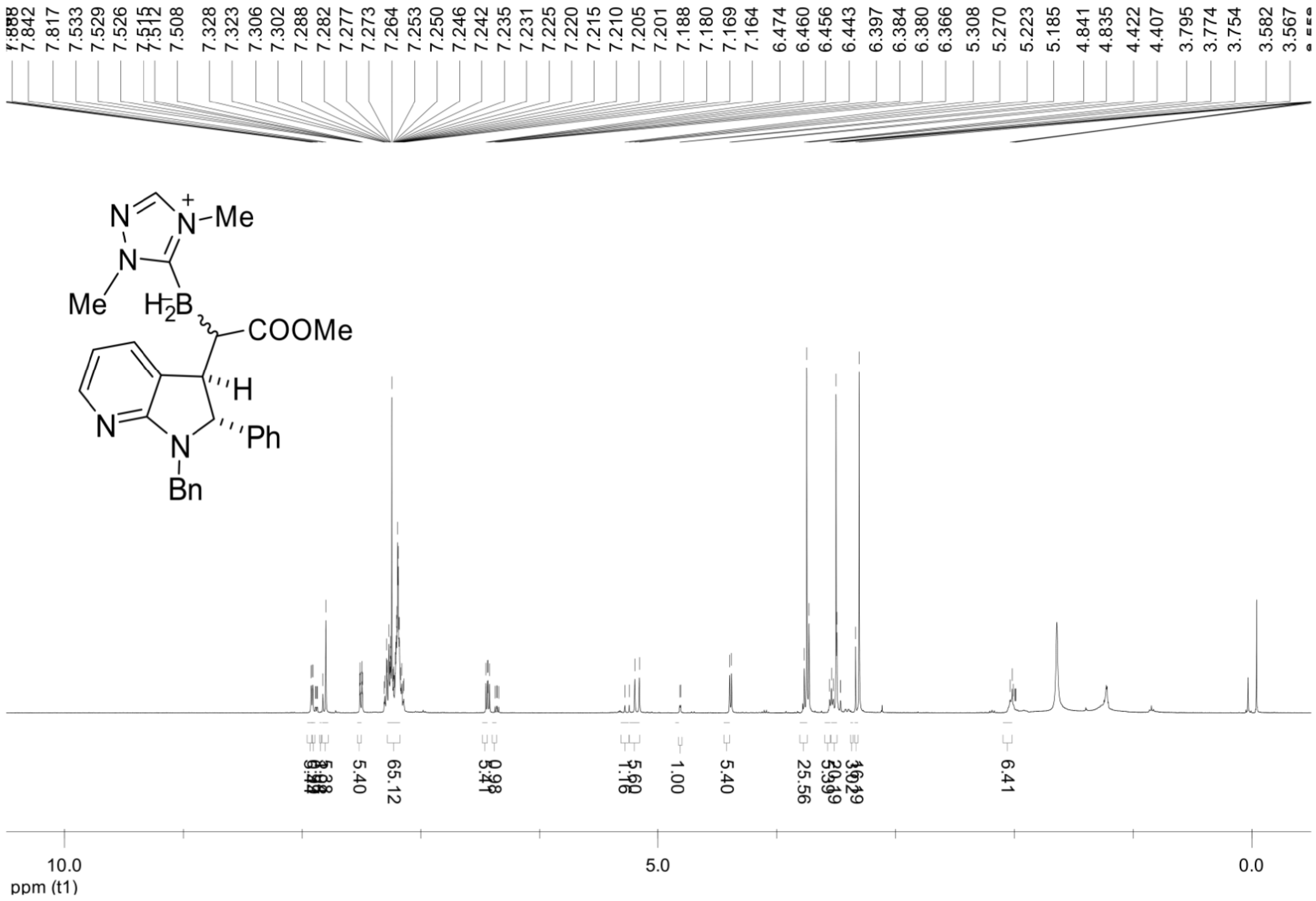

Supplementary Figure 167. ^{1}H NMR spectrum of **3ja+4ja** (400 MHz, $CDCl_3$)

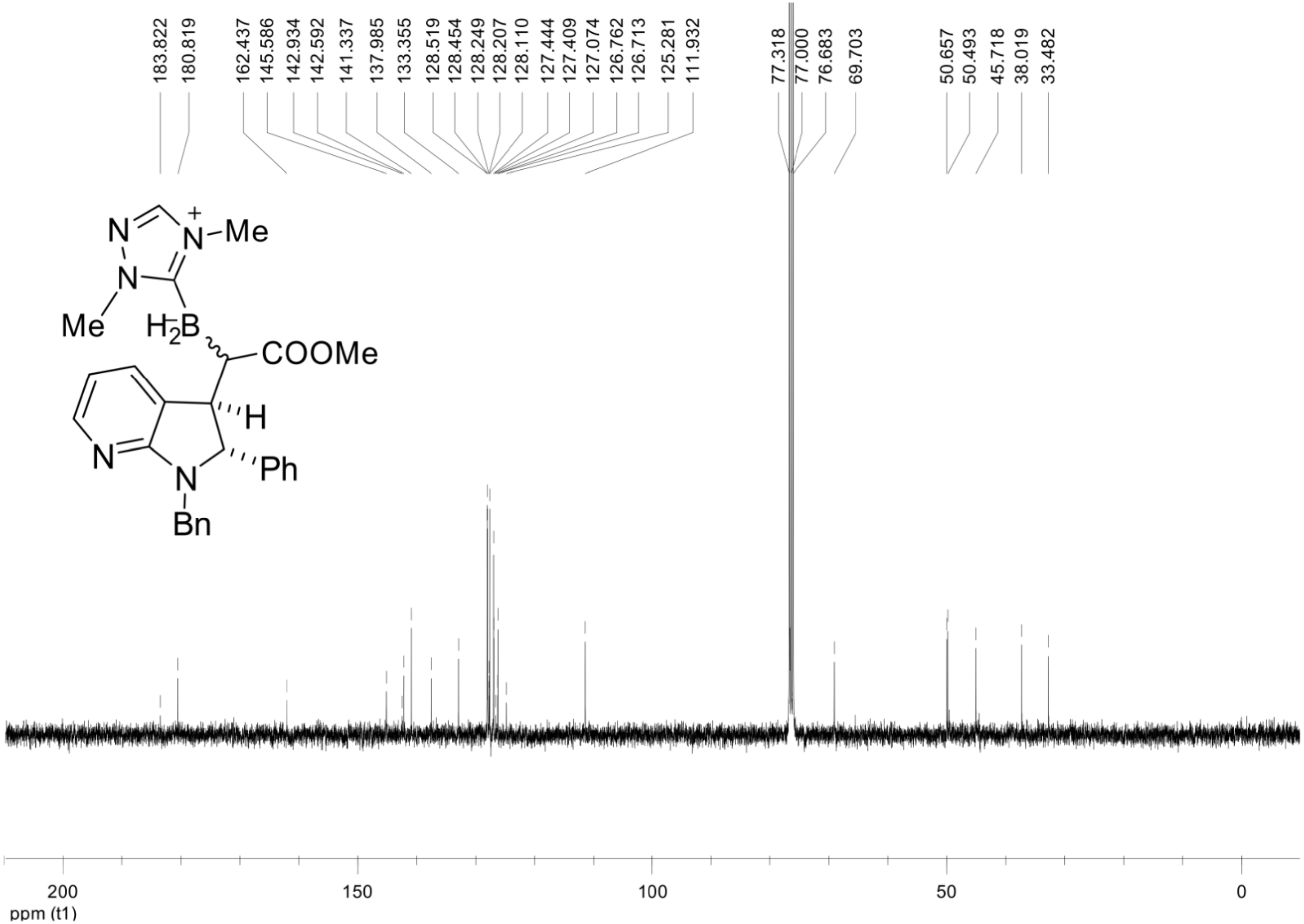

Supplementary Figure 168. ^{13}C NMR spectrum of **3ja+4ja** (100 MHz, $CDCl_3$)

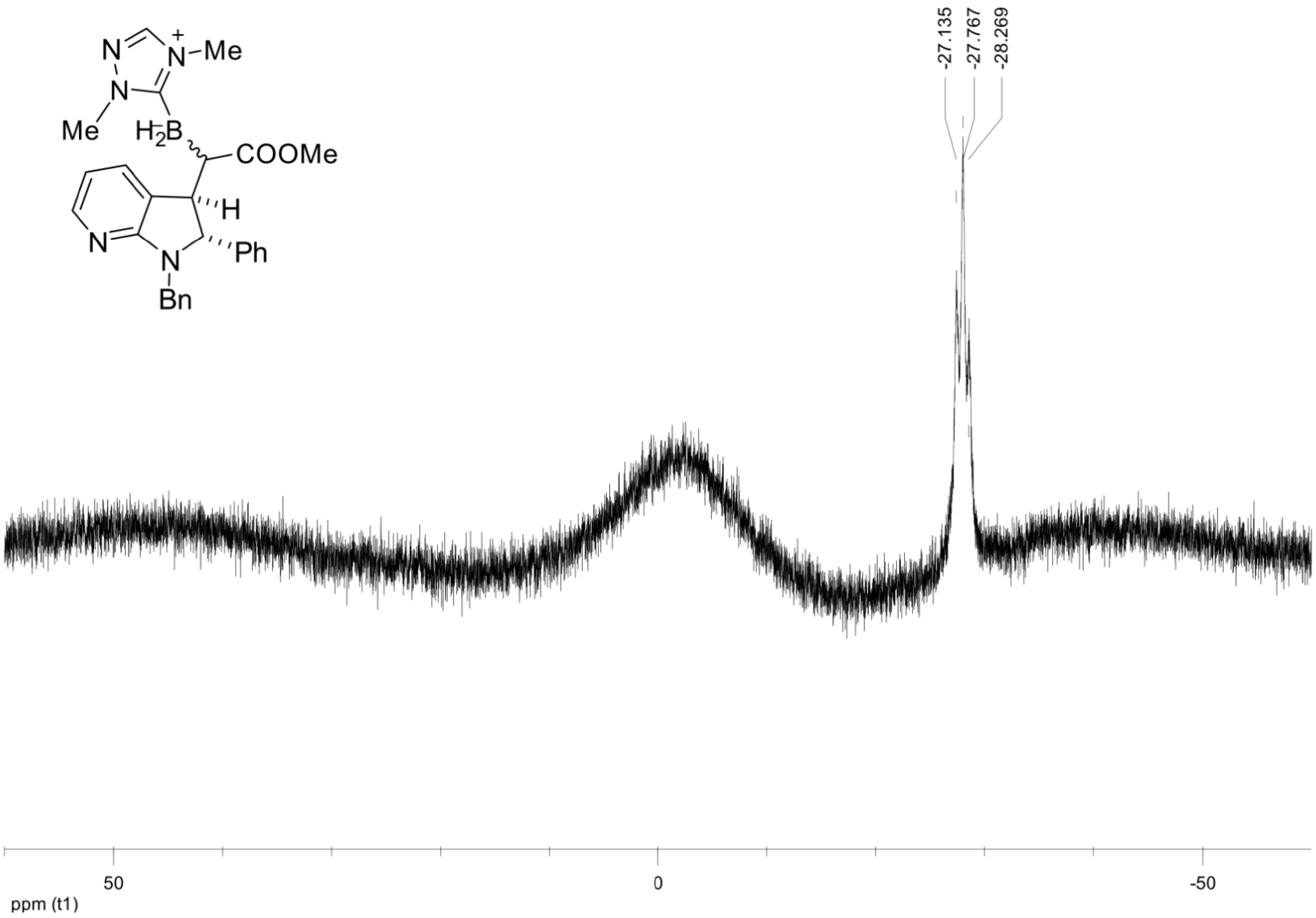

Supplementary Figure 169. ^{11}B NMR spectrum of **3ja+4ja** (128.4 MHz, $CDCl_3$)

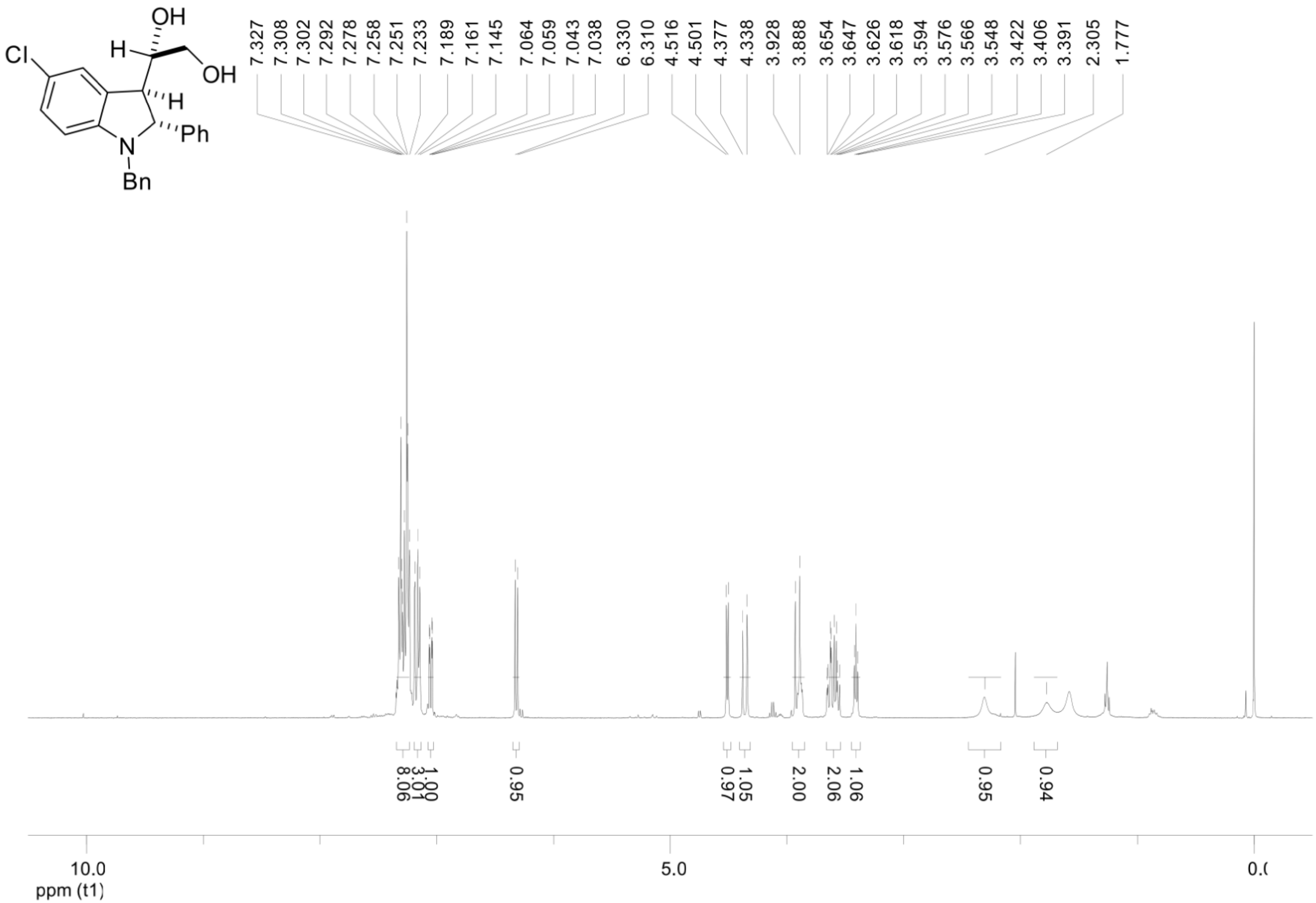

Supplementary Figure 170. ^{1}H NMR spectrum of **5a** (400 MHz, $CDCl_3$)

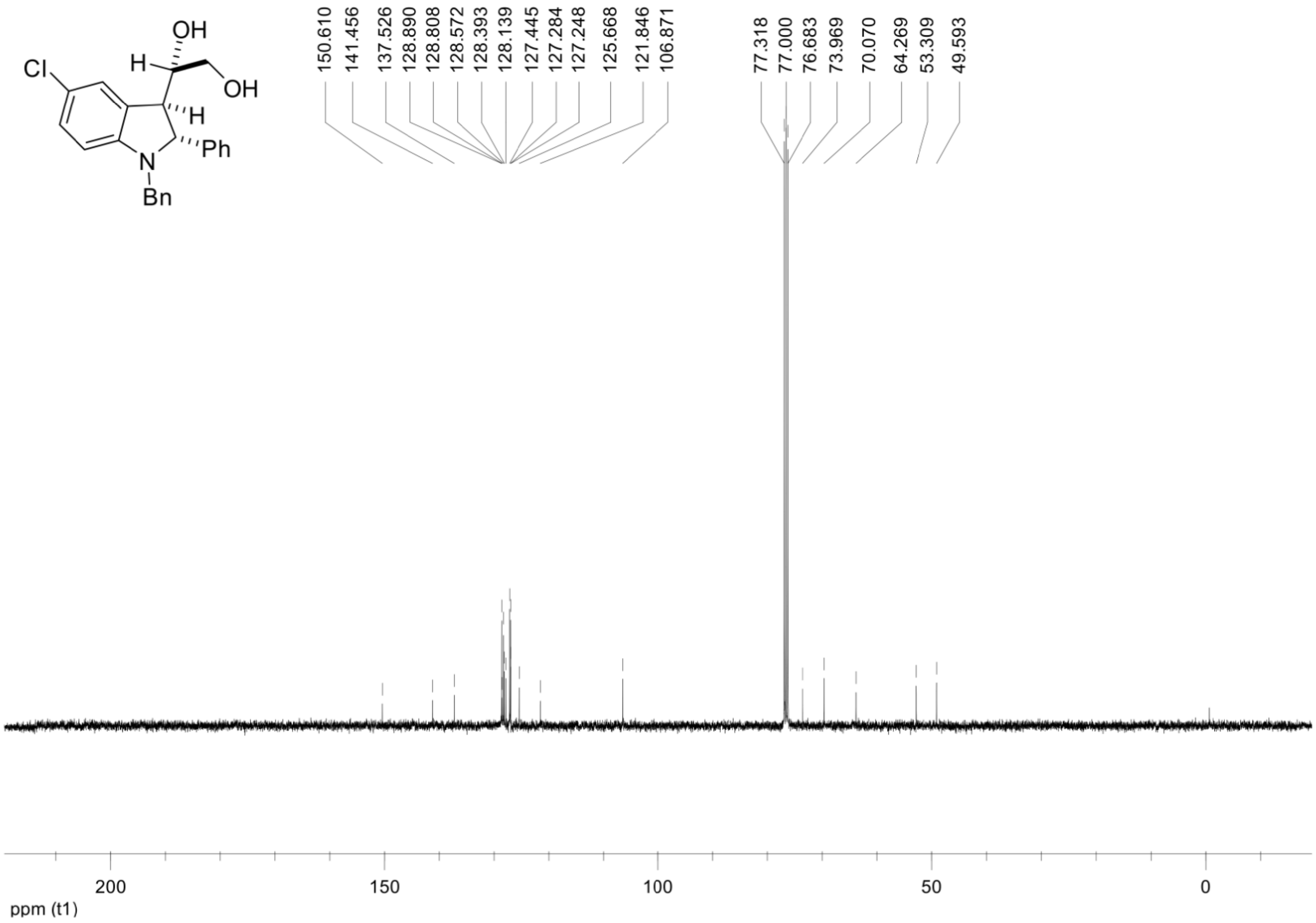

Supplementary Figure 171. ^{13}C NMR spectrum of **5a** (100 MHz, $CDCl_3$)

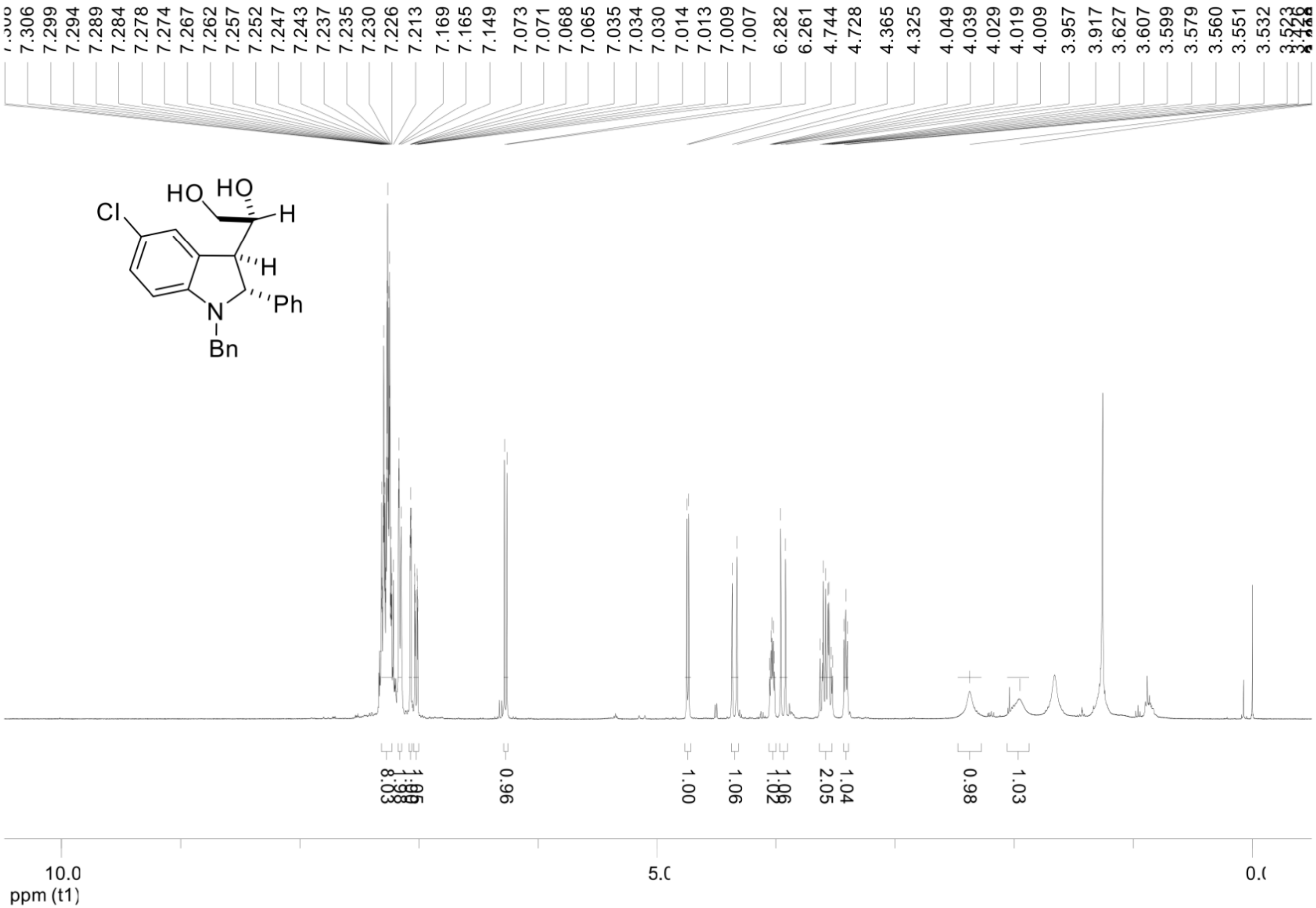

Supplementary Figure 172. ^{1}H NMR spectrum of **5aa** (400 MHz, $CDCl_3$)

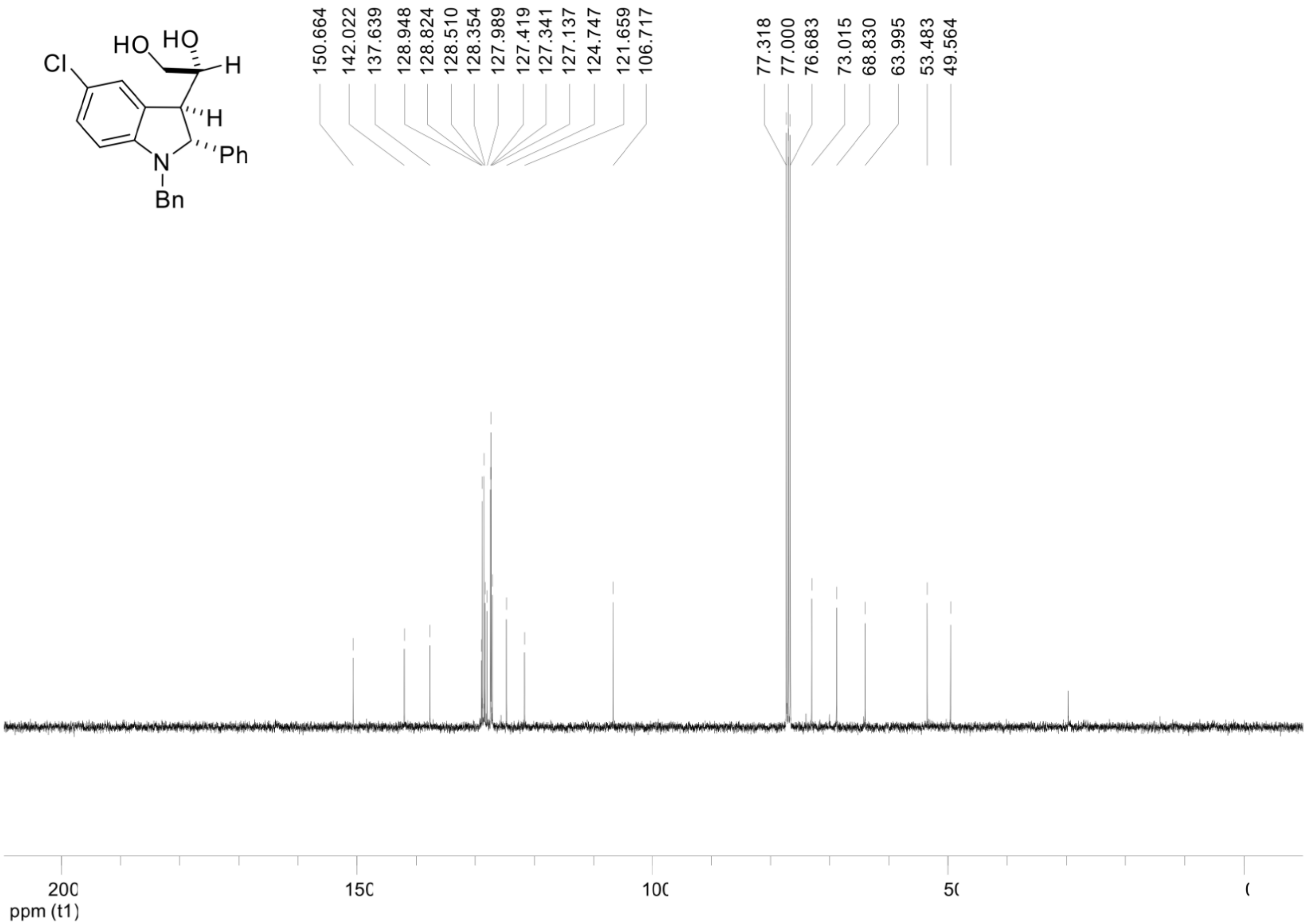

Supplementary Figure 173. ^{13}C NMR spectrum of **5aa** (100 MHz, $CDCl_3$)

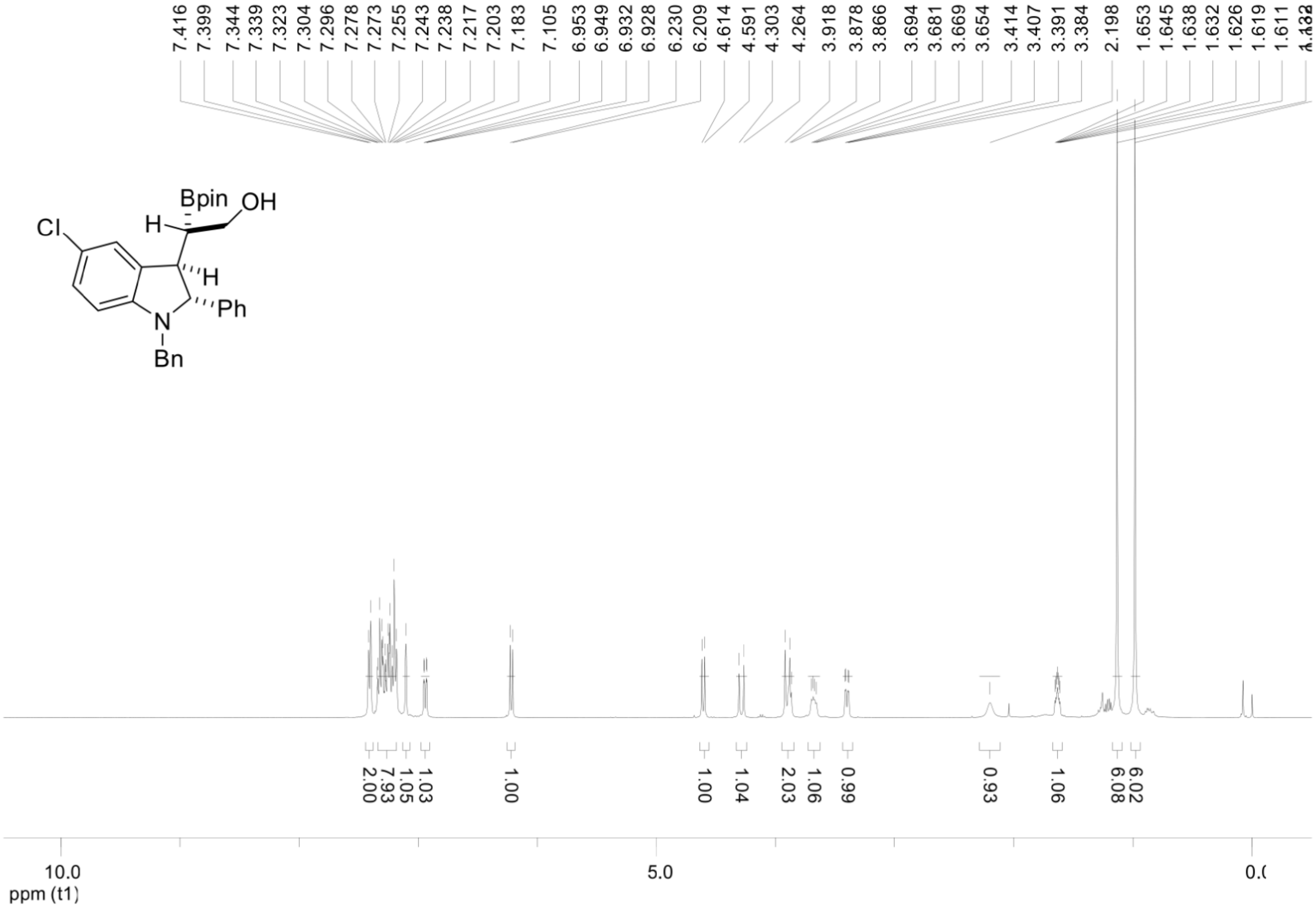

Supplementary Figure 174. ^{1}H NMR spectrum of **6a** (400 MHz, $CDCl_3$)

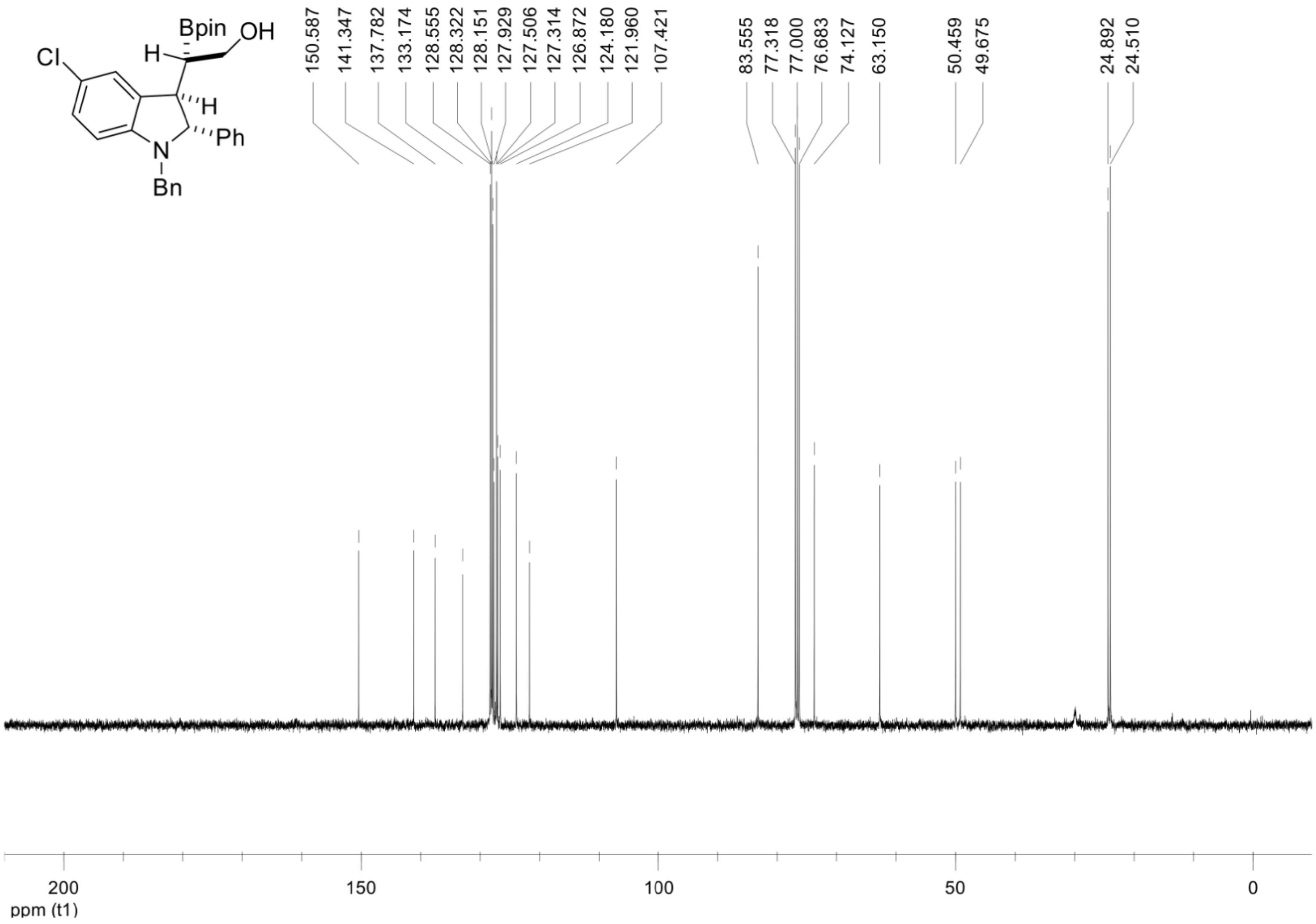

Supplementary Figure 175. ^{13}C NMR spectrum of **6a** (100 MHz, $CDCl_3$)

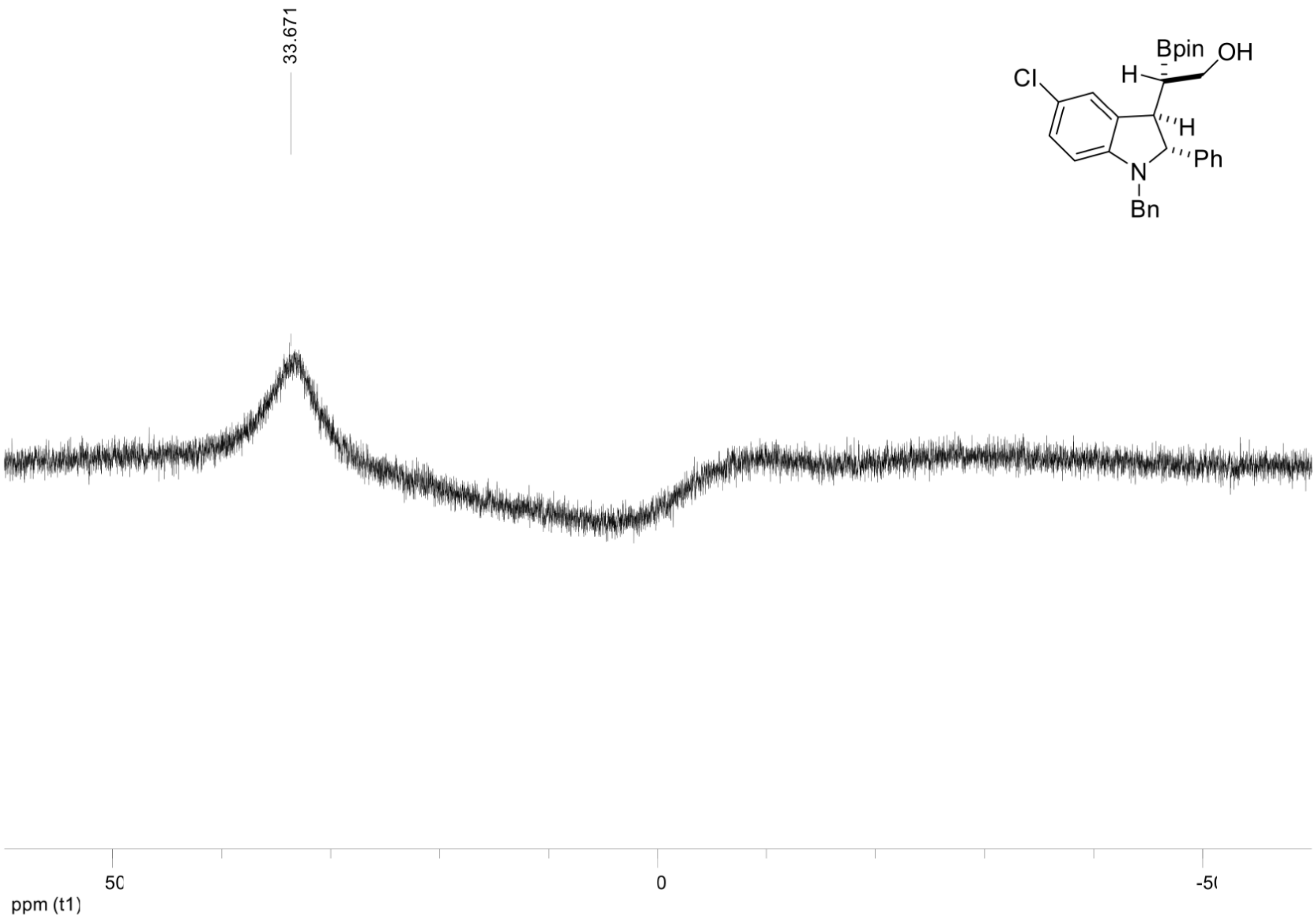

Supplementary Figure 176. ^{11}B NMR spectrum of **6a** (128.4 MHz, $CDCl_3$)

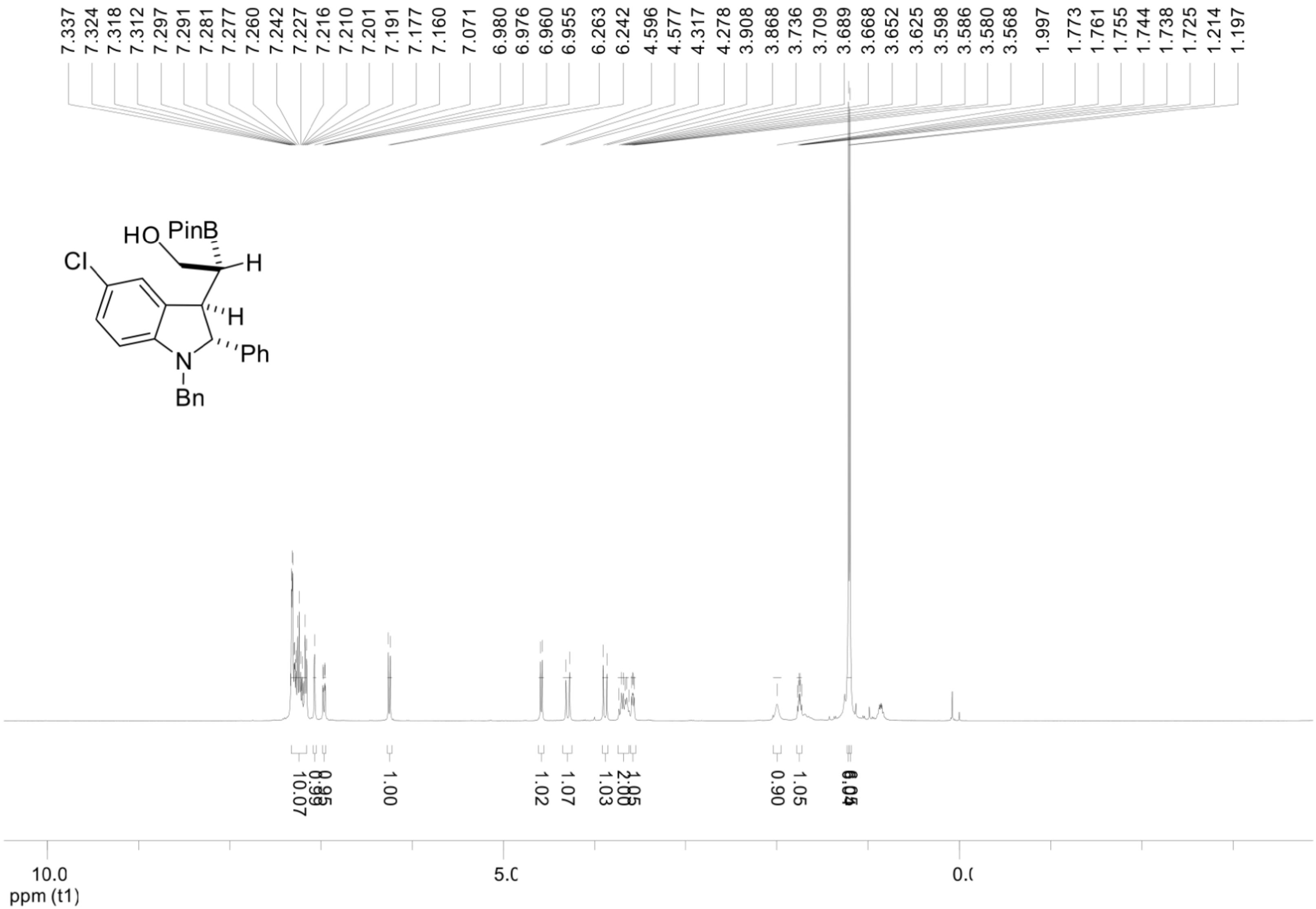

Supplementary Figure 177. ^{1}H NMR spectrum of **6aa** (400 MHz, $CDCl_3$)

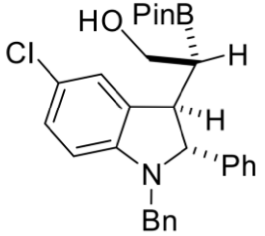

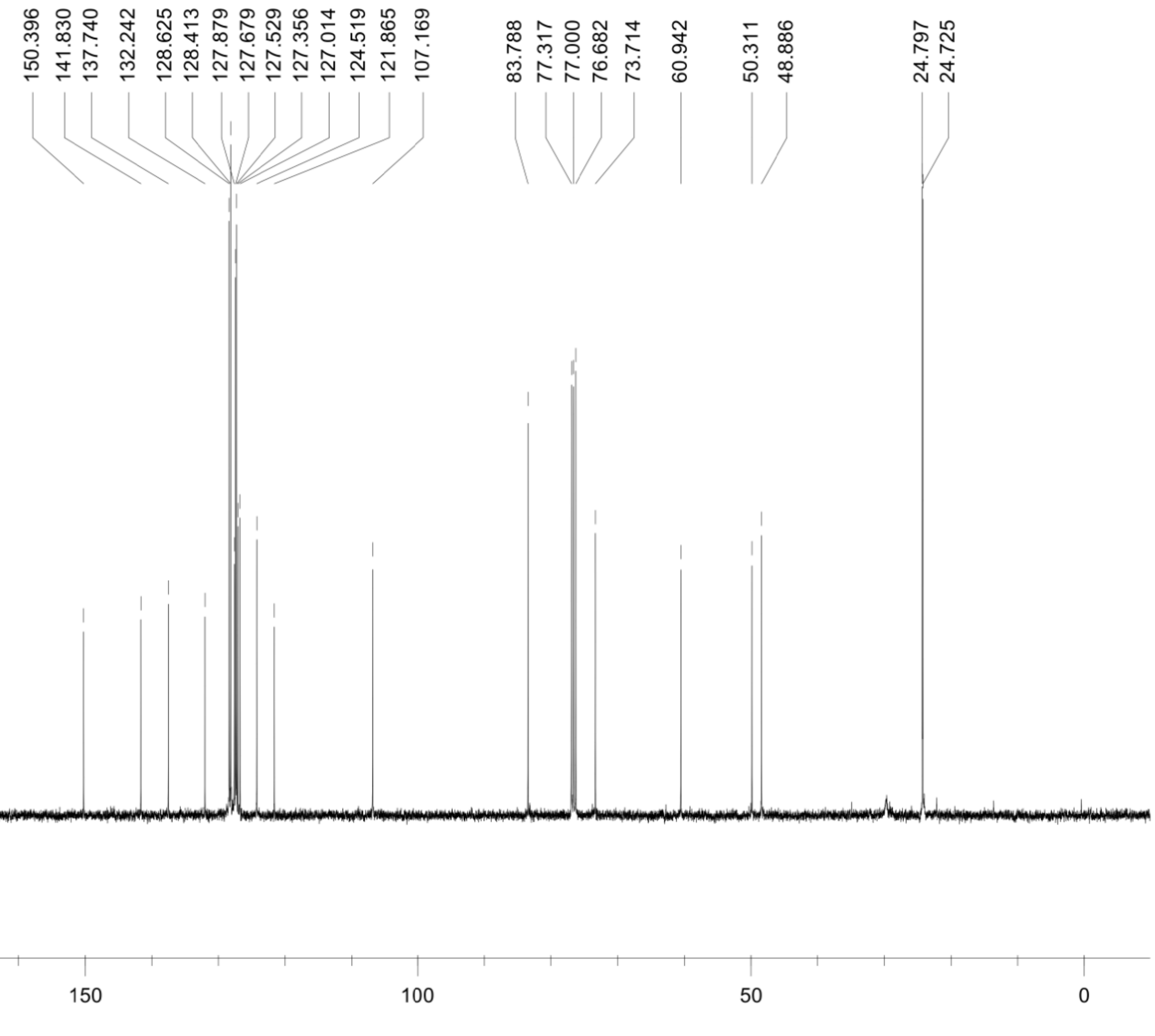

Supplementary Figure 178. ^{13}C NMR spectrum of **6aa** (100 MHz, $CDCl_3$)

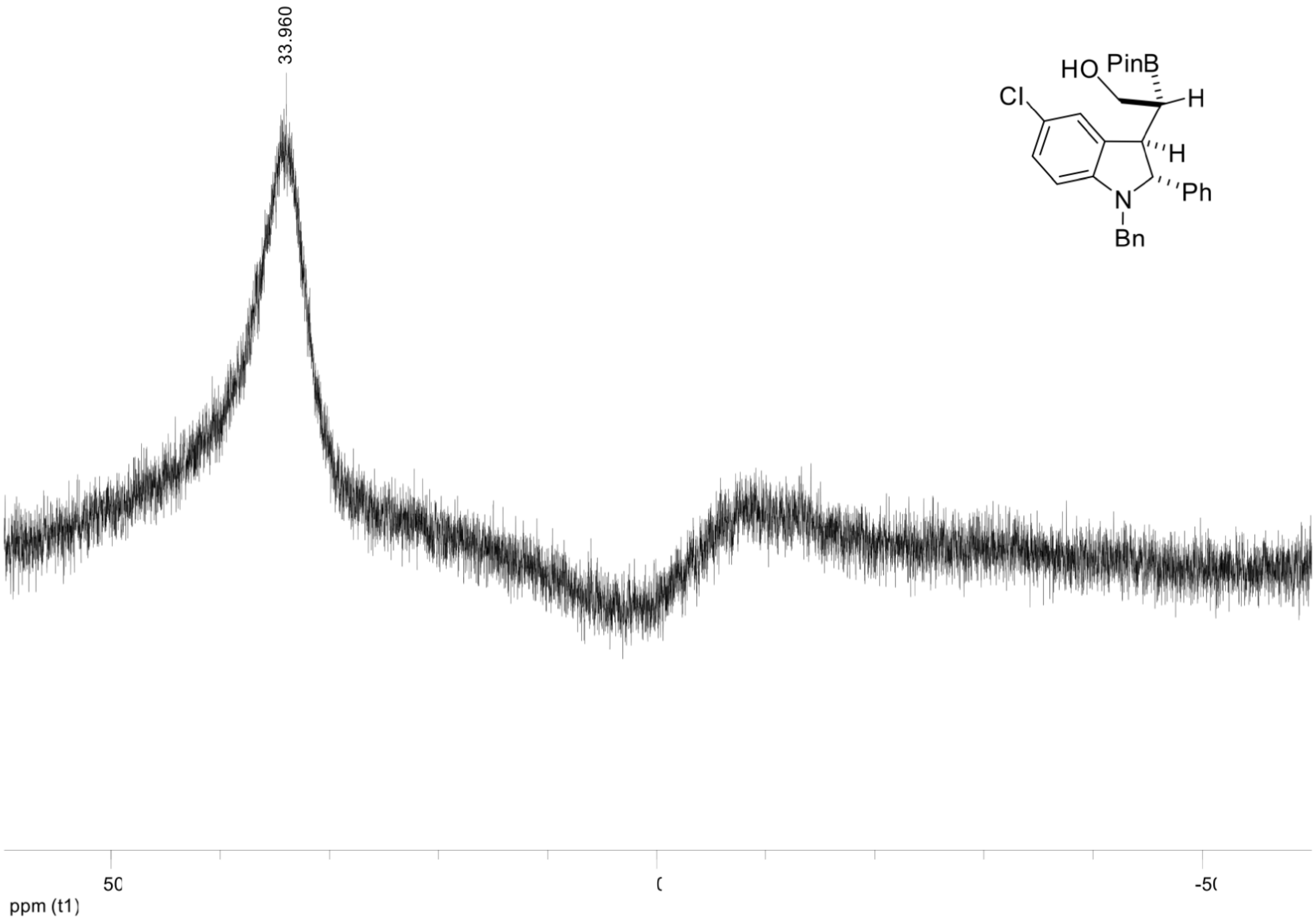

Supplementary Figure 179. ^{11}B NMR spectrum of **6aa** (128.4 MHz, $CDCl_3$)

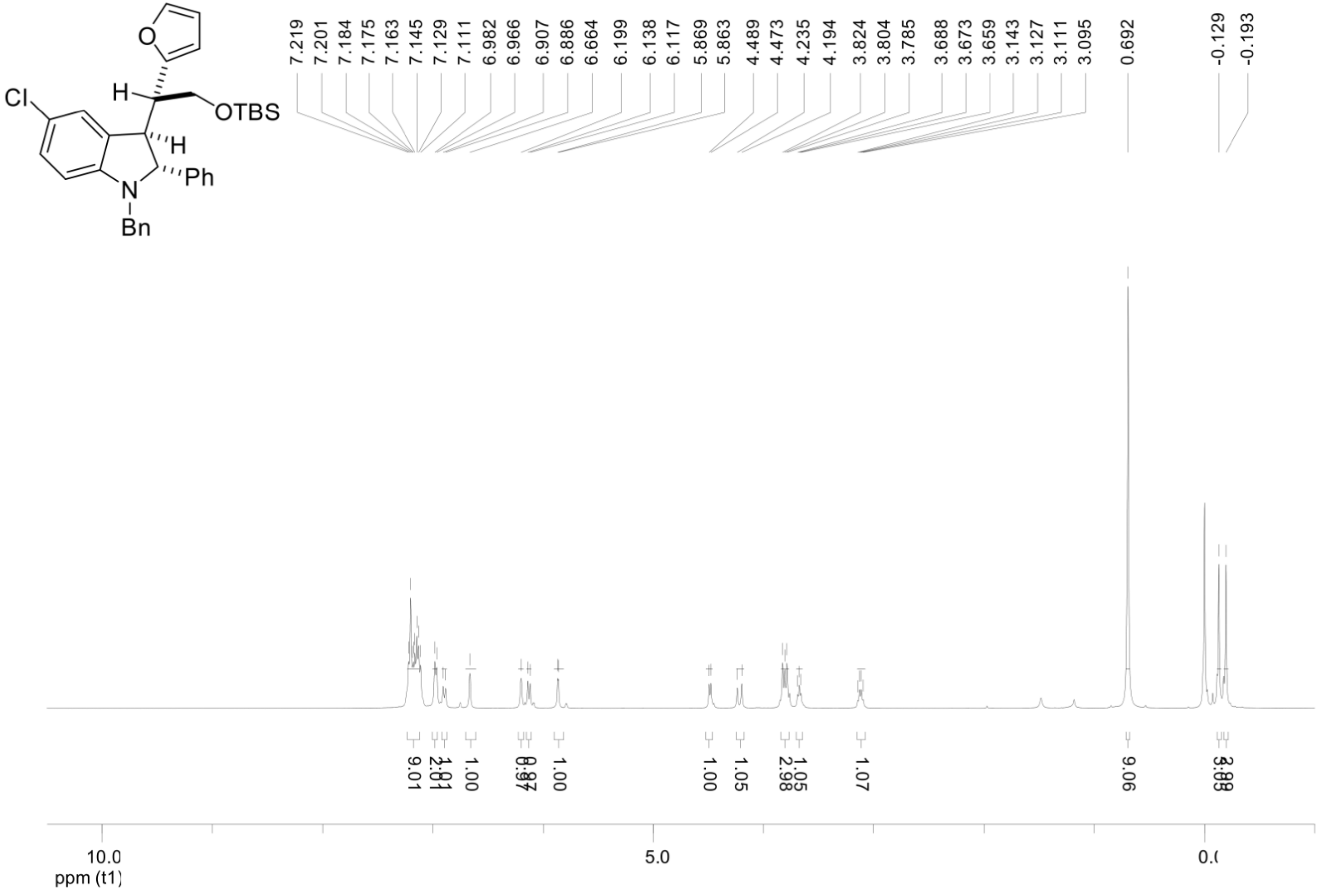

Supplementary Figure 180. ^{1}H NMR spectrum of **7a** (400 MHz, $CDCl_3$)

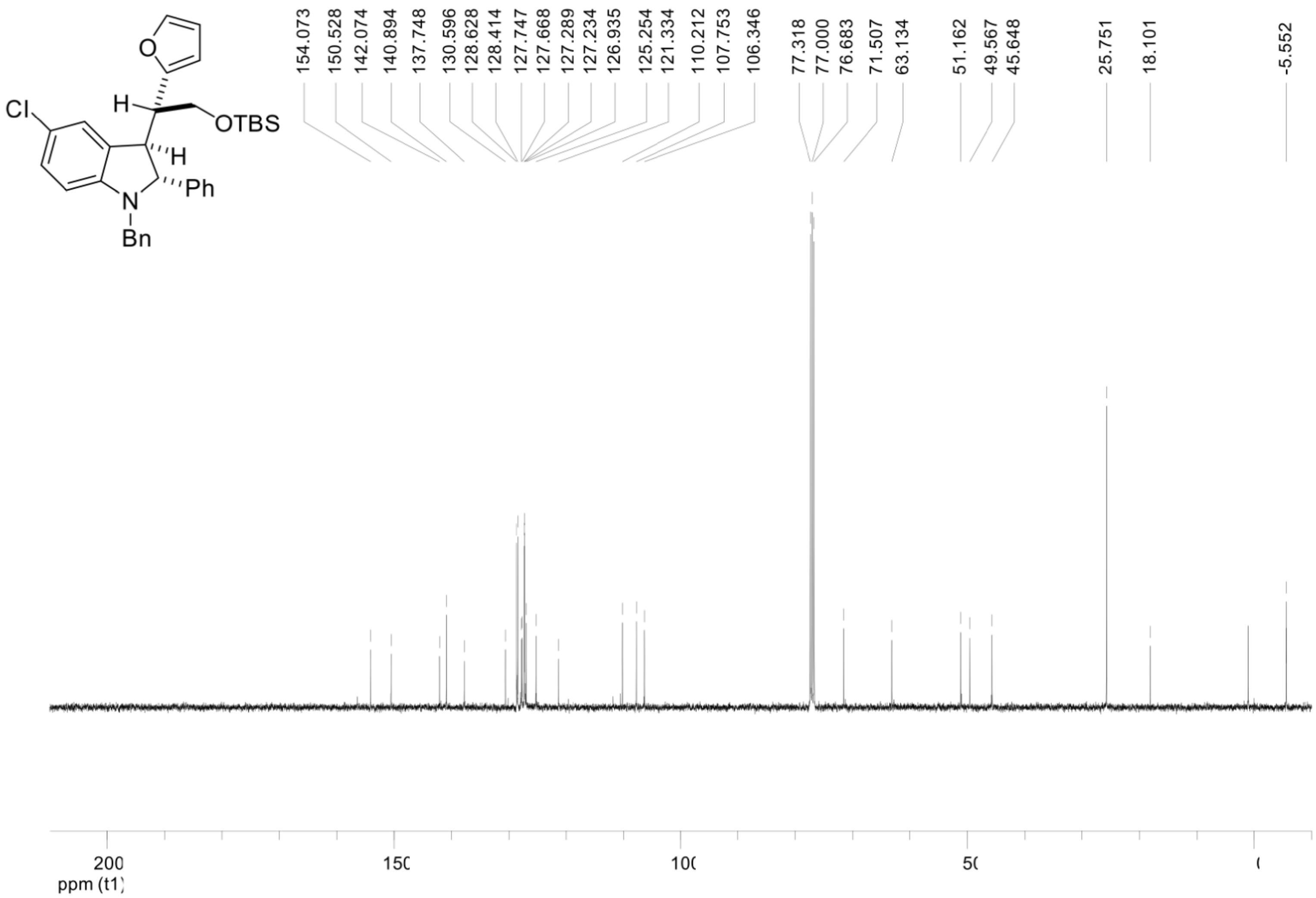

Supplementary Figure 181. ^{13}C NMR spectrum of **7a** (100 MHz, $CDCl_3$)

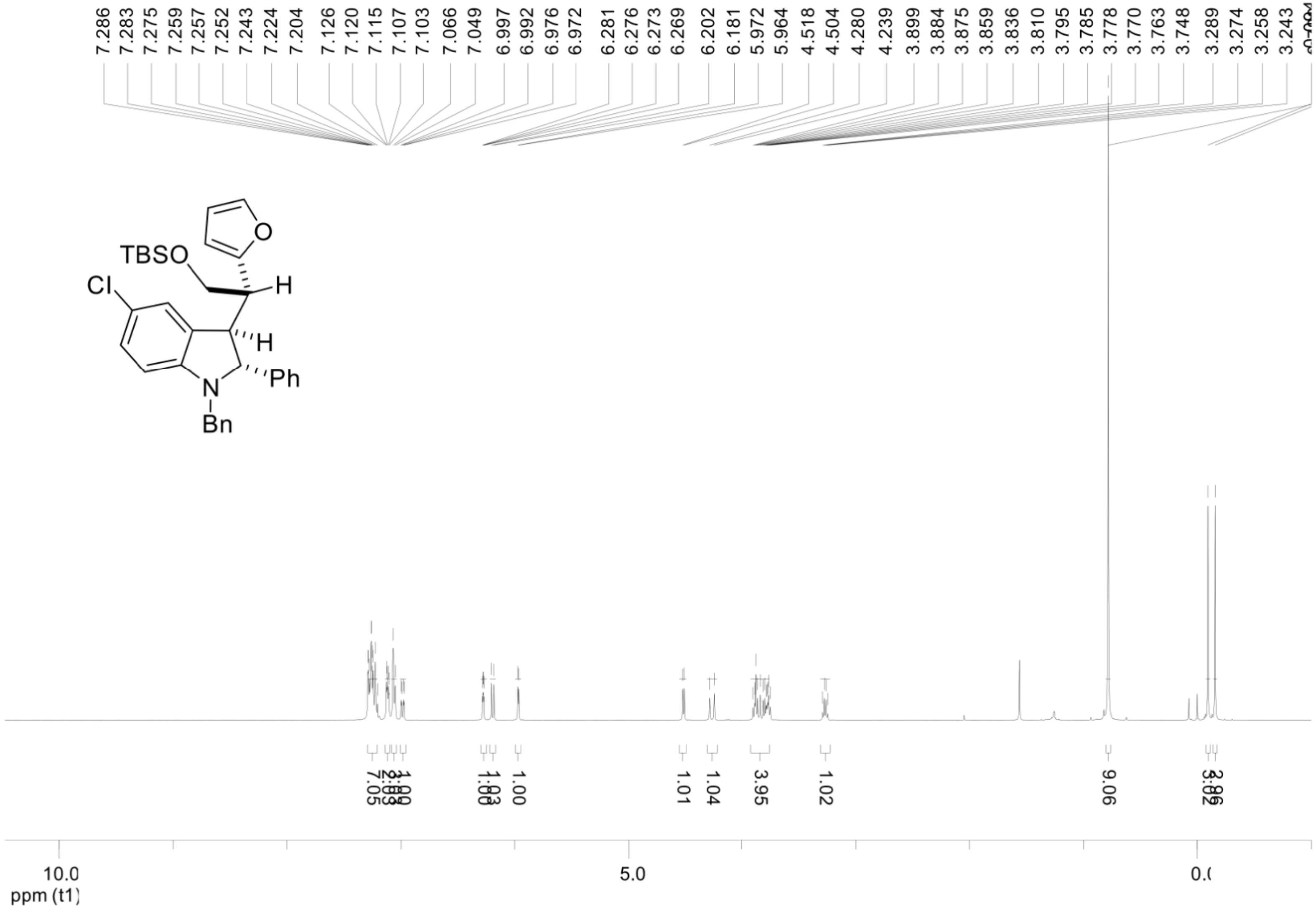

Supplementary Figure 182. ^{1}H NMR spectrum of **7aa** (400 MHz, $CDCl_3$)

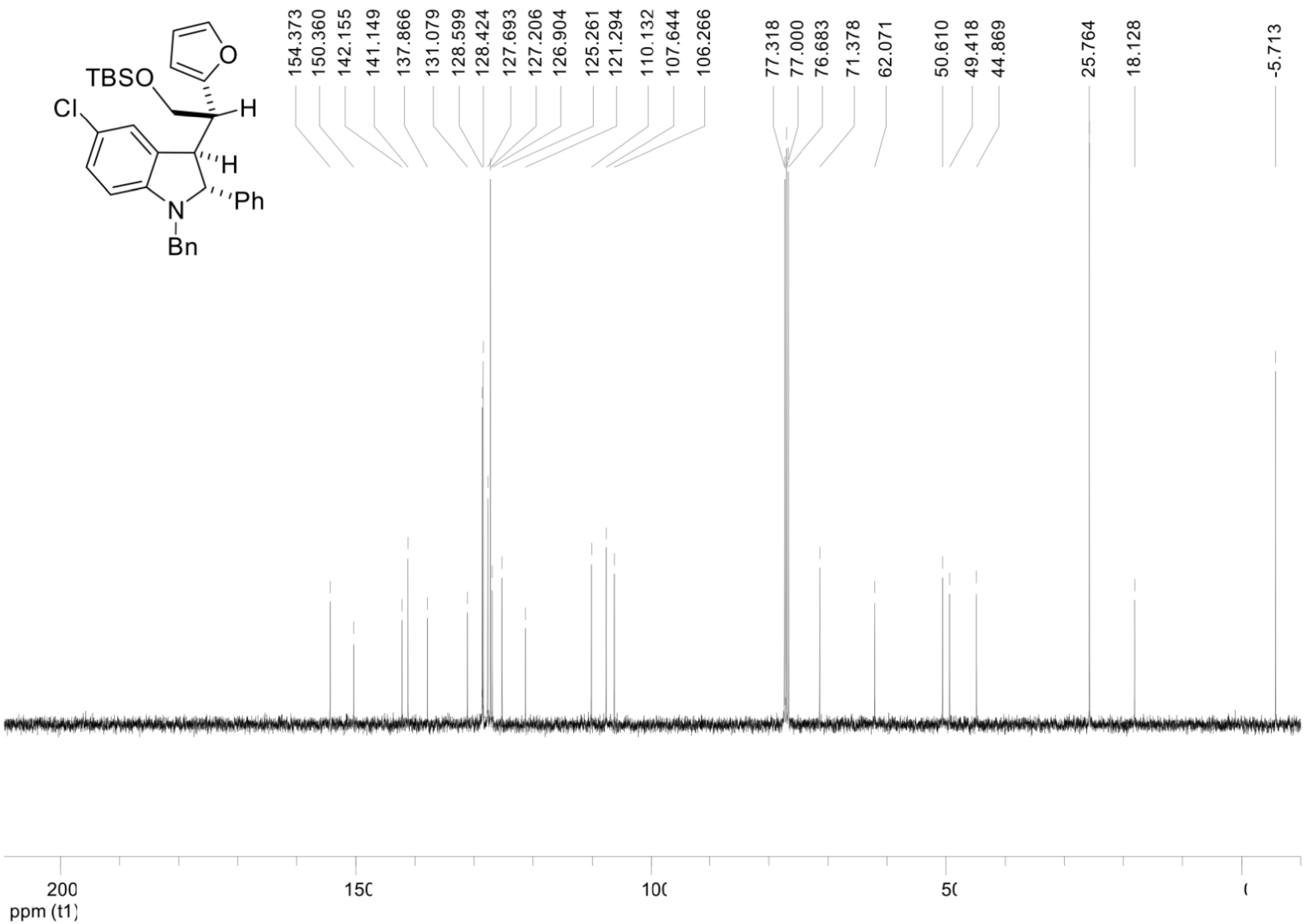

Supplementary Figure 183. ^{13}C NMR spectrum of **7aa** (100 MHz, $CDCl_3$)

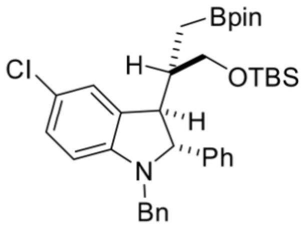

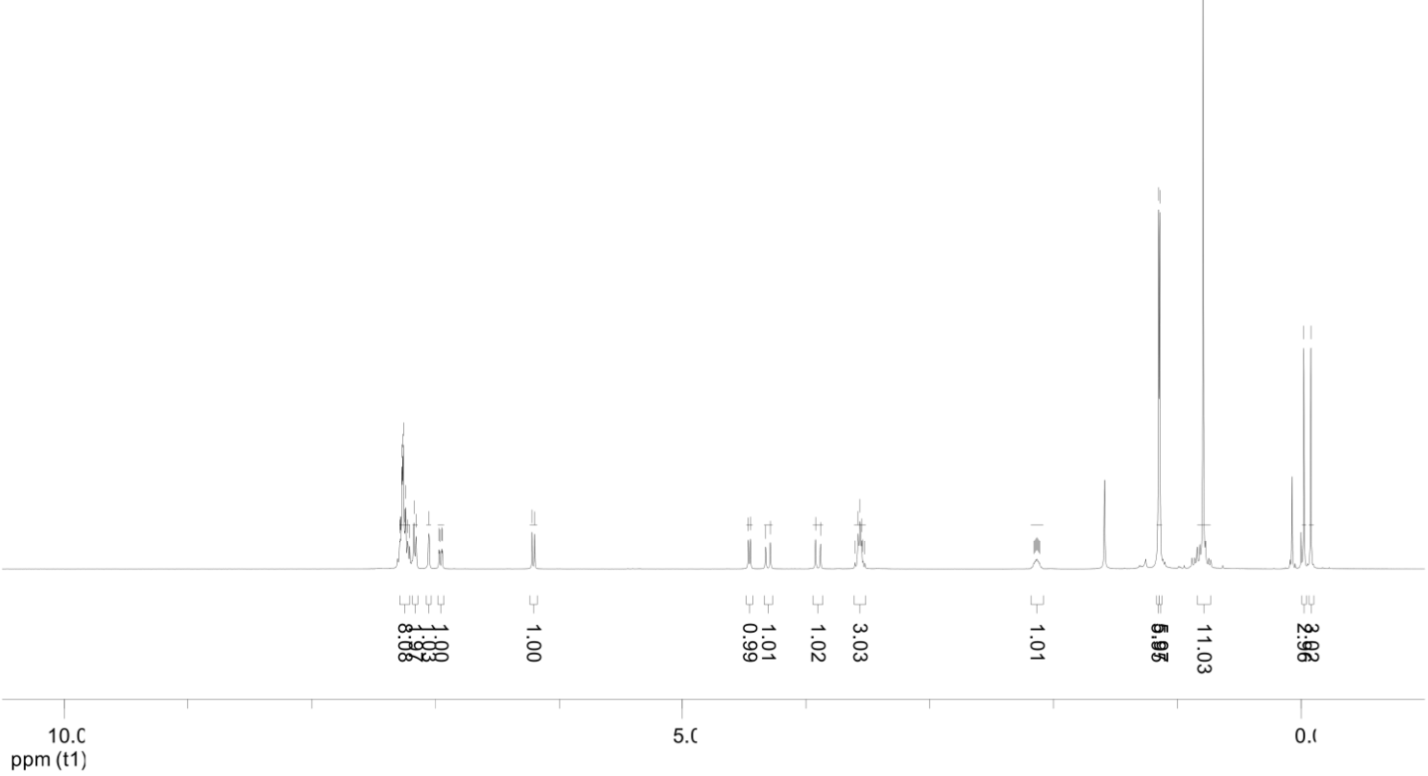

Supplementary Figure 184. ^{1}H NMR spectrum of **8a** (400 MHz, $CDCl_3$)

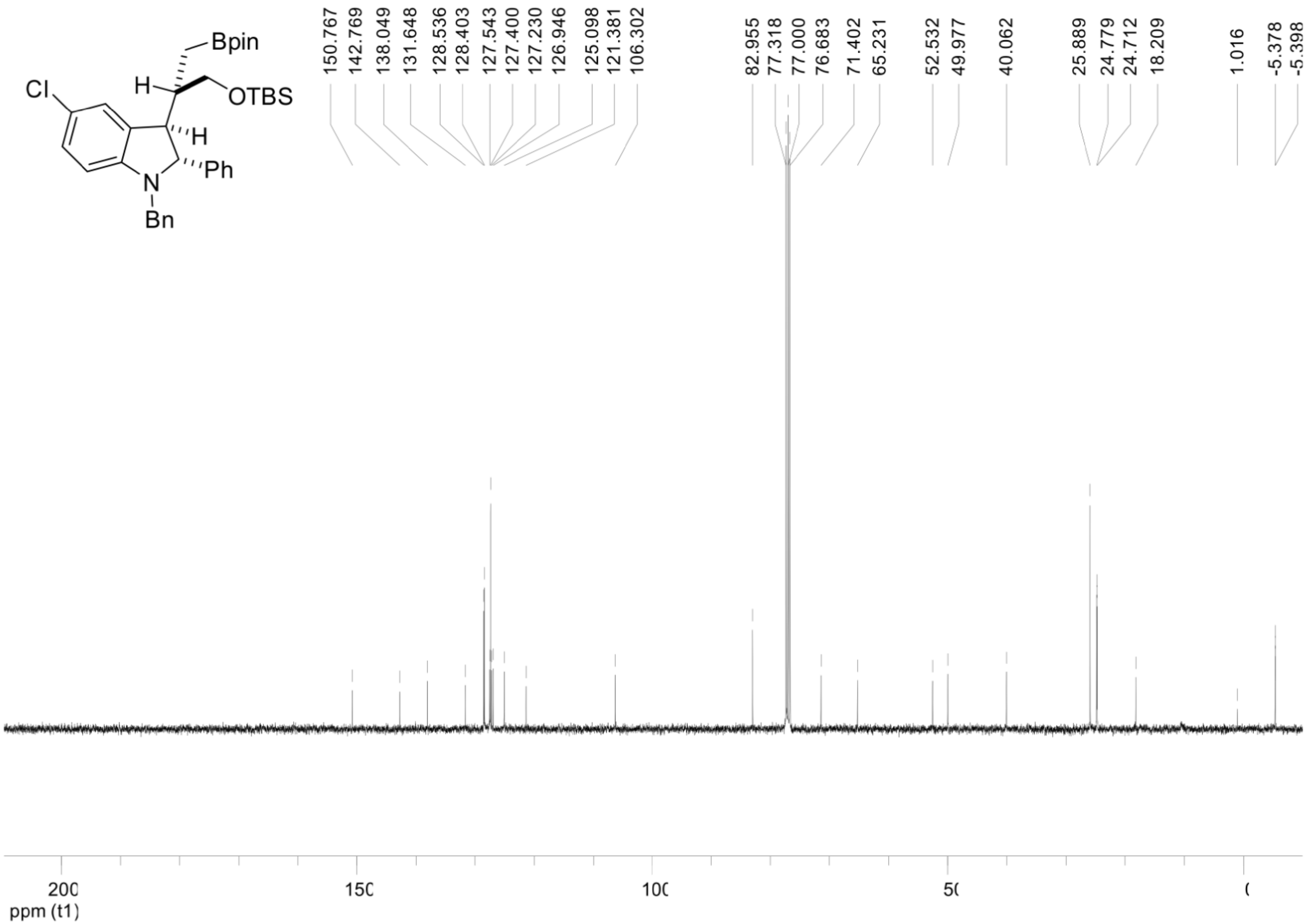

Supplementary Figure 185. ^{13}C NMR spectrum of **8a** (100 MHz, $CDCl_3$)

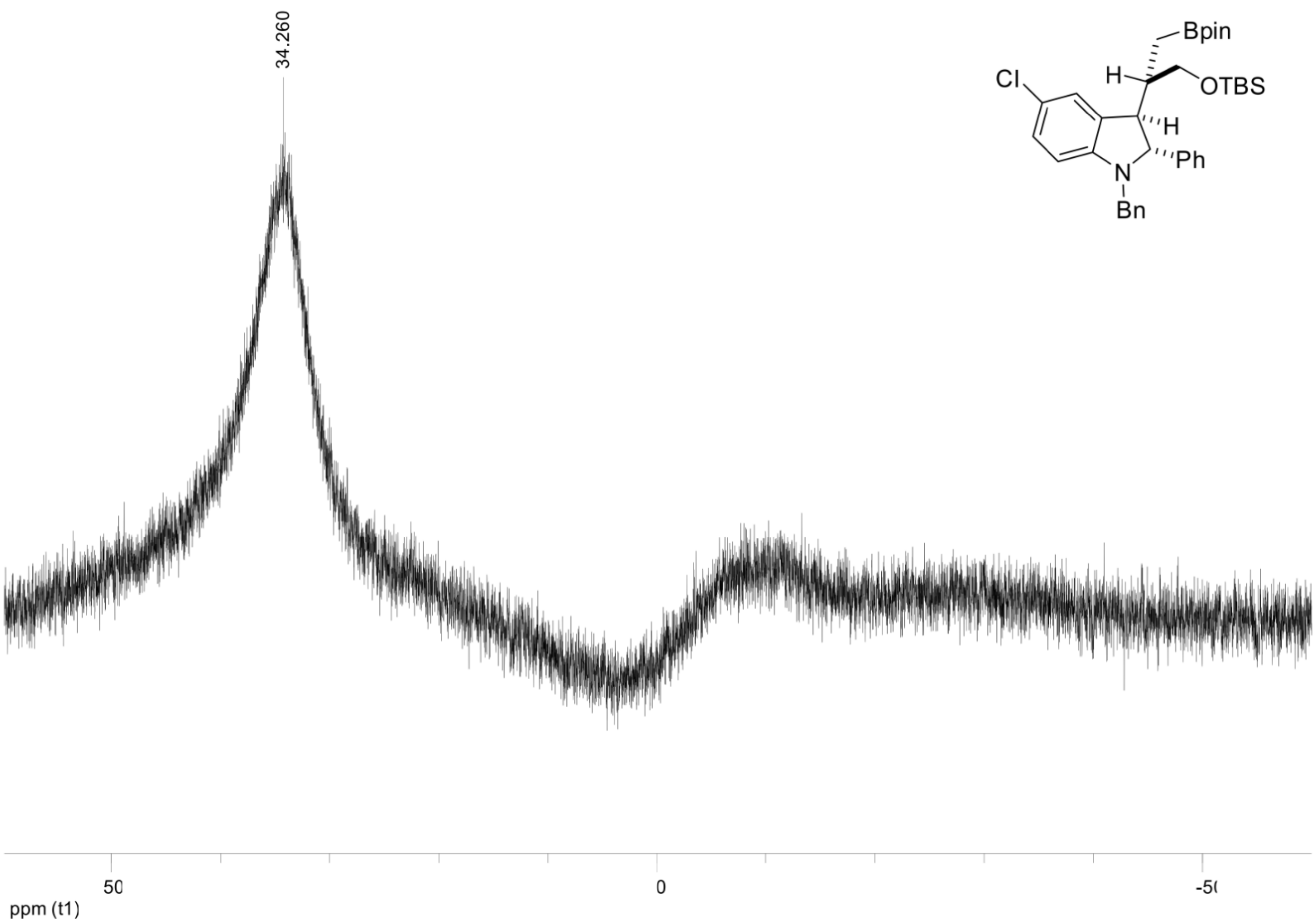

Supplementary Figure 186. ^{11}B NMR spectrum of **8a** (128.4 MHz, $CDCl_3$)

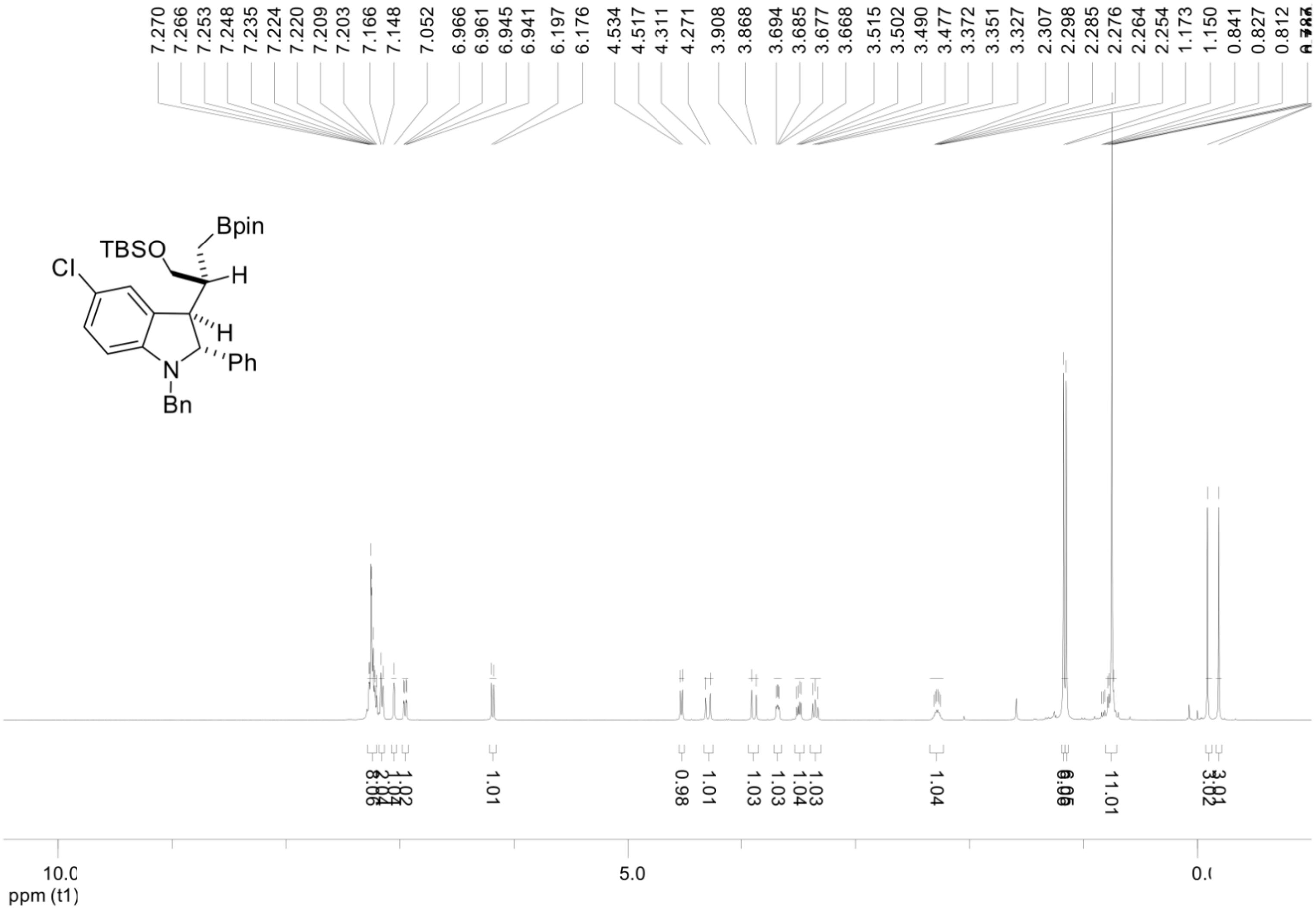

Supplementary Figure 187. ^{1}H NMR spectrum of **8aa** (400 MHz, $CDCl_3$)

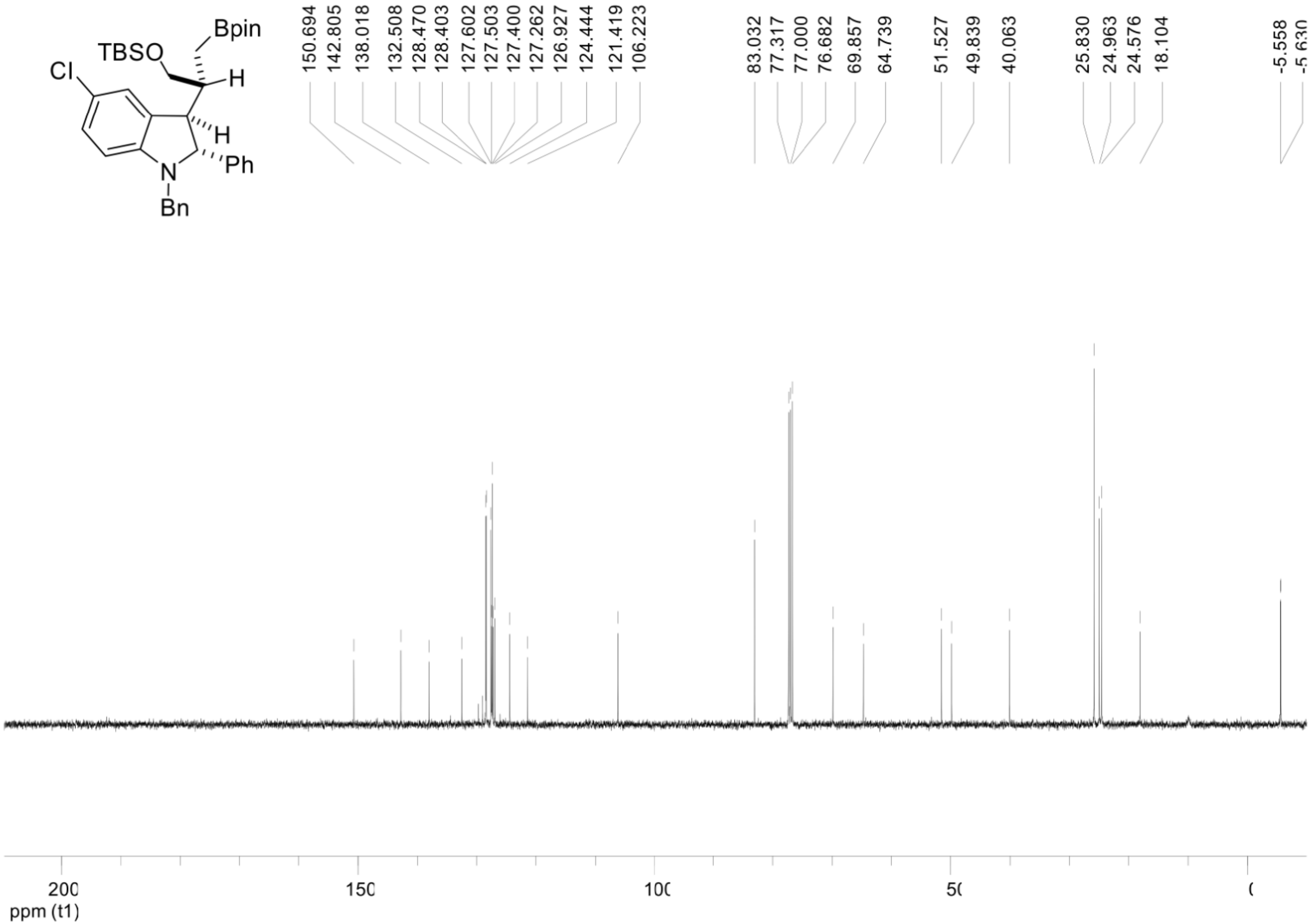

Supplementary Figure 188. ^{13}C NMR spectrum of **8aa** (100 MHz, $CDCl_3$)

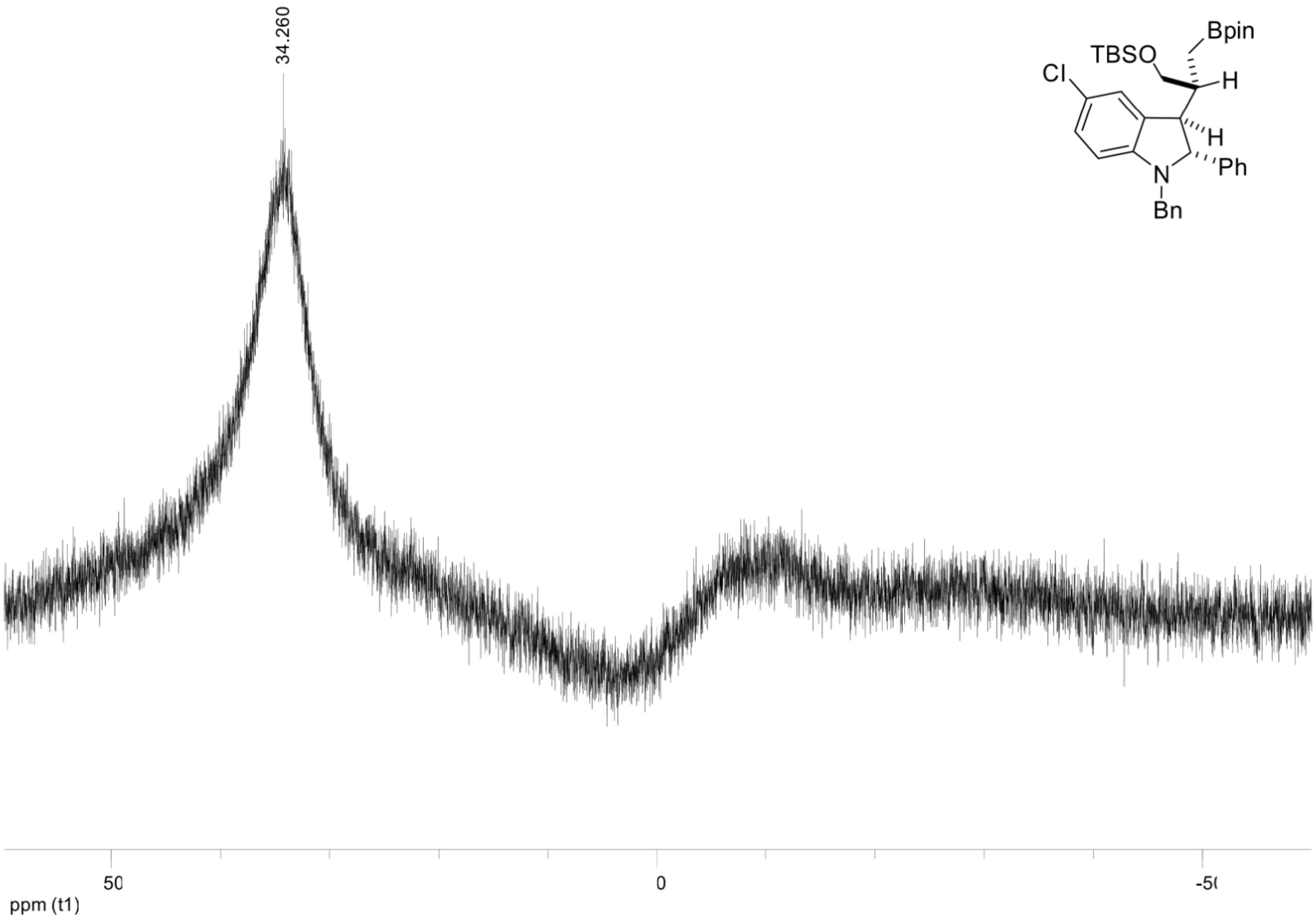

Supplementary Figure 189. ^{11}B NMR spectrum of **8aa** (128.4 MHz, $CDCl_3$)
