# Supplementary material for: Stereoselective hydrogen atom transfer to acyclic radicals: a switch enabling diastereodivergent borylative radical cascades
Source: Nat Commun. 2022 Jan 20;13:426. doi: 10.1038/s41467-022-28071-8 (PMC8776760; doi:10.1038/s41467-022-28071-8)
Supplement: Supplementary file 2 — Description of Additional Supplementary Files [file 41467_2022_28071_MOESM2_ESM.pdf]

Description of Additional Supplementary Files

File name: Supplementary Data 1

Description: Cartesian coordinates for all optimized geometries in the computational studies of HAT controlled by NHC-BH3/thiol catalyst.
