# Supplementary material for: Stereoselective hydrogen atom transfer to acyclic radicals: a switch enabling diastereodivergent borylative radical cascades
Source: Nat Commun. 2022 Jan 20;13:426. doi: 10.1038/s41467-022-28071-8 (PMC8776760; doi:10.1038/s41467-022-28071-8)
Supplement: Supplementary file 3 — Supplementary Data 1 [file 41467_2022_28071_MOESM3_ESM.pdf]

Cartesian coordinates for all optimized geometries in the computational studies of HAT controlled by NHC-BH$_3$/thiol catalyst

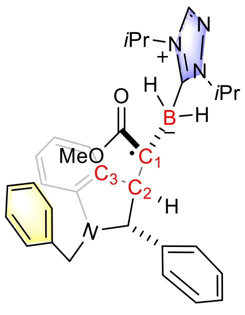

ϕ = D(B-C$_1$-C$_2$-C$_3$)

Geometries of **Int-III-A** with varied frozen dihedral angles (ϕ) of B-C$_1$-C$_2$-C$_3$:

ϕ = -179.5°

O	0.13814400	1.88637300	1.09625600
O	-2.07029200	1.59103900	1.47841100
N	2.85709700	-0.43005100	0.48539700
N	-3.01159500	0.46853500	-1.32719400
N	-4.24258300	-0.60346300	0.08458600
C	-0.96242600	1.09403000	1.26730300
C	1.57040800	-0.05542700	2.35049200
C	1.75713200	-2.45114100	-0.36416200
C	2.78714200	0.22187800	1.71079000
C	0.67269100	-0.88990900	1.45592100
H	0.51898700	-1.89000500	1.88021600
C	-0.69344000	-0.32958100	1.10800200
C	2.38377800	-3.40833500	0.44461600
H	2.71191000	-3.12489800	1.44086900
C	-2.96212900	-0.45162500	-0.33316200
C	3.01899900	1.02526800	-1.52781600
C	2.59931600	-4.70246200	-0.02429600
H	3.08261000	-5.43602600	0.61500400
C	1.55778700	-1.03836000	0.14256700
H	1.08575100	-0.44912600	-0.65192900
C	-5.08776400	0.22595400	-0.63774800
H	-6.14913500	0.26754600	-0.46150300
C	-0.01004800	3.26586300	1.44007200
H	0.99465900	3.68818700	1.39828500
H	-0.66649800	3.78458900	0.73542900
H	-0.42178800	3.37156900	2.44717400
C	1.37087100	-2.81051700	-1.65854200
H	0.89000300	-2.07007700	-2.29153600
C	3.69347900	0.00560800	-0.62102500

H	4.62196700	0.41271800	-0.21021600
H	3.96967200	-0.87769300	-1.20841400
C	2.20001800	-5.05583200	-1.31678000
H	2.36902000	-6.06438200	-1.68324500
C	-1.82584000	1.03661900	-2.00406800
H	-0.96953700	0.66592800	-1.44194700
C	-4.31425000	0.89494000	-1.52870700
H	-4.57590600	1.62464400	-2.27622800
C	3.74642800	1.02983300	2.32902500
C	2.62299000	2.27145000	-1.02167800
H	2.81482200	2.50620500	0.02036200
C	1.58812400	-4.10550900	-2.13411500
H	1.27959000	-4.37034800	-3.14163900
C	-4.63585000	-1.35473100	1.29518900
H	-3.80333500	-2.03570500	1.47750200
C	1.97128600	3.19248200	-1.83903400
H	1.67280700	4.15569200	-1.43494400
C	1.69896800	2.87984200	-3.17498200
H	1.19728600	3.60180900	-3.81316400
C	2.72498700	0.71328800	-2.85915700
H	3.00533800	-0.26124500	-3.25121400
C	2.07184100	1.63458900	-3.68242100
H	1.85817900	1.38035300	-4.71693300
C	-1.84949200	2.56239300	-1.89318000
H	-2.02995000	2.85374500	-0.85597200
H	-0.88433700	2.95987900	-2.21367000
H	-2.62762700	3.00395900	-2.52550300
C	-1.72335200	0.52895700	-3.44179600
H	-2.57712300	0.86161800	-4.04263200
H	-0.80731100	0.91518700	-3.89793500
H	-1.68817900	-0.56381500	-3.45981600
C	-4.75722500	-0.38204000	2.47270700
H	-5.60181400	0.30090000	2.32616500
H	-4.92458500	-0.93688700	3.40105700
H	-3.84522100	0.21379700	2.56046900
B	-1.64331900	-1.22323400	0.17096600
C	-5.90454300	-2.17073700	1.04708200
H	-5.79075200	-2.82480200	0.17799200
H	-6.11001500	-2.79308000	1.92253700
H	-6.77935400	-1.53032100	0.89064700
H	-1.95075700	-2.26846000	0.72008900
H	-1.02283200	-1.53500000	-0.83287600
C	1.31934400	0.42967200	3.62282200
H	0.37044600	0.21188600	4.10679000

C	2.27519000	1.23559400	4.26063700
C	3.47185100	1.53222200	3.60740200
H	4.21143900	2.16132500	4.09541900
H	2.08201100	1.62990300	5.25344700
H	4.68528900	1.26650700	1.83840800

ϕ = -150°

O	-0.31178400	1.78885700	1.19323000
O	1.94254500	1.64977900	1.26529500
N	-2.80912300	0.39128900	-1.00105800
N	2.93274300	-1.31168500	0.69608800
N	4.38928600	0.06868300	-0.09503300
C	0.89708600	1.47990100	0.62825600
C	-1.38364100	2.17103700	-1.31979500
C	-1.40719300	-1.50435700	-1.72358600
C	-2.70210900	1.78353900	-1.03431600
C	-0.49935700	0.94483100	-1.45352700
H	-0.27570700	0.77066700	-2.51269900
C	0.81193800	0.91908900	-0.70686400
C	-1.62410900	-1.52941200	-3.10725200
H	-1.82683800	-0.59899100	-3.62923400
C	3.11769400	-0.36916100	-0.26221900
C	-3.50783400	-0.60700600	1.17675600
C	-1.58873300	-2.73165300	-3.80989400
H	-1.75102900	-2.73404500	-4.88400100
C	-1.45869500	-0.20516800	-0.94936600
H	-1.20306000	-0.41384100	0.09831400
C	4.98614000	-0.57591600	0.97870800
H	5.99495700	-0.36537700	1.29112000
C	-0.25511000	2.40859500	2.47813500
H	-1.27382100	2.73001400	2.69597900
H	0.08562800	1.70187700	3.24200900
H	0.42341900	3.26555600	2.46944600
C	-1.19213500	-2.71493600	-1.05730300
H	-1.07148800	-2.70945200	0.02195700
C	-3.86037100	-0.31069400	-0.27425600
H	-4.77425000	0.28672300	-0.33675700
H	-4.06415900	-1.25303900	-0.79500300
C	-1.34604200	-3.93352700	-3.13761400
H	-1.31588600	-4.86975300	-3.68744600
C	1.65937700	-2.01516400	0.94189300
H	0.99021100	-1.65220500	0.16424500
C	4.07083000	-1.44488000	1.47424400

H	4.13114800	-2.13821500	2.29640300
C	-3.70184400	2.74428300	-0.86432400
C	-3.22388000	0.43594000	2.06922100
H	-3.29157800	1.46301200	1.72428000
C	-1.15358600	-3.92328400	-1.75674200
H	-0.97916000	-4.85289100	-1.22193000
C	4.99263000	1.20936100	-0.81418000
H	4.31237500	1.40107900	-1.64466900
C	-2.82855300	0.16023200	3.37705700
H	-2.61010800	0.97771600	4.05779700
C	-2.70532400	-1.16190400	3.81336500
H	-2.39419100	-1.37381600	4.83220200
C	-3.38074400	-1.92712800	1.62163500
H	-3.57857600	-2.74185500	0.92995800
C	-2.98401500	-2.20708100	2.93197100
H	-2.89092200	-3.23846200	3.26081600
C	1.09333300	-1.62995400	2.31244500
H	1.08798100	-0.54476000	2.43010600
H	0.06741200	-1.99732400	2.41207300
H	1.68931500	-2.06250800	3.12401300
C	1.83610800	-3.52374600	0.76441100
H	2.54236500	-3.93351200	1.49472700
H	0.87586600	-4.02662900	0.90894100
H	2.19594000	-3.75488200	-0.24145800
C	5.02202900	2.43541800	0.10312100
H	5.70738600	2.27884500	0.94395100
H	5.36740800	3.31032100	-0.45630300
H	4.02272400	2.62537500	0.50072100
B	2.01643100	0.08404700	-1.34888700
C	6.36731800	0.83309200	-1.36946600
H	6.30618400	-0.05669000	-2.00235300
H	6.75519600	1.65969800	-1.97159500
H	7.09135300	0.64205300	-0.56987300
H	2.54104200	0.72956600	-2.24515100
H	1.56512000	-0.92354800	-1.87040900
C	-1.05685200	3.50755400	-1.46366700
H	-0.03018400	3.79278500	-1.67813200
C	-2.05151200	4.48423100	-1.29842500
C	-3.35716200	4.09609100	-0.99892300
H	-4.12718500	4.85098700	-0.86470600
H	-1.80392100	5.53661700	-1.39725400
H	-4.72364500	2.46221100	-0.63082300

$\phi = -120°$

O	0.51779600	-1.38916000	1.29262800
O	-1.69333200	-1.06764200	1.64461500
N	2.81084400	-0.09752000	-1.32955700
N	-3.56706400	1.19952300	0.41324600
N	-4.47028000	-0.72282000	0.03495700
C	-0.73354100	-1.03929400	0.86308600
C	1.29036300	-1.82733100	-1.49701400
C	1.29342900	1.88186200	-1.22519900
C	2.65139200	-1.48200700	-1.49713700
C	0.46191200	-0.56419400	-1.34877800
H	0.17457100	-0.23760000	-2.35652500
C	-0.79772800	-0.63150500	-0.52717400
C	1.43837700	2.29785100	-2.55441700
H	1.77412200	1.57769000	-3.29453500
C	-3.39406100	0.04224600	-0.27048900
C	4.09504400	0.34116900	0.77297700
C	1.16305800	3.61504000	-2.91803000
H	1.27641800	3.92446800	-3.95316400
C	1.53471300	0.44880800	-0.81733600
H	1.53444600	0.39503400	0.27647400
C	-5.30000100	-0.05630400	0.92510600
H	-6.20886500	-0.49134400	1.30513400
C	0.62971300	-1.74926800	2.67100800
H	1.69059200	-1.93402700	2.83771600
H	0.28105800	-0.93798900	3.31761800
H	0.03745500	-2.64303500	2.89016500
C	0.88893600	2.81669300	-0.26599600
H	0.80099000	2.50266500	0.77044900
C	4.03230900	0.45829300	-0.74153200
H	4.88782600	-0.03933400	-1.20666000
H	4.08381700	1.51321300	-1.03079700
C	0.74076800	4.53806900	-1.95649500
H	0.52654000	5.56393100	-2.24188600
C	-2.57599400	2.29173900	0.47426000
H	-1.77066700	1.98635200	-0.19306700
C	-4.73269600	1.15246500	1.16057300
H	-5.05531900	1.97172500	1.78101000
C	3.62655500	-2.46101200	-1.69764700
C	4.38939100	-0.88186800	1.39186000
H	4.62052400	-1.74847000	0.78145100
C	0.60802000	4.13678200	-0.62666800
H	0.29796700	4.85125400	0.13136800
C	-4.63792100	-2.13740300	-0.35385500
H	-3.87675500	-2.30492200	-1.11686000

C	4.37068300	-0.99718200	2.78188200
H	4.60917300	-1.94999900	3.24648300
C	4.04920500	0.10703200	3.57583400
H	4.03425600	0.01553500	4.65808900
C	3.77482000	1.44095000	1.57844300
H	3.53079100	2.38976400	1.10733100
C	3.74971800	1.32760000	2.96984400
H	3.49789500	2.19156800	3.57854900
C	-2.02533100	2.40339600	1.89865200
H	-1.64669800	1.43376300	2.23103700
H	-1.21607700	3.13828500	1.92441700
H	-2.79909800	2.73348800	2.60059400
C	-3.17321200	3.59777100	-0.04960100
H	-3.98219100	3.96010400	0.59457800
H	-2.39403100	4.36429100	-0.08321000
H	-3.56513500	3.46373300	-1.06131900
C	-4.34901800	-3.03833600	0.85100800
H	-5.10728300	-2.90444800	1.63083100
H	-4.36353100	-4.08822600	0.54247900
H	-3.37154000	-2.79562100	1.27402500
B	-2.16866100	-0.27844200	-1.26351800
C	-6.01961800	-2.37083800	-0.96717200
H	-6.19235700	-1.69940400	-1.81284600
H	-6.09098300	-3.40226400	-1.32406600
H	-6.82016600	-2.22058900	-0.23467200
H	-2.46223800	-1.20429000	-2.01029700
H	-2.00487100	0.69704900	-1.98083100
C	0.88852700	-3.13659800	-1.69117300
H	-0.16959700	-3.38341300	-1.68434600
C	1.86050300	-4.13131700	-1.88043400
C	3.21216600	-3.78669000	-1.88598600
H	3.96427800	-4.55686200	-2.03397600
H	1.56134300	-5.16537100	-2.02176000
H	4.68353600	-2.21408900	-1.70424600

ϕ = -90°

O	0.48555700	-1.21014000	1.38608500
O	-1.71104900	-0.81299900	1.73994600
N	2.81641500	-0.03523400	-1.31610700
N	-3.66741000	1.19722000	0.17823800
N	-4.55765200	-0.76575500	0.06711100
C	-0.75211300	-0.81596300	0.95981400
C	1.12530700	-1.56585200	-1.69463600

C	1.48781300	2.03043200	-0.87090700
C	2.51378600	-1.34656700	-1.71102600
C	0.44242000	-0.27916200	-1.25564700
H	0.14362300	0.23683600	-2.17892000
C	-0.81552600	-0.42765200	-0.44078500
C	1.69880800	2.60568800	-2.13071400
H	2.02389900	1.96766200	-2.94706600
C	-3.46938500	-0.05136400	-0.31289000
C	4.23439500	-0.05174400	0.74308800
C	1.50138000	3.97136000	-2.32871000
H	1.66770200	4.40435300	-3.31110300
C	1.62695600	0.54129100	-0.65351900
H	1.68187100	0.34838000	0.42102000
C	-5.42040200	0.02688900	0.81174800
H	-6.34380400	-0.34788900	1.21956800
C	0.58702900	-1.57676000	2.76559100
H	1.64044500	-1.80189300	2.93006600
H	0.26613500	-0.75468800	3.41264500
H	-0.03772400	-2.44859900	2.98234300
C	1.09227000	2.85359200	0.18903300
H	0.94288200	2.41484400	1.17212400
C	4.11281500	0.30721300	-0.72885800
H	4.89228700	-0.18642000	-1.31575000
H	4.25576900	1.38564700	-0.85659900
C	1.09038800	4.78433700	-1.26810900
H	0.93908600	5.84880200	-1.42256100
C	-2.67783100	2.29295800	0.12281300
H	-1.82908700	1.88824800	-0.42903900
C	-4.86139900	1.25980400	0.87837700
H	-5.20707600	2.16332800	1.35194100
C	3.38098700	-2.35505000	-2.13636200
C	4.43101700	-1.37929400	1.14793400
H	4.53875000	-2.15645300	0.39829200
C	0.88990200	4.22229800	-0.00646000
H	0.58757700	4.84960500	0.82787600
C	-4.71021100	-2.22624000	-0.09356600
H	-3.95672300	-2.50317500	-0.83140300
C	4.47264700	-1.70963700	2.50242500
H	4.63316100	-2.74195000	2.80053000
C	4.31056200	-0.71790800	3.47401700
H	4.34504700	-0.97605000	4.52861400
C	4.06731800	0.93276600	1.72475600
H	3.89518400	1.96211900	1.41975800
C	4.10385600	0.60518100	3.08141200

H	3.97248800	1.38195400	3.82949000
C	-2.22030100	2.63495000	1.54338600
H	-1.86343700	1.73313400	2.04678700
H	-1.41308600	3.36914200	1.49872500
H	-3.03758400	3.06492400	2.13285100
C	-3.23280500	3.49506400	-0.64104400
H	-4.08556800	3.94508500	-0.12030900
H	-2.45129900	4.25473700	-0.73475500
H	-3.55229000	3.20192600	-1.64464500
C	-4.38915100	-2.92083100	1.23369100
H	-5.13277400	-2.66639700	1.99740900
H	-4.39942700	-4.00684100	1.09875200
H	-3.40546900	-2.60781700	1.59200600
B	-2.21535000	-0.50513100	-1.21796300
C	-6.09601800	-2.57329700	-0.63964600
H	-6.29766700	-2.03748100	-1.57138300
H	-6.14846400	-3.64716100	-0.84030500
H	-6.88932900	-2.33561200	0.07733000
H	-2.37941900	-1.65092500	-1.61224900
H	-2.19939500	0.22406900	-2.19668300
C	0.59066200	-2.77829900	-2.09196800
H	-0.48405600	-2.92800200	-2.06678800
C	1.45554600	-3.80092200	-2.51326000
C	2.83274900	-3.58201700	-2.53412200
H	3.50018700	-4.37548500	-2.85931400
H	1.05211300	-4.76120800	-2.81959800
H	4.45585400	-2.20519600	-2.15793300

ϕ = -60°

O	-1.99140000	-0.02556000	2.02502600
O	0.25377800	0.20233800	2.10099100
N	2.71590300	-0.36958900	-1.31412600
N	-3.57431400	0.99073700	-0.61256800
N	-4.68132400	-0.71666500	0.10812100
C	-0.73825300	-0.10993100	1.44366100
C	1.01799700	-1.93345400	-1.21469400
C	1.56072300	1.70621800	-0.57337000
C	2.37043200	-1.70767700	-1.54086100
C	0.43224200	-0.59086400	-0.78024600
H	0.09858300	-0.14917300	-1.73970500
C	-0.80799100	-0.57339000	0.07044400
C	1.42691700	2.26146300	-1.85427700
H	1.46254100	1.60286900	-2.71720400

C	-3.45603400	-0.33239700	-0.33431400
C	4.62309700	-0.13879300	0.29629700
C	1.28039700	3.63851800	-2.02330000
H	1.18430700	4.05342700	-3.02310600
C	1.70580200	0.20843400	-0.39567800
H	1.97625900	-0.00942800	0.64103300
C	-5.54889500	0.36679400	0.13560900
H	-6.57272700	0.28503100	0.45736600
C	-1.99697200	0.43504400	3.37775400
H	-3.04247900	0.42160000	3.69015900
H	-1.39742000	-0.21681300	4.01957700
H	-1.59242800	1.44986500	3.44898300
C	1.55367900	2.56054100	0.53558400
H	1.64361600	2.13113400	1.52706000
C	4.10267000	0.05129600	-1.11886200
H	4.72262000	-0.49190300	-1.83775400
H	4.16517400	1.11110900	-1.38812700
C	1.27637900	4.48475600	-0.91022400
H	1.17195000	5.55827700	-1.04020100
C	-2.48325800	1.86898400	-1.08771200
H	-1.62599900	1.21257300	-1.22456400
C	-4.85326200	1.43727000	-0.32035500
H	-5.15505300	2.46078000	-0.46695000
C	3.15671000	-2.74134400	-2.05415400
C	4.89836900	-1.41854400	0.79853600
H	4.76597700	-2.28464500	0.15794000
C	1.41546000	3.94043500	0.36800400
H	1.41527800	4.59057200	1.23856200
C	-5.00167600	-2.06951300	0.61680700
H	-4.32668200	-2.73915900	0.08318100
C	5.32196700	-1.58656400	2.11622900
H	5.53130900	-2.58423000	2.49175800
C	5.47647800	-0.47806000	2.95237300
H	5.80614600	-0.61071200	3.97884100
C	4.77417400	0.96418700	1.14444400
H	4.54125500	1.95778200	0.77046500
C	5.20017500	0.79887400	2.46355300
H	5.31080200	1.66600400	3.10865300
C	-2.13690700	2.90355100	-0.01618800
H	-1.91517500	2.40812900	0.93111700
H	-1.25679900	3.47147600	-0.32435500
H	-2.96520500	3.60380300	0.14057900
C	-2.83683700	2.49763700	-2.43589700
H	-3.68894100	3.18125000	-2.35330300

H	-1.97835000	3.07124000	-2.79654300
H	-3.07774700	1.72830000	-3.17453200
C	-4.68902600	-2.14187500	2.11355700
H	-5.32610400	-1.44799100	2.67367400
H	-4.87151900	-3.15382000	2.48786700
H	-3.64412000	-1.87773800	2.28783100
B	-2.13976700	-1.25236400	-0.53038900
C	-6.44414000	-2.45508400	0.28760000
H	-6.65891700	-2.32571900	-0.77697700
H	-6.59807000	-3.50679200	0.54358900
H	-7.16911300	-1.87215800	0.86563800
H	-2.32291000	-2.32118900	0.02527500
H	-2.02129000	-1.43134500	-1.73049400
C	0.45138100	-3.18523400	-1.38058800
H	-0.58728000	-3.35062800	-1.12123500
C	1.23989000	-4.23275500	-1.88434100
C	2.57304800	-4.00401600	-2.22075500
H	3.17971200	-4.81733700	-2.60986300
H	0.80861500	-5.22136400	-2.00878000
H	4.19972300	-2.58390200	-2.30944400

$\phi = -30°$

O	1.63908300	-0.02497400	-1.60320400
O	-0.23286300	1.13687900	-2.09491700
N	-2.73935900	-1.27459800	0.32804000
N	3.03268800	2.10799600	0.33036800
N	3.88961300	0.13830100	0.53074100
C	0.44931900	0.56170700	-1.24199500
C	-1.66068900	0.02866700	1.90372200
C	-3.54486100	0.85247200	-0.67779800
C	-2.39063200	-1.15101600	1.67444200
C	-1.30136100	0.65565000	0.56344000
H	-1.50611600	1.73637700	0.56142200
C	0.16138000	0.46028300	0.17736000
C	-4.78728400	0.65447100	-0.06949800
H	-4.91085500	-0.17928600	0.61314600
C	2.74481800	0.83851000	0.71939300
C	-1.32551200	-2.88205700	-0.94922600
C	-5.85453700	1.51406700	-0.34137600
H	-6.81441500	1.34901600	0.14087000
C	-2.34829500	-0.04614100	-0.38430900
H	-1.88223300	-0.31042000	-1.33257600
C	4.88262800	0.95839600	0.01282900

H	5.87159300	0.59669100	-0.21348400
C	1.95254700	0.06028800	-2.99535900
H	2.90847800	-0.45157500	-3.11638700
H	1.18221100	-0.43104900	-3.59546600
H	2.03866200	1.10187700	-3.31920300
C	-3.38511900	1.92679600	-1.56592100
H	-2.41669100	2.07731100	-2.03341100
C	-2.69899500	-2.53998200	-0.39218200
H	-3.03595900	-3.32622000	0.29053500
H	-3.42889100	-2.49953600	-1.20906600
C	-5.69171400	2.57983200	-1.22642400
H	-6.52280900	3.24651500	-1.43957900
C	2.09427700	3.24951600	0.39029700
H	1.16748800	2.83185800	0.78337200
C	4.34368400	2.19600900	-0.11258000
H	4.77527400	3.11791800	-0.46482600
C	-2.74926800	-1.99244700	2.73172800
C	-0.24515600	-3.09566000	-0.08093300
H	-0.39890000	-3.02558900	0.99150800
C	-4.45157400	2.78225400	-1.83845200
H	-4.31646100	3.60728300	-2.53308800
C	4.04910500	-1.31264700	0.75059500
H	3.08461100	-1.63781300	1.14152900
C	1.02141200	-3.37665100	-0.58685900
H	1.84756800	-3.54407100	0.09731900
C	1.23150900	-3.44091200	-1.96692700
H	2.21994200	-3.66290400	-2.35850400
C	-1.10297900	-2.93342500	-2.32903400
H	-1.93097700	-2.75146300	-3.00983500
C	0.16757700	-3.21297900	-2.83874900
H	0.32295200	-3.25296300	-3.91329900
C	1.83353600	3.81302900	-1.00934500
H	1.38664600	3.06048400	-1.66226700
H	1.13585900	4.65244300	-0.93747200
H	2.75612600	4.18490400	-1.46830700
C	2.61751400	4.30195000	1.37164200
H	3.55563100	4.74590700	1.02122800
H	1.88473800	5.10709900	1.47549000
H	2.78894300	3.85965800	2.35667800
C	4.30939900	-2.01759300	-0.58210300
H	5.25377200	-1.69208000	-1.03190100
H	4.37013500	-3.09864900	-0.42402700
H	3.49104500	-1.81106600	-1.27280200
B	1.31974900	0.33948500	1.28641600

C	5.13473100	-1.57616900	1.79612000
H	4.90860300	-1.05598600	2.73079700
H	5.19497600	-2.64854400	2.00311900
H	6.11989200	-1.24854100	1.44619600
H	1.40159100	-0.79602400	1.71807900
H	1.07914100	1.06717100	2.23366500
C	-1.36754800	0.41929600	3.20253100
H	-0.82812800	1.34199400	3.38513700
C	-1.74331400	-0.40213500	4.27409200
C	-2.40782300	-1.60619800	4.03139600
H	-2.68784500	-2.24526100	4.86454800
H	-1.50971500	-0.10525500	5.29188600
H	-3.30563000	-2.90787000	2.55471600

$\phi = 0.1°$

O	0.68372600	2.92154900	0.56198600
O	2.22987900	1.78370300	-0.63140900
N	-2.74863100	0.01145100	1.18606800
N	4.24173000	-0.55035700	-0.11287400
N	2.60063600	-1.39438800	-1.23058200
C	1.34094200	1.77068500	0.22731500
C	-0.92612600	-0.41582400	2.53332200
C	-1.99741100	1.95240600	-0.13855800
C	-2.25601100	-0.76233300	2.24349400
C	-0.47352300	0.69688800	1.60591500
H	-0.56409300	1.65931100	2.12799100
C	0.88963200	0.59565100	0.95838700
C	-2.63323000	2.94813300	0.61330100
H	-2.85144400	2.75675300	1.65958300
C	2.91932200	-0.83762700	-0.03551000
C	-3.43769700	-1.37467600	-0.78236500
C	-2.98333800	4.16263200	0.02771900
H	-3.47251200	4.92849000	0.62320500
C	-1.60547600	0.64007300	0.49997400
H	-1.24323300	-0.03501400	-0.29168000
C	3.70805600	-1.42494300	-2.06286800
H	3.66919800	-1.81874500	-3.06447300
C	1.00220600	4.07144600	-0.22500800
H	0.35112000	4.86590400	0.14028500
H	2.05480900	4.34732000	-0.10993200
H	0.80030600	3.88057600	-1.28231700
C	-1.74437300	2.18670900	-1.49318400
H	-1.27001100	1.41112700	-2.08648000

C	-3.85172200	-0.43491800	0.34020100
H	-4.59997000	-0.90845900	0.98212800
H	-4.32327800	0.45533900	-0.08892600
C	-2.71044200	4.39691900	-1.32405200
H	-2.98485900	5.34377100	-1.78020800
C	5.01852100	0.17501500	0.91123200
H	4.33027800	0.28983300	1.74917600
C	4.74018900	-0.89732400	-1.35990600
H	5.77068500	-0.74429400	-1.63298000
C	-2.92931600	-1.72115700	3.00538000
C	-2.93598700	-2.65399500	-0.50076900
H	-2.86205000	-2.98421200	0.53053200
C	-2.09292200	3.40375700	-2.08424500
H	-1.88563000	3.57186700	-3.13743700
C	1.23163800	-1.76503700	-1.64187500
H	0.64174100	-1.73366100	-0.72500500
C	-2.52429200	-3.49631900	-1.53315300
H	-2.14423000	-4.48704200	-1.30051600
C	-2.60329700	-3.07207000	-2.86313300
H	-2.28824900	-3.73214200	-3.66631000
C	-3.49694000	-0.95328200	-2.11573000
H	-3.85771600	0.04687300	-2.34074700
C	-3.08720700	-1.79538100	-3.15227500
H	-3.14563600	-1.45409200	-4.18198700
C	5.40740300	1.56067900	0.38692100
H	4.51807600	2.09289100	0.04252900
H	5.88970800	2.13463200	1.18417000
H	6.11513700	1.48469400	-0.44628200
C	6.21945700	-0.65941800	1.36196500
H	6.93164100	-0.81499600	0.54412300
H	6.74782300	-0.14030000	2.16693200
H	5.89867400	-1.63712700	1.73203600
C	0.68996100	-0.72295100	-2.62451700
H	1.22386600	-0.77167800	-3.58047700
H	-0.37097300	-0.91056400	-2.81372600
H	0.81995300	0.28022300	-2.21234100
B	1.89687300	-0.63192000	1.19333100
C	1.19909900	-3.18885800	-2.19765700
H	1.60132900	-3.90067100	-1.47140300
H	0.16455400	-3.46130100	-2.41776900
H	1.77369100	-3.27573200	-3.12639400
H	1.29916300	-1.67940700	1.34193500
H	2.53062200	-0.42846100	2.22127100
C	-0.27495600	-0.99813200	3.61079600

H	0.75117000	-0.72547800	3.83133700
C	-0.93798700	-1.96184400	4.38345000
C	-2.25184200	-2.31650300	4.07575500
H	-2.76316900	-3.06777000	4.67152800
H	-0.42632100	-2.43573100	5.21540300
H	-3.95348900	-2.00248800	2.78174000

$\phi = 30°$

O	0.54501600	3.01824900	0.73750300
O	2.23611800	1.98174700	-0.35051900
N	-2.81781600	-0.18945600	1.09462700
N	4.02060200	-0.51581800	0.11223100
N	2.69580700	-1.00959900	-1.51671700
C	1.26046600	1.89804700	0.40729400
C	-1.04423800	-0.33735700	2.56798400
C	-2.14818700	1.78015000	-0.26510400
C	-2.31708700	-0.83174000	2.23604000
C	-0.64383400	0.72852400	1.56930400
H	-0.83086600	1.71274200	2.01490000
C	0.74761300	0.67365400	0.99122400
C	-2.98203800	2.69877600	0.38392800
H	-3.34684400	2.46186000	1.37890800
C	2.71813300	-0.74129900	-0.18772300
C	-3.16802800	-1.74082300	-0.83135300
C	-3.33749400	3.89399800	-0.23825800
H	-3.98333000	4.59988900	0.27664000
C	-1.70635200	0.51288700	0.42736400
H	-1.25064300	-0.15539800	-0.31672600
C	3.97295300	-0.92243900	-2.05036200
H	4.17703900	-1.08974800	-3.09440100
C	0.90655800	4.21083500	0.04022200
H	0.23788300	4.98605400	0.41588200
H	1.95153600	4.47699900	0.22524700
H	0.76099100	4.08397400	-1.03677300
C	-1.69748000	2.07090100	-1.55580700
H	-1.06622000	1.35372500	-2.06994300
C	-3.79265900	-0.82328300	0.20879400
H	-4.51041400	-1.36944600	0.82708600
H	-4.35118400	-0.02799500	-0.29670800
C	-2.86565400	4.18706600	-1.52148100
H	-3.14217000	5.11966100	-2.00500900
C	4.51879000	-0.07001600	1.42789400
H	3.63789200	-0.06575500	2.07039200

C	4.80523400	-0.61413700	-1.02569900
H	5.87143000	-0.46249000	-1.00794700
C	-2.93776300	-1.79455400	3.03540400
C	-2.50659100	-2.91543600	-0.44381500
H	-2.46869200	-3.18523900	0.60692900
C	-2.04637900	3.26996200	-2.18083200
H	-1.68343500	3.48305900	-3.18257300
C	1.46999300	-1.18710300	-2.31984000
H	0.66925900	-1.31979700	-1.59120800
C	-1.88279400	-3.72310000	-1.39345400
H	-1.37545300	-4.63050800	-1.07914700
C	-1.91224900	-3.37006600	-2.74576100
H	-1.43955500	-4.00945200	-3.48573500
C	-3.17051600	-1.38345000	-2.18422200
H	-3.65276200	-0.45814500	-2.48889500
C	-2.55179500	-2.19377100	-3.13943000
H	-2.57193500	-1.90719300	-4.18735500
C	5.07068800	1.35479900	1.32180800
H	4.32332700	2.01243500	0.87285600
H	5.32941800	1.72484300	2.31856700
H	5.97830800	1.38439000	0.70850600
C	5.53366300	-1.07264800	1.98098700
H	6.42534200	-1.13094800	1.34706800
H	5.85508700	-0.76177500	2.97933700
H	5.09369200	-2.07103000	2.05322400
C	1.20802000	0.08090300	-3.13791600
H	1.96817500	0.21075000	-3.91644100
H	0.23106300	0.00895500	-3.62598800
H	1.23161300	0.95952900	-2.48871800
B	1.46799800	-0.74529200	0.82712800
C	1.56187000	-2.44661800	-3.18107800
H	1.74985200	-3.32885700	-2.56310800
H	0.61447500	-2.59120400	-3.70479500
H	2.35489500	-2.36973000	-3.93287300
H	0.65249300	-1.57068200	0.44948200
H	1.86229200	-1.14835200	1.91080300
C	-0.39517200	-0.78110000	3.70816800
H	0.59032100	-0.39707000	3.95184700
C	-1.00674300	-1.74808800	4.51946000
C	-2.26447800	-2.24572000	4.17766300
H	-2.73500800	-2.99933000	4.80331000
H	-0.49890600	-2.11358500	5.40663800
H	-3.91688100	-2.19106900	2.78580000

ϕ = 60°

O	-0.88633800	1.66038500	-2.86424200
O	-2.62202300	1.55717600	-1.41972700
N	2.85091400	-0.85098800	-0.66906200
N	-3.54134500	-1.02075100	0.40270000
N	-3.39122500	0.81273000	1.52864300
C	-1.46382200	1.22323100	-1.70000900
C	0.87077900	-1.58971700	-1.58393000
C	2.49172200	1.62143300	-0.71731300
C	2.13648400	-1.96905300	-1.10694500
C	0.70415100	-0.08481400	-1.44786100
H	0.90501900	0.38154600	-2.41727800
C	-0.62155700	0.36634500	-0.89002100
C	3.46472000	1.82931100	-1.70204300
H	3.82070400	0.97618600	-2.27205100
C	-2.65631400	-0.08572200	0.82807200
C	3.45137400	-1.02287500	1.75112300
C	3.97140600	3.10735700	-1.93728600
H	4.73020700	3.25544500	-2.70079500
C	1.89288200	0.25744500	-0.47677500
H	1.50652700	0.21987800	0.54817500
C	-4.73128500	0.45609300	1.51673000
H	-5.48899600	1.04186200	2.00940500
C	-1.70324600	2.51509200	-3.66561700
H	-1.09881500	2.77389100	-4.53604200
H	-2.62106700	2.00544300	-3.97575000
H	-1.98434200	3.41836900	-3.11534200
C	2.03518600	2.71478700	0.02823800
H	1.28417300	2.55227400	0.79696000
C	3.93277700	-0.95875200	0.31193000
H	4.53014500	-1.83931600	0.05930500
H	4.58229900	-0.08699300	0.18098900
C	3.50607100	4.19518500	-1.19459100
H	3.90103300	5.19042700	-1.37785600
C	-3.19793700	-2.13932500	-0.49506000
H	-2.10835000	-2.15575200	-0.51915200
C	-4.82632000	-0.69536000	0.80724800
H	-5.68135900	-1.30397300	0.56538700
C	2.53930500	-3.30642100	-1.16746300
C	2.85867500	-2.18664100	2.26013600
H	2.78201300	-3.06730800	1.63014800
C	2.53469000	3.99542000	-0.21169300
H	2.17081400	4.83609100	0.37270100

C	-2.88077900	2.07710500	2.09477500
H	-1.80271900	2.03291300	1.93846000
C	2.34989100	-2.21324900	3.55773400
H	1.89220800	-3.12174900	3.93879800
C	2.42529200	-1.07613500	4.36567800
H	2.02833000	-1.09824400	5.37658900
C	3.51536800	0.11332300	2.56599500
H	3.95125500	1.02725000	2.17026600
C	3.00825600	0.08873400	3.86637600
H	3.06495100	0.97959300	4.48563000
C	-3.73301500	-1.84762400	-1.90015800
H	-3.39616200	-0.86393600	-2.23577900
H	-3.38100800	-2.61002400	-2.60156000
H	-4.82862800	-1.85690400	-1.91329300
C	-3.69178100	-3.46984200	0.07386800
H	-4.78566600	-3.51806500	0.11009500
H	-3.34374400	-4.28762400	-0.56354300
H	-3.30150200	-3.63012600	1.08246900
C	-3.45228100	3.26618300	1.31721800
H	-4.53566700	3.34923200	1.45982200
H	-2.99573400	4.19405400	1.67549700
H	-3.24625600	3.14782900	0.25148000
B	-1.06731200	-0.11387600	0.57535400
C	-3.16432300	2.14226400	3.59699400
H	-2.73600700	1.27835600	4.11272900
H	-2.72174200	3.05062800	4.01605000
H	-4.23978700	2.17196200	3.80372300
H	-0.53357500	0.61215500	1.40654900
H	-0.66264100	-1.23810600	0.79977200
C	0.02547900	-2.52046600	-2.16348600
H	-0.93201400	-2.20154100	-2.56562600
C	0.41610000	-3.86702800	-2.22553200
C	1.66228800	-4.24566700	-1.72496900
H	1.96644000	-5.28791700	-1.76826100
H	-0.24501100	-4.60835900	-2.66416100
H	3.50830300	-3.62119100	-0.79365800

$\phi = 90°$

O	2.04057800	2.28429300	1.46311800
O	0.18431100	2.38200300	2.76006100
N	-2.52082400	-0.99745400	-0.07155300
N	2.90309800	-0.69055100	0.49630600
N	3.48287600	0.74579700	-1.00602600

C	0.71747500	1.97923900	1.72453500
C	-1.41427300	-0.41845300	1.85538300
C	-3.41585100	1.27782500	-0.48864000
C	-2.00682600	-1.46529600	1.12362200
C	-1.34887700	0.83390400	0.98488200
H	-1.82329000	1.67570500	1.49838100
C	0.10566000	1.15606700	0.70330100
C	-4.61646900	0.99598400	0.17336200
H	-4.66941600	0.12146400	0.81459900
C	2.40424000	0.23025200	-0.36480900
C	-1.62208400	-2.14798900	-2.07579400
C	-5.72906900	1.81995800	0.00280000
H	-6.65555600	1.58990300	0.52174400
C	-2.18044200	0.42157200	-0.28267800
H	-1.54704100	0.51268400	-1.16870800
C	4.65170300	0.16486900	-0.53153300
H	5.62542300	0.43574600	-0.90324300
C	2.69251200	3.04391300	2.48320900
H	3.70985700	3.21052200	2.12472800
H	2.18509000	3.99816800	2.65204700
H	2.71008500	2.49576700	3.43074700
C	-3.35125900	2.39650000	-1.32744000
H	-2.42151300	2.61736400	-1.84677100
C	-2.83521500	-1.83669000	-1.21799300
H	-3.30018500	-2.75788500	-0.85283800
H	-3.59376200	-1.31335100	-1.81080000
C	-5.65520400	2.93743800	-0.83195400
H	-6.52246100	3.57770800	-0.96571600
C	2.09125900	-1.49917700	1.43090800
H	1.06323600	-1.17833500	1.27551400
C	4.28485300	-0.73940900	0.41014800
H	4.87745600	-1.40833800	1.01152200
C	-2.06299100	-2.75725100	1.66116800
C	-0.72109800	-3.15384600	-1.70092100
H	-0.93033700	-3.76035300	-0.82494700
C	-4.46161400	3.22436700	-1.49708700
H	-4.39731900	4.08854200	-2.15252100
C	3.44179500	1.85315500	-1.98136900
H	2.38804700	1.94787300	-2.24532200
C	0.44941800	-3.36448500	-2.42818800
H	1.13508800	-4.15375400	-2.13191500
C	0.73922600	-2.56755700	-3.53876100
H	1.65354900	-2.72972800	-4.10251100
C	-1.33104400	-1.36801100	-3.19976000

H	-2.02421200	-0.58541600	-3.49842800
C	-0.15608200	-1.57045700	-3.92512000
H	0.05939500	-0.94869200	-4.78938100
C	2.48962100	-1.18373400	2.87315900
H	2.40444000	-0.11086900	3.06459100
H	1.81725100	-1.70894000	3.55454800
H	3.51744400	-1.49794800	3.08671700
C	2.18656800	-2.98445700	1.08293100
H	3.20123300	-3.37260400	1.22805600
H	1.50628700	-3.54598700	1.72922600
H	1.88908700	-3.14829800	0.04428000
C	3.90475000	3.15042600	-1.31302000
H	4.94960600	3.07641700	-0.99085800
H	3.82505400	3.98457400	-2.01666700
H	3.28093700	3.35865200	-0.44115500
B	0.84288200	0.54368300	-0.58205400
C	4.24082900	1.49814600	-3.23666600
H	3.89596300	0.55232500	-3.66377500
H	4.11003400	2.28421300	-3.98577700
H	5.31357900	1.41656200	-3.03082600
H	0.69246300	1.29393000	-1.53679100
H	0.34488300	-0.52262700	-0.87704100
C	-0.93640800	-0.62976100	3.13862500
H	-0.49154200	0.19682900	3.68268100
C	-1.01307100	-1.91687200	3.69644200
C	-1.55808800	-2.96454200	2.95103500
H	-1.60573800	-3.96238700	3.37912900
H	-0.64518700	-2.09608800	4.70216700
H	-2.50115900	-3.58043800	1.10640000

$\phi = 120°$

O	1.25728900	1.70539100	2.93208100
O	2.95869200	0.78873800	1.75867000
N	-2.87831100	0.27542100	0.52051600
N	2.46775100	-1.67433000	-0.50049500
N	3.86734800	-0.17006000	-1.16151800
C	1.76372800	1.08860600	1.81471900
C	-1.20634600	-0.11140800	2.06573900
C	-2.03676600	2.33746500	-0.58181700
C	-2.48459300	-0.49258800	1.61490500
C	-0.67448700	0.99695300	1.14858800
H	-0.79460600	1.94845900	1.68870600
C	0.78346000	0.85254900	0.76579500

C	-2.99390800	3.14466100	0.04660600
H	-3.51022300	2.75928400	0.92072600
C	2.57764800	-0.35273400	-0.78681200
C	-3.31459800	-1.23947900	-1.41926600
C	-3.28935200	4.41327600	-0.45140700
H	-4.03654100	5.02730800	0.04395300
C	-1.69389600	0.96971300	-0.02832500
H	-1.26388700	0.36521700	-0.83270500
C	4.56634000	-1.36687500	-1.08763200
H	5.61214400	-1.44074200	-1.33406000
C	2.20328600	1.94628100	3.97720600
H	1.64714600	2.44392300	4.77260200
H	2.63213700	1.00734000	4.34155300
H	3.02120400	2.58225000	3.62609000
C	-1.38192200	2.82311400	-1.71999700
H	-0.63143400	2.20599300	-2.20308600
C	-3.86447600	-0.20976300	-0.44699000
H	-4.71581500	-0.61473200	0.10718700
H	-4.23011300	0.66314000	-0.99733200
C	-2.62916700	4.89344000	-1.58511800
H	-2.85974000	5.88122800	-1.97407700
C	1.25144500	-2.32910200	0.02726900
H	0.48835600	-1.55164100	0.04432300
C	3.68662000	-2.31110700	-0.67241200
H	3.81855800	-3.36447800	-0.49033400
C	-3.20660200	-1.49079000	2.27721000
C	-3.28337400	-2.60236000	-1.09268300
H	-3.70376700	-2.93666500	-0.14962300
C	-1.67438100	4.09491800	-2.21634100
H	-1.15663100	4.46030100	-3.09892900
C	4.49455700	1.13290300	-1.46044100
H	3.66609800	1.84150000	-1.46997700
C	-2.71423400	-3.53029000	-1.96521900
H	-2.70643700	-4.58444900	-1.70215900
C	-2.15814900	-3.10758800	-3.17505100
H	-1.71457900	-3.83064300	-3.85354300
C	-2.75677000	-0.82773800	-2.63698100
H	-2.76983900	0.22782300	-2.89688300
C	-2.17821200	-1.75236600	-3.50791800
H	-1.74685300	-1.41403800	-4.44554900
C	1.50378200	-2.79703200	1.46215000
H	1.89589300	-1.97142300	2.06059100
H	0.56765700	-3.13409200	1.91372500
H	2.22386600	-3.62264000	1.48984500

C	0.78837200	-3.44668400	-0.90628700
H	1.52682400	-4.25402300	-0.96693400
H	-0.14796000	-3.86741000	-0.53135800
H	0.60030900	-3.06095700	-1.91116500
C	5.46344800	1.51467600	-0.33774600
H	6.30873400	0.81876700	-0.29077400
H	5.86377800	2.51664000	-0.52103900
H	4.94250500	1.50332000	0.62169900
B	1.35978600	0.70251500	-0.73356600
C	5.14982200	1.10649000	-2.84301000
H	4.42763900	0.82302300	-3.61380000
H	5.54053300	2.09959200	-3.08294900
H	5.98930700	0.40352200	-2.87981800
H	1.73132000	1.79829400	-1.12011200
H	0.53896000	0.27726100	-1.52453800
C	-0.65509700	-0.71865800	3.18417500
H	0.32324000	-0.42060800	3.53973800
C	-1.36869300	-1.72546900	3.85247100
C	-2.63233300	-2.10136100	3.39713700
H	-3.18437000	-2.88091300	3.91510400
H	-0.93530300	-2.20834200	4.72291200
H	-4.19332000	-1.78912100	1.93918700

ϕ = 150°

O	-1.96679100	0.10271400	1.96258300
O	-0.60577400	-1.55177000	2.68514700
N	2.79796000	0.98920800	-0.12501400
N	-3.57287100	0.13462400	-0.72165300
N	-3.08414100	-1.92606200	-0.29964200
C	-0.86576100	-0.71107900	1.82671300
C	2.09790100	-0.16217800	1.74868900
C	2.99909000	-1.16435800	-1.34312900
C	2.83817800	0.93326500	1.26466300
C	1.33532200	-0.79241700	0.58571400
H	1.47175800	-1.88375300	0.57584800
C	-0.16123200	-0.50976200	0.57147300
C	4.35101000	-1.26108600	-0.99709100
H	4.75396800	-0.58152700	-0.25354700
C	-2.53342100	-0.72945300	-0.63029500
C	1.46826400	2.88683200	-1.06014500
C	5.17236700	-2.21100200	-1.60694000
H	6.22043000	-2.27546500	-1.32714600
C	2.06882200	-0.17296000	-0.65839100

H	1.33085400	0.16896000	-1.38507600
C	-4.46067300	-1.80941900	-0.18234200
H	-5.09422100	-2.64487900	0.06381800
C	-2.76621100	-0.14052300	3.12440200
H	-3.53377600	0.63497000	3.12419900
H	-3.23598100	-1.12818500	3.07773300
H	-2.16429800	-0.08499500	4.03505000
C	2.48541500	-2.03285400	-2.31515000
H	1.43573000	-1.95972900	-2.58765300
C	2.82774700	2.21848000	-0.89435700
H	3.52548700	2.90833000	-0.40755000
H	3.25148700	1.99885800	-1.88135100
C	4.65350000	-3.07426400	-2.57337700
H	5.29430400	-3.81080200	-3.05000700
C	-3.45274000	1.58663300	-0.97037400
H	-2.39994500	1.74720800	-1.19962300
C	-4.76694200	-0.51353900	-0.44259600
H	-5.71658500	-0.00543900	-0.45529700
C	3.54632300	1.76565000	2.13766900
C	0.68030500	3.18012700	0.06244800
H	1.04012000	2.91881200	1.05248000
C	3.30535400	-2.98116400	-2.92670600
H	2.89394400	-3.64362300	-3.68369400
C	-2.32944500	-3.19099600	-0.14680600
H	-1.28011400	-2.89721200	-0.16507200
C	-0.56069100	3.79503500	-0.08581000
H	-1.15887900	4.01794300	0.79271800
C	-1.03662100	4.12319000	-1.35914500
H	-2.00074700	4.61166600	-1.47302600
C	0.97570300	3.20199800	-2.32968100
H	1.56981100	2.96070100	-3.20783100
C	-0.26888000	3.81819900	-2.48264700
H	-0.63610400	4.05686700	-3.47685000
C	-3.80295300	2.36677300	0.29833900
H	-3.13679400	2.06936800	1.10997800
H	-3.68049000	3.43788500	0.11596800
H	-4.84132000	2.19218700	0.60222200
C	-4.29356000	1.99379700	-2.18109300
H	-5.36669200	1.87656400	-1.99368600
H	-4.10922300	3.04704600	-2.41104100
H	-4.02679300	1.39897700	-3.05897400
C	-2.62915800	-3.84744200	1.20210900
H	-3.68489100	-4.12437600	1.29535100
H	-2.03894500	-4.76379800	1.29196700

H	-2.34885200	-3.18664100	2.02486500
B	-0.96254900	-0.44078900	-0.82817600
C	-2.61297300	-4.10550100	-1.34112800
H	-2.36303200	-3.60093400	-2.27830200
H	-2.01027000	-5.01496800	-1.26508300
H	-3.66791900	-4.39933500	-1.37321200
H	-0.53321700	-1.31499500	-1.57042400
H	-0.80974700	0.61986000	-1.40279200
C	2.13250500	-0.47961600	3.09994800
H	1.57268900	-1.33042100	3.46711600
C	2.86087200	0.33317800	3.98127300
C	3.54241000	1.45254500	3.50079600
H	4.09508500	2.08587700	4.18971300
H	2.88594100	0.09397900	5.04002700
H	4.10489300	2.62048600	1.76919400

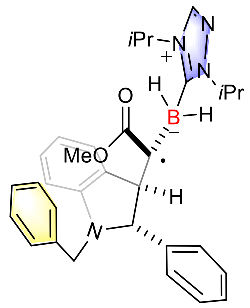

Int-III-A

O	0.51830000	-1.38385200	1.29236900
O	-1.69250500	-1.06170600	1.64576500
N	2.81142100	-0.09322000	-1.32937700
N	-3.57095400	1.19779500	0.40809900
N	-4.47274000	-0.72640700	0.03583900
C	-0.73308200	-1.03296000	0.86386500
C	1.28629800	-1.81830400	-1.50409000
C	1.29817200	1.88912700	-1.21466400
C	2.64822200	-1.47640300	-1.50395000
C	0.46151800	-0.55371400	-1.34776000
H	0.17383200	-0.22132000	-2.35352000
C	-0.79795600	-0.62419700	-0.52617100
C	1.44587200	2.31131700	-2.54162400
H	1.78321300	1.59457500	-3.28433200
C	-3.39706200	0.03848800	-0.27196200
C	4.10003300	0.33046600	0.77346700
C	1.17107600	3.63015300	-2.89967100
H	1.28666400	3.94444100	-3.93309300

C	1.53737300	0.45395300	-0.81301800
H	1.53844200	0.39565300	0.28049900
C	-5.30317400	-0.05753200	0.92359600
H	-6.21175700	-0.49200200	1.30493000
C	0.63082400	-1.74512000	2.67042800
H	1.69167500	-1.93073500	2.83634300
H	0.28306800	-0.93409400	3.31783200
H	0.03806200	-2.63864000	2.88920400
C	0.89130000	2.81935400	-0.25199500
H	0.80094000	2.50041500	0.78273900
C	4.03509500	0.45649600	-0.74020000
H	4.88876400	-0.04028100	-1.20960800
H	4.08834300	1.51295100	-1.02350800
C	0.74635100	4.54855900	-1.93478900
H	0.53250600	5.57569700	-2.21584200
C	-2.58084000	2.29113000	0.46566200
H	-1.77367800	1.98299200	-0.19820900
C	-4.73678100	1.15240100	1.15521400
H	-5.06013000	1.97340400	1.77295500
C	3.62059400	-2.45683500	-1.71088900
C	4.39686100	-0.89578600	1.38472000
H	4.62842200	-1.75843700	0.76889600
C	0.61070300	4.14100400	-0.60714200
H	0.29857100	4.85180300	0.15349600
C	-4.63905400	-2.14257100	-0.34785600
H	-3.87903900	-2.31157100	-1.11167800
C	4.38024500	-1.01934700	2.77406400
H	4.62065300	-1.97455900	3.23270500
C	4.05834300	0.07973200	3.57494200
H	4.04502600	-0.01815300	4.65665900
C	3.77935600	1.42504300	1.58584800
H	3.53342900	2.37630200	1.12066000
C	3.75625500	1.30342800	2.97657600
H	3.50403400	2.16341900	3.59071800
C	-2.03340400	2.41035600	1.89067100
H	-1.65411500	1.44281800	2.22840500
H	-1.22526100	3.14646900	1.91455200
H	-2.80908100	2.74270400	2.58942500
C	-3.17781100	3.59406200	-0.06611100
H	-3.98851800	3.95897400	0.57443200
H	-2.39904000	4.36092200	-0.10176500
H	-3.56738600	3.45463000	-1.07800500
C	-4.34642100	-3.03884500	0.85957700
H	-5.10327600	-2.90320100	1.64046300

H	-4.36002500	-4.08985800	0.55485000
H	-3.36844600	-2.79322700	1.27978600
B	-2.17128500	-0.28363100	-1.26406400
C	-6.02153800	-2.38026500	-0.95772200
H	-6.19719100	-1.71171700	-1.80508000
H	-6.09170000	-3.41288700	-1.31137700
H	-6.82095000	-2.22934600	-0.22412400
H	-2.46054400	-1.21702500	-2.00313000
H	-2.01381600	0.68746300	-1.98868900
C	0.88075100	-3.12542800	-1.70472700
H	-0.17802000	-3.36942000	-1.69808100
C	1.84997800	-4.12157800	-1.90054300
C	3.20251300	-3.78040400	-1.90584900
H	3.95244600	-4.55170900	-2.05891200
H	1.54799200	-5.15407800	-2.04711200
H	4.67820300	-2.21261700	-1.71755700

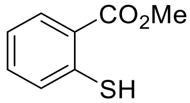

RSH E

C	-0.25732600	-1.75473500	-0.00021900
C	-0.03381300	-0.36141000	-0.00019500
C	-1.15496600	0.51026000	0.00002200
C	-2.44434500	-0.05338300	0.00018400
C	-2.63590500	-1.42727900	0.00014800
C	-1.53544200	-2.29016300	-0.00005700
H	0.60255900	-2.41233400	-0.00038000
H	-3.30457200	0.60951800	0.00034300
H	-3.64605300	-1.82632700	0.00027900
H	-1.67567300	-3.36609400	-0.00009200
S	-1.12311600	2.28892500	0.00014200
H	0.22492200	2.39864400	0.00008900
C	1.36999500	0.12998600	-0.00040200
O	1.73329600	1.29940000	-0.00018000
O	2.27203600	-0.87563900	0.00000600
C	3.65000900	-0.46699800	0.00020400
H	3.87484300	0.12872700	0.88851100
H	4.22684000	-1.39145700	0.00027800
H	3.87509300	0.12876100	-0.88801400

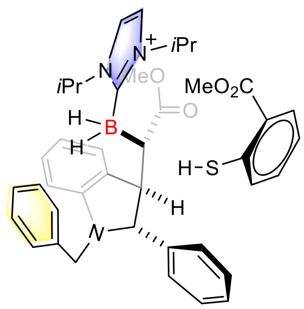

Int-III-A-cpx-1

O	1.10711000	2.34879400	-1.73112800
O	-0.54828200	1.73955000	-3.15415600
N	-3.10244600	-0.55152600	0.74560400
N	0.26879400	3.64208000	1.19402300
N	2.10866000	2.51172500	1.14845500
C	-0.08282800	1.71289000	-2.01154000
C	-3.10946200	1.21120600	-0.71724400
C	-2.72317500	-2.13166500	-1.13448100
C	-3.70964600	0.64720200	0.42703400
C	-1.95113300	0.32886500	-1.16339300
H	-2.01469700	0.11019200	-2.23226700
C	-0.66053200	1.05442500	-0.85971400
C	-3.93316600	-2.00409400	-1.83287000
H	-4.47340000	-1.06258700	-1.79271600
C	0.77012100	2.40186100	0.97719600
C	-2.46074100	-2.33210300	2.30271400
C	-4.44057800	-3.07045500	-2.57236900
H	-5.37762000	-2.95657700	-3.11008100
C	-2.17441400	-0.97175300	-0.32056900
H	-1.23632500	-1.28202300	0.14455500
C	2.44558900	3.81589600	1.47802500
H	3.45936100	4.12467900	1.66665500
C	1.72336100	2.99840000	-2.84263800
H	2.61617900	3.48397300	-2.44454300
H	2.00127600	2.27411900	-3.61385500
H	1.05389500	3.73930300	-3.28980800
C	-2.04364200	-3.35300100	-1.18375000
H	-1.12376700	-3.47830600	-0.61948800
C	-3.59535700	-1.51610600	1.70745000
H	-4.10364900	-0.96284800	2.50497100
H	-4.33950000	-2.19229000	1.25718300
C	-3.74671000	-4.28324400	-2.62511900
H	-4.14241500	-5.11334900	-3.20312800
C	-1.14725400	4.01434800	0.98976600
H	-1.66683600	3.07594100	0.79945800

C	1.28963300	4.52813100	1.50018600
H	1.10936300	5.56997700	1.70617700
C	-4.75164800	1.32043900	1.07454400
C	-1.37122900	-1.68552200	2.90128200
H	-1.34454200	-0.60105900	2.91914600
C	-2.54681600	-4.42238600	-1.92827400
H	-2.00402800	-5.36288300	-1.95605900
C	3.05769500	1.39296100	0.94194200
C	-0.30522400	-2.42363600	3.41314000
H	0.54200300	-1.91014000	3.85744500
C	-0.31351400	-3.81974000	3.33250900
H	0.52280500	-4.39304300	3.72121000
C	-2.46115000	-3.72756600	2.22258700
H	-3.28779400	-4.23386100	1.73176800
C	-1.39519600	-4.46991900	2.73691000
H	-1.40463100	-5.55327700	2.65958000
B	-0.09189800	1.09067300	0.63710200
H	0.58392300	0.10041200	0.84968900
H	-1.01541400	1.07081300	1.42774500
C	-3.52760300	2.43029200	-1.21961400
C	-4.57042300	3.11631500	-0.57343600
C	-5.16209000	2.55914900	0.56245300
H	-3.03901200	2.84009300	-2.09902400
H	-4.92195900	4.06706300	-0.96230900
H	-5.97260600	3.08682300	1.05823400
H	-5.23924900	0.90039900	1.94806800
H	0.62962900	-2.44842500	0.60582900
S	1.95517600	-2.63651600	0.75787200
C	2.39214300	-1.82531200	-0.76857100
C	3.73233300	-1.48270500	-1.08087900
C	1.38880100	-1.47775700	-1.69067900
C	4.01793800	-0.81444100	-2.28663500
C	4.82751900	-1.79974600	-0.13824000
C	1.68883000	-0.81001200	-2.87132200
H	0.35662200	-1.72823800	-1.48071000
C	3.01119600	-0.47586600	-3.17789200
H	5.04902300	-0.56352400	-2.50367200
O	4.70849800	-2.45961700	0.87994200
O	6.01526900	-1.24647100	-0.49954800
H	0.88094900	-0.52422500	-3.53723300
H	3.24903400	0.04126600	-4.10172900
C	7.10850100	-1.52528500	0.38991000
H	6.90420500	-1.12647900	1.38717200
H	7.27478700	-2.60233400	0.47168500

H	7.97608400	-1.03363900	-0.04978500
H	2.55317100	0.72813500	0.24241100
C	-1.73390100	4.64876600	2.25027200
H	-1.26490000	5.61213200	2.47995200
H	-2.80335600	4.81889100	2.09896300
H	-1.60940700	3.98595400	3.11085900
C	-1.27246700	4.90526100	-0.24798900
H	-0.77103700	5.86816700	-0.09921600
H	-0.82947100	4.41198800	-1.11684300
H	-2.32866100	5.09029700	-0.45701100
C	4.34728100	1.88834900	0.28814100
H	4.96347800	1.02902300	0.01526900
H	4.12877500	2.45243100	-0.62278400
H	4.93782900	2.51783200	0.96285800
C	3.29744600	0.64961100	2.25671200
H	3.78429500	1.30196100	2.99068300
H	2.34817100	0.29750200	2.66677100
H	3.92934400	-0.22571800	2.08568600

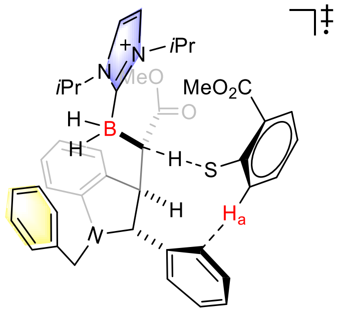

TS-A-major

O	2.40286900	0.49993100	-0.87063000
O	1.06336800	0.57321600	-2.68345500
N	-3.23374700	0.68480900	0.07475800
N	1.56573000	3.49573200	0.22036800
N	2.42464500	2.20861900	1.72759700
C	1.17714900	0.52860700	-1.46484400
C	-1.96358900	1.37210500	-1.70600300
C	-3.02348900	-1.67986600	-0.69518100
C	-3.09563800	1.64292900	-0.90930100
C	-1.26587000	0.12976600	-1.16784400
H	-1.08198700	-0.58398400	-1.97422100
C	0.05938200	0.48667400	-0.49339300
C	-3.95263400	-1.58538500	-1.74053000
H	-4.18874600	-0.61028000	-2.15684400
C	1.33294800	2.37068600	0.94314300
C	-4.04974700	-0.12954000	2.23849300

C	-4.56989200	-2.73001300	-2.24357400
H	-5.28928600	-2.64372900	-3.05318500
C	-2.31809000	-0.44514000	-0.16052600
H	-1.83261800	-0.70852300	0.78086500
C	3.33742600	3.22632400	1.50026300
H	4.27676100	3.28617300	2.02400200
C	3.52151300	0.46721200	-1.77125800
H	4.38742600	0.21238000	-1.16103400
H	3.37187000	-0.29210800	-2.54075500
H	3.65674000	1.44285600	-2.24800400
C	-2.72999700	-2.93898500	-0.15909500
H	-2.01196600	-3.01472000	0.65268200
C	-4.40287900	0.52110700	0.91450400
H	-4.81977900	1.51731000	1.10085000
H	-5.18549700	-0.06742200	0.40988500
C	-4.26480300	-3.98505600	-1.70958500
H	-4.74591700	-4.87630500	-2.10229800
C	0.63916400	4.05773900	-0.78836900
H	-0.19391100	3.35839000	-0.83406600
C	2.79753000	4.03576300	0.55307000
H	3.17419000	4.93752200	0.09989900
C	-3.88641100	2.76866000	-1.16609300
C	-3.00674200	0.39028700	3.01680600
H	-2.44278000	1.24016700	2.64493700
C	-3.34245200	-4.08660200	-0.66723200
H	-3.10278800	-5.05763300	-0.24323700
C	2.66083000	1.05539600	2.62422700
C	-2.66357000	-0.21096400	4.22588100
H	-1.84449500	0.19290000	4.81416200
C	-3.36172100	-1.33562300	4.67534300
H	-3.09106800	-1.80595400	5.61635300
C	-4.73469700	-1.26152600	2.68619100
H	-5.52469100	-1.68622200	2.07231700
C	-4.39796000	-1.85981000	3.90288100
H	-4.93632200	-2.74171100	4.23819500
B	0.02343000	1.44696600	0.84306400
H	-0.09291100	0.78991500	1.85819200
H	-0.93862500	2.18157200	0.77216900
C	-1.61931800	2.20571200	-2.75697800
C	-2.40323300	3.34266100	-3.01747300
C	-3.51733000	3.61341500	-2.22046000
H	-0.74580600	1.97240100	-3.35507100
H	-2.15151500	3.99843100	-3.84553900
H	-4.12641000	4.48952000	-2.42727700

H	-4.76777700	2.98642800	-0.57224300
H	0.43406900	-0.73660300	0.22488600
S	0.94892400	-2.00024900	0.92225200
C	1.64778600	-2.64929000	-0.58357700
C	3.03583800	-2.83957400	-0.78064200
C	0.78157400	-2.88735400	-1.66470900
C	3.51232300	-3.22550600	-2.04335700
C	4.01754000	-2.67686400	0.32811700
C	1.26874700	-3.26864800	-2.91185900
H	-0.28590100	-2.76791800	-1.51580500
C	2.64031700	-3.43837800	-3.10665700
H	4.58014800	-3.35606400	-2.17773800
O	3.83690400	-2.95889200	1.49364700
O	5.20314200	-2.18258700	-0.13120600
H	0.57336300	-3.43347200	-3.72936500
H	3.02732300	-3.73799900	-4.07551400
C	6.26053800	-2.13308900	0.83999700
H	6.03726300	-1.39935200	1.61778400
H	6.40026400	-3.11002700	1.30982900
H	7.15353800	-1.84004600	0.28742800
H	1.81147200	0.39393400	2.46021200
C	0.10730700	5.41454800	-0.32520900
H	0.90330200	6.16600600	-0.27425000
H	-0.65028200	5.76394100	-1.03198400
H	-0.35675300	5.32913800	0.66102400
C	1.30756900	4.11147500	-2.16322100
H	2.16760600	4.79029800	-2.17073500
H	1.63223900	3.11762700	-2.48026600
H	0.58126600	4.47483900	-2.89487200
C	3.92962700	0.31234700	2.20984100
H	3.99681000	-0.62843800	2.76031300
H	3.88430200	0.07073200	1.14758800
H	4.83117200	0.90361200	2.40966200
C	2.67504900	1.51450900	4.08262600
H	3.50046600	2.20905200	4.27580900
H	1.73633600	2.01169200	4.34245000
H	2.80271200	0.64910500	4.73909500

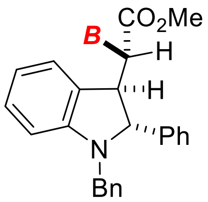

3a-i-Pr

O	2.59451800	-0.51206200	2.28508600
O	1.83939000	-2.59934000	1.87668800
N	-2.77895800	-1.30360300	-0.26416000
N	2.26148200	2.12559300	-0.31638200
N	3.79131300	0.61241600	-0.48248300
C	1.70482600	-1.39813300	1.72907900
C	-0.82100800	-2.53200400	-0.23290800
C	-2.49655000	0.08282100	1.77391400
C	-2.06739300	-2.35794500	-0.85784300
C	-0.65743600	-1.48755800	0.85327600
C	0.00906700	-3.57834100	-0.60413300
H	0.95815700	-3.70653200	-0.09670800
C	0.66682000	-0.69312200	0.92618000
H	0.45429700	0.24815300	1.45087900
C	-3.27630600	-0.70633500	2.62979800
H	-3.42743800	-1.75290800	2.38243500
C	2.44461600	0.78767100	-0.45543900
C	-3.21696500	0.59656400	-1.82389800
C	-3.85773900	-0.15350400	3.76910100
H	-4.46035900	-0.77603900	4.42462000
C	-1.83806900	-0.51611600	0.55248000
H	-1.43794200	0.30376800	-0.05952800
C	4.44242700	1.83186900	-0.37472800
H	5.51476000	1.92357200	-0.37905200
C	3.63495200	-1.11345200	3.06351800
H	4.27611300	-0.29296900	3.39021300
H	4.20648500	-1.83406100	2.47156000
H	3.22112700	-1.63820700	3.92955700
C	-2.32739600	1.43763400	2.07472200
H	-1.75125000	2.05930200	1.39591100
C	-3.79070100	-0.53996400	-0.99045600
H	-4.49430500	-0.13113600	-0.25663500
H	-4.35716700	-1.23393800	-1.61748800
C	-3.66906700	1.19916100	4.07024500
H	-4.12250800	1.62852000	4.95906100
C	0.94763700	2.78928000	-0.19351000
H	0.21540100	1.99368400	-0.33439200
C	3.48071400	2.78253600	-0.26393300
H	3.55982000	3.85096300	-0.15257400
C	-3.44448400	1.92836700	-1.45904500
H	-4.07267100	2.14365600	-0.59834200
C	-2.90327700	1.99520200	3.21861800
H	-2.75971800	3.04955600	3.43901500

C	4.45188600	-0.70326100	-0.64773100
H	3.77728900	-1.42367700	-0.18347300
C	-2.86217300	2.97633200	-2.17615500
H	-3.05622300	4.00522700	-1.88458700
C	-2.03355000	2.70019800	-3.26472600
H	-1.57939600	3.51234800	-3.82526000
C	-2.37514900	0.32964000	-2.91306800
H	-2.16365400	-0.69997300	-3.18388400
C	-1.78792100	1.37306500	-3.62720300
H	-1.13528800	1.15000900	-4.46622400
C	0.79685300	3.38789400	1.20761300
H	0.91393200	2.61774900	1.97442800
H	-0.19349500	3.83916300	1.31535300
H	1.54188600	4.17101100	1.38543000
C	0.76138600	3.82020600	-1.30775100
H	1.47469700	4.64683900	-1.21842300
H	-0.24936000	4.23206200	-1.25491300
H	0.88123400	3.35198300	-2.28762200
C	5.79441200	-0.73742500	0.08327100
H	6.54858000	-0.11865700	-0.41467300
H	6.16942700	-1.76424400	0.08786900
H	5.69398300	-0.40374500	1.11892400
B	1.30044500	-0.33726200	-0.58254900
C	4.58694100	-1.03424600	-2.13571900
H	3.60351800	-1.04547900	-2.61020800
H	5.04367700	-2.02077400	-2.25986200
H	5.21912500	-0.29520900	-2.64012200
H	1.77107200	-1.32441700	-1.09560600
H	0.42444800	0.10905400	-1.29629500
C	-2.47779100	-3.21072400	-1.88536700
H	-3.43439100	-3.07968700	-2.38163000
C	-1.62111400	-4.25088500	-2.26667300
H	-1.92647300	-4.91818000	-3.06838000
C	-0.39170000	-4.44105000	-1.63491700
H	0.25755000	-5.25502500	-1.94344800
H	-0.83578900	-1.97131500	1.82415300

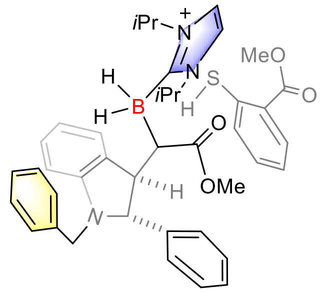

Int-III-A-cpx-2

O	-1.23702200	-0.29473400	-1.90371200
O	-1.37240100	1.94019100	-1.57989600
N	1.89186400	-2.29780400	0.65964200
N	-0.69477900	4.19888700	0.26032700
N	1.38650700	3.67033800	0.05174200
C	-1.03837200	0.82848400	-1.14981200
C	0.25907100	-1.22250900	1.87838900
C	1.24172000	-1.80864800	-1.66469800
C	1.36369700	-2.08823300	1.93750300
C	-0.01091400	-0.85288600	0.43187100
H	-0.78546400	-1.53108900	0.05435400
C	-0.39319100	0.57478100	0.12555800
C	0.56545900	-3.00912300	-1.91456300
H	0.13015700	-3.54737100	-1.08011600
C	0.18687700	3.19278800	0.47119900
C	4.26485300	-1.55159800	0.40148400
C	0.44304100	-3.49709400	-3.21292800
H	-0.09289900	-4.42516100	-3.39316400
C	1.34579100	-1.27474600	-0.25408700
H	2.00638000	-0.39356700	-0.25465600
C	1.25201400	4.96455400	-0.42815400
H	2.08182700	5.52913600	-0.81798600
C	-1.60849500	-0.09251400	-3.27240500
H	-1.48282300	-1.06517700	-3.74775100
H	-2.64714600	0.23288100	-3.35101400
H	-0.95233500	0.64630100	-3.74019600
C	1.82356600	-1.12733900	-2.73646400
H	2.37462200	-0.21189500	-2.54824200
C	3.27321900	-2.70531900	0.43433600
H	3.54696100	-3.42401300	1.21220400
H	3.31400800	-3.24292100	-0.51908900
C	1.00872400	-2.79745400	-4.28433100
H	0.91338100	-3.17820400	-5.29717700
C	-2.14728900	4.09523300	0.51398400
H	-2.34494000	3.02489400	0.55684800

C	-0.05591700	5.29740900	-0.28958200
H	-0.57333400	6.20882800	-0.53544100
C	1.76763400	-2.65223700	3.15086000
C	4.46300500	-0.74359600	1.53100900
H	3.91292900	-0.95543600	2.44265200
C	1.70409400	-1.61316900	-4.04114700
H	2.15726800	-1.06779500	-4.86432500
C	2.62862200	2.87426500	-0.00935700
H	2.44517800	2.01429700	0.63656900
C	5.35064600	0.33084500	1.48546200
H	5.49889000	0.94489800	2.36918900
C	6.05235500	0.61582500	0.30973500
H	6.75261300	1.44588700	0.27945400
C	4.95446500	-1.24439500	-0.77652500
H	4.78250100	-1.84630900	-1.66495300
C	5.84775500	-0.17127400	-0.82435000
H	6.38184900	0.04794700	-1.74465200
C	-2.93372700	4.68244200	-0.65810100
H	-2.62133300	4.20756200	-1.59020000
H	-4.00004000	4.49393900	-0.50631900
H	-2.80749600	5.76744500	-0.73953500
C	-2.49134800	4.73650100	1.85911300
H	-2.25430800	5.80634000	1.85772600
H	-3.56022200	4.62425800	2.06536700
H	-1.93131300	4.25355900	2.66392400
C	2.85617400	2.38546000	-1.44177200
H	3.07168200	3.22326000	-2.11438700
H	3.70165100	1.69176400	-1.46640000
H	1.96297300	1.87329700	-1.80840300
B	-0.08534600	1.76032900	1.15834600
C	3.81244200	3.66514400	0.54694900
H	3.60255300	4.02369700	1.55849500
H	4.68706100	3.01245900	0.58353700
H	4.06371100	4.52485700	-0.08365800
H	0.88340800	1.48853200	1.84081500
H	-1.03109100	1.89062900	1.92363900
C	-0.44580200	-0.91210000	3.02974900
H	-1.28295900	-0.22655400	2.97606000
C	-0.05065300	-1.46917300	4.25522600
C	1.04411100	-2.33284000	4.30662500
H	1.35321900	-2.75955800	5.25706200
H	-0.58856400	-1.21690100	5.16431400
H	2.62264200	-3.31855100	3.20763000
H	-3.86775400	-1.23270400	3.40471600

S	-4.04653900	-0.58073200	2.23717300
C	-3.40153800	-1.91426600	1.23093600
C	-3.52458100	-1.92626000	-0.17801300
C	-2.76242700	-2.99937100	1.85462400
C	-3.04895100	-3.02867700	-0.90445300
C	-4.09001500	-0.82107800	-1.00058800
C	-2.28158100	-4.07386200	1.11496300
H	-2.61274300	-2.98993100	2.92854600
C	-2.43546700	-4.10124100	-0.27302500
H	-3.16048600	-3.00075100	-1.98202800
O	-4.43152400	-0.93255800	-2.16339700
O	-4.15343300	0.34025700	-0.31978000
H	-1.77819000	-4.88681100	1.62926500
H	-2.06688000	-4.94047100	-0.85398400
C	-4.61985700	1.49068400	-1.04649100
H	-3.81201100	1.86337000	-1.67978400
H	-5.49336200	1.23725300	-1.65016600
H	-4.87378600	2.22827500	-0.28532000

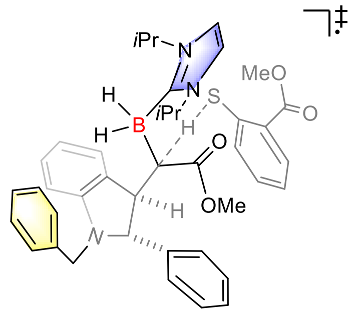

TS-A-minor

O	-1.47827300	-0.53134100	-1.32771600
O	-0.61040500	1.52415700	-1.63147900
N	2.22789400	-2.32352400	0.77975000
N	-0.58603500	3.90564300	0.36692200
N	1.42430100	3.55751600	-0.33544900
C	-0.77431700	0.54371100	-0.91292600
C	0.71535400	-1.30124700	2.18944200
C	1.17468100	-2.00836900	-1.43243200
C	1.88640600	-2.07614900	2.11397000
C	0.22179200	-1.01603100	0.78648100
H	-0.57327700	-1.73005700	0.54325100
C	-0.26259600	0.39610300	0.48017400
C	0.42708000	-3.18945700	-1.53271300
H	0.05882800	-3.66231100	-0.62742800
C	0.43766000	3.01823600	0.42381500
C	4.46204600	-1.43793100	0.07823800

C	0.14645400	-3.74575900	-2.77757100
H	-0.44413500	-4.65527800	-2.84153200
C	1.47302400	-1.39425900	-0.08230000
H	2.06256300	-0.47787300	-0.23372600
C	1.00435400	4.75533300	-0.89293500
H	1.63186700	5.34237100	-1.54189500
C	-1.92226500	-0.51688100	-2.69565600
H	-2.51055300	-1.42351700	-2.81579200
H	-2.54613700	0.35689200	-2.88847200
H	-1.05666900	-0.51816300	-3.36179500
C	1.65324000	-1.41091400	-2.60147800
H	2.24877400	-0.50625700	-2.52930000
C	3.58285500	-2.64980600	0.35508400
H	4.03576500	-3.28469200	1.12231500
H	3.51477100	-3.25782900	-0.55369300
C	0.62023800	-3.13606700	-3.94436900
H	0.40006800	-3.57094700	-4.91502700
C	-1.91104600	3.70390700	0.98400200
H	-1.81230400	2.78669100	1.56212600
C	-0.25857900	4.97414000	-0.45089600
H	-0.93968500	5.78662000	-0.64073600
C	2.51997900	-2.52755000	3.27397500
C	4.68679000	-0.47129300	1.06983600
H	4.23547600	-0.59419700	2.04938300
C	1.37740800	-1.96888800	-3.85355200
H	1.75480400	-1.49058800	-4.75297800
C	2.70992300	2.90392800	-0.65750100
H	2.77945100	2.05934600	0.02817200
C	5.47202200	0.64896600	0.80002300
H	5.63951100	1.38871900	1.57750100
C	6.04589700	0.81950900	-0.46379500
H	6.67204900	1.68361300	-0.66729500
C	5.01923500	-1.24472900	-1.19020700
H	4.82335700	-1.97236600	-1.97346800
C	5.81246200	-0.12728800	-1.46178500
H	6.24597300	0.00197500	-2.44951000
C	-2.97597400	3.50214600	-0.09719500
H	-2.65884500	2.73308900	-0.80386800
H	-3.91065600	3.18371700	0.37037200
H	-3.15833700	4.43098800	-0.64979000
C	-2.23821300	4.85055800	1.94223500
H	-2.33501700	5.80414800	1.41169900
H	-3.18992600	4.64847600	2.44180200
H	-1.46163000	4.95430700	2.70487400

C	2.68345200	2.37850600	-2.09539500
H	2.68096600	3.20290000	-2.81755800
H	3.57203100	1.76543600	-2.27452700
H	1.78477000	1.77992700	-2.25907600
B	0.47525700	1.63191800	1.24370100
C	3.87741000	3.85348800	-0.38656400
H	3.85767400	4.21352600	0.64598000
H	4.81580300	3.31940400	-0.54967600
H	3.86447400	4.71924500	-1.05753800
H	1.63315800	1.31315800	1.43957900
H	-0.02091800	1.82394800	2.33883400
C	0.16428900	-0.98278200	3.42105200
H	-0.73211800	-0.37485800	3.47147000
C	0.79098400	-1.43237300	4.59358700
C	1.95554500	-2.19518100	4.51219000
H	2.44332300	-2.53573600	5.42150000
H	0.37329800	-1.17626400	5.56219900
H	3.43050700	-3.11645600	3.22818300
H	-1.58250500	0.37216600	1.15505400
S	-2.99375200	0.25988300	1.75637000
C	-3.35908100	-1.34884800	1.07440500
C	-4.15326100	-1.54702300	-0.07862000
C	-2.74491100	-2.46886300	1.66183500
C	-4.25770700	-2.82718800	-0.63850300
C	-4.88291300	-0.46928500	-0.81405300
C	-2.87350000	-3.74010600	1.10590500
H	-2.13107700	-2.32424100	2.54415700
C	-3.62575000	-3.92171300	-0.05552400
H	-4.84268200	-2.94239100	-1.54496400
O	-4.97753400	-0.42878900	-2.02798000
O	-5.48680700	0.41755000	-0.00284200
H	-2.37707300	-4.58330000	1.57742500
H	-3.72259300	-4.90658500	-0.50196000
C	-6.18123000	1.48095500	-0.67337300
H	-5.49277500	2.05203800	-1.30168500
H	-6.98269700	1.08332700	-1.30168100
H	-6.58830500	2.10905700	0.11880600

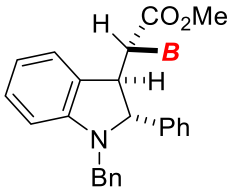

4a-i-Pr

O	-1.86672600	0.45288600	2.13353600
O	-1.25347000	-1.72020900	2.17134900
N	3.02863100	0.45584900	0.01611700
N	-3.31345600	0.73459400	-0.80449000
N	-3.59064500	-1.35453500	-0.32776200
C	-1.12145100	-0.60531000	1.69150600
C	1.91363500	-0.39956700	1.84112000
C	2.73689900	-1.67348400	-1.22952900
C	2.92485900	0.47456500	1.40479700
C	1.13280800	-0.90317200	0.64155500
H	0.98869000	-1.98574300	0.66286300
C	-0.25734700	-0.22914400	0.53734200
C	4.01598000	-2.09355400	-0.85091100
H	4.54596900	-1.53755500	-0.08441700
C	-2.64885400	-0.44190300	-0.68476800
C	2.27607900	2.56621400	-1.05961100
C	4.60535400	-3.20435400	-1.45761600
H	5.59960600	-3.52023900	-1.15316900
C	2.05442600	-0.49228400	-0.55749500
H	1.44894300	0.02228600	-1.30802900
C	-4.83839400	-0.75399700	-0.24940100
H	-5.73057600	-1.30225200	0.00144300
C	-2.83251400	0.15941900	3.15071700
H	-3.19408500	1.12525700	3.50601300
H	-3.66475200	-0.41815900	2.73623400
H	-2.38025800	-0.40669000	3.96814200
C	2.05760500	-2.38242100	-2.22914100
H	1.06054000	-2.06268300	-2.51862400
C	3.42336200	1.60765700	-0.77856200
H	4.23223100	2.12662200	-0.25514000
H	3.84338300	1.24327500	-1.72303500
C	3.92337000	-3.90703400	-2.45222500
H	4.38350500	-4.76965600	-2.92611100
C	-2.67650200	2.03968800	-1.08514500
H	-1.61322100	1.82463400	-1.19177900
C	-4.66250300	0.55953200	-0.54287300
H	-5.37013300	1.37054900	-0.58496700
C	3.70435900	1.18280200	2.32488900
C	1.71285600	3.31966300	-0.01822300
H	2.11975700	3.22972600	0.98459800
C	2.64595000	-3.49166500	-2.83608900
H	2.10901200	-4.03018500	-3.61248000
C	-3.33610000	-2.79909200	-0.10594000

H	-2.25592800	-2.87758500	0.00609200
C	0.63616900	4.17037200	-0.26151200
H	0.21557000	4.75455900	0.55199400
C	0.10289800	4.27835500	-1.55006000
H	-0.72395400	4.95631500	-1.74267100
C	1.72106500	2.66607600	-2.33965100
H	2.13876400	2.07273700	-3.14910300
C	0.64109500	3.51717900	-2.58756800
H	0.22531300	3.58655900	-3.58872400
C	-2.87738400	2.98405300	0.10214800
H	-2.48955800	2.52545600	1.01386600
H	-2.33663700	3.91525700	-0.07992600
H	-3.93602700	3.22811200	0.24551700
C	-3.19750200	2.60827300	-2.40585300
H	-4.26763300	2.83703100	-2.35259900
H	-2.66513700	3.53435100	-2.63801000
H	-3.03247000	1.90128300	-3.22340300
C	-3.98454600	-3.27430900	1.19397000
H	-5.07717700	-3.19847200	1.16883100
H	-3.73250300	-4.32706500	1.34916800
H	-3.59118000	-2.70696600	2.03922300
B	-1.06789900	-0.68396500	-0.86385900
C	-3.78577300	-3.59128600	-1.33515700
H	-3.26708000	-3.23684600	-2.23001200
H	-3.55601600	-4.65211100	-1.19964200
H	-4.86542800	-3.49445100	-1.49579600
H	-0.87562600	-1.85951500	-1.08619300
H	-0.68753100	-0.01671000	-1.80652000
C	1.70804900	-0.61185500	3.19707800
H	0.92910700	-1.29383400	3.52340800
C	2.49659700	0.07865800	4.13024800
C	3.47312500	0.97372500	3.68982200
H	4.07695500	1.51263100	4.41509600
H	2.34298700	-0.07894000	5.19346400
H	4.48162200	1.86649500	1.99727300
H	-0.12570300	0.85878300	0.55310700

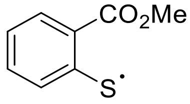

RS• E

C	0.52013900	-1.75283200	0.00000900
C	0.11483400	-0.40795000	0.00003100

C	1.07365700	0.63378300	-0.00002000
C	2.43882700	0.28282400	-0.00008700
C	2.82192500	-1.05228800	-0.00010700
C	1.86761400	-2.07957600	-0.00005900
H	-0.24063400	-2.52528100	0.00004800
H	3.19247300	1.06359700	-0.00012200
H	3.88011900	-1.29793300	-0.00016000
H	2.17969300	-3.11858600	-0.00007400
S	0.59630800	2.31942100	0.00001300
C	-1.29854000	-0.01459400	0.00010800
O	-1.67152300	1.15795200	0.00002800
O	-2.16748000	-1.04021300	0.00005800
C	-3.56016400	-0.67385400	0.00002100
H	-3.80111700	-0.08600800	-0.88917700
H	-4.10801600	-1.61547700	-0.00001400
H	-3.80117200	-0.08604200	0.88922600

7.4 Cartesian coordinates for all optimized geometries in the computational studies of HAT controlled by NHC-BH_3/Lewis acid

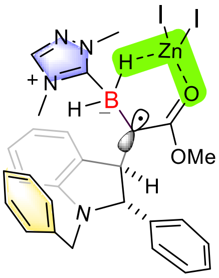

Int-III-B-1

O	-0.35652900	1.23183800	-3.10280400
O	-1.92158900	0.86023500	-1.55166000
N	3.68207400	0.65134500	-0.65884500
N	-0.36984000	-3.57688300	-0.76981200
N	1.13560900	-3.27139700	0.70356700
C	-0.76817400	0.62401900	-1.98253400
C	2.62085600	-1.17667000	-1.59968200
C	2.01972900	2.45850300	-1.18191200
C	3.79156900	-0.70360900	-0.97365500
C	1.63794800	-0.03423500	-1.73638200
H	1.70696300	0.36809600	-2.75267700
C	0.19935200	-0.25420400	-1.35238200
C	2.81323600	3.06813700	-2.16102500
H	3.66086800	2.52344000	-2.56692600
C	0.20366100	-2.61369400	-0.03318400
C	3.96798700	0.77989200	1.82178600
C	2.51554300	4.35692300	-2.60438600
H	3.13793300	4.82438700	-3.36242200
C	2.25969900	1.03361600	-0.75125200
H	1.78143300	0.87493800	0.22144900
C	1.09053000	-4.59298200	0.34470600
H	1.73758000	-5.34793000	0.76384000
C	-1.21539300	2.25654100	-3.63971500
H	-0.71519200	2.60310900	-4.54315800
H	-2.20403400	1.85223700	-3.86757300
H	-1.30739100	3.07124900	-2.91737000
C	0.93197100	3.15855300	-0.64517700
H	0.31261300	2.69732900	0.12019500
C	4.44386300	1.24023300	0.45499900
H	5.50079200	0.99990400	0.30763900
H	4.34619700	2.32596200	0.36076700
C	1.41923000	5.04664600	-2.07749500
H	1.18864000	6.04954400	-2.42545300
C	-1.46376100	-3.39735000	-1.72116500
H	-1.14734600	-2.71636600	-2.51481500
C	4.88349200	-1.55836800	-0.78441100

C	4.56805100	-0.30973300	2.46806300
H	5.44130600	-0.78095100	2.02676500
C	0.62829900	4.44528500	-1.09581200
H	-0.22022700	4.97425700	-0.67130500
C	2.11508300	-2.66454300	1.60613500
H	1.68861500	-1.77042500	2.05586300
C	4.05788100	-0.79318800	3.67494300
H	4.54259400	-1.63092900	4.16910700
C	2.92841500	-0.20086600	4.24611700
H	2.52451800	-0.58200500	5.17952300
C	2.85540600	1.38733400	2.42390600
H	2.39424800	2.25128100	1.95272700
C	2.32852300	0.89387700	3.61869000
H	1.45254800	1.36488900	4.05412600
B	-0.20101400	-1.06642900	-0.02900200
H	-1.41939800	-1.12310100	0.12761500
H	0.28170100	-0.52668300	0.93910800
C	2.55058400	-2.47776000	-2.07417200
H	1.65499500	-2.82582700	-2.58126100
C	3.63199900	-3.34812500	-1.87206200
C	4.78333500	-2.88145600	-1.23256500
H	5.62410700	-3.55281400	-1.08147100
H	3.57640800	-4.37426500	-2.22107300
H	5.79263300	-1.21144100	-0.30642600
H	-1.69896400	-4.37623900	-2.13468400
H	-2.33548400	-2.97707200	-1.21385300
H	3.01070800	-2.39467700	1.04605900
H	2.36317000	-3.38231300	2.38893000
N	0.17039900	-4.81432800	-0.55530500
Zn	-2.69430300	0.26934600	0.25513500
I	-4.72709300	-1.21450600	-0.14068400
I	-1.98488100	1.93016200	2.05189000

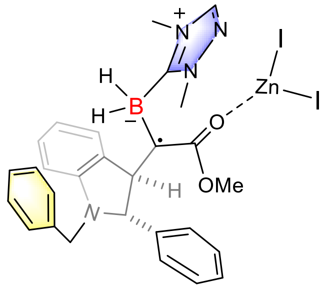

Int-III-B-2

C	-4.61468900	3.87856800	-1.81226300
C	-3.42192200	3.15337400	-1.94334600
C	-4.53798600	1.19688200	-1.04549900
C	-5.73294300	1.90863700	-0.91197500
C	-5.75309200	3.25412500	-1.29936500
H	-4.65046900	4.92371500	-2.10314700
H	-2.52435000	3.62986100	-2.32578200
H	-6.62892500	1.43961400	-0.51809200

H	-6.67565200	3.81836400	-1.19395600
N	-4.29488000	-0.15846600	-0.78781700
C	-2.84006100	-0.38372000	-0.78410100
H	-2.45845400	-0.26506500	0.23949400
C	-5.05420500	-0.90982200	0.21379200
H	-5.00817500	-1.96864800	-0.06184700
H	-6.10249000	-0.60827100	0.13959300
C	-2.44780200	-1.75109900	-1.28885800
C	-3.05226400	-2.29781200	-2.42757300
C	-1.42794200	-2.46352600	-0.65142000
C	-2.62873200	-3.52897700	-2.92729600
H	-3.85551300	-1.74894300	-2.91059400
C	-0.99365400	-3.69131200	-1.15437500
H	-0.95406700	-2.04963400	0.23361000
C	-1.59385700	-4.22663100	-2.29579400
H	-3.10438700	-3.94562700	-3.81091300
H	-0.18738100	-4.21934600	-0.65404900
H	-1.26313600	-5.18418900	-2.68805100
C	-4.53789900	-0.73116800	1.63309900
C	-3.84170100	-1.76653800	2.26825700
C	-4.67895900	0.49612300	2.29843900
C	-3.31533400	-1.59336600	3.55129600
H	-3.70333600	-2.71180700	1.74945200
C	-4.15254500	0.67302800	3.57819500
H	-5.19294100	1.31570000	1.80551100
C	-3.47457400	-0.37352500	4.21127500
H	-2.78306200	-2.40935900	4.03204900
H	-4.27311300	1.62723800	4.08310600
H	-3.07109700	-0.23656200	5.21063500
C	-3.38722900	1.82514000	-1.54963300
C	-2.26298500	0.81418500	-1.64045800
C	-0.92472300	1.21592000	-1.08402300
C	0.22484600	0.51819600	-1.58213000
C	0.41156100	2.11202900	1.07041400
N	0.50671000	1.30085900	2.13618200
C	2.37767500	2.26262000	2.07297000
H	3.39184200	2.57477900	2.26725500
N	1.61704300	2.74526800	1.03898800
C	-0.49423700	0.35988500	2.62579600
H	-1.47531100	0.67334600	2.27504400
H	-0.25941700	-0.64311500	2.26295100
H	-0.46884900	0.36734400	3.71461000
C	2.05992200	3.66807200	-0.00322300
H	2.20245600	3.11148700	-0.93039900
H	1.30589900	4.44239600	-0.14311800
H	3.00739500	4.11034100	0.30361200
O	1.40081700	0.71273500	-1.14268300
O	0.01007800	-0.35973900	-2.56891900
C	1.10087100	-1.19088900	-2.99729100
H	1.38888900	-1.87144600	-2.19075200

H	1.95617200	-0.58199200	-3.30291000
H	0.71198800	-1.76347500	-3.83783400
H	-2.16666200	0.46810000	-2.67610900
B	-0.84238500	2.30112400	0.08655600
H	-0.77139700	3.41148200	-0.41750300
H	-1.85706700	2.26341100	0.75038000
N	1.72100100	1.37358900	2.76855800
Zn	2.87617600	-0.23941100	-0.21301200
I	4.99257100	1.08476200	-0.52921500
I	2.16267000	-2.33978300	0.97275000

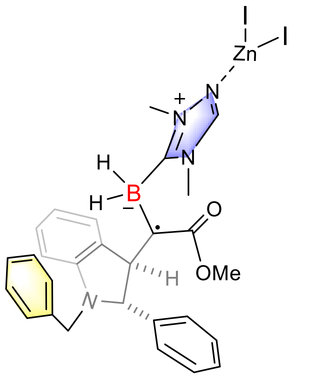

Int-III-B-3

C	-1.03659100	2.01562200	-2.81358600
C	-1.63325100	0.76811400	-2.56796100
C	-2.93536300	1.76191800	-0.77273300
C	-2.33191400	3.00613100	-0.99725300
C	-1.38161500	3.11345800	-2.02081900
H	-0.30473700	2.12463500	-3.60742400
H	-1.36913000	-0.10287000	-3.15924400
H	-2.59822500	3.87665300	-0.40691000
H	-0.91081800	4.07556800	-2.20236800
N	-3.94369900	1.43017100	0.11760300
C	-4.19955700	-0.02318000	0.11209200
H	-3.88699500	-0.44799300	1.07022300
C	-4.24469600	2.19348400	1.32340700
H	-5.27707200	1.96222000	1.60727000
H	-4.21633900	3.25826000	1.07225100
C	-5.66635100	-0.34635400	-0.09617300
C	-6.47697000	0.44685700	-0.91702100
C	-6.21795100	-1.48110100	0.51043800
C	-7.81374300	0.10833900	-1.12990100
H	-6.05122400	1.33413500	-1.37608700
C	-7.55368200	-1.82428600	0.29524600
H	-5.59328900	-2.09796000	1.15275200
C	-8.35585500	-1.02899700	-0.52592000
H	-8.43385200	0.73305200	-1.76698900
H	-7.96940700	-2.70694500	0.77339500
H	-9.39718500	-1.29089200	-0.69057100
C	-3.30041900	1.88198200	2.47061200

C	-3.63851300	0.91371400	3.42348500
C	-2.03650900	2.48518000	2.54155300
C	-2.72898700	0.53754100	4.41323200
H	-4.61840200	0.44426800	3.38096400
C	-1.12518600	2.11223400	3.53005000
H	-1.76261900	3.24258700	1.81311300
C	-1.46744200	1.13218600	4.46546900
H	-3.00467900	-0.22073100	5.14043500
H	-0.15097200	2.59191200	3.57529800
H	-0.75846500	0.84211200	5.23569600
C	-2.56140000	0.64749000	-1.54494600
C	-3.27414600	-0.59703600	-1.02072200
C	-2.23289000	-1.58813000	-0.55121700
C	-1.69611900	-2.51605200	-1.52975200
C	-0.00695800	-1.58591200	0.85667400
N	0.78368900	-0.54235500	0.57231800
C	2.12805300	-2.16999800	0.86768500
H	3.03160400	-2.75555400	0.95016400
N	0.86952100	-2.62038900	1.06224300
C	0.37074900	0.83674100	0.32074300
H	-0.63911300	0.95214100	0.70447400
H	1.05431700	1.50287300	0.84576600
H	0.39172900	1.04973300	-0.74747000
C	0.48341400	-4.00107600	1.36232200
H	-0.09976800	-4.01821200	2.28268700
H	-0.11621500	-4.36345800	0.52790800
H	1.39159300	-4.59335400	1.48038000
O	-0.77072000	-3.30953700	-1.32380200
O	-2.28803100	-2.44960900	-2.76130300
C	-1.77784600	-3.36330600	-3.73870500
H	-1.90816200	-4.39841500	-3.40951100
H	-0.71235500	-3.18960800	-3.91697600
H	-2.35227100	-3.17659800	-4.64671700
H	-3.88931800	-1.03886000	-1.80955600
B	-1.60981200	-1.59053700	0.93517100
H	-1.90803500	-0.60133900	1.56321500
H	-1.95020600	-2.58823300	1.54600700
N	2.11279400	-0.89136800	0.56314800
Zn	3.83571000	0.18324600	-0.04269000
I	5.76557100	-1.26370400	0.68619300
I	3.46030900	2.31811000	-1.28056000

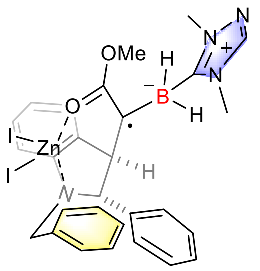

Int-III-B-4

C	1.43329100	-0.99959800	-4.74726200
C	0.38752800	-0.24336400	-4.20381100
C	1.59802200	-0.14330100	-2.11644000
C	2.64475300	-0.89744700	-2.63644500
C	2.54647900	-1.32084100	-3.96793000
H	1.37945100	-1.33518500	-5.77840100
H	-0.48400300	0.01230600	-4.79985400
H	3.49982000	-1.17579200	-2.03376800
H	3.35212100	-1.91121500	-4.39341700
N	1.45749000	0.34848600	-0.76000700
C	0.38172800	1.42331200	-0.83180000
H	-0.18702900	1.35945700	0.09537700
C	2.77613100	0.71926200	-0.13316900
H	3.31165600	1.38236600	-0.81942500
H	3.32942200	-0.22012000	-0.06671500
C	0.88534400	2.84223400	-0.99887300
C	1.73331900	3.21560300	-2.05219000
C	0.46051500	3.82443800	-0.09718400
C	2.16160500	4.53705400	-2.18084400
H	2.06293900	2.47078400	-2.76934300
C	0.87329100	5.14978300	-0.23365600
H	-0.16505800	3.53975800	0.74299800
C	1.73195400	5.50921600	-1.27340700
H	2.82823800	4.80856800	-2.99421000
H	0.53899100	5.89560300	0.48160700
H	2.06470900	6.53773300	-1.37741800
C	2.70233100	1.34533800	1.24083600
C	2.98912500	2.70655000	1.41153400
C	2.41731700	0.57044200	2.37246500
C	2.95352900	3.28840300	2.67839400
H	3.22976900	3.31619100	0.54612000
C	2.36948100	1.15166600	3.63966100
H	2.24729700	-0.49630600	2.27331400
C	2.63271600	2.51364100	3.79540800
H	3.17440900	4.34611700	2.79038100
H	2.13253500	0.53505300	4.50111600
H	2.60076700	2.96593000	4.78270300
C	0.48089900	0.18086600	-2.88512100
C	-0.50236400	1.00049900	-2.07924700
C	-1.81640000	0.33622500	-1.72953100
C	-1.79333500	-1.07075800	-1.41406800
C	-4.08727100	0.99226900	-0.47620800
N	-5.16704500	0.21791200	-0.28478800
C	-4.89146000	1.14691000	1.57882000
H	-4.98246900	1.45755900	2.60847800
N	-3.91973000	1.60652200	0.72997000
C	-5.83608900	-0.64342200	-1.24874100
H	-5.23834300	-0.67244800	-2.15518300
H	-5.92222600	-1.64382000	-0.82285100
H	-6.83311800	-0.24765200	-1.45442800

C	-2.86240500	2.55119500	1.07894700
H	-2.02079100	2.00777500	1.51450900
H	-2.54805500	3.07094600	0.17518900
H	-3.25430200	3.26940200	1.80146100
O	-0.77037900	-1.76359000	-1.19064300
O	-3.00074300	-1.65578000	-1.35956300
C	-3.01661900	-3.02288700	-0.89711200
H	-2.62077700	-3.07527200	0.12005100
H	-2.41773200	-3.65753700	-1.55367600
H	-4.06420500	-3.32254900	-0.92108000
H	-0.76149100	1.91681200	-2.61420100
B	-3.17063800	1.19947000	-1.78549400
H	-2.91017800	2.38453900	-1.86134200
H	-3.80957300	0.86887800	-2.77138900
N	-5.68113600	0.29838000	0.98203000
Zn	0.57704500	-1.39038700	0.30369000
I	-1.13070700	-0.75721500	2.18583100
I	2.38033900	-3.17858700	0.50743700

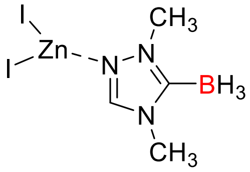

2d-ZnI_2 complex

C	-3.54918900	-0.95850300	-0.00008900
C	-2.26647300	0.85781700	-0.00024700
H	-1.94879900	1.89043300	-0.00052600
N	-1.43529400	-0.15882000	-0.00016800
N	-2.24453600	-1.27613200	-0.00013100
N	-3.54437700	0.41918300	-0.00017600
C	-1.66226800	-2.61421100	0.00030400
H	-2.49114300	-3.31915800	0.00056600
H	-1.04314300	-2.74323600	-0.88835700
H	-1.04303800	-2.74257200	0.88897400
C	-4.75736000	1.23476500	0.00004400
H	-5.34571400	0.99560800	0.88709800
H	-4.47467100	2.28775700	-0.00133600
H	-5.34719300	0.99367100	-0.88549000
B	-4.85876300	-1.85175900	0.00021500
H	-5.48413700	-1.51193400	-0.99126200
H	-4.58748100	-3.02882000	0.00031100
H	-5.48356100	-1.51168000	0.99200500
Zn	0.68389900	0.08369800	-0.00020600
I	1.03416700	2.57793000	0.00010400
I	2.07917100	-1.98493100	0.00001700

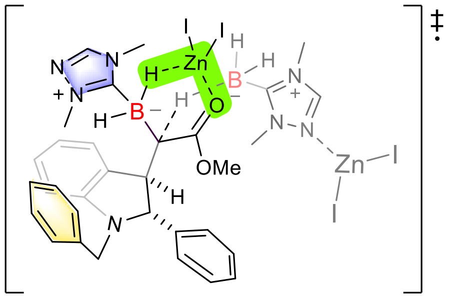

TS-B-major-1

O	-0.62319200	1.11758600	-0.48680700
O	1.45478800	1.87148200	-0.70565800
N	-0.50363600	-3.41080600	-0.39111400
N	3.63080200	-2.69398100	1.66910400
N	3.80044600	-0.94208800	2.86512900
C	0.69839900	0.98079800	-0.25976800
C	0.25541900	-2.43667500	1.55727700
C	-0.93482200	-1.44721100	-1.82548700
C	-0.13482000	-3.63793700	0.93179200
C	0.06647400	-1.29695400	0.57229000
H	-0.92652100	-0.86886400	0.74407900
C	1.09182800	-0.16334500	0.55982800
C	-2.32293600	-1.43523000	-1.62322300
H	-2.73744900	-1.92442200	-0.74743800
C	3.32761700	-1.38701200	1.66628500
C	0.90917900	-4.87361200	-1.84373600
C	-3.16808000	-0.81794500	-2.54391900
H	-4.24108000	-0.81754800	-2.37402400
C	-0.02547300	-2.07243800	-0.79277400
H	0.99084700	-2.14424100	-1.20507000
C	4.34029000	-2.01281500	3.52504000
H	4.79256000	-1.94605500	4.50289200
C	-1.00705700	2.13337000	-1.43673900
H	-2.07384800	1.99135100	-1.59697800
H	-0.79235300	3.13062500	-1.04448300
H	-0.46363700	1.99564900	-2.37108800
C	-0.41096300	-0.85761900	-2.97880400
H	0.66428500	-0.85922500	-3.14287300
C	-0.48737700	-4.47678300	-1.39915800
H	-1.02551500	-5.33639100	-0.98995400
H	-1.06920200	-4.11596700	-2.25268200
C	-2.63569100	-0.21554500	-3.69099200
H	-3.29491600	0.26492600	-4.40744000
C	3.32124200	-3.69643500	0.65799300
H	4.20932000	-4.30472500	0.49061100
C	-0.14429500	-4.84182800	1.64411700
C	1.59622600	-5.92740000	-1.22642200
H	1.09903800	-6.51862400	-0.46344700
C	-1.25858200	-0.24504700	-3.90992600
H	-0.83752900	0.21028000	-4.80170800
C	3.83386300	0.45251000	3.31376700

H	2.85816100	0.91138500	3.17611500
C	2.91370700	-6.22509900	-1.58124100
H	3.42999200	-7.04911700	-1.09671500
C	3.56569400	-5.46679900	-2.55657300
H	4.59227300	-5.69346000	-2.82883800
C	1.56572300	-4.13681300	-2.84075100
H	1.03354500	-3.33734700	-3.34901200
C	2.88660100	-4.42323600	-3.18992100
H	3.38179700	-3.83660800	-3.95802000
B	2.68509300	-0.48776900	0.50232200
H	3.33287900	0.55811800	0.63101300
H	2.96147000	-0.97825500	-0.56720300
C	0.64237800	-2.43934900	2.88996400
H	0.94633300	-1.51662800	3.36739300
C	0.66287300	-3.64593400	3.60600000
C	0.26267200	-4.82920500	2.98359200
H	0.27324000	-5.76205800	3.54001800
H	0.98993000	-3.65640500	4.64066800
H	-0.45279200	-5.76951600	1.17583800
H	2.50209300	-4.32089800	1.01201500
H	3.03524900	-3.19808000	-0.26513100
H	4.57100600	1.01500300	2.73901300
H	4.09732300	0.46675000	4.37200800
N	4.24481100	-3.10523100	2.81850000
H	0.79328000	0.52042100	1.90787100
B	0.31603300	1.18850400	3.07298000
C	-1.05287900	1.62275100	2.49135500
H	0.27571500	0.34393600	3.92229800
H	1.07916200	2.09855900	3.23041700
N	-1.31416700	2.80982200	1.83277300
N	-2.18280400	0.90129800	2.31188500
C	-2.53170300	2.72614800	1.26054300
C	-0.35168300	3.90520900	1.67627700
N	-3.09449600	1.56688800	1.52345000
C	-2.49521700	-0.41199200	2.86000300
H	-2.98734100	3.48184300	0.63836500
H	0.50964900	3.56242200	1.09868700
H	-0.84668900	4.72879100	1.16103200
H	-0.00753300	4.22882100	2.65815400
H	-3.37971300	-0.34006200	3.49563700
H	-2.69345500	-1.11591500	2.05171100
H	-1.63381300	-0.74709600	3.43304600
Zn	3.48008400	2.03664700	-0.49000300
I	3.77882400	3.85709200	1.29942400
I	4.69580300	1.41300000	-2.61951600
Zn	-4.61312400	0.76674500	0.27068200
I	-5.53491100	-1.49557400	0.80962400
I	-5.21445700	2.61880600	-1.33240300

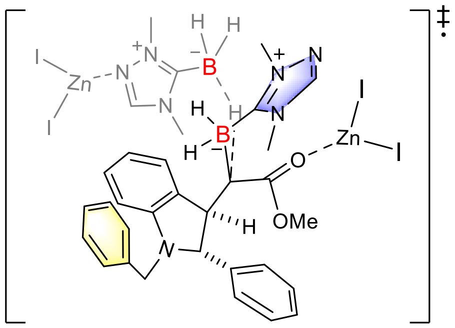

TS-B-major-2

O	-1.57739300	-0.66415800	-1.73173100
O	-2.90072300	-1.08472700	0.04167700
N	1.20365900	2.69779300	-0.34907100
N	-2.99476100	-1.56563000	2.82373600
N	-3.18059400	0.55402500	2.81732400
C	-1.78435100	-0.71462100	-0.40554100
C	1.71452700	0.65747700	0.57311900
C	-0.78349700	2.16255400	-1.72959200
C	2.13091100	1.99617700	0.40041100
C	0.43765000	0.43421900	-0.21148700
H	0.71900300	0.03934200	-1.19475700
C	-0.63283800	-0.44742400	0.43355500
C	-0.11331700	2.11130100	-2.95849400
H	0.95965800	1.93842600	-2.96840600
C	-2.29134800	-0.45142500	2.57458000
C	0.11149600	4.58349700	0.83999400
C	-0.81314600	2.29221500	-4.15110900
H	-0.28392200	2.25395900	-5.09933800
C	-0.04447300	1.90773600	-0.43720000
H	-0.69592200	2.17609100	0.40556100
C	-4.38092600	-0.01716400	3.15345500
H	-5.27280000	0.54715800	3.37694300
C	-2.68828600	-0.93741800	-2.60293900
H	-2.28516500	-0.87023700	-3.61260400
H	-3.09741200	-1.93254700	-2.40990800
H	-3.45954500	-0.17237500	-2.47683900
C	-2.15688300	2.42163900	-1.71476400
H	-2.68740900	2.46836000	-0.76876600
C	1.05324500	4.15295800	-0.27327000
H	2.04536900	4.59245600	-0.14532400
H	0.67228000	4.50060600	-1.23844100
C	-2.19170000	2.53096100	-4.12919500
H	-2.73526600	2.67229300	-5.05890100
C	-2.57761600	-2.93679000	2.56435100
H	-1.49196800	-2.95623200	2.48668400
C	3.33755900	2.44843500	0.95185300
C	0.47526900	4.41860000	2.18461000
H	1.45823900	4.02733300	2.42995700
C	-2.86302800	2.59795500	-2.90693100
H	-3.93302500	2.78040900	-2.87188700

C	-2.93772700	1.98511300	2.64349800
H	-3.11491500	2.26781700	1.60433500
C	-0.42009800	4.73906600	3.20505400
H	-0.12316200	4.61253900	4.24224500
C	-1.69431500	5.22581400	2.89500900
H	-2.38853500	5.48152700	3.69054000
C	-1.16881900	5.06203000	0.53944200
H	-1.46802700	5.16910000	-0.49971600
C	-2.06831800	5.38448100	1.55937400
H	-3.05678000	5.75936500	1.30927800
B	-0.77773700	-0.30998800	2.06505700
H	-0.37718700	0.78596600	2.39901300
H	-0.11096900	-1.15273700	2.63144300
C	2.45401100	-0.20319900	1.35382900
H	2.05675000	-1.17951000	1.59118800
C	3.69102100	0.22266800	1.89262600
C	4.11021300	1.55091700	1.68709700
H	5.05025600	1.88233900	2.11715700
H	4.23214500	-0.40838300	2.58871400
H	3.67610900	3.46799900	0.80914000
H	-2.92119100	-3.56944000	3.38321000
H	-3.02054800	-3.26953900	1.62266700
H	-1.91040700	2.21568600	2.91645800
H	-3.62454700	2.53994300	3.28337500
N	-4.29951200	-1.32091500	3.16124700
H	-0.03238400	-1.82233200	0.20846100
B	0.33431900	-3.13620600	-0.17263400
C	1.73640200	-2.81646700	-0.75951800
H	0.32394100	-3.74551600	0.86136900
H	-0.50616300	-3.39651300	-0.99069800
N	1.99814100	-2.24261400	-1.99187800
N	2.93993300	-2.77853800	-0.14107600
C	3.28569800	-1.82542600	-2.00952700
C	1.03099700	-2.09727400	-3.07789900
N	3.89295100	-2.12441900	-0.88628100
C	3.31975000	-3.41191800	1.11410100
H	3.74766800	-1.27003900	-2.81178100
H	0.19726500	-1.47899900	-2.74545700
H	1.53274800	-1.62957000	-3.92561600
H	0.65766500	-3.08235500	-3.36293100
H	3.87117600	-4.33220600	0.90662100
H	3.96354500	-2.73716500	1.67765100
H	2.41121600	-3.63530500	1.66969800
Zn	-4.87884400	-1.06768100	-0.16502700
I	-5.70392200	-3.40831700	-0.46732300
I	-5.70601600	1.32499300	-0.17955200
Zn	5.29983700	-0.62012600	0.01115600
I	6.97939800	-1.78965900	1.51124300
I	5.48426300	1.07337000	-1.89427100

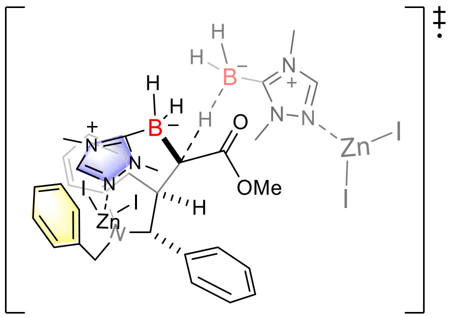

TS-B-major-3

O	1.76025900	-0.71754900	1.68724800
O	0.33767500	-1.01201600	3.40623400
N	-0.04894500	2.00138600	-1.39948700
N	-2.92533500	0.21267500	1.96628100
N	-3.02322000	2.26911800	1.38821400
C	0.74617700	-0.28106500	2.50718100
C	0.45693800	3.09673800	0.55772100
C	0.50733600	-0.34177900	-0.88403900
C	0.05223400	3.24464000	-0.78384400
C	0.68584400	1.62166100	0.84045300
H	1.74519700	1.41001000	0.65945300
C	0.31490800	1.09731200	2.22620300
C	1.76360900	-0.35117000	-1.50791700
H	2.29965900	0.58288700	-1.64473300
C	-2.30348800	1.38792100	2.16364200
C	-2.27555900	1.66204900	-2.54686500
C	2.31273000	-1.54452800	-1.97357100
H	3.28295700	-1.53617900	-2.46200300
C	-0.07812300	0.95109500	-0.36622800
H	-1.12175900	0.76181600	-0.06642200
C	-4.01329000	1.59129600	0.75797300
H	-4.71771400	2.01504800	0.05950600
C	2.05667800	-2.11778600	1.76799200
H	2.77758500	-2.31608300	0.97600800
H	2.46992000	-2.38187800	2.74632100
H	1.15315000	-2.70814600	1.60501500
C	-0.19962500	-1.53922600	-0.76847900
H	-1.18922600	-1.54162600	-0.32091600
C	-0.76023500	1.78157100	-2.65124700
H	-0.49880300	2.59764000	-3.33125100
H	-0.35984400	0.86572200	-3.09613800
C	1.60862100	-2.74723000	-1.82928600
H	2.04065300	-3.67529400	-2.19072000
C	-2.64335200	-1.07388400	2.59797900
H	-3.54751400	-1.41763000	3.10537700
C	-0.16076400	4.51366800	-1.33271200
C	-3.09196300	2.80347100	-2.50165000
H	-2.63434800	3.78780600	-2.50388900
C	0.34739200	-2.73983200	-1.23481100
H	-0.22107600	-3.66023700	-1.13574900

C	-2.78132600	3.70857900	1.23146200
H	-2.06192500	4.01408400	1.98549600
C	-4.48504200	2.69025500	-2.49159900
H	-5.09991900	3.58613600	-2.48226000
C	-5.08671900	1.42871000	-2.52578600
H	-6.16711500	1.33158000	-2.53055700
C	-2.88906500	0.40117900	-2.54502400
H	-2.27497200	-0.49508100	-2.56912200
C	-4.28284100	0.28478900	-2.54684100
H	-4.73858200	-0.70029500	-2.60240900
B	-0.98240700	1.66398800	3.04576900
H	-0.91807400	2.85614700	3.22936300
H	-1.12325400	1.07799200	4.09174700
C	0.65378600	4.21590800	1.35252800
H	0.95562100	4.09775500	2.38472600
C	0.42803300	5.49491900	0.81714200
C	0.03128800	5.63424200	-0.51301500
H	-0.13766900	6.62458200	-0.92675300
H	0.56438400	6.37291900	1.44063100
H	-0.45754100	4.63919300	-2.36853700
H	-2.35707400	-1.79592300	1.83248100
H	-1.82383600	-0.93612200	3.30111700
H	-3.72580000	4.23863500	1.36535900
H	-2.36669700	3.90447500	0.24190700
N	-3.97672000	0.32512100	1.09099000
H	1.45554300	1.76103200	3.09037000
B	2.56249100	2.24681300	3.76157300
C	3.59936200	1.33008100	3.06138200
H	2.64350100	3.40874400	3.47187000
H	2.36867000	1.97668200	4.91480200
N	3.98546400	0.06565900	3.47147000
N	4.16285000	1.44895000	1.83579500
C	4.68916600	-0.51714000	2.48140900
C	3.54197800	-0.57736200	4.70927200
N	4.81628400	0.29607900	1.45710200
C	4.12817900	2.60621400	0.95343100
H	5.07717800	-1.52448700	2.49301600
H	2.46869100	-0.77533300	4.64896300
H	4.09162300	-1.51182600	4.82877700
H	3.74348700	0.08718700	5.54901700
H	3.64744600	2.34271400	0.01079000
H	3.55387700	3.38365000	1.45252400
H	5.14631700	2.94449600	0.75179000
Zn	-5.13720800	-1.15318600	0.14184000
I	-3.88682900	-3.23153100	-0.50470100
I	-7.48761000	-0.26675600	0.09331600
Zn	5.25058500	-0.42765100	-0.46993600
I	5.38571200	1.19549600	-2.37341900
I	5.79631600	-2.87852800	-0.19173000

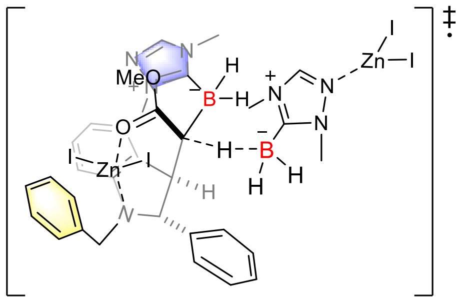

TS-B-major-4

O	-1.48902900	0.18442200	-0.23091400
O	0.54408600	-0.72985000	-0.12515200
N	3.36942800	0.36359200	0.51657300
N	-2.24303100	4.33117900	0.08136000
N	-1.00123900	3.29606800	-1.29882200
C	-0.21731200	0.19397800	0.24710700
C	2.21582300	2.41577800	0.68416300
C	3.26992200	0.37055900	3.09391900
C	3.21327500	1.71328800	0.00393200
C	1.49556100	1.50819500	1.65659100
H	1.39122100	1.99236500	2.62974700
C	0.05341500	1.23649100	1.20402200
C	3.82703400	1.56295300	3.58159600
H	3.70601400	2.48528800	3.02050700
C	-1.36087900	3.31423500	0.01862100
C	5.75765300	-0.04917100	-0.35869600
C	4.54686700	1.57120000	4.77552200
H	4.97517100	2.50052800	5.13998100
C	2.48530700	0.31378100	1.79803300
H	1.96278500	-0.63886100	1.77596200
C	-1.70101500	4.28861500	-1.94100500
H	-1.60662300	4.49305800	-2.99664700
C	-1.76004300	-0.78078000	-1.26548200
H	-2.77609000	-0.56733300	-1.60023600
H	-1.04984600	-0.68240100	-2.09048000
H	-1.68760700	-1.80142000	-0.88213000
C	3.44696200	-0.80753600	3.82818900
H	3.02705200	-1.73574000	3.45005800
C	4.79409700	-0.07352100	0.80415100
H	5.19005900	0.56072900	1.59887500
H	4.70142300	-1.08741600	1.19793900
C	4.71535800	0.38951200	5.50263800
H	5.27336200	0.39815400	6.43440400
C	-2.96547300	4.77215900	1.26488800
H	-2.25823900	4.97649200	2.07020800
C	3.86843000	2.28821000	-1.08722500
C	5.72966400	-1.01783200	-1.37025200
H	5.01617000	-1.83326500	-1.32488900
C	4.16225100	-0.79981500	5.02688700
H	4.28698500	-1.72241600	5.58616000
C	-0.07754000	2.39379100	-1.99164500

H	0.71356800	2.07696900	-1.32153900
C	6.62612200	-0.95622900	-2.43778200
H	6.57785800	-1.70784600	-3.21960500
C	7.57928200	0.06196500	-2.49690000
H	8.27721500	0.10817100	-3.32783800
C	6.73424500	0.95573100	-0.41670100
H	6.76983200	1.70857300	0.36643400
C	7.63868100	1.01429700	-1.47653500
H	8.38545000	1.80252300	-1.50724100
B	-1.00651300	2.45596000	1.34127100
H	-0.62046800	3.26217400	2.16975700
H	-2.09283700	2.04814700	1.69582400
C	1.95240800	3.75035500	0.37764500
H	1.19976400	4.29281700	0.93874000
C	2.65222300	4.35739800	-0.66833000
C	3.58080300	3.61858300	-1.40725700
H	4.09368800	4.07728000	-2.24716700
H	2.46122800	5.39701800	-0.91765300
H	4.56860300	1.72454300	-1.68416500
H	-3.51506800	5.67183000	0.99344900
H	-3.65455300	3.98657800	1.58194200
H	0.38264300	2.93108300	-2.82061800
H	-0.61078200	1.52493300	-2.37346500
N	-2.47093100	4.94342100	-1.11985400
H	-0.36018400	0.43063700	2.47406800
B	-0.71908600	-0.24432300	3.60057400
C	-1.90861800	-0.94866900	2.88625700
H	0.19969000	-0.96908400	3.86916900
H	-1.01402300	0.61469700	4.38490500
N	-1.83877900	-2.10977100	2.13693300
N	-3.14704300	-0.47720100	2.62478800
C	-2.99287600	-2.25370500	1.45386900
C	-0.65856900	-2.97371200	2.01936200
N	-3.81880100	-1.26370000	1.71872300
C	-3.82702300	0.65077800	3.24449800
H	-3.22023300	-3.05143600	0.76226900
H	-0.26059900	-3.17162900	3.01345400
H	-0.95632900	-3.90655700	1.54027200
H	0.09958900	-2.48024800	1.41029900
H	-4.65299500	0.27925000	3.85714400
H	-3.10034200	1.17620000	3.85895800
H	-4.19943900	1.31600300	2.46585000
Zn	2.43868200	-1.07192000	-0.82303300
I	2.27581700	-0.36275400	-3.27899100
I	2.86040100	-3.41162200	0.22680800
Zn	-5.07454800	-0.62475300	0.13493500
I	-5.14594800	1.82887400	-0.35600300
I	-5.83108200	-2.74026100	-0.99054700

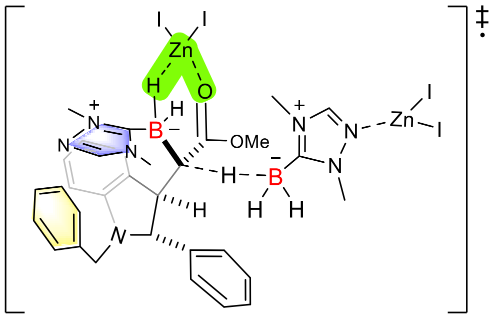

TS-B-minor-1

O	0.09652600	1.27809300	1.83831900
O	0.90168400	-0.27721200	0.43730900
N	-4.87640000	1.03432200	0.69194900
N	-3.91602600	-2.30649300	0.01754600
N	-3.97120800	-1.26269000	-1.83558100
C	-0.08082700	0.41079500	0.82869700
C	-3.13836700	0.40810000	2.07096900
C	-3.78056100	3.15457300	-0.04176100
C	-4.53131500	0.39245500	1.87573100
C	-2.47227300	1.17462300	0.93957400
C	-2.57165400	-0.18738100	3.18734100
H	-1.49566500	-0.18325000	3.32261600
C	-1.39449700	0.37237800	0.20943800
C	-3.97567600	3.92437600	1.11461100
H	-4.05887400	3.42600800	2.07732800
C	-3.16792100	-1.51626100	-0.76388900
C	-6.87618700	0.87309500	-0.79786600
C	-4.07235400	5.31205900	1.03341600
H	-4.22403100	5.89706800	1.93601000
C	-3.68505700	1.64258200	0.05261100
H	-3.58186500	1.25231900	-0.96349300
C	-5.16425700	-1.90220300	-1.62825600
H	-6.00134100	-1.83776700	-2.30570600
C	1.34235500	1.17066500	2.56993500
H	1.46919200	0.14939300	2.93571500
H	1.24835100	1.87505800	3.39531200
H	2.18961200	1.44044800	1.93891300
C	-3.69007200	3.80086500	-1.27739900
H	-3.51837800	3.21290100	-2.17472900
C	-6.22003300	1.52035500	0.40817800
H	-6.18602400	2.60540300	0.25453700
H	-6.83787600	1.35496600	1.29430100
C	-3.97548900	5.95074000	-0.20738400
H	-4.05054800	7.03237600	-0.27037100
C	-3.55469600	-2.92838500	1.28851900
H	-2.55898600	-2.60544900	1.57903000
C	-7.79362600	-0.17303600	-0.64749200
H	-8.02742400	-0.53944900	0.34845800
C	-3.78287300	5.19264000	-1.36222000
H	-3.70261100	5.68124500	-2.32896600

C	-3.60261600	-0.52228100	-3.04004100
H	-3.21904300	-1.21123300	-3.79715000
C	-8.40928100	-0.75068500	-1.76034600
H	-9.12554600	-1.55573400	-1.62459900
C	-8.10271400	-0.29409200	-3.04525600
H	-8.58456800	-0.73686200	-3.91209200
C	-6.58303700	1.33226800	-2.08968700
H	-5.89364200	2.16400600	-2.21289600
C	-7.18543100	0.74963200	-3.20726500
H	-6.95780200	1.12376400	-4.20190700
B	-1.65017600	-1.04339500	-0.57134900
H	-1.12919400	-1.05743000	-1.65763900
H	-1.15387000	-1.96839500	0.08567000
C	-5.36792900	-0.22997100	2.80863700
H	-6.44216000	-0.27469600	2.66321500
C	-4.78444400	-0.81306200	3.94289200
H	-5.42766600	-1.29282300	4.67540300
C	-3.40245300	-0.80231200	4.13695500
H	-2.96738500	-1.27149300	5.01309300
H	-2.00033200	2.06974700	1.35041800
H	-4.27375200	-2.61665700	2.04397600
H	-3.57404400	-4.01265100	1.16525600
H	-4.48951700	-0.01059800	-3.41424700
H	-2.83150500	0.20673300	-2.79865300
N	-5.15963200	-2.55270100	-0.49800000
H	-1.07159700	1.27544400	-0.95687600
B	-0.67432800	2.07617200	-2.04507800
C	0.81331000	1.62774900	-2.05173600
H	-1.32398200	1.68848700	-2.98082100
H	-0.85738600	3.19522600	-1.65846200
N	1.39714900	0.56123800	-2.69905800
N	1.79687400	2.10134800	-1.25783000
C	2.65361700	0.40328500	-2.22895900
C	0.74779800	-0.27598100	-3.70656000
N	2.93644800	1.33653500	-1.34727100
C	1.70484100	3.26333700	-0.38195300
H	3.33725300	-0.38009600	-2.51985300
H	0.04390700	-0.94804100	-3.21576600
H	0.22852800	0.37170600	-4.41355500
H	1.51124600	-0.86319700	-4.21442800
H	1.33229700	4.11010600	-0.95955500
H	1.01453900	3.05467000	0.43740100
H	2.69549100	3.47759700	0.01266300
Zn	0.69566600	-2.31247400	0.33339700
I	1.85916500	-3.28770600	-1.70299400
I	0.41143000	-3.05620100	2.76798500
Zn	4.75475800	1.30682900	-0.27036000
I	5.02553400	2.82643600	1.71059100
I	6.24712000	-0.30077200	-1.48955300

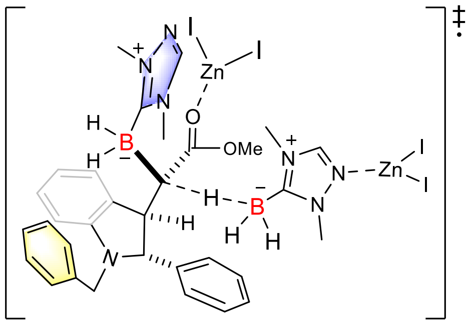

TS-B-minor-2

O	0.44470600	0.52888700	0.38284000
O	1.68078900	-1.14766800	-0.51155600
N	-3.65279000	-2.35777200	1.01423300
N	1.29460200	-4.15023400	0.79614000
N	1.66750500	-4.04195900	-1.29568900
C	0.55011000	-0.72547100	-0.11036000
C	-1.83891300	-1.25445000	1.89457900
C	-4.31103700	-0.32774400	-0.26094400
C	-2.89472000	-2.15193200	2.15246900
C	-1.88752400	-0.84023900	0.43136400
C	-0.93287700	-0.91924100	2.89017200
H	-0.13128400	-0.21845400	2.68469300
C	-0.68642500	-1.43774100	-0.31076200
C	-4.57596600	0.62212400	0.73679000
H	-4.03093800	0.57947900	1.67527600
C	0.73304900	-3.74072000	-0.35217300
C	-5.34785100	-3.55724900	-0.31649000
C	-5.51597400	1.63083500	0.52403200
H	-5.70019400	2.36799700	1.30006100
C	-3.26340100	-1.40736400	-0.04992600
H	-3.13758800	-1.95452600	-0.98630300
C	2.74624500	-4.60246300	-0.66261900
H	3.64463100	-4.91864900	-1.16963400
C	1.64275500	1.26890200	0.66113900
H	2.19545100	0.80980900	1.48365800
H	1.30955300	2.26400600	0.95603800
H	2.28678800	1.32584900	-0.22159500
C	-5.03229100	-0.27070100	-1.45870700
H	-4.85088800	-1.02087300	-2.22271500
C	-4.97826000	-2.94617000	1.02250400
H	-5.74444700	-2.20989100	1.31413300
H	-4.98607600	-3.73185300	1.78656600
C	-6.21254500	1.69637900	-0.68688000
H	-6.94266400	2.48329300	-0.85333200
C	0.74516300	-4.02551900	2.14345100
H	-0.32254000	-3.82854200	2.06631700
C	-6.61686200	-3.34523700	-0.86404200
H	-7.33018100	-2.71911400	-0.33406400
C	-5.97464000	0.73794600	-1.67477600
H	-6.52361400	0.77243900	-2.61176600

C	1.55405000	-3.72529900	-2.71667600
H	1.15374100	-2.71768700	-2.82014300
C	-6.96582300	-3.91231100	-2.09160200
H	-7.95492700	-3.73730400	-2.50563000
C	-6.04022500	-4.68891600	-2.79058300
H	-6.30582300	-5.12280700	-3.75046800
C	-4.42366900	-4.33944300	-1.02175200
H	-3.42647200	-4.47713500	-0.61490700
C	-4.76713900	-4.89756000	-2.25288500
H	-4.03732800	-5.49175700	-2.79559300
B	-0.69885300	-3.06416500	-0.57679200
H	-1.45042900	-3.57898700	0.21753400
H	-1.06269600	-3.32539000	-1.70337200
C	-3.03969400	-2.72184000	3.42339300
H	-3.85179600	-3.40572700	3.64597300
C	-2.11063600	-2.38375600	4.41539500
H	-2.21747200	-2.82114100	5.40436500
C	-1.06752400	-1.48894300	4.16587800
H	-0.36954500	-1.22645800	4.95430800
H	-1.88294200	0.24780500	0.33766800
H	0.93174900	-4.95714100	2.67816400
H	1.23467800	-3.19877300	2.66004500
H	2.54892500	-3.76932300	-3.15995600
H	0.88104300	-4.43063900	-3.20761500
N	2.54824600	-4.68126100	0.62708800
H	-0.91687400	-0.91705600	-1.68864000
B	-1.21082600	-0.41099500	-2.95731100
C	-0.64986000	1.01004000	-2.69438900
H	-0.53507600	-1.08959200	-3.68244600
H	-2.40276900	-0.49812200	-3.03382200
N	0.67696700	1.39974600	-2.78526100
N	-1.25498100	2.07900800	-2.12658500
C	0.80457800	2.62856500	-2.24145900
C	1.76679400	0.57557500	-3.31284200
N	-0.35733300	3.07146000	-1.81373400
C	-2.67832900	2.27650600	-1.88123500
H	1.72611600	3.18398100	-2.14662800
H	1.96393900	-0.25029800	-2.62794700
H	1.48155700	0.18746700	-4.29041400
H	2.66073800	1.19313600	-3.40130200
H	-2.91081500	2.13451800	-0.82624300
H	-2.94310800	3.29035700	-2.18598000
H	-3.22773300	1.54708300	-2.47062800
Zn	3.54093400	-1.47269100	0.12326700
I	4.91406100	-1.63535200	-1.99640800
I	3.88041200	-1.46572800	2.60810200
Zn	-0.36330000	4.33121200	-0.09404800
I	1.57302900	5.88656900	-0.45298100
I	-1.88535300	3.51919200	1.70536800

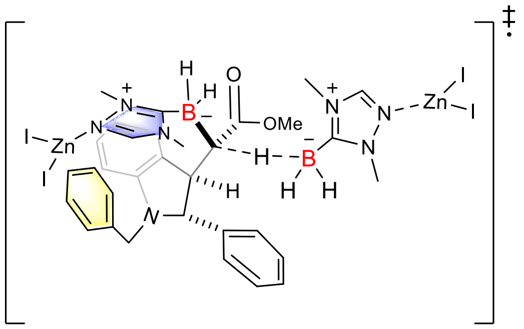

TS-B-minor-3

O	-2.77500500	-0.39819400	-1.44262100
O	-2.43585700	-2.60797500	-1.13170800
N	1.48830200	1.51091100	0.54973000
N	2.89757800	-1.26699800	-0.92820300
N	2.45790000	-1.85098800	1.07761700
C	-2.03065300	-1.46182500	-0.98174100
C	0.21080000	1.04200800	-1.31355900
C	-0.53533900	1.87598900	1.96609300
C	1.38062900	1.65442600	-0.83241700
C	-0.56877100	0.43498100	-0.15909300
C	-0.08765100	1.05773400	-2.66809200
H	-1.00029200	0.58840600	-3.02016700
C	-0.78242000	-1.07245100	-0.29978400
C	-1.15713500	2.99627000	1.39296600
H	-1.06651300	3.17114900	0.32437900
C	1.88674000	-1.70415900	-0.16361000
C	3.49545100	1.71220300	2.01138700
C	-1.89547000	3.87939700	2.17936000
H	-2.38118100	4.73446100	1.71862200
C	0.24913800	0.90206200	1.10175400
H	0.51214000	0.04088200	1.72082300
C	3.76572600	-1.51064100	1.00387100
H	4.46680500	-1.51372500	1.82366300
C	-3.90967500	-0.73434400	-2.25171100
H	-3.59056500	-1.19412900	-3.19186500
H	-4.42814600	0.20589700	-2.44225200
H	-4.57030700	-1.44032400	-1.74159900
C	-0.63770600	1.68568700	3.34816000
H	-0.16198800	0.82149200	3.80292000
C	2.31016300	2.40112800	1.36212700
H	1.68576900	2.85283400	2.14085800
H	2.66242100	3.22397900	0.73309000
C	-2.01269800	3.66459700	3.55693700
H	-2.59176300	4.35061100	4.16793000
C	2.90019100	-1.00618500	-2.36574500
H	1.88224600	-0.78867100	-2.67444900
C	3.33472600	0.99033800	3.20100900
H	2.35508600	0.95544700	3.67189600
C	-1.37428200	2.57026200	4.14158800
H	-1.45126900	2.40053500	5.21176200

C	1.78033700	-2.31270300	2.28955200
H	2.33327600	-3.15341400	2.71288500
C	4.42229400	0.34527800	3.80045500
H	4.28246700	-0.20169200	4.72891100
C	5.68947200	0.41867600	3.21600700
H	6.53745300	-0.08103500	3.67322300
C	4.77313500	1.79213500	1.44167000
H	4.92063400	2.35760300	0.52632100
C	5.86330400	1.15212800	2.03673800
H	6.84895000	1.23107400	1.58744900
B	0.40984800	-2.13885500	-0.63154000
H	0.21011500	-3.20656600	-0.08617100
H	0.48081200	-2.31570200	-1.83016000
C	2.27349900	2.27393800	-1.71222000
H	3.20172200	2.71340700	-1.36508900
C	1.95979600	2.28704000	-3.07785000
H	2.64991400	2.76405400	-3.76785700
C	0.79400800	1.68952200	-3.56063600
H	0.56835100	1.71271400	-4.62203500
H	-1.54958200	0.90892600	-0.11309100
H	3.53127600	-0.13980400	-2.55318100
H	3.28086500	-1.88506700	-2.89159800
H	1.73704900	-1.49602300	3.01219800
H	0.77314000	-2.62501400	2.02525000
N	4.06648100	-1.15276000	-0.21914100
H	-1.24209900	-1.50410100	1.15224300
B	-1.75285600	-1.88894400	2.37774200
C	-3.23358200	-1.90997900	1.92771300
H	-1.30446100	-2.98073000	2.61504500
H	-1.46373600	-1.00353300	3.13537600
N	-3.92301300	-2.97512600	1.37679300
N	-4.06633700	-0.86045400	1.73572300
C	-5.07106300	-2.51999000	0.84125900
C	-3.37230400	-4.31949800	1.20959700
N	-5.19891700	-1.22565300	1.03971100
C	-3.83783100	0.52576500	2.11440500
H	-5.79421800	-3.11480700	0.30359500
H	-2.56034200	-4.26114500	0.48245600
H	-3.00463700	-4.67744200	2.17088700
H	-4.16359600	-4.97356200	0.84174600
H	-3.10182000	0.54093200	2.91485000
H	-3.46248900	1.09466900	1.26279900
H	-4.77891600	0.96117600	2.44964600
Zn	-6.48652700	-0.04416400	-0.12386500
I	-6.01358800	2.40697200	-0.30088000
I	-8.14056900	-1.65416300	-1.13712900
Zn	5.99615900	-0.48621500	-0.72776800
I	7.55603100	-2.05669300	0.47764800
I	6.18887100	1.43548300	-2.32570500

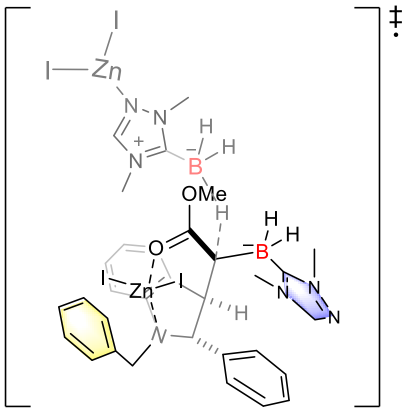

TS-B-minor-4

O	-0.83083000	-1.99843100	-0.03415600
O	0.18489500	-0.04333900	0.36772600
N	3.08384900	0.69806500	0.33741700
N	3.40561600	-4.67161900	0.28178000
N	1.89027100	-4.30764000	-1.16282400
C	0.17694900	-1.29269500	0.51491600
C	2.47572700	-0.04847700	2.48703000
C	4.59273400	-1.38349700	0.30063300
C	2.83491500	1.06220800	1.71782900
C	2.42902500	-1.30880300	1.65224300
C	2.30150000	0.08138700	3.86144700
H	2.07929500	-0.79202700	4.46227300
C	1.08739000	-2.02134600	1.36309900
C	5.46373400	-1.28559000	1.39915100
H	5.13061400	-0.79135200	2.30716900
C	2.15273900	-4.18710000	0.17392400
C	4.26751400	2.79015100	-0.62357100
C	6.75195300	-1.81345900	1.32950300
H	7.41707500	-1.72558000	2.18367800
C	3.17547100	-0.84565800	0.35196200
H	2.65909700	-1.18582400	-0.54498600
C	2.99125000	-4.85169700	-1.76849500
H	3.04924700	-5.05919300	-2.82646800
C	-1.66624100	-1.30615100	-0.98989900
H	-2.01297600	-0.35190500	-0.59313300
H	-2.50570700	-1.97324500	-1.18163800
H	-1.10257900	-1.11596500	-1.90630800
C	5.04106800	-2.02053200	-0.86037200
H	4.37613800	-2.09169800	-1.71685100
C	4.29767100	1.30717800	-0.33170600
H	4.39235100	0.76060800	-1.27223400
H	5.17582400	1.08036300	0.27739500
C	7.18435600	-2.46280500	0.16776800
H	8.18417600	-2.88363600	0.11876000
C	4.23865000	-4.77001800	1.47336300
H	3.62257600	-4.53701900	2.33887300
C	3.60333600	3.29409100	-1.74883800
H	3.08493700	2.61643500	-2.41967400

C	6.32445800	-2.56826300	-0.92535400
H	6.64676200	-3.07884900	-1.82733500
C	0.65464000	-3.92847700	-1.84741200
H	-0.17241200	-4.02710100	-1.14851100
C	3.60836100	4.66158300	-2.02530600
H	3.07207100	5.03405300	-2.89233000
C	4.29734600	5.54223200	-1.19120400
H	4.30154400	6.60670100	-1.40630200
C	4.98143100	3.68079600	0.19101700
H	5.52173200	3.29844600	1.05319900
C	4.99270500	5.04761100	-0.08458400
H	5.54643300	5.72442000	0.55971100
B	1.15534500	-3.65634100	1.34042800
H	1.60306300	-4.04175900	2.40021700
H	0.07256600	-4.15569200	1.12655100
C	2.86096400	2.34164600	2.27257800
H	3.03711200	3.21467300	1.66235700
C	2.64368800	2.46845900	3.64933700
H	2.67873900	3.45703200	4.09735800
C	2.39797600	1.34785000	4.44670200
H	2.26480200	1.46002600	5.51856400
H	3.03922100	-2.06716800	2.14727500
H	4.62451400	-5.78806000	1.54536800
H	5.06670100	-4.06452600	1.39012200
H	0.72172300	-2.89331500	-2.19045000
H	0.51254100	-4.59119200	-2.70273000
N	3.94007100	-5.08989500	-0.90744800
H	0.24754600	-1.81427600	2.61334100
B	-0.69398200	-1.64334900	3.65644000
C	-1.77219300	-0.80385700	2.90853300
H	-1.01352800	-2.77565000	3.88795300
H	-0.12613700	-1.03000300	4.51351000
N	-1.78595200	0.55871000	2.68060000
N	-2.85203100	-1.24998800	2.23006000
C	-2.82346400	0.85633200	1.87396100
C	-0.74937600	1.50056500	3.10638900
N	-3.50549600	-0.23018700	1.57764700
C	-3.26858300	-2.63642900	2.07076300
H	-3.06468400	1.84126500	1.50129900
H	0.12129300	1.37750200	2.46372900
H	-1.13436100	2.51374000	3.00073100
H	-0.48281400	1.29002000	4.13967400
H	-2.94608100	-3.18383100	2.95431500
H	-4.35140800	-2.67042900	1.96537000
H	-2.78963700	-3.05971700	1.18567300
Zn	1.26732900	1.22815900	-0.78030700
I	1.62016500	-0.01525800	-3.06236100
I	0.07855200	3.42799500	-0.26186600
Zn	-5.05224800	-0.24060000	0.13864600
I	-5.72625500	2.16062100	-0.12800900

I	-5.68302000	-2.44045200	-0.88628500

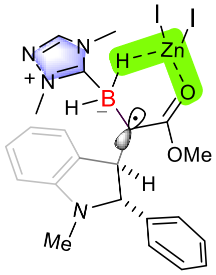

Int-III-B-Me

O	-0.68165300	-1.28895200	-2.54345700
O	1.20812200	-0.75525300	-1.47859900
N	-4.16683800	-0.78378700	0.58763500
N	-1.25029000	3.40809900	1.20958100
N	-0.08009200	3.48452300	-0.56636700
C	-0.03550500	-0.62425900	-1.57709900
C	-3.29874300	1.07993000	-0.46158600
C	-2.53963000	-2.54235500	-0.17987900
C	-4.33374100	0.57912400	0.35345700
C	-2.34657000	-0.05181800	-0.78455200
H	-2.61933900	-0.46617800	-1.76118100
C	-0.86609100	0.19611400	-0.71618900
C	-3.45455700	-3.17644400	-1.02889600
H	-4.38320800	-2.67001400	-1.27603300
C	-0.53518700	2.62755900	0.38782100
C	-3.17120700	-4.43960500	-1.54940500
H	-3.88880700	-4.92708900	-2.20362800
C	-2.76470600	-1.12976600	0.29974000
H	-2.15507800	-0.95299400	1.19937100
C	-0.56279900	4.73215700	-0.27233500
H	-0.35113700	5.61124500	-0.86191500
C	0.07477000	-2.27157500	-3.27716700
H	-0.62516100	-2.68629300	-4.00130800
H	0.92983200	-1.80907300	-3.77503600
H	0.42189800	-3.04983500	-2.59349600
C	-1.34267300	-3.19058700	0.14666600
H	-0.62500400	-2.71032500	0.80647800
C	-4.77745300	-1.41759400	1.74196900
H	-5.85960100	-1.26262900	1.72624400
H	-4.59056600	-2.49255600	1.69381700
C	-1.96724700	-5.07658100	-1.23400700
H	-1.74725400	-6.05865400	-1.64302300
C	-2.06651700	2.98685800	2.34335400
H	-2.16711600	3.84097000	3.01147200
C	-5.36316400	1.41916200	0.78975500
C	-1.05309400	-4.44926100	-0.38405200
H	-0.11685500	-4.93508000	-0.12443100
C	0.76170700	3.10680900	-1.70234400
H	0.24592800	2.35192700	-2.29908300

B	-0.22364200	1.05731000	0.47571100
H	1.00783200	1.02831900	0.47544800
H	-0.60782900	0.63408700	1.54096100
C	-3.33668000	2.38812800	-0.91796300
H	-2.56130700	2.75697300	-1.58385100
C	-4.36955100	3.23958600	-0.49619100
C	-5.36290900	2.75191100	0.35601100
H	-6.15467800	3.41540900	0.69214300
H	-4.39064400	4.27337400	-0.82487500
H	-6.14727400	1.05425300	1.44445900
H	-3.04731700	2.66884700	1.98275900
H	-1.56854800	2.16117400	2.84827100
H	1.71090400	2.70053300	-1.34750000
H	0.94570300	3.99344000	-2.30955500
N	-1.29081400	4.71573600	0.81107800
Zn	2.38295500	-0.25172200	0.12009100
I	4.24197200	1.33016400	-0.61455500
I	2.20947800	-2.06631200	1.88563900
H	-4.37890000	-1.03220000	2.69504600

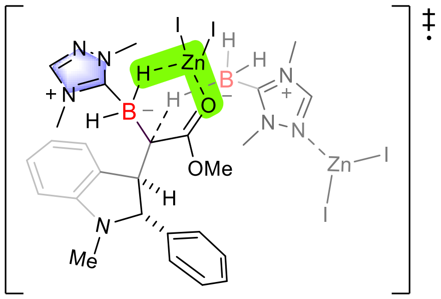

TS-B-major-Me

O	-0.38786000	-0.94369500	0.37406200
O	1.75210200	-1.54343200	0.44552700
N	-0.71445200	3.34403100	1.82687700
N	3.76038600	2.64537600	-1.78498800
N	3.50944200	3.73351700	0.02645200
C	0.91858200	-0.62368500	0.28758100
C	0.20311100	3.16247800	-0.28022400
C	-1.05707600	0.97834500	2.45393500
C	-0.28718900	4.04333600	0.70527400
C	0.07962600	1.74018800	0.23904700
H	-0.85674300	1.31985800	-0.14120300
C	1.21455900	0.76147400	-0.06481700
C	-2.41725600	0.92345400	2.11304500
H	-2.82095500	1.64241600	1.40686200
C	3.31273000	2.50353600	-0.52665000
C	-3.25335500	-0.03215400	2.68782000
H	-4.30534000	-0.05933600	2.41834200
C	-0.14864400	1.98249100	1.78024800
H	0.83474200	1.97034300	2.27973900
C	4.02703600	4.55132800	-0.94194900
H	4.26551900	5.59140600	-0.78049000

C	-0.69378700	-2.25208400	0.89897500
H	-1.77470900	-2.26865500	1.02246300
H	-0.35937400	-3.03271500	0.21099000
H	-0.20177400	-2.39264500	1.86123700
C	-0.55812500	0.08319700	3.40255800
H	0.49287300	0.12287300	3.67920600
C	-0.80978700	3.99136300	3.11987600
H	-1.47391000	4.85749200	3.05947300
H	-1.24005500	3.28928300	3.83717100
C	-2.74195500	-0.94131000	3.62272900
H	-3.39396100	-1.68713400	4.06693700
C	3.82613400	1.63764000	-2.83989200
H	3.04860900	1.83209400	-3.57948300
C	-0.30352000	5.42557900	0.49083000
C	-1.39707500	-0.87463600	3.98501600
H	-0.99441000	-1.56637300	4.71930900
C	3.16397700	4.15438400	1.38219000
H	3.10934500	3.27114600	2.01592200
B	2.76600200	1.17422200	0.18673100
H	3.51470500	0.31273900	-0.28175300
H	2.97944600	1.25270900	1.37473700
C	0.63116400	3.66089600	-1.50209100
H	0.99753600	2.98663400	-2.26489100
C	0.61882800	5.04639000	-1.73185600
C	0.15686600	5.91255600	-0.73977000
H	0.15293400	6.98433700	-0.91781800
H	0.97488300	5.43817100	-2.67917800
H	-0.66070600	6.10778700	1.25477200
H	4.81056200	1.69510400	-3.30461300
H	3.67687800	0.65072900	-2.40844200
H	2.20021000	4.66617900	1.37131100
H	3.94320300	4.82187100	1.75450100
N	4.19777900	3.91111200	-2.06430500
H	1.04175800	0.56691900	-1.58695200
B	0.68520700	0.30102400	-2.93473300
C	-0.64194800	-0.44358400	-2.63501300
H	0.55866000	1.37903200	-3.44526600
H	1.56362000	-0.40541300	-3.33582100
N	-0.78615400	-1.80370400	-2.43668500
N	-1.85424000	0.05790900	-2.30598000
C	-2.02508100	-2.04157000	-1.96161300
C	0.29049900	-2.78742500	-2.59228500
N	-2.70980100	-0.92347400	-1.85882100
C	-2.29713100	1.44269000	-2.39726900
H	-2.41173900	-3.00594600	-1.66787200
H	1.07616500	-2.59635600	-1.85840300
H	-0.12798500	-3.78358200	-2.44604200
H	0.71822800	-2.70120900	-3.59053500
H	-3.14440700	1.51009300	-3.08209700
H	-2.60472900	1.80126000	-1.41488700

H	-1.46036100	2.03560800	-2.75904700
Zn	3.79153400	-1.45226400	0.28305500
I	4.31364200	-2.45301600	-2.02141500
I	4.90700100	-1.51695900	2.55228900
Zn	-4.37072100	-0.75294000	-0.54733400
I	-5.54248000	1.45287800	-0.39335700
I	-4.80611900	-3.08740500	0.30156700
H	0.17072800	4.32615700	3.49999400

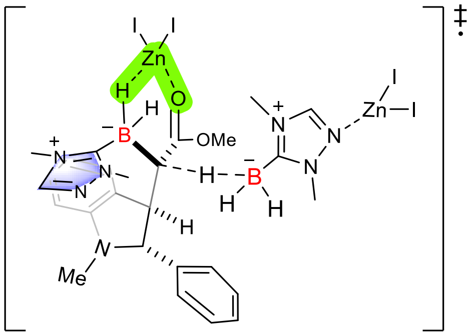

TS-B-minor-Me

O	-0.59250600	1.39973700	1.52521100
O	0.36496900	-0.22759700	0.31383400
N	-5.26800900	0.78858400	-0.38253000
N	-4.31377500	-1.41258900	-2.32807000
N	-4.31702700	-2.35286100	-0.42038000
C	-0.65493800	0.47811200	0.55004300
C	-3.82847300	0.62512800	1.40718600
C	-4.46183100	3.12321500	-0.77775000
C	-5.13438800	0.38247000	0.93977100
C	-3.02133600	1.29444300	0.30558400
C	-3.45634000	0.25107800	2.68955600
H	-2.44600900	0.43407600	3.03841900
C	-1.88590000	0.40810800	-0.21568700
C	-5.08208700	3.73251500	0.32356600
H	-5.32859800	3.13610600	1.19743600
C	-3.55845900	-1.56615400	-1.22891600
C	-5.38239200	5.09361400	0.30167000
H	-5.86349400	5.55357000	1.16020900
C	-4.11989900	1.64312700	-0.75801800
H	-3.77541300	1.36445900	-1.75722400
C	-5.49698600	-2.60013100	-1.06537200
H	-6.29391100	-3.19393900	-0.64464200
C	0.56191900	1.33705700	2.39855200
H	0.64645300	0.33669800	2.82863300
H	0.37490400	2.08287500	3.16998800
H	1.47509000	1.58073700	1.85463900
C	-4.15348800	3.90150000	-1.89793000
H	-3.66416700	3.43849100	-2.75033700
C	-6.56552700	1.01339100	-0.98711600
H	-7.12415800	1.83456200	-0.51497800
H	-7.16287500	0.09875200	-0.93746700

C	-5.06609300	5.86543800	-0.82046000
H	-5.30031400	6.92591900	-0.83614800
C	-3.99774200	-0.65700800	-3.53056200
H	-2.99094400	-0.25457700	-3.43570800
C	-4.44958100	5.26656200	-1.91949900
H	-4.20040500	5.85915900	-2.79521500
C	-3.92392600	-2.86975400	0.89392500
H	-4.81311800	-3.25857300	1.38852600
B	-2.06914700	-1.04799100	-0.94738200
H	-1.45874200	-1.10791000	-1.98407200
H	-1.62428000	-1.93694900	-0.20771300
C	-6.08517000	-0.22705400	1.76351000
H	-7.10067700	-0.39602800	1.41989400
C	-5.69605700	-0.60067300	3.05845600
H	-6.42554800	-1.07137800	3.71187500
C	-4.40032800	-0.37063400	3.52438000
H	-4.12064800	-0.66641600	4.53004200
H	-2.58885700	2.22854400	0.66830800
H	-4.06398300	-1.31947400	-4.39565300
H	-4.71419400	0.15921500	-3.64205700
H	-3.17704700	-3.65610200	0.77772000
H	-3.50604700	-2.06576400	1.49455400
N	-5.52776500	-2.03411600	-2.24186600
H	-1.42213400	1.22608800	-1.40124600
B	-0.94669700	1.94663500	-2.50000700
C	0.53943100	1.53031500	-2.31034500
H	-1.49335700	1.46454300	-3.45725800
H	-1.19142700	3.08806800	-2.22358100
N	1.19804600	0.42648300	-2.80552000
N	1.41916000	2.06683800	-1.43857400
C	2.38675600	0.31103100	-2.17416400
C	0.67879500	-0.48326400	-3.82636100
N	2.55955000	1.30514000	-1.33155500
C	1.21793800	3.27908900	-0.65408000
H	3.09904000	-0.48717500	-2.32071800
H	-0.07304000	-1.13419700	-3.38046300
H	0.24193300	0.10999900	-4.62999800
H	1.50184700	-1.08924400	-4.20193800
H	0.83556200	4.05923900	-1.31256800
H	0.49370300	3.09215000	0.14123100
H	2.17213800	3.57594100	-0.22466000
Zn	0.17650700	-2.26234800	0.31047300
I	1.54094100	-3.36690000	-1.52244800
I	-0.41844200	-2.86922400	2.72678700
Zn	4.21165400	1.34771500	-0.01293600
I	4.26954300	3.06890400	1.81503800
I	5.79156300	-0.42119500	-0.83045400
H	-6.42827100	1.26171400	-2.04250400

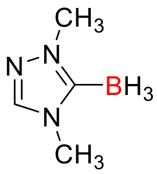

2d

C	-0.01644100	0.60768000	-0.00005400
C	-0.50057600	-1.55934000	0.00003700
H	-1.08993500	-2.46417800	0.00006800
N	0.80192100	-1.52039100	0.00000500
N	1.07998400	-0.17383400	-0.00006600
N	-1.03972100	-0.29958300	0.00000100
C	2.46948100	0.25624300	0.00007300
H	2.96930800	-0.13038100	-0.89076700
H	2.47973500	1.34472300	-0.00102900
H	2.96864000	-0.12847400	0.89212600
C	-2.44965200	0.06760300	0.00002200
H	-2.66861300	0.66769400	-0.88525700
H	-3.05333300	-0.84125800	0.00011500
H	-2.66854400	0.66782600	0.88522900
B	-0.20063800	2.18926300	-0.00007600
H	-0.85916000	2.45487300	0.99425100
H	0.87262600	2.75158500	-0.00034700
H	-0.85968800	2.45481900	-0.99406100

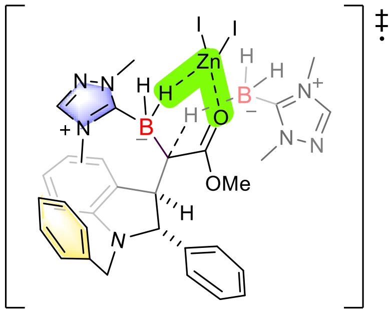

TS-B-major'

O	1.15987200	2.09719900	-1.61625300
O	1.95344700	0.09184700	-1.01779400
N	-3.28077400	1.60920900	-0.39679900
N	-0.17688000	0.12046900	3.96253300
N	-1.63316300	-0.87633700	2.77647400
C	1.17133700	1.04927800	-0.77682100
C	-1.88026200	2.17961400	1.33660800
C	-1.71553300	1.50895000	-2.31463800
C	-3.21661000	1.95287800	0.95075500
C	-0.97584200	1.94158200	0.14575000
H	-0.72948000	2.91417900	-0.29850000
C	0.32879200	1.18130600	0.40350400
C	-1.97436600	2.79324900	-2.81270700
H	-2.35904100	3.55332600	-2.13847000
C	-0.43161700	-0.24213000	2.69343300
C	-4.72300600	-0.41494400	-0.72339500

C	-1.75239300	3.08795300	-4.15674100
H	-1.95679500	4.08704000	-4.53200900
C	-1.93761100	1.19594300	-0.85099100
H	-1.80533500	0.11287800	-0.70375500
C	-2.04908200	-0.82033700	4.07878400
H	-2.98240500	-1.23769100	4.42398900
C	1.88398900	1.95624000	-2.85550700
H	1.62403400	2.83977700	-3.43725000
H	2.95765600	1.91909400	-2.66867500
H	1.56138600	1.05433100	-3.37617000
C	-1.24773600	0.52231400	-3.18809500
H	-1.03857500	-0.47547700	-2.81277800
C	-4.47271300	1.05093200	-1.03244800
H	-5.32993900	1.65896000	-0.72613500
H	-4.35216100	1.19106900	-2.11119900
C	-1.27666100	2.09844200	-5.02472900
H	-1.10961700	2.32768400	-6.07335500
C	0.97729900	0.84228400	4.47525100
H	0.70268500	1.87970600	4.67652700
C	-4.25786100	2.12016900	1.86877900
C	-5.62496300	-0.80221300	0.27687300
H	-6.21284600	-0.04733700	0.79130800
C	-1.02816800	0.81510300	-4.53762400
H	-0.66441000	0.03636900	-5.20205900
C	-2.37722900	-1.48272800	1.67369600
H	-1.68249400	-1.89857300	0.94510100
C	-5.77843700	-2.14802100	0.61688100
H	-6.48707900	-2.43270100	1.38990300
C	-5.02373000	-3.12543600	-0.03645300
H	-5.13611000	-4.17216500	0.23123100
C	-3.99182500	-1.40837600	-1.39162900
H	-3.30392900	-1.12760900	-2.18319500
C	-4.13033300	-2.75264600	-1.04466700
H	-3.53540500	-3.50207100	-1.55726100
B	0.51453700	-0.08480200	1.41030500
H	1.65378500	-0.12037500	1.80657800
H	0.28074800	-1.13250900	0.78652200
C	-1.58400300	2.63182700	2.61228100
H	-0.55603800	2.85030600	2.88064600
C	-2.62083500	2.79774200	3.54483200
C	-3.93916600	2.53889100	3.16762100
H	-4.74040700	2.66449400	3.89084500
H	-2.39602200	3.12479800	4.55495200
H	-5.28942600	1.93808300	1.59052900
H	1.30402700	0.35863700	5.39633400
H	1.76289600	0.81518000	3.72471300
H	-3.00789400	-0.73384900	1.20000600
H	-3.00629800	-2.28103300	2.06574400
N	-1.17466000	-0.21636300	4.83440100
H	1.11330900	2.23098500	1.06980400

B	1.87734900	3.29082600	1.61655200
C	3.04527600	3.33491700	0.58957100
H	1.13705100	4.23636200	1.58315300
H	2.18135500	2.85288700	2.69448100
N	4.11295100	2.47955800	0.48939900
N	3.19279900	4.14153200	-0.48741000
C	4.81634600	2.81250900	-0.63601300
C	4.36584800	1.32630500	1.35014500
N	4.27853500	3.82591800	-1.25982000
C	2.24728000	5.14485900	-0.94819100
H	5.70766700	2.29436700	-0.95633900
H	3.70442900	0.50250900	1.07608100
H	5.40152400	1.01016800	1.22181400
H	4.19105500	1.60993900	2.38783500
H	2.75905100	5.77482200	-1.67461100
H	1.38915800	4.64826000	-1.40995200
H	1.90678300	5.73709600	-0.09874500
Zn	1.66401900	-1.80514600	-0.30071500
I	3.78067400	-2.66905800	0.82473600
I	-0.05675300	-3.10748300	-1.72330600

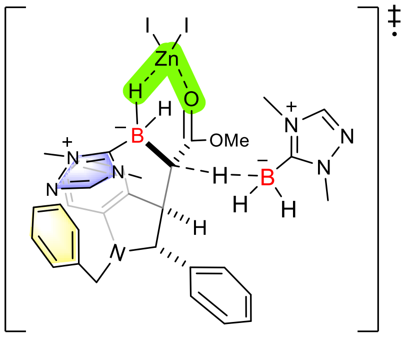

TS-B-minor'

O	-0.96605100	1.69466200	-2.28755800
O	-2.28015700	1.07959000	-0.58070600
N	3.41014400	-0.51863600	-0.76118200
N	1.12514900	-2.61716300	0.86722500
N	1.77862500	-1.16131200	2.27442200
C	-1.12041800	1.13193000	-1.07283400
C	1.48257000	-0.66035000	-2.01943800
C	3.50579700	1.97117500	-0.91017300
C	2.71469600	-1.26308000	-1.70992600
C	1.33677600	0.63908000	-1.24698300
C	0.61691900	-1.24318700	-2.93168400
H	-0.33826900	-0.77873100	-3.15142400
C	0.07724500	0.70218000	-0.37600700
C	3.91164100	2.10994500	-2.24604700
H	3.64379700	1.33997900	-2.96537900
C	0.87028600	-1.36808200	1.27985100
C	5.24322300	-1.06891800	0.84584300
C	4.65807600	3.21555200	-2.64889500
H	4.96652700	3.31044100	-3.68621100

C	2.70429200	0.75642100	-0.47669400
H	2.52680400	0.83871700	0.59899000
C	2.53762400	-2.29474900	2.39378700
H	3.36177300	-2.39094000	3.08318200
C	-2.18695300	2.03932800	-2.98202600
H	-2.80576400	1.14799800	-3.10867200
H	-1.86357200	2.42834500	-3.94738300
H	-2.74603700	2.79222900	-2.42204400
C	3.86797600	2.95738300	0.01134000
H	3.53990500	2.86781300	1.04308500
C	4.84789000	-0.64475600	-0.55658700
H	5.33287600	0.31111900	-0.78822000
H	5.23435700	-1.36764800	-1.27916100
C	5.01110000	4.20024100	-1.72015600
H	5.59295900	5.06195800	-2.03422400
C	0.42529200	-3.39237100	-0.15334300
H	-0.26870800	-2.74746700	-0.68482100
C	5.57248300	-2.39836500	1.13324800
H	5.52729100	-3.14240000	0.34256700
C	4.61351600	4.06928600	-0.38960200
H	4.88019600	4.83097400	0.33747900
C	1.87663000	0.02222200	3.12605200
H	1.32692300	-0.14304700	4.05628400
C	5.95284600	-2.77912500	2.42219000
H	6.21228000	-3.81400100	2.62560400
C	5.99597500	-1.83075600	3.44788600
H	6.29577500	-2.12278400	4.45015500
C	5.30185200	-0.12197600	1.87797100
H	5.07652400	0.91814900	1.65612000
C	5.66770200	-0.49921500	3.17211200
H	5.71761900	0.24751900	3.96010300
B	-0.28559800	-0.36324100	0.81451200
H	-0.69560000	0.19103500	1.80440200
H	-1.17632300	-1.13479700	0.42509100
C	3.08505700	-2.46638700	-2.31983700
H	4.01983600	-2.96022000	-2.07545300
C	2.20794700	-3.03852400	-3.25298300
H	2.48993700	-3.97083100	-3.73464700
C	0.98281600	-2.44462600	-3.55854900
H	0.30940000	-2.90964600	-4.27083000
H	1.28843900	1.46810500	-1.95621000
H	1.16234400	-3.78476200	-0.85144100
H	-0.11816100	-4.20682800	0.32955500
H	2.92954900	0.20649400	3.34120600
H	1.44885900	0.87925200	2.60935800
N	2.15865300	-3.20789000	1.54317900
H	0.30446600	1.92932900	0.39184600
B	0.40327600	3.16635200	1.12415000
C	-1.07600600	3.60325200	0.92080000
H	0.69368800	2.83144500	2.24295900

H	1.20366100	3.79862300	0.49531800
N	-2.18098100	3.27955200	1.65973800
N	-1.59452000	4.23378100	-0.15705600
C	-3.28693300	3.68547300	0.96595200
C	-2.16242100	2.56618300	2.93207500
N	-2.96316400	4.28475900	-0.14896500
C	-0.84714000	4.79811800	-1.27105600
H	-4.29706000	3.51821800	1.30709000
H	-1.84923000	1.53489200	2.76873900
H	-1.47056800	3.06562800	3.61250600
H	-3.16937700	2.56450500	3.34705600
H	-0.05459900	5.44350500	-0.88954900
H	-0.40281100	3.99673100	-1.86540500
H	-1.54833900	5.37267400	-1.87460700
Zn	-2.98512700	-0.69044800	0.12611000
I	-4.43230000	-0.43050400	2.20201400
I	-3.22456400	-2.18627200	-1.95357500

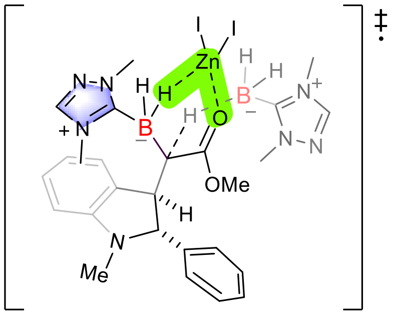

TS-B-major-Me'

O	0.50639000	2.57553300	0.05158200
O	-1.28943800	1.25690200	0.26659500
N	3.85579300	-0.45837600	1.05664200
N	0.68668300	-2.85716900	-2.66586600
N	1.25910800	-3.30988600	-0.66898400
C	-0.08707400	1.37982500	-0.08811200
C	3.12042400	-0.53474800	-1.12388700
C	2.53223000	1.42894200	1.96836600
C	4.03537200	-1.05410300	-0.18763900
C	2.21210000	0.45729700	-0.42811900
H	2.54952200	1.47190500	-0.67112500
C	0.71538800	0.35375100	-0.73811900
C	3.47028000	2.44900000	1.75844800
H	4.21892300	2.32769900	0.98057900
C	0.63263400	-2.35966300	-1.41922200
C	3.44977900	3.59970100	2.54416700
H	4.18119800	4.38484100	2.37267600
C	2.53332500	0.19514900	1.09131900
H	1.78109800	-0.50638700	1.48819000
C	1.68554600	-4.29959900	-1.51457500
H	2.22507500	-5.17407300	-1.18476000
C	-0.19829300	3.57879400	0.81177000

H	0.52439300	4.38064500	0.95834900
H	-1.06685500	3.93618300	0.25716800
H	-0.50657800	3.17045900	1.77458800
C	1.59053100	1.56912700	2.99195000
H	0.86174500	0.78225300	3.16530400
C	4.37136900	-1.04664400	2.27312600
H	5.44330300	-1.23862200	2.17216400
H	4.23095000	-0.34077100	3.09482500
C	2.49555600	3.74150900	3.55836200
H	2.48256600	4.63735800	4.17278700
C	0.18924800	-2.25610400	-3.89383400
H	1.02964300	-1.88072100	-4.48098400
C	4.96952800	-2.02433300	-0.56302100
C	1.56993200	2.72222200	3.78266500
H	0.83021200	2.81688500	4.57265400
C	1.47871200	-3.28414300	0.77580400
H	0.68504300	-2.71317600	1.25790500
B	-0.11626800	-1.03398000	-0.91848500
H	-1.05084400	-0.85781600	-1.66109800
H	-0.55584900	-1.38799700	0.18532900
C	3.18860200	-0.91566600	-2.45444500
H	2.50828600	-0.47696200	-3.17670100
C	4.13091200	-1.88016900	-2.84849700
C	5.00066200	-2.42896900	-1.90481400
H	5.72002900	-3.18354200	-2.21128000
H	4.17640100	-2.20315000	-3.88369200
H	5.65798800	-2.45184400	0.15857600
H	-0.34876700	-3.01805800	-4.45886600
H	-0.46840300	-1.43107900	-3.63356400
H	2.44749000	-2.83105900	0.98677200
H	1.45880700	-4.30811300	1.15185400
N	1.34633500	-4.05236000	-2.74780000
H	0.68317400	0.92395100	-2.09490600
B	0.66353300	1.57227900	-3.35486700
C	-0.33450100	2.72273200	-3.03512600
H	1.80078200	1.92056700	-3.52453300
H	0.22325800	0.71744900	-4.07741000
N	-1.70090800	2.65120800	-2.94205900
N	-0.06393600	3.99176100	-2.65099500
C	-2.15766400	3.86059500	-2.49495400
C	-2.50673700	1.45109100	-3.15144100
N	-1.18017600	4.70579900	-2.30534600
C	1.25479000	4.56719900	-2.44230400
H	-3.20152500	4.07768900	-2.32572600
H	-2.40482900	0.77931500	-2.29773300
H	-3.55283200	1.74166200	-3.25109400
H	-2.17249500	0.94916700	-4.05959700
H	1.13769600	5.64796500	-2.37444100
H	1.68093700	4.16965800	-1.51712300
H	1.90066500	4.30305100	-3.27985600

Zn	-2.12540100	-0.52989500	0.81134300
I	-4.30954000	-0.93585500	-0.43422900
I	-1.48612500	-1.16318200	3.23071800
H	3.87388000	-1.99406100	2.54554200

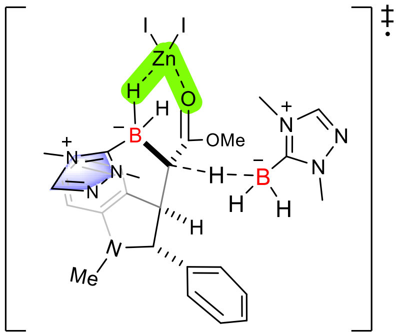

TS-B-minor-Me'

O	0.30295000	1.09368500	-2.35785000
O	-1.44429200	1.09655500	-0.95212900
N	3.50118400	-1.21442000	0.89170400
N	1.44619600	-0.98707400	3.20317800
N	0.74685300	-2.58394800	1.98446900
C	-0.21499700	0.87379300	-1.13353200
C	2.19746900	-1.36315300	-0.99948100
C	4.47139200	0.91249000	0.01357800
C	3.05035000	-2.05613300	-0.11931100
C	2.10514100	0.09052800	-0.56656300
C	1.58202000	-2.02180500	-2.05221100
H	0.91284800	-1.48314300	-2.71436200
C	0.70281300	0.48332000	-0.08204900
C	5.22047500	0.38950200	-1.05193500
H	4.90843700	-0.53965400	-1.52023900
C	0.71719000	-1.22969500	2.10258600
C	6.35827700	1.05219900	-1.50956800
H	6.92938800	0.63581600	-2.33467600
C	3.23154800	0.19318600	0.51931000
H	2.86298200	0.73261700	1.39525900
C	1.51760100	-3.07482900	3.00171100
H	1.72004100	-4.12542100	3.14311100
C	-0.64701500	1.42507500	-3.39708900
H	-1.39892900	0.63684400	-3.47791700
H	-0.05526400	1.49262000	-4.30988100
H	-1.13591800	2.37661400	-3.17666400
C	4.88930700	2.10562300	0.61123800
H	4.31047600	2.52144400	1.43111700
C	4.66311100	-1.53633500	1.69420200
H	5.59528300	-1.56680100	1.11097300
H	4.51820300	-2.50156100	2.18732700
C	6.76474400	2.24812700	-0.90982800
H	7.65136300	2.76380700	-1.26749800
C	1.74319800	0.30318100	3.80701400
H	1.23821700	1.08101200	3.23665100

C	6.02670400	2.77376200	0.15101900
H	6.33539100	3.70210800	0.62347300
C	0.04895300	-3.37510600	0.96655700
H	0.45506300	-4.38575500	0.98117900
B	-0.09046200	-0.19841100	1.18237600
H	-0.57984700	0.63232500	1.90698800
H	-1.01351600	-0.91146300	0.75623200
C	3.30993600	-3.41728800	-0.30156000
H	3.98554200	-3.95427000	0.35664400
C	2.68320300	-4.07212800	-1.37285400
H	2.87753700	-5.12979400	-1.52923900
C	1.82617200	-3.39307800	-2.24035900
H	1.34785900	-3.91924700	-3.05976300
H	2.37452400	0.74676300	-1.39602700
H	1.39286200	0.30232400	4.84095000
H	2.82216600	0.46941300	3.78894900
H	-1.02154300	-3.39093300	1.17615000
H	0.21396500	-2.93980400	-0.01589700
N	1.96487500	-2.11785700	3.76957500
H	0.96391000	1.81885600	0.45631000
B	1.15811800	3.14962000	0.93477800
C	-0.06837400	3.76200700	0.19717600
H	1.04861900	3.05149100	2.12876300
H	2.22663900	3.45672500	0.48325700
N	-1.37489700	3.81059800	0.60217100
N	-0.12447000	4.17949900	-1.08797500
C	-2.13318300	4.20570800	-0.46386600
C	-1.86690400	3.40063300	1.91334200
N	-1.39767900	4.45005200	-1.51547400
C	0.99291500	4.29235400	-2.01326600
H	-3.20782500	4.29879700	-0.42753300
H	-1.78047200	2.31861900	2.01469700
H	-1.28135800	3.89724200	2.68837900
H	-2.91706800	3.67878100	1.99596800
H	1.83102500	4.77616300	-1.51084100
H	1.29949800	3.29857000	-2.34883700
H	0.65816800	4.89022400	-2.85994700
Zn	-2.58539700	-0.37381100	-0.13388900
I	-4.39660200	0.47317200	1.43416000
I	-2.54447700	-2.27701500	-1.86505300
H	4.77658800	-0.78084400	2.47588500
